# 900°C thermal-force coupling carbonization tailoring hard carbon microstructures enables high performance sodium-ion pouch batteries

**DOI:** 10.1038/s41467-026-77440-0

**Published:** 2026-09-08

**Authors:** Zongfu Sun, Huawei Liu, Yihao Cheng, Huan Li, Zhiyuan Han, Ying Tang, Xiang Zhang, Chunsheng Shi, Fang He, Yuting Yang, Biao Chen, Chunnian He

**Affiliations:** 1 School of Materials Science and Engineering and Tianjin Key Laboratory of Composite and Functional Materials, Tianjin University, Tianjin, PR China; 2 School of Energy (National Industry-Education Platform for Energy Storage, Tianjin University, Tianjin, PR China; 3 School of Materials Science and Engineering, Collaborative Innovation Center of Materials Science, Nankai University, Tianjin, PR China; 4 Tsinghua Shenzhen International Graduate School, Tsinghua University, Shenzhen, PR China; 5 State Key Laboratory of Precious Metal Functional Materials, Tianjin University, Tianjin, PR China; 6 Department of Economics, University of New Mexico, Albuquerque, NM USA

**Keywords:** Energy, Batteries, Batteries, Batteries

## Abstract

Hard carbon materials are promising candidates for advancing large-scale energy storage in sodium-ion batteries. However, a fundamental trade-off often exists between sodium storage sites and fast diffusion kinetics in hard carbon. Herein, we overturn this long-standing constraint through a machine learning design paradigm and low-temperature thermal-force coupling carbonization that enables the microstructure in hard carbon of both abundant sodium storage sites and rapid Na^+^ diffusion. Benefiting from this low-temperature construction, the sodium-ion pouch cell exhibits a high specific energy (based on the entire cell) of 208.1 Wh kg^−1^ at 1.5-4.3 V. Besides, the required carbonization temperature reduces from 1300 °C to 900 °C, leading to an energy consumption reduction of 49.7% and a carbon emission reduction of 47.5%. This work not only provides a double-field regulation strategy to prepare high-performance hard carbon under low temperature, but also contributes a viable pathway toward sustainable sodium-ion batteries.

## Abstract (web summary)

Hard carbon is promising for sodium-ion batteries but faces a trade-off between storage capacity and ion transport. Here, authors use machine learning and low-temperature carbonization to achieve fast sodium storage while reducing energy use and emissions.

## Introduction

Sodium-ion batteries (SIBs) offer significant potential for advancing large-scale energy storage in the context of global carbon neutrality solutions, due to their lower cost and higher safety compared to lithium-ion batteries^1–5^. However, the practical application of SIBs is majorly hindered by the limitations of the hard carbon (HC) negative material, which struggles to simultaneously exhibit the high sodium storage sites and fast diffusion kinetics^6–8^. HC with long-range ordered carbon layers and nanopores provides abundant sodium storage sites, its diffusion kinetics are inherently constrained by low defect (*I*_*D*_*/I*_*G*_), low specific surface area (SSA), and low interlayer spacing (*d*_*002*_)^9,10^. While appropriately increasing the *I*_*D*_*/I*_*G*_, SSA, and *d*_*002*_ of HC improves the diffusion kinetics of Na^+^, this inevitably reduces the number of ordered carbon layers and nanopores, thereby limiting the sodium storage sites^11,12^. The lack of clarity regarding the relationship between the carbonization process, microstructure, and performance hinders the rational design of high-performance HC^13^. Furthermore, the HC preparation still depends on the high-temperature (1200–1800 °C) thermal-field-derived carbonization strategy, which is difficult to trade off the advantages of various microstructures^14,15^. Therefore, shifting the design paradigm and developing controllable preparation strategies of HC microstructures are crucial for overcoming the trade-off between reversible sodium storage sites and Na^+^ diffusion kinetics.

Recently, structure modification strategies combined with high-temperature carbonization (pre-oxidation, molecular cross-linking, and heteroatom doping) are widely used to construct Na^+^ diffusion pathways and sodium storage sites, achieving high initial Coulombic efficiency (ICE) and reversible plateau capacity of HC^16–18^. Due to the inability of these modification strategies to significantly suppress high-temperature-driven structure graphitization, the Na^+^ diffusion kinetics often fail to match the abundance of storage sodium sites, resulting in a bottleneck characterized by abundant sites but slow diffusion^19^. To optimize the carbonization process, emerging rapid carbonization technologies (thermal shock, Joule heating, and flash carbonization) are employed to construct a wide highway in HC for Na^+^ diffusion^20–22^. These technologies prevent the compact stacking of the carbon layer structure through short-duration high-temperature treatment and ultra-rapid heating rates, thereby optimizing Na^+^ diffusion kinetics. The HC obtained through rapid carbonization typically exhibits long-range ordered carbon layers and a large-volume closed-pore structure, which further increases the sodium storage capacity^23,24^. And the improved Na^+^ diffusion rate remains insufficient due to excessive packing and the long-range diffusion of Na^+^.

The complex evolution of various microstructure features (*d*_*002*_, *I*_*D*_*/I*_*G*_, SSA, nanopores, and carbon layer) is inherent to the high-temperature carbonization, posing significant challenges to the optimization of the HC microstructure^25^. Furthermore, traditional research methods are unable to systematically elucidate the relationship between structure and performance due to the limited number of experiments and low efficiency. Although numerous studies have reached a certain consensus regarding the influence of structure on performance, it is limited to qualitative descriptions and thus fails to provide precise guidance^26^. Based on data from current literature regarding processes, structures, and performance, machine learning (ML) uncovers complex nonlinear relationships behind vast variables, revealing the key microstructure influence rules within trade-offs to provide quantifiable structure parameter thresholds^27,28^. Moreover, introducing an additional driving force during the low-temperature carbonization mitigates the impact of temperature on the structure, gradually promotes the ordered development of the structure, achieving a balance between sodium storage sites and diffusion kinetics. Importantly, transitioning from high-temperature carbonization to low-temperature carbonization represents a crucial technology challenge to achieve the optimal microstructure of HC, in response to the call for energy conservation and emission reduction.

We analyze the nonlinear relationship between complex structure parameters and electrochemical performance based on the preparation-microstructure-performance database through the ML design paradigm. It reveals that the microstructure synergy window of high-performance HC is concentrated in moderate *d*_*002*_ (0.376–0.394 nm), moderate *I*_*D*_*/I*_*G*_ (1.10–2.30), and low SSA (0.26–17.84 m^2^ g^−1^). By a thermal-force coupling preparation strategy, we obtain the HC (HC-10-900), featuring *d*_*002*_ = 0.384 nm, *I*_*D*_*/I*_*G*_ = 1.33, and SSA = 2.28 m^2^ g^−1^ under a low temperature of 900 °C with a pressure of 10 MPa. The HC-10-900 exhibits abundant Na^+^ storage sites and fast Na^+^ diffusion kinetics as expected. The pouch-type cells achieve competitive electrochemical performance, with a high specific energy (based on the entire cell) of 208.1 Wh kg^−1^ at 1.5-4.3 V. Based on batch processing in the laboratory, low-temperature thermal-force (900 °C–10 MPa) coupling carbonization significantly reduces energy consumption (from 214.2 to 107.8 kWh) and carbon emission (from 98.87 to 51.94 g) compared to traditional high-temperature (1300 °C) carbonization. Overall, this work establishes a design pattern, preparation method, and mechanistic insight for achieving high-performance HC, offering a viable pathway toward sustainable SIB technologies.

## Results

### ML reveals the relationship between microstructure and performance

**Fig. 1:**
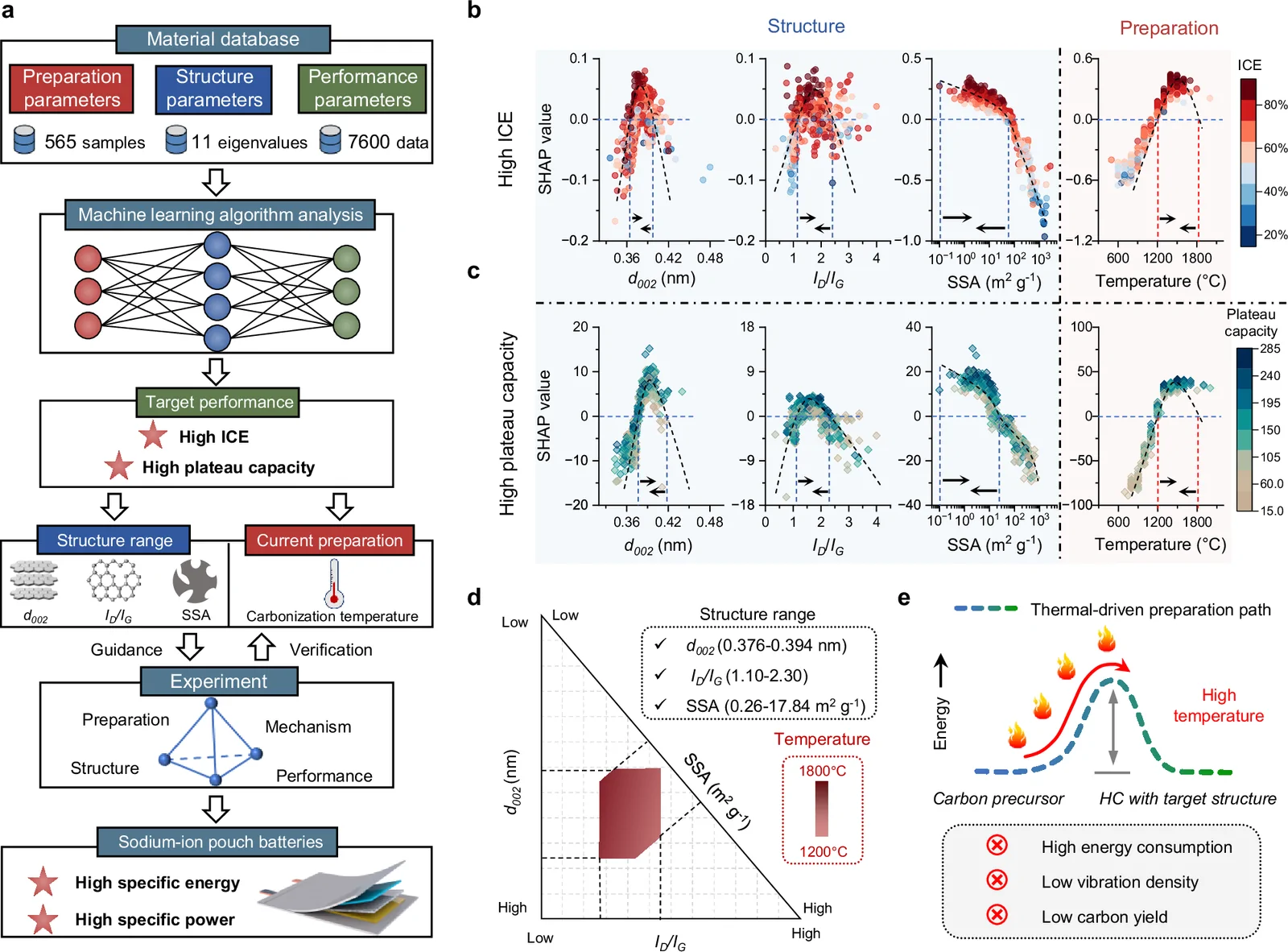
ML reveals the high-performance HC microstructures. **a** Schematic diagram of ML-driven the HC microstructure design (Blue cylinder: data volume and sample size, red circle: preparation parameters, blue circle: structure parameters, green circle: performance parameters), and the parameter database of HC for ML in Supplementary data 1. **b** Relationship of ICE with each structure feature (The ICE color scale ranges from blue to red, with values from 0 to 100%). **c** Relationship of reversible plateau capacity with each structure feature (The color scale for plateau capacity ranges from brown to green, corresponding to values from 15 to 290 mAh g^-1^). **d** The key structure features of the high-performance HC (The red shapes represent the range of structural parameters). **e** Thermal-driven preparation path of HC (The red flame indicates the carbonization temperature).

The workflow for revealing the relationship between microstructure and performance of HC is outlined in Fig. 1a, primarily comprising database construction, ML analysis, experimental validation, and mechanistic investigation. Preparation parameters (carbonization temperature, carbonization time, and heating rate), microstructure parameters (*d*_*002*_, *I*_*D*_*/I*_*G*_, SSA, total pore volume (V_total_), medium basal coherence length (*L*_*a*_)_,_ and crystal size in the stacking direction (*L*_*c*_)), and performance parameters (ICE, reversible plateau capacity, and reversible capacity) are collected in the database by data mining from the massive literature. The database contains 565 samples and 7600 data values, detecting 245 articles on HC sodium storage published between 2014 and 2025 (Supplementary Data 1). Screen out highly correlated feature values and target values through correlation analysis between variables to improve the accuracy of ML (Supplementary Fig. 1). The carbonization temperature as a typical process parameter for regulating microstructure, the *d*_*002*_, *I*_*D*_*/I*_*G*_, and SSA are crucial microstructure features for Na^+^ diffusion, and ICE and reversible plateau capacity are key manifestations of reversible sodium storage sites (Supplementary Figs. 7 and 8). In the ML algorithm calculations, the data of the database were randomly divided into training and test sets in the ratio of 8:2, and hyperparameter optimization was performed exclusively on the training set using BayesSearchCV^29^. The optimization objective was set to *R*^2^, the random seed was fixed at 42, and the number of iterations was set to 20. During the model evaluation phase, fivefold cross-validation was used as the primary framework, supplemented by tenfold cross-validation to confirm that the results were insensitive to the number of validation folds and demonstrated robustness^30^.

In calculations of ICE, the Random Forest (RF) model exhibits the highest fivefold cross-validation mean *R*^2^ (0.826), indicating strong regression performance (Supplementary Fig. 2). In calculations of reversible plateau capacity, the fivefold cross-validation mean *R*^2^ for RF (0.805) is slightly lower than that for eXtreme Gradient Boosting (XGB, 0.811), the hold-out test *R*^2^ for RF (0.794) is higher than that for XGB (0.780), resulting in a smaller generalization gap (Table S1). Besides, the ranking of features by their contribution to ICE and reversible plateau capacity remains consistent in the RF model by a cross-model Shapley Additive exPlanations (SHAP) ranking comparison (Supplementary Fig. 3). Therefore, comprehensively evaluating the *R*^2^ value, predictive performance, cross-validation stability, generalization gap, and interpretability, the RF model was selected as the final model. The data points were distributed around the reference line in the target value calculations with a small error (Supplementary Fig. 4, and Table S2). The results of the RF regression modeling algorithm were analyzed by the SHAP interpretable model^31,32^. Compared to *d*_*002*_ and *I*_*D*_*/I*_*G*_, SSA exhibits a higher degree of influence on ICE and reversible plateau capacity, and HC with a low SSA is more likely to achieve high ICE and reversible plateau capacity (Supplementary Figs. 5 and 6). Appropriately increasing *d*_*002*_ and *I*_*D*_*/I*_*G*_ within a certain range is beneficial for improving the diffusion rate of Na^+^, thereby enhancing the reversible sodium storage capacity of HC. Besides, the performance of HC under an ether electrolyte is better than that of an ester electrolyte, which is related to the composition of the SEI^33^.

The range of microstructure features for high ICE and high reversible plateau capacity was determined by lowess fitting^34,35^. HC with high ICE generally possesses a *d*_*002*_ of 0.360–0.394 nm, an *I*_*D*_*/I*_*G*_ of 1.10–2.36, and a SSA of 0.1–77.0  m^2^ g^−1^ (Fig. 1b). HC with high reversible plateau capacity typically possesses a *d*_*002*_ of 0.376–0.420 nm, an *I*_*D*_*/I*_*G*_ of 1.05–2.30, and a SSA of 0.26–17.84 m^2^ g^−1^ (Fig. 1c). Integrating these two structure parameter ranges yields a microstructure synergy range of high-performance HC characterized by low SSA (0.26–17.84 m^2^ g^−1^), moderate *d*_*002*_ (0.376–0.394 nm), and moderate *I*_*D*_*/I*_*G*_ (1.10–2.30, Fig. 1d). HC falling within this target zone consistently achieve both high ICE and high plateau capacity across recent literature reports (Supplementary Fig. 9). These findings establish that low SSA suppresses irreversible interfacial reactions, while moderate *d*_*002*_ and *I*_*D*_*/I*_*G*_ provide an optimal balance between reversible sodium storage sites and Na^+^ diffusion kinetics. The *I*_*D*_*/I*_*G*_ may compensate for the kinetic lag caused by *d*_*002*_ by providing additional active sites. The HC with this structure relies on high-temperature carbonization driven by a single thermal field (1200–1800 °C, Fig. 1e). High temperatures accelerate the formation of reversible sodium storage sites, particularly significantly reducing SSA^36,37^. Therefore, achieving the target microstructure of high-performance HC at low carbonization temperature represents a critical yet unresolved challenge, with substantial implications for reducing energy consumption and manufacturing cost.

### 900°C thermal-force coupling carbonization prepares the tailored microstructures

**Fig. 2:**
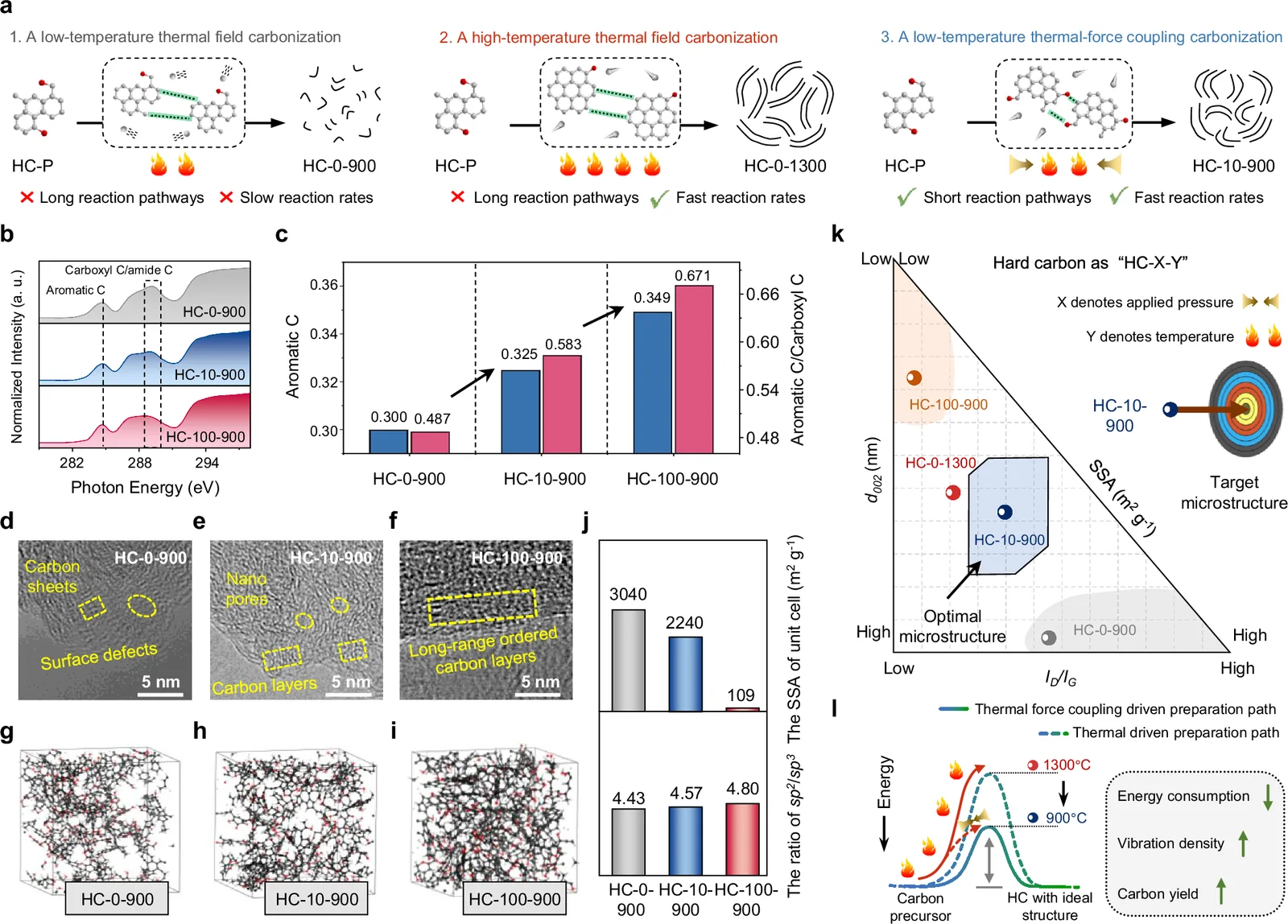
900°C thermal-force coupling carbonization prepares the tailored microstructures. **a** Schematic diagram of a low-temperature thermal-force coupling strategy for preparing HC (The gray spheres represent carbon atoms, the red sphere represents an oxygen atom, the flame represents the carbonization temperature, the brown arrow represents the pressure, and the black lines represent the microstructure of HC). **b** Near-edge X-ray absorption fine structure spectroscopy, **c** Aromatic C and Aromatic C/Carboxyl C, **d**–**f** HRTEM images, and **g**–**i** MD simulations of HC-0-900, HC-10-900, and HC-100-900 (Black represents carbon atoms, gray represents hydrogen atoms, and red represents oxygen atoms). **j** The *sp*^2^/*sp*^3^ ratio and SSA of the unit cell from MD simulations. **k** The *d*_*002*_, *I*_*D*_*/I*_*G*_, and SSA of HC-0-900, HC-10-900, HC-100-900, and HC-0-1300. **l** Carbonization temperature of the thermal-driven preparation path and thermal-force coupling-driven preparation path.

Traditional views often regard high carbonization temperature as a key process parameter, but temperature is only one of the process methods used to induce microstructural evolution. Thermal-force coupling strategies are proposed to prepare HC conforming to the microstructure synergy range at low carbonization temperatures, validating the accuracy of ML-driven design (Fig. 2a). First, glucose undergoes dehydration through pre-carbonization to yield a carbon matrix with an amorphous structure (HC-P, Supplementary Fig. 10). Under single thermal field conditions, the HC-P undergoes thermal decomposition and expansion, and the ordered cross-linking of carbon atoms requires overcoming long-range reaction pathways^38,39^ (Supplementary Fig. 11). However, the cross-linking reaction rate of carbon atoms is slow at low temperatures, easily forming highly disordered microstructures (high *d*_*002*_, high *I*_*D*_*/I*_*G*_, and high SSA). High temperature provides sufficient energy to accelerate the reaction rate compared to low temperature, which is difficult to control the ordered cross-linking rate of carbon atoms, tending to form excessively graphitized microstructures (low *d*_*002*_, low *I*_*D*_*/I*_*G*_, and low SSA). In contrast, thermal-force coupling carbonization not only compacts the material into a dense block, significantly reducing the SSA, but also promotes the slow growth of the carbon structure, keeping the *I*_*D*_*/I*_*G*_ and *d*_*002*_ within reasonable ranges (Supplementary Figs. 12 and 13). This also creates conditions for the formation of short-layer ordered carbon layers and closed-pore structures at low carbonization temperatures, thereby reducing the carbonization temperature required to achieve the target microstructure.

Thermal-force coupling was applied during carbonization to regulate the microstructure of HC, with samples denoted as HC-X-Y (X denotes applied pressure, Y denotes temperature). The morphology of HC-0-900 exhibits thin carbon flakes, while the thermal-force coupling-synthesized HC displays irregular and dense blocks (Supplementary Fig. 14). This morphological transition results from thermal-force coupling effects that promote the rearrangement of the carbon framework and interlayer slippage, which facilitates the reduction of SSA. Near-edge X-ray absorption fine structure (NEXAFS) spectroscopy reveals a progressive increase in the aromatic index with increasing pressure, indicating enlarged planar *sp*^2^ domains and the growth of nano-graphitic regions (Fig. 2b, c)^40^. High-resolution transmission electron microscopy (HRTEM) clearly shows that applied pressure promotes carbon layer stacking, and eventual evolution toward a graphite-like microstructure occurs as pressure increases (Fig. 2d–f, and Supplementary Fig. 15). Since the graphitization process of the material is accompanied by volume contraction, the application of pressure significantly reduces the Gibbs free energy, providing the thermodynamic driving force for structural ordering at low temperatures. Furthermore, the applied pressure enhances interlayer van der Waals interactions by compressing the interlayer spacing, reducing the activation energy required for relative sliding, rotation, and stacking of the carbon layers, thereby effectively compensating for the lack of thermal energy at low temperatures (Supplementary Fig. 16). Molecular dynamics (MD) simulations confirm that under the thermal-force coupling effect, the short carbon fragments accelerate cross-linking to promote the epitaxial growth of the carbon layer and form a closed-pore structure between the curved pseudo-graphite microdomains (Fig. 2g–i, and Supplementary Figs. 17 and 18)^41,42^. Meanwhile, structure variations of the unit cell exhibit consistent trends with characterization results of HRTEM (Fig. 2j). Combined with both experiments and simulations confirmed that low-temperature thermal-driven pyrolysis reactions result in high SSA, high defects, and open pore structures (Supplementary Fig. 19). In contrast, force field coupling at low temperatures promotes carbon layer stacking, surface defect toward bulk defect coupling, and closed-pore forming, leading to the transition from short-range to long-range order.

Comprehensive characterization via X-ray diffraction (XRD), Raman spectroscopy, and N_2_ adsorption-desorption isotherms tests quantified key microstructure parameters (*d*_*002*_, *I*_*D*_*/I*_*G*_, and SSA), enabling identification of HC configurations within the optimal microstructure. The *d*_*002*_ of HC-0-900, HC-10-900, and HC-0-1300 were calculated as 0.414, 0.384, and 0.380 nm, respectively (Supplementary Fig. 20). Besides, the R factor, *L*_*a*_, and *L*_*c*_ increase, and |g^o^| values decrease of HC with increasing pressure, indicating that the compression and orderliness of the carbon layer become more pronounced (Supplementary Figs. 21 and 22)^43,44^. The *I*_*D*_*/I*_*G*_ of HC-0-900, HC-10-900, and HC-0-1300 were calculated as 1.72, 1.33, and 1.05, respectively (Supplementary Fig. 23). The SSA of HC-0-900, HC-10-900, and HC-0-1300 is 436.5, 2.28, and 0.68 m^2^ g^−1^, respectively (Supplementary Fig. 24). The *I*_*D*_*/I*_*G*_ and SSA decrease progressively with increasing pressure, which is attributed to the densification of the material, where surface-exposed defects are gradually incorporated into the bulk defects. Comparing these quantitative parameters with the ML-obtained microstructure range, it reveals that HC-10-900 satisfies the combination of low SSA, moderate d_002_, and moderate *I*_*D*_*/I*_*G*_ (Fig. 2k, and Table S3). The *d*_*002*_ and SSA of HC-0-1300 align within the microstructure synergy range, yet the *I*_*D*_*/I*_*G*_ remains low due to the difficulty in controlling structure graphitization under high temperature. The thermal-force coupling strategy enables HC-10-900 carbonization at only 900 °C to achieve a targeted microstructure, which enables reducing the carbonization temperature by 400 °C compared to thermal-field-driven processing (1300 °C, Fig. 2l).

The nanopore structure of HC was researched to comprehensively analyze the microstructure. Pore size distribution tests showed that the nanopore size of HC tends to increase and then decrease as the pressure increases, while the nanopore volume decreases (Table S4). The small-angle X-ray scattering (SAXS) pattern of HC-0-900 exhibits the broadest humps around the scattering vector (Q) of 0.2 Å^−1^, demonstrating a high number of closed pores^45^ (Supplementary Figs. 25 and 26). Although the closed pores in HC-10-900 decrease, the high closed pore aperture is provided. Besides, the closed pore volume of HC-10-900 is up to 0.178 cm^3 ^g^−1^ by true density analysis of degassing with helium (Supplementary Fig. 27). The changes in open-pore and closed-pore structures indicate that the disordered carbon layers at low pressure are rearranged to form new pores, which subsequently transform into a low-porosity, ordered graphitic arrangement at high pressure. The SSA of the closed pore was calculated by subtracting the SSA measured by SAXS from the SSA measured by N_2_ adsorption/desorption^46^. HC-10-900 possesses a higher closed-pore SSA compared to other materials, which provides more bulk defects to improve Na^+^ diffusion kinetics. X-ray photoelectron spectroscopy (XPS) tests showed that the *sp*^2^/*sp*^3^ ratio increases with pressure, indicating that pressure promotes ordered cross-linking of atoms (Supplementary Figs. 28–30). HC-10-900 exhibits more C=O bonds compared to other materials, which is beneficial for enhancing the curvature of the carbon layer to form a closed-pore structure^47^. Besides, the local curvature and large interlayer spacing induced by the C=O bonds provide open diffusion pathways for Na^+^, preventing interlayer blockage.

The low-temperature thermal-force coupling carbonization (LTFC) is mainly applicable to carbon precursors that are prone to defects and high SSA at low temperatures^48^. The versatility of the strategy is further demonstrated using sucrose, cellulose, and phenolic resins as examples. In terms of microstructure, thermal-force coupling generally promotes the shrinkage and growth of carbon layers, which is not affected by precursor properties (Supplementary Fig. 31). In terms of performance, the ICE, reversible capacity, and plateau capacity of different precursor-derived HC were enhanced after LTFC (Supplementary Fig. 32). More significantly, the ICE of cellulose-derived HC at LTFC improved from 54.5% to 82.7% compared to low-temperature thermal field carbonization (LTC), the reversible capacity increased from 134.9 to 312.5 mAh g^−1^, and the storage mode of Na^+^ is transformed from a slope type to a plateau-slope type. The key to the LTFC is to promote the orderly development of carbon structures at low carbonization temperatures, which significantly improves the reversible performance of HC sodium storage.

### Performance of sodium-ion pouch batteries based on tailored HC microstructures

**Fig. 3:**
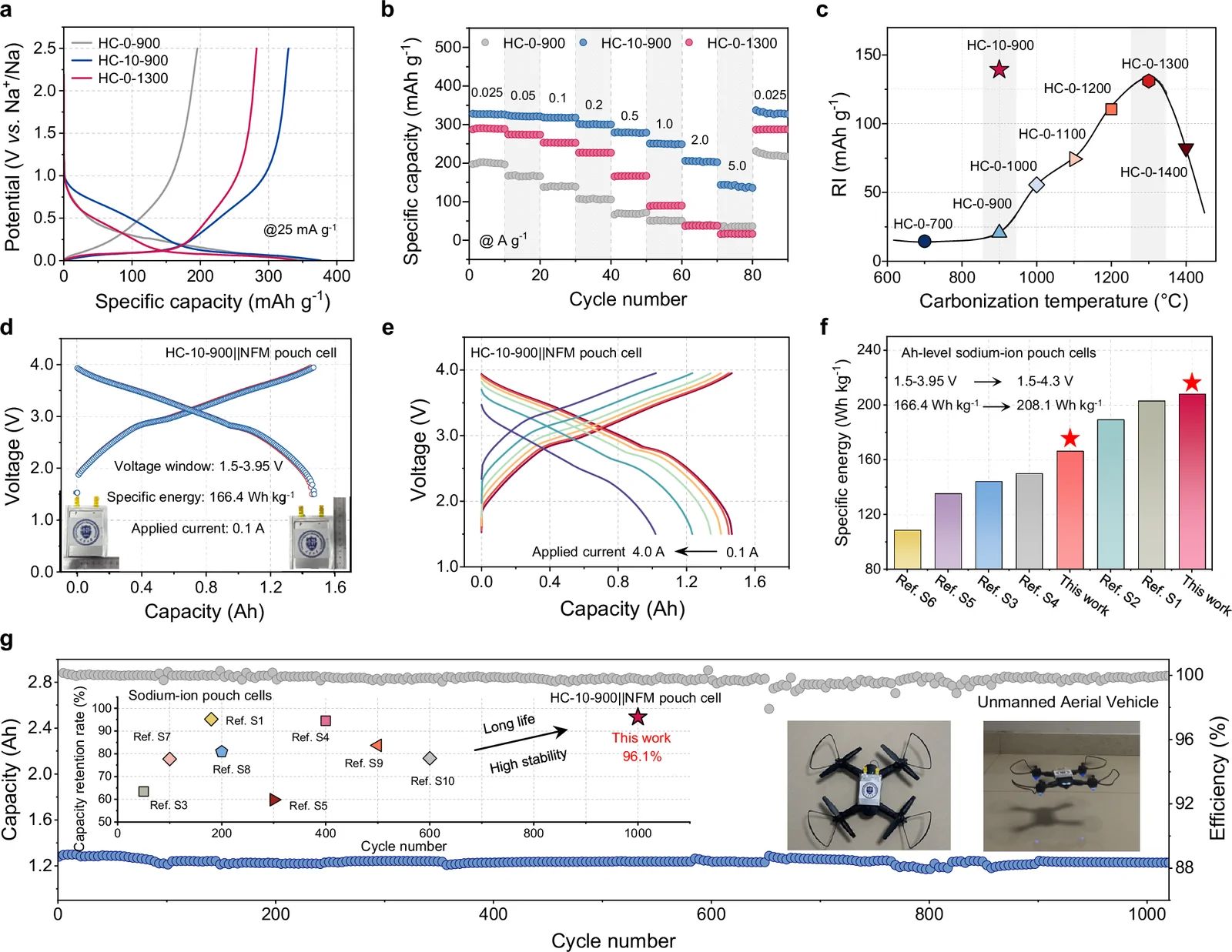
Electrochemical performance of the tailored HC microstructures. **a** Initial charging and discharging curves at 28 ± 0.3 °C. **b** Rate performance at 28 ± 0.3 °C. **c** RI of samples at different carbonization temperatures at 28 ± 0.3 °C. **d** Charging and discharging curves at 1.5–3.95 V of a sodium-ion pouch cell at 28 ± 0.3 °C. **e** Rate performance at 1.5–3.95 V of the sodium-ion pouch cell at 28 ± 0.3 °C. **f** Comparison of specific energy in sodium-ion pouch cells (Supplementary Table 9). **g** Cycling performance and stability comparison at 1.5–3.95 V of a sodium-ion pouch cell (Inset: unmanned aerial vehicle in flight) at 28 ± 0.3 °C (Supplementary Table 9).

To rigorously validate HC microstructure design, we conducted comprehensive evaluations across multiple battery types, ranging from coin half-cells and coin full-cells to Ah-level pouch cells. HC-10-900 displays a pair of sharp oxidation and reduction peaks and low potential hysteresis, suggesting the deintercalation reversibility at low potential^49^ (Supplementary Figs. 33 and 34). The initial discharge/charge capacities of HC-0-900, HC-10-900, and HC-0-1300 are 358.1/195.8, 376.6/329.3, and 340.7/282.4 mAh g^−1^, respectively, with the corresponding ICE of 54.7%, 87.4%, and 82.9% (Fig. 3a, and Supplementary Figs. 35 and 36). The HC-10-900 achieves a high reversible plateau capacity of 159.4 mAh g^−1^, which is comparable to HC-0-1300 and high to HC-0-900 (Supplementary Fig. 37). The reversible sodium storage performance of HC-10-900 is attributed to its carbon layers with wide layer spacing and abundant closed-pore structure. Besides, HC-10-900 provides the charge capacities of 318.0, 301.2, 279.3, 249.7, 205.1, and 143.5 mAh g^−1^ at 0.1, 0.2, 0.5, 1.0, 2.0, and 5.0 A g^−1^, respectively (Fig. 3b, and Supplementary Fig. 38). The rate performance of HC-10-900 is noticeably competitive to the previously reported carbon material (Supplementary Fig. 39). Thanks to the diffusion network formed by bulk defects and short-range carbon layers in HC-10-900, the diffusion kinetics of Na^+^ within the carbon layers and closed pores are enhanced. Specifically, HC-10-900 displays a charge capacity of 198.7 mAh g^−1^ after 6000 cycles at 2 A g^−1^, with a capacity retention of 87.7% (Supplementary Fig. 40). Moreover, the charging and discharging curves of HC-10-900 are almost unchanged, and the low potential plateau is maintained during the high-current long-cycle, demonstrating the structural stability.

The ICE and reversible plateau capacity of HC prepared at varying carbonization temperatures under atmospheric pressure were measured to evaluate the merits of the LTFC (Table S5). A performance factor (RI) is defined as the product of ICE and plateau capacity, which was introduced to quantify the overall reversibility of sodium storage (Fig. 3c). Under a single thermal-field processing, HC-0-900 exhibits low RI values due to the highly disordered structure at low temperatures, which provides limited reversible sodium storage sites and complex diffusion pathways. Increasing the carbonization temperature promotes the formation of ordered carbon layers and a closed-pore structure, providing abundant reversible sodium storage sites, thereby resulting in the high RI values observed for HC-0-1300 (Supplementary Fig. 41). In sharp contrast, the thermal-force coupling approach yields HC with even higher RI at only 900 °C. This finding departs fundamentally from the long-established trend in traditional HC systems, where both ICE and plateau capacity increase monotonically with carbonization temperature^50^ (Supplementary Fig. 42). By breaking this temperature-performance constraint, the thermal-force strategy enables HC-10-900 to achieve comprehensive electrochemical performance at substantially reduced temperatures (Supplementary Fig. 43, and Table S6). Moreover, compared with reported modification strategies and ultrafast synthesis techniques, LTFC also offers advantages (Table S7). This is attributed to the synergistic effect of large layer spacing, ordered carbon layers, large-sized closed pores, and abundant bulk defects.

Based on the Na storage performance of HC-10-900 exhibited in the half cell, the sodium-ion coin-type full cells were assembled to evaluate practical application. Firstly, a SIB full-cell (HC-10-900||NVP) was assembled with HC-10-900 as a negative material and Na_3_V_2_(PO_4_)_3_ (NVP) as a positive material. The NVP positive material displays a discharge plateau of around 3.35–3.38 V (Supplementary Fig. 44). Benefiting from the low-potential characteristics of HC-10-900, the HC-10-900||NVP full-cell still maintains the working plateau at *ca*. 3.26–3.37 V (Supplementary Fig. 45). The HC-10-900||NVP full cell provides a discharge capacity of 157.3 mAh g^−1^ in the 3rd cycle, and the capacity retention of the HC-10-900||NVP full cell is 81.5% after 60 cycles (Supplementary Fig. 46). To improve specific energy, a SIB full-cell (HC-10-900||NFM) was assembled with HC-10-900 as negative material and commercialized NaNi_1/3_Fe_1/3_Mn_1/3_O_2_ (NFM) as a positive material (Supplementary Fig. 47). The potential window of the HC-10-900||NFM full cell is increased to 4.0 V, and the discharge capacity is increased to 244.3 mAh g^−1^ in the 3rd cycle (Supplementary Figs. 48–50). The capacity retention of the full battery after 80 cycles is up to 89.1%, and the cycling efficiency is close to 100% (Supplementary Fig. 51).

In pursuit of high specific energy and high specific power, we further assembled an Ah-level sodium-ion pouch cell (Table S8). The HC-10-900||NFM pouch cell provided a competitive specific energy of 208.1 Wh kg^−1^ at 1.5–4.3 V based on the entire device mass (Supplementary Fig. 52). Besides, the HC-10-900||NFM pouch cell provided a discharge capacity of 1.47 Ah at 1.5–3.95 V and a specific energy of 166.4 Wh kg^−1^ (Fig. 3d). The HC-10-900||NFM pouch cell delivers discharge capacities of 1.02 Ah at 4.0 A, demonstrating competitive rate performance (Fig. 3e). The capacity and specific energy of HC-10-900||NFM pouch cell, competitive to the previously reported Ah-level SIBs (Fig. 3f, and Table S9). The HC-10-900||NFM pouch cell still delivers a specific energy of 124.3 Wh kg^−1^ at 2.0 A (Supplementary Fig. 53). The HC-10-900||NFM pouch cell exhibits capacities of 1.23 Ah after 1000 cycles at 1.0 A, with a capacity retention of 96.1% and a high average CE of 99.8% (Fig. 3g, and Supplementary Fig. 54). The cycling stability of the HC-10-900||NFM pouch cell competitive that of previous works. Collectively, these results demonstrate that HC-10-900 enables SIBs suitable for practical deployment across consumer electronics, electric mobility, and large-scale energy-storage systems, offering a viable alternative to costlier lithium-ion batteries.

### Sodium storage kinetics and mechanisms of tailored HC microstructures

**Fig. 4:**
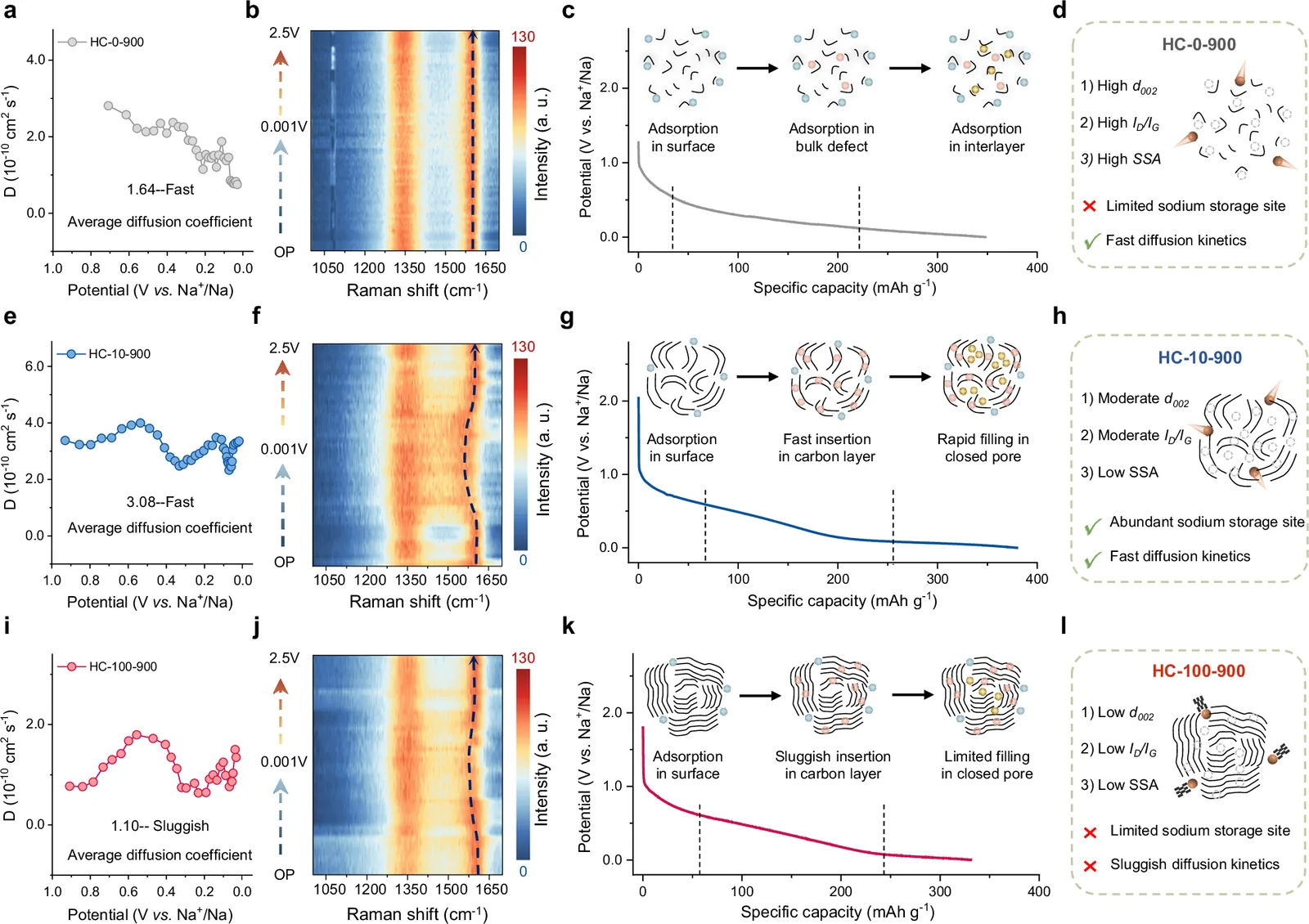
Sodium storage kinetics and mechanisms of tailored HC microstructures. The diffusion coefficient, in-situ Raman during initial charge-discharge cycle at 0.001–2.5 V in 25 mA g^−1^ (Applied temperature: 28 ± 0.3 °C, the dashed line represents the G-peak shift), sodium storage mechanism at 28 ± 0.3 °C (The circle represents the Na^+^, and the Raman intensity scale ranges from blue to red, with values from 0 to 130), and structure characteristics (The sphere represents diffusion kinetics, and the dotted circle represents the sodium storage site) of **a**–**d** HC-0-900, **e**–**h** HC-10-900, and **i**–**l** HC-100-900.

The cyclic voltammetry (CV), galvanostatic intermittent titration technique (GITT), and electrochemical impedance spectroscopy (EIS) were conducted to investigate the Na^+^ diffusion kinetics on the HC electrode. The reduction peak and oxidation peak of HC-10-900 show a higher current response than those with HC-0-900 and HC-100-900, demonstrating diffusion kinetics and reversibility of Na^+^ storage^51^ (Supplementary Figs. 55–57). Moreover, HC-10-900 provides a high diffusion-controlled capacity contribution at different sweep speeds, indicating rapid sodium storage performance at low potentials. In the low-potential region (<0.1 V), the Na⁺ diffusivity of HC-0-900 exhibits a continuously decreasing trend, whereas thermal-force coupling synthesized HC exhibit a trend of first decreasing and then increasing (Fig. 4a, and Supplementary Fig. 58). This difference originates from thermal-force coupling promoting carbon layer stacking, which creates closed pores between the curved carbon layers, thereby achieving the insertion in carbon layer and the filling in closed pores of Na^+^^52^. The HC-10-900 possesses a high diffusion coefficient, indicating that the diffusion kinetics of Na^+^ in the low-potential plateau are significantly enhanced (Fig. 4e, i). In the charging-discharging process, HC-10-900 possessed a lower internal resistance of the electrolyte (*R*_*e*_), the electrolyte/electrode interfacial resistance (*R*_*s*_), and the charge-transfer resistance (*R*_ct_) compared to the other materials, implying robust charge-transfer kinetics^53^ (Supplementary Figs. 59–61). The above results indicate that the Na^+^ diffusion and efficient electron transport of HC-10-900 are attributable to the construction of diffusion pathways and the formation of conductive networks.

The in-situ Raman of HC-0-900, HC-10-900, and HC-100-900 were conducted to systematically reveal the sodium storage mechanisms of different microstructures. During the discharge process, the Raman spectrum of HC-0-900 shows a gradual weakening of the D peak intensity and a non-shifted G peak, indicating that Na^+^ is primarily adsorbed on surface defects, bulk defects, and the carbon layer surface^54^ (Fig. 4b–d). This is determined by the structural characteristics of the high *d*_*002*_, high *I*_*D*_*/I*_*G*_, and high SSA. The intensity of the G peak of the HC-10-900 becomes weaker, and the position is not shifted during the discharge to 0.5 V, indicating that Na^+^ is adsorbed on the surface and defects (Fig. 4f–h). The G band shifts to lower frequencies as discharge progresses toward the plateau region, proving that Na⁺ is inserted into the carbon layers, which weakens and lengthens the C-C bond^55^. As the potential continues to drop to 0.001 V, the position of the G peak remains unchanged, which implies the pore-filling behavior of Na^+^. The broad peak near the scattering vector first disappears and then returns to the initial state, demonstrating that Na⁺ filling is highly reversible during discharge and charge cycles^56^ (Supplementary Fig. 62). Moreover, when discharged to 0.001 V, the sodium characteristic peak shifted toward the sodium-metal peak, and quasi-metallic sodium clusters were observed in the HRTEM test, suggesting that sodium is stored in the closed pore in the form of a quasi-metal^57^ (Supplementary Fig. 63). The G peak of HC-100-900 exhibits a relatively weak shift and a difficult return to its initial position during the charge-discharge process (Fig. 4j–l). This is attributed to the low *d*_*002*_, low *I*_*D*_*/I*_*G*_, and low SSA restricting the diffusion kinetics of Na^+^. These results demonstrate that only HC-10-900 provides the optimal balance of interlayer spacing, defect concentration, and pore structure required for fully reversible transitions among surface adsorption, interlayer insertion, and closed-pore filling.

### Laboratory-scale cost and CO_2_ emission assessment

**Fig. 5:**
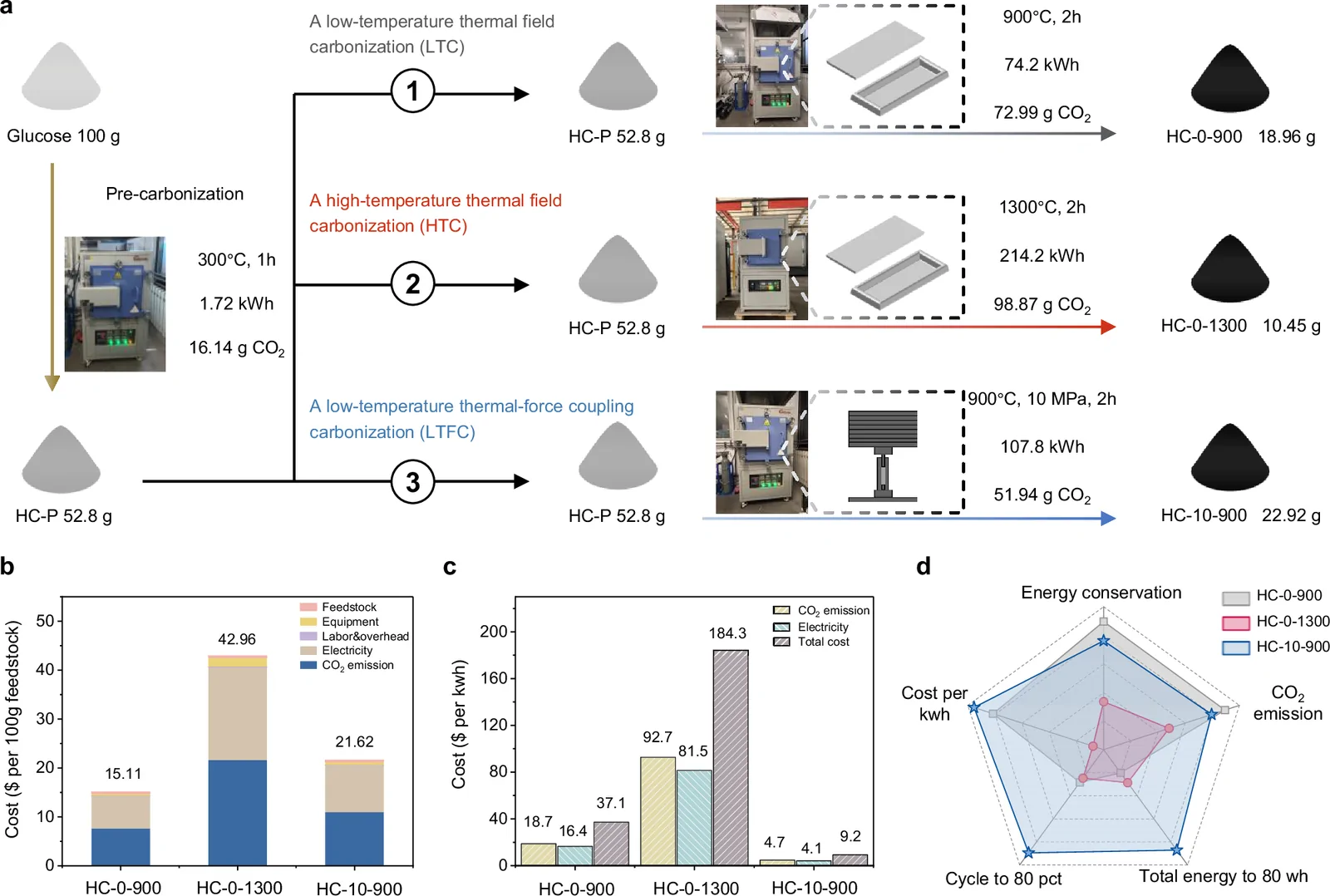
Laboratory-scale cost and CO_2_ emission assessment. **a** The preparation procedures of HC-0-900, HC-0-1300, and HC-10-900 in the laboratory. **b** Cost of preparing HC-0-900, HC-0-1300, and HC-10-900 using 100 g feedstock. **c** The cost per kWh required in the full battery systems assembled in HC-0-900, HC-0-1300, and HC-10-900, respectively. **d** Comparison of the different preparation processes.

To compare the LTC, high-temperature thermal field carbonization (HTC), and LTFC, we conducted a laboratory-scale cost and CO_2_ emission assessment. Private costs are computed using bottom-up cost accounting, and CO_2_ emissions are monetized via the Social Cost of Carbon (SCC). A levelized cost per kWh links production cost to battery performance. Based on actual laboratory preparation processes (Fig. 5a), we conducted a detailed comparison of the differences in carbon yield, energy consumption, and greenhouse gas emissions between three carbonization pathways. In the carbon emissions calculation, it is assumed that the carbon-containing gases produced during the pre-carbonization and high-temperature carbonization stages consist solely of CO_2_. Taking 100 g of glucose as an example, 100 g of glucose undergoes pre-carbonization to yield 52.8 g of HC-P, consuming 1.72 kWh of energy and emitting 16.14 g of carbon dioxide (CO_2_). Similarly, the same HC-P was maintained for each carbonization process. 52.8 g of HC-P was subsequently carbonized in batches of 8 g via three pathways due to limited mold space. During the carbonization process, the energy consumption of LTFC (107.8 kWh) is higher than that of LTC (74.2 kWh) because the mold and high-temperature alloy absorb part of the heat, yet significantly lower than that of HTC (214.2 kWh). Actual industrial production would allow the treatment of kilogram-scale glucose and HC-P in a single batch, significantly reducing the energy consumption required per kilogram of HC. The CO_2_ emissions during the carbonization process for LTFC (51.94 g) are significantly lower than those for LTC (72.99 g) and HTC (98.87 g). The low carbon emissions provide high carbon yield, with the HC-10-900 achieving a carbon yield as high as 43.4% in LTFC.

We evaluated the laboratory-scale cost and environmental performance of HC materials preparation by comparing it with LTC, HTC, and LTFC methods^58,59^. Within the current comparative framework, we assume that the three production technology pathways face similar levels of engineering complexity as they scale up. The specific calculation details of the laboratory-scale cost are presented in Tables S10 and S11. In the comprehensive evaluation of three different pathways, the primary private costs of HC production include feedstock, equipment depreciation, labor and overhead, and electricity expenses. We also include the carbon taxes of carbon emissions to capture the societal cost of HC production (energy consumption and process emissions). The total cost (private and social cost) of producing HC from 100 g of glucose via processes LTC, HTC, and LTFC is 15.11 $, 42.95 $, and 21.62 $, respectively (Fig. 5b, and Table S12). Electricity costs and the SCC account for most of the total costs, primarily due to the energy-intensive laboratory-scale batch processing. The HC obtained through the three methods was assembled into full cells, and the total energy provided for the full cell to degrade to 80% of its energy was calculated. As shown in Fig. 5c, the per kWh SCC, electricity cost, and total cost for HC-10-900 are only 4.7 $, 4.1 $, and 9.2 $, respectively, significantly lower than those for HC-0-900 and HC-0-1300 due to the higher HC output and performance (Supplementary Fig. 64, and Table S13). The radar chart clearly shows the comprehensive advantages of LTFC in energy efficiency, emission reduction, and cost reduction (Fig. 5d). Through its high-value products and efficient process, LTFC achieves the dual improvements of environmental and economic benefits in the laboratory small-scale trial stage.

## Discussion

In summary, low-temperature thermal-force coupling carbonization guided by ML-driven microstructure design was established to tailor promising HC microstructures, in which *d*_*002*_ = 0.384 nm, *I*_*D*_*/I*_*G*_ = 1.33, and SSA = 2.28 m^2^ g^−1^ that belong to the targeted microstructure with a zone of moderate *d*_*002*_ (0.376–0.394 nm), moderate *I*_*D*_*/I*_*G*_ (1.10–2.30), and low SSA (0.26–17.84 m^2^ g^−1^). Compared to traditional high-temperature (1300 °C) derived HC, the HC-10-900 synthesized at 900 °C under 10 MPa simultaneously exhibits higher Na^+^ storage sites and faster Na^+^ diffusion kinetics, together with a large energy consumption reduction (from 214.2 to 107.8 kWh) and a large carbon emission reduction of (from 98.87 to 51.94 g). The Ah level HC-10-900||NFM pouch cell delivers a high specific energy of 208.1 Wh kg^−1^ at 1.5–4.3 V based on the entire device mass, competitive with the previously reported Ah-level SIBs. Moreover, it maintains high capacity retention of 96.1% over 1000 cycles. These results demonstrate an energy-saving pathway to design, fabricate, and screen next-generation HC negative materials for SIBs.

## Methods

### Materials

Glucose (C_6_H_12_O_6_, purity ≥99%, source: Macklin), acetylene black (purity ≥99.5%, average particle size was 20–40 nm, source: Canrd), carboxymethyl cellulose (CMC, purity ≥94%, source: KeJing), Glass fiber membrane (Whatman GF/C, pore size: 1.2 μm, thickness: 260 μm, diameter: 17 mm, porosity ≥86%, and source: Canrd), polypropylene (Celgard 2400, thickness: 25 μm, porosity: 41%, pore size: 50 nm, source: Kejing), NFM (particle size of 4.0–6.0 μm, tapped density of 1.38 g cm^−3^, source: Canrd), NVP (particle size of 4.0–8.0 μm, tapped density of 0.74 g cm^−3^, source: Canrd), polyvinylidene fluoride (PVDF, Mw ≈ 500,000 g mol^−1^ source: Canrd), N-methyl-2-pyrollidone solvent (NMP, purity ≥99%, source: Aladdin) were used as received. The NaPF_6_ (purity ≥99.8%), ethylene carbonate (EC, purity ≥99.8%), diethyl carbonate (DEC, purity ≥99.8%), diglyme (purity ≥99.8%), and Dimethyl Carbonate (DMC, purity ≥99.5%, anhydrous) were purchased from Suzhou Duoduo Chemical Co., Ltd. Aluminium foil (15 μm, 99.6%) and copper foil (purity ≥99.9%, 10 μm thickness, battery grade) were obtained from Shenzhen Kejing Star Technology Co., Ltd. Sodium lumps (99.9% purity, thickness: 0.45 mm) were purchased from Aladdin Biochemical Technology Co., Ltd.

### Materials synthesis

Glucose was pre-carbonized at 300 °C for 1 h in a muffle furnace with an argon atmosphere and subsequently cooled to 25 °C. The obtained black substance was named HC-P. The HC-P was filled into a graphite mold and subsequently placed in a muffle furnace with an argon atmosphere. Multi-layer high-temperature alloy blocks are placed above the graphite mold to apply 10 MPa pressure to the material in the mold. The material was heated to 900 °C and maintained for 2 h, the obtained bulk carbon was named HC-10-900. Keeping the carbonization temperature constant and changing the pressure to 5 MPa, the obtained bulk carbon was named HC-5-900. The HC-P was filled into a graphite mold and subsequently placed in a hot press sintering furnace with an argon atmosphere. The material was heated to 900 °C and maintained for 2 h by a graphite heat generator, meanwhile, the graphite upper press head and lower press head were driven by a pressurizing device to apply 50 MPa pressure to the material in the mold. The obtained bulk carbon was named HC-50-900. Keeping the carbonization temperature constant and changing the pressure to 100 MPa, the obtained bulk carbon was named HC-100-900. As a comparative experiment, HC-P was carbonized in a muffle furnace with an argon atmosphere at 900 °C for 2 h at atmospheric pressure, and the HC obtained was named HC-0-900. To demonstrate the advantages of low-temperature thermal-force coupling, HC-P carbonized at 700 °C, 1000 °C, 1100 °C, 1200 °C, 1300 °C, and 1400 °C for 2 h was respectively named HC-0-700, HC-0-1000, HC-0-1100, HC-0-1200, HC-0-1300, and HC-0-1400.

Before carbonization experiments, the heating equipment (tube furnaces (Anhui BEQ Equipment Technology Co., Ltd, BTF-1600C), muffle furnaces (Bona Therm Furnace Co., Ltd, BR-14AS-8), and hot-press carbonization furnaces (Shanghai Chenhua Science Technology Co., Ltd, ZT-50-22Y) is evacuated using a vacuum pump to create a negative-pressure environment inside the chamber, thereby eliminating interference from air. Subsequently, the vacuum pump is shut off, and argon gas is introduced to fill the furnace chamber until exhaust gas is regularly expelled. Argon gas is continuously supplied throughout the entire carbonization process.

### Material characterizations

The morphology and microstructure of HC were characterized by scanning electron microscopy (SEM, Hitachi S4800) and HRTEM (JEOL JEM-F200). The crystallinity of HC was investigated by XRD (Bruker D8 Advance diffractometer, Cu Kα, *λ* = 1.54056 Å). The graphitization degree of HC was studied by Raman spectroscopy (collected using a 532 nm laser, Horiba Confocal Raman Microscopy). The SSA of HC was calculated through nitrogen adsorption-desorption isotherms. The closed pore volume was analyzed by true density testing (AccuPycII 1340 analyzer) using helium for degassing. The closed pore structures within the HC were obtained through the SAXS (Xenocs Xeuss 2.0). The elemental content was studied using the XPS (Thermo Scientific K-Alpha). The NEXAFS was collected at XAFCA@SSLS. The mass percentage of the C element of HC was determined by using a carbon-sulfur analyzer (CS844, LECO).

In-situ Raman and XRD were tested by a customized electrolytic cell device. Among them, the observation window of the in-situ Raman system was quartz, and the observation window of the XRD system was beryllium. In the ex-situ XPS and SAXS test, the coil cells that had reached the desired potential were disassembled in the glove box, and the electrodes were washed with DMC, and the samples were loaded into glass. To avoid the influence of air on the sample in the ex-situ XPS test, the electrodes were all carried in an inert atmosphere and transferred into the chamber using a vacuum transfer box. An ionic gun with an energy of 1 kV was selected, and the estimated sputter rate is about 5 nm s^−1^, the etching depth is 10 nm. The in-situ and ex-situ tests of the cells were initial discharged and charged to the desired potential points at a specific current of 25 mA g^−1^ at 28 °C.

### Coin cell preparation

The negative material was obtained by mixing active material (80%), acetylene black (10%), and CMC (10%) in a deionized water solvent. The stirred slurry was single-sided coated on the copper foil by an automatic coating machine and vacuum dried at 80 °C for 12 h. The negative electrodes were cut into 12 mm discs with an average mass loading of ≈ 1.6 mg cm^−2^ by a manual punching machine (MSK-T10). The half-cells were assembled using coin cells (CR 2032, Canrd) in an Ar-filled glovebox (H_2_O and O_2_ levels both <0.01ppm). The electrolyte was 1.0 mol L^−1^ NaPF_6_ dissolved in diglyme (amount 120 μL). A Glass fiber membrane was used as the separator. The positive material was obtained by mixing active material (NFM and NVP) (80%), acetylene black (10%), and PVDF (10%) in NMP solvent. The stirred slurry was single-sided coated on the aluminum foil by an automatic coating machine and vacuum dried at 80 °C for 12 h. The positive electrodes were cut into 12 mm discs with a mass loading of 1.0–2.5 mg cm^−2^ by a manual punching machine (MSK-T10). The HC-10-900||NVP (N/P of 1.24), HC-0-900||NFM (N/P of 1.06), HC-0-1300||NFM (N/P of 1.11), and HC-10-900||NFM (N/P of 1.08) full cells were assembled in coin cells (CR 2032) by NVP and NFM as positive materials, respectively. In HC-10-900||NVP full cell, 1.0 mol L^−1^ NaPF_6_ dissolved in diglyme was used as an electrolyte. In HC-10-900||NFM full cell, 1.0 mol L^−1^ NaPF_6_ dissolved in EC and DEC (1:1 volume ratio, amount 120 μL) was used as electrolyte.

### Pouch cell preparation

The pouch cell negative electrodes were fabricated using an active material (HC-10-900), conductive agent (acetylene black) and binder (CMC) in weight ratio of 90:5:5. The pouch cell positives were fabricated using an active material (NFM), conductive agent (acetylene black) and binder (PVDF) in weight ratio of 96:2:2. The slurry was uniformly coated on to both sides of Al foil, followed by drying for 12 h at 80 °C. For these pouch cells, the double-sided positive and negative electrodes were stacked layer by layer with a polypropylene separator (N/P of 1.07), and 1.0 mol L^−1^ NaPF_6_ dissolved in EC and DEC (1:1 volume ratio) was used as electrolyte. Weigh out 3 g of electrolyte and use a rubber-tipped pipette to dispense it into the assembled dry cell. The pouch cell rest (12 h), formation (0.1 A change to 2.5 V), and aging (45 °C, 12 h) are performed sequentially after vacuum sealing (180 °C, 5 s). Use scissors to cut open the gas chamber to release the gas, and then perform a secondary vacuum seal (180 °C, 5 s). The specific pouch battery composition is provided in Supplementary Table S8. All pouch cell fabrication was performed in a dry room with a dew point of −60 °C.

### Electrochemical characterizations

The charge-discharge cycle were measured at various specific currents with potential windows of 0.001–2.5 V (*vs*. Na^+^/Na) for half cells with HC, 2–3.8 V for NVP half cell and HC-10-900||NVP full cell, 2–4.0 V for NFM half cell, HC-10-900|| NFM full cell, HC-0-900||NFM full cell, and HC-0-1300||NFM full cell were tested using a NEWARE battery testing system (WHW-200L-160CH-B). The charge-discharge cycle was measured at various specific currents with potential windows of 1.5-3.95 V and 1.5-4.3 V for HC-10-900||NFM pouch cell on the NEWARE battery testing system under a fixing device with 1.0-MPa external pressure. The CV curves and EIS (potentiostatic mode, frequency range 1.0 MHz to 0.01 Hz, amplitude 5 mV, with 10 data points per frequency decade) were obtained at a CHI760D electrochemical workstation (Shanghai Chenhua). A total of 50 points were collected within the test frequency range. A quasistationary potential was applied for 600 s before carrying out the EIS measurement. The GITT was employed by the Land system, incorporating a pulse-specific current of 25 mA g^−1^ for 30 min, followed by a relaxation period of 2 h. All electrochemical performance tests mentioned in this paper were conducted in a constant-temperature test chamber at 28 ± 0.3 °C.

### Molecular dynamics simulation

Classic MD simulations are carried out to explore the carbonization behaviors of glucose at the molecular level. First, the MD simulation box with dimensions of around 40 × 40 × 40 Å^3^ is created, then 337 glucose molecules are randomly placed into the as-created MD box through Packmol^60^. To eliminate the potential surface effects, periodic boundary conditions (PBCs) are imposed in the three orthogonal directions. The ReaxFF is utilized to describe the atomic interactions in the investigated systems^61^. The ReaxFF force field is demonstrated to accurately capture the breaking and formation of covalent bonds of various carbon-based structures subjected to a variety of conditions, such as heating and straining. The version of ReaxFF parametrized is utilized to explore the carbonized behaviors of glucose molecules under high temperature conditions^62^.

Before MD simulations, an energy minimization is first performed to optimize the configuration of the glucose-based systems. Then, MD simulation with 200,000 timesteps is performed at 300 K within the NPT (constant number of particles, constant pressure, and constant temperature) ensemble to obtain an equilibrated glucose-based system. Subsequently, MD simulation with 300,000 timesteps is performed at 473.15 K within the NPT ensemble to increase the unit cell size and provide space for the pre-carbonization reaction. The lattice basis vectors are balanced thereafter. Following this, MD simulations with 1,500,000 timesteps are carried out at 3000 K under the NVT ensemble to carbonize glucose. During the MD simulations, the temperature is controlled using the Nose-Hoover thermostat. Afterwards, the byproducts of small molecules such as CO_2_, CO, H_2_O, and so on, are removed and annealed at 300 K within the NPT ensemble to retain the Carbonized glucose. Finally, carbonized glucose was graphitized under varying pressures to investigate the effect of pressure on its structure. During the carbonization simulation, the temperature was set at 1173.15 K, and the pressures were atmospheric, 10 MPa, and 100 MPa, respectively, followed by 2,000,000 timesteps in the NPT system. To balance computational efficiency and accuracy, one timestep is set to 0.15. The dynamics of atoms in the system follow the classical Newtonian motion. All the MD simulations are implemented using the Large-scale Atomic/Molecular Massively Parallel Simulator^63^.

The disordered microstructure of HC exhibits strong dependence on synthesis parameters, particularly annealing temperature, which significantly hinders the development of a structure model. Although MD simulations allow for the specification of specific thermodynamic temperatures, it is difficult to establish a direct mapping between the simulated system and the actual experimental environment. The time scale of all-atom MD simulations is typically limited to the nanosecond range, whereas experimental annealing processes often last for several hours. This vast gap of several orders of magnitude between the two time scales makes comparisons between simulations and experiments highly complex^64^.

### Laboratory-scale cost and CO_2_ emission assessment

Our analysis uses a bottom-up cost accounting framework with a cradle-to-gate system boundary. We do not employ a discounted cash flow model or standard life cycle assessment impact methods (e.g., ReCiPe). Environmental impact is limited to the climate change category, monetized through the SCC.

To quantify the cost and environmental impact of the three processes, we calculate the private production cost and the associated CO_2_ emissions of 100 g glucose-derived HC negative material, and translate emissions into social costs using scenario-specific SCC. The calculation for each component is detailed below. All parameters are scenario-specific and taken from Supplementary Table S10.

### Production cost

The production cost of HC is decomposed into four major components: feedstock, equipment depreciation, labor and overhead, and electricity consumption. The total private production cost per process is calculated as the sum of these components:

1 $${C}_{{private}}={C}_{{feed}}+{C}_{{eqpt}}+{C}_{{labor}}+{C}_{{elec}}$$

Feedstock cost $${C}_{{feed}}$$($/100 g) is determined by multiplying the feedstock mass input $${m}_{f}$$(100 g) by the unit feedstock price $${p}_{{feed}}$$($/kg):

2 $${C}_{{feed}}={p}_{{feed}}*{m}_{f}$$

Equipment depreciation cost ($${C}_{{eqpt}}$$($/100 g)) arises from the pre-carbonization and pyrolysis stages, which is a function of the process-specific processing time (h) and the hourly equipment depreciation rate $${c}_{{eqpt}}$$($/hr):

3 $${C}_{{eqpt}}={c}_{{eqpt}}*\left({h}_{{pre}}+{h}_{{pyro}}*B\right),$$

where $B={m}_{{pre}}/8$ is the number of 8 g pyrolysis batches processed per 100 g of feedstock, ${m}_{{pre}}$(g) is the output of pre-treated HC material from 100 g glucose feedstock that can be used in the pyrolysis process. The hourly equipment depreciation rate is obtained by straight-line depreciation of equipment cost over a 15-year useful life with continuous operation (8760 h/yr), converted at 7 RMB/USD. Pre-carbonization uses a standard muffle furnace (10,000 RMB) for all pathways. Pyrolysis equipment costs are 15,000 RMB for LTFC (muffle furnace with graphite mold and alloy press), 30,000 RMB for HTC (high-temperature muffle furnace), and 10,000 RMB for LTC (standard muffle furnace).

Labor and overhead costs ($${C}_{{labor}}$$($/100 g)) are assumed to scale proportionally with total equipment depreciation cost, using a fixed ratio (λ):

4 $${C}_{{labor}}=\lambda*{C}_{{eqpt}}.$$

Electricity costs ($${C}_{{elec}}$$($/100 g)) include power consumption during both pre-carbonization and pyrolysis. The total electricity use ($${E}_{{total}}$$(kWh)) is multiplied by the electricity price ($${p}_{{elec}}$$($/kWh)):

5 $${E}_{{total}}={E}_{{pre}}+B*{E}_{{pyro}},$$

6 $${C}_{{elec}}={E}_{{total}}*{p}_{{elec}}.$$

### Cost per kWh

Cost per kWh is calculated from the total production cost divided by the total energy delivered by a full battery cell over its lifetime.

Let *C*₁ and *C*_*N*_ denote the discharge capacities (Wh) at cycles 1 and *N*, respectively. We assume an exponential capacity decay that can be calculated as

7 $${C}_{N}={C}_{1}*{r}^{N},$$

Where r is the per-cycle retention rate.

The total energy E(N) in kWh/kgHC delivered through N cycles is the geometric sum.

8 $$E\left(N\right)={C}_{1}*\frac{1-{r}^{N}}{1-r}$$

We evaluate this at the point where the cell capacity declines to 80% of its initial value (${N}_{80\%}$):

9 $${N}_{80\%}=1+\frac{{\mathrm{ln}}\left(0.8\right)}{{\mathrm{ln}}r}$$

The cost per kWh is then

10 $${c}_{{kWh}}=\frac{{Cos}{t}_{{kgHC}}}{E\left({N}_{80\%}\right)}$$

where ${Cos}{t}_{{kgHC}}$ is the unit private cost per kilogram of HC output

11 $${Cos}{t}_{{kgHC}}=\frac{{C}_{{private}}}{{m}_{{HC}}}*1000,$$

and ${m}_{{HC}}$ is the total output of HC per kg of feedstock calculated as ${m}_{{HC}}=B*{y}_{8g}$, and ${y}_{8g}$(g) is the mass of HC produced per batch.

Therefore, this levelized cost also captures the performance of the HC material produced.

### Emissions cost

We consider the societal cost of both electricity-related and process-related CO_2_ emissions monetized using the SCC.

Electricity-related emissions are computed as total energy use multiplied by the grid emission intensity (γ in kg CO_2_/kWh):

12 $${M}_{{elec}}={E}_{{total}}*\frac{\gamma }{{m}_{{HC}}}*1000$$

Process emissions arise from pre-carbonization and pyrolysis reactions, expressed as

13 $${M}_{{pre}}=\frac{\left(\frac{{m}_{{feed}}}{100}*{\eta }_{{pre}}\right)}{1000}$$

14 $${M}_{{pyro}}=B*\frac{{\eta }_{{pyro}}}{1000}$$

Total process-related emissions are

15 $${M}_{{process}}=({M}_{{pre}}+{M}_{{pyro}})*\frac{1000}{{m}_{{HC}}}$$

The social cost of emissions can be expressed by multiplying the emissions by the SCC:

16 $${C}_{{CO}2}=\left({M}_{{elec}}+{M}_{{process}}\right)*{SCC}$$

This emission cost can be added to the private production cost to get the full social cost of production:

17 $$S{C}_{{kgHc}}={Cos}{t}_{{kgHC}}+{C}_{{CO}2}$$

## Supplementary information

Supplementary Information

Description of Additional Supplementary Files

Supplementary Data 1

Transparent Peer Review file

## Source data

Source Data

## Data Availability

All data are available within the paper, Supplementary Information, and Source Data file. Among these, the ML database containing the reported 565 samples and 7600 data values is stored in a Supplementary Data file, the source data is in an Excel spreadsheet, and accession code and direct link are stored in https://github.com/erweixi/Targeted-Screened-Hard-Carbon. The Figs. 1(a,e), 2(a,k,l), 4(c,d,g,h,k,l), 5a, Supplementary Figs. 13(c,f), 19 and 61a. were both created in PowerPoint. Source data are provided with this paper.

## Code availability

Code is available on zenodo repository^65^: https://zenodo.org/records/21620218.

## References

[1] Zhao, C. et al. Rational design of layered oxide materials for sodium-ion batteries. *Science* **370**, 708–711 (2020). 10.1126/science.aay9972 33154140

[2] Yang, Y. et al. Decoupling the air sensitivity of Na-layered oxides. *Science* **385**, 744–752 (2024). 10.1126/science.adm9223 39146426

[3] Vaalma, C., Buchholz, D., Weil, M. & Passerini, S. A cost and resource analysis of sodium-ion batteries. *Nat. Rev. Mater.* **3**, 18013 (2018).

[4] Li, Y. et al. Origin of fast charging in hard carbon anodes. *Nat. Energy* **9**, 134–142 (2024).

[5] Wang, B. et al. Electron paramagnetic resonance as a tool to determine the sodium charge storage mechanism of hard carbon. *Nat. Commun.* **15**, 3013 (2024). 10.1038/s41467-024-45460-3 PMC11001870 38589362

[6] Zhang, Y. et al. Redefining closed pores in carbons by solvation structures for enhanced sodium storage. *Nat. Commun.* **16**, 3634 (2025). 10.1038/s41467-025-59022-8 PMC12003850 40240373

[7] Ruan, J. et al. Current collector interphase design for high-energy and stable anode-less sodium batteries. *Nat. Sustain.* **8**, 530–541 (2025).

[8] Tang, Z. et al. Revealing the closed pore formation of waste wood-derived hard carbon for advanced sodium-ion battery. *Nat. Commun.* **14**, 6024 (2023). 10.1038/s41467-023-39637-5 PMC10533848 37758706

[9] Hyun, J. C. et al. Design guidelines for a high-performance hard carbon anode in sodium ion batteries. *Energy Environ. Sci.* **17**, 2856–2863 (2024).

[10] Cheng, Z. et al. Interlayer-expanded carbon anodes with exceptional rates and long-term cycling via kinetically decoupled carbonization. *Joule* **9**, 101812 (2025).

[11] Chen, X. et al. Filling carbon: A microstructure-engineered hard carbon for efficient alkali metal ion storage. *Energy Environ. Sci.* **16**, 4041–4053 (2023).

[12] Sun, Y. et al. Solvent co-intercalation in layered cathode active materials for sodium-ion batteries. *Nat. Mater.* **24**, 1441–1449 (2025). 10.1038/s41563-025-02287-7 PMC12404988 40681866

[13] Liu, J. et al. Precisely tunable instantaneous carbon rearrangement enables low-working-potential hard carbon toward sodium-ion batteries with enhanced energy density. *Adv. Mater.* **36**, 2407369 (2024). 10.1002/adma.202407369 39221669

[14] Zhang, Y. et al. Ultrafast synthesis of hard carbon anodes for sodium-ion batteries. *Proc. Natl. Acad. Sci. USA* **118**, e2111119118 (2021). 10.1073/pnas.2111119118 PMC8545444 34663702

[15] Sun, Z. et al. Advanced hard carbon materials for practical applications of sodium-ion batteries developed by combined experimental, computational, and data analysis approaches. *Prog. Mater. Sci.* **149**, 101401 (2025).

[16] Liu, H. et al. Highways construction in amorphous carbon anode enables 10C fast-charging sodium-ion batteries. *Angew. Chem. Int. Ed.* **65**, e25941 (2026). 10.1002/anie.202525941 41630195

[17] Qiu, C. et al. One-step construction of closed pores enabling high plateau capacity hard carbon anodes for sodium-ion batteries: Closed-pore formation and energy storage mechanisms. *ACS Nano* **18**, 11941–11954 (2024). 10.1021/acsnano.4c02046 38652811

[18] Wang, F. et al. Revitalizing sodium-ion batteries via controllable microstructures and advanced electrolytes for hard carbon. *eScience* **4**, 100181 (2024).

[19] Xu, Y.-J. et al. Cellulose-grafting boosted pyrolysis nucleation: Achieving low-temperature construction of hard carbon anodes with long low-voltage plateau and ultrafast Na storage kinetics. *Energy Storage Mater.* **75**, 104031 (2025).

[20] Cui, J. et al. Data-driven intelligent carbonization unifies diverse biomass into high-performance hard carbon negative electrodes. *Nat Commun*. **17**, 3885 (2026). 10.1038/s41467-026-70411-5 PMC13125499 41826331

[21] Qiu, Y. et al. Rapid closed pore regulation of biomass-derived hard carbons based on flash joule heating for enhanced sodium ion storage. *Adv. Funct. Mater.* **35**, 2423559 (2025).

[22] Wang, H. et al. Thermal shock reducing amorphous carbon ratio in hard carbon for improved rate capability of sodium ion storage. *Carbon* **229**, 119528 (2024).

[23] Song, Z. et al. Joule heating for structure reconstruction of hard carbon with superior sodium ion storage performance. *Chem. Eng. J.* **496**, 154103 (2024).

[24] Luo, F. et al. Rapid flash heating of hard carbon: Enhanced sodium storage via hierarchical graphitization. *Energy Storage Mater.* **86**, 104929 (2026).

[25] Wang, A. et al. Lignin derived hard carbon for sodium ion batteries: Recent advances and future perspectives. *Prog. Mater. Sci.* **152**, 101452 (2025).

[26] Wen, Q. et al. Carbon engineering for sodium batteries: multi-role architectures bridging material design and hybrid system innovation. *Chem. Soc. Rev.* **54**, 9317–9369 (2025). 10.1039/d5cs00515a 40963370

[27] Owusu, S. Y., Doulah, A. B. M. S. U., Edlabadkar, V. A., George, K. J. & Leventis, C. S. The effect of nanomorphology as quantified via the K-index on the drug delivery properties of isocyanate-derived aerogels. *ACS Appl. Polym. Mater.* **6**, 10655–10668 (2024).

[28] Owusu, S. Y., Boateng, M. A. & Soni, R. U. Machine learning predictions of drug release from isocyanate-derived aerogels. *J. Mater. Chem. B* **13**, 6233–6245 (2025). 10.1039/d5tb00289c 40341491

[29] Han, Z. et al. Machine-learning-assisted design of a binary descriptor to decipher electronic and structural effects on sulfur reduction kinetics. *Nat. Catal.* **6**, 1073–1086 (2023).

[30] Rao, L., Yuan, Y., Shen, X., Yu, G. & Chen, X. Designing nanotheranostics with machine learning. *Nat. Nanotechnol.* **19**, 1769–1781 (2024). 10.1038/s41565-024-01753-8 39362960

[31] Owusu, S. Y. Machine learning prediction of the reversible capacities of a biomass-derived hard carbon anode for sodium-ion batteries. *RSC Sustain.* **3**, 3133–3143 (2025).

[32] Lin, T. et al. Investigating explainable transfer learning for battery lifetime prediction under state transitions. *eScience* **4**, 100280 (2024).

[33] Bai, P. et al. Solid electrolyte interphase manipulation towards highly stable hard carbon anodes for sodium ion batteries. *Energy Storage Mater.* **25**, 324–333 (2020).

[34] Li, J. et al. Machine learning-assisted low-dimensional electrocatalysts design for hydrogen evolution reaction. *Nano-Micro Lett.* **15**, 227 (2023). 10.1007/s40820-023-01192-5 PMC10575847 37831203

[35] Moon, J. et al. Active learning guides discovery of a champion four-metal perovskite oxide for oxygen evolution electrocatalysis. *Nat. Mater.* **23**, 108–115 (2024). 10.1038/s41563-023-01707-w 37919351

[36] Chen, Y. et al. Defect-selectivity and “order-in-disorder” engineering in carbon for durable and fast potassium storage. *Adv. Mater.* **34**, e2108621 (2022). 10.1002/adma.202108621 34850465

[37] Sun, Z. et al. Edge-oxidation-induced densification towards hybrid bulk carbon for low-voltage, reversible and fast potassium storage. *Energy Storage Mater.* **53**, 482–491 (2022).

[38] Bobyleva, Z. V. et al. Caramelization as a key stage for the preparation of monolithic hard carbon with advanced performance in sodium-ion batteries. *ACS Appl. Energy Mater.* **6**, 181–190 (2022).

[39] Zhang, X. et al. A powder-metallurgy-based strategy toward three-dimensional graphene-like network for reinforcing copper matrix composites. *Nat. Commun.* **11**, 2775 (2020). 10.1038/s41467-020-16490-4 PMC7265306 32488100

[40] Yin, X. et al. Enabling fast Na^+^ transfer kinetics in the whole-voltage-region of hard-carbon anodes for ultrahigh-rate sodium storage. *Adv. Mater.* **34**, e2109282 (2022). 10.1002/adma.202109282 35075693

[41] Qian, Y. et al. Pre-construction gas nanochamber strategy: Producing hard carbon rich in closed pores and sp^2^-C for sodium-ion batteries. *Energy Storage Mater.* **75**, 104092 (2025).

[42] Zeng, Y. et al. Identifying the importance of functionalization evolution during pre-oxidation treatment in producing economical asphalt-derived hard carbon for Na-ion batteries. *Energy Storage Mater.* **73**, 103808 (2024).

[43] Owusu, S. Y., Soni, R. U. & Leventis, C. S. Catalytic synthesis of iron-doped graphitic aerogels from poly(phloroglucinol-terephthalaldehyde-urethane) precursors. *Carbon Trends* **21**, 100573 (2025).

[44] Sundaramoorthy, S. et al. High-capacity anode for sodium-ion batteries using hard carbons derived from polyurea-cross-linked silica xerogel powders. *ACS Appl. Energy Mater.* **7**, 9289–9299 (2024).

[45] Yang, R. et al. Growth space engineering of closed pores in hard carbon for low-temperature sodium-ion batteries. *Energy Storage Mater.* **80**, 104415 (2025).

[46] Sun, Z. et al. Oxygen-driven bulk defect engineering in carbon to reduce voltage hysteresis for fast potassium storage at low voltage. *Appl. Catal. B Environ. Energy* **343**, 123473 (2024).

[47] Bai, X. et al. Heat-resistant super-dispersed oxide strengthened aluminum alloys. *Nat. Mater.* **23**, 747–754 (2024). 10.1038/s41563-024-01884-2 38671162

[48] Qin, X. et al. Self-pressure silicon–carbon anodes for low-external-pressure solid-state Li-ion batteries. *ACS Nano* **19**, 17760–17773 (2025). 10.1021/acsnano.5c03017 40304407

[49] He, J. et al. Sieving pore design enables stable and fast alloying chemistry of silicon negative electrodes in Li-ion batteries. *Nat. Commun.* **16**, 4858 (2025). 10.1038/s41467-025-60191-9 PMC12104450 40414886

[50] Xue, Y. et al. Substitution index-prediction rules for low-potential plateau of hard carbon anodes in sodium-ion batteries. *Adv. Mater.* **37**, 2417886 (2025). 10.1002/adma.202417886 40275813

[51] Hu, Q. et al. Understanding the sodium storage behavior of closed pores/carbonyl groups in hard carbon. *ACS Nano* **18**, 21491–21503 (2024). 10.1021/acsnano.4c06281 39082107

[52] Duan, J. et al. Substituent-induced microstructural modulation of epoxy resin-derived hard carbon for high-performance sodium-ion battery anodes. *Chem. Eng. J.* **508**, 161184 (2025).

[53] Zheng, P. et al. Multi boron-doping effects in hard carbon toward enhanced sodium ion storage. *J. Energy Chem.* **100**, 730–738 (2025).

[54] Chen, X. et al. An overall understanding of sodium storage behaviors in hard carbons by an “Adsorption-Intercalation/Filling” hybrid mechanism. *Adv. Energy Mater.* **12**, 2200886 (2022).

[55] Wu, C. et al. Industrial-scale hard carbon designed to regulate electrochemical polarization for fast sodium storage. *Angew. Chem. Int. Ed.* **63**, e202406889 (2024). 10.1002/anie.202406889 38742478

[56] Morikawa, Y., Nishimura, S.-I., Hashimoto, R.-I., Ohnuma, M. & Yamada, A. Mechanism of sodium storage in hard carbon: an X-ray scattering analysis. *Adv. Energy Mater.* **10**, 1903176 (2019).

[57] Wang, Z. et al. Probing the energy storage mechanism of quasi-metallic Na in hard carbon for sodium-ion batteries. *Adv. Energy Mater.* **11**, 2003854 (2021).

[58] Wang, J. et al. Direct recycling of spent cathode material at ambient conditions via spontaneous lithiation. *Nat. Sustain.* **7**, 1283–1293 (2024).

[59] Wang, J. et al. Sustainable upcycling of spent LiCoO_2_ to an ultra-stable battery cathode at high voltage. *Nat. Sustain.* **6**, 797–805 (2023).

[60] Martínez, L., Andrade, R., Birgin, E. G. & Martínez, J. M. Packmol: a package for building initial configurations for molecular dynamics simulations. *J. Comput. Chem.* **30**, 2157–2164 (2009). 10.1002/jcc.21224 19229944

[61] Duin, A. C. T., Dasgupta, S., Lorant, F. & Goddard, W. A. ReaxFF: A reactive force field for hydrocarbons. *J. Phys. Chem. A* **105**, 9396–9409 (2001).

[62] Kowalik, M. et al. Atomistic scale analysis of the carbonization process for C/H/O/N-based polymers with the ReaxFF reactive force field. *J. Phys. Chem. B* **123**, 5357–5367 (2019). 10.1021/acs.jpcb.9b04298 31145615

[63] Plimpton, S. Fast parallel algorithms for short-range molecular dynamics. *J. Comput. Phys.* **117**, 1–19 (1995).

[64] Li, J., Peng, C., Wang, J., Li, J. & Zhang, H. Towards an atomistic understanding of hard carbon electrode materials and sodium behaviors. *Diam. Relat. Mater.* **129**, 109355 (2022).

[65] Sun, Z. et al. 900°C thermal-force coupling carbonization tailoring hard carbon microstructures enables high performance sodium-ion pouch batteries. Zenodo https://zenodo.org/records/21620218. 10.1038/s41467-026-77440-0 PMC13650117 42850225

