# Supplementary material for: 900°C thermal-force coupling carbonization tailoring hard carbon microstructures enables high performance sodium-ion pouch batteries
Source: Nat Commun. 2026 Sep 8;17:10620. doi: 10.1038/s41467-026-77440-0 (PMC13650117; doi:10.1038/s41467-026-77440-0)
Supplement: Supplementary file 1 [file 41467_2026_77440_MOESM1_ESM.pdf]

Supplementary Information

900°C Thermal-Force Coupling Carbonization Tailoring Hard Carbon Microstructures Enables High Performance Sodium-Ion Pouch Batteries

Zongfu Sun[1], Huawei Liu[1], Yihao Cheng[1], Huan Li[2], Zhiyuan Han[3], Ying Tang[1], Xiang Zhang[1,4], Chunsheng Shi[1,4], Fang He[1,4], Yuting Yang[5,6]*, Biao Chen[1,4]*, Chunnian He[1,4,7]*

1 School of Materials Science and Engineering and Tianjin Key Laboratory of Composite and Functional Materials, National Industry-Education Platform of Energy Storage, Tianjin University, Tianjin 300350, P. R. China.
Zongfu Sun, Huawei Liu, Yihao Cheng, Ying Tang, Xiang Zhang, Chunsheng Shi, Fang He, Biao Chen & Chunnian He
2 School of Materials Science and Engineering, Collaborative Innovation Center of Materials Science, Nankai University, Tianjin 300350, China.
Huan Li
3 Tsinghua Shenzhen International Graduate School, Tsinghua University, Shenzhen 518055, P. R. China.
Zhiyuan Han
4 State Key Laboratory of Precious Metal Functional Materials, Tianjin University, Tianjin 300350, P. R. China.
Xiang Zhang, Chunsheng Shi, Fang He, Biao Chen & Chunnian He
5 School of Energy (National Industry-Education Platform for Energy Storage), Tianjin University, 300350, China.
Yuting Yang
6 Department of Economics, University of New Mexico, Albuquerque, NM 87131, USA.
Yuting Yang
7 Joint School of National University of Singapore and Tianjin University, International Campus of Tianjin University, Binhai New City, Fuzhou 350207, China.
Chunnian He

Correspondence to: Yuting Yang, Biao Chen & Chunnian He
ORICD: https://orcid.org/0000-0003-2474-5333 (Yuting Yang), https://orcid.org/0000-0002-6181-2155 (Biao Chen), https://orcid.org/0000-0002-5768-6598 (Chunnian He)
E-mail: ytyang@tju.edu.cn (Yuting Yang), bchen@tju.edu.cn (Biao Chen), cnhe08@tju.edu.cn (Chunnian He)

Table of Contents

1. Supplementary Note 1-18

Note 1: Brief description of Supplementary Fig. 6.

Note 2: Brief description of Supplementary Fig. 7.

Note 3: Brief description of Supplementary Fig. 10.

Note 4: Brief description of Supplementary Fig. 11.

Note 5: Brief description of Supplementary Fig. 13.

Note 6: Brief description of Supplementary Fig. 16.

Note 7: Brief description of Supplementary Fig. 19.

Note 8: Brief description of Supplementary Fig. 20.

Note 9: Brief description of Supplementary Fig. 22.

Note 10: Brief description of Supplementary Fig. 23.

Note 11: Brief description of Supplementary Fig. 25.

Note 12: Brief description of Supplementary Fig. 27.

Note 13: Brief description of Supplementary Fig. 29.

Note 14: Brief description of Supplementary Fig. 30.

Note 15: Brief description of Supplementary Fig. 52.

Note 16: Brief description of Supplementary Fig. 55.

Note 17: Brief description of Supplementary Fig. 58.

Note 18: Brief description of Supplementary Fig. 63.

2. Supplementary Fig. 1-64

Fig. 1: Spearman correlation heatmap between core features, missing variables, and target variable.

Fig. 2: The 5-fold CV R^2 score of different regression models.

Fig. 3: The heatmap of SHAP ranking consistency across different models.

Fig. 4: The performance of using the RF model on the training data.

Fig. 5: The SHAP analysis honeycomb diagram of ICE and reversible plateau capacity.

Fig. 6: The robustness results for the leave-one-feature-out of the locked RF model.

Fig. 7. The change in R^2 after progressively adding missing variables in the RF model.

Fig. 8. The SHAP analysis honeycomb diagram of reversible capacity.

Fig. 9: The verification of microstructure characteristics and performance of HC.

Fig. 10. The microstructure of HC-P.

Fig. 11. The TGA-FTIR of glucose and HC-P.

Fig. 12: The digital images of HC-P.

Fig. 13: Schematic diagram of the preparation of samples.

Fig. 14: The SEM images of samples.

Fig. 15: The HRTEM images of samples.

Fig. 16: Comparison of activation energies.

Fig. 17: Initial model of glucose and model of HC-P after pre-carbonization.

Fig. 18: The pore structure visualization and atomic density of the model.

Fig. 19: The evolution mechanism of HC microstructures.

Fig. 20: The XRD pattern and L_a, L_c of samples.

Fig. 21: The R-factor of samples.

Fig. 22: The $|g^o|$ of samples.

Fig. 23: The Raman spectra of samples.

Fig. 24: The nitrogen adsorption/desorption isotherms and pore size distribution of samples.

Fig. 25: The SAXS pattern and the SAXS patterns presenting the pores of the samples.

Fig. 26: The 2D SAXS images of samples.

Fig. 27: The volume structure of samples.

Fig. 28: The XPS survey spectra of samples.

Fig. 29: High-resolution C 1*s* and O 1*s* XPS spectra of samples.

Fig. 30: The sp^2/sp^3 ratio and C=O ratio of samples.

Fig. 31: The XRD patterns of sucrose, cellulose, and phenolic resin-derived carbon under the LTC and LTFC.

Fig. 32: Initial charging and discharging curves and dQ/dV curves of sucrose, cellulose, and phenolic resin-derived carbon under the LTC and LTFC.

Fig. 33: The CV curves at 0.1 mV s^{-1} of electrodes.

Fig. 34: The capacity-potential normalized curves of electrodes.

Fig. 35: The initial discharge/charge capacities of electrodes.

Fig. 36: The long-cycle performance of electrodes at 25 mA g^{-1}.

Fig. 37: Specific capacity and percentage of slope region and plateau region at discharge and charge.

Fig. 38: Rate performance of electrodes.

Fig. 39: Plateau capacity at different specific currents and comparison of rate capacity.

Fig. 40: Long-cycle performance and charge-discharge curves of HC-10-900.

Fig. 41: Charge-discharge curves of electrodes at 25 mA g^{-1}.

Fig. 42: Comparison of ICE, plateau capacity, and carbonization temperature of electrodes.

Fig. 43: Comparison of electrochemical performance of electrodes.

Fig. 44: Charge-discharge curves and cycle performance at 25 mA g^{-1} of NVP positive electrode.

Fig. 45: Charge-discharge curves of Na||NVP, Na||HC-10-900, and HC-10-900||NVP full cell.

Fig. 46: Cycle performance at 25 mA g^{-1} of HC-10-900||NVP full cell.

Fig. 47: Charge-discharge curves and cycle performance at 25 mA g^{-1} of NFM positive electrode.

Fig. 48: Charge-discharge curves of Na||NFM, Na||HC-0-900, and HC-0-900||NFM full cell.

Fig. 49: Charge-discharge curves of Na||NFM, Na||HC-10-900, and HC-10-900||NFM full cell.

Fig. 50: Charge-discharge curves of Na||NFM, Na||HC-0-1300, and HC-0-1300||NFM full cell.

Fig. 51: Cycle performance of HC-0-900||NFM, HC-10-900||NFM, and HC-0-1300||NFM full cell.

Fig. 52: Charging and discharging curves at 1.5-4.3 V of the sodium-ion pouch cell.

Fig. 53: Rate performance of sodium-ion pouch cell.

Fig. 54: Charging and discharging curves of a sodium-ion pouch cell.

Fig. 55: CV curves at different scanning rates of electrodes.

Fig. 56: Contour plots of CV patterns for electrodes with different scan rates.

Fig. 57: The b values and diffusion-controlled capacity contribution of HC-10-900.

Fig. 58: GITT curves of HC-0-900, HC-5-900, HC-10-900, HC-50-900, and HC-100-900.

Fig. 59: In-situ EIS of HC-0-900, HC-5-900, HC-10-900, HC-50-900, and HC-100-900.

Fig. 60: The DRT transformation of in-situ EIS spectra for HC-10-900.

Fig. 61: The equivalent circuit diagram and the fitting results of R_e, R_s, and R_{ct}.

Fig. 62: In-situ XRD of HC-10-900.

Fig. 63: Ex-situ SAXS and ex-situ XPS of HC-10-900.

Fig. 64: The cost per kWh required in the half battery systems assembled in samples.

3. Supplementary Table 1-13

Table 1. The performance, generalization gaps, and interpretability comparison of multi-models.

Table 2. The R^2 of the training and test sets of the ICE and reversible plateau capacity.

Table 3. Carbon layer structure of HC.

Table 4. Pore structure information of HC.

Table 5. The ICE and reversible plateau capacity of HC prepared at varying carbonization temperatures under atmospheric pressure.

Table 6. The Comparison of electrochemical performance.

Table 7. Comparison of synthesis strategies and carbonization technologies.

Table 8. The specifications of HC-10-900||NFM pouch cell.

Table 9. Comparison of specific energy in sodium-ion pouch cells.

Table 10. Parameter assumptions for the laboratory-scale cost and CO_2 emission assessment.

Table 11. Calculation results of the laboratory-scale cost and CO_2 emission assessment.

Table 12. Cost of preparing HC using 100g feedstock.

Table 13. The cost per kWh required for the full battery systems assembled in HC.

4. Supplementary References

Supplementary Figs.

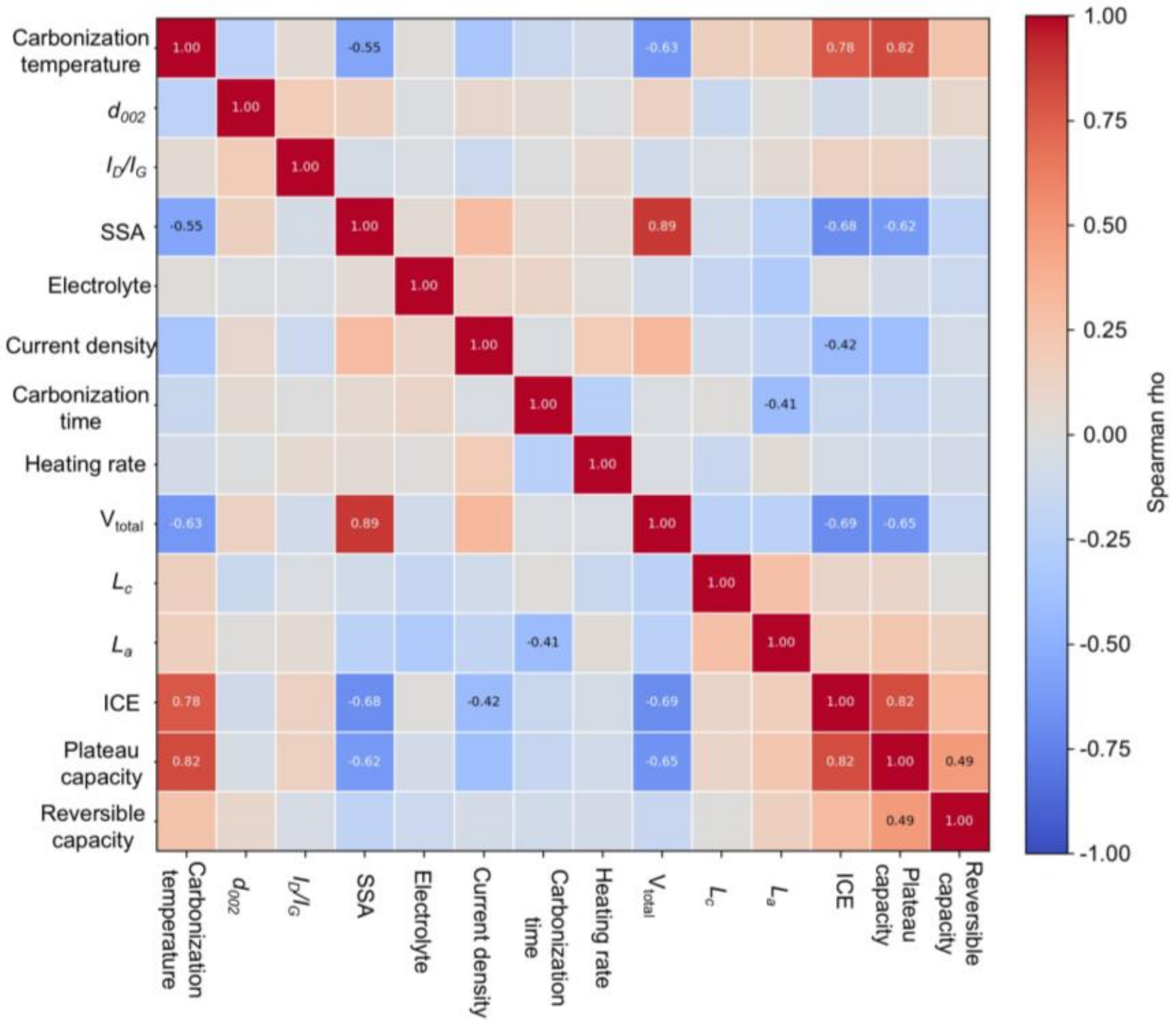

Supplementary Fig. 1. Spearman correlation heatmap between core features, missing variables, and the target variable.

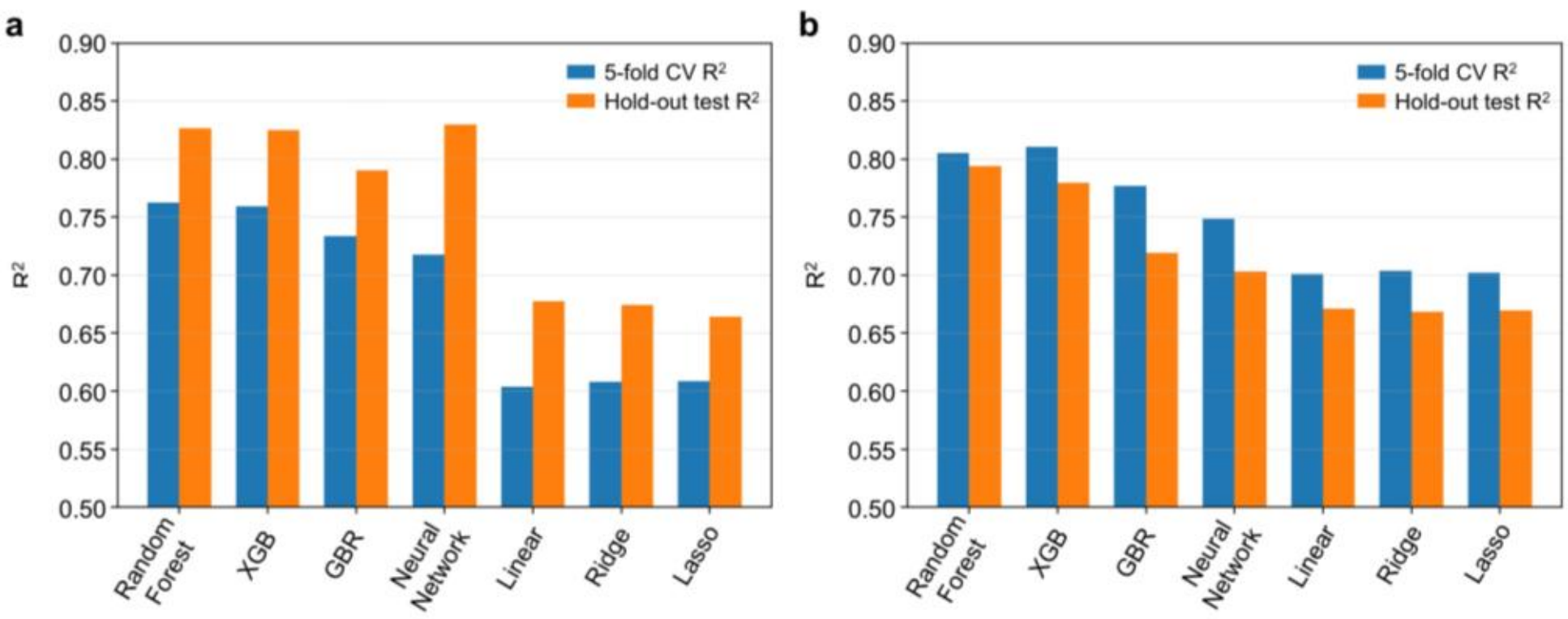

Supplementary Fig. 2. 5-fold cross-validation (CV) R^2 score of different regression models in (a) initial Coulombic efficiency (ICE) and (b) reversible plateau capacity.

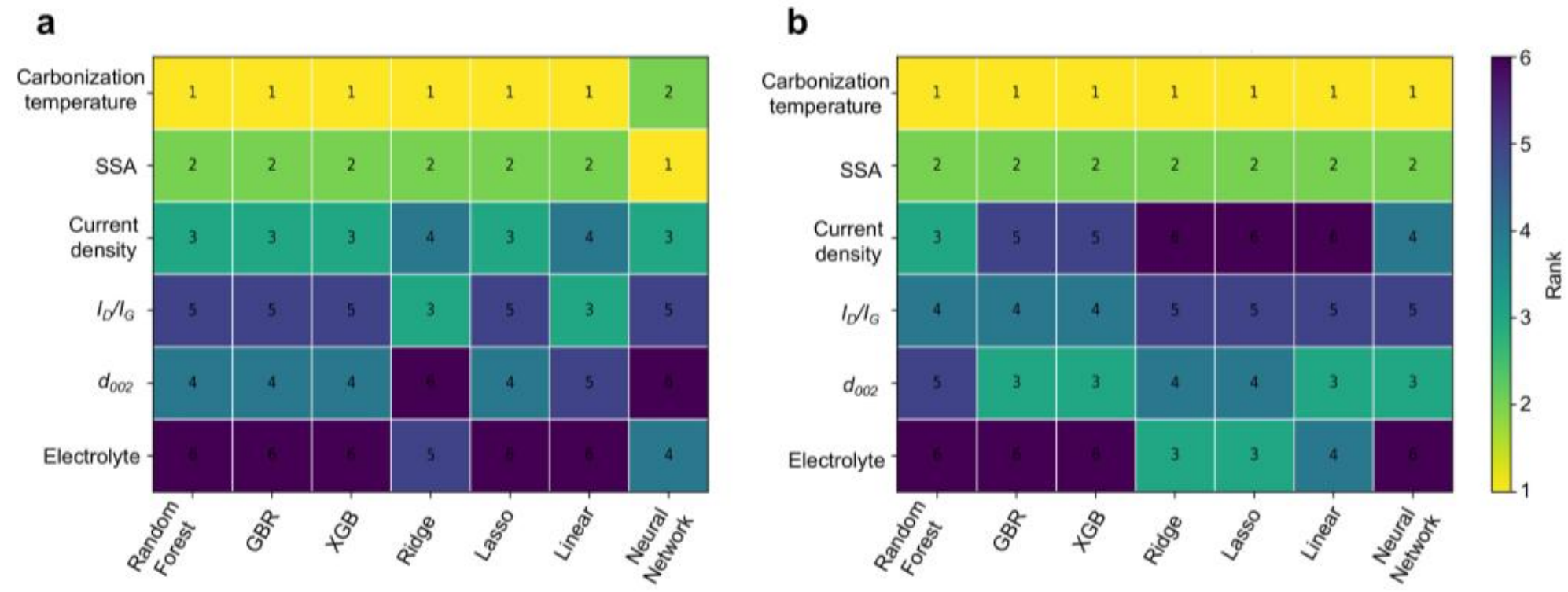

Supplementary Fig. 3. The heatmap of Shapley Additive exPlanations (SHAP) ranking consistency across different models in (a) ICE and (b) reversible plateau capacity.

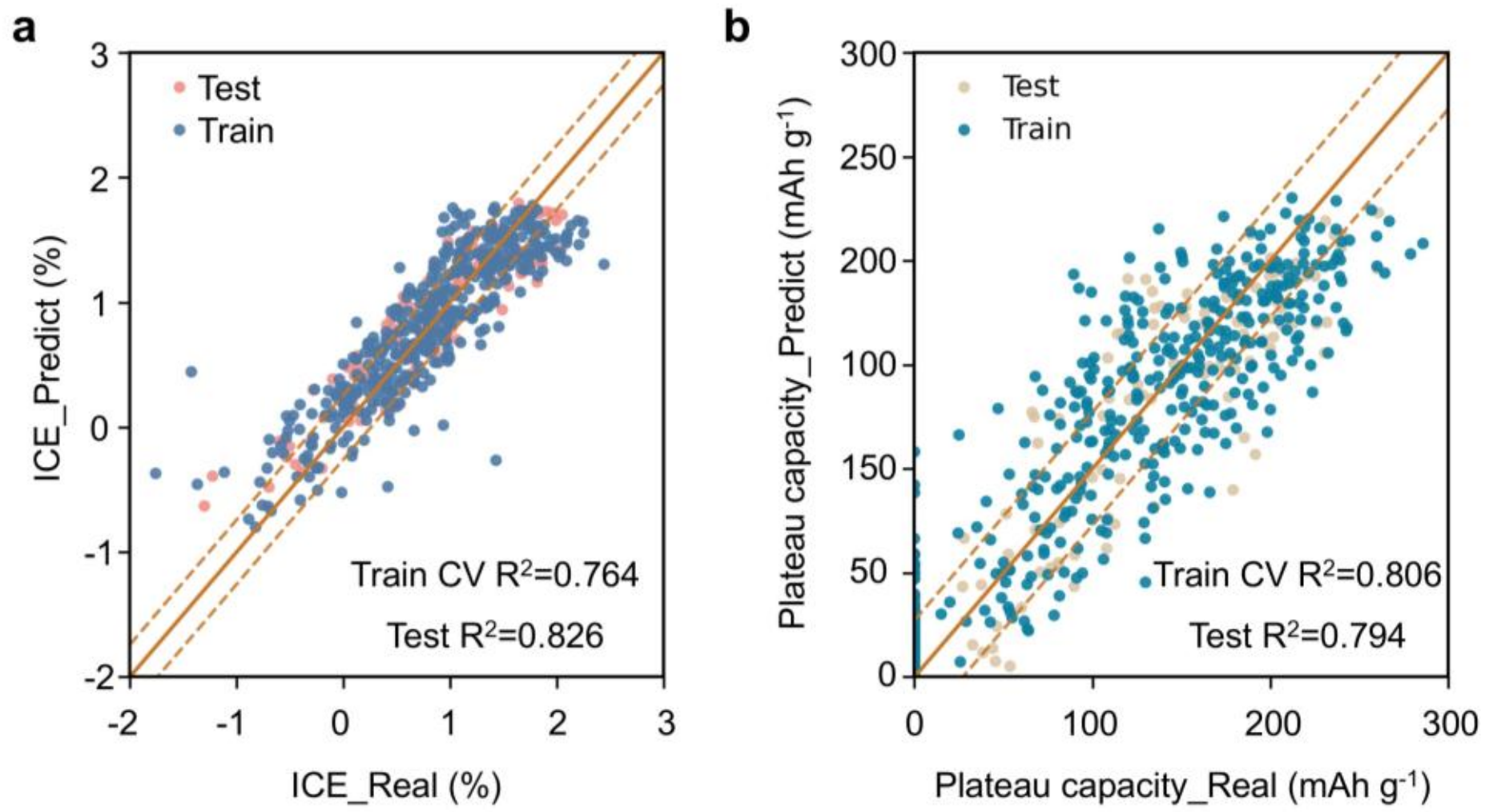

Supplementary Fig. 4. Performance of using the Random Forest (RF) model on the training data of (a) ICE and (b) reversible plateau capacity.

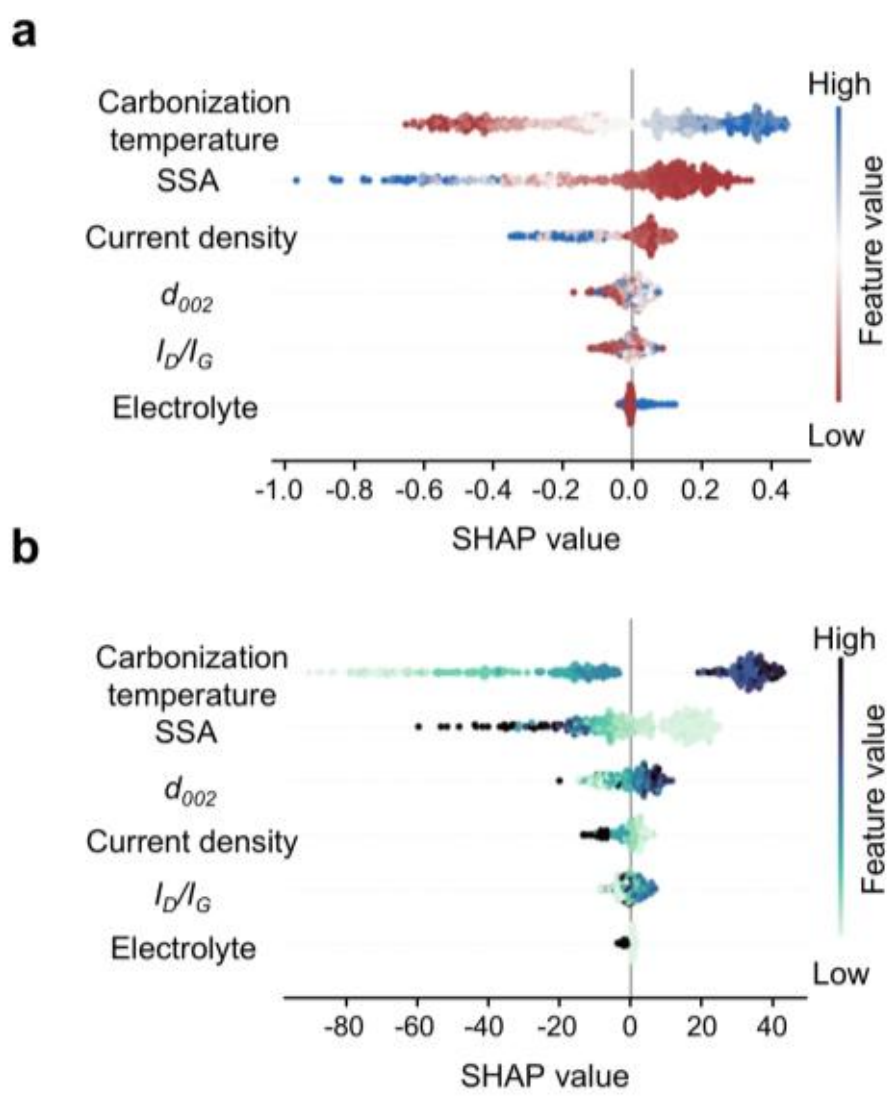

Supplementary Fig. 5. The SHAP analysis honeycomb diagram of (a) ICE and (b) reversible plateau capacity.

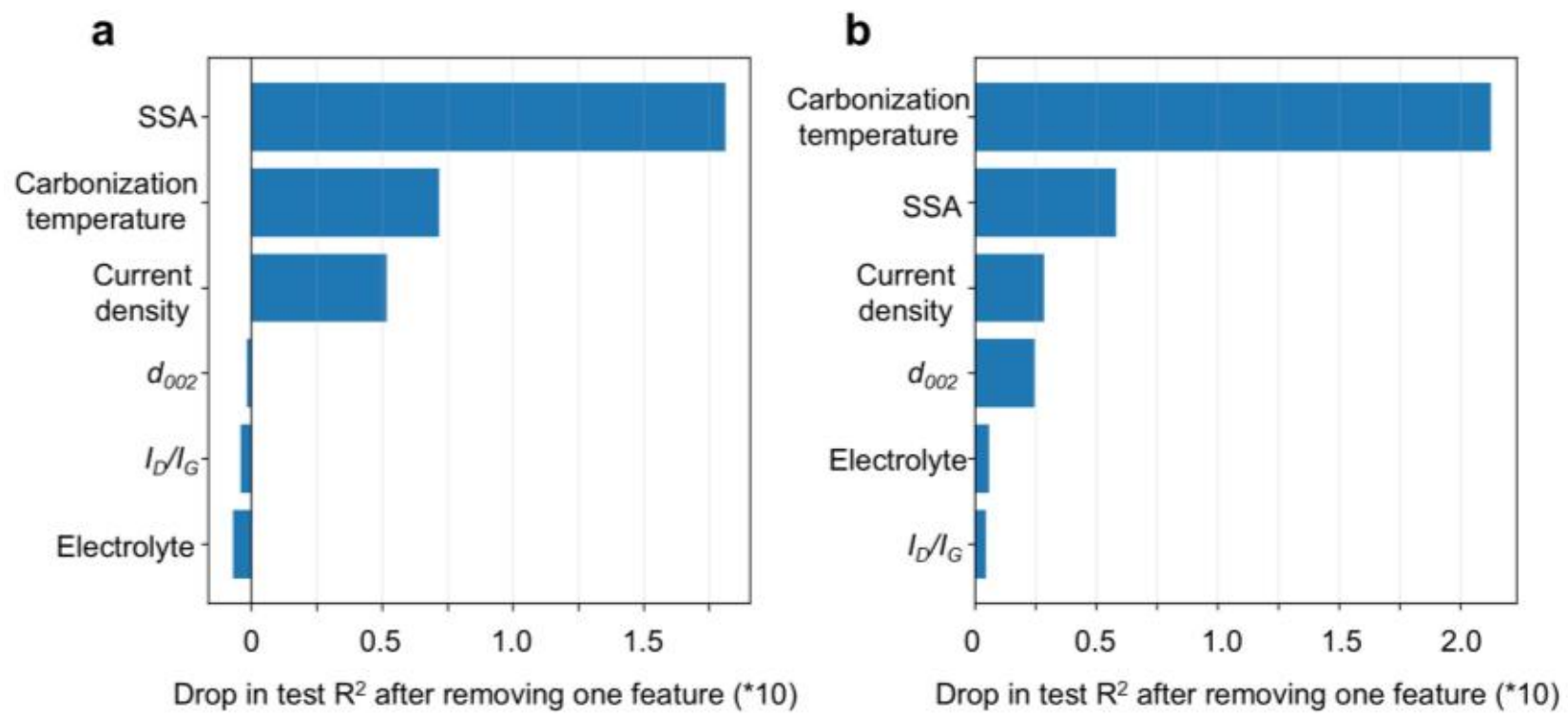

Supplementary Fig. 6. Robustness results for the leave-one-feature-out of the locked RF model in (a) ICE and (b) reversible plateau capacity.

Supplementary Note 1: For ICE, in the leave-one-feature-out analysis of the locked RF model, removing SSA causes the test R^2 to decrease by 0.181, removing carbonization temperature causes it to decrease by 0.072, and removing specific current causes it to decrease by 0.052, which is highly consistent with the main ranking in SHAP. For reversible plateau capacity, in the leave-one-feature-out analysis of the locked RF model, carbonization temperature is the most influential factor, removing this feature causes the test R^2 to decrease by 0.213. SSA is the next most influential factor, followed by specific current and d_{002} as the second-tier variables. Therefore, through dual validation via SHAP interpretive analysis and feature ablation experiments, the reliability of the key influencing factors for various electrochemical performance metrics has been enhanced.

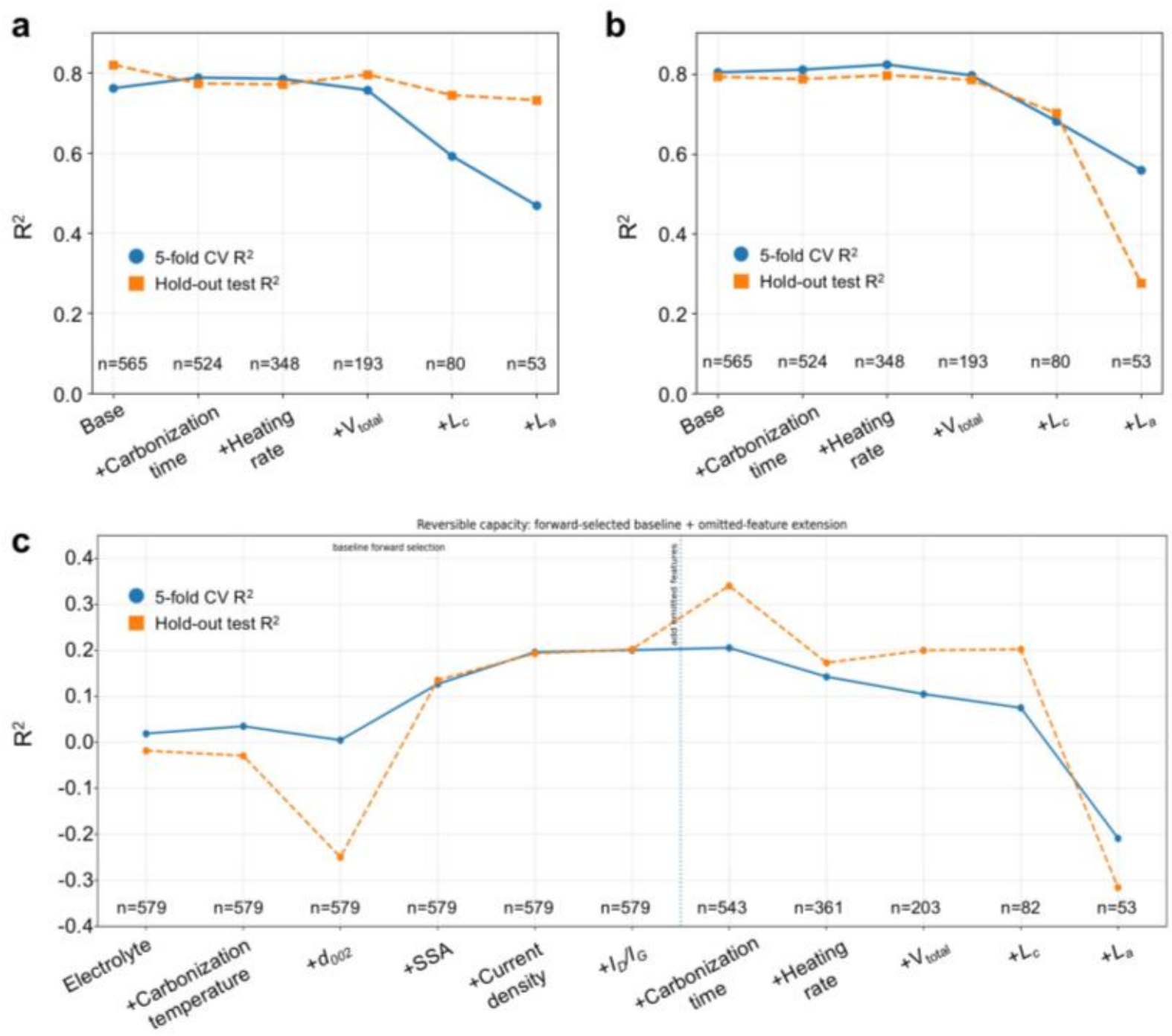

Supplementary Fig. 7. The R^2 change of the RF model after progressively adding missing variables in (a) ICE, (b) reversible plateau capacity, and (c) reversible capacity.

Supplementary Note 2: Compared to the carbonization temperature, the carbonization time and heating rate did not show a sufficiently strong and consistent independent contribution. Moreover, the proportion of missing values for V_{total}, L_c, and L_a is relatively high, and continuing to include them would rapidly reduce the number of complete samples and weaken the stability of the model. And there is significant multicollinearity among some of the variables, particularly, V_{total} overlaps significantly with SSA. Given current limitations in data coverage, independent information gain, and model stability, these variables (carbonization time, heating rate, V_{total}, L_c, and L_a) are not yet sufficient to serve as core input variables for machine learning (ML) models.

The main reason for the unclear correlation between the characteristic value and reversible capacity is that reversible capacity is contributed by two components: the slope region and the plateau region. Various structure parameters contribute to reversible capacity, meaning that a single structure parameter fails to independently explain changes in reversible capacity. There is significant multicollinearity among the variables, making it difficult for the model to disentangle the independent contributions of these correlated variables, which results in low correlation.

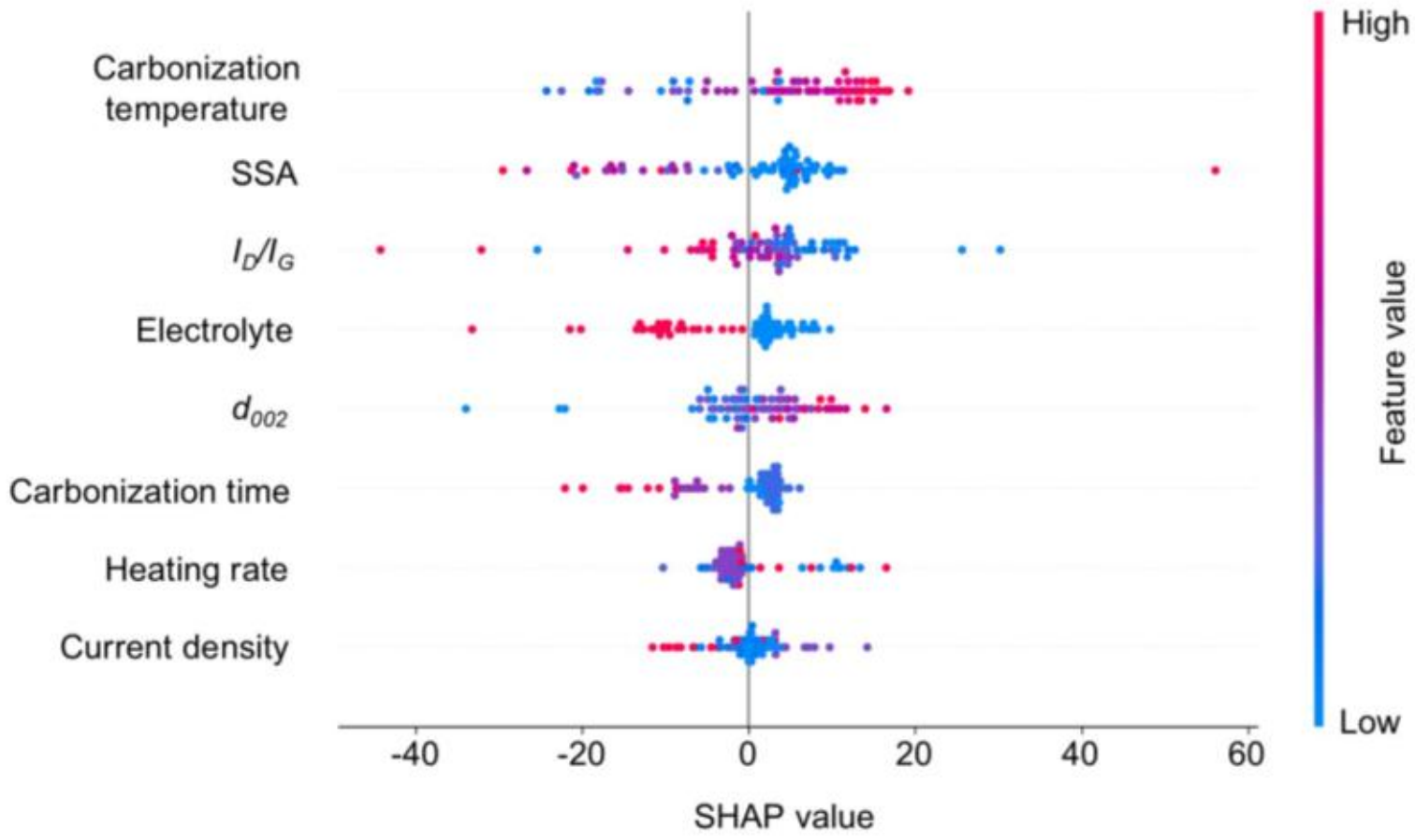

Supplementary Fig. 8. The SHAP analysis honeycomb diagram of reversible capacity.

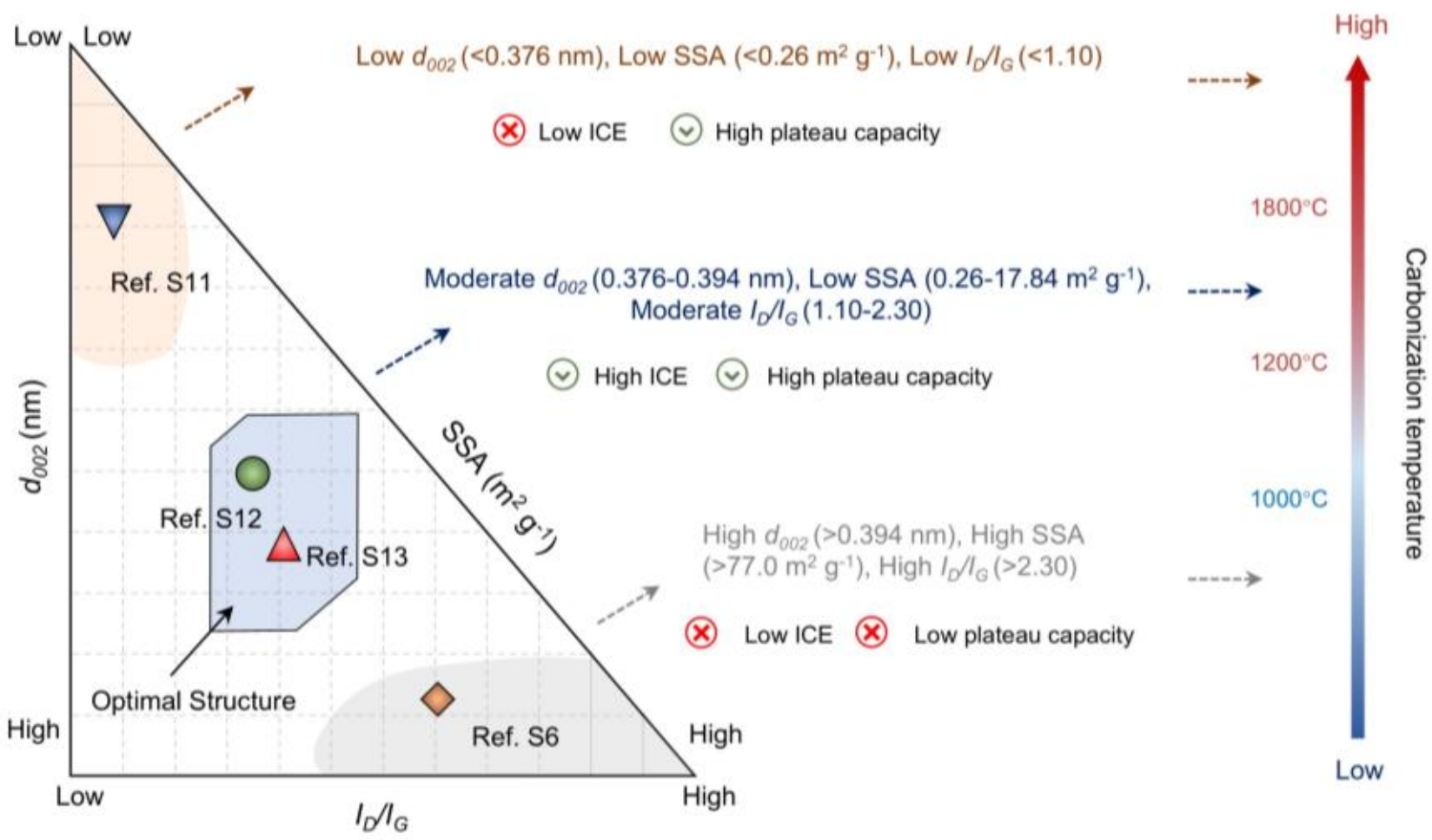

Supplementary Fig. 9. The verification of microstructure characteristics and performance of HC.

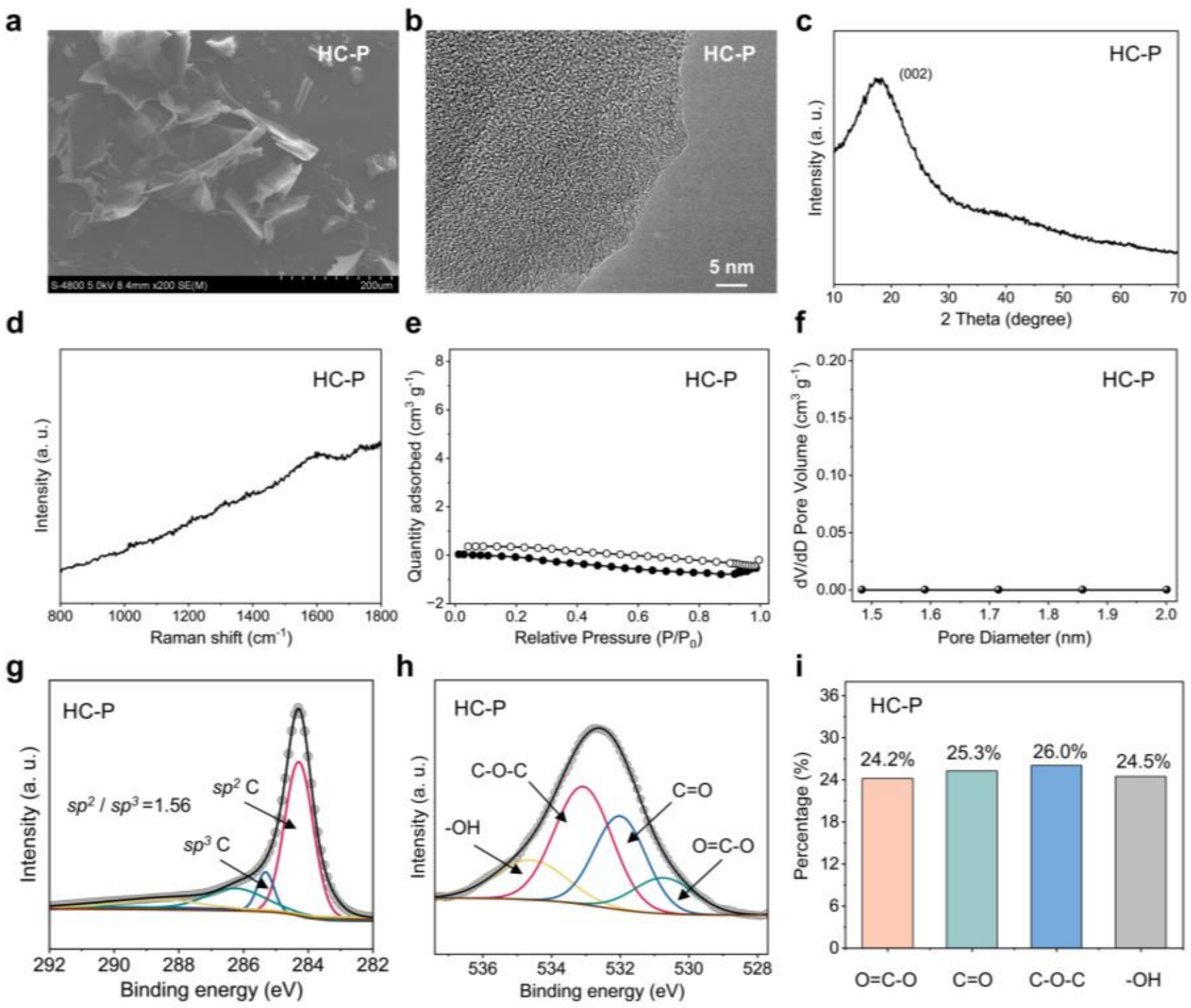

Supplementary Fig. 10. The microstructure of HC-P: (a) Scanning electron microscopy (SEM) image. (b) High-resolution transmission electron microscopy (HRTEM) image. (c) X-ray diffraction (XRD) pattern. (d) Raman spectra. (e) Nitrogen adsorption/desorption isotherms. (f) Pore size distribution. High-resolution (g) C 1*s* and (h) O 1*s* X-ray photoelectron spectroscopy (XPS) spectra. (i) The percentage of oxygen-containing functional groups.

Supplementary Note 3: Morphological and structure characterization reveal that HC-P exhibits irregular, thin carbon flakes, abundant defects, and randomly distributed, short carbon frameworks. The XRD pattern and Raman spectra further confirmed the amorphous structure of HC-P, due to significant decomposition reactions occurring during low-temperature treatment. HC-P exhibits a dense, amorphous polymer network due to dehydration-condensation during a low-temperature process, with no significant carbonization and aromatization having occurred, the material lacks a distinct pore structure. Furthermore, the sp^2/sp^3 ratio of HC-P reaches as low as 1.56 and rich oxygen-containing functional groups, indicating a low-crystallinity, unstable, and highly disordered carbon structure.

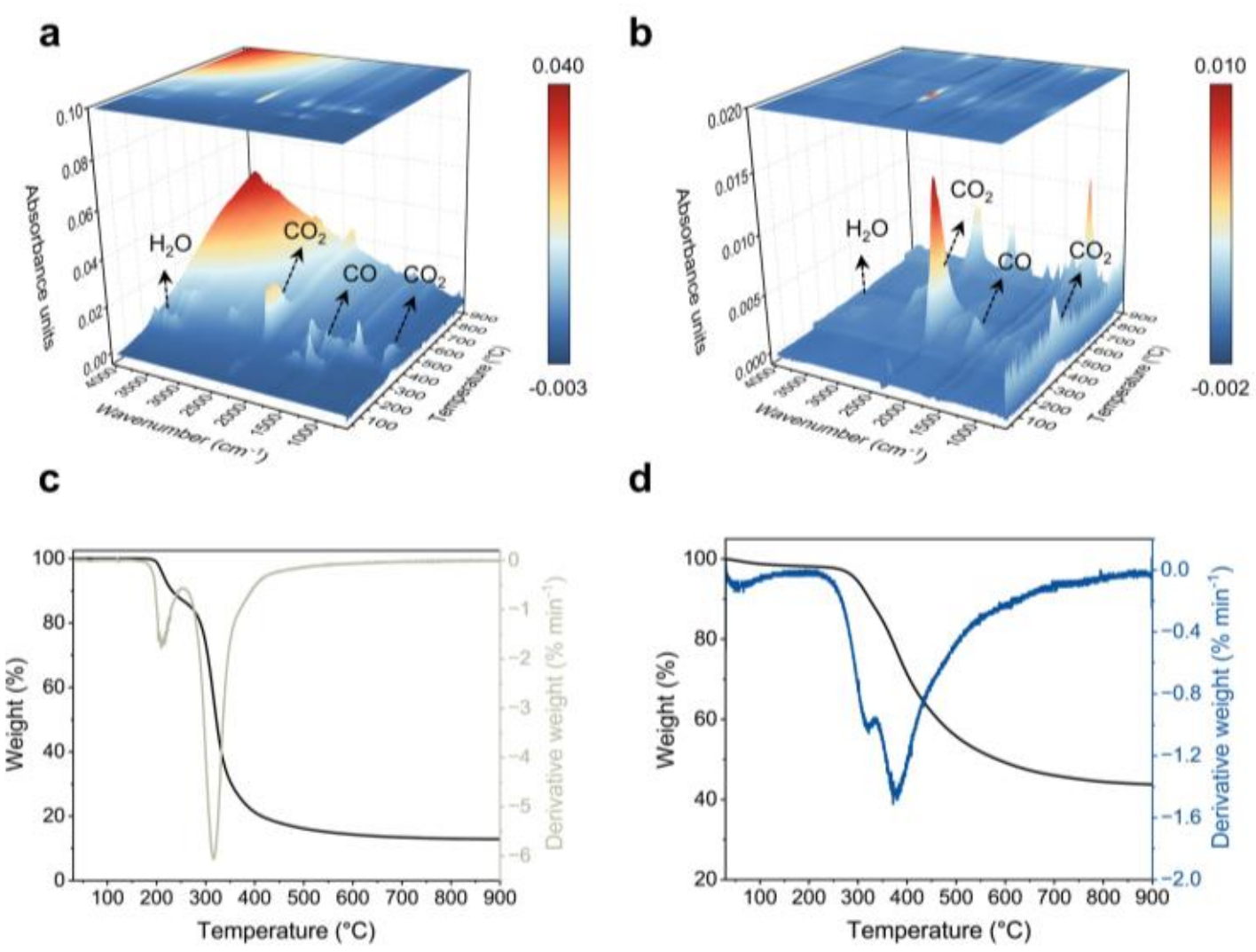

Supplementary Fig. 11. In situ thermogravimetry analysis-Fourier transform infrared (TGA-FTIR) of (a, c) the direct pyrolysis process of glucose and (b, d) the pyrolysis process of glucose after pre-carbonization.

Supplementary Note 4: In situ TGA-FTIR analysis reveals characteristic gas-phase signatures of CO_2, CO, and H_2O during pyrolysis treatment, indicating significant decomposition reactions occur throughout the pyrolysis process. During the pre-carbonization process at 300°C of glucose, characteristic gas-phase peaks of CO_2 and H_2O were detected at 550-750 cm^{-1}, 2250 to 2500 cm^{-1}, and 2750-3750 cm^{-1}, indicating significant decomposition reactions resulting from dehydration between hydroxyl groups and the breaking of carbon-oxygen bonds. Furthermore, as the carbonization temperature increases to 900°C, the amount of CO_2 gas released continues to rise, and the decline in material quality becomes more pronounced. In contrast, when glucose undergoes pre-carbonization at 300°C followed by high-temperature carbonization at 900°C, CO_2 emissions are reduced, and carbon yield is increased.

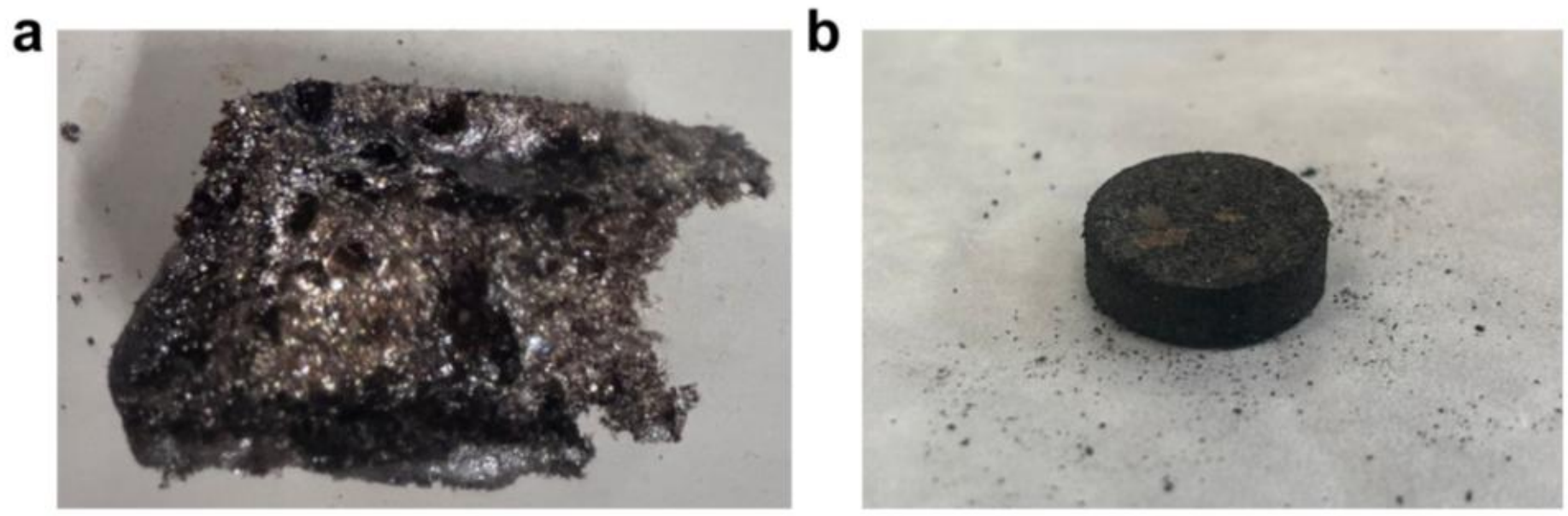

Supplementary Fig. 12. The digital images of the HC-P at (a) atmospheric pressure and (b) hot pressing.

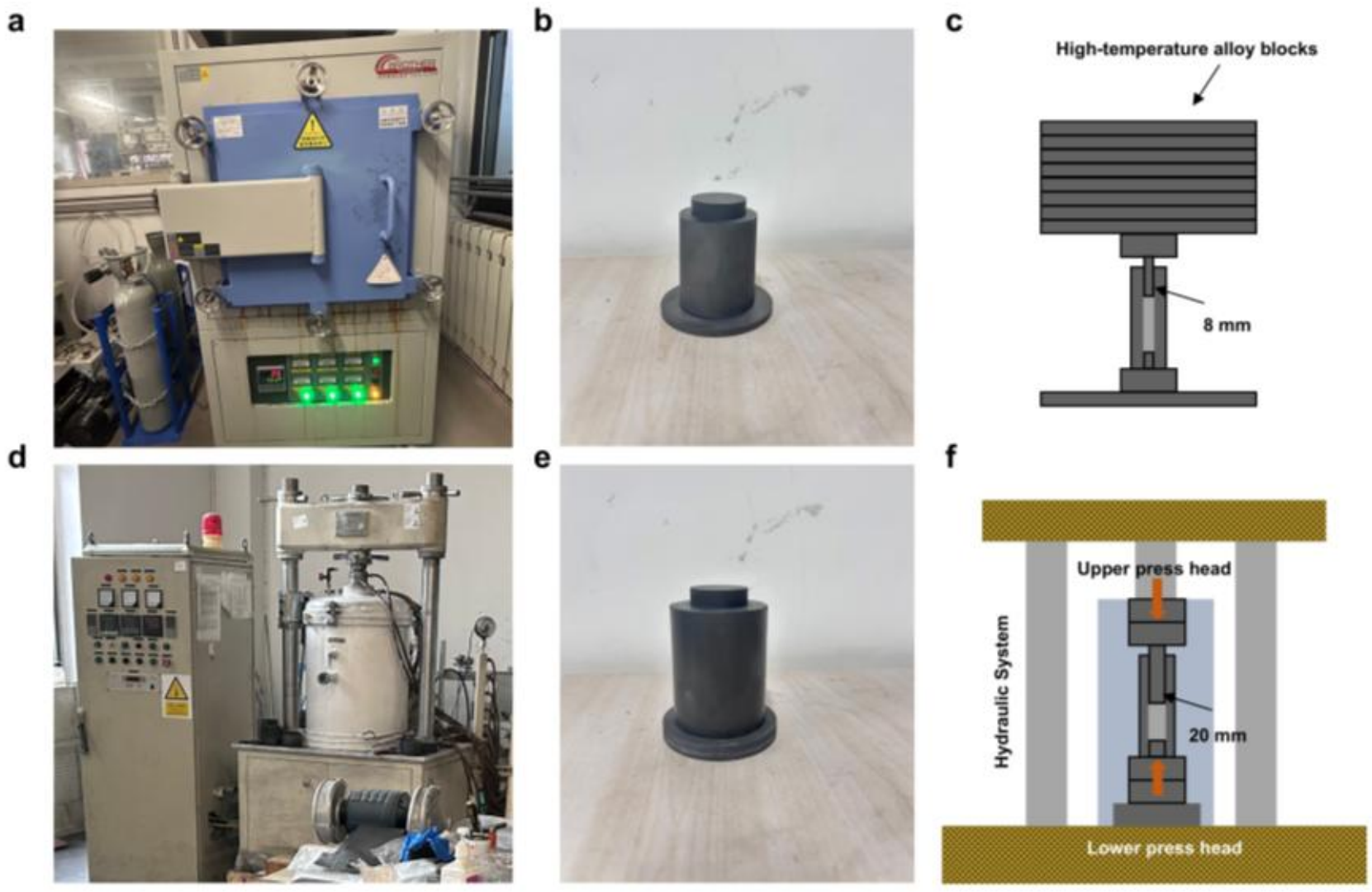

Supplementary Fig. 13. (a-c) Schematic diagram of the preparation of HC-5-900 and HC-10-900 in a muffle furnace. (d-f) Schematic diagram of the preparation of HC-50-900 and HC-100-900 in a hot-press carbonization furnace.

Supplementary Note 5: To standardize equipment types for subsequent economic analysis, a thermal-force coupling carbonization experiment of HC-5-900 and HC-10-900 was conducted in a muffle furnace with an argon atmosphere. High-temperature alloy blocks resistant to 1100°C are placed on graphite molds to apply pressure during the carbonization process. The diameter of the graphite mold press head is 8 mm. During the preparation of HC-5-900, a 25.6 kg high-temperature alloy block was used to apply 5 MPa of pressure on the material within the graphite mold. During the preparation of HC-10-900, a 51.3 kg high-temperature alloy block was used to apply 10 MPa of pressure on the material within the graphite mold.

To systematically investigate the effect of pressure on material structure, high-pressure experiments of HC-50-900 and HC-100-900 were conducted in a hot-press carbonization furnace. The diameter of the graphite mold press head is 20 mm. During the preparation of HC-50-900, the graphite upper press head and lower press head were driven by a pressurizing device to apply 50 MPa pressure to the material in the mold. During the preparation of HC-100-900, the graphite upper press head and lower press head were driven by a pressurizing device to apply 100 MPa pressure to the material in the mold.

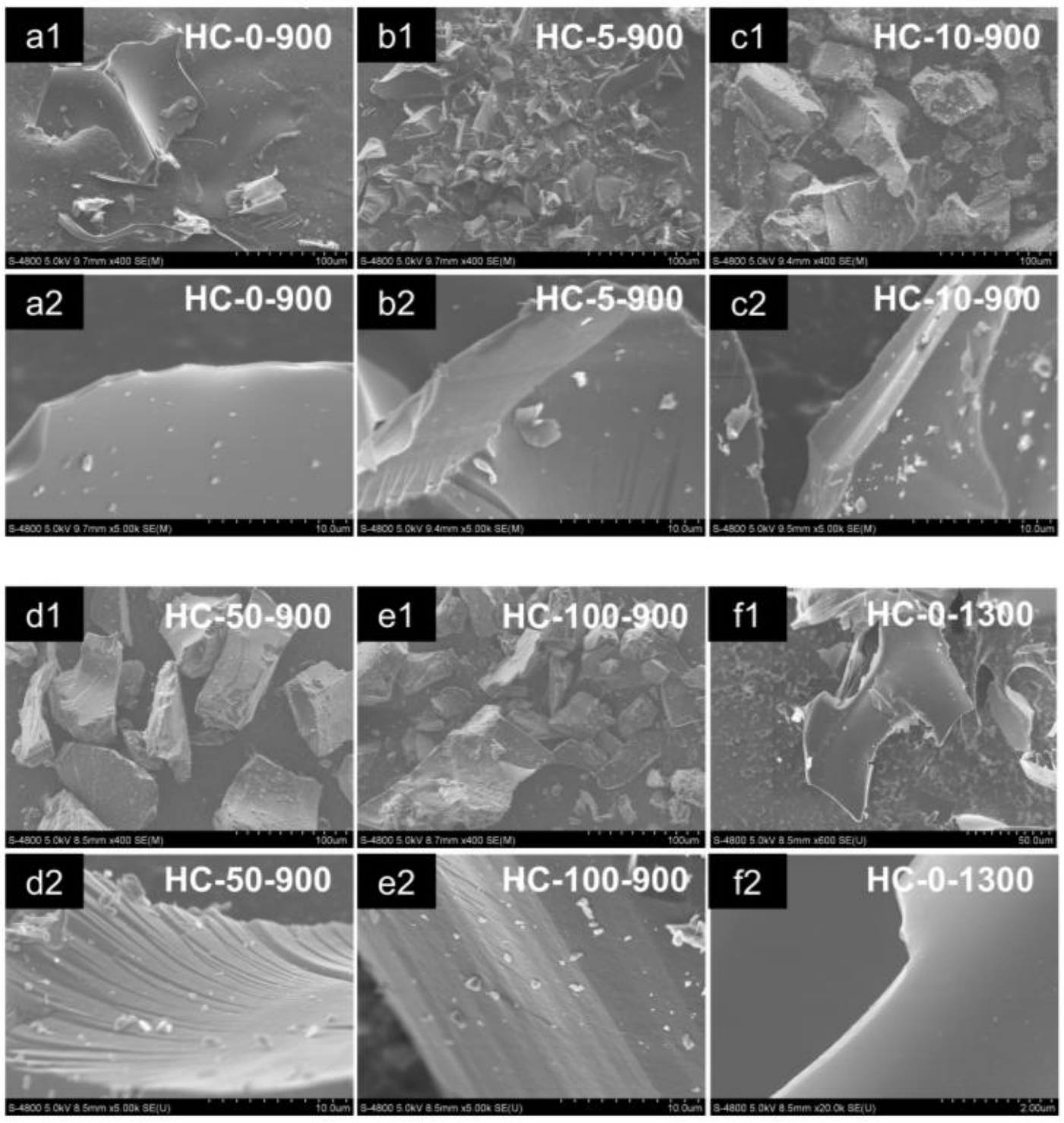

Supplementary Fig. 14. The SEM images of (a) HC-0-900, (b) HC-5-900, (c) HC-10-900, (d) HC-50-900, (e) HC-100-900, and (f) HC-0-1300.

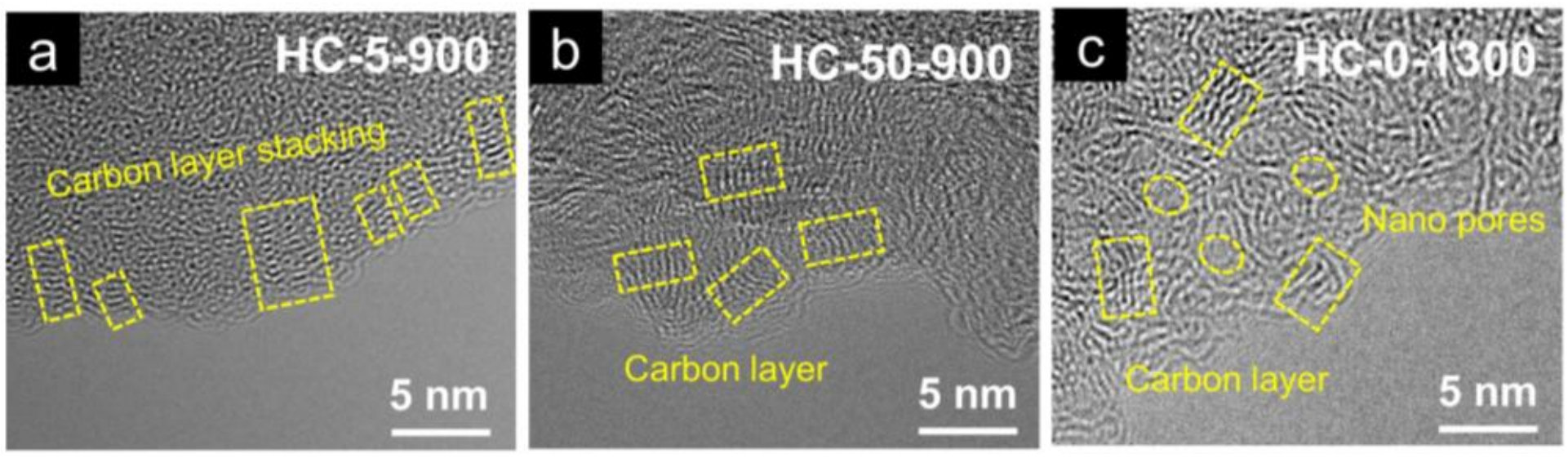

Supplementary Fig. 15. HRTEM images of (a) HC-5-900, (b) HC-50-900, and (c) HC-0-1300.

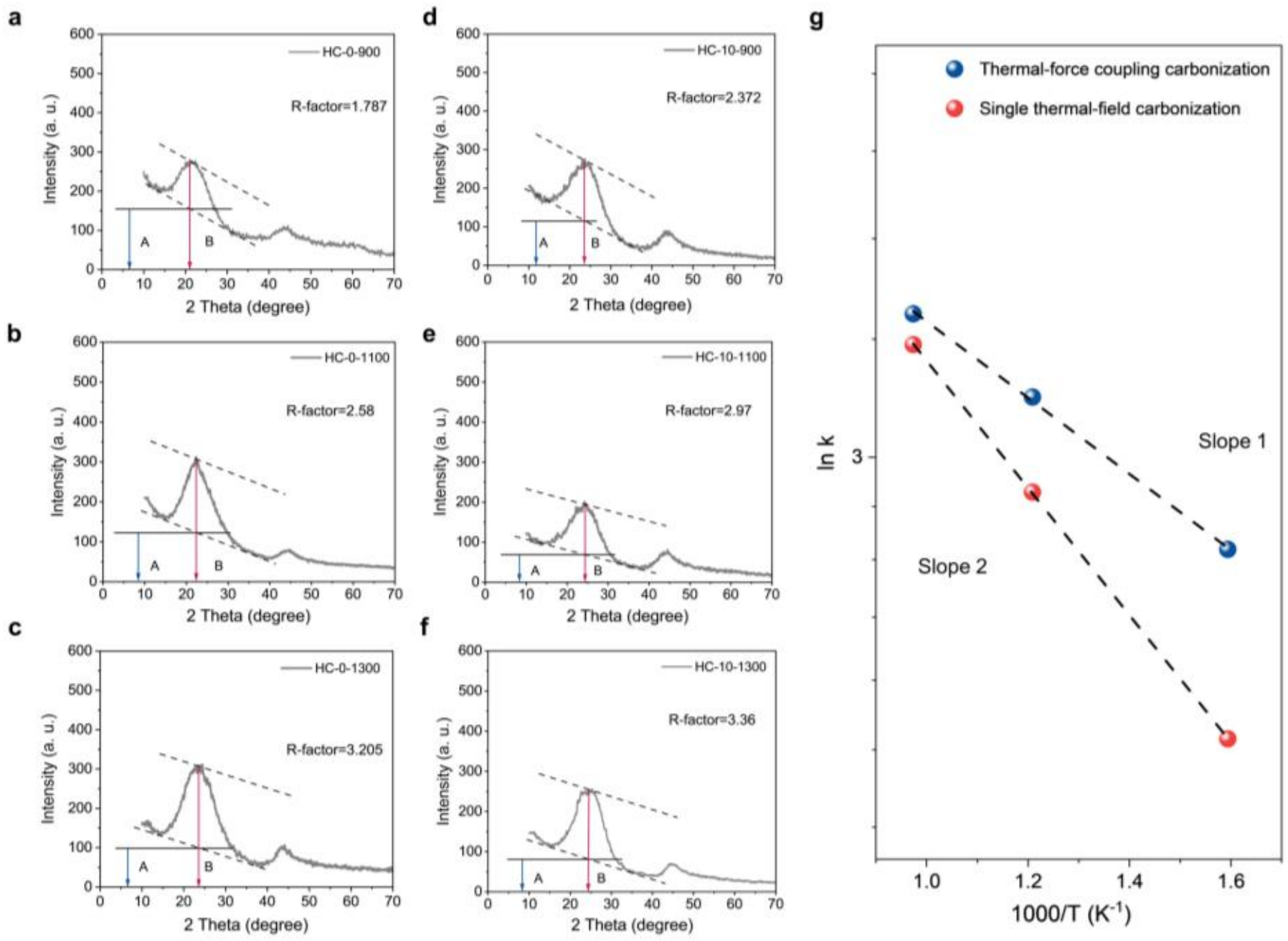

Supplementary Fig. 16. The R-factor of (a) HC-0-900, (b) HC-0-1100, (c) HC-0-1300, (d) HC-10-900, (e) HC-10-1100, and (f) HC-10-1300. (g) Comparison of activation energies of thermal-force coupling carbonization and single thermal-field carbonization.

Supplementary Note 6: According to the Arrhenius equation, a set of rate constants k corresponding to different temperatures T (900, 1100, 1300℃) is measured at fixed pressure P (Atmospheric pressure and 10 MPa). The slope is then determined based on the linear relationship between ln k and 1/T, thereby yielding the apparent activation energy at that pressure.

Arrhenius equation: $$k(P,T) = A(P)exp\left[-\frac{E_aP}{RT}\right] \quad (1)$$

Take the natural logarithm: $$\ln k(P,T) = \ln A(p) - \frac{E_aP}{R}\frac{1}{T} \quad (2)$$

Here, the degree of graphitization of HC is converted into a rate constant (k). The graphitization degree (R-factor) is obtained by measuring the ratio between the (002) peak intensity (B) and the background intensity (A) in the XRD pattern[14]. The low R factor indicates that under the formation conditions, the graphene layers lacked sufficient thermal energy to rotate into a parallel alignment. Calculations show that the slope of the thermal-force coupling process is significantly smaller than that of the conventional pyrolysis process, indicating that the additional pressure significantly reduces the activation energy required for the ordered rearrangement of the carbon layer.

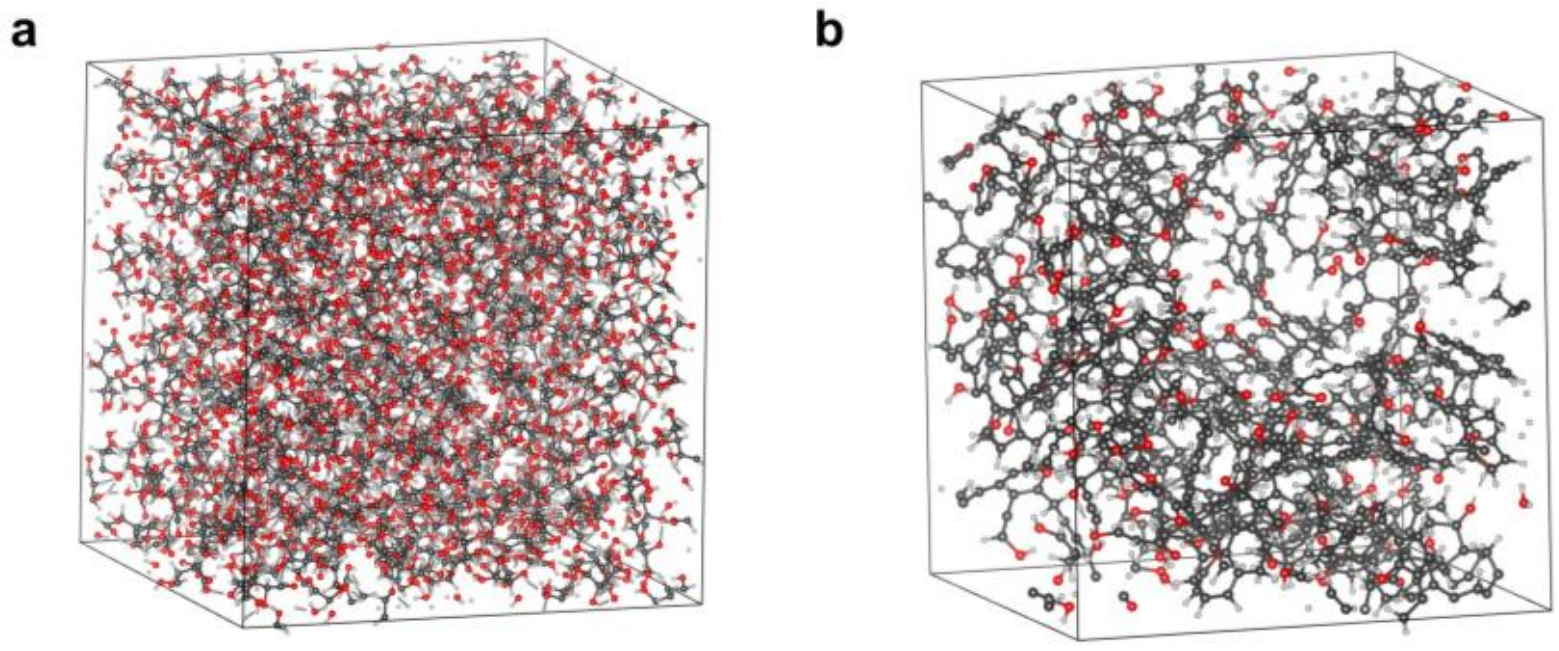

Supplementary Fig. 17. (a) Initial model of glucose and (b) model of HC-P after pre-carbonization (Black represents carbon atoms, gray represents hydrogen atoms, and red represents oxygen atoms).

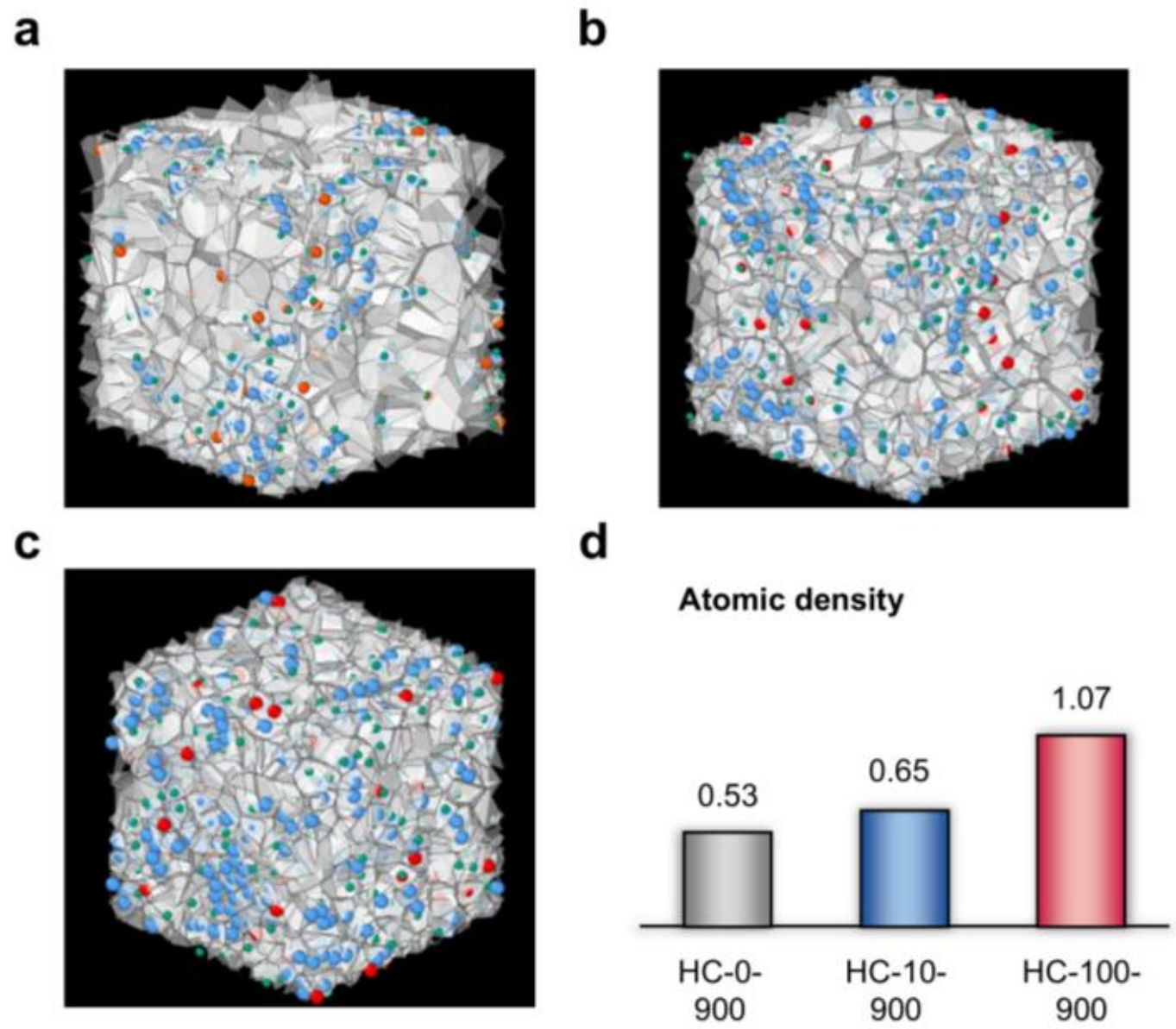

Supplementary Fig. 18. (a-c) Pore structure visualization of HC-0-900, HC-10-900, and HC-100-900 models. (d) Atomic density of HC-0-900, HC-10-900, and HC-100-900 models.

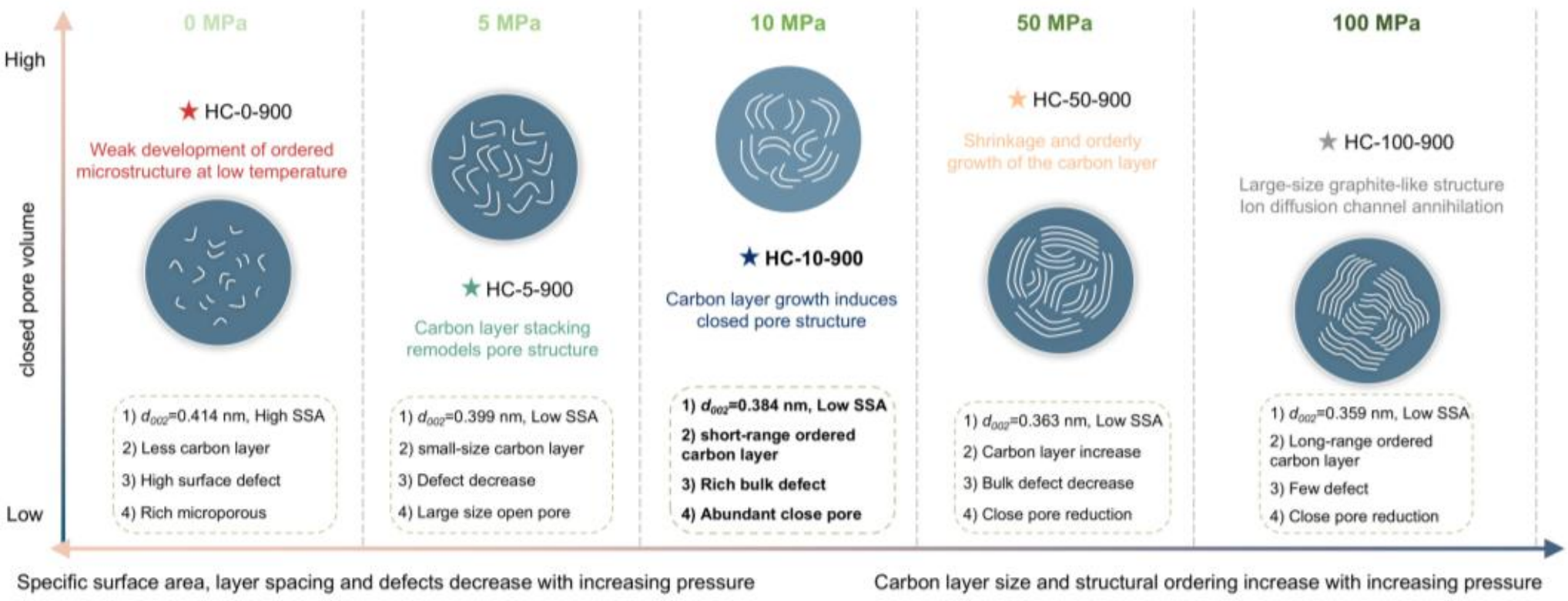

Supplementary Fig. 19. The evolution mechanism of HC microstructure under the low-temperature thermal-force coupling carbonization (LTFC).

Supplementary Note 7: The evolution mechanism of the HC microstructure under the LTFC was analyzed by adjusting the pressure. Under low-temperature thermal field carbonization (LTC), the carbon matrix undergoes significant decomposition reactions, resulting in the breaking of carbon-oxygen bonds and the release of decomposition gases, which hinder the orderly cross-linking of the carbon framework. This results in the HC-0-900 exhibiting high I_D/I_G, high SSA, high d_{002}, randomly distributed carbon flakes, and abundant micropores. During low-temperature and low-pressure coupling carbonization, the applied pressure creates a spatially confined carbonization environment, promoting the ordered cross-linking of carbon atoms and the formation of carbon layers. The stacking of small carbon layers induces the formation of high-diameter open pores. As the pressure increased to 10 MPa, the carbon layers were further compressed and grew in an ordered manner, inducing the formation of a closed-cell structure between the curved carbon layers. When the pressure reaches 50 MPa, the carbon layer size further increases and its curvature decreases, resulting in a reduction in the number and volume of closed pores. Under the high pressure of 100 MPa, the carbonization space is drastically compressed, accelerating the rate of ordered cross-linking among carbon atoms, thereby inducing the formation of long-range ordered graphite-like carbon layers. This also eliminates defects and closed pores, thereby blocking the diffusion pathways for Na^+. Among these, the HC-10-900 provides wide layer spacing, a suitable carbon layer size, and abundant bulk defects under LTFC, which is the microstructure basis for achieving excellent overall electrochemical performance.

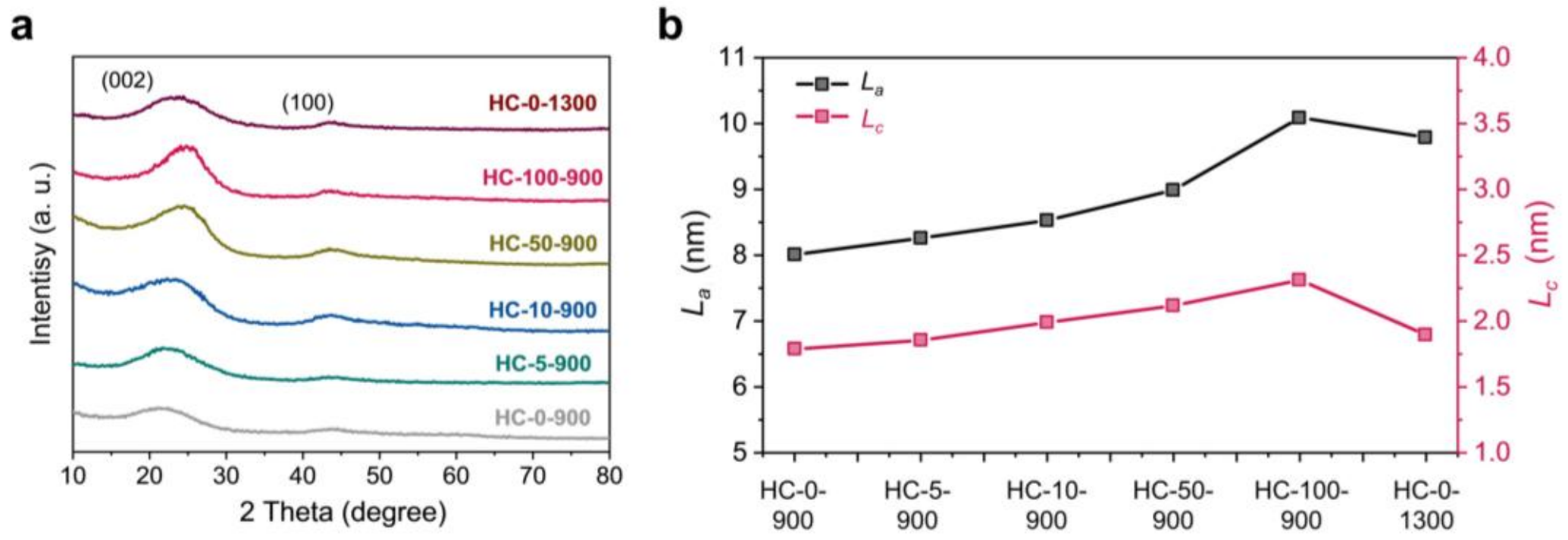

Supplementary Fig. 20. (a) XRD pattern and (b) L_a, L_c of HC-0-900, HC-5-900, HC-10-900, HC-50-900, HC-100-900, and HC-0-1300.

Supplementary Note 8: L_a: lateral size of the crystallite was calculated by the following equation[2]: L_a (nm)=Kλ/βsinθ (K=0.89, β is half width of (100) diffraction peak, λ =0.154056 nm). L_c: stacking height of the crystallite was calculated by the following equation: L_c (nm)= Kλ/βcosθ (K=0.89, β is half width of (002) diffraction peak, λ =0.154056 nm). d_{002}: layer spacing was calculated by the Bragg equation: d_{002} =nλ/2sinθ, θ is the incoming ray, the included angle between the reflection ray and the reflected crystal plane, λ is the wavelength, and n refers to the reflection series.

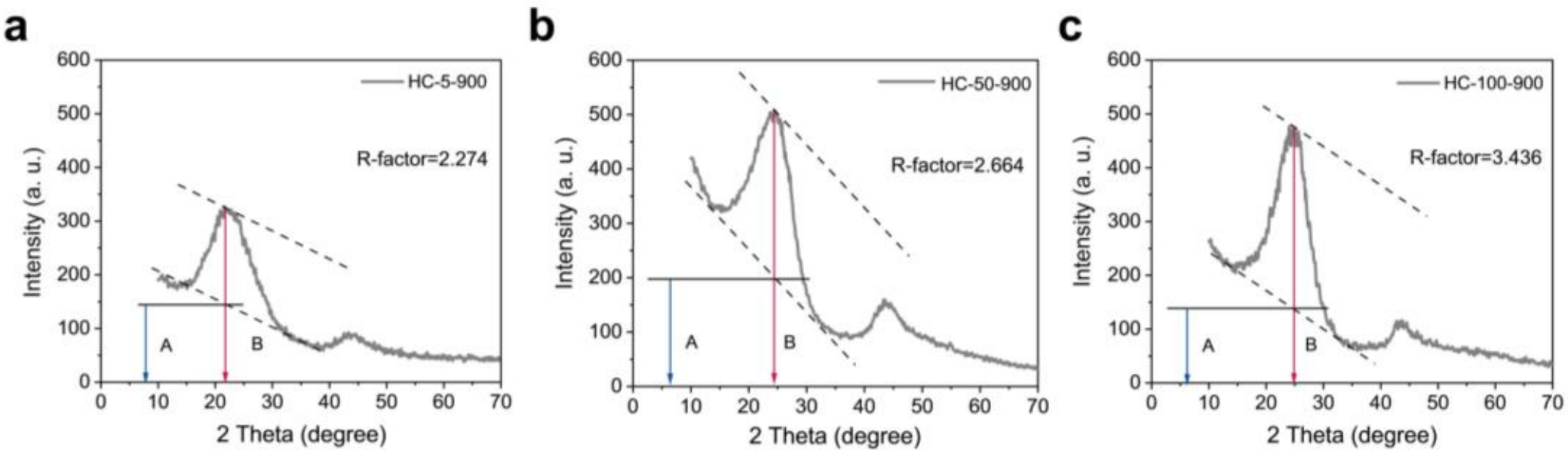

Supplementary Fig. 21. (a-c) R-factor of HC-5-900, HC-50-900, and HC-100-900.

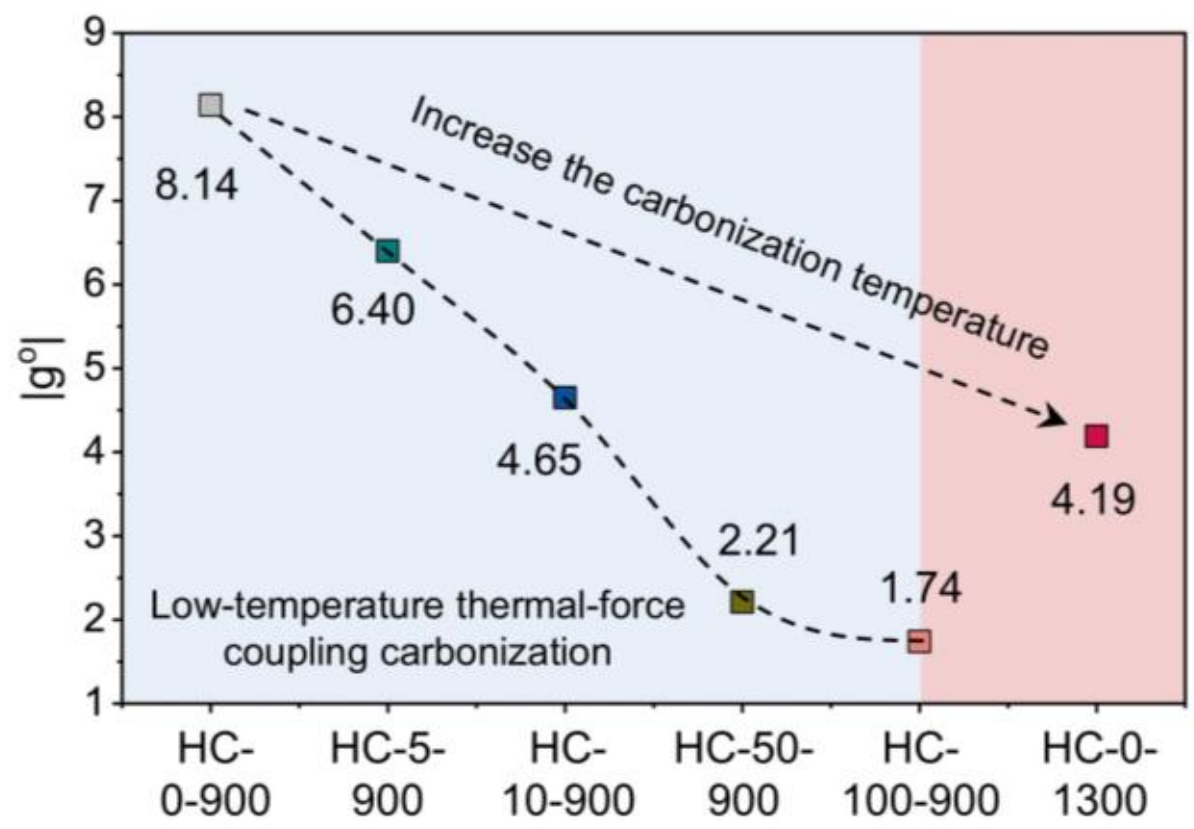

Supplementary Fig. 22. The |g°| of HC-0-900, HC-5-900, HC-10-900, HC-50-900, HC-100-900, and HC-0-1300.

Supplementary Note 9: The accurate position of the 002 peak of HC was measured by XRD tests. The d_{002} of HC was calculated by the Bragg equation. Thus, the graphitization degree could be calculated using formula[15].

$$g^o = (3.440 - d_{002})/(3.440 - 3.354) \quad (3)$$

Where g is the graphitization degree. The 3.440 is the interplanar spacing in carbon with a turbostratic structure. The d_{002} is the value of interlayer spacing of graphite obtained by XRD characterization, and the 3.354 is the interplanar spacing in an ideal defect-free crystal of graphite. (units are in Å)

In this work, the absolute value of g° is used to assess the degree of graphitization in HC materials. And the smaller the absolute value of g°, indicating a higher degree of graphitization. The |g°| of HC-0-900, HC-5-900, HC-10-900, HC-50-900, and HC-100-900 are 8.14, 6.40, 4.65, 2.21, and 1.74, respectively. This further indicates that as pressure increases, the degree of graphitization of HC gradually increases.

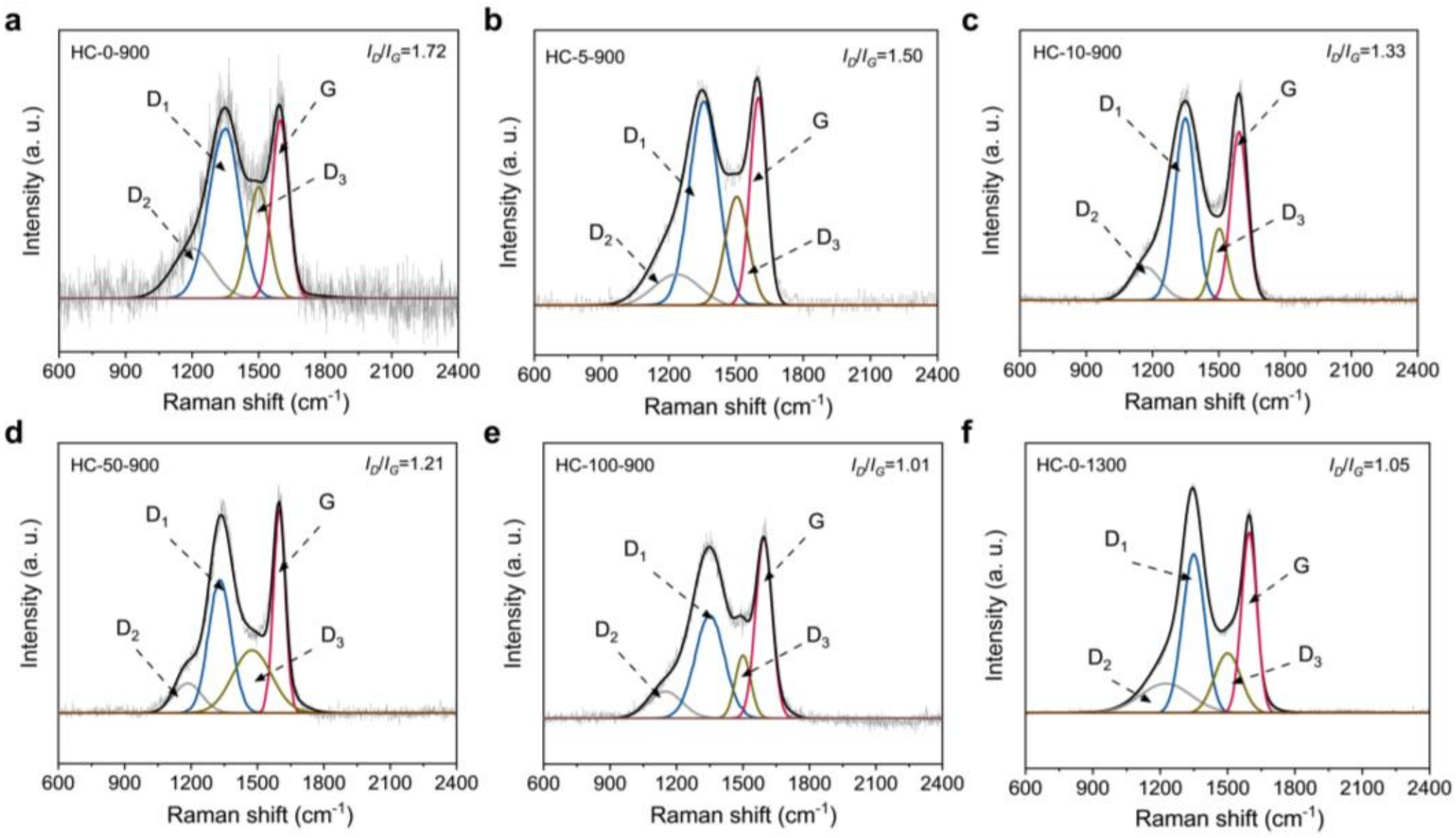

Supplementary Fig. 23. Raman spectra of (a) HC-0-900, (b) HC-5-900, (c) HC-10-900, (d) HC-50-900, (e) HC-100-900, and (f) HC-0-1300.

Supplementary Note 10: The multi-peak fitting of the Raman spectrum is performed by the Gaussian fitting function of Origin software. Based on the Lorentz function, Raman spectra of carbon materials were further fitted with D_1 (1345 cm^{-1}), D_2 (1200 cm^{-1}), D_3 (1500 cm^{-1}), and G band (1590 cm^{-1})[16]. The D_1 and G bands correspond to defect structure and graphitic lattice within graphene layers, respectively. The D_2 and D_3 bands correspond to changes in defects and oxygen-containing groups growing along the carbon edge or carbon layer, respectively.

The I_D/I_G of HC-0-900, HC-5-900, HC-10-900, HC-50-900, HC-100-900, and HC-0-1300 are 1.72, 1.50, 1.33, 1.21, 1.01, and 1.05 by using the integrated areas of the D and G peaks, respectively. The high I_D/I_G of HC-0-900 indicates a rich defect structure. This is attributed to the weak ordered cross-linking reaction and significant decomposition reaction during low-temperature carbonization. As pressure increases, the I_D/I_G of the HC decreases sequentially, indicating that surface-exposed defects are reduced due to the densification of the material. Among these, ordered carbon layers and appropriate defects of HC-10-900 form a Na^+ diffusion network, which improves the diffusion kinetics of Na^+. Compared to force fields, further increasing the carbonization temperature also promotes graphitization development of HC, yet this process is difficult to control. HC-0-1300 exhibits a low I_D/I_G, and its low-defect and highly ordered structure increases the resistance to Na^+ diffusion.

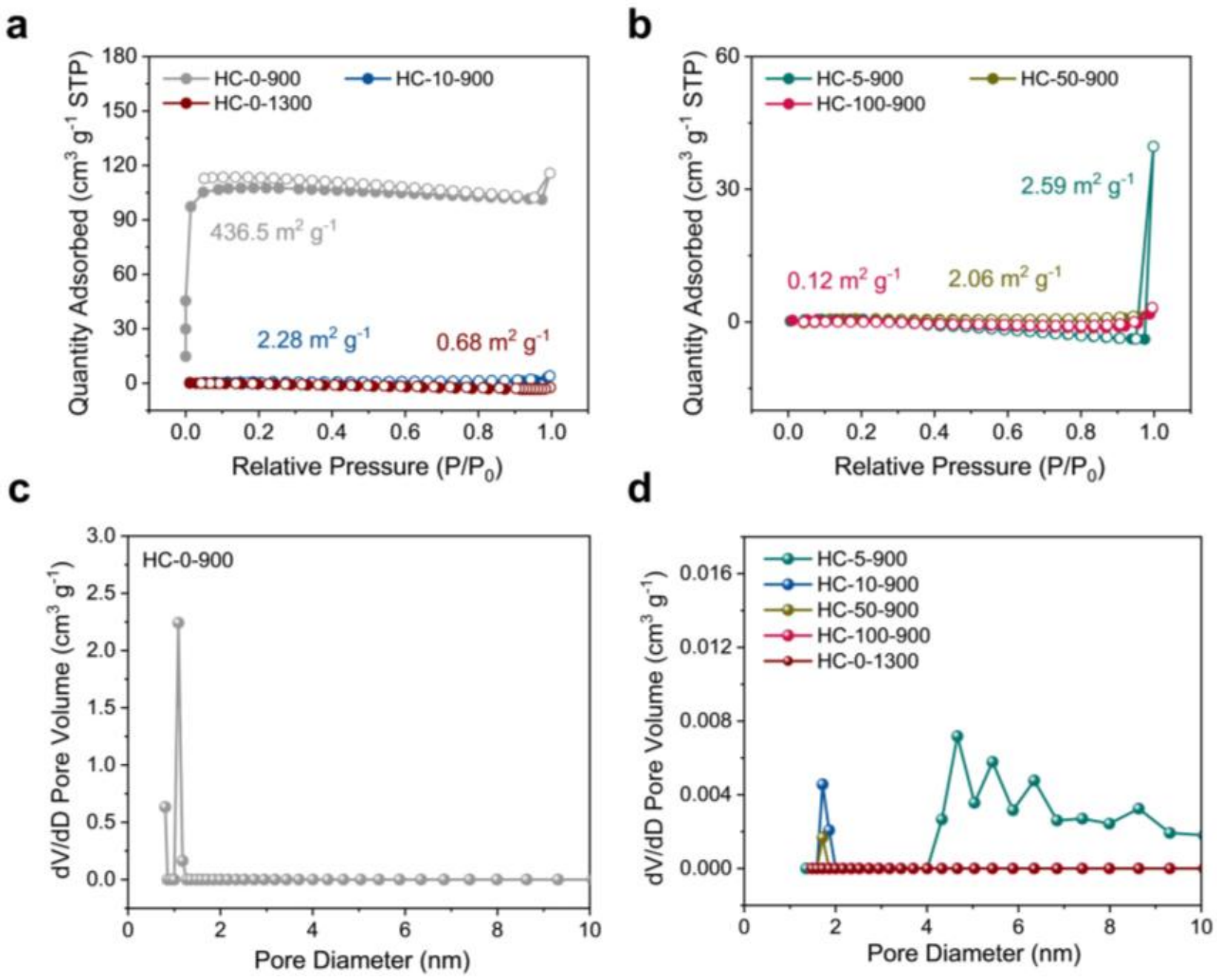

Supplementary Fig. 24. (a, b) Nitrogen adsorption/desorption isotherms of HC-0-900, HC-5-900, HC-10-900, HC-50-900, HC-100-900, and HC-0-1300. (c, d) Pore size distribution of HC-0-900, HC-5-900, HC-10-900, HC-50-900, HC-100-900, and HC-0-1300.

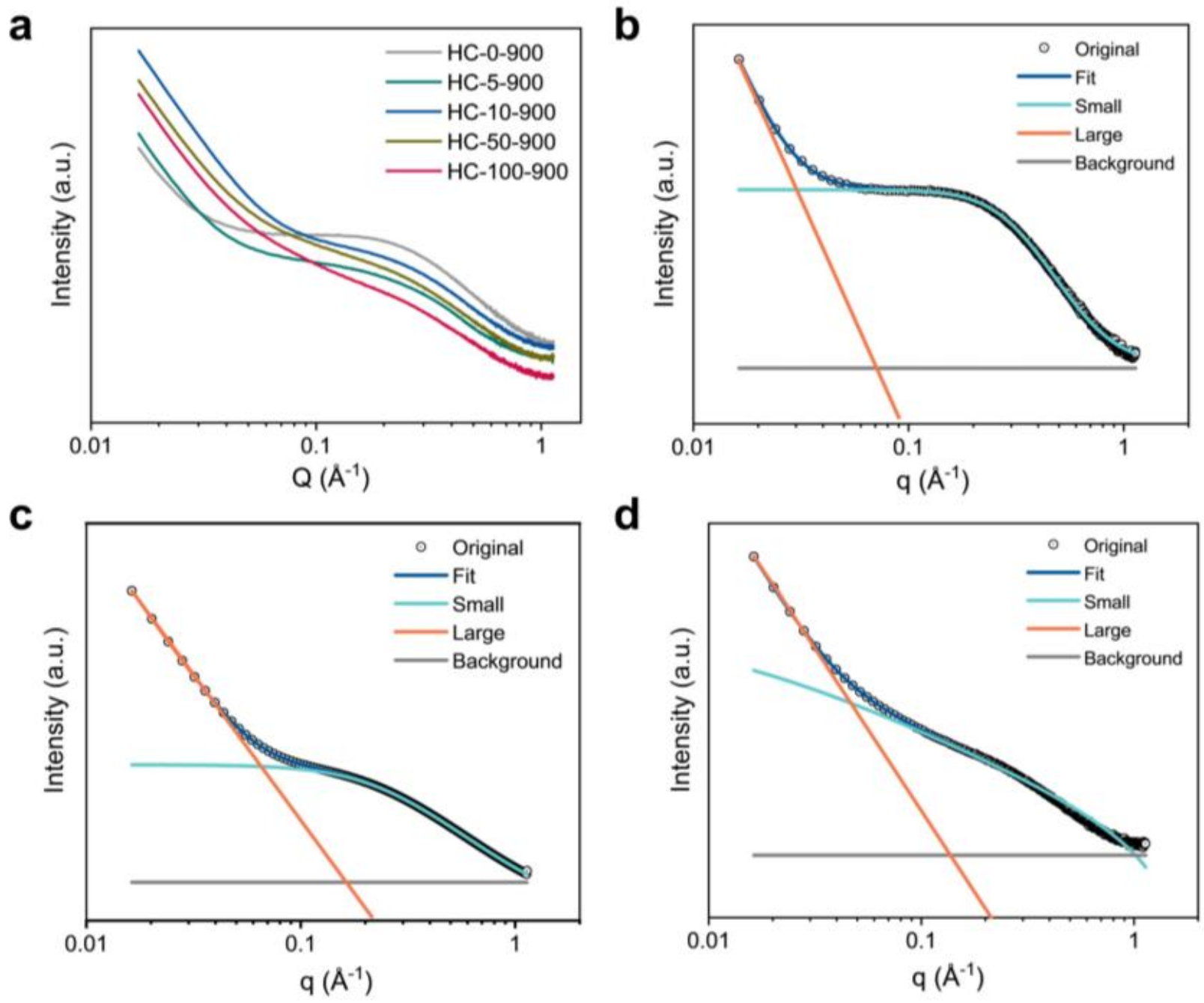

Supplementary Fig. 25. (a) Small-angle X-ray scattering (SAXS) pattern of HC-0-900, HC-5-900, HC-10-900, HC-50-900, and HC-100-900. (b-d) SAXS patterns presenting pores of HC-0-900, HC-10-900, and HC-100-900.

Supplementary Note 11: SAXS Analysis

Porod's law is one of the basic formulae in SAXS, which mainly describes the asymptotic behavior of the intensity as a function of scattering vector.

It is well known that only the scattering by an ideal two-phase system with a sharp boundary and with constant but different electron density in each phase obeys the Porod's law[17]:

$$lim q^4 I(q) = K \qquad (4)$$

where q is the scattering vector, q=4πsinθ/λ, 2θ is the scattering angle, λ is the incident X-ray wavelength, I(q) is the point-collimation (pinhole) scattering intensity, and K is the Porod constant. The specific surface (total surface per unit of volume) of the sample can be calculated by the Porod method[18]:

$$S_V = \pi P(1-P)\frac{K}{Q} \qquad (5)$$

where K is the Porod constant. P is the volume fraction of the dispersed phase, for porous systems, P is called porosity, and it is usually derived from the densities of the sample or the absolute intensity of the primary beam; Q is the invariant, which is given by:

$$Q = \int_0^\infty q^2 I(q) dq \qquad (6)$$

Q should be calculated by extrapolating scattering intensity towards zero and infinitely large scattering angle, i.e., into regions where no experimental data are available. The first extrapolation can be done by applying the Guinier approximation, and the second extrapolation can be done by applying Porod's q^{-4} dependence.

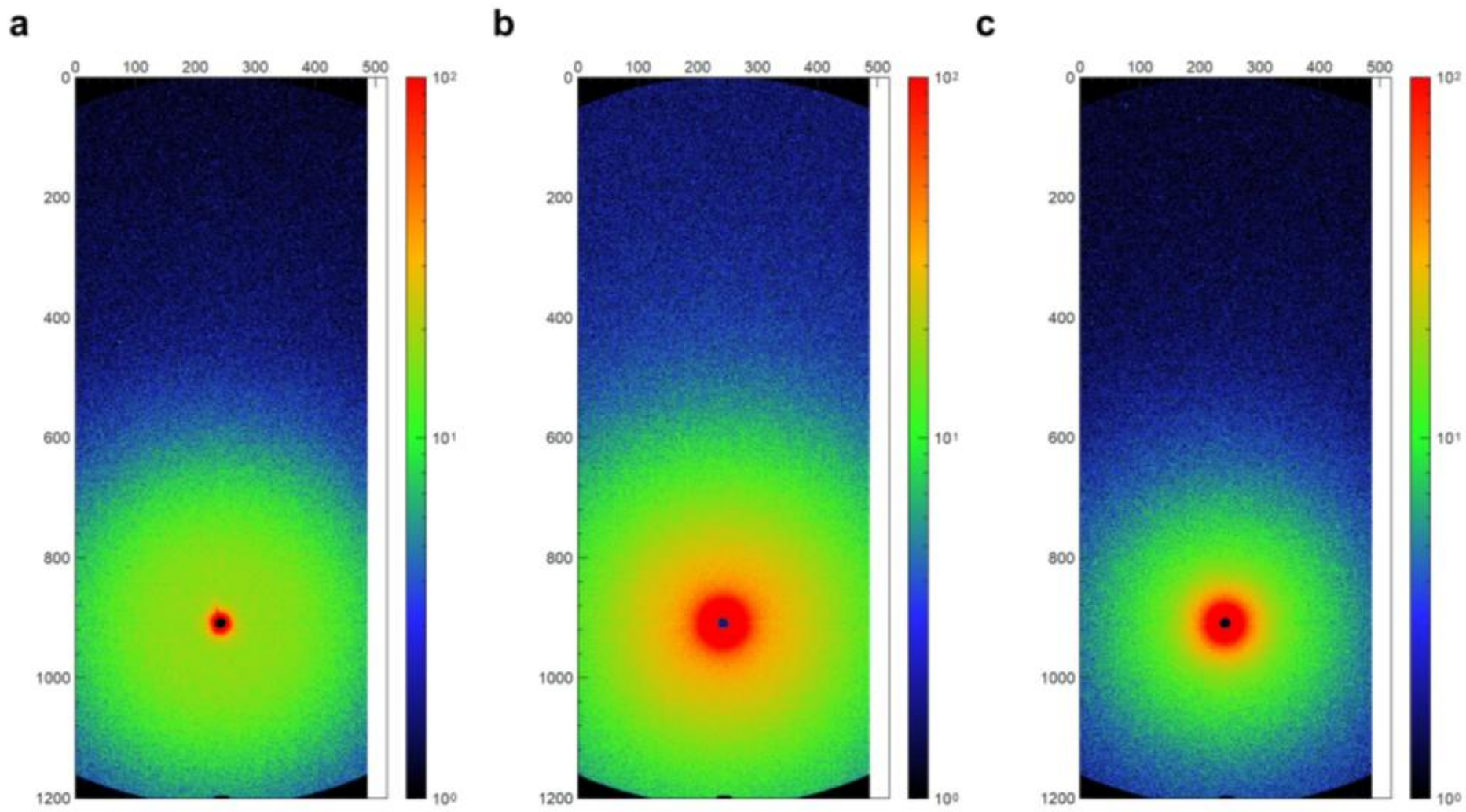

Supplementary Fig. 26. 2D SAXS images of (a) HC-0-900, (b) HC-10-900, and (c) HC-100-900.

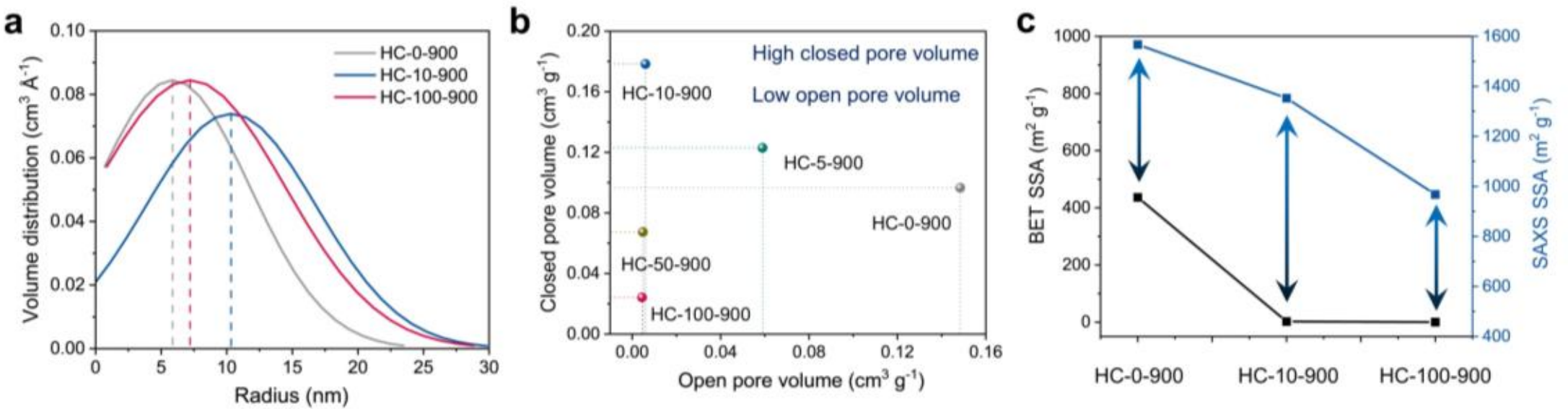

Supplementary Fig. 27. (a) The volume distribution of closed pores in HC-0-9000, HC-10-900, and HC-100-900. (b) The open pore volume and closed pore volume of HC-0-900, HC-5-900, HC-10-900, HC-50-900, and HC-100-900. (c) The BET SSA and SAXS SSA of HC-0-9000, HC-10-900, and HC-100-900.

Supplementary Note 12: The volume of closed pores can be calculated by the following formula:

$$V_{closed\ pores} = 1/\rho_{true} - 1/2.26 \qquad (7)$$

Here, 2.26 g cm^{-3} is the true density of graphite without pores[19].

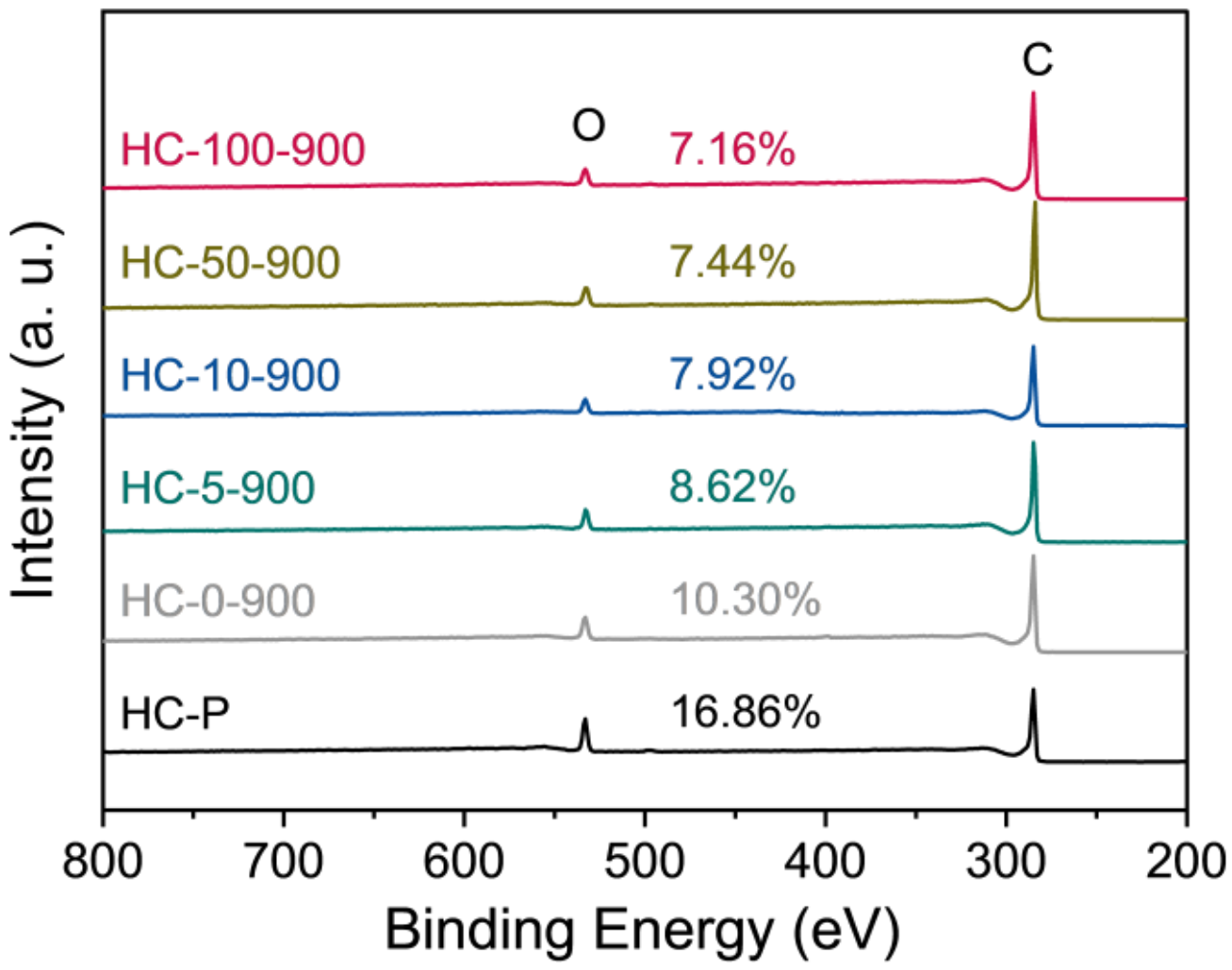

Supplementary Fig. 28. The XPS of HC-P, HC-0-900, HC-5-900, HC-10-900, HC-50-900, and HC-100-900.

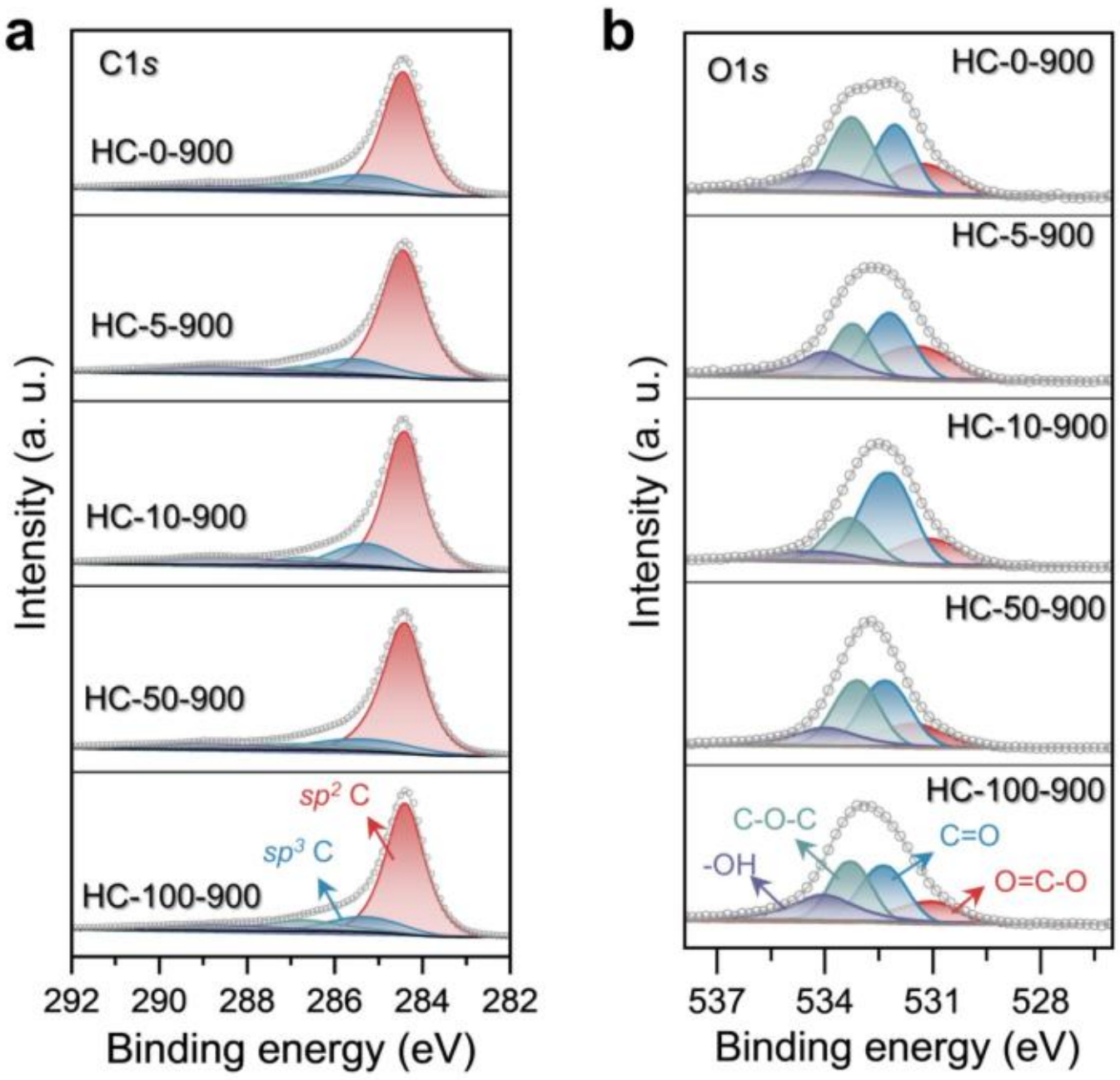

Supplementary Fig. 29. High-resolution (a) C 1*s* and (b) O 1*s* XPS spectra of HC-0-900, HC-5-900, HC-10-900, HC-50-900, and HC-100-900.

Supplementary Note 13: The C 1*s* spectra are fitted to four peaks with binding energies of 284.5 eV, 285.2 eV, 286.5 eV, and 288.8 eV, corresponding to sp^2 C, sp^3 C, C-O, and O=C–O, respectively[20]. The O 1*s* spectra are fitted to four peaks with binding energies of 534.35, 533.40, 532.02, and 531.14 eV, corresponding to –OH, C–O–C, C=O, and O=C–O, respectively.

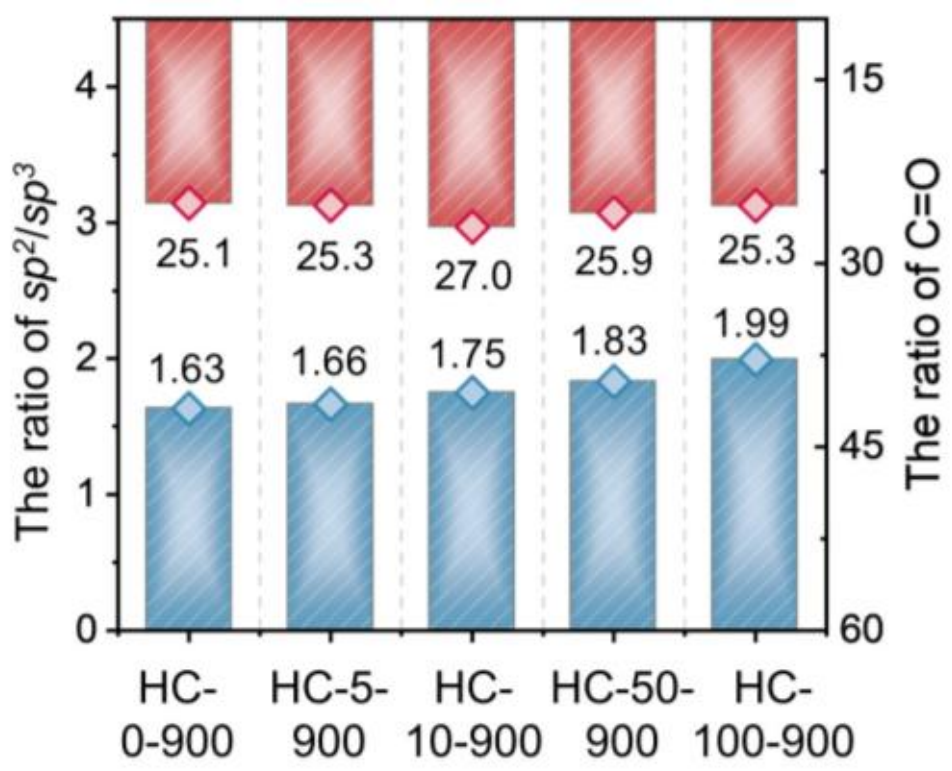

Supplementary Fig. 30. The sp^2/sp^3 ratio and C=O ratio of HC-0-900, HC-5-900, HC-10-900, HC-50-900, and HC-100-900.

Supplementary Note 14: Compared to LTC, the thermal-force coupling effect causes the oxygen content in the HC to decrease as pressure increases. And the C=O bond content first increases and then decreases as pressure increases, while the sp^2/sp^3 ratio continues to rise. This indicates that low-pressure assistance at the thermal field promotes C=O bonds cross-linking and the ordered stacking of carbon layers, inducing the formation of closed pores. However, under high-pressure assistance at the thermal field, the C=O bonds first cross-link and eventually break, as the carbon layer continues to develop toward a highly graphitized state, resulting in the elimination of closed-pores. HC-10-900 has a high content of C=O bonds (27.0%) and a moderate carbon layer structure, which promotes the formation of closed-pores. Moreover, the curvature of the carbon layer induced by the C=O bond facilitates the rapid Na^+ diffusion into the closed pores.

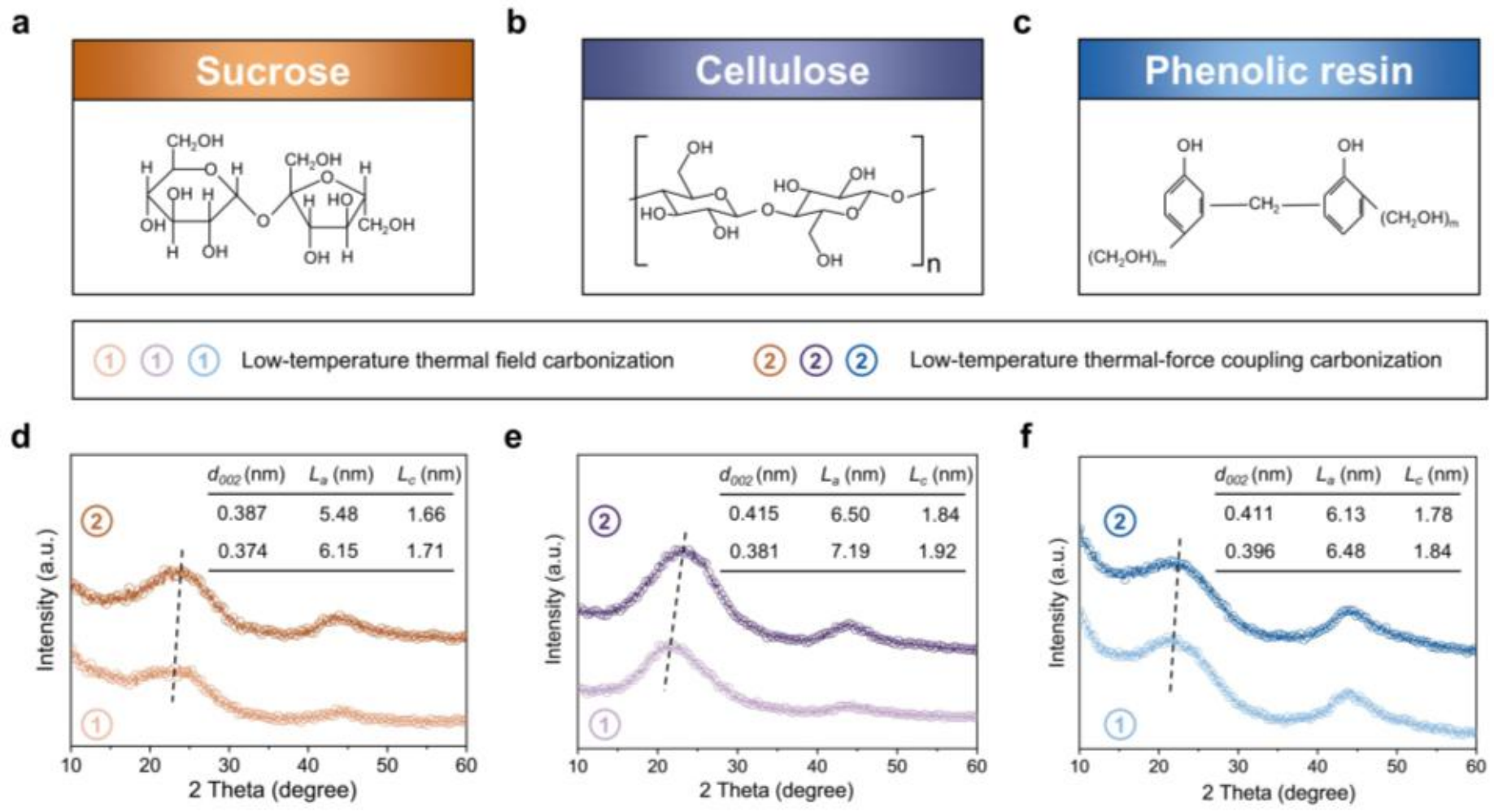

Supplementary Fig. 31. XRD patterns of (a, d) sucrose, (b, e) cellulose, and (c, f) phenolic resin derived carbon under LTC and LTFC.

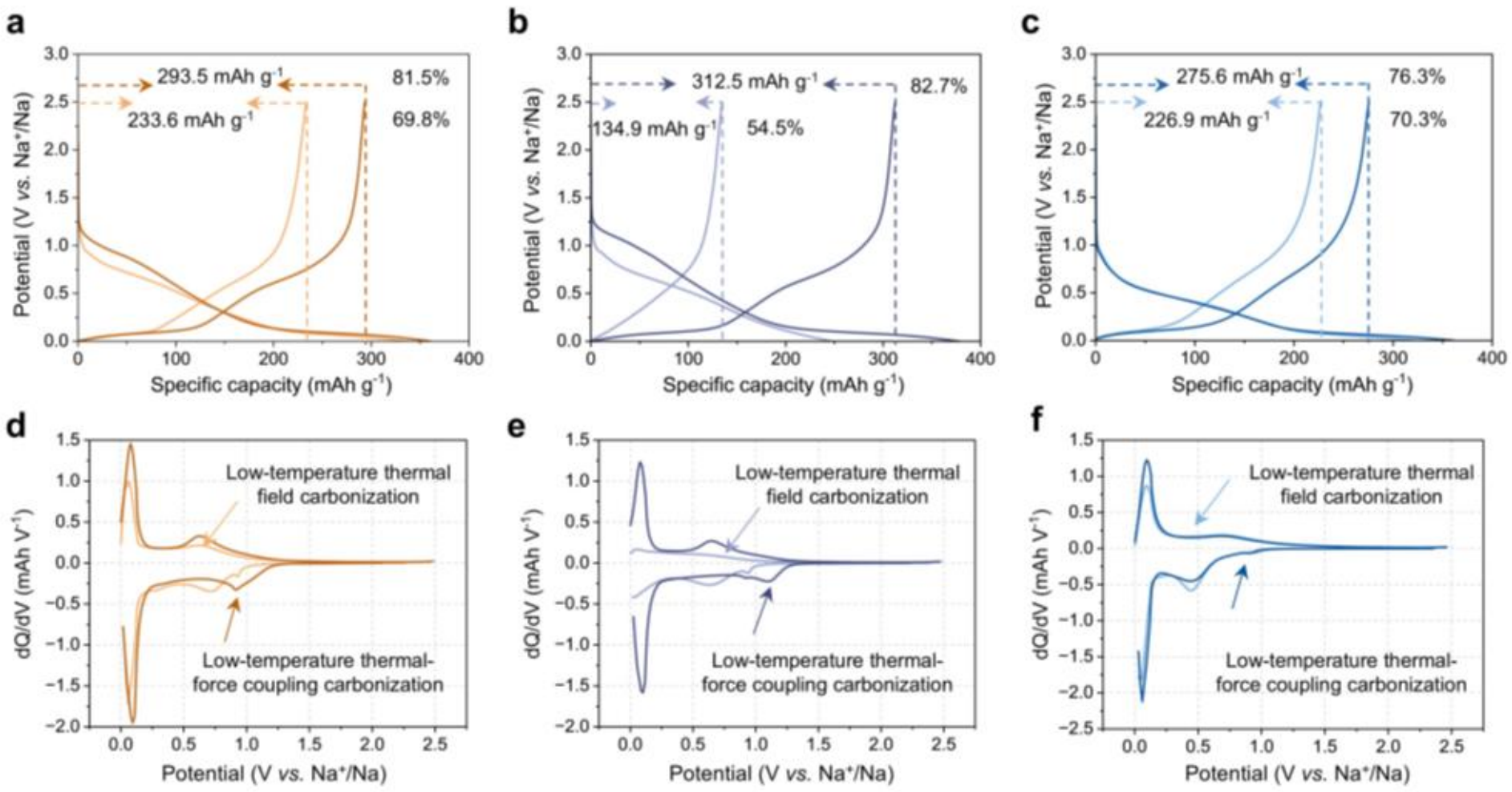

Supplementary Fig. 32. Initial charging and discharging curves and dQ/dV curves of (a, d) sucrose, (b, e) cellulose, and (c, f) phenolic resin derived carbon under LTC and LTFC at 28±0.3 °C.

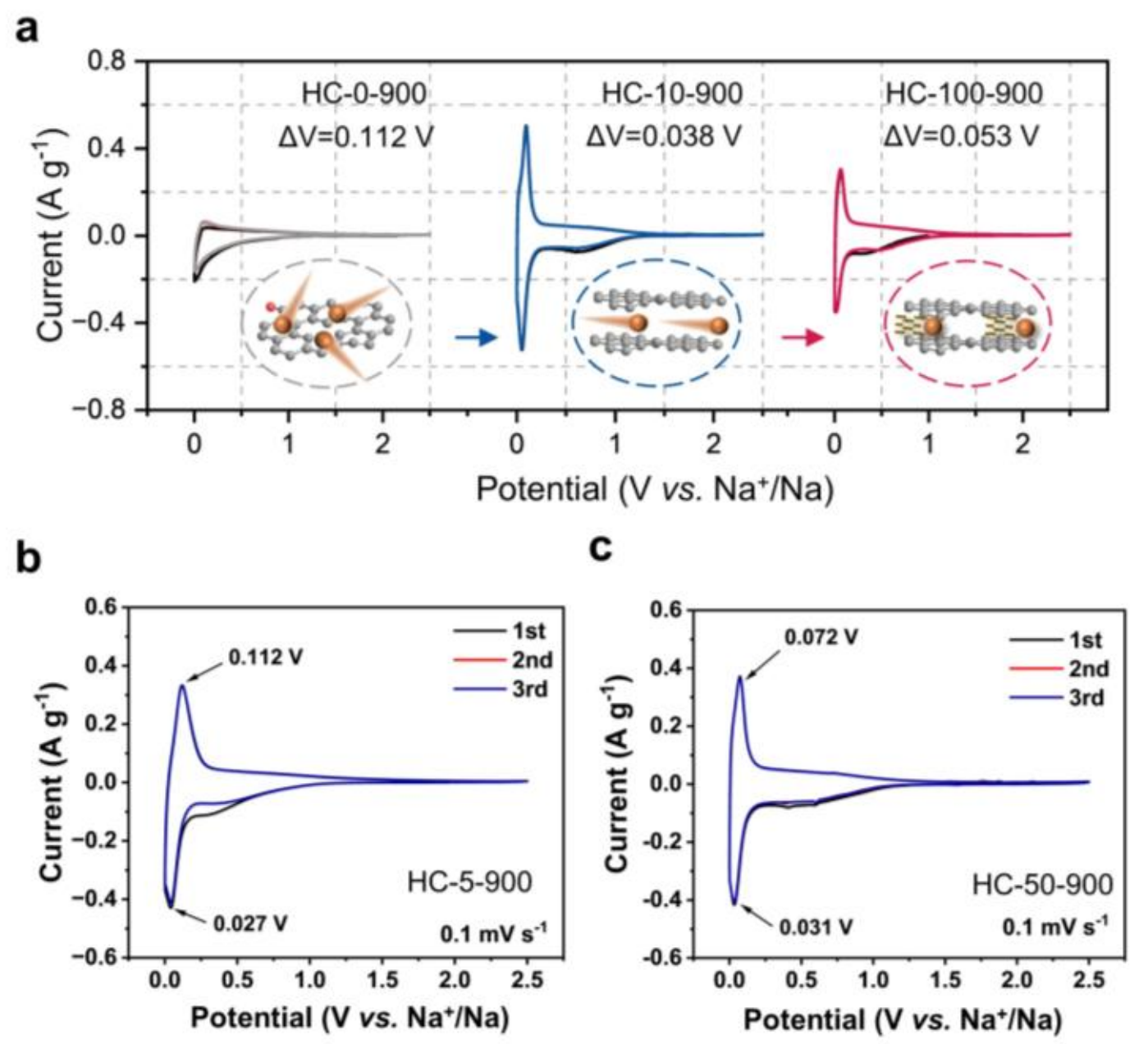

Supplementary Fig. 33. (a-c) CV curves at 0.1 mV s^{-1} of HC-0-900, HC-5-900, HC-10-900, HC-50-900, and HC-100-900 at 28±0.3 ℃.

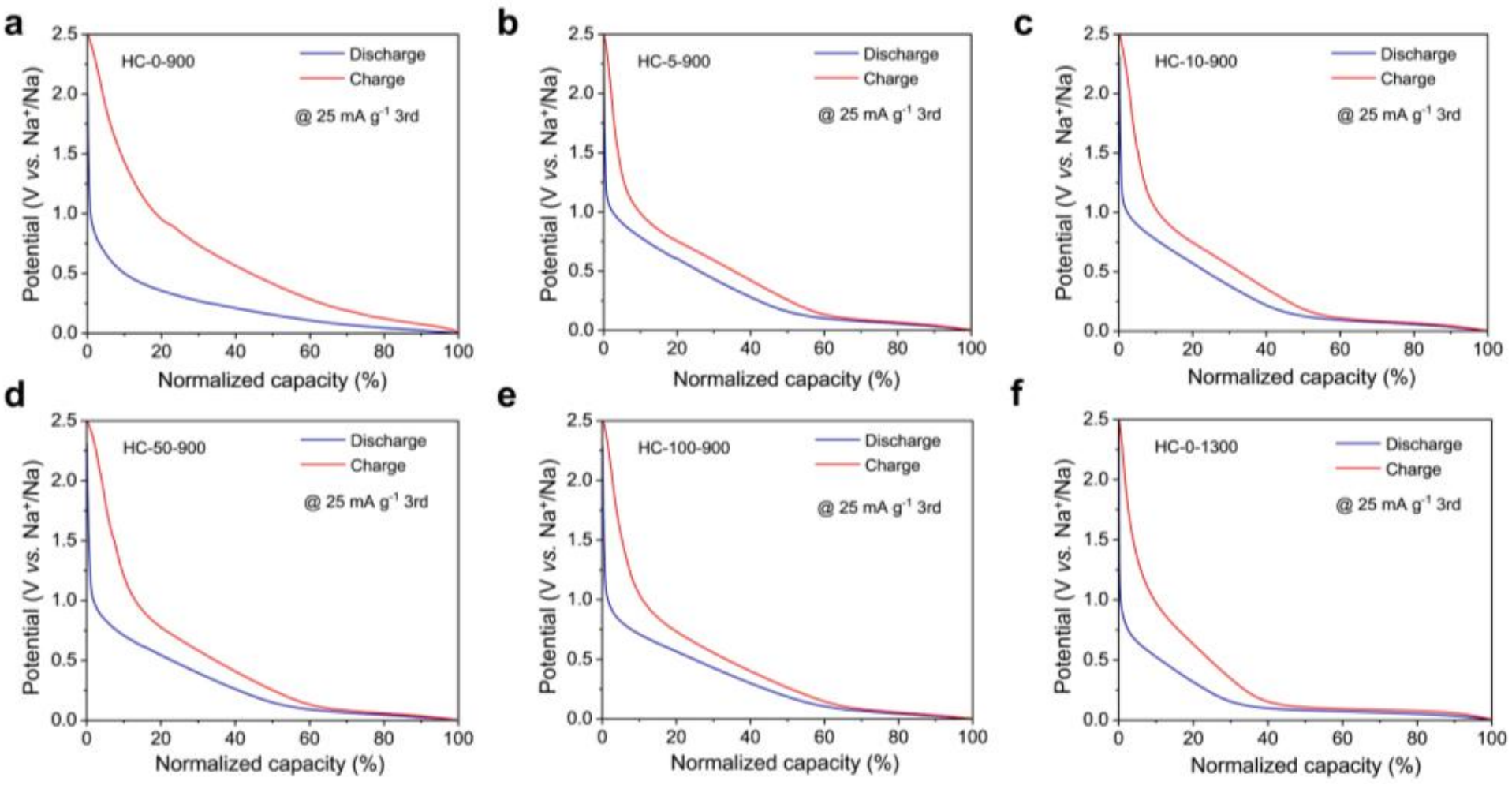

Supplementary Fig. 34. Capacity-potential normalized curves of (a) HC-0-900, (b) HC-5-900, (c) HC-10-900, (d) HC-50-900, (e) HC-100-900, and (f) HC-0-1300 at 28±0.3 ℃.

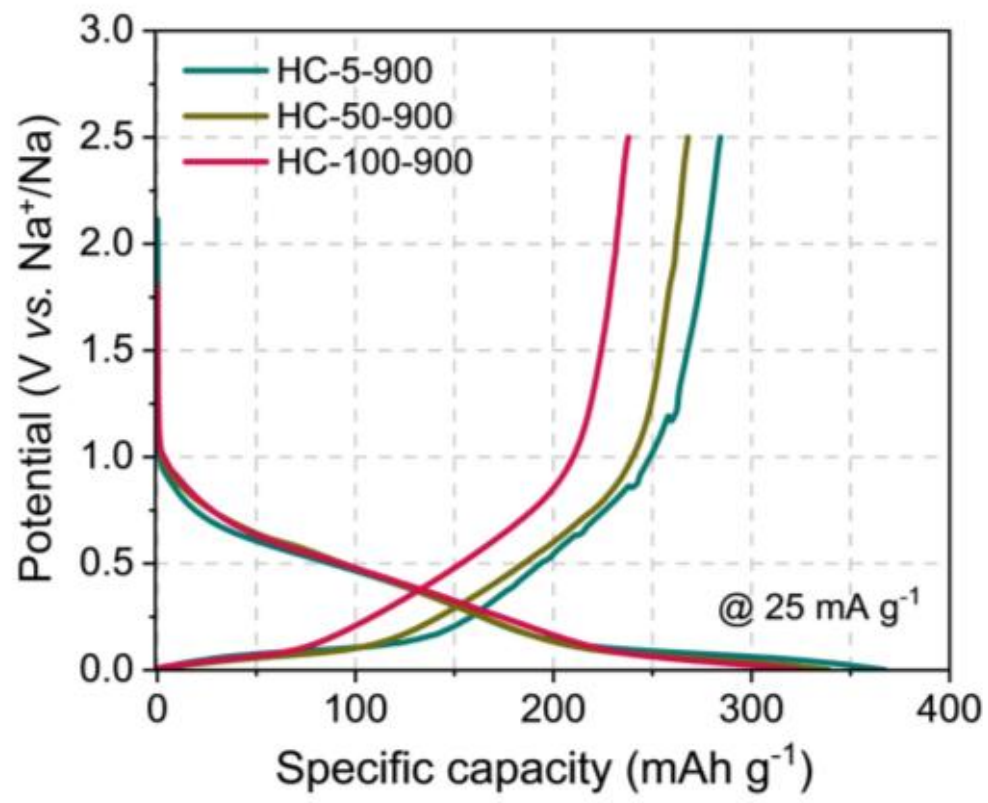

Supplementary Fig. 35. The initial discharge/charge capacities of HC-5-900, HC-50-900, and HC-100-900 at 28±0.3 ℃.

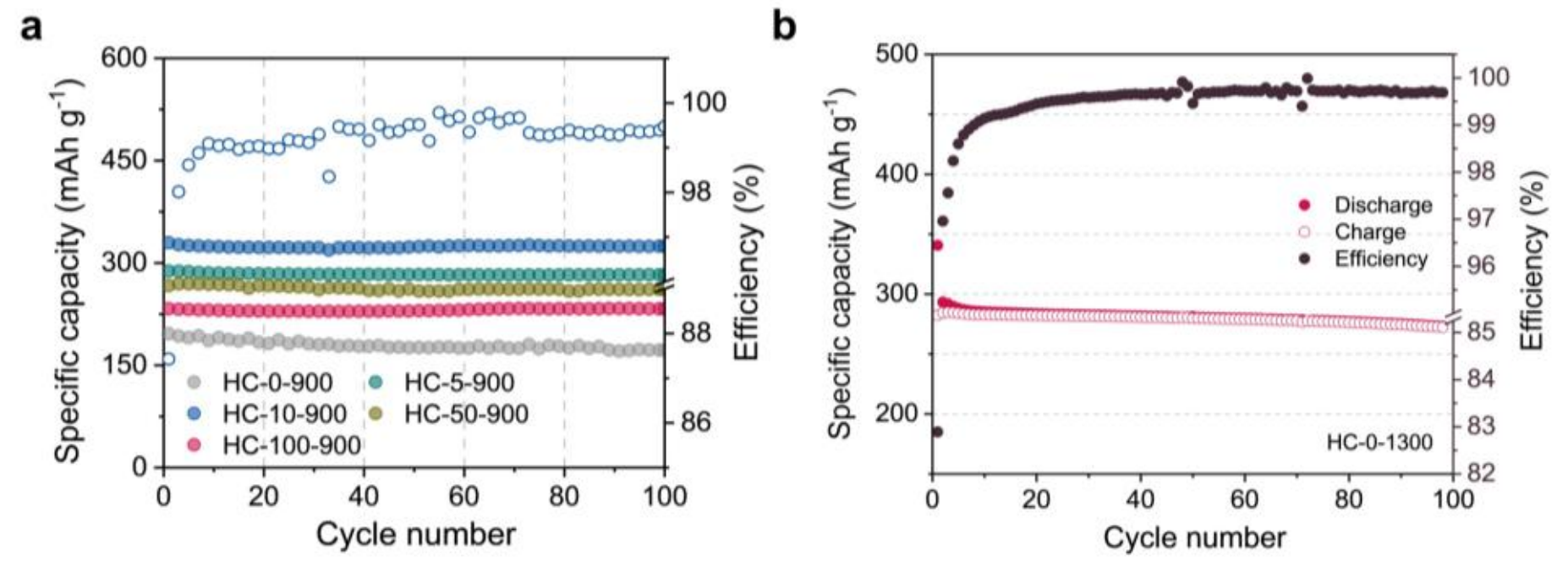

Supplementary Fig. 36. (a, b) Long-cycle performance of HC-0-900, HC-5-900, HC-10-900, HC-50-900, HC-100-900, and HC-0-1300 in a button-type half-cell at 25 mA g^{-1} (Applied temperature: 28±0.3 ℃).

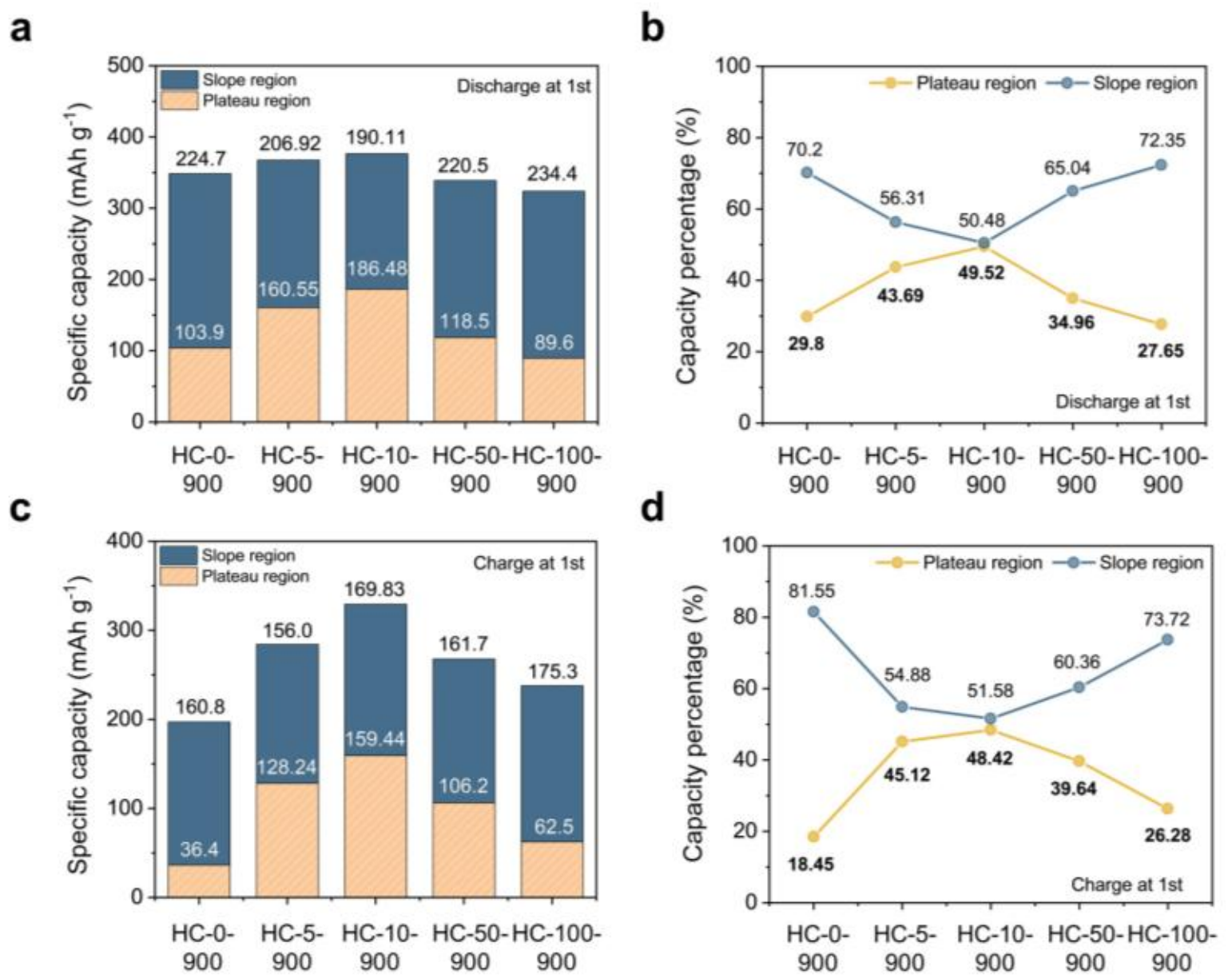

Supplementary Fig. 37. (a, c) Specific capacity and (b, d) percentage of slope region and plateau region at discharge and charge of HC-0-900, HC-5-900, HC-10-900, HC-50-900, and HC-100-900 at 28±0.3 ℃.

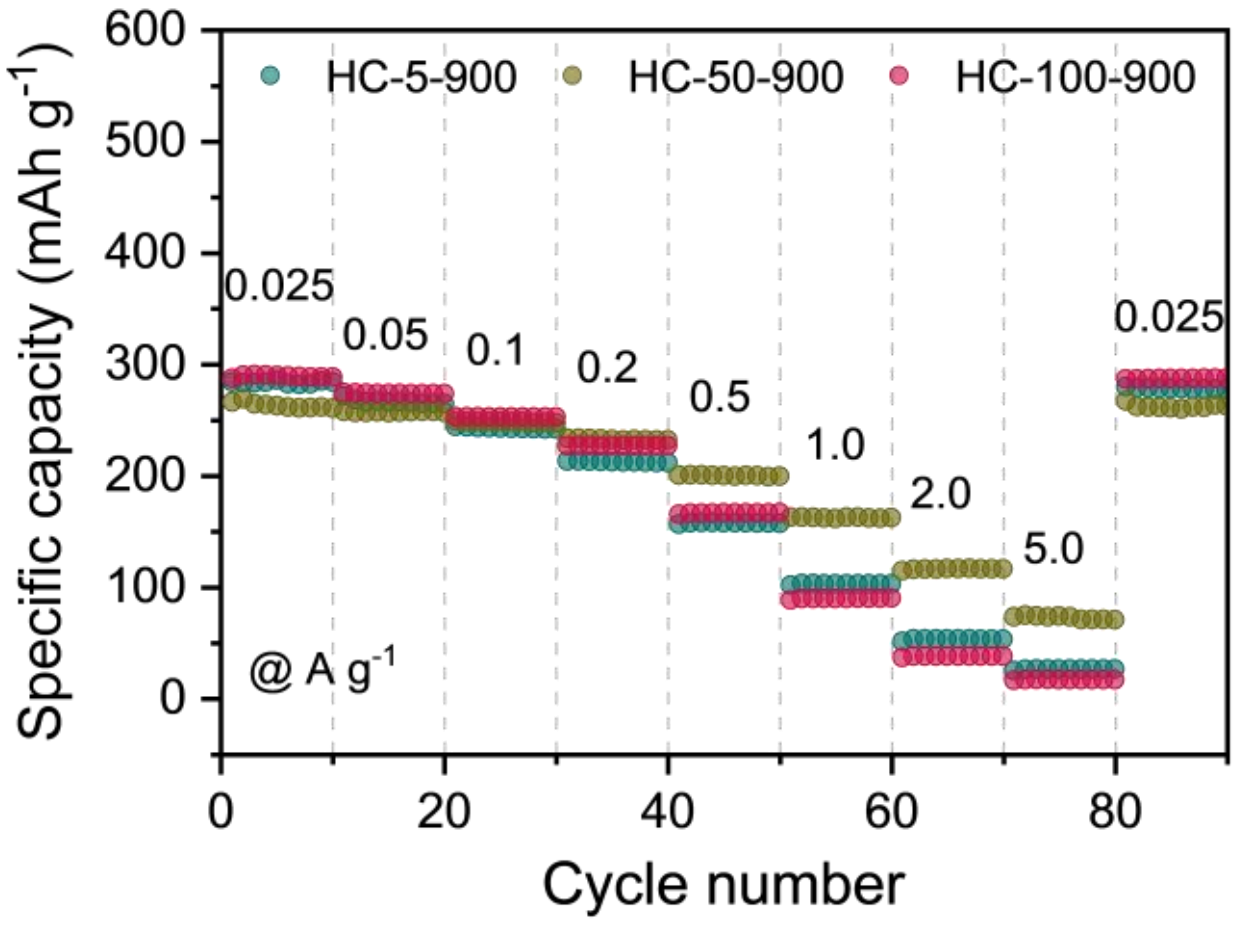

Supplementary Fig. 38. Rate performance of HC-5-900, HC-50-900, and HC-100-900 at 28±0.3 °C.

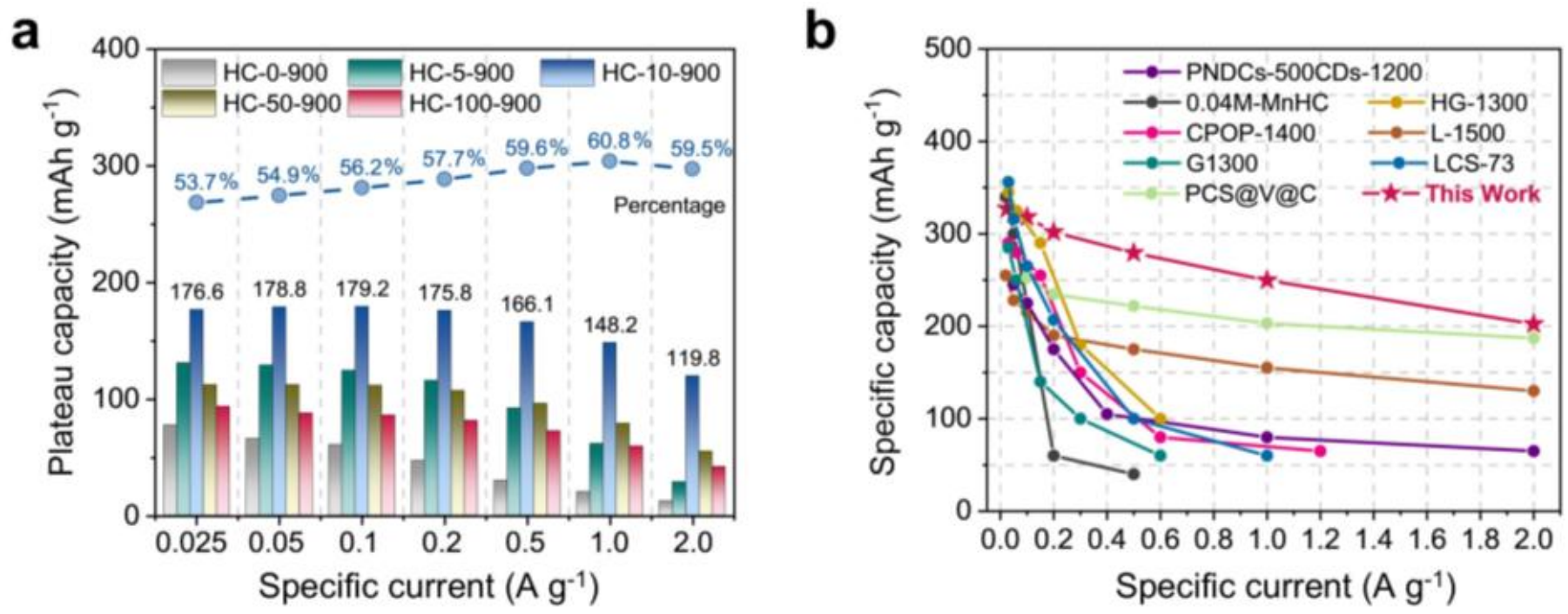

Supplementary Fig. 39. (a) Plateau capacity and percentage at different specific currents at 28±0.3 °C. (b) Comparison of the rate capacity of HC-10-900 and other reported carbon materials[20-27].

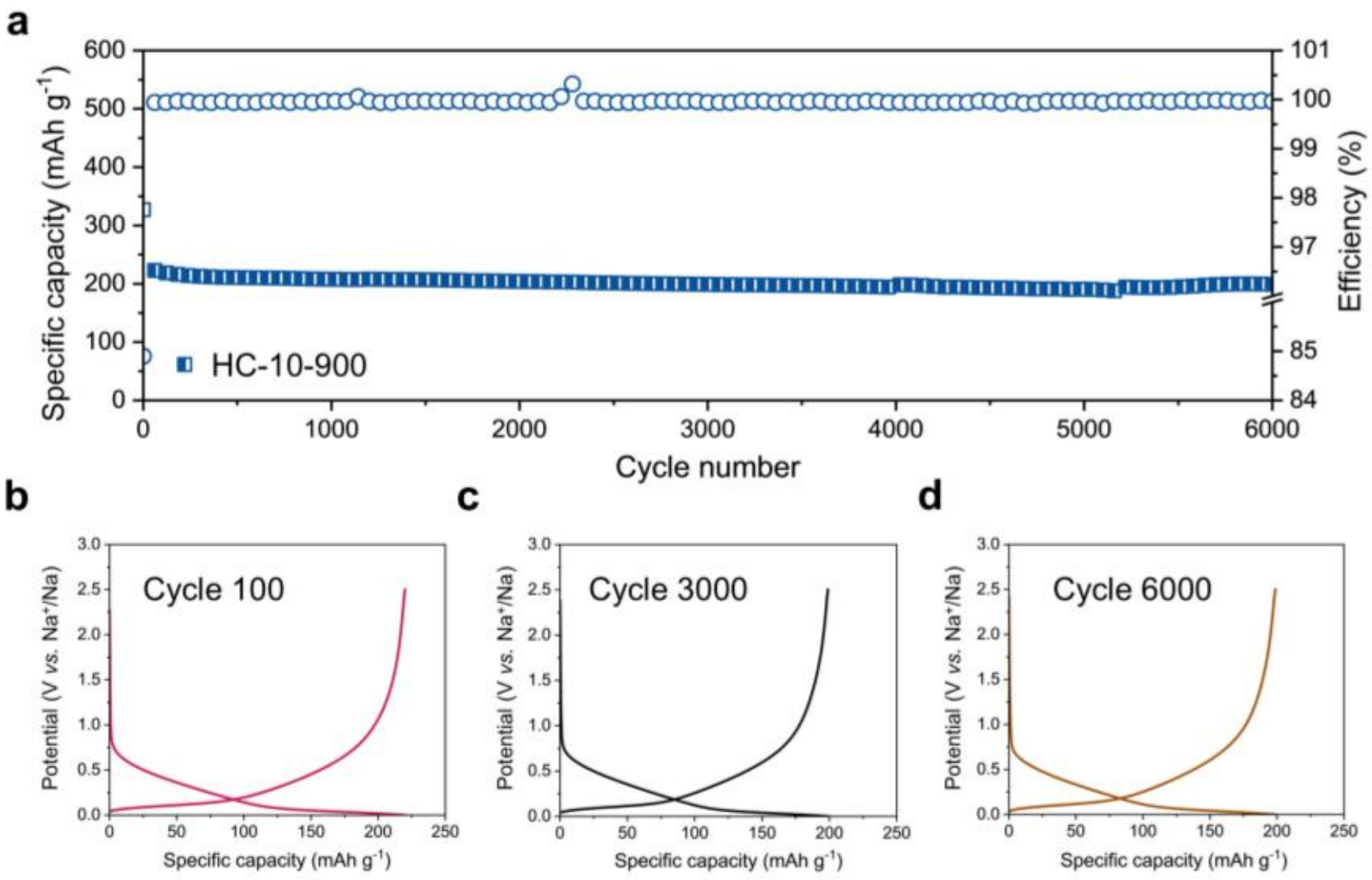

Supplementary Fig. 40. (a) Long-cycle performance of HC-10-900 in a button-type half-cell at 2.0 A g^{-1} (Applied temperature: 28±0.3 °C). Charge-discharge curves of HC-10-900 at (b) 100th, (c) 3000th, and (d) 6000th at 2.0 A g^{-1}.

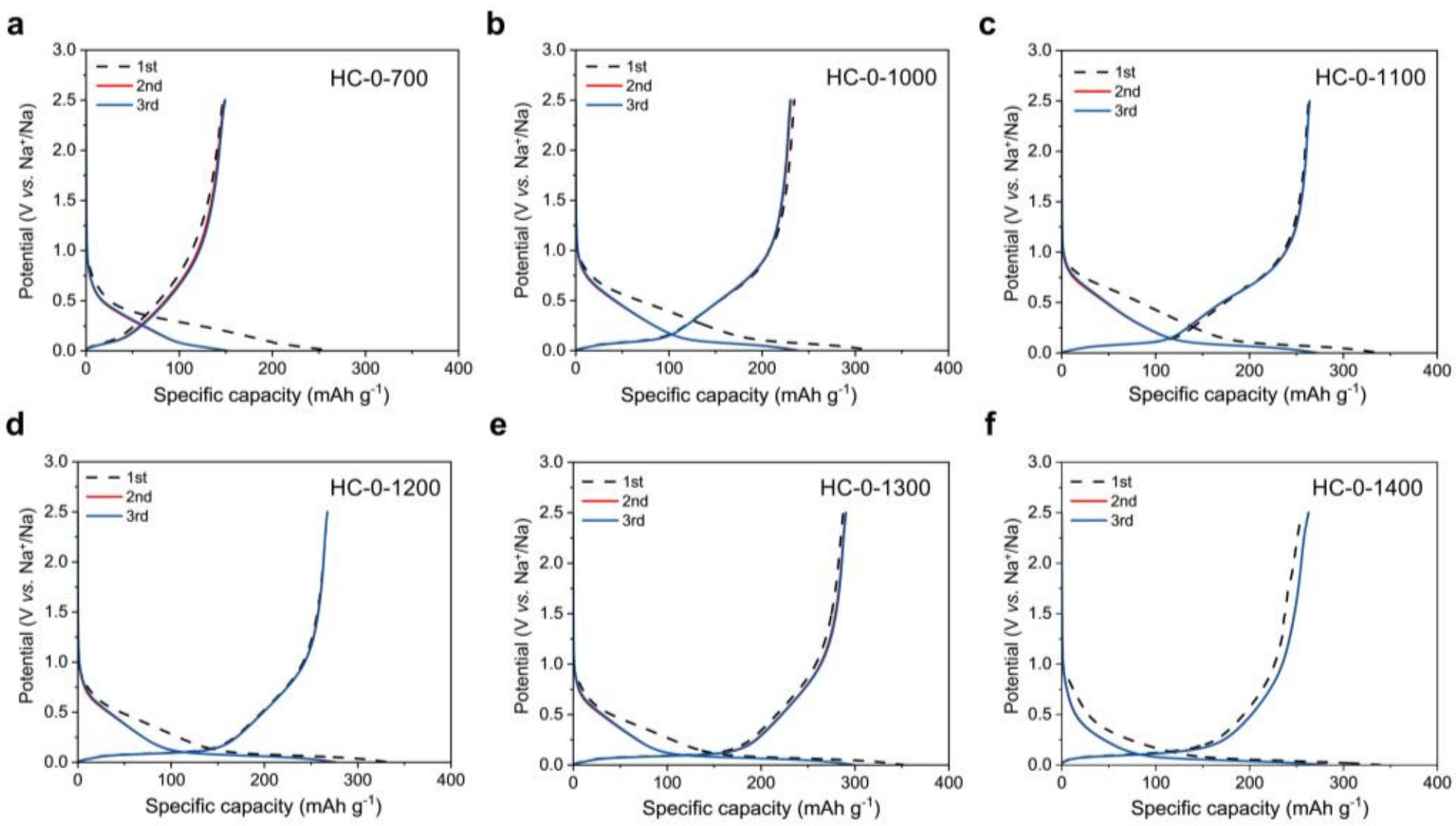

Supplementary Fig. 41. Charge-discharge curves of (a) HC-0-700, (b) HC-0-1000, (c) HC-0-1100, (d) HC-0-1200, (e) HC-0-1300, and (f) HC-0-1400 at 25 mA g^{-1} (Applied temperature: 28±0.3 °C).

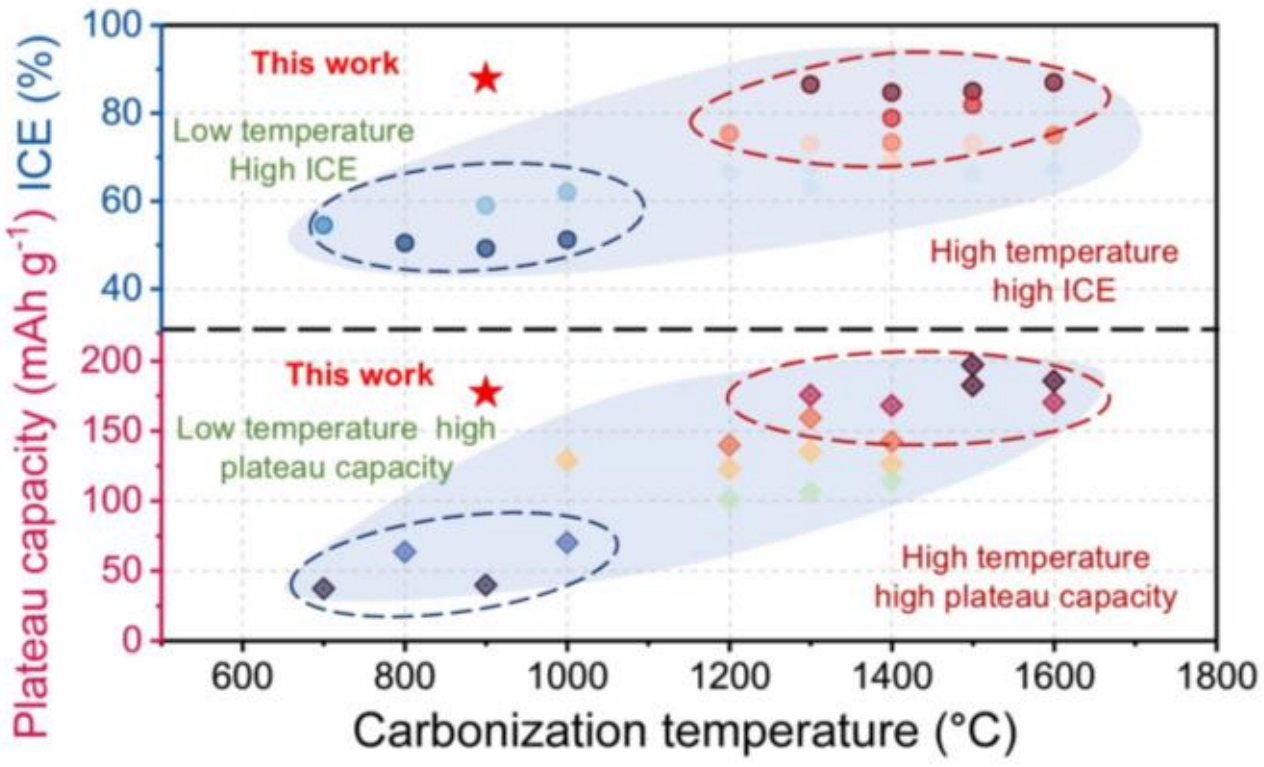

Supplementary Fig. 42. Comparison of ICE, plateau capacity, and carbonization temperature of HC-10-900 and other reported carbon materials[28-53].

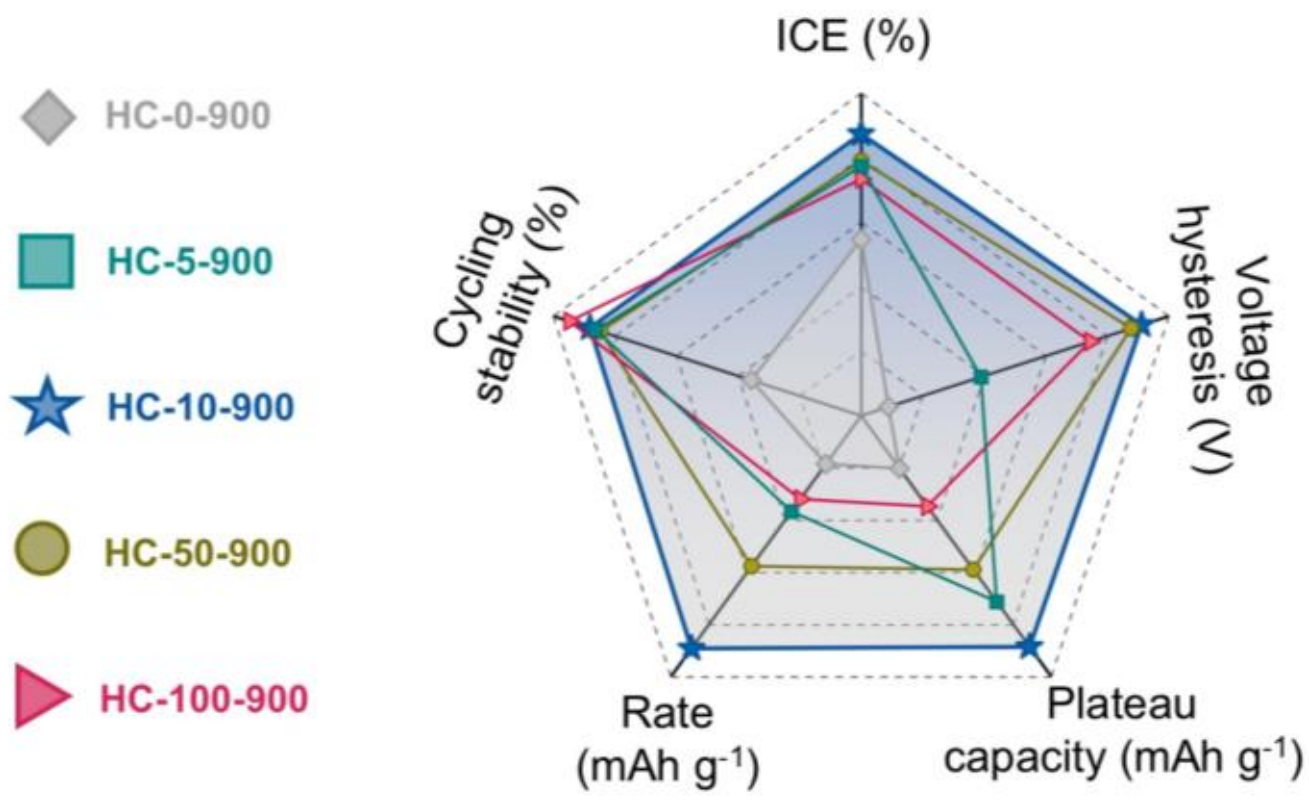

Supplementary Fig. 43. Comparison of electrochemical performance.

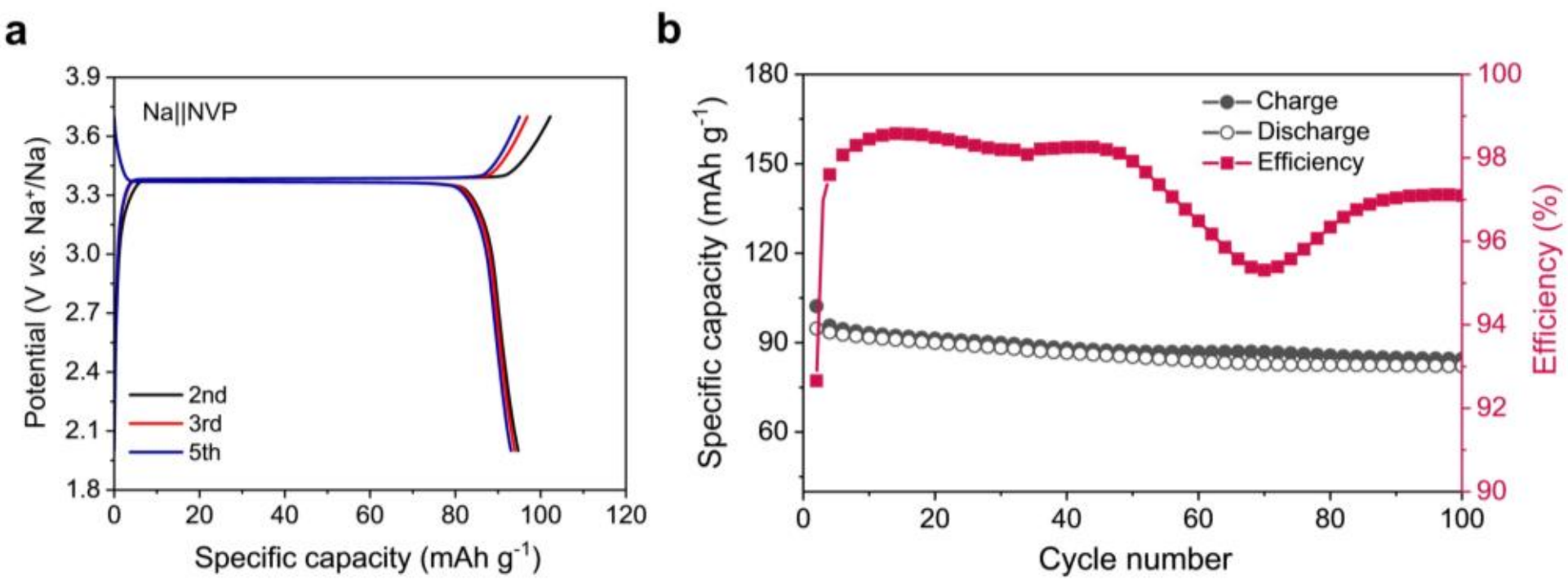

Supplementary Fig. 44. (a) Charge-discharge curves and (b) cycle performance at 25 mA g^{-1} of $Na_3V_2(PO_4)_3$ (NVP) positive electrodes in a button-type half-cell (Applied temperature: 28±0.3 ℃).

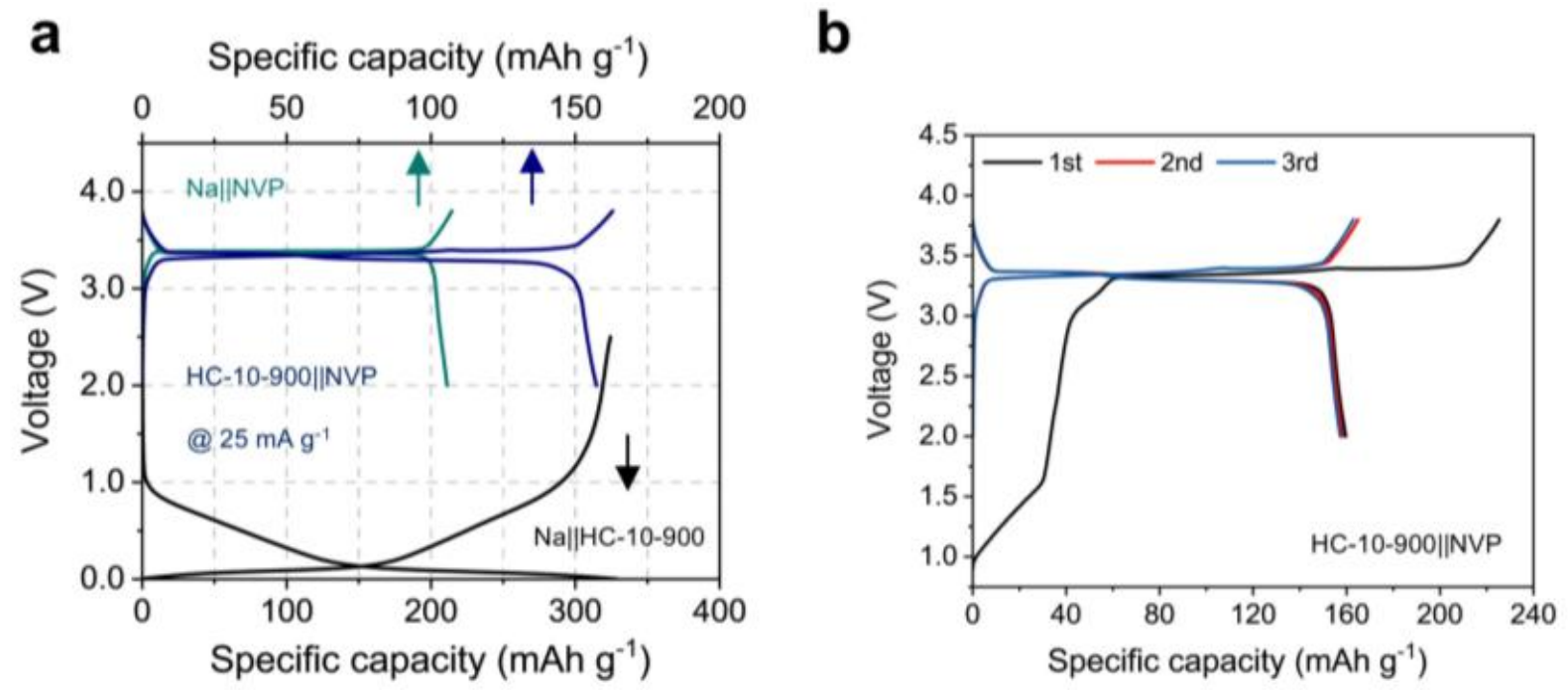

Supplementary Fig. 45. (a) Charge-discharge curves at 3rd of Na||NVP, Na||HC-10-900, and HC-10-900||NVP full cell. (b) Charge-discharge curves of HC-10-900||NVP full cell (Applied temperature: 28±0.3 ℃).

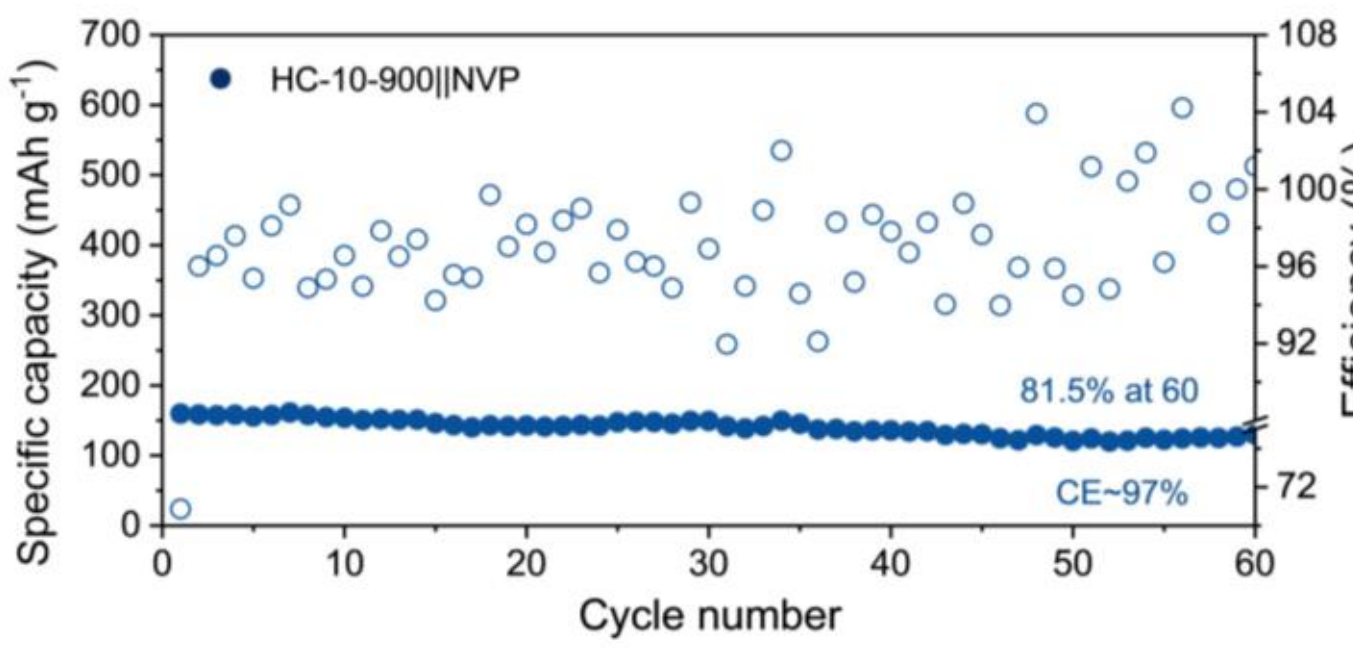

Supplementary Fig. 46. Cycle performance at 25 mA g^{-1} of HC-10-900||NVP button-type full cell (Applied temperature: 28±0.3 ℃).

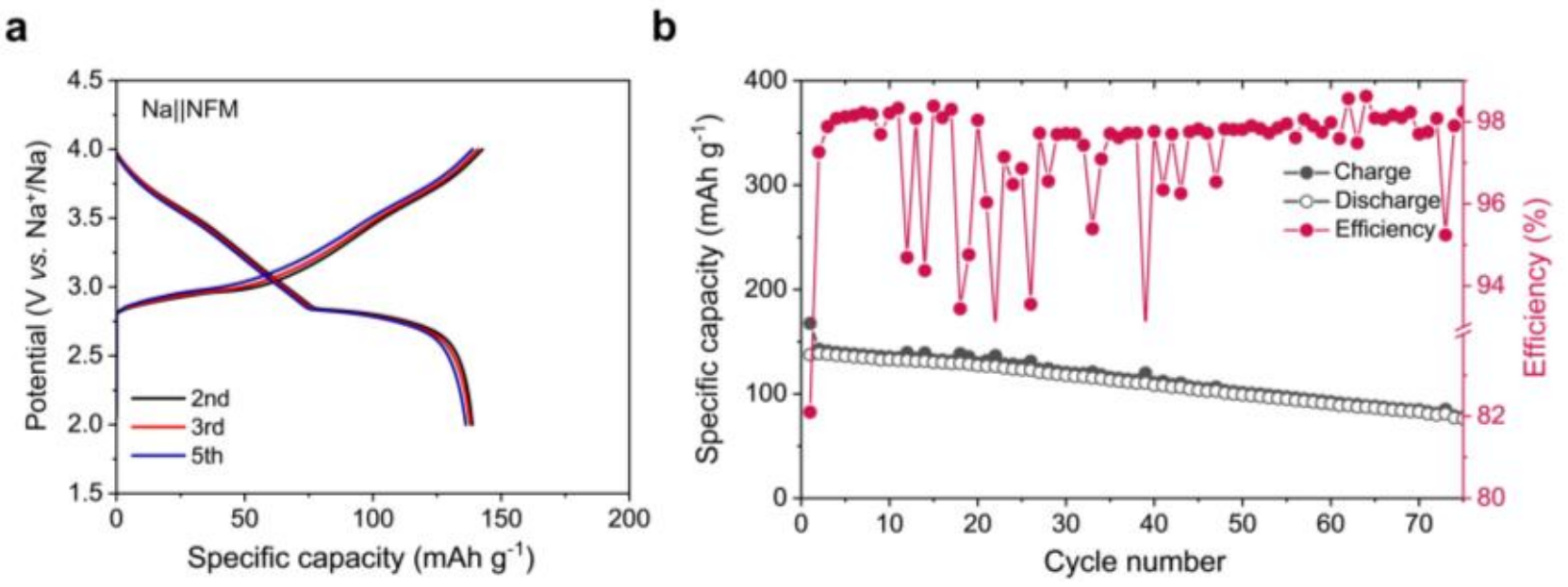

Supplementary Fig. 47. (a) Charge-discharge curves and (b) cycle performance at 25 mA g^{-1} of $NaNi_{1/3}Fe_{1/3}Mn_{1/3}O_2$ (NFM) positive electrodes in a button-type half-cell (Applied temperature: 28±0.3 ℃).

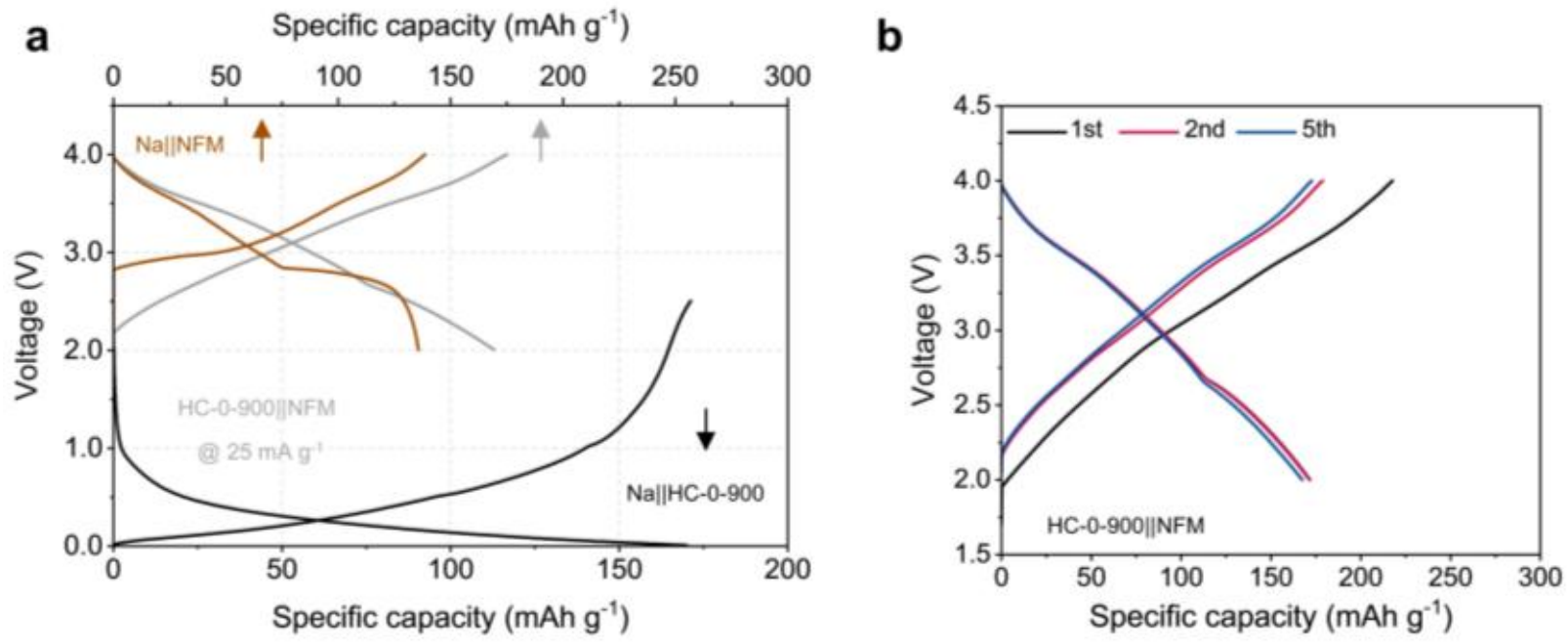

Supplementary Fig. 48. (a) Charge-discharge curves at 3rd of Na||NFM, Na||HC-0-900, and HC-0-900||NFM full cell. (b) Charge-discharge curves of HC-0-900||NFM full cell (Applied temperature: 28±0.3 ℃).

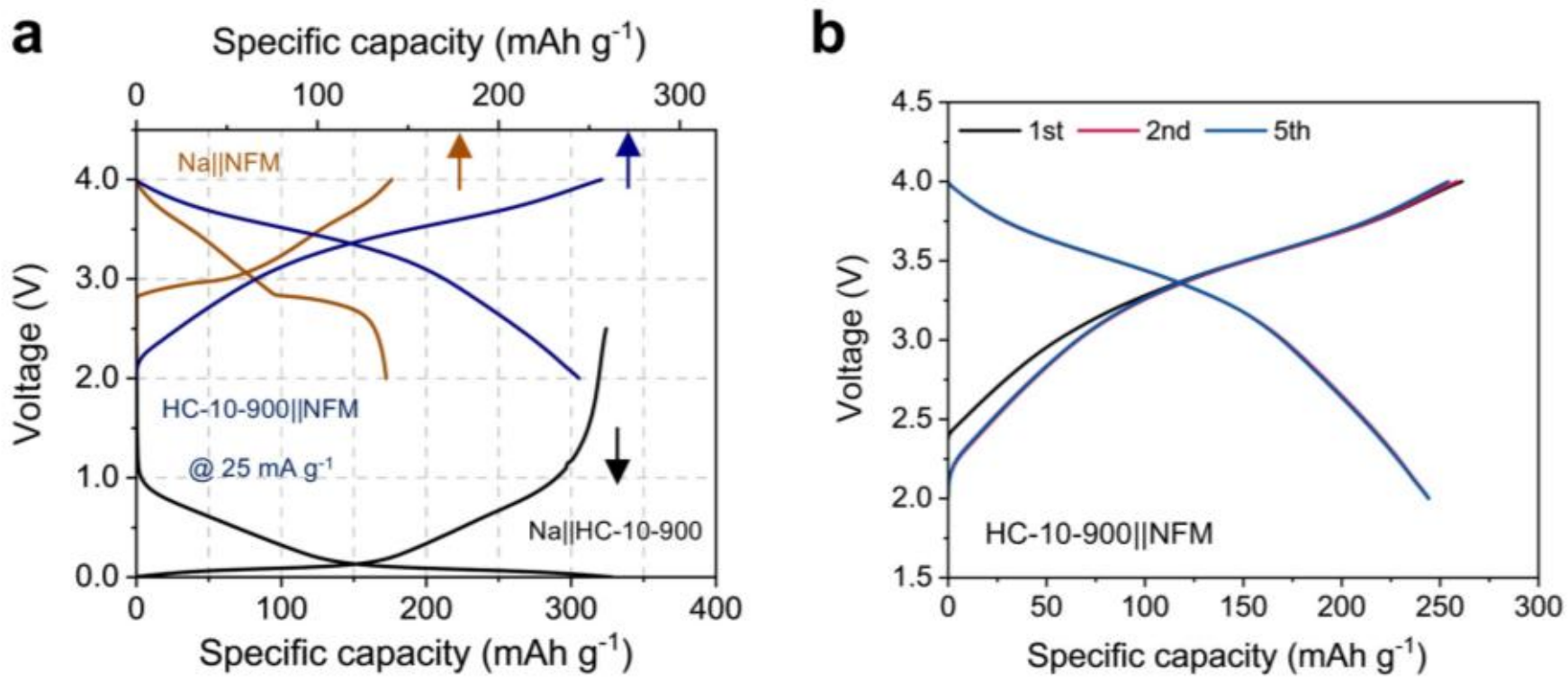

Supplementary Fig. 49. (a) Charge-discharge curves at 3rd of Na||NFM, Na||HC-10-900, and HC-10-900||NFM full cell. (b) Charge-discharge curves of HC-10-900||NFM full cell (Applied temperature: 28±0.3 ℃).

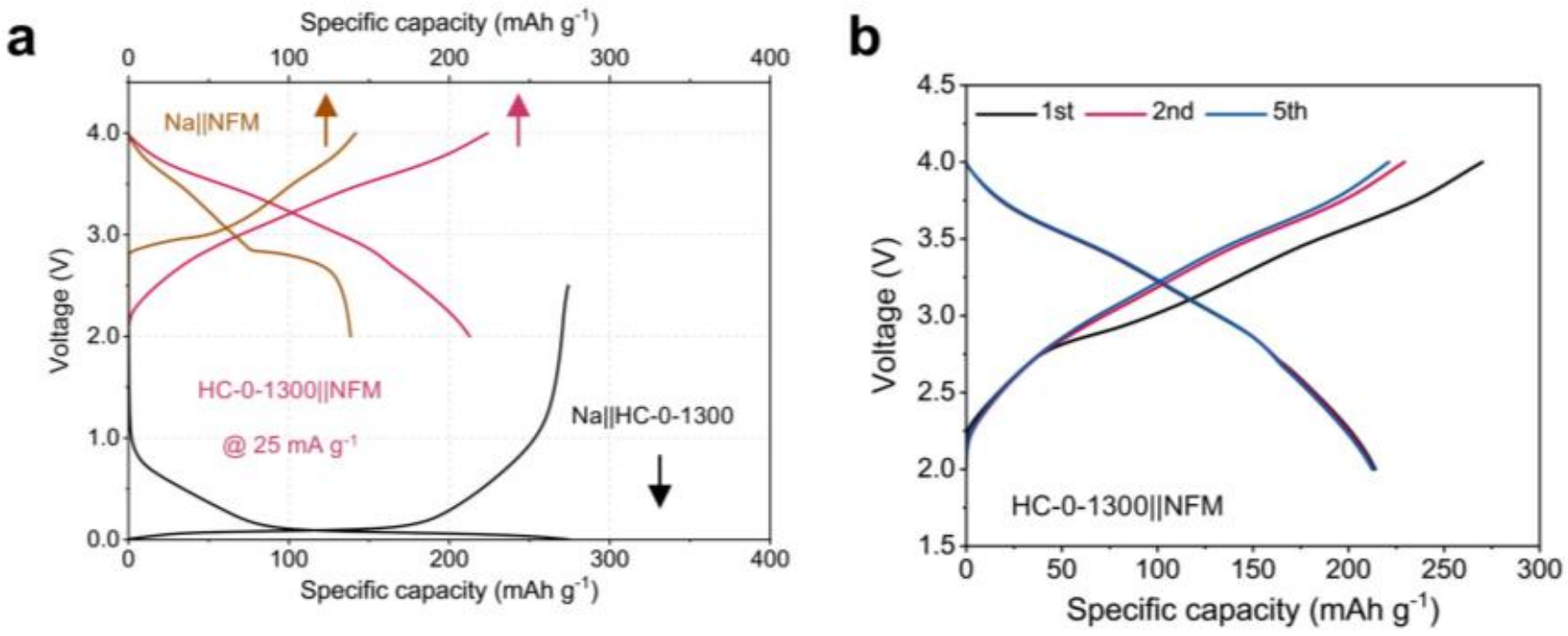

Supplementary Fig. 50. (a) Charge-discharge curves at 3rd of Na||NFM, Na||HC-0-1300, and HC-0-1300||NFM full cell. (b) Charge-discharge curves of HC-0-1300||NFM full cell (Applied temperature: 28±0.3 ℃).

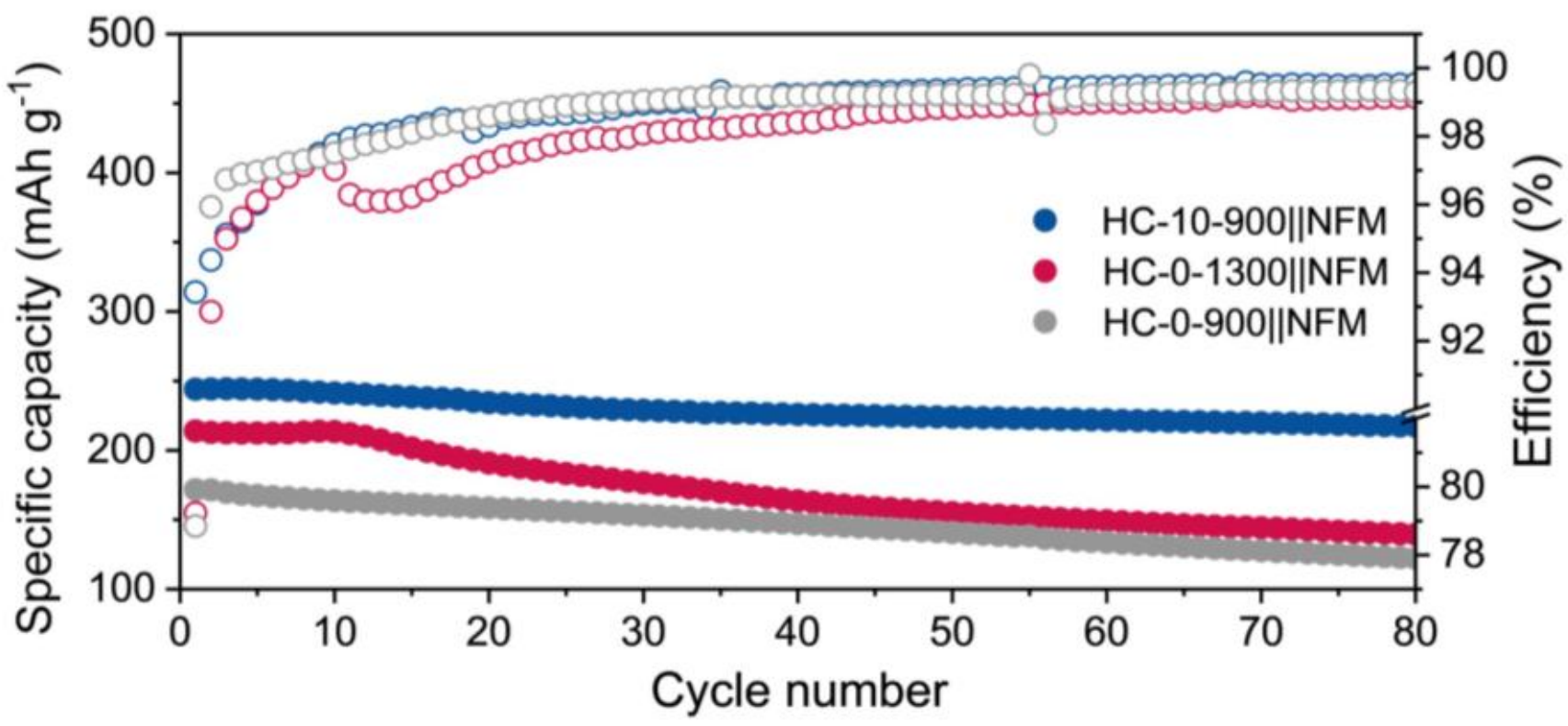

Supplementary Fig. 51. Cycle performance at 25 mA g^{-1} of HC-0-900||NFM, HC-10-900||NFM, and HC-0-1300||NFM button-type full cell (Applied temperature: 28±0.3 ℃).

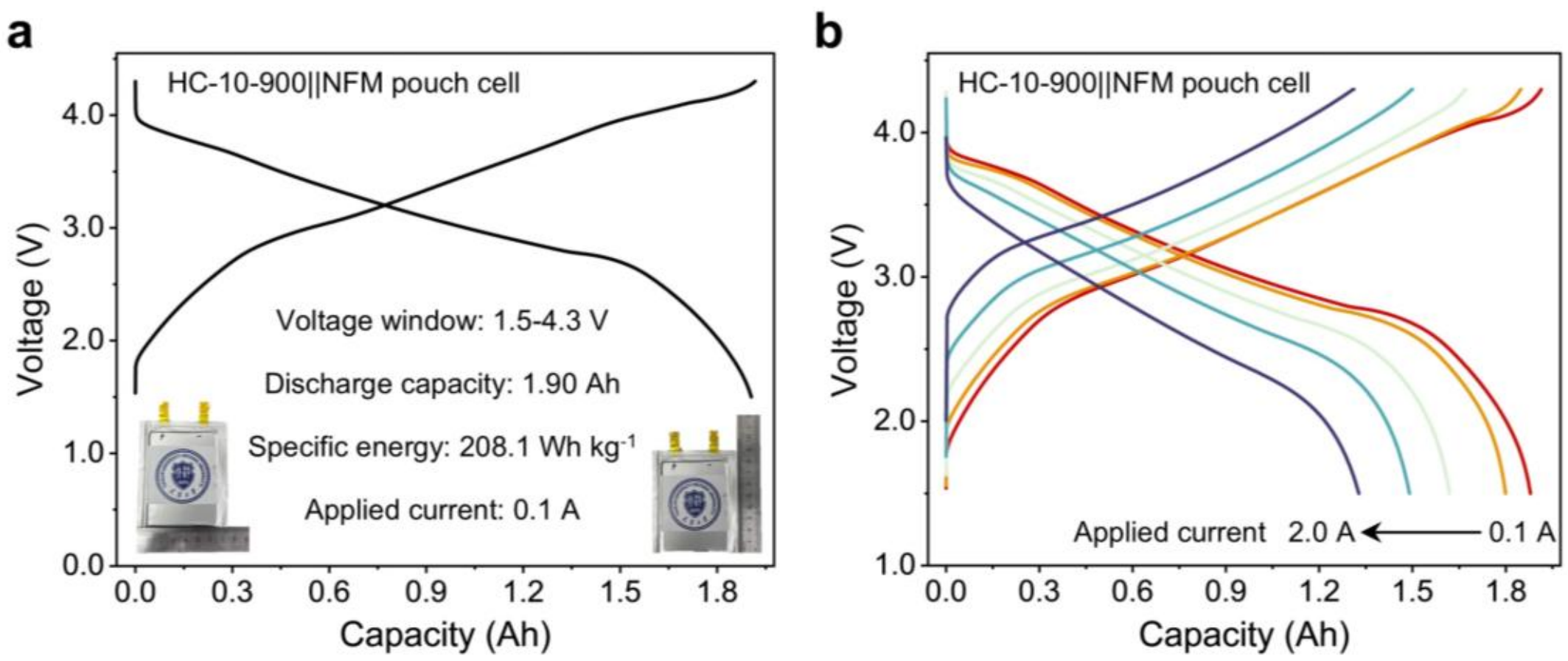

Supplementary Fig. 52. (a) Charging and discharging curves at 1.5-4.3 V of the sodium-ion pouch cell. (b) Rate performance at 1.5-4.3 V of the sodium-ion pouch cell (Applied temperature: 28±0.3 ℃).

Supplementary Note 15: An assembled sodium-ion pouch cell primarily consists of the negative electrode, the positive electrode, the aluminum current collector, the PP separator, the electrolyte, the aluminum tab, the nickel tab, and the aluminum-plastic film with pattern, with masses of 7.25 g, 12.15 g, 2.58 g, 0.74 g, 3.00 g, 0.06 g, 0.21 g, and 1.86 g, respectively. The total weight of the sodium-ion pouch cell is 27.85 g. On the negative electrode side, the active material mass loading is 7.0 mg cm^{-2}, the active material mass fraction is 90%, and the electrode dimensions are 77 mm × 56 mm. On the positive electrode side, the active material mass loading is 12.5 mg cm^{-2}, the active material mass fraction is 96%, and the electrode dimensions are 75 mm × 54 mm.

Based on the weight of the pouch cell, taking all components (electrode materials, binder, conductive agents, separator, electrolyte, tabs, current collectors, and Al plastic casing, etc) into account. The pouch cell provides a discharge capacity of 1.47 Ah at 1.5-3.95 V and a discharge specific energy of 166.4 Wh kg^{-1}. The pouch cell provides a discharge capacity of 1.90 Ah at 1.5-4.3 V and a discharge specific energy of 208.1 Wh kg^{-1}.

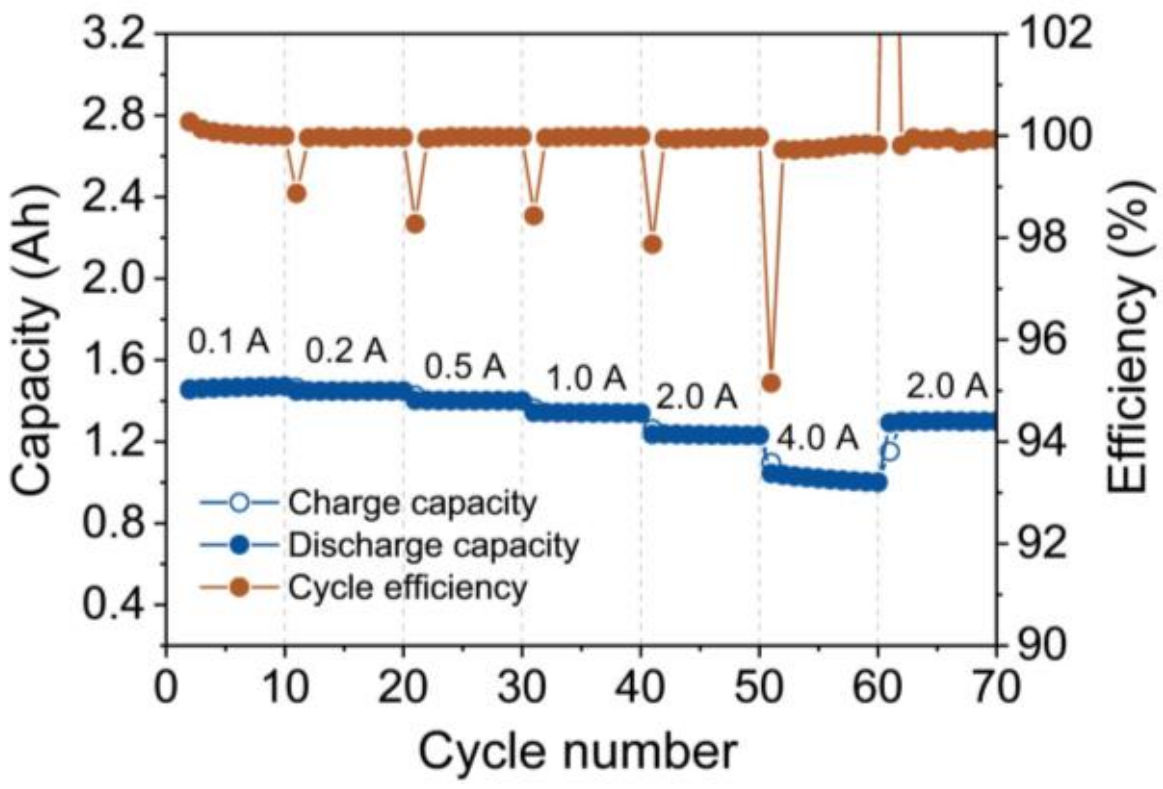

Supplementary Fig. 53. Rate performance at 1.5-3.95 V of the sodium-ion pouch cell (Applied temperature: 28±0.3 ℃).

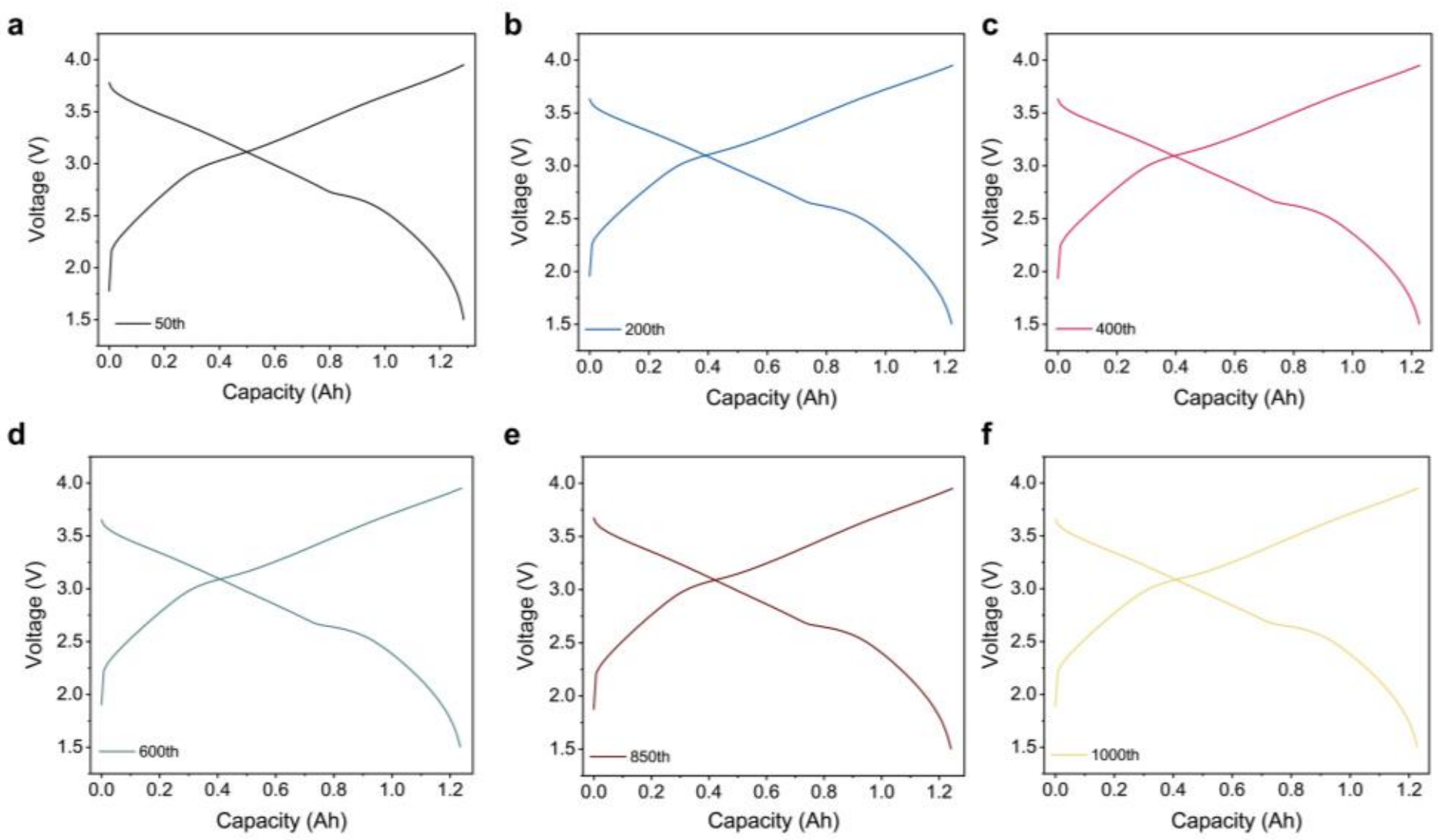

Supplementary Fig. 54. Charging and discharging curves at 1.5-3.95 V of a sodium-ion pouch cell at (a) 50th, (b) 200th, (c) 400th, (d) 600th, (e) 850th, and (f) 1000th in 1.0 A (Applied temperature: 28±0.3 ℃).

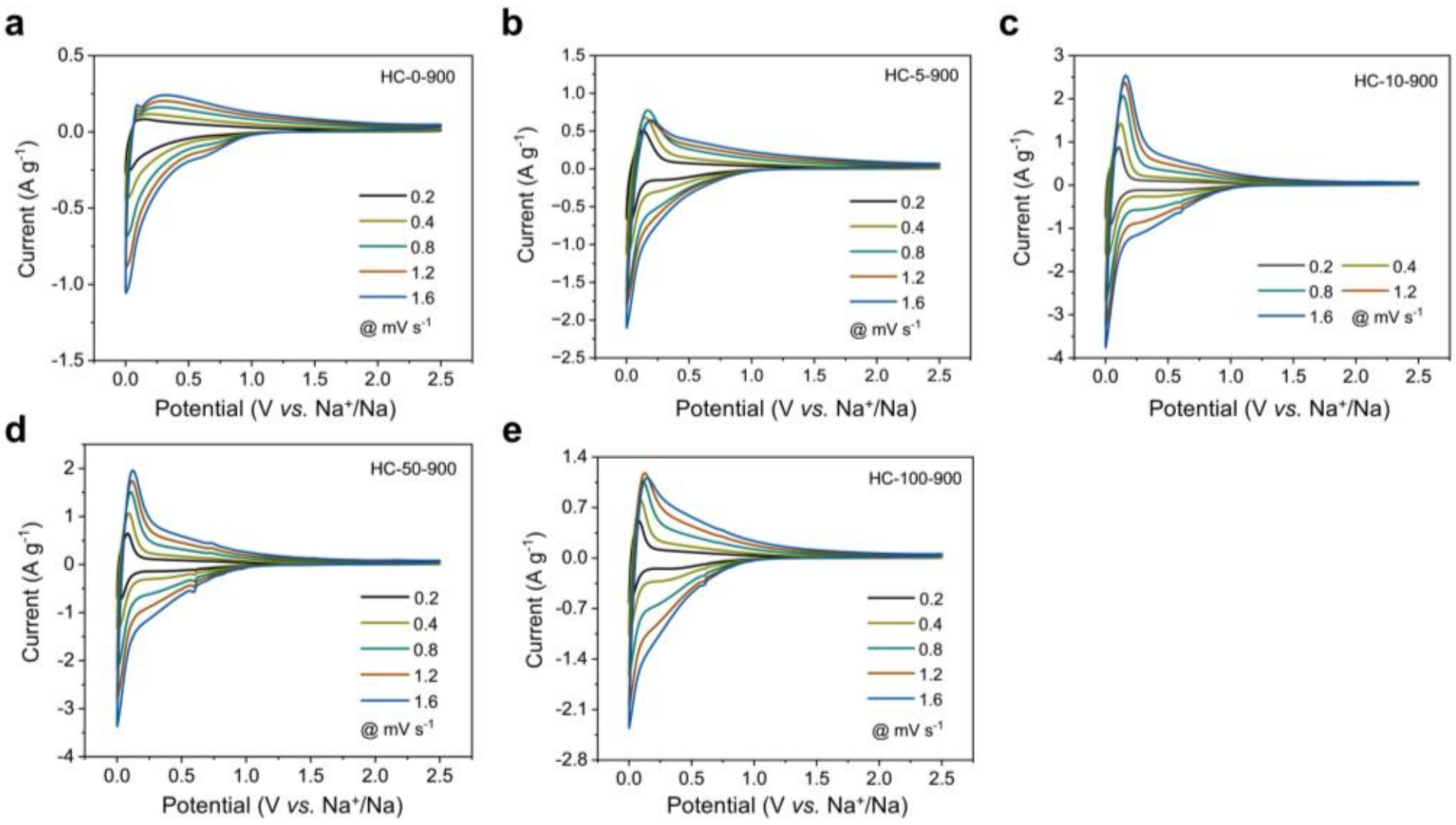

Supplementary Fig. 55. Cyclic voltammetry (CV) curves at different scanning rates of (a) HC-0-900, (b) HC-5-900, (c) HC-10-900, (d) HC-50-900, and (e) HC-100-900 (Applied temperature: 28±0.3 °C).

Supplementary Note 16: To quantify the surface capacitance contribution and the contribution of diffusion control in the overall capacity, the current (i) versus the scan rate (v) was:

$$i = av^b \tag{8}$$

where a and b are adjustable parameters, and i and v denote the peak current and scan rate, respectively. The contributions of diffusion control and surface capacitance can be expressed by Equations (9) and (10):

$$i = k_1 v + k_2 v^{1/2} \tag{9}$$

$$i/v^{1/2} = k_1 v^{1/2} + k_2 \tag{10}$$

where k_1 and k_2 are constants at a fixed potential, and k_1v and $k_2v^{1/2}$ are the contributions of surface-driven capacitive processes and ion diffusion-controlled reactions[54].

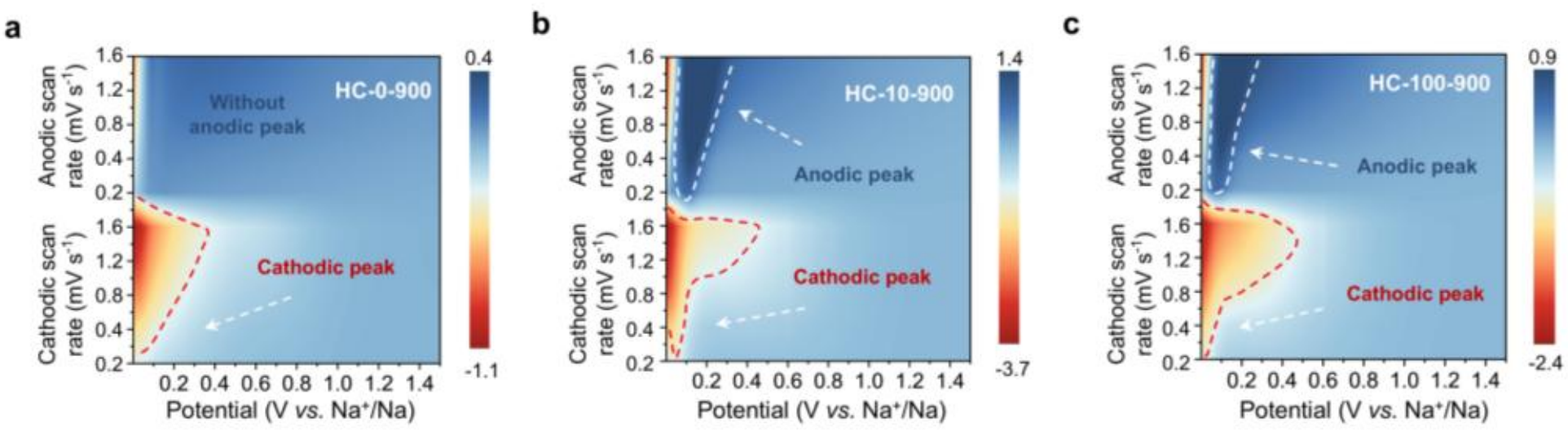

Supplementary Fig. 56. Contour plots of CV patterns for (a) HC-0-900, (b) HC-10-900, and (c) HC-100-900 with different scan rates (Applied temperature: 28±0.3 °C).

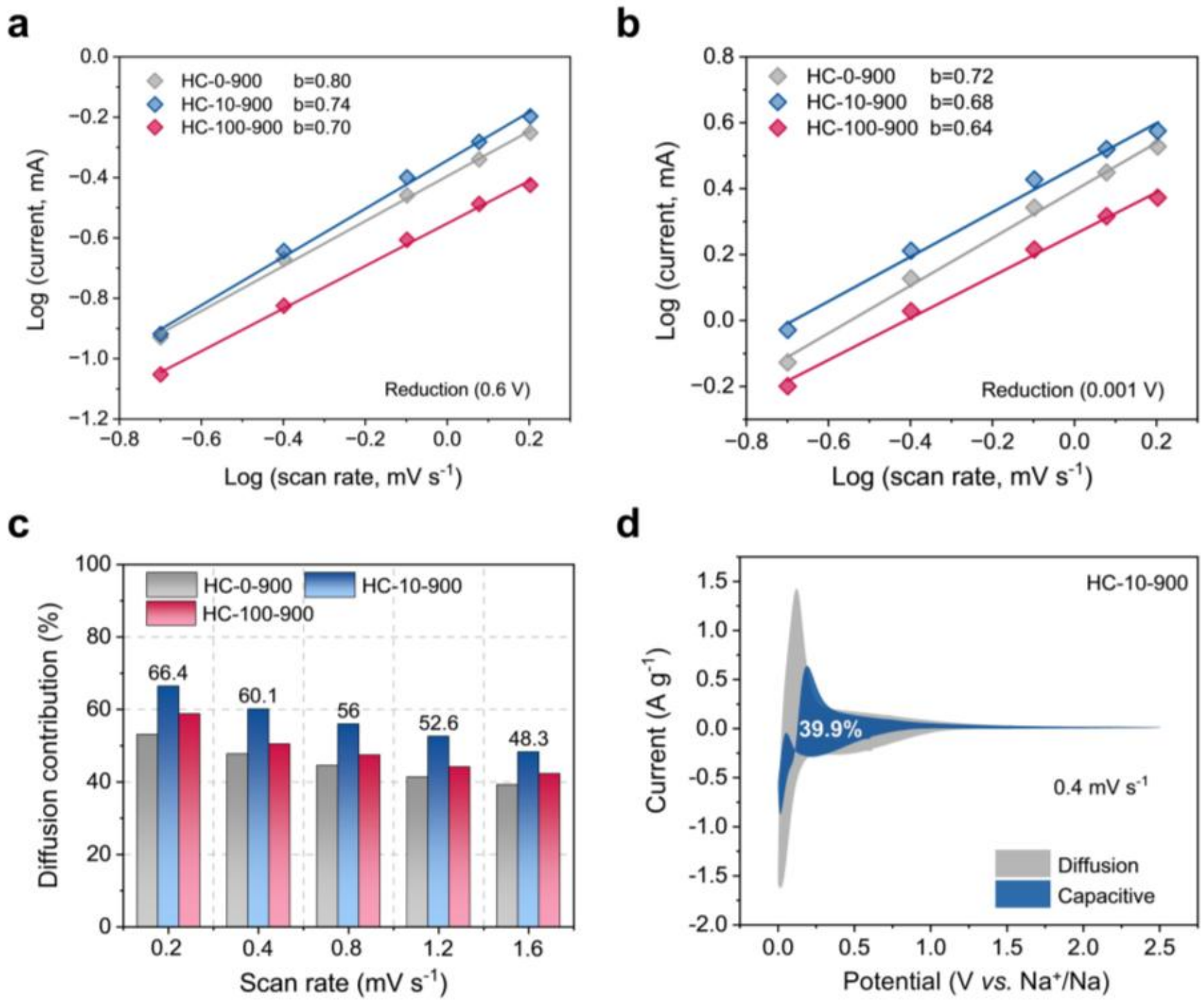

Supplementary Fig. 57. Log (i) vs Log (v) at (a) 0.6 V and (b) 0.001 V of HC-0-900, HC-10-900, and HC-100-900. (c) Diffusion-controlled capacity contribution at different scanning rates. (d) Contribution ratio of diffusion capacities at 0.4 mV s^{-1} of HC-10-900 .

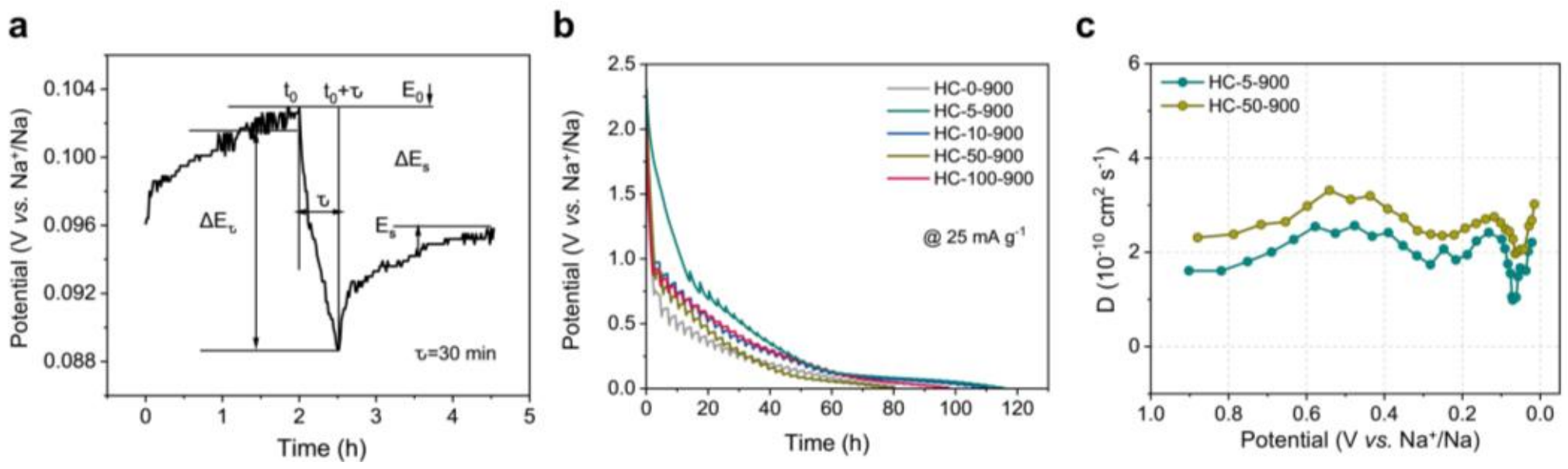

Supplementary Fig. 58. (a) The relationship between potential and time during discharge of HC-10-900 electrode at 0.1 V. (b) The galvanostatic intermittent titration technique (GITT) curves of HC-0-900, HC-5-900, HC-10-900, HC-50-900, and HC-100-900. (c) Diffusion coefficient of HC-5-900 and HC-50-900 (Applied temperature: 28±0.3 °C).

Supplementary Note 17: Na-ion diffusion coefficient (D_{Na+}) can be determined by applying Fick's second law with the following equation:

$$D_{Na^+} = \frac{4}{\pi\tau}\left(\frac{m_B V_M}{M_B S}\right)^2 \left(\frac{dE_S}{dE_\tau}\right)^2 \qquad (11)$$

Where D_{Na+} represents the diffusion coefficient of Na^+ ($cm^2\ s^{-1}$), m_B denotes the mass of the active material (g), Vm is the molar volume of electrode material ($cm^3\ mol^{-1}$), M_B is the relative molecular mass of the material ($g\ mol^{-1}$), S represents the effective surface area of the electrode in contact with the electrolyte (cm^2), τ is the relaxation time, dE_S represents the potential change during the charging/discharging process, and dE_τ represents the potential change when the cell reaches equilibrium during rest[55].

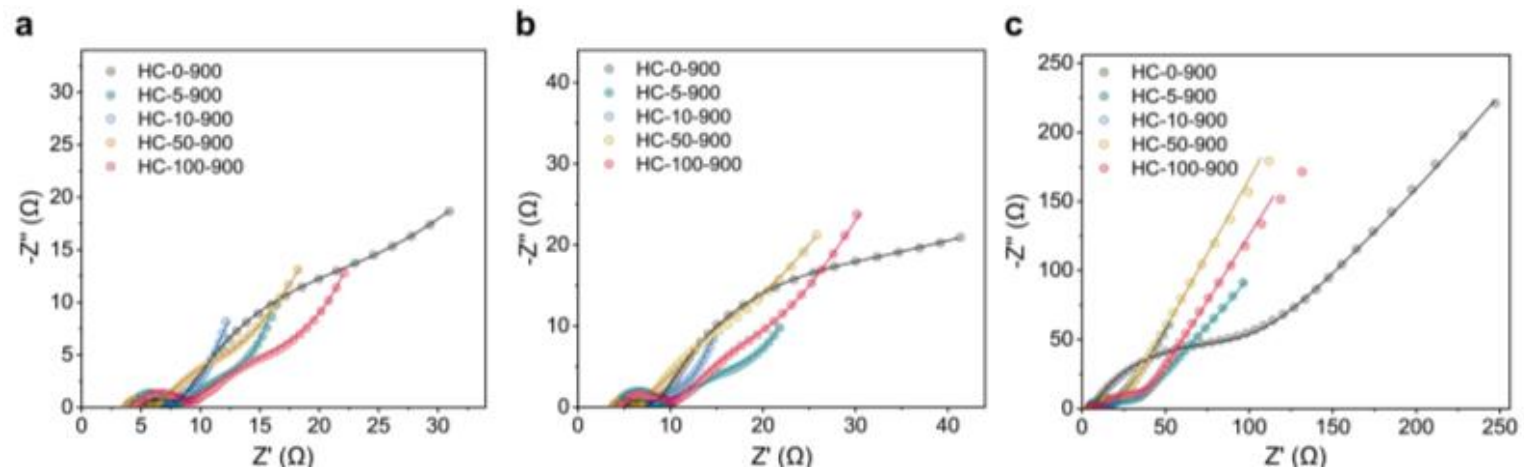

Supplementary Fig. 59. In-situ electrochemical impedance spectroscopy (EIS) during initial discharge cycle at (a) 0.01 V, (b) 0.1 V, and (c) 0.6 V of HC-0-900, HC-5-900, HC-10-900, HC-50-900, and HC-100-900 (Applied temperature: 28±0.3 ℃).

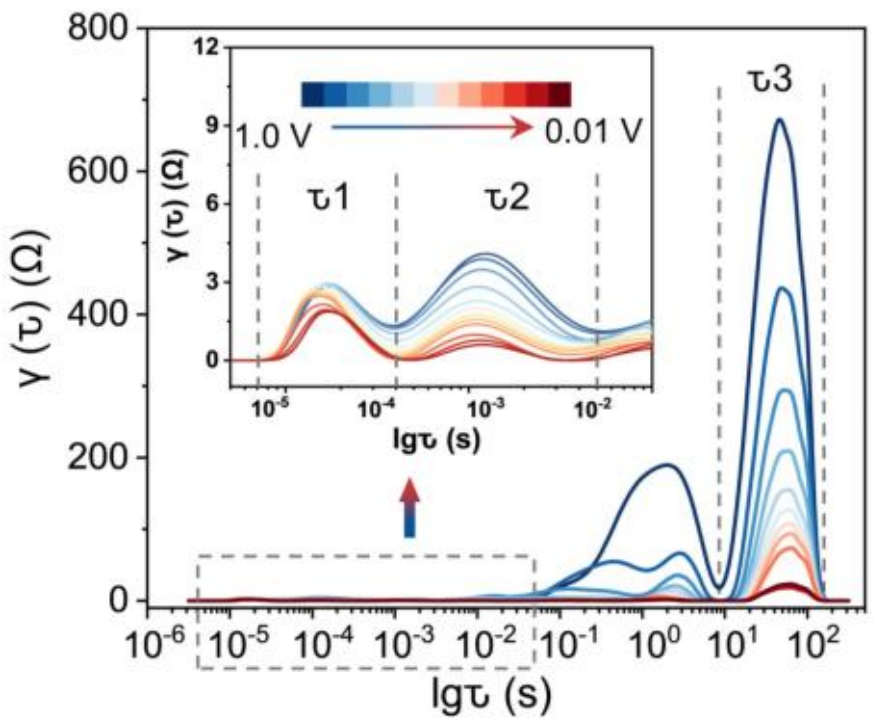

Supplementary Fig. 60. The distribution of relaxation time (DRT) transformation of in-situ EIS spectra for HC-10-900.

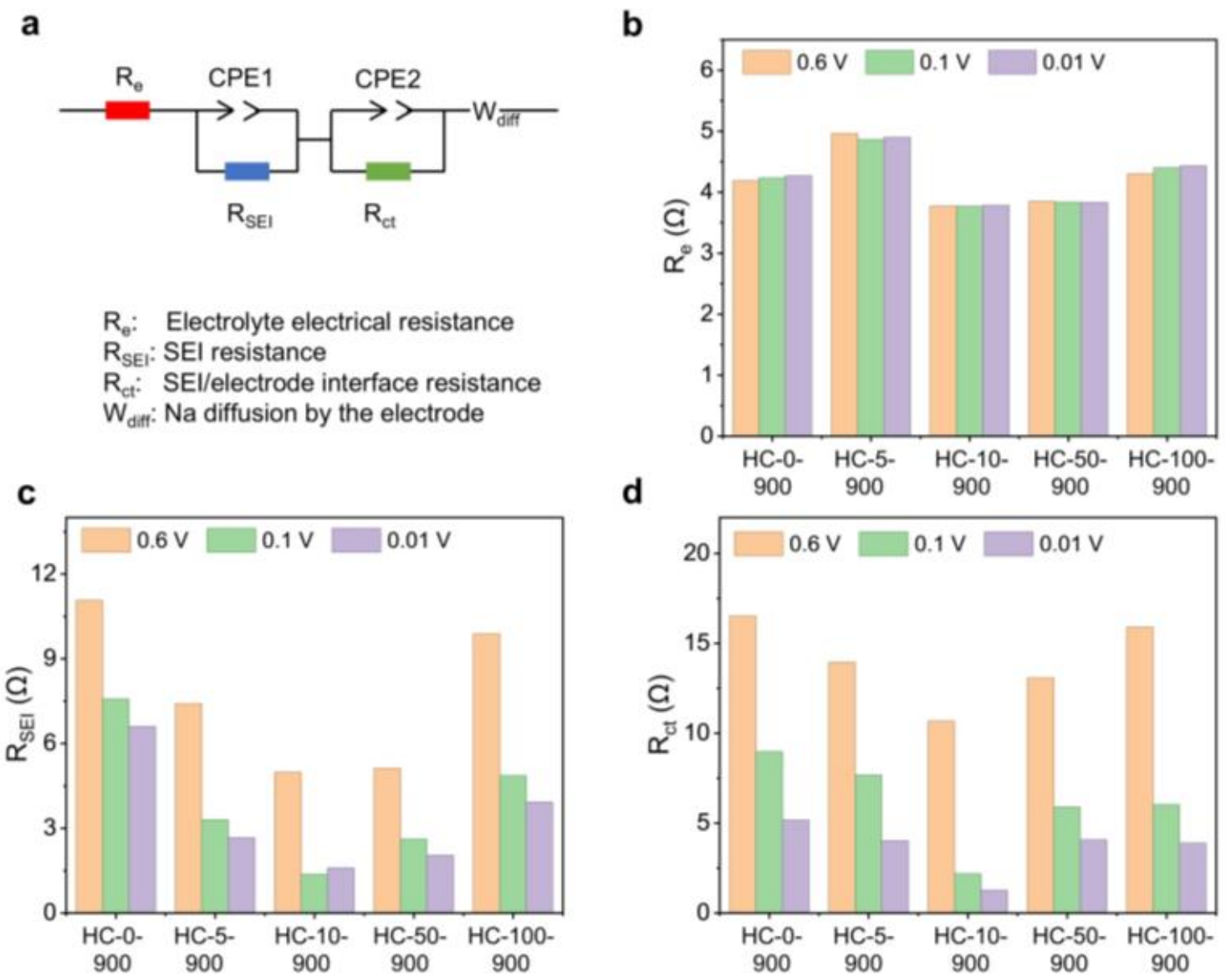

Supplementary Fig. 61. (a) The equivalent circuit diagram. The fitting results of (b) the internal resistance of the electrolyte (R_e), (c) the electrolyte/electrode interfacial resistance (R_s), and (d) the charge-transfer resistance (R_{ct}) in different depths of discharge.

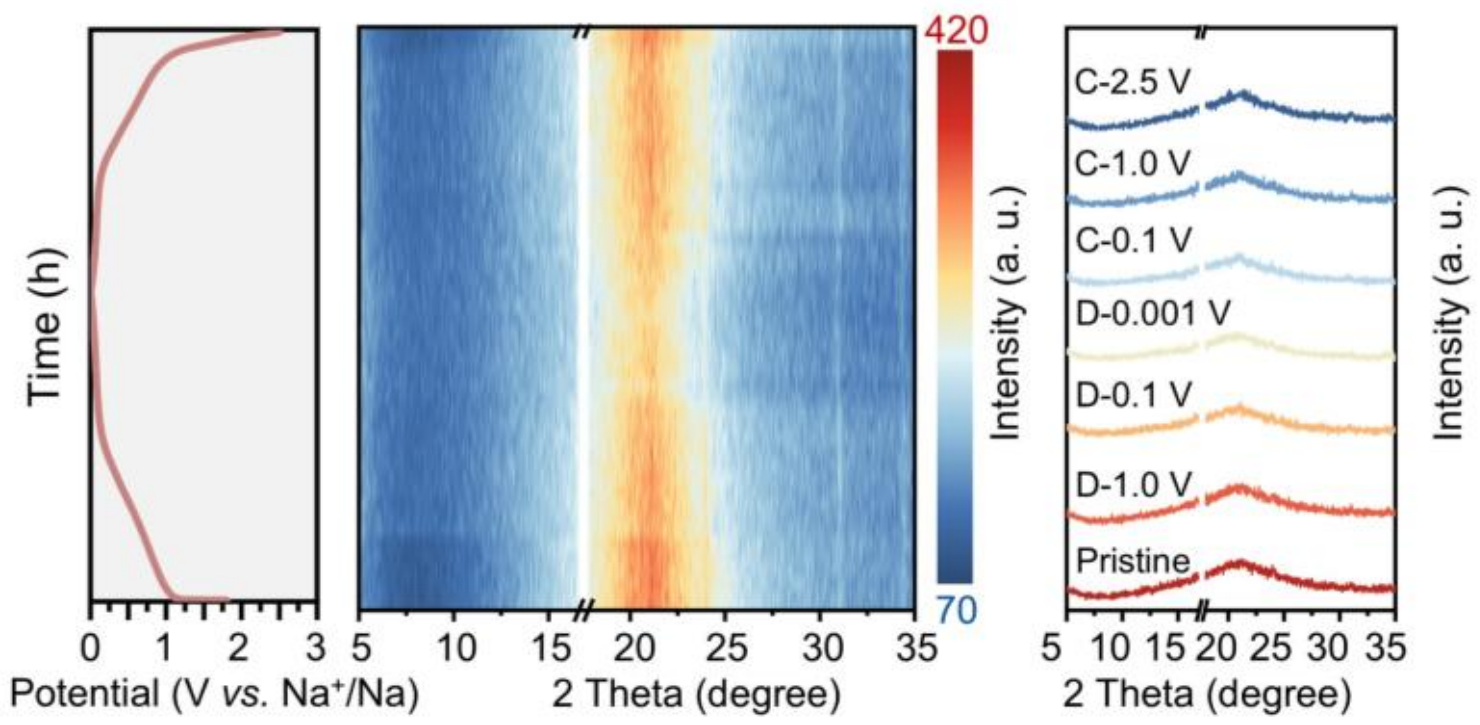

Supplementary Fig. 62. In-situ XRD during initial charge-discharge cycle of HC-10-900 at 25 mA g^{-1} (Voltage window of 0.001-2.5 V, applied temperature: 28±0.3 ℃). The color scale ranges from blue to red, with intensities ranging from 70 to 420.

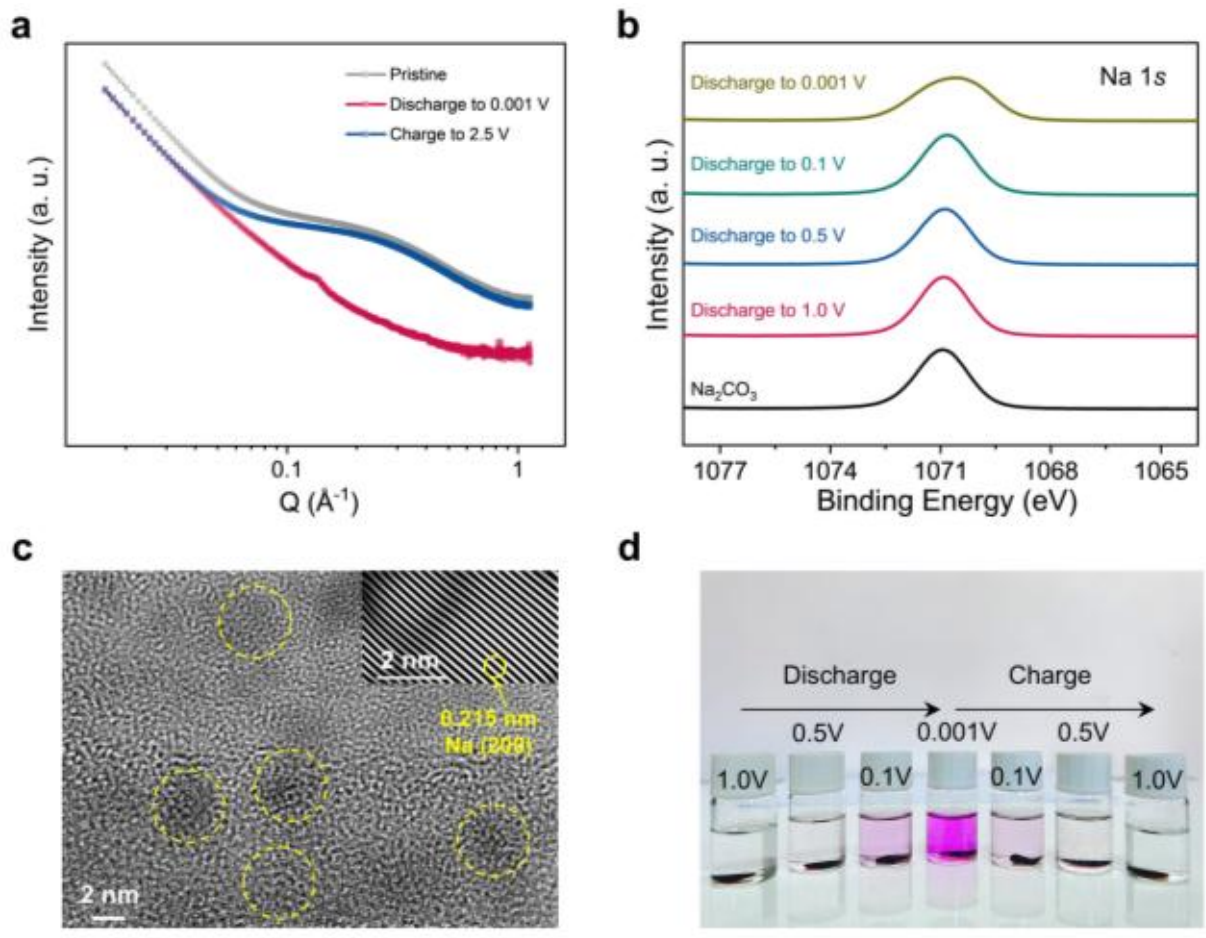

Supplementary Fig. 63. (a) Ex-situ SAXS, (b) ex-situ XPS, (c) HRTEM image at discharge to 0.001 V, and (d) the color reaction of phenolphthalein of HC-10-900. The HC-10-900 electrodes were prepared at the corresponding potential during the initial charge-discharge cycle for ex situ testing (Applied current is 25 mA g^{-1}, voltage window of 0.001-2.5 V, applied temperature: 28±0.3 °C). Prior to testing, the HC-10-900 electrodes were soaked in 30 mL of DMC solution for 12 h and this process was repeated twice at 28±0.3 °C, and then dried at 60 °C. All of the above ex-situ tests were performed in a glove box to prevent contact with air.

Supplementary Note 18: The SAXS patterns of HC-10-900 in the initial stage exhibit a higher shoulder peak at ≈0.3 Å^{-1}, which originates from abundant closed pores. When HC-10-900 is fully sodiation, the broad peak in the SAXS spectrum disappears, indicating that the closed-pore structure has been filled with Na^+. Ex-situ XPS analysis was conducted to further investigate the storage state of sodium within the closed pores. As the potential was discharged to 0.5 V, the position of the Na peak in the XPS spectrum remained unchanged, indicating that sodium is stored in the ramp region in ionic form. When discharged to 0.1 V, the Na peak shifts toward the characteristic of metallic sodium, and upon further discharge to 0.001 V, this shift becomes even more pronounced, confirming the emergence of a metallic sodium phase in the plateau region. A large number of sodium clusters were observed in the HRTEM test, while closed pores cannot be observed, suggesting that the plateau region is caused by the filling of the closed pores by the sodium clusters[56]. The presence of the quasi-metallic sodium cluster in HC-10-900 is further confirmed by the color reaction of phenolphthalein. Along with the discharge process, the clear and transparent ethanol solution of phenolphthalein turns pink and gradually deepens, especially in the plateau region[57]. The filling behavior of Na^+ in closed pores and the storage state of quasi-metallic sodium clusters were demonstrated by a variety of ex situ characterization techniques.

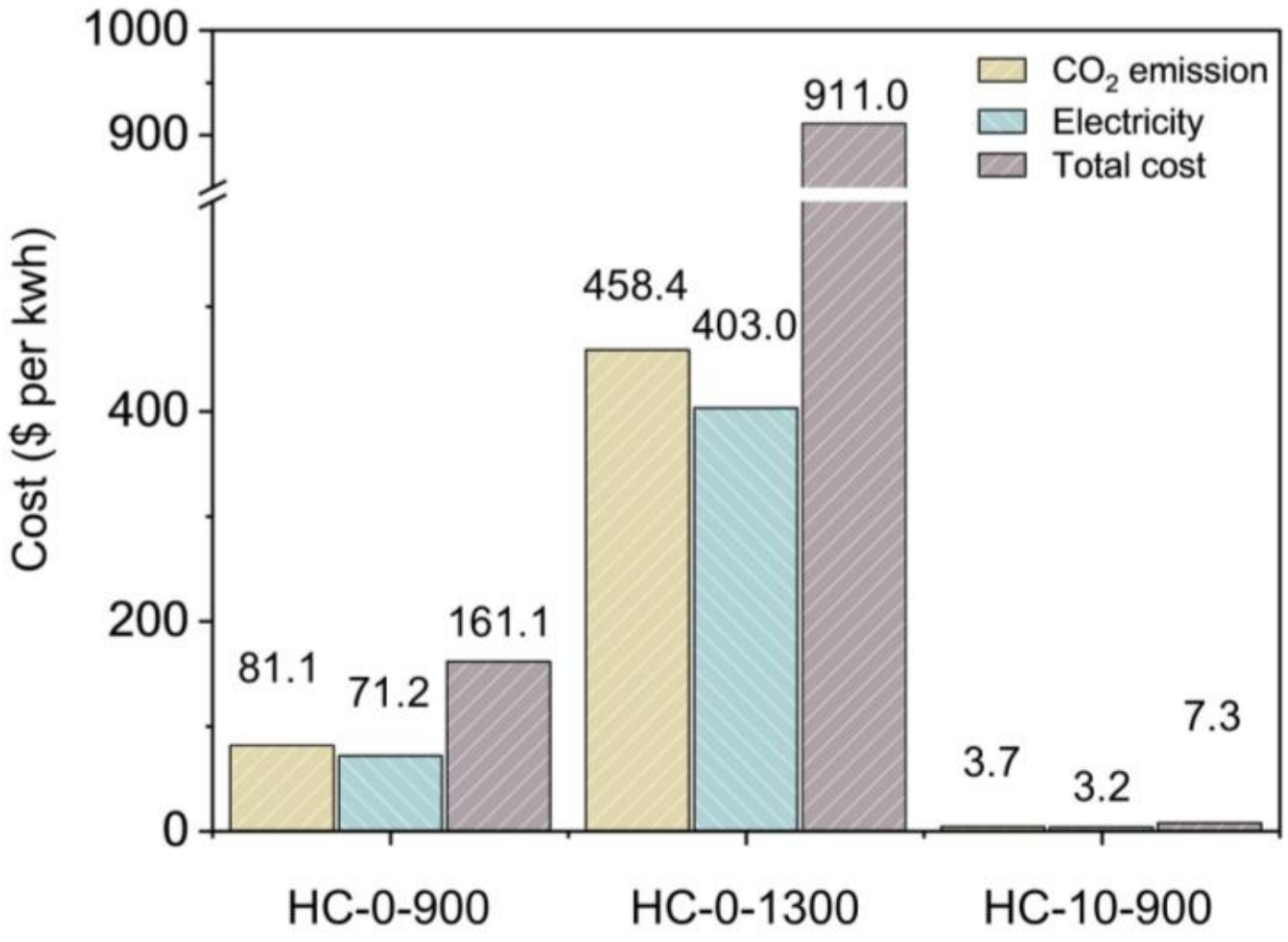

Supplementary Fig. 64. The cost per kWh required in the half battery systems assembled in HC-0-900, HC-0-1300, and HC-10-900, respectively.

Supplementary Table 1. The performance, generalization gaps, and interpretability comparison of multi-models.

Task	Model	5-fold CV mean R^2	5-fold CV std	Test R^2	Generalization gap	SHAP analysis
ICE	Random Forest	0.763	0.019	0.826	-0.062	Yes
	XGB	0.759	0.024	0.825	-0.062	Yes
	GBR	0.734	0.037	0.790	-0.052	Yes
	Neural Network	0.718	0.055	0.830	-0.106	No
	Linear	0.604	0.131	0.678	-0.062	Yes
	Ridge	0.608	0.136	0.674	-0.053	Yes
	Lasso	0.608	0.147	0.664	-0.041	Yes
Plateau capacity	Random Forest	0.805	0.041	0.794	0.012	Yes
	XGB	0.811	0.041	0.780	0.031	Yes
	GBR	0.777	0.041	0.719	0.060	Yes
	Neural Network	0.749	0.042	0.703	0.045	No
	Linear	0.701	0.047	0.671	0.030	Yes
	Ridge	0.704	0.047	0.668	0.036	Yes
	Lasso	0.702	0.047	0.669	0.033	Yes

Supplementary Table 2. The R^2 of the training and test sets of the ICE and reversible plateau capacity in the RF model.

Task	n	CV setting	CV R^2	Train-CV R^2	Test R^2	Test MAE	Test RMSE	CV setting	CV R^2	Test R^2
ICE	565	5-fold	0.763	0.764	0.826	0.257	0.311	10-fold	0.767	0.826
Plateau capacity	565	5-fold	0.805	0.806	0.794	26.93	33.40	10-fold	0.805	0.797

Supplementary Table 3. Carbon layer structure of HC.

Sample	(002)	d_{002} (nm)	L_a (nm)	L_c (nm)	R-factor	$\lvert g^o \rvert$	I_D/I_G
HC-0-900	21.44	0.414	8.01	1.787	1.787	8.14	1.72
HC-5-900	22.26	0.399	8.26	1.854	2.274	6.40	1.50
HC-10-900	23.14	0.384	8.53	1.990	2.372	4.65	1.33
HC-50-900	24.49	0.363	8.99	2.117	2.664	2.21	1.21
HC-100-900	24.77	0.359	10.29	2.312	3.436	1.74	1.01
HC-0-1300	23.40	0.380	9.79	1.895	3.205	4.19	1.05

Supplementary Table 4. Pore structure information of HC.

Sample	SSA BET (m^2 g^{-1})	Open pore volume (cm^3 g^{-1})	SSA SAXS (m^2 g^{-1})	SSA closed open (m^2 g^{-1})	Closed pore radius (nm)	True density (g cm^{-3})	Closed pore volume (cm^3 g^{-1})
HC-0-900	436.5	0.14820	1567.43	1130.93	5.87	1.8551	0.09658
HC-5-900	2.59	0.05900	-	-	-	1.7685	0.12297
HC-10-900	2.28	0.00603	1352.57	1350.29	10.3	1.6109	0.17829
HC-50-900	2.06	0.00486	-	-	-	1.9614	0.06736
HC-100-900	0.12	0.04500	967.84	967.72	7.2	2.1426	0.02424
HC-0-1300	0.68	-	-	-	-	-	-

Supplementary Table 5. The ICE and reversible plateau capacity of HC were prepared at varying carbonization temperatures under atmospheric pressure.

Sample	Carbonization temperature (°C)	ICE (%)	Reversible plateau capacity (mAh g^{-1})	Performance factor (RI)
HC-0-700	700	55.60	26.14	14.53
HC-0-900	900	56.60	36.40	20.60
HC-0-1000	1000	73.69	75.39	55.55
HC-0-1100	1100	77.70	95.66	74.33
HC-0-1200	1200	80.38	137.60	110.60
HC-0-1300	1300	82.89	158.20	131.13
HC-0-1400	1400	75.16	109.34	82.18
HC-10-900	900	87.40	159.40	139.32

Supplementary Table 6. The Comparison of electrochemical performance.

Sample	ICE[a] (%)	Votage hysteresis[b] (V)	Pleteau capacity[c] (mAh g^{-1})	Rate capacity[d] (mAh g^{-1})	Cycling stability[e] (%)
HC-0-900	54.70	0.112	36.40	51.80	87.5
HC-5-900	77.35	0.085	128.24	102.74	98.1
HC-10-900	87.40	0.038	159.44	249.70	98.5
HC-50-900	79.03	0.041	106.20	161.40	97.8
HC-100-900	73.39	0.053	62.50	89.45	99.9

a: ICE at 25 mA g^{-1}.

b: The difference between the oxidation peak and the reduction peak of the CV curve at 0.1 mV s^{-1}.

c: Pleteau capacity of charge at 25 mA g^{-1}.

d: Charge capacity at 1.0 A g^{-1}.

e: Capacity retention rate after 100 cycles at 25 mA g^{-1}.

Supplementary Table 7. Comparison of LTFC with current synthesis strategies and ultrafast carbonization technologies.

Negative electrode	Modification strategies	Process Conditions	Structural Features	Electrochemical performance	Ref
Modification strategies based on traditional thermal field carbonization					
TC1500	Pore engineering	5°C min^{-1}<br>900°C 2h<br>1500°C 2h	d_{002}: 0.367 nm<br>I_D/I_G: 1.14<br>SSA: 1.8 m^2 g^{-1}	75.6% at 20 mA g^{-1}<br>331.51 mAh g^{-1}<br>131.4mAh g^{-1} at 3 A g^{-1}	58
HC-Pc + 1.0 K	Molten activation	5°C min^{-1}<br>300°C 1h<br>1600°C 2h	d_{002}: 0.382 nm<br>I_D/I_G: 1.29<br>SSA: 0.1 m^2 g^{-1}	84.4% at 30 mA g^{-1}<br>357.0 mAh g^{-1}<br>40.5mAh g^{-1} at 1 A g^{-1}	59
ZAHC-1500	Catalytic engineering	5°C min^{-1}<br>500°C 1h<br>2°C min^{-1}<br>1500°C 2h	d_{002}: 0.376 nm<br>I_D/I_G: 2.80<br>SSA: 3.7 m^2 g^{-1}	80.9% at 20 mA g^{-1}<br>342.9 mAh g^{-1}<br>111.9mAh g^{-1} at 1 A g^{-1}	60
CSM-0.8	Pre-oxidation	5°C min^{-1}<br>300°C 1h<br>1200°C 2h	d_{002}: 0.391 nm<br>I_D/I_G: 2.68<br>SSA: 3.0 m^2 g^{-1}	87.9% at 30 mA g^{-1}<br>339.3 mAh g^{-1}<br>99.1mAh g^{-1} at 0.6 A g^{-1}	61
HC-1.5	Heteroatom doping	3°C min^{-1}<br>600°C 2h<br>1200°C 2h	d_{002}: 0.365 nm<br>I_D/I_G: 1.12<br>SSA: 45 m^2 g^{-1}	68.1% at 30 mA g^{-1}<br>299.7 mAh g^{-1}<br>212mAh g^{-1} at 2 A g^{-1}	62
Emerging ultrafast synthesis technologies					
HC-S1200	-	120°C min^{-1}<br>1200°C<br>5min<br>25 MPa	d_{002}: 0.388 nm<br>I_D/I_G: 1.15<br>SSA: 8.7 m^2 g^{-1}	92% at 30 mA g^{-1}<br>340.9 mAh g^{-1}<br>145.8mAh g^{-1} at 3 A g^{-1}	63
HC600-J-1500	-	5°C min^{-1}<br>600°C 3h<br>1500°C	d_{002}: 0.393 nm<br>I_D/I_G: 2.40<br>SSA: 3.0 m^2 g^{-1}	92.4% at 25 mA g^{-1}<br>362.0 mAh g^{-1}<br>135.0mAh g^{-1} at 2 A g^{-1}	64
HC (30s)	-	5°C min^{-1}<br>800°C 3h<br>1550°C	d_{002}: 0.362 nm<br>I_D/I_G: 1.16<br>SSA: 8.3 m^2 g^{-1}	88.4% at 30 mA g^{-1}<br>289.8 mAh g^{-1}<br>187.5mAh g^{-1} at 1.5 A g^{-1}	2
CTSFS 1300	-	20 MPa<br>1300°C 10s	d_{002}: 0.398 nm<br>I_D/I_G: 1.13<br>SSA: 7.3 m^2 g^{-1}	58.8% at 50 mA g^{-1}<br>303.3 mAh g^{-1}<br>240.5mAh g^{-1} at 2 A g^{-1}	65
HC-10-900	-	5°C min^{-1}<br>300°C 1h<br>900°C 2h<br>10MPa	d_{002}: 0.384 nm<br>I_D/I_G: 1.33<br>SSA: 2.3 m^2 g^{-1}	87.4% at 25 mA g^{-1}<br>329.3 mAh g^{-1}<br>205.1mAh g^{-1} at 2 A g^{-1}<br>143.5mAh g^{-1} at 5 A g^{-1}	This work

Supplementary Table 8. The specifications of HC-10-900||NFM pouch cell.

Cell component	Specification	Parameters
Negative electrode (HC-10-900)	Active material mass loading (mg cm^{-2}, each side)	7.0
	Material mass (g)	7.25
	Size (mm × mm)	77*56
	Active material ratio	0.90
	Nickel tabs	0.21
Electrolyte	$NaPF_6$ (mol L^{-1})	1.0
	EC: DEC ratio	1:1
	Quality (g)	3.0
Separator (PP)	Size (mm × mm)	80*58
	Mass (g)	0.74
Positive electrode (NFM)	Active material mass loading (mg cm^{-2}, each side)	12.5
	Material mass (g)	12.15
	Size (mm × mm)	75*54
	Active material ratio	0.96
	Aluminum tabs	0.06
Current collector	Aluminum foil (g)	2.58
Package (Al-plastic film)	Size (mm × mm)	90*74
	Mass (g)	1.86
Pouch cell	Total weight (g)	27.85
Pouch cell (1.5-3.95 V)	Discharge capacity (Ah)	1.47
	Specific energy (Wh kg^{-1})	166.4 (based on the entire cell)
Pouch cell (1.5-4.3 V)	Discharge capacity (Ah)	1.90
	Specific energy(Wh kg^{-1})	208.1 (based on the entire cell)

Supplementary Table 9. Comparison of specific energy in sodium-ion pouch cells.

Positive electrode	Negative electrode	Electrolyte	Capacity (Ah)	Cycling stability (%)	Specific energy (Wh kg^{-1})	Ref.
$Na_{2/3}Ni_{1/3}Mn_{1/3}Ti_{1/3}O_2$	PFPI-13	1.0 M $NaPF_6$ in EC: DEC (1:1)	2.2	-	108.5	6
$NaNi_{1/3}Fe_{1/3}Mn_{1/3}O_2$	OP-HC	1.0 M $NaSO_3CF_3$ in DEGDME	1.239	94.5% at 400	150.0	4
$Na_3V_2(PO_4)_3$	PPHC900	1.0 M $NaClO_4$ in EC: DMC (1:1)	0.1	59.8% at 300	135.2	5
$Na_4Fe_3(PO_4)_2P_2O_7$	Metallic Sodium	1.0 M $NaPF_6$ in DEGDME	2.28	95.2% at 180	203.0	1
$Na_3V_2(PO_4)_3$	Metallic Sodium	NZSP–TLM	0.1145	-	220.0	66
$Na_3V_2(PO_4)_3$	HC (30 s)	1.0 M $NaPF_6$ in diglyme	--	-	189.3 (Based on active material mass)	2
$Na_3V_2(PO_4)_3$@C	CFG	1.0 M $NaClO_4$ in diglyme	--	63.4% at 50	144.0 (Based on active material mass)	3
$NaNi_{1/3}Fe_{1/3}Mn_{1/3}O_2$	TAC-1400	1.0 M $NaClO_4$ in EC: DEC (1:1)	0.4	-	254.3 (Based on active material mass)	67
$Na_3V_2(PO_4)_3$	G1500	1.0 M $NaPF_6$ in EC: DMC (1:1)	--	-	209.0 (Based on active material mass)	26
$Na_3V_2(PO_4)_3$	PAHC-1500	1.0 M $NaPF_6$ in diglyme	--	77.7% at 100	242.1 (Based on active material mass)	7
commercial PB	RHC	1.0 M $NaPF_6$ in DEGDME	0.72	80.9% at 200	-	8
Prussian blue	HC-AL	1.0 M $NaPF_6$ in DEGDME	0.77	83.7% at 500	-	9
VF-NFMO	HC	1.0 M $NaPF_6$ in EC: DMC (1:1)	2.94	78.0% at 600	120.0	10
$NaNi_{1/3}Fe_{1/3}Mn_{1/3}O_2$	HC-10-900	1.0 M $NaPF_6$ in EC: DEC (1:1) (1.5-3.95 V)	1.47 at 0.1 A<br>1.23 at 2.0 A	96.1% at 1000	166.4 (based on the entire cell)<br>124.3 (based on the entire cell)	This work

Supplementary Table 10. Parameter assumptions for the laboratory-scale cost and CO_2 emission assessment.

Process	Variables	Unit	Data source	HC-10-900	HC-0-1300	HC-0-900
Full battery	Negative material mass	mg	Experiment	3.23	4.88	3.57
Full battery	Positive material mass	mg	Experiment	2.99	4.4	9.28
Full battery	C1_full battery	Wh	Experiment	0.002174	0.002585	0.001586
Full battery	C80_capacity	Wh	Experiment	0.001955	0.001749	0.001127
Full battery	R80	--	Experiment	0.8906	0.6517	0.7122
Full battery	N/P	--	Experiment	1.08	1.11	1.06
Half battery	C1_half_battery	Wh	Experiment	0.000159	0.000182	0.000107
Half battery	C40_half_battery	Wh	Experiment	0.000158	0.000155	0.000088
Half battery	R40	--	Experiment	0.9937	0.8517	0.8224
Half battery	Negative electrode mass	mg	Experiment	1.56	2.06	0.91
Pre-carbonization	Pre-carbonization Energy	kWh/1kg feedstock	Experiment	1.72	1.72	1.72
Pre-carbonization	Pre-carbonization output ratio	--	Experiment	0.528	0.528	0.528
Pre-carbonization	Mass fraction of C in pre-treated feed	%	Experiment	67.5	67.5	67.5
Pre-carbonization	Pre-carbonization CO_2 emissions	g/100g feed	Experiment	16.14	16.14	16.14
Pre-carbonization	Hours run	--	Experiment	2	2	2
Pyrolysis	HC mass per 8g pre-treated feed	g	Experiment	3.472	1.584	2.872
Pyrolysis	Mass fraction of C in HC	%	Experiment	93.5	82.50	82.75
Pyrolysis	Pyrolysis CO_2 emission	g/8g feed	Experiment	7.87	14.98	11.06
Pyrolysis	Pyrolysis Energy	kWh/8g feedstock	Experiment	15.4	30.6	10.6
Pyrolysis	Hard Carbon output ratio	--	Experiment	0.0787	0.1498	0.1106
Pyrolysis	Hours run	--	Experiment	5	8	5

Electricity price	$/kWh	Assumption	0.088	0.088	0.088
Carbon intensity	$kgCO_2/kWh$	Assumption	0.5	0.5	0.5
SCC_China	$/kg$CO_2$	Assumption	0.015	0.015	0.015
SCC_Global	$/kg$CO_2$	Assumption	0.2	0.2	0.2
Hourly equipment depreciation cost	$/hr	Assumption	0.016	0.033	0.011
Labor & overhead (% of equipment depreciation cost)	--	Assumption	0.05	0.05	0.05
Feedstock price	$/kg	Assumption	4	4	4

Supplementary Table 11. Calculation results of the laboratory-scale cost and CO_2 emission assessment.

-	**HC-10-900**	**HC-0-1300**	**HC-0-900**
feed_cost	0.4	0.4	0.4
eqpt_pre	0.021743858	0.021743858	0.021743858
labor_pre	0.001087193	0.001087193	0.001087193
elec_pre	0.15136	0.15136	0.15136
eqpt_pyro	0.570776255	1.826484016	0.380517515
labor_pyro	0.028538813	0.091324201	0.019025876
elec_pyro	9.4864	18.8496	6.5296
total_eqpt	0.592520113	1.848227874	0.402261373
total_labor_overhead	0.029626006	0.092411394	0.020113069
total_electricity	9.63776	19.00096	6.68096
total_electricity_use	109.52	215.92	75.92
elec_emissions_kg	54.76	107.96	37.96
process_emissions_pre_kg	0.01614	0.01614	0.01614
process_emissions_pyro_kg	0.051942	0.098868	0.072996
total_emissions_kg	54.828082	108.075008	38.049136
carbon_cost_china	0.82242123	1.62112512	0.57073704
carbon_cost_global	10.9656164	21.6150016	7.6098272
total_private_cost	10.65990612	21.34159927	7.503334442

total_w_scc_china	11.48232735	22.96272439	8.074071482
total_w_scc_global	21.62552252	42.95660087	15.11316164
output_mass_g	22.9152	10.4544	18.9552
cost_per_g	0.465189312	2.041398767	0.395845702
cost_per_kg	465.1893118	2041.398767	395.8457015
cost_per_g_w_scc_china	0.501079081	2.196465066	0.425955489
cost_per_kg_w_scc_china	501.0790806	2196.465066	425.9554888
cost_per_g_w_scc_global	0.943719563	4.108949425	0.797309532
cost_per_kg_w_scc_global	943.7195625	4108.949425	797.309532
retention_per_cycle	0.998673648	0.995128389	0.995738408
decay_pct_per_cycle	0.132635206	0.487161142	0.426159214
cycle_to_80_pct	170	47	54
total_energy_1000_wh	0.12043808	0.052660823	0.036696153
total_energy_to80_wh	0.033107214	0.010882546	0.007664926
total_energy_to80_kwh_per_kg_HC	102.4991155	22.30030004	21.47038067
value_1000_total_usd	1.20E-05	5.27E-06	3.67E-06
value_to80_total_usd	3.31E-06	1.09E-06	7.66E-07
negative electrode_share_from_np	0.519230769	0.526066351	0.514563107
rev_per_kg_HC_1000_usd	19.36072975	5.676862056	5.289211928
rev_per_kg_HC_to80_usd	5.322069461	1.173143746	1.104786578
cost_per_kwh	4.538471474	91.54131393	18.43682735
cost_per_kwh_w_scc_china	4.888618582	98.49486609	19.83921456
cost_per_kwh_w_scc_global	9.207099573	184.2553426	37.13532351
retention_per_cycle_half	0.999842283	0.995993508	0.995124623
cycle_to_80_pct_half	1416	57	47
total_energy_to80_wh_half	0.020178935	0.000929127	0.00045042
total_energy_to80_kwh_per_kg_HC_half	129.3521503	4.510327403	4.949667706
cost_per_kwh_half	3.596301343	452.6054506	79.97419727
cost_per_kwh_half_w_scc_china	3.873759187	486.9857263	86.05739094
cost_per_kwh_half_w_scc_global	7.295739269	911.0091259	161.0834463

Supplementary Table 12. Cost of preparing HC using 100g feedstock.

-	Composition	HC-0-900	HC-10-900	HC-0-1300
Precursor	$C_6H_{12}O_6$ (g)	100	100	100
Private costs	Feedstock price ($)	0.4	0.4	0.4
	Equipment depreciation ($)	0.40	0.59	1.85
	Labor and overhead ($)	0.02	0.03	0.09
	Electricity ($)	6.68	9.64	19.00
Social costs	CO_2 emissions of electricity ($)	7.59	10.95	21.59
	CO_2 emissions of materials ($)	0.018	0.014	0.023
Total cost	Private and social costs ($)	15.11	21.62	42.95
HC	Yield (g)	18.96	22.92	10.45

Supplementary Table 13. The cost per kWh required for the full battery systems assembled in HC.

-	Composition	HC-0-900	HC-10-900	HC-0-1300
Private costs	Feedstock price ($/kg HC)	21.10	17.46	38.26
	Equipment depreciation ($/kg HC)	21.22	25.86	176.79
	Labor and overhead ($/kg HC)	1.06	1.29	8.84
	Electricity ($/kg HC)	352.46	420.58	1817.51
Social costs	CO_2 emissions ($/kg HC)	401.46	478.53	2067.55
Total cost	Private and social costs ($/kg HC)	797.31	943.72	4108.95
Full battery	Total energy (kWh/kg HC)	21.47	102.50	22.30
Cost per kWh	Equipment depreciation ($/kWh)	0.99	0.25	7.93
	Electricity ($/kWh)	16.42	4.10	81.50
	CO_2 emissions ($/kWh)	18.70	4.67	92.72
	Total cost ($/kWh)	37.14	9.21	184.26

Supplementary References

1. Ruan, J. et al. Current collector interphase design for high-energy and stable anode-less sodium batteries. *Nat. Sustain.* **8**, 530-541 (2025).
2. Liu, J. et al. Precisely tunable instantaneous carbon rearrangement enables low-working-potential hard carbon toward sodium-ion batteries with enhanced energy density. *Adv. Mater.* **36**, 2407369 (2024).
3. Chen, W. et al. High-performance flexible freestanding anode with hierarchical 3D carbon-networks/Fe_7S_8/graphene for applicable sodium-ion batteries. *Adv. Mater.* **31**, 1806664 (2019).
4. You, S. et al. Hard carbon with opened pore structure for enhanced sodium storage performance. *Energy Environ. Sci.* **17**, 8189-8197 (2024).
5. Bhawana, K. et al. Endorsing Na^+ storage mechanism in low tortuosity, high plateau capacity hard carbon towards development of high-performance sodium-ion pouch cells. *Carbon* **214**, 118319 (2023).
6. Xiao, S. et al. Insight into the role of closed-pore size on rate capability of hard carbon for fast-charging sodium-ion batteries. *Adv. Mater.* **37**, 2501434 (2025).
7. Ren, G. et al. Elemental regulation and ultra-micropore defects engineering for enhancing low-potential Na^+ storage in biomass-derived hard carbon. *Energy Storage Mater.* **80**, 104367 (2025).
8. Wu, C. et al. Industrial-scale hard carbon designed to regulate electrochemical polarization for fast sodium storage. *Angew. Chem. Int. Ed.* **63**, e202406889 (2024).
9. Wu, C. et al. Unraveling structure-performance relationship in hard carbon for sodium-ion battery by coupling key structural parameters. *Energy Environ. Sci.* **18**, 6019-6031 (2025).
10. Tang, Y. et al. Sustainable layered cathode with suppressed phase transition for long-life sodium-ion batteries. *Nat. Sustain.* **7**, 348-359 (2024).
11. Peng, J., Wang, H., Shi, X., & Fan, H. Ultrahigh plateau-capacity sodium storage by plugging open pores. *Adv. Mater.* **37**, 2410326 (2025).
12. Wang, F. et al. Pushing slope- to plateau-type behavior in hard carbon for sodium-ion batteries via local structure rearrangement. *Energy Environ. Sci.* **18**, 4312-4323 (2025).
13. Lan, N. et al. Hyper-crosslinking to customize ultrathin-wall closed pores in pitch-derived carbon for sodium-ion batteries. *Adv. Mater.* **37**, 2419528 (2025).
14. Yin, X. et al. Enabling fast Na^+ transfer kinetics in the whole voltage-region of hard-carbon anodes for ultrahigh rate sodium storage. *Adv. Mater.* **34**, 2109282 (2022).
15. Owusu, S. Y., Soni, R. U. & Leventis, C. S. Catalytic synthesis of iron-doped graphitic aerogels from poly(phloroglucinol-terephthalaldehyde-urethane) precursors. *Carbon Trends* **21**, 100573 (2025).
16. Lu, H. et al. Exploring sodium-ion storage mechanism in hard carbons with different microstructure prepared by ball-milling method. *Small* **14**, 1802694 (2018).
17. Koberstein, J., Morra, B. & Stein, R. The determination of diffuse-boundary thicknesses of polymers by small-angle X-ray scattering. *J. Appl. Cryst.* **13**, 34-45 (1980).
18. Koch, M., Vachette, P. & Svergun, D. Small-angle scattering: a view on the properties, structures and structural changes of biological macromolecules in solution. *Q. Rev. Biophys.* **36**, 147-227 (2003).
19. Li, Y. et al. Regulating pore structure of hierarchical porous waste cork-derived hard carbon anode for enhanced Na storage performance. *Adv. Energy Mater.* **9**, 1902852 (2019).
20. Zhao, J. et al. Catalytic defect-repairing using manganese ions for hard carbon anode with high-

capacity and high-initial Coulombic-efficiency in sodium-ion batteries. *Adv. Energy Mater.* **13**, 230044 (2023).
21. Huang, Y. et al. Rationally designing closed pore structure by carbon dots to evoke sodium storage sites of hard carbon in low-potential region. *Adv. Funct. Mater.* **34**, 2308392 (2023).
22. Lu, Y. et al. Pre-oxidation-tuned microstructures of carbon anodes derived from pitch for enhancing Na storage performance. *Adv. Energy Mater.* **8**, 1800108 (2018).
23. Tang, Z. et al. Revealing the closed pore formation of waste wood-derived hard carbon for advanced sodium-ion battery. *Nat. Commun.* **14**, 6024 (2023).
24. Xu, Z. et al. The Role of Hydrothermal carbonization in sustainable sodium-ion battery anodes. *Adv. Energy Mater.* **12**, 2200208 (2022).
25. Guo, Z. et al. Investigating the superior performance of hard carbon anodes in sodium-ion compared with lithium- and potassium-ion batteries. *Adv. Mater.* **35**, 2304091 (2023).
26. Cheng, D., Li, Z., Zhang, M., Duan, Z., Wang, J. & Wang, C. Engineering ultrathin carbon layer on porous hard carbon boosts sodium storage with high initial coulombic efficiency. *ACS Nano* **17**, 19063-19075 (2023).
27. Chen, H., Sun, N., Zhu, Q., Soomro, R. & Xu, B. Microcrystalline hybridization enhanced coal-based carbon anode for advanced sodium-ion batteries. *Adv. Sci.* **9**, 2200023 (2022).
28. Nie, W. et al. Surface organic nitrogen-doping disordered biomass carbon materials with superior cycle stability in the sodium-ion batteries. *J. Power Sources* **522**, 230994 (2022).
29. Zhang, Y. et al. Optimizing the crystallite structure of lignin-based nanospheres by resignification for high-performance sodium-ion battery anodes. *Energy Technol.* **8**, 1900694 (2020).
30. Ghimbeu, C., Zhang, B., Yuso, A., Réty, B. & Tarascon, J. Valorizing low cost and renewable lignin as hard carbon for Na-ion batteries: Impact of lignin grade. *Carbon* **153**, 634-647 (2019).
31. Pan, G. et al. Vascular tissue-derived hard carbon with ultra-high rate capability for sodium-ion storage. *Carbon* **224**, 118955 (2024).
32. Bobyleva, Z. et al. Caramelization as a key stage for the preparation of monolithic hard carbon with advanced performance in sodium-ion batteries. *ACS Appl. Energy Mater.* **6**, 181-190 (2023).
33. Nita, C., Zhang, B., Dentzer, J. & Ghimbeu, C. Hard carbon derived from coconut shells, walnut shells, and corn silk biomass waste exhibiting high capacity for Na-ion batteries. *J. Energy Chem.* **58**, 207-218 (2021).
34. Huang, S. et al. Three-dimensional hierarchical porous hard carbon for excellent sodium/potassium storage and mechanism investigation. *Mater. Today Energy* **20**, 100673 (2021).
35. Zhang, Q., Deng, X., Ji, M., Li, Y. & Shi, Z. Hard carbon microspheres derived from resorcinol formaldehyde resin as high-performance anode materials for sodium-ion battery. *Ionics* **26**, 4523-4532 (2020).
36. Zhang, Y. et al. Designing hard carbon microsphere structure via halogenation amination and oxidative polymerization reactions for sodium ion insertion mechanism investigation. *J. Colloid Interface Sci.* **668**, 202-212 (2024).
37. Zhu, Y., Chen, M., Li, Q., Yuan, C. & Wang, C. High-yield humic acid-based hard carbons as promising anode materials for sodium-ion batteries. *Carbon* **123**, 727-734 (2017).
38. Wang, P., Zhu, X., Wang, Q., Xu, X., Zhou, X. & Bao, J. Kelp-derived hard carbons as advanced

anode materials for sodium-ion batteries. *J. Mater. Chem. A* **5**, 5761-5769 (2017).
39. Nie, W. et al. Hierarchical porous carbon anode materials derived from rice husks with high capacity and long cycling stability for sodium-ion batteries. *ChemElectroChem* **7**, 631-641 (2020).
40. Rao, Y., Saisrinu, Y., Khatua, S., Bharathi, K. & Patro, L. Nitrogen doped soap-nut seeds derived hard carbon as an efficient anode material for Na-ion batteries. *J. Alloys Compd.* **968**, 171917 (2023).
41. Wang, J., Yan, L., Ren, Q., Fan, L., Zhang, F. & Shi, Z. Facile hydrothermal treatment route of reed straw-derived hard carbon for high performance sodium ion battery. *Electrochim. Acta* **291**, 188-196 (2018).
42. Sun, N. et al. Extended "adsorption–insertion" model: A new insight into the sodium storage mechanism of hard carbons. *Adv. Energy Mater.* **9**, 1901351 (2019).
43. Zhang, X. et al. Pore structure modification of pitch-derived hard carbon for enhanced pore filling sodium storage. *Energy Technol.* **10**, 2200612 (2022).
44. Wang, X. et al Enhancing sodium-ion energy storage of commercial activated carbon by constructing closed pores via ball milling. *Nanomaterials* **14**, 14010065 (2024).
45. Ding, H. et al. Pre-crosslinking enables a promising cyclodextrin-derived carbon anode with large plateau capacity for sodium ion batteries. *New J. Chem.* **48**, 1182-1191 (2024).
46. Song, N. et al. A novel method of enhancing the nitrogen content in biomass hard carbon: Pyrolysis-deposition-self-doping strategy. *Int. J. Energy Res.* **46**, 9892-9899 (2022).
47. Zhang, X. et al. Insight into the effect of structural differences among pitch fractions on sodium storage performance of pitch-derived hard carbons. *Carbon* **226**, 119165 (2024).
48. Au, H. et al. A revised mechanistic model for sodium insertion in hard carbons. *Energy Environ. Sci.* **13**, 3469-3479 (2020).
49. Wang, K. et al. Altering thermal transformation pathway to create closed pores in coal-derived hard carbon and boosting of Na^+ plateau storage for high-performance sodium-ion battery and sodium-ion capacitor. *Adv. Funct. Mater.* **32**, 2203725 (2022).
50. Yang, B. et al. Engineering hard carbon with high initial coulomb efficiency for practical sodium-ion batteries. *J. Power Sources* **492**, 229656 (2021).
51. Liu, P., Li, Y., Hu, Y., Li, H., Chen, L. & Huang, X. A waste biomass derived hard carbon as a high-performance anode material for sodium-ion batteries. *J. Mater. Chem. A* **4**, 13046-13052 (2016).
52. Xu, R., Sun, N., Zhou, H., Chang, X., Soomro, R. & Xu, B. Hard carbon anodes derived from phenolic resin/sucrose cross-linking network for high-performance sodium-ion batteries. *Battery Energy* **2**, 20220054 (2023).
53. Wang, B. et al. Resolution of the reciprocity between radical species from precursor and closed pore formation in hard carbon for sodium storage. *J. Colloid Interface Sci.* **664**, 681-690 (2024).
54. Fan, S. et al. Revealing capacitive slope capacity of open pore carbon for ultrahigh-rate sodium-ion storage. *Adv. Mater.* e13223 (2025).
55. Huang, P. et al. Spatiotemporal evolution in hard carbon synthesis via electrothermal coupling strategy for high-performance sodium-ion batteries. *Adv. Mater.* **37**, 2507521 (2025).
56. Xue, Y. et al. Substitution index-prediction rules for low-potential plateau of hard carbon anodes in sodium-ion batteries. *Adv. Mater.* **37**, 2417886 (2025).
57. Mao, P. et al. Hard carbon with tailored microstructure via thermal regulation for high-

efficiency sodium-ion batteries. *J. Colloid Interface Sci.* **710**, 140026 (2026).
58. Wang, M. et al. Gas-driven pore engineering: From micropore generation to closed-pore structure modulation in coal-derived hard carbon for sodium-ion batteries. *Carbon* **248**, 121171 (2026).
59. Wen, C. et al. Construction of loose closed pore structure in pitch derived hard carbon via molten activation strategy for high-performance sodium-ion batteries. *Chem. Eng. J.* **515**, 163754 (2025).
60. Cheng, F. Wang, M. Ju, Z. Jiang, J. Xing, Y. & Gui, X. Unlocking plateau capacity of coal-derived hard carbon with tailored microcrystal structure via induced-catalytic engineering for sodium-ion batteries. *Chem. Eng. J.* **519**, 165512 (2025).
61. Zhang, S. et al. Oxidative-driven microstructure regulation of starch-derived hard carbon for efffcient Na-ion storage. *Chem. Eng. J.* **519**, 165355 (2025).
62. Wang, Z. et al. Tuning the disordered microdomains in hard carbon via nitrogen doping for high-performance sodium storage. *Chem. Eng. J.* **523**, 168490 (2025).
63. Song, Z. et al. Joule heating for structure reconstruction of hard carbon with superiorsodium ion storage performance. *Chem. Eng. J.* **496**, 154103 (2024).
64. Qiu, Y. et al. Rapid closed pore regulation of biomass-derived hard carbons based on flash joule heating for enhanced sodium ion storage. *Adv. Funct. Mater.* **35**, 2423559 (2025).
65. Yuan, M. Yu, S. Wang, K. Mi, C. & Shen, L. Ultrafast synthesis of hard carbon for high-rate and low-temperature sodium-ion storage through fash Joule heating. *Solid State Ionics* **414**, 116622 (2024).
66. An, H. et al. Hybrid electrolyte enables solid-state sodium batteries sustaining 50,000 cycles. *Nat. Sustain.* **8**, 661-671 (2025).
67. Wang, K., Sun, F., Wang, H., Wu, D., Zhao, G. & Su, X. Spatially confined carbonization-induced reorganization of microcrystals and nanopores in carbon framework for enhanced sodium plateau storage. *Adv. Energy Mater.* **15**, 2405294 (2025).
