# Supplementary material for: 900°C thermal-force coupling carbonization tailoring hard carbon microstructures enables high performance sodium-ion pouch batteries
Source: Nat Commun. 2026 Sep 8;17:10620. doi: 10.1038/s41467-026-77440-0 (PMC13650117; doi:10.1038/s41467-026-77440-0)
Supplement: Supplementary file 2 [file 41467_2026_77440_MOESM2_ESM.pdf]

Description of Additional Supplementary Files

Supplementary Data 1: The reported database of parameters of hard carbon for sodium-ion batteries.
