# Supplementary material for: 900°C thermal-force coupling carbonization tailoring hard carbon microstructures enables high performance sodium-ion pouch batteries
Source: Nat Commun. 2026 Sep 8;17:10620. doi: 10.1038/s41467-026-77440-0 (PMC13650117; doi:10.1038/s41467-026-77440-0)
Supplement: Supplementary file 3 [file 41467_2026_77440_MOESM4_ESM.pdf]

nature portfolio

Peer Review File

900°C Thermal-Force Coupling Carbonization Tailoring Hard Carbon Microstructures Enables High Performance Sodium-Ion Pouch Batteries

Corresponding Author: Professor Chunnian He

This file contains all reviewer reports in order by version, followed by all author rebuttals in order by version.

Version 0:

Reviewer comments:

Reviewer #1

(Remarks to the Author)
This is a comprehensive work from ML, experimental menchanistic research to feasibility assessment. I have few concerns reagrding ML and feasibity assessment part.
ML:
1. The manuscript state 0.761 and 0.758 are the R2 of training and test set for ICE and capacity. I think there should be four R2 instead only two?
2. What is the hyperparameters searching space? What tuning method was used?
3. The ideal parameters space seems quite broad. The only conclusion might be that the small SSA is preferable, but this is also proven by many previous research (ref: the closed pores hypothesis). Please motivate the ML a bit more by clarifying the contribution to this area.
4. It seems the model chosen in this study performs similar R2 0.6-0.76, did the author compare the shapley analysis between the models? Did they show the similar trend on feature importancce and partial contribution to the capacity and ICE? A comparison is recommended to enhance the robustness of the ML conclusion.

Feasibility assessment:
5. Figure5c, it is not clear that a total cost equal to CO2 emission+electrcity. What is the total cost, and what is the emission cost(carbon tax? where the emission comes from? LCA?), what is the electrcity cost (where is other component in the OPEX and CAPEX).

6. How is the 16.14 g of CO2 emissions calculated?
7. How did the author calculated the CAPEX and OPEX? Where the hourly CAPEX rate comes from, and what it included.
8. What is the scope of TEA and LCA? What was the standard LC Impact Method be used? For TEA did a discounted cash flow model has been used?

Reviewer #2

(Remarks to the Author)
This work presents desirable hard carbon structural parameters for high initial cycle efficiency (ICE) and reversible plateau capacity through machine learning, and a thermal-force coupling synthesis method was used to obtain hard cardon with optimized structure through low temperature heat treatment. Although machine learning provides the correlation between several key structural parameters with desired properties, the input parameters should be carefully selected. In this work, the input parameters are ID/IG, SSA, and d002, which are the structural parameters of synthesized hard carbon materials, were adopted. However, the correlations between these parameters and ICE or reversible plateau capacity have been shown in various works and are well known in the field. Furthermore, the suggested range for each parameter is rather wide, so the hard carbon structures could be significantly different even within the suggested range. As for the thermal-force coupling synthesis method, this work presents an alternative way to avoid the trade-off effect for temperature tuning, and the required temperature for achieving desirable hard carbon structure is significantly reduced, showing a possible way for hard carbon optimization and energy saving.

1. The authors mentioned that high ID/IG is beneficial for Na-ion diffusion. Is there direct correlation between high ID/IG and fast Na-ion diffusion?
2. How does C=O bond associate with graphene sheet curvature?
3. There is often a large drop in Na-ion diffusivity at the beginning of plateau capacity from GITT measurement. However, the thermal-force coupling synthesized samples barely show any drop at the beginning of plateau capacity, showing that the Na-ion kinetics is significantly improved in these samples. Is there any possible reason for this difference between this work and other works?
4. Figure 56 in the supporting information was used to support the statement of the appearance of quasi-metallic Na cluster. However, XPS is a very surface-limited detection. How does XPS present Na cluster formation which takes place in the bulk of hard carbon?
5. It could be expected that there would be more functional groups or impurities left in the thermal-force coupling synthesized hard carbon because of the low synthesis temperature, the applied pressure and the confinement of the mold. Is there a possibility that this could affect the performance of hard carbon?
6. NVP is usually recognized as a stable cathode material, and the half cell test for NVP in this work also shows nice stability. What is the reason for the degradation in the NVP/HC full cell?
7. The length of 1 timestep was not mentioned in the MD simulation method.

Reviewer #3

(Remarks to the Author)
What are the noteworthy results?

This work merge computational and experimental approaches to assist in solving a long standing problem with the performance and commercialization of sodium-ion batteries, which is to improve sodium storage sites and diffusion kinetics and thereby improve battery performance. The authors achieved this goal and could produce batteries with capacities as high as 1.47 Ah at a high energy density of 216.1 Wh/kg.

Will the work be of significance to the field and related fields?

Yes

How does it compare to the established literature?

It is an improvement of data previously obtained.

If the work is not original, please provide relevant references.

The work is new

Does the work support the conclusions and claims, or is additional evidence needed?

Yes, it supports

Are there any flaws in the data analysis, interpretation and conclusions?

Didn't see any discrepancies

Do these prohibit publication or require revision?

Yes, I recommend publication after addressing these revisions.

Is the methodology sound? Does the work meet the expected standards in your field?

Yes

Is there enough detail provided in the methods for the work to be reproduced?

Yes

Comments that need to be addressed.

1. For the paper to be suitable for a very high impact factor journal such as Nature Communications, the authors need to probe more into the findings and try to explain the observations. Explain how and why these results are obatined and how they match or differ from previous studies. For most parts of the work, results are reported without attempting to explain what accounted for the results. For example why did HC-10-900 give a better reversible capacity and rate performance than HC-0-900? How does pressure influence the material obtained and by extension the performance? This is just an example but try to dig more into all the claims made in the work and incorporate appropriate references to support the explanations.

2. Does morphology of the materials influence their properties in any way? In other studies it is found that morphology influences material properties as it relates to their ability to load and release drugs (https://pubs.acs.org/doi/10.1021/acsapm.4c01765) (https://pubs.rsc.org/en/content/articlelanding/2021/za/d5tb00289c/unauth).
Can this translate to morphology being able to influence the battery performance of the HC's obtained here?

3. The features used for machine learning (ML) analysis can be improved.The authors should include current density, crystallite size (La and Lc), annealing time, pore volume, and heating rate in the analysis as these features can also significantly affect sodium-ion battery performance.

4. For work of high impact as this one, the K-fold cross-validation should be improved. Instead of 5-fold, can you switch it to 10-fold for us to see how it affects the analysis? The 8:2 training:testing is standard, so is okay. But the validation can be improved to reduce bias.

5. Can you explain how you adjusted the ML parameters to improve accuracy of the findings?

6. Did the ranking obtained with SHAP match literature findings? Can you do feature ablation ranking also to confirm the SHAP results?

7. ML was primarily used in this work to identify high-performing microstructures. Since a lot of data is available from the literature, and looking at the scope and high impact of this journal, it will be useful to use ML to predict the reversible capacities of the HC's as well. If the predictions match the experimental data obtained, that can further authenticate the ML analysis. For more information on how to go about that, you can refer to this latest work. (https://pubs.rsc.org/en/content/articlehtml/2025/su/d5su00360a)

8. Can you expand on the chemistry of the synthesis or suggest a mechanism for the transition of glucose into the HC? Is it only water which is lost during pyrolysis? Can we know the chemical structure of the HC? The XPS of HC shows the presence of some functional groups. Can you check the XPS of as-made samples and samples at 300 degrees celcius also? So that there can be a direct comparison of the transition of the chemical composition along pyrolysis.

9. Carbonization yield of ~50% seems low for commercialization purposes of these materials. Can you suggest ways in which the yields can be improved? Can you do direct carbonization without pre-aromatization at 300 degrees celcius to see if results of their battery performance are similar? So we know if we can skip the intermediate step or not, or why that step is important. And also if that can improve the yield without affecting the performance.

10. The cost factor can also be improved by removing the conductive agent as shown in some works. (https://pubs.acs.org/doi/abs/10.1021/acsaem.4c01769). Can it apply in this case? Can you try to do one analysis with the best sample without incorporating the conductive agent and we compare the battery performance?

11.Calculate the degree of graphitization using the obtained interlayer spacing values to see what influence it has on the reversible capacities and ICE. For reference on how the calculations can be done, you can refer to (https://www.sciencedirect.com/science/article/pii/S2667056925001221).

12. Was the Raman data determined using the peak maxima values or the integrated peak intensities? The peak intensities give more accurate results since they are determined using the area under the curve rather than the peak height, and those values tend to be smaller. Can you make changes to reflect this if needed? The peak intesity fittings can be achieved in Origin.

Reviewer #4

(Remarks to the Author)
This manuscript reports a pressure-assisted carbonization strategy to prepare hard carbon anodes at 900 °C, which is claimed to simultaneously enable abundant sodium storage sites and fast Na^+ diffusion. The authors combine a literature-derived machine-learning analysis with experimental synthesis and demonstrate promising electrochemical performance, including an Ah-level pouch cell with a reported energy density of 216.1 Wh kg^{-1}. However, the claims relating to machine learning guided design, “thermal-force coupling” preparation, and sustainability appear to be overstated, and there are major issues to be addressed.

1. The practical scientific value of this ML component is unclear, and it provides very limited predictive insight and appears largely disconnected from the experimental strategy.
First, the dataset is assembled from heterogeneous literature sources with substantial inconsistencies and hidden variables. These factors introduce strong uncertainty that limit the reliability of ML correlations. The reported R2 value is only ~0.75, which is insufficient to support reliable conclusions.

Second, the ML results do not appear to generate new insights beyond understanding already known in the hard-carbon field. It does not provide predictive capability, but a region of structure parameters. How do the ML-derived structural design principles translate into processing parameters? The current ML and experimental sections function as two largely independent narratives rather than a truly integrated design framework.

2. The manuscript presents “thermal-force coupling carbonization” as a new preparation paradigm. However, the method essentially corresponds to pressure-assisted or hot-press carbonization. Similar approaches have been widely used in powder metallurgy, carbon materials processing, and hot-press sintering.
Moreover, it is not evident that this specific thermal–force coupling strategy is necessary. Indeed, the authors themselves note that many recent studies already report high-performance hard carbon materials within the proposed structural window but using various synthesis approaches.

3. This work repeated claim that high-temperature carbonization inevitably leads to strong trade-offs, while most comparisons are only made against HC-0-1300, which represents a relatively modest anode without optimization. However, recent advances in hard carbon synthesis—including transient temperature processing, flash carbonization, and Joule-heating-based synthesis—have demonstrated the ability to precisely engineer carbon microstructures within extremely short timescales while maintaining high energy efficiency. These approaches are not discussed or benchmarked in the manuscript.

4. When discussing about sustainability or energy consumption, the authors claims to lower the carbonization temperature from 1300 °C to 900 °C. However, this relies on high-pressure hot pressing, which itself requires additional energy input and costly specialized equipment. More importantly, hot pressing presents substantial challenges for industrial-scale production, particularly in terms of throughput and continuous processing. The TEA and economic analysis are significantly flawed by only considering 10-50 g material: Such data is not reliable for evaluating scale up feasibility and far from what the industry practiced.

5. While the pouch-cell performance is potentially promising, the origin and validity of the reported performance require further clarification. The detailed energy-density calculation should be defined, the cell-level parameters should be fully disclosed to enable fair comparison with previous reports, and to what extent the observed pouch-cell performance arises specifically from the proposed hard-carbon. Specifically, the ICE is only 87.4%. Was any prelithiation method used in pouch cell to compenstate? The areal density used (1.6 mg cm-2) may be lower than typical commercial Ah-level pouch cells (5-10 mg cm-2).

Minor comments
1. Maybe avoid using “an ideal microstructure”. How do you know it is ideal?
2. The introduction states only the initial stage of hard carbon research, without introducing recent progress and understanding. This raises many misinterpretations of their methods.
3. The authors state that microstructure features are coupled in the traditional thermal field, but independent in theirs. Such independent control is not supported by any evidence. Note: a different combination of feature parameters does not indicate independent control.
4. How do the authors perform MD with thermal and pressure fields to guide experiments?
5. Figure errors and grammar issues, such as “HC-0-90” should be HC-0-900, etc.

Version 1:

Reviewer comments:

Reviewer #1

(Remarks to the Author)
The revised ML part has been substantially improved, and I appreciate the authors’ efforts in this regard. The additional model comparison, validation have made this part much more transparent and complete. However, I remain concerned about the framing of the economic and environmental assessment. The authors have clarified that no standard LCIA method and no discounted cash-flow model were used; the analysis is basically a simplified laboratory-scale bottom-up cost and CO_2 accounting exercise. Therefore, I do not think it should be referred to as a formal LCA or TEA. Since this section is not the core contribution of the manuscript, I do not suggest to redo a complete LCA/TEA, but I strongly recommend that the authors should revise the terminology and tone down the related claims. Terms like CAPEX and OPEX should also be used with caution, because the current CAPEX is just an allocated equipment depreciation cost, while the OPEX-like term appears to be a simplified labor/overhead proxy rather than a complete operating expenditure. I would recommend authors to use a much more conservative framing style, such as “laboratory-scale cost and CO_2 emission assessment”, this would be more appropriate.

Reviewer #2

(Remarks to the Author)
My concerns have been well addressed.

Reviewer #3

(Remarks to the Author)
The manuscript has been significantly improved. I therefore recommend that it gets published in its current form.

Reviewer #4

(Remarks to the Author)
The authors have struggled to address the previous comments. This manuscript attempts to present a new paradigm for hard carbon anodes by combining Machine Learning (ML) with a "Thermal-Force Coupling" carbonization. While the electrochemical performance reported is high, the scientific novelty and contribution can be low. It becomes evident that the ML integration is redundant and the sustainability claims are largely unsubstantiated. The core contribution of this work is the synthesis (thermal+force field) of a hard carbon material with improved electrochemical performances. This advancement may not meet the high standards for publication in Nature Communications.

1. The authors' response to previous concerns regarding the ML component remains unsatisfactory.
While they have provided improved R2 values and model comparisons, they have failed to address the teleological flaw in their research design: this ML provides very limited predictive insight and appears largely disconnected from the experimental strategy. The "Target Zone" identified is already a well-established consensus in the field. Using ML to confirm what is already known adds technical complexity without providing new scientific insight. In addition, in its current form, the ML acts as a "black box" decorator rather than a functional discovery tool.

2. The "Force-Field" (pressure) is a new primary experimental variable, yet the authors have failed to move beyond a phenomenological description. There is a lack of understanding of how 10-100 MPa of pressure compensates for a 400C thermal gap. Without evidence of how pressure lowers the activation energy for carbon layer rearrangement or alters degassing kinetics, the manuscript remains a report of an experimental observation, not a study of a scientific mechanism.

3. The authors continue to emphasize highly specific energy (49.7%) and carbon reduction (47.5%) which are highly speculative. Quantifying "sustainability" to a 0.1% precision based on a laboratory pellet press is scientifically disingenuous and inappropriate. There are just too many factors that can easily jeopardize these idealized numbers.

Version 2:

Reviewer comments:

Reviewer #4

(Remarks to the Author)
I think the authors have taken the previous review comments seriously and have thoroughly upgraded the manuscript, notably by transforming phenomenological observations into deep, rigorous scientific insights.The work provides profound insights into the low-temperature synthesis of high-performance hard carbon anodes and now meets the high standards of Nature Communications. I fully support its publication.

Open Access This Peer Review File is licensed under a Creative Commons Attribution 4.0 International License, which permits use, sharing, adaptation, distribution and reproduction in any medium or format, as long as you give appropriate credit to the original author(s) and the source, provide a link to the Creative Commons license, and indicate if changes were made.
In cases where reviewers are anonymous, credit should be given to 'Anonymous Referee' and the source.
The images or other third party material in this Peer Review File are included in the article's Creative Commons license, unless indicated otherwise in a credit line to the material. If material is not included in the article's Creative Commons license and your intended use is not permitted by statutory regulation or exceeds the permitted use, you will need to obtain permission directly from the copyright holder.
To view a copy of this license, visit https://creativecommons.org/licenses/by/4.0/

Table of Contents

Reviewer #1 (Remarks to the Author) **1**
Comment #1 1
Comment #2 3
Comment #3 5
Comment #4 8
Comment #5 12
Comment #6 15
Comment #7 18
Comment #8 19
Reviewer #2 (Remarks to the Author) **21**
Comment #1 26
Comment #2 29
Comment #3 31
Comment #4 33
Comment #5 36
Comment #6 39
Comment #7 42
Reviewer #3 (Remarks to the Author) **44**
Comment #1 44
Comment #2 48
Comment #3 51
Comment #4 56
Comment #5 58
Comment #6 61
Comment #7 64
Comment #8 68
Comment #9 72
Comment #10 77
Comment #11 78
Comment #12 81
Reviewer #4 (Remarks to the Author) **84**
Comment #1 85
Comment #2 94
Comment #3 99
Comment #4 104
Comment #5 106
Comment #6 113
Comment #7 114
Comment #8 117
Comment #9 119
Comment #10 121

Point-by-Point Response

(Black type: Reviewer's comments; Blue type: Our response; Red type: Our revision)

Reviewer #1 (Remarks to the Author):

This is a comprehensive work from ML, experimental mechanistic research to feasibility assessment. I have a few concerns regarding ML and the feasibility assessment part.

Response: We sincerely thank the reviewer for their comprehensive summary and highly positive evaluation of our work. We have carefully considered all your suggested changes regarding ML and feasibility assessment and have incorporated them into the revised manuscript to further enhance its scientific rigor and clarity. Our detailed, point-by-point responses below.

Comment #1. The manuscript states that 0.761 and 0.758 are the R^2 of the training and test set for ICE and capacity. I think there should be four R^2 instead of only two?

Response: We greatly appreciate your professional suggestions regarding the coefficient of determination (R^2) for the training and test sets. Splitting data into training and test sets in machine learning (ML), and calculating R^2 for each set, is crucial for assessing the ability of the model to fit the data and generalize. Regarding the fact that the presentation of the ML results in the original manuscript is incomplete, making it impossible to distinguish between the expression of initial Coulombic efficiency (ICE) and reversible plateau capacity during the training and testing phases, respectively. In the revised version, we have supplemented the 5-fold cross-validation (CV) mean ± standard deviation, train-CV, and hold-out test metrics of the ICE and reversible plateau capacity, and added MAE and RMSE to provide a more comprehensive picture of the error magnitude and generalization ability of the model (**Table R1**).

Table R1. The R^2 in the training and test sets of the ICE and reversible plateau capacity.

Task	n	CV setting	CV mean R^2	Train-CV R^2	Test R^2	Test MAE	Test RMSE
ICE	565	5-fold	0.763 ± 0.019	0.764	0.826	0.257	0.311
Plateau capacity	565	5-fold	0.805 ± 0.041	0.806	0.794	26.93	33.40

In the ICE and reversible plateau capacity calculations, the data points were distributed around the reference line with a small error. For ICE, the 5-fold CV mean R^2 is 0.763 ± 0.019, and the train-CV R^2 is 0.764, the hold-out test R^2 is 0.826 (**Figure R1a**). For plateau capacity, the 5-fold CV mean R^2 is 0.805 ± 0.041, the train-CV R^2 is 0.806, and the hold-out test R^2 is 0.794 (**Figure R1b**). The ICE and reversible plateau capacity both exhibit high R^2 values on both the training and test sets, indicating that the Random Forest (RF) model possesses excellent generalization and predictive capabilities. Furthermore, the small difference between the R^2 values for the training set and the test set in the ICE and reversible plateau capacity calculations indicates the RF model is more robust. This provides a robust basis for elucidating the physical relationship between the microstructure of hard carbon (HC) and its ICE and reversible plateau capacity.

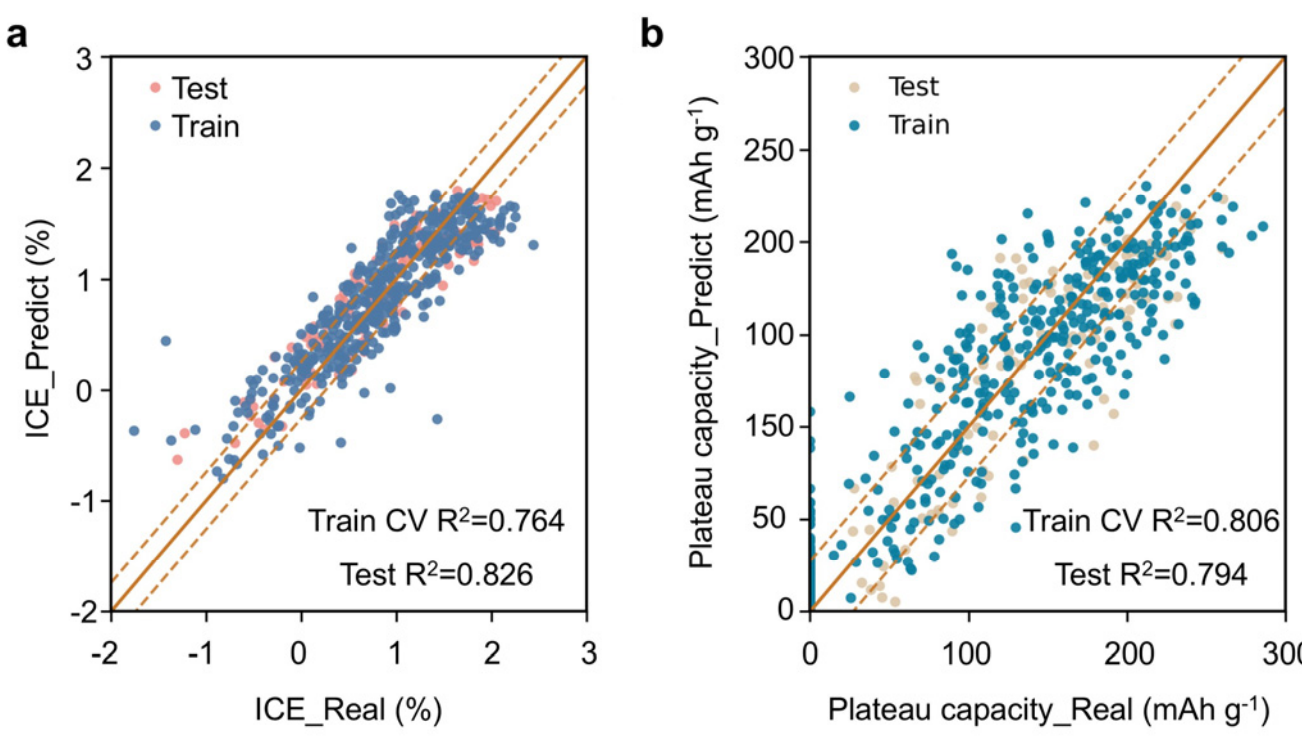

Figure R1. Performance of using the RF model on the training data of (a) ICE and (b) reversible plateau capacity.

Our revision:

1. Added detailed description.

p 7, line 132-136 (Results)

In calculations of ICE, the Random Forest (RF) model exhibits the highest 5-fold cross-validation mean R^2 (0.826), indicating strong regression performance (Supplementary Fig. 2). In calculations of reversible plateau capacity, the 5-fold cross-validation mean R^2 for RF (0.805) is slightly lower than that for eXtreme Gradient Boosting (XGB, 0.811), the hold-out test R^2 for RF (0.794) is higher than that for XGB (0.780), resulting in a smaller generalization gap (Table S1).

p 7, line 140-141 (Results)

The data points were distributed around the reference line in the target value calculations with a small error (Supplementary Fig. 4, Table S2).

2. Added figures and tables.

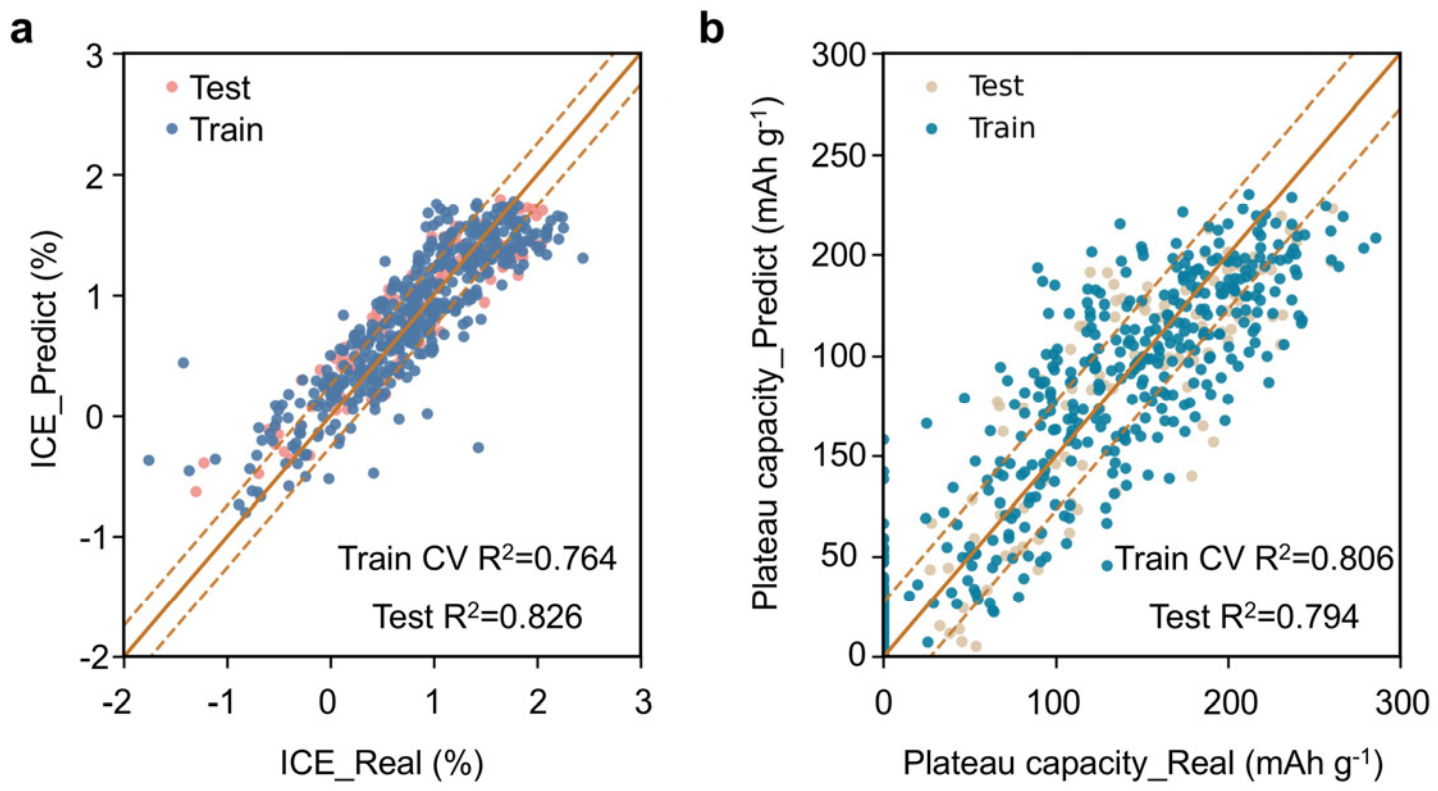

Supplementary Fig. 4. Performance of using the Random Forest (RF) model on the training data of (a) ICE and (b) reversible plateau capacity.

Supplementary Table 2. The R^2 of the training and test sets of the ICE and reversible plateau capacity in the RF model.

Task	n	CV setting	CV R^2	Train-CV R^2	Test R^2	Test MAE	Test RMSE	CV setting	CV R^2	Test R^2
ICE	565	5-fold	0.763	0.764	0.826	0.257	0.311	10-fold	0.767	0.826
Plateau capacity	565	5-fold	0.805	0.806	0.794	26.93	33.40	10-fold	0.805	0.797

Comment #2. What is the hyperparameter searching space? What tuning method was used?

Response: We sincerely appreciate the reviewers for pointing out the lack of transparency in ML methods. The hyperparameter search space refers to the range of a set of predefined parameter configurations that the model cannot automatically learn from the data during training. These parameters determine the structure of the model and learning strategy, directly influencing the physical relationship between the microstructure of HC and its ICE and reversible plateau capacity. In the hyperparameter search space, Bayesian optimization exhibits high efficiency, high robustness, and intelligence. This is attributed to the fact that it does not require any assumptions about the form of the objective function and automatically fits complex surfaces to find the global optimum. We have further integrated the ML workflow (**Figure R2**).

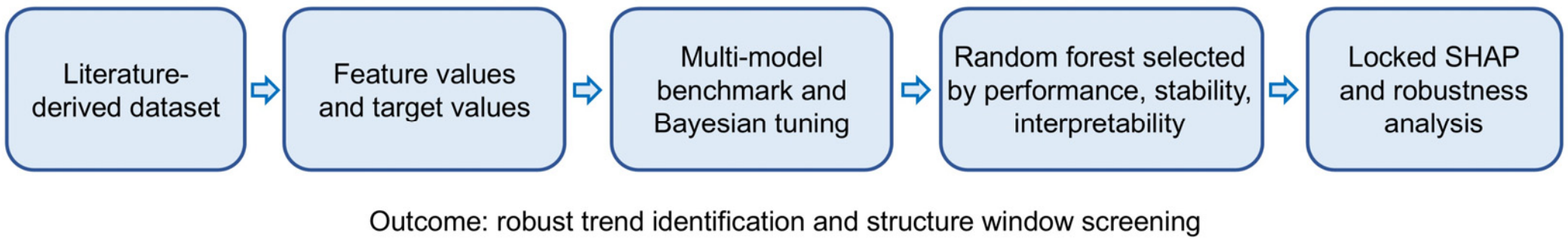

Figure R2. The workflow of ML.

In the ML algorithm calculations, seven regression models were evaluated for predictive analysis of HC, including Linear Regression, Gradient Boosted Regressor (GBR), Ridge Regression, Lasso Regression, Neural Network Regression, RF, and eXtreme Gradient Boosting (XGB). The data of the database were randomly divided into training and test sets in the ratio of 8:2. To improve model accuracy while preventing data leakage, hyperparameter optimization was performed exclusively on the training set using BayesSearchCV. The optimization objective was set to R^2, the random seed was fixed at 42, and the number of iterations was set to 20. During the model evaluation phase, a 5-fold CV was used as the primary framework, supplemented by a 10-fold CV to confirm that the results were insensitive to the number of validation folds and demonstrated robustness. In selecting the final model, RF presented the most favorable performance. Besides, the small difference in R^2 between the training and test sets indicates no overfitting for the RF model. More importantly, the RF model supports robust subsequent Shapley Additive exPlanations (SHAP) analysis, thereby addressing the need for cross-model interpretability. Based on this workflow, different RF models were developed for the two targets, and a preferred structure window that benefits both ICE and reversible plateau capacity was further identified through SHAP analysis.

Table R2. The hyperparameter search space for the main model.

**Model**	**Hyperparameter search space**
GBR (ICE)	n_estimators: 1–200; learning_rate: 0.01–1.0; max_depth: 1–10
GBR (plateau)	n_estimators: 1–100; learning_rate: 0.01–1.0; max_depth: 5–10
RF	n_estimators: 100–600; max_depth: 5–30; min_samples_split: 2–10; min_samples_leaf: 1–10; max_features: {sqrt, log2, None}; bootstrap: {True, False}
XGB	n_estimators: 1–150; max_depth: 5–10; learning_rate: 0.03–0.1 (log); subsample: 0.5–0.6; colsample_bytree: 0.6–1.0; reg_alpha / reg_lambda: 1e-5–1.0 (log)
Ridge	alpha: 0.01–10.0 (log)
Lasso	alpha: 0.01–10.0 (log)

Furthermore, we have summarized the hyperparameter search spaces for each model and provided the best parameters for the final RF model in terms of ICE and reversible plateau capacity, to facilitate evaluation of the hyperparameter tuning process and reproduction of the analysis (**Table R2, 3**).

Table R3. The best parameters for the final RF model.

Task	Final Random Forest best parameters
ICE	max_depth=30, max_features=log2, min_samples_leaf=1, min_samples_split=2, n_estimators=600, bootstrap=True
Plateau capacity	max_depth=30, max_features=sqrt, min_samples_leaf=1, min_samples_split=4, n_estimators=161, bootstrap=True

Our revision:

1. Added detailed description.

p 6, line 120-124 (Results)

In the ML algorithm calculations, the data of the database were randomly divided into training and test sets in the ratio of 8:2, and hyperparameter optimization was performed exclusively on the training set using BayesSearchCV[29]. The optimization objective was set to R^2, the random seed was fixed at 42, and the number of iterations was set to 20.

Comment #3. The ideal parameter space seems quite broad. The only conclusion might be that the small SSA is preferable, but this is also proven by many previous studies (ref: the closed pores hypothesis). Please motivate the ML a bit more by clarifying the contribution to this area.

Response: We fully agree with the view of reviewers that a low SSA has a more significant impact on the performance of HC. The existing literature has widely established a consensus that low SSA is beneficial for enhancing the electrochemical performance of HC, with the closed-pore hypothesis serving as the theoretical basis. The primary motivation for incorporating ML into this study is not to re-validate this known trend, but rather to address the challenges of qualitative ambiguity and strong parameter coupling in traditional experimental and theoretical analyses. This work aims to advance the design of HC materials from an empirical trial-and-error approach to data-driven quantitative prediction and multi-objective optimization.

Quantifying nonlinear thresholds: Traditional views often generally assume that the lower the SSA, the more pronounced the performance of HC, but they lack a precise definition of the critical threshold (***Adv. Energy Mater. 2024,*** 14, 19, 2303833). Training a nonlinear regression model using ML captures the complex nonlinear relationship between SSA and ICE, as well as reversible plateau capacity (**Figure R3**). When the SSA falls below a certain threshold, the increase in ICE tends to plateau, and further reductions in SSA may result in irreversible loss of micropore volume.

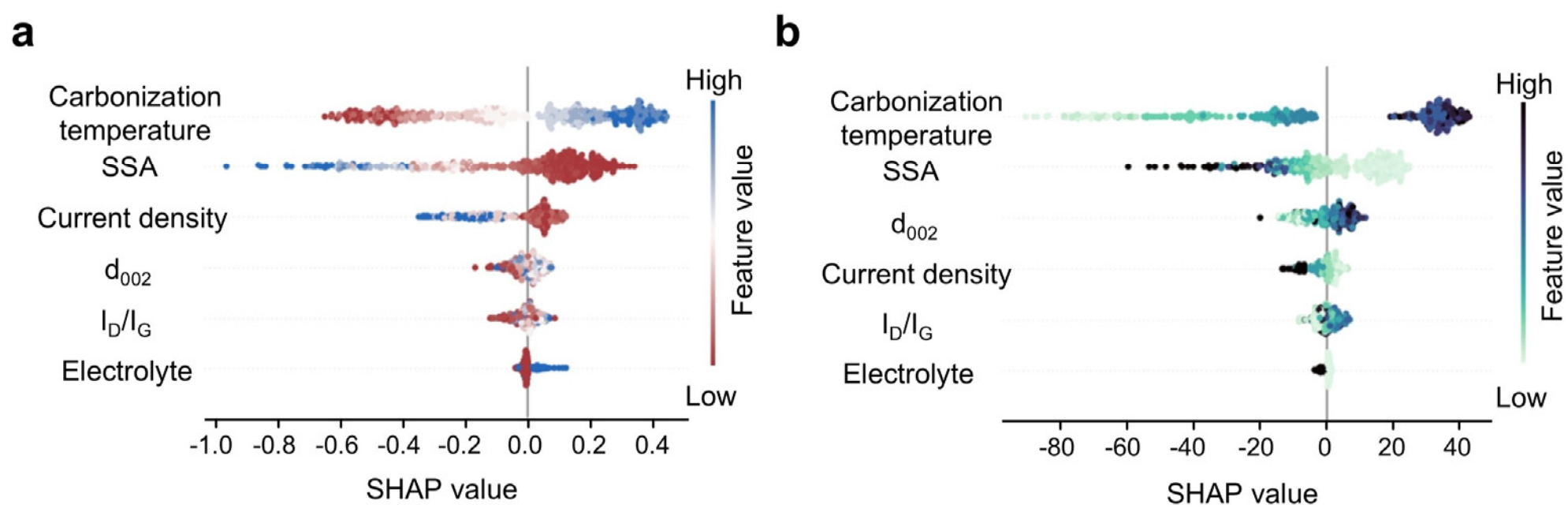

Figure R3. The SHAP analysis honeycomb diagram of (a) ICE and (b) reversible plateau capacity.

Decoupling strong coupling effects among multiple parameters: In the synthesis of HC, structure parameters (SSA, d_{002}, and I_D/I_G) are often highly correlated. Traditional univariate analysis struggles to disentangle these intertwined effects, leading to misinterpretations of the role of single factors (***Mater. Today 2019***, 23, 87-104). The ML model utilizes multiple regression to statistically control for other variables, thereby independently assessing the contribution of SSA to ICE and reversible plateau capacity. The SHAP analysis further reveals the synergistic mechanisms among the parameters (***Nat. Commun. 2025,*** 16, 7818). When d_{002} is in a wider range, the negative impact of SSA on ICE is partially offset. Conversely, in a narrower d_{002}, even a slight increase in SSA leads to a rapid decrease in ICE.

Finding the right balance between performance: The core challenge with HC lies in the inherent trade-off between ICE and high reversible capacity. Although low SSA improves the ICE of HC, it often comes at the expense of microporous sodium storage capacity. In this work (**Figure R4**), HC with high ICE generally possesses a d_{002} of 0.360-0.394 nm, an I_D/I_G of 1.10-2.36, and a SSA of 0.1-77.0 $m^2\ g^{-1}$. And HC with high reversible plateau capacity typically possesses a d_{002} of 0.376-0.420 nm, an I_D/I_G of 1.05-2.30, and a SSA of 0.26-17.84 $m^2\ g^{-1}$. Integrating these two structure parameter ranges yields a well-defined microstructure “target zone” characterized by low

SSA (0.26-17.84 m^2 g^{-1}), moderate d_{002} (0.376-0.394 nm), and moderate I_D/I_G (1.10-2.30).

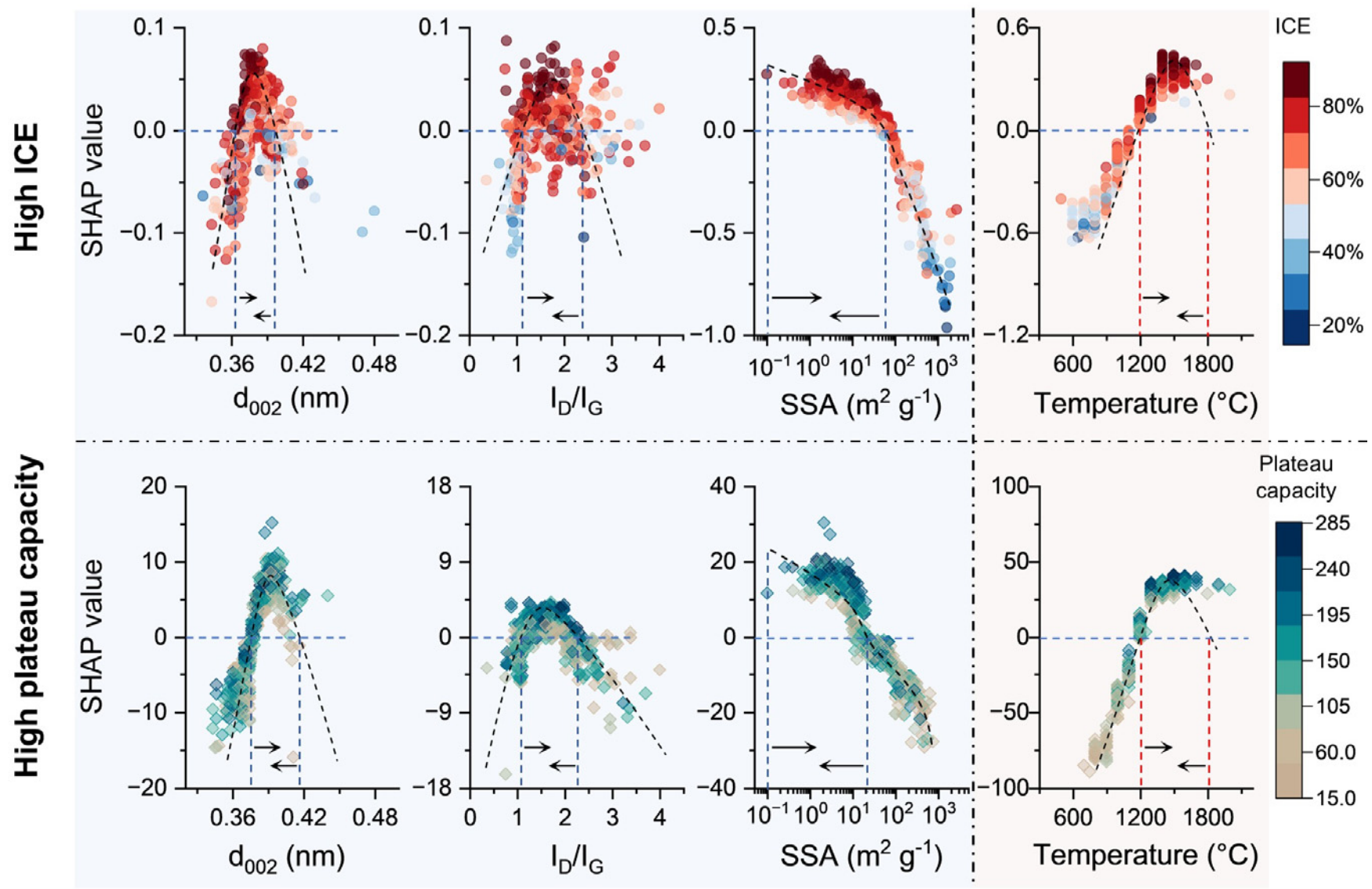

Figure R4. Relationship of ICE and reversible plateau capacity with each structure feature.

Therefore, the ML work in this study goes beyond simple trend recognition. The core value lies in transforming the empirical rules of HC material design into quantifiable design guidelines. The study provides a data-driven, rational design framework for developing high-performance HC materials by precisely defining critical thresholds for key parameters, decoupling complex structure-performance relationships, and establishing boundaries for multi-objective optimization.

Our revision:

1. Added detailed description.

p 5, line 82-84 (Introduction)

Furthermore, traditional research methods are unable to systematically elucidate the relationship between structure and performance due to the limited number of experiments and low efficiency.

p 5, line 84-86 (Introduction)

Although numerous studies have reached a certain consensus regarding the influence of structure on performance, the range of microstructure characteristics for high-performance HC remains undetermined[26].

p 5, line 86-88 (Introduction)

Based on data from current literature regarding processes, structures, and performance, machine learning (ML) uncovers complex nonlinear relationships behind vast variables, revealing the key microstructure influence rules within trade-offs to provide quantifiable optimal ranges[27,28].

p 8, line 143-145 (Results)

Compared to d_{002} and I_D/I_G, SSA exhibits a higher degree of influence on ICE and reversible plateau capacity, and HC with a low SSA is more likely to achieve high ICE and reversible plateau capacity (Supplementary Fig. 5, 6).

Comment #4. It seems the model chosen in this study performs similar R^2 of 0.6-0.76. Did the author compare the Shapley analysis between the models? Did they show a similar trend in feature importance and partial contribution to the capacity and ICE? A comparison is recommended to enhance the robustness of the ML conclusion.

Response: We deeply appreciate the reviewers for raising this critical issue regarding SHAP analysis across different models, which is essential for enhancing the robustness of ML conclusions. We fully agree that SHAP analysis of a single model is unlikely to support strong conclusions, given that different models exhibit similar predictive performance. To improve the accuracy and predictability of ML results, we have added a systematic comparison of various models, including Linear, Ridge, Lasso, GBR, XGB, Neural Network, and RF. When selecting the final model, we evaluate it based on R^2, predictive performance, CV stability, the generalization gap, and interpretability.

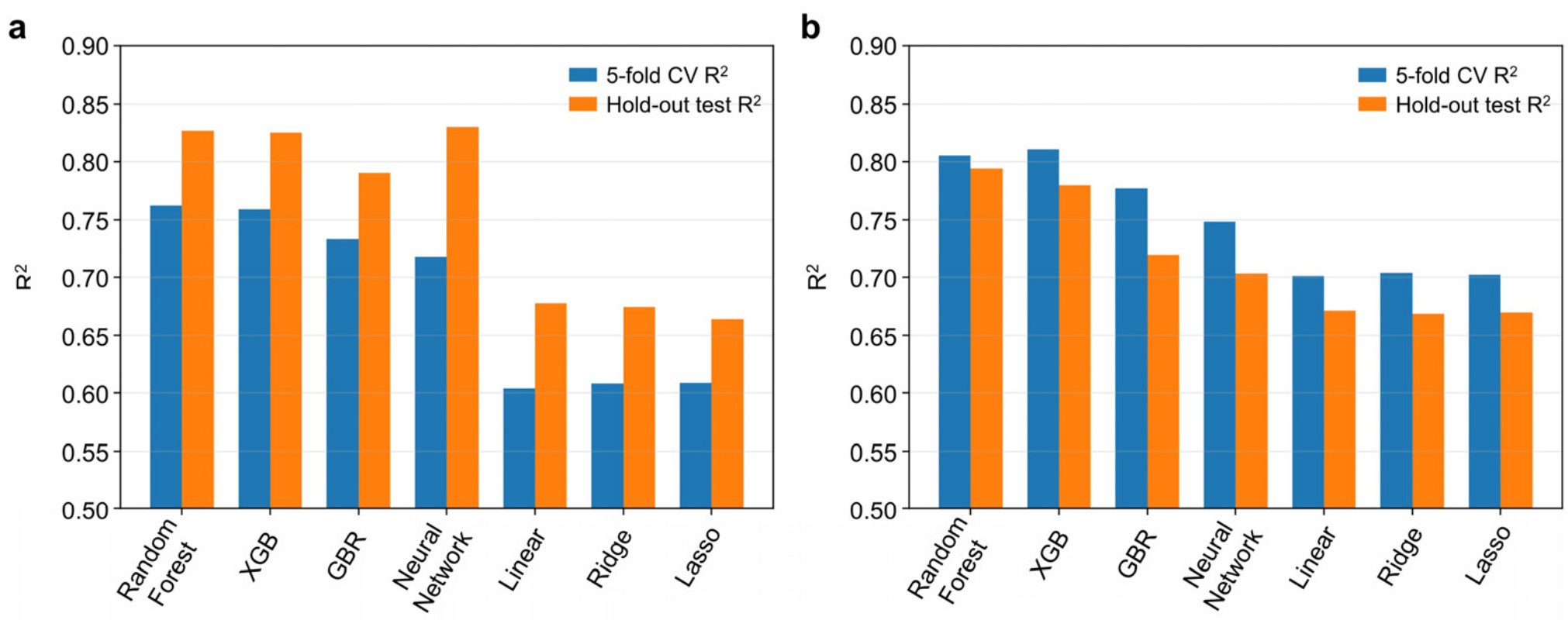

Figure R5. The performance comparison of multiple models in (a) ICE and (b) reversible plateau capacity.

In calculations of ICE, the 5-fold CV mean R^2 of RF, XGB, GBR, Neural Network, Linear, Ridge, and Lasso are 0.763, 0.759, 0.734, 0.718, 0.604, 0.608, and 0.608, respectively (**Figure R5a**). In contrast, the RF model exhibits the highest R^2 (0.826), indicating its strong regression performance.

Besides, the RF model achieves an R^2 on the training set that is comparable to that of the XGB (0.825) and Neural Network (0.830) models and higher than that of other models. In calculations of reversible plateau capacity, the 5-fold CV mean R^2 of RF, XGB, GBR, Neural Network, Linear, Ridge, and Lasso are 0.805, 0.811, 0.777, 0.749, 0.701, 0.704, and 0.702, respectively (**Figure R5b**). Although the CV mean R^2 for RF (0.805) is slightly lower than that for XGB (0.811), the hold-out test R^2 for RF (0.794) is higher than that for XGB (0.780), resulting in a smaller generalization gap. Therefore, after comprehensively evaluating the R^2 value, predictive performance, CV stability, generalization gap, and interpretability, the RF model was selected as the final model (**Table R4**).

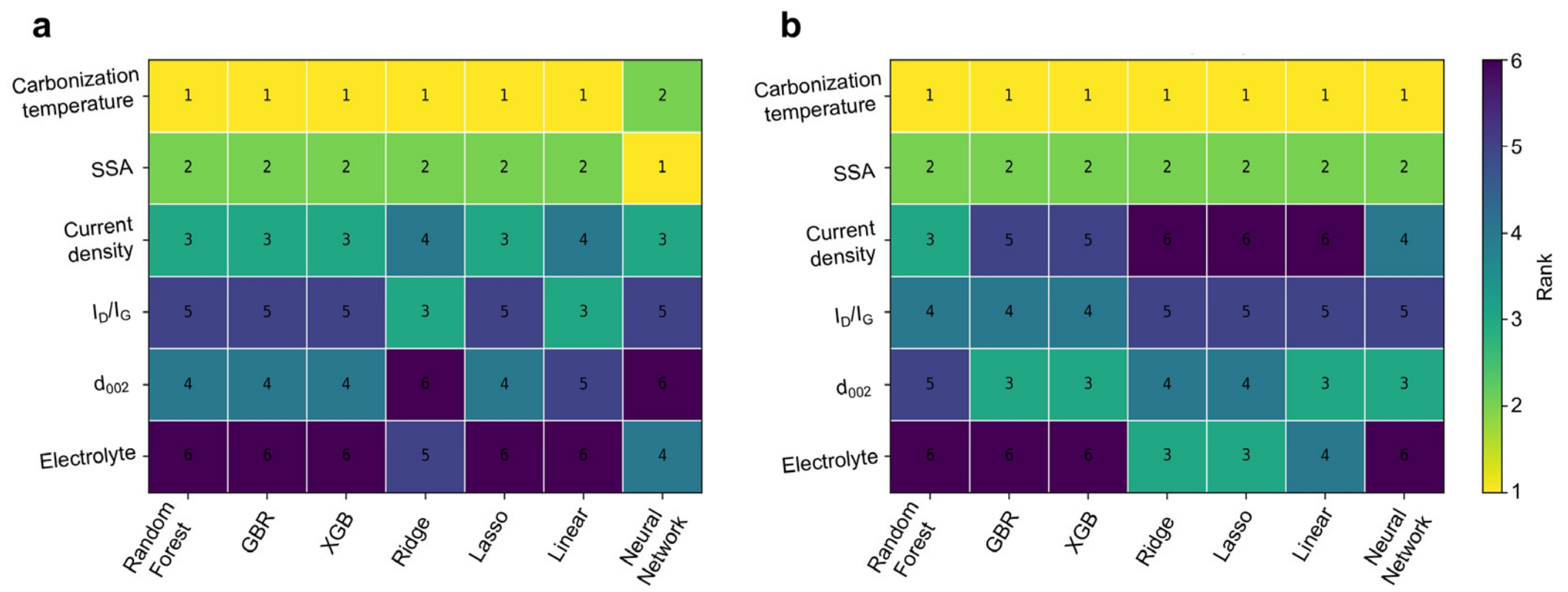

Figure R6. The heatmap of SHAP ranking consistency across different models in (a) ICE and (b) reversible plateau capacity.

We further conducted a cross-model SHAP ranking comparison of GBR, XGB, Ridge, and locked RF to analyze the contribution of each feature to the ICE and reversible plateau capacity, respectively. For ICE, the contributions of carbonization temperature and SSA consistently rank among the top factors across different models, while current density also remains at a consistently high level of importance (**Figure R6a**). For reversible plateau capacity, carbonization temperature, and SSA play a consistently dominant role across different models. Moreover, d_{002} and I_D/I_G both contribute moderately in different models for ICE and reversible plateau capacity (**Figure R6b**). Notably, the ranking of features by their contribution to ICE and reversible plateau capacity remains consistent in the RF model. Consequently, the conclusions drawn from machine learning demonstrate good robustness across multiple models, and the RF model stands out for its accuracy and predictive power.

Table R4. The performance, generalization gaps, and interpretability comparison of multi-models.

Task	Model	CV mean R^2	CV std	Test R^2	Generalization gap	SHAP analysis
ICE	Random Forest	0.763	0.019	0.826	-0.062	Yes
ICE	XGB	0.759	0.024	0.825	-0.062	Yes
ICE	GBR	0.734	0.037	0.790	-0.052	Yes
ICE	Neural Network	0.718	0.055	0.830	-0.106	No
ICE	Linear	0.604	0.131	0.678	-0.062	Yes
ICE	Ridge	0.608	0.136	0.674	-0.053	Yes
ICE	Lasso	0.608	0.147	0.664	-0.041	Yes
Plateau capacity	Random Forest	0.805	0.041	0.794	0.012	Yes
Plateau capacity	XGB	0.811	0.041	0.780	0.031	Yes
Plateau capacity	GBR	0.777	0.041	0.719	0.060	Yes
Plateau capacity	Neural Network	0.749	0.042	0.703	0.045	No
Plateau capacity	Linear	0.701	0.047	0.671	0.030	Yes
Plateau capacity	Ridge	0.704	0.047	0.668	0.036	Yes
Plateau capacity	Lasso	0.702	0.047	0.669	0.033	Yes

Our revision:

1. Added detailed description.

p 7, line 136-138 (Results)

Besides, the ranking of features by their contribution to ICE and reversible plateau capacity remains consistent in the RF model by a cross-model Shapley Additive exPlanations (SHAP) ranking comparison (Supplementary Fig. 3).

p 7, line 138-140 (Results)

Therefore, comprehensively evaluating the R^2 value, predictive performance, cross-validation stability, generalization gap, and interpretability, the RF model was selected as the final model.

2. Added figures and tables.

Supplementary Table 1. The performance, generalization gaps, and interpretability comparison of multi-models.

Task	Model	5-fold CV mean R^2	5-fold CV std	Test R^2	Generalization gap	SHAP analysis
ICE	Random Forest	0.763	0.019	0.826	-0.062	Yes
	XGB	0.759	0.024	0.825	-0.062	Yes
	GBR	0.734	0.037	0.790	-0.052	Yes
	Neural Network	0.718	0.055	0.830	-0.106	No
	Linear	0.604	0.131	0.678	-0.062	Yes
	Ridge	0.608	0.136	0.674	-0.053	Yes
	Lasso	0.608	0.147	0.664	-0.041	Yes
Plateau capacity	Random Forest	0.805	0.041	0.794	0.012	Yes
	XGB	0.811	0.041	0.780	0.031	Yes
	GBR	0.777	0.041	0.719	0.060	Yes
	Neural Network	0.749	0.042	0.703	0.045	No
	Linear	0.701	0.047	0.671	0.030	Yes
	Ridge	0.704	0.047	0.668	0.036	Yes
	Lasso	0.702	0.047	0.669	0.033	Yes

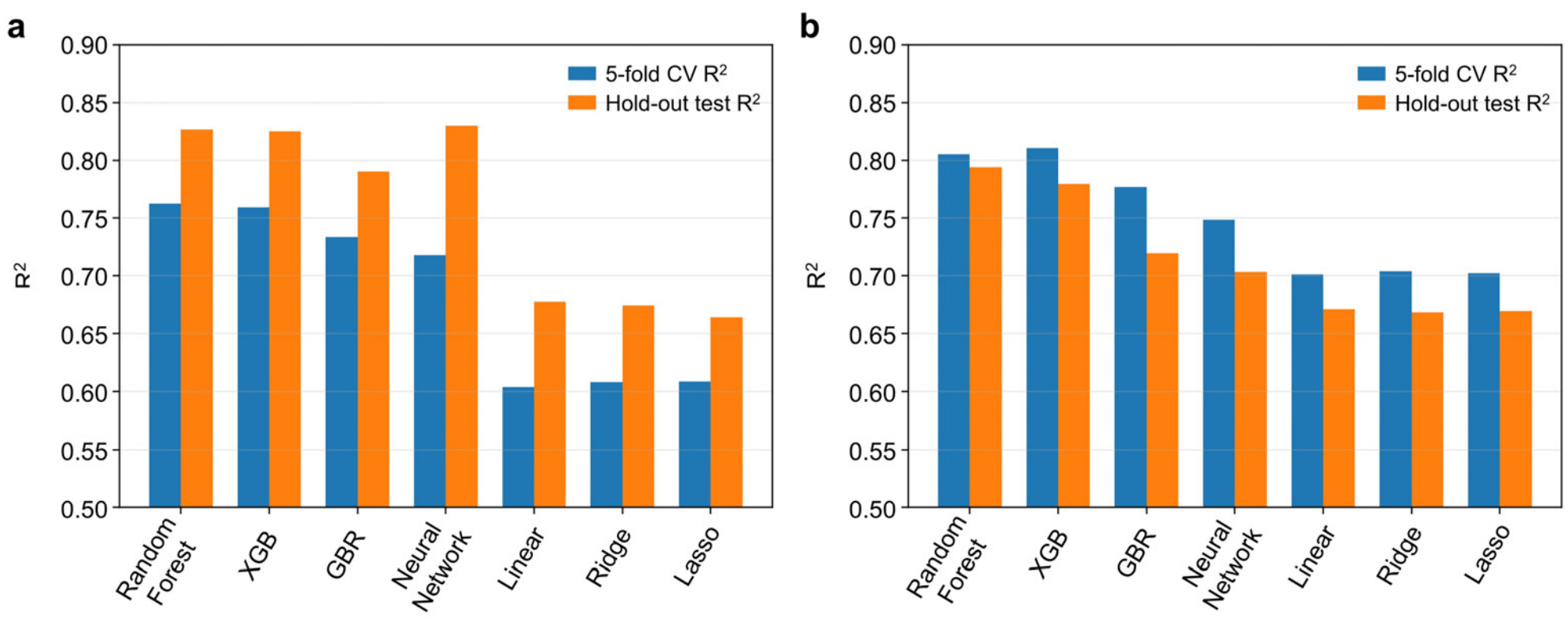

Supplementary Fig. 2. 5-fold cross-validation (CV) R^2 score of different regression models in (a) initial Coulombic efficiency (ICE) and (b) reversible plateau capacity.

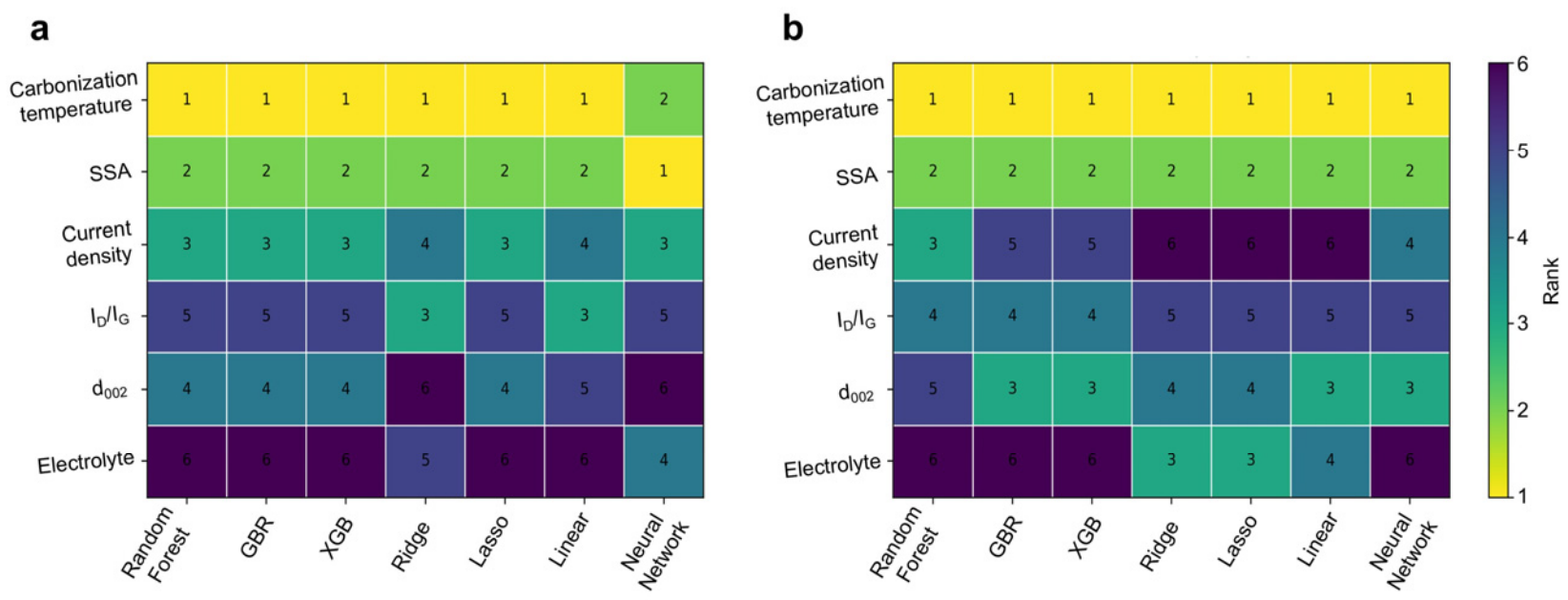

Supplementary Fig. 3. The heatmap of Shapley Additive exPlanations (SHAP) ranking consistency across different models in (a) ICE and (b) reversible plateau capacity.

Comment #5. Figure 5c, it is not clear whether the total cost equals CO_2 emission + electricity. What is the total cost, and what is the emission cost (carbon tax? where the emission comes from? LCA?), what is the electricity cost (where is the other component in the OPEX and CAPEX)?

Response: Thank you for your professional reminder, sincerely. In a feasibility assessment, the total production cost of HC includes private costs and social costs. Private costs primarily consist of feedstock, CAPEX, OPEX, and electricity. Social costs primarily refer to carbon taxes, which are taxes levied on carbon dioxide (CO_2) emissions. CO_2 emissions consist of two components: CO_2 generated from the decomposition of materials during the HC production process, which is determined through experimental testing. And CO_2 emissions resulting from electricity consumption during that process, the carbon intensity is 0.5 $kgCO_2/kWh$.

Feedstock price: The price of glucose is 4 $/kg.

CAPEX: refers to equipment costs. The equipment prices of low-temperature thermal field carbonization (LTC), high-temperature thermal field carbonization (HTC), and low-temperature thermal-force coupling carbonization (LTFC) are 1428.6 $, 4285.7 $, and 2142.9 $, respectively. Assuming the equipment operates normally for 15 years, the hourly equipment costs are calculated to be 0.01087 $/h, 0.03262 $/h, and 0.01631 $/h, respectively.

OPEX: refers to labor expenses, which account for 5% of CAPEX. The labor expenses of LTC, HTC, and LTFC are 0.5435×10^{-3} $/h, 1.6310×10^{-3} $/h, and 0.8155×10^{-3} $/h, respectively.

Electricity: refers to the electricity consumed during the HC production process, which is recorded in real time by an electricity meter, and the electricity price is 0.088 $/kWh.

Carbon taxes: refer to a charge levied on all CO_2 emissions, and the international carbon tax rate is 0.2 $/kgCO_2$.

Table R5. Cost of preparing HC-0-900, HC-0-1300, and HC-10-900 using 100g feedstock.

-	Composition	HC-0-900	HC-10-900	HC-0-1300
Precursor	$C_6H_{12}O_6$ (g)	100	100	100
Private costs	Feedstock price ($)	0.4	0.4	0.4
	CAPEX ($)	0.40	0.59	1.85
	OPEX ($)	0.02	0.03	0.09
	Electricity ($)	6.68	9.64	19.00
Social costs	CO_2 emissions of electricity ($)	7.59	10.95	21.59
	CO_2 emissions of materials ($)	0.018	0.014	0.023
Total cost	Private and social costs ($)	15.11	21.62	42.95
HC	Yield (g)	18.96	22.92	10.45

Table R6. The cost per kWh required for the full battery systems assembled in HC.

-	Composition	HC-0-900	HC-10-900	HC-0-1300
Private costs	Feedstock price ($/kg HC)	21.10	17.46	38.26
	CAPEX ($/kg HC)	21.22	25.86	176.79
	OPEX ($/kg HC)	1.06	1.29	8.84
	Electricity ($/kg HC)	352.46	420.58	1817.51
Social costs	CO_2 emissions ($/kg HC)	401.46	478.53	2067.55
Total cost	Private and social costs ($/kg HC)	797.31	943.72	4108.95
Full battery	Total energy (kWh/kg HC)	21.47	102.50	22.30
Cost per kWh	CAPEX ($/kWh)	0.99	0.25	7.93
	Electricity ($/kWh)	16.42	4.10	81.50
	CO_2 emissions ($/kWh)	18.70	4.67	92.72
	Total cost ($/kWh)	37.14	9.21	184.26

The total cost (private and social cost) of producing HC from 100 g of glucose via processes LTC, HTC, and LTFC is 15.11 $, 42.95 $, and 21.62 $, respectively (**Table R5**). The costs of feedstock, CAPEX, OPEX, electricity, and carbon taxes in HC-0-900 are 0.40 $, 0.40 $, 0.02 $, 6.68 $, and 7.61 $, respectively. The costs of feedstock, CAPEX, OPEX, electricity, and carbon taxes in HC-0-1300 are 0.40 $, 1.85 $, 0.09 $, 19.00 $, and 21.62 $, respectively. The costs of feedstock, CAPEX, OPEX, electricity, and carbon taxes in HC-10-900 are 0.40 $, 0.59 $, 0.03 $, 9.64 $, and 10.96 $, respectively. The carbon output mass of producing HC-0-900, HC-0-1300, and HC-10-900 from 100 g of glucose is 18.96 g, 10.45 g, and 22.92g, respectively. Based on calculations, the total cost of producing one kilogram of HC-0-900, HC-0-1300, and HC-10-900 is 943.72 $, 4108.95 $, and 797.31 $, respectively. The electricity cost of producing one kilogram of HC-0-900, HC-0-1300,

and HC-10-900 is 352.46 $, 1817.51 $, and 420.58 $, respectively. The carbon taxes of producing one kilogram of HC-0-900, HC-0-1300, and HC-10-900 are 401.46 $, 2067.55 $, and 478.53 $, respectively. The energy of one kilogram of HC-0-900, HC-0-1300, and HC-10-900 is 4.95 kWh, 4.51 kWh, and 129.35 kWh, respectively. Therefore, the per kWh carbon taxes, electricity cost, and total cost for HC-10-900 are only 4.7 $, 4.1 $, and 9.2 $, respectively (**Table R6**).

Our revision:

1. Added detailed description.

p 19, line 393-396 (Results)

In the comprehensive evaluation of three different pathways, the primary private costs of HC production include feedstock, capital expenditures (CAPEX), labor and overhead, and electricity expenses. We also include the carbon taxes of carbon emissions to capture the societal cost of HC production (energy consumption and process emissions).

p 19, line 396-398 (Results)

The total cost (private and social cost) of producing HC from 100 g of glucose via processes LTC, HTC, and LTFC is 15.11 $, 42.95 $, and 21.62 $, respectively (Fig. 5b, Table S11).

p 19, line 400-403 (Results)

As shown in Figure 5c, the per kWh social cost of carbon, electricity cost, and total cost for HC-10-900 are only 4.7 $, 4.1 $, and 9.2 $, respectively, significantly lower than those for HC-0-900 and HC-0-1300 due to the higher HC output and performance (Supplementary Fig. 64, Table S12).

2. Added tables.

Supplementary Table 11. Cost of preparing HC using 100g feedstock.

-	Composition	HC-0-900	HC-10-900	HC-0-1300
Precursor	$C_6H_{12}O_6$ (g)	100	100	100
Private costs	Feedstock price ($)	0.4	0.4	0.4
	CAPEX ($)	0.40	0.59	1.85
	OPEX ($)	0.02	0.03	0.09
	Electricity ($)	6.68	9.64	19.00
Social costs	CO_2 emissions of electricity ($)	7.59	10.95	21.59
	CO_2 emissions of materials ($)	0.018	0.014	0.023
Total cost	Private and social costs ($)	15.11	21.62	42.95
HC	Yield (g)	18.96	22.92	10.45

Supplementary Table 12. The cost per kWh required for the full battery systems assembled in HC.

-	Composition	HC-0-900	HC-10-900	HC-0-1300
Private costs	Feedstock price ($/kg HC)	21.10	17.46	38.26
	CAPEX ($/kg HC)	21.22	25.86	176.79
	OPEX ($/kg HC)	1.06	1.29	8.84
Social costs	Electricity ($/kg HC)	352.46	420.58	1817.51
	CO_2 emissions ($/kg HC)	401.46	478.53	2067.55
Total cost	Private and social costs ($/kg HC)	797.31	943.72	4108.95
Full battery	Total energy (kWh/kg HC)	21.47	102.50	22.30
Cost per kWh	CAPEX ($/kWh)	0.99	0.25	7.93
	Electricity ($/kWh)	16.42	4.10	81.50
	CO_2 emissions ($/kWh)	18.70	4.67	92.72
	Total cost ($/kWh)	37.14	9.21	184.26

Comment #6. How are the 16.14 g of CO_2 emissions calculated?

Response: We greatly appreciate the insightful questions raised by the reviewers regarding CO_2 emissions. In the carbon emission calculations, we assume that the carbon-containing gases produced during the pre-carbonization and high-temperature carbonization stages consist solely of CO_2 (***Adv. Energy Mater. 2022,*** 12, 18, 2200208; ***Adv. Sustainable Syst. 2022,*** 6, 6, 2200047; ***Phil. Trans. R. Soc. A 2021,*** 379, 2209, 20200340). Determine the mass percentage of the C element in pre-carbonized and high-temperature carbonized products by complete combustion using a carbon-sulfur analyzer. Calculating CO_2 emissions for the pre-carbonization and high-temperature carbonization stages based on the constant carbon content.

Pre-carbonization stage: $C_6H_{12}O_6 \rightarrow C_1 + CO_2$

High-temperature carbonization stage: $C_1 \rightarrow C_2 + CO_2$

During the pre-carbonization stage, the mass fraction of the C element in the pre-carbonization product was 67.5%, and the yield of the pre-carbonization product was 52.8% (**Table R7**). Calculations show that 100 g of glucose yields 52.8 g of pre-carbonization products and releases 16.14 g of CO_2 gas. The mass fraction of the C element in the HC obtained from 8 g of pre-carbonized material subjected to low-temperature thermal field carbonization, low-temperature thermal-force coupling carbonization, and high-temperature thermal field carbonization was 82.75%, 93.50%, and 82.50%, respectively, while the yields of HC were 35.9%, 43.4%, and 19.8%, respectively. Calculations show that 8 g of pre-carbonized material produces 11.06 g, 7.87 g, and

14.98 g of CO_2 gas, respectively, during low-temperature thermal field carbonization, low-temperature thermal-force coupling carbonization, and high-temperature thermal field carbonization.

Table R7. The CO_2 emissions from products generated during the pre-carbonization and high-temperature carbonization stages.

Process	Parameters	HC-0-900	HC-10-900	HC-0-1300
Pre-carbonization stage	$C_6H_{12}O_6$ (g)	100	100	100
	Carbon yield (g)	52.8	52.8	52.8
	Mass fraction of C	67.5%	67.5%	67.5%
	CO_2 emission	16.14	16.14	16.14
High-temperature carbonization stage	Pre-carbonization product (g)	8	8	8
	Carbon yield (g)	2.872	3.472	1.584
	Mass fraction of C	82.75%	93.50%	82.50%
	CO_2 emission	11.06	7.87	14.98

Our revision:

1. Added detailed description.

p 18, line 375-377 (Results)

In the carbon emissions calculation, it is assumed that the carbon-containing gases produced during the pre-carbonization and high-temperature carbonization stages consist solely of CO_2.

p 22, line 451-452 (Methodology)

The mass percentage of the C element of HC was determined by using a carbon-sulfur analyzer (CS844, LECO).

2. Added tables.

Supplementary Table 9. Parameter assumptions for the techno-economic assessment.

Process	Variables	Unit	Data source	HC-10-900	HC-0-1300	HC-0-900
Full battery	Anode mass	mg	Experiment	3.23	4.88	3.57
Full battery	Cathode mass	mg	Experiment	2.99	4.4	9.28
Full battery	C1_full battery	Wh	Experiment	0.002174	0.002585	0.001586
Full battery	C80_capacity	Wh	Experiment	0.001955	0.001749	0.001127

Full battery	R80	--	Experiment	0.8906	0.6517	0.7122
Full battery	N/P	--	Experiment	1.08	1.11	1.06
Half battery	C1_half_battery	Wh	Experiment	0.000159	0.000182	0.000107
Half battery	C40_half_battery	Wh	Experiment	0.000158	0.000155	0.000088
Half battery	R40	--	Experiment	0.9937	0.8517	0.8224
Half battery	Anode mass	mg	Experiment	1.56	2.06	0.91
Pre-carbonization	Pre-carbonization Energy	kWh/1kg feedstock	Experiment	1.72	1.72	1.72
Pre-carbonization	Pre-carbonization output ratio	--	Experiment	0.528	0.528	0.528
Pre-carbonization	Mass fraction of C in pre-treated feed	%	Experiment	67.5	67.5	67.5
Pre-carbonization	Pre-carbonization CO_2 emissions	g/100g feed	Experiment	16.14	16.14	16.14
Pre-carbonization	Hours run	--	Experiment	2	2	2
Pyrolysis	HC mass per 8g pre-treated feed	g	Experiment	3.472	1.584	2.872
Pyrolysis	Mass fraction of C in HC	%	Experiment	93.5	82.50	82.75
Pyrolysis	Pyrolysis CO_2 emission	g/8g feed	Experiment	7.87	14.98	11.06
Pyrolysis	Pyrolysis Energy	kWh/8g feedstock	Experiment	15.4	30.6	10.6
Pyrolysis	Hard Carbon output ratio	--	Experiment	0.0787	0.1498	0.1106
Pyrolysis	Hours run	--	Experiment	5	8	5
	Electricity price	$/kWh	Assumption	0.088	0.088	0.088
	Carbon intensity	$kgCO_2/kWh$	Assumption	0.5	0.5	0.5
	SCC_China	$\$/kgCO_2$	Assumption	0.015	0.015	0.015
	SCC_Global	$\$/kgCO_2$	Assumption	0.2	0.2	0.2
	Hourly CAPEX	$/hr	Assumption	0.016	0.033	0.011
	Labor & overhead (% of CAPEX)	--	Assumption	0.05	0.05	0.05
	Feedstock price	$/kg	Assumption	4	4	4

Comment #7. How did the author calculate the CAPEX and OPEX? Where the hourly CAPEX rate comes from, and what it includes.

Response: We thank the reviewer for raising this important point. The hourly CAPEX rate is derived from the equipment purchase cost using straight-line depreciation over a 15-year useful life, assuming continuous (24/7) operation:

$$\text{Hourly CAPEX} = (\text{Equipment cost in RMB} / 7) / (15 \times 8760)$$

Pre-carbonization and pyrolysis use separate equipment. Pre-carbonization uses a standard muffle furnace (10,000 RMB) for all scenarios. Pyrolysis equipment differs by pathway (**Table R8**):

Table R8. The equipment purchases cost and the hourly CAPEX rate of the three production technology pathways.

Scenario	Pyrolysis equipment	Cost (RMB)	Hourly rate ($/hr)
HC-10-900 (LTFC)	Muffle furnace + graphite mold + alloy press blocks	15,000	0.016
HC-0-1300 (HTC)	Muffle furnace for 1300°C	30,000	0.033
HC-0-900 (LTC)	Standard muffle furnace	10,000	0.011

The total CAPEX for each scenario is then calculated by evenly attributing the equipment cost to the process based on the number of hours the process uses — that is, the hourly depreciation rate is multiplied by the total processing time:

$$\text{Total CAPEX} = \text{pre-carbonization rate} \times h\_pre + \text{pyrolysis rate} \times h\_pyro \times B,$$

where B = [pre-carbonization output / 8 g] = 7 batches per 100 g glucose. OPEX consists of electricity (measured energy × $0.088/kWh), labor & overhead (5% of CAPEX), and feedstock ($4/kg glucose). Electricity dominates the private cost (~89–90%) due to the energy-intensive laboratory-scale batch processing.

Our revision:

1. Added detailed description.

p 25, line 549-553 (Methodology)

The hourly CAPEX rate is obtained by straight-line depreciation of equipment cost over a 15-year useful life with continuous operation (8,760 h/yr), converted at 7 RMB/USD. Pre-carbonization uses a standard muffle furnace (10,000 RMB) for all pathways. Pyrolysis equipment costs are 15,000 RMB for LTFC (muffle furnace with graphite mold and alloy press), 30,000 RMB for HTC (high-temperature muffle furnace), and 10,000 RMB for LTC (standard muffle furnace).

Comment #8. What is the scope of TEA and LCA? What was the standard LC Impact Method used? For TEA, has a discounted cash flow model been used?

Response: We thank the reviewer for this important methodological question. Our analysis is a comparative techno-economic assessment (TEA) with environmental cost internalization, rather than a comprehensive life cycle assessment (LCA). The system boundary is cradle-to-gate: from glucose feedstock through pre-carbonization and pyrolysis to the HC product. We additionally compute a levelized cost per kWh that connects production cost to battery performance (cycle life to 80% capacity retention).

We do not employ a standard LCA impact method (e.g., ReCiPe, CML). Our environmental assessment focuses on the climate change category: we quantify CO_2 emissions from electricity consumption and process reactions, then monetize them via the Social Cost of Carbon (SCC) at \$0.015/kgCO_2 (China) and \$0.20/kgCO_2 (global). This provides a direct monetary comparison of environmental externalities alongside private costs.

We use bottom-up cost accounting rather than a discounted cash flow (DCF) model. The purpose is comparative benchmarking of three lab-scale processes, not investment appraisal. All costs are in present-value terms. A full LCA and DCF-based TEA would be natural next steps at pilot scale.

Our revision:

1. Added detailed description.

p 18, line 370-373 (Results)

To compare the LTC, high-temperature thermal field carbonization (HTC), and LTFC, we conducted a cradle-to-gate techno-economic assessment (TEA) with environmental cost internalization. Private costs are computed using bottom-up cost accounting, and CO_2 emissions are monetized via the Social Cost of Carbon (SCC). A levelized cost per kWh links production cost to battery performance.

p 25, line 529-531 (Methodology)

Our analysis uses a bottom-up cost accounting framework with a cradle-to-gate system boundary. We do not employ a discounted cash flow model or standard LCA impact methods (e.g., ReCiPe). Environmental impact is limited to the climate change category, monetized through the SCC.

We sincerely thank you for your constructive comments and careful evaluation of our work. We hope that the substantial revisions and new analyses provided in this response satisfactorily address all of your concerns.

Point-by-Point Response

(Black type: Reviewer's comments; Blue type: Our response; Red type: Our revision)

Reviewer #2 (Remarks to the Author):

This work presents desirable hard carbon structural parameters for high initial cycle efficiency (ICE) and reversible plateau capacity through machine learning, and a thermal-force coupling synthesis method was used to obtain hard carbon with an optimized structure through low-temperature heat treatment. Although machine learning provides the correlation between several key structural parameters with desired properties, the input parameters should be carefully selected. In this work, the input parameters are I_D/I_G, SSA, and d_{002}, which are the structural parameters of synthesized hard carbon materials, and were adopted. However, the correlations between these parameters and ICE or reversible plateau capacity have been shown in various works and are well known in the field. Furthermore, the suggested range for each parameter is rather wide, so the hard carbon structures could be significantly different even within the suggested range. As for the thermal-force coupling synthesis method, this work presents an alternative way to avoid the trade-off effect for temperature tuning, and the required temperature for achieving a desirable hard carbon structure is significantly reduced, showing a possible way for hard carbon optimization and energy saving.

Response: We sincerely thank the reviewer for their positive evaluation and for recognizing the significant value of our work to energy conservation and emission reduction. We also sincerely appreciate the decision and constructive comments you have provided on our manuscript. In response, we have supplemented the manuscript and Supplementary Information with a variety of characterizations and tests. We believe that these additions have enhanced the overall scientific rigor of the manuscript. Below, we discuss the specific suggestions you raised and provide point-by-point explanations.

(1) Although machine learning provides the correlation between several key structural parameters with desired properties, the input parameters should be carefully selected.

Response: We are very grateful for the insightful questions of reviewers regarding the selection of input parameters. Hard carbon (HC) exhibits a rich and complex microstructure. In particular, the

interplay of various microstructure features during the carbonization process makes it difficult to control the preparation, which has long been a key factor limiting improvements in its performance. Uncovering the intrinsic relationships among the process, structure, and performance of HC is crucial to overcoming the aforementioned constraints. In this work, carbonization time and heating rate have been added to the process parameters; medium basal coherence length (L_a), crystal size in the stacking direction (L_c), and total pore volume (V_{total}) have been added to the structure parameters, and reversible capacity has been supplemented in the performance parameters. Screen out highly correlated feature values and target values through correlation analysis between variables to improve the accuracy of machine learning (ML, **Figure R1**).

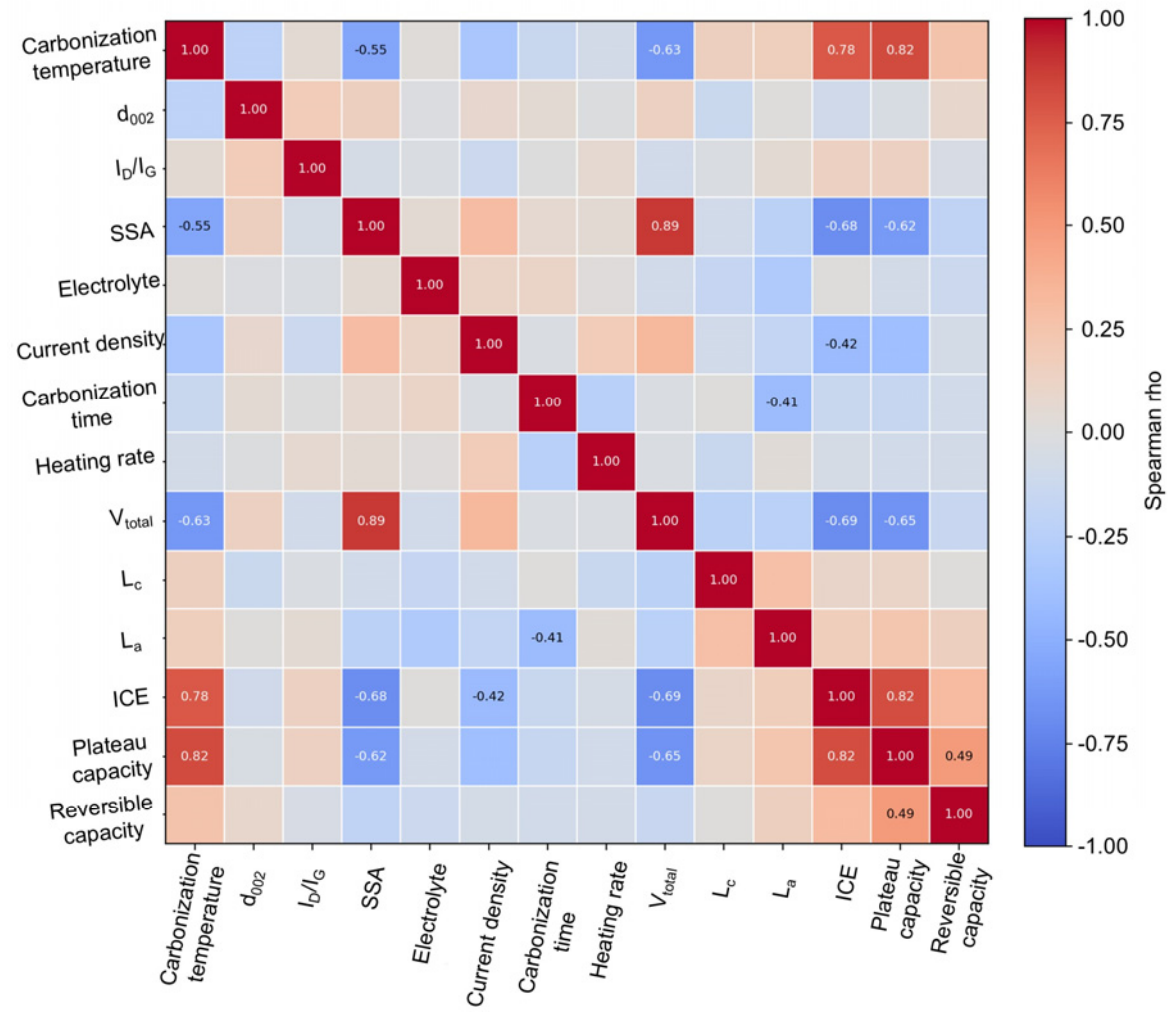

Figure R1. Spearman correlation heatmap between core features, missing variables, and the target variable.

Compared to the carbonization temperature, the carbonization time and heating rate did not show a sufficiently strong and consistent independent contribution (**Figure R2**). Moreover, the proportion of missing values for V_{total}, L_c, and L_a is relatively high, and continuing to include them would rapidly reduce the number of complete samples and weaken the stability of the model. And there is significant multicollinearity among some of the variables, particularly, V_{total} overlaps significantly with SSA. Given current limitations in data coverage, independent information gain, and model stability, these variables (carbonization time, heating rate, V_{total}, L_c, and L_a) are not yet sufficient to serve as core input variables for ML models.

The main reason for the unclear correlation between the characteristic value and reversible

capacity is that reversible capacity is contributed by two components: the slope region and the plateau region. Various structure parameters contribute to reversible capacity, meaning that a single structure parameter fails to independently explain changes in reversible capacity. There is significant multicollinearity among the variables, making it difficult for the model to disentangle the independent contributions of these correlated variables, which results in low correlation.

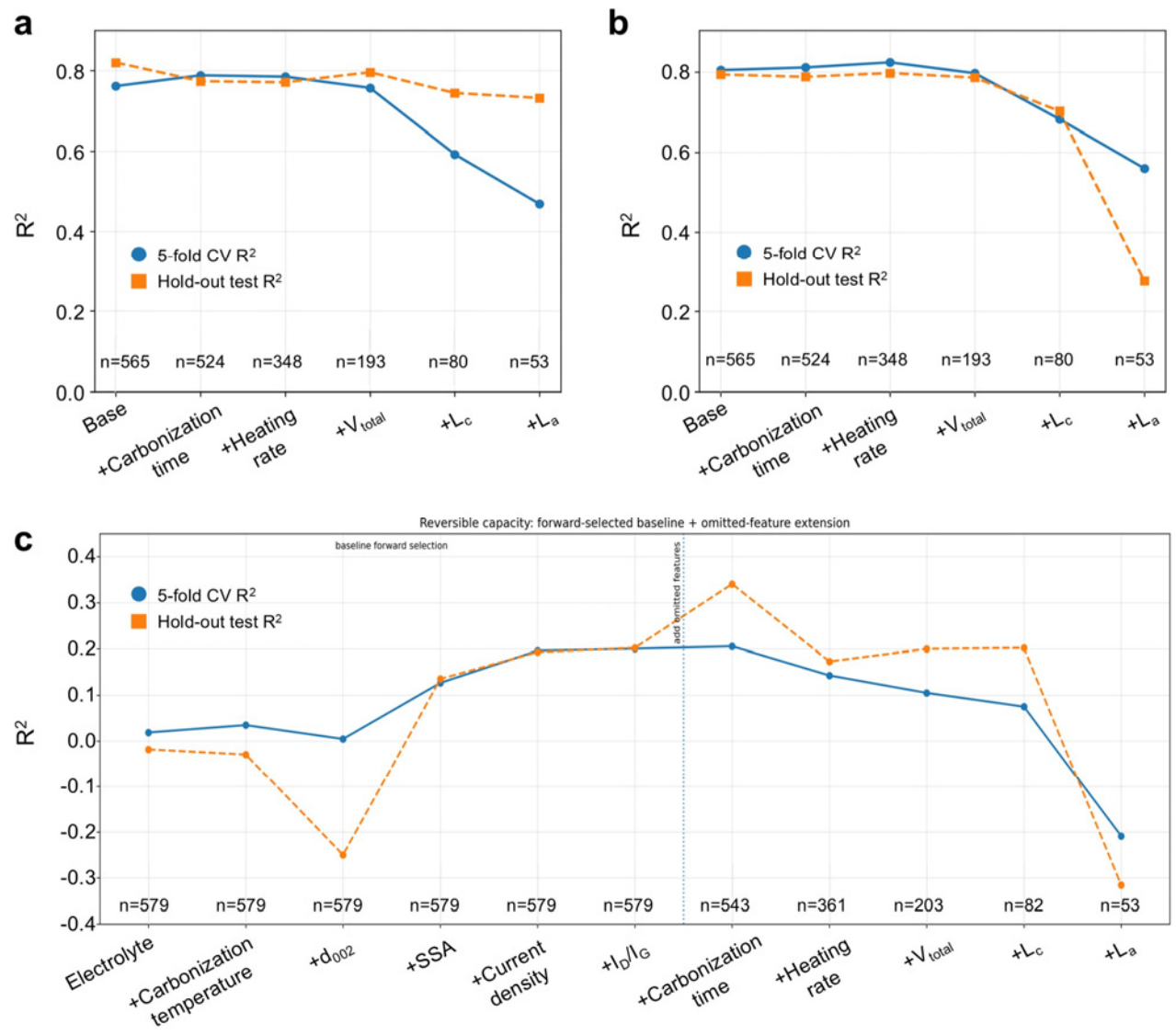

Figure R2. The R^2 change of the RF model after progressively adding missing variables in (a) ICE, (b) reversible plateau capacity, and (c) reversible capacity.

For this reason, we selected the carbonization temperature as a typical process parameter for regulating microstructure, d_{002}, I_D/I_G, and SSA as key structure parameters for evaluating diffusion kinetics, ICE, and reversible plateau capacity as key performance parameters for evaluating the reversible sodium storage performance. In ML, structure parameters and process parameters serve as feature values, while performance parameters serve as target values. These parameters were carefully selected due to the strong interdependencies among them. Furthermore, these parameters share the common feature of being widely documented in the literature, which provides a wealth of data to support the effectiveness of ML algorithms.

Our revision:

1. Added detailed description.

p 6, line 111-114 (Results)

Preparation parameters (carbonization temperature, carbonization time, and heating rate), microstructure parameters (d_{002}, I_D/I_G, SSA, total pore volume (V_{total}), medium basal coherence

length (L_a), and crystal size in the stacking direction (L_c)), and performance parameters (ICE, reversible plateau capacity, and reversible capacity) are collected in the database by data mining from the massive literature.

p 6, line 117-120 (Results)

The carbonization temperature as a typical process parameter for regulating microstructure, the d_{002}, I_D/I_G, and SSA are crucial microstructure features for Na^+ diffusion, and ICE and reversible plateau capacity are key manifestations of reversible sodium storage sites (Supplementary Fig. 7, 8).

2. Added figures.

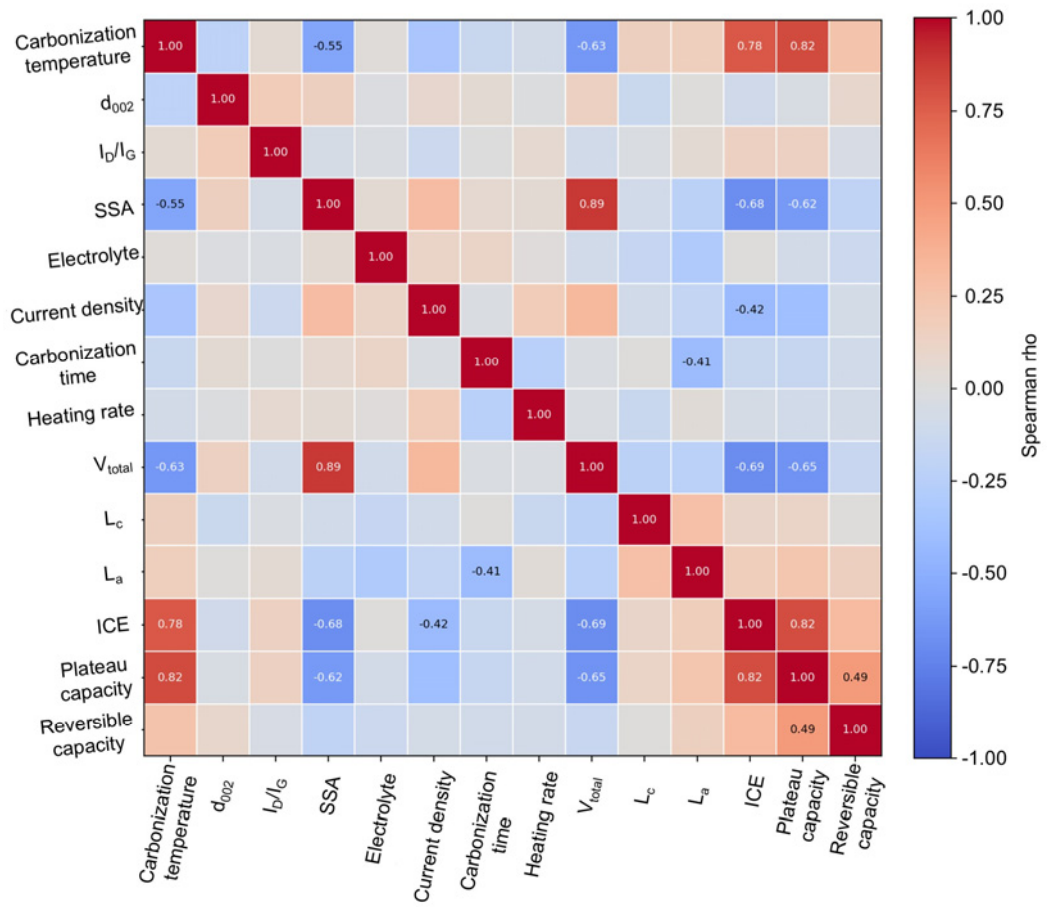

Supplementary Fig. 1. Spearman correlation heatmap between core features, missing variables, and the target variable.

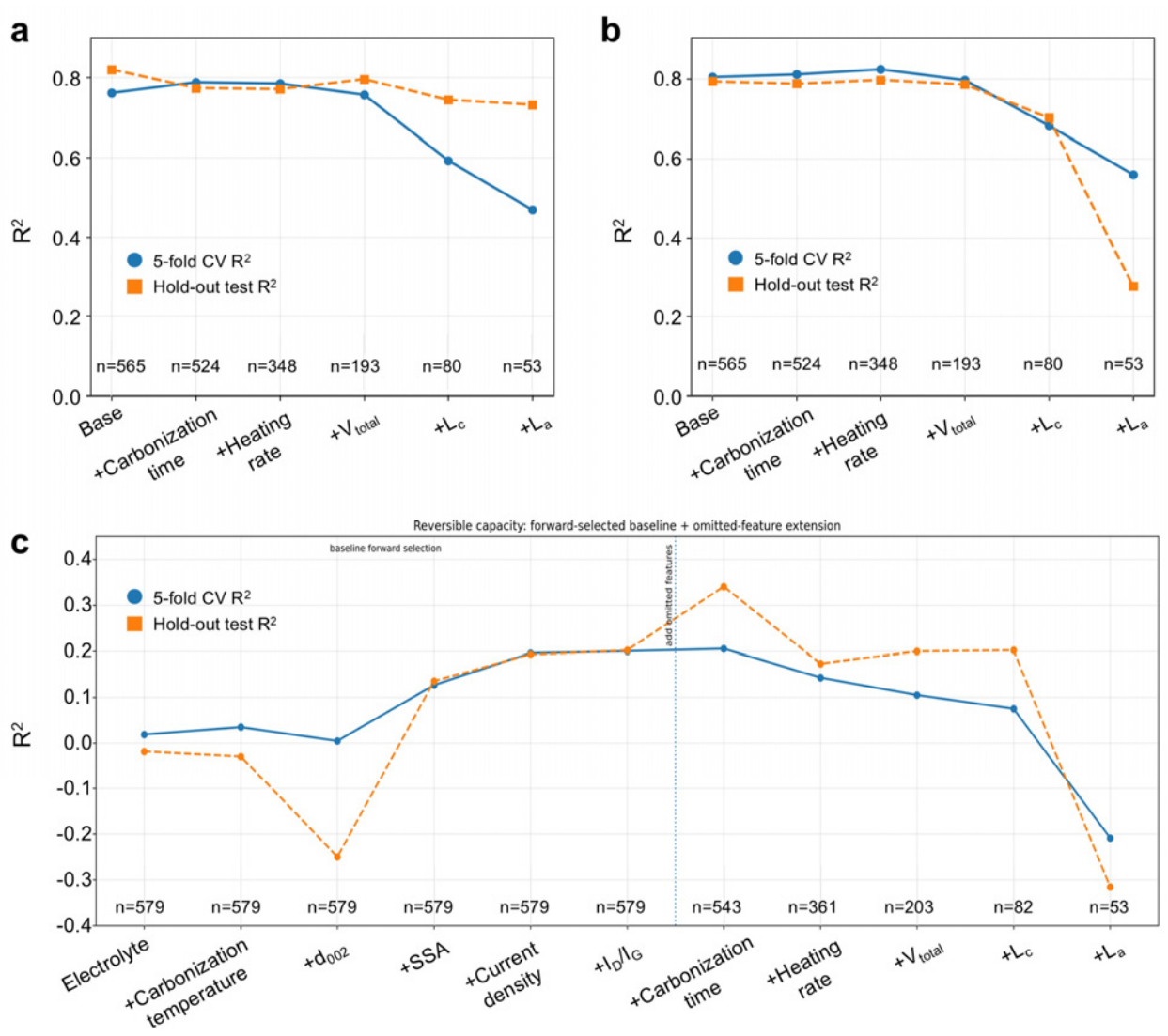

Supplementary Fig. 7. The R^2 change of the RF model after progressively adding missing variables in (a) ICE, (b) reversible plateau capacity, and (c) reversible capacity.

(2) The correlations between these parameters and ICE or reversible plateau capacity have been shown in various works and are well known in the field. Furthermore, the suggested range for each parameter is rather wide, so the hard carbon structures could be significantly different even within the suggested range.

Response: We greatly appreciate the insightful comments of reviewers regarding the possibility of significant variations in HC structures within a relatively broad range of recommendations. And we fully acknowledge that the correlation between these parameters and performance has been demonstrated in various studies. The vast majority of current studies rely on a limited number of experiments to obtain a few and singular structure parameters and performance parameters. Comparing limited data to identify structural parameters that yield optimal performance is insufficient to clearly and systematically reveal the correlation between the structure and properties of HC. Moreover, the structure parameter values obtained under optimal performance conditions may not always be the best choice, as there is an element of chance involved. By leveraging a vast and comprehensive database, ML derives effective weightings from the complex and diverse relationships between structure and performance. This enhances the accuracy and systematic nature of the correlation analysis between the structure parameters and performance parameters of HC. More importantly, ML models rapidly predict various structure parameters to identify potential high-performance HC materials (**Figure R3**).

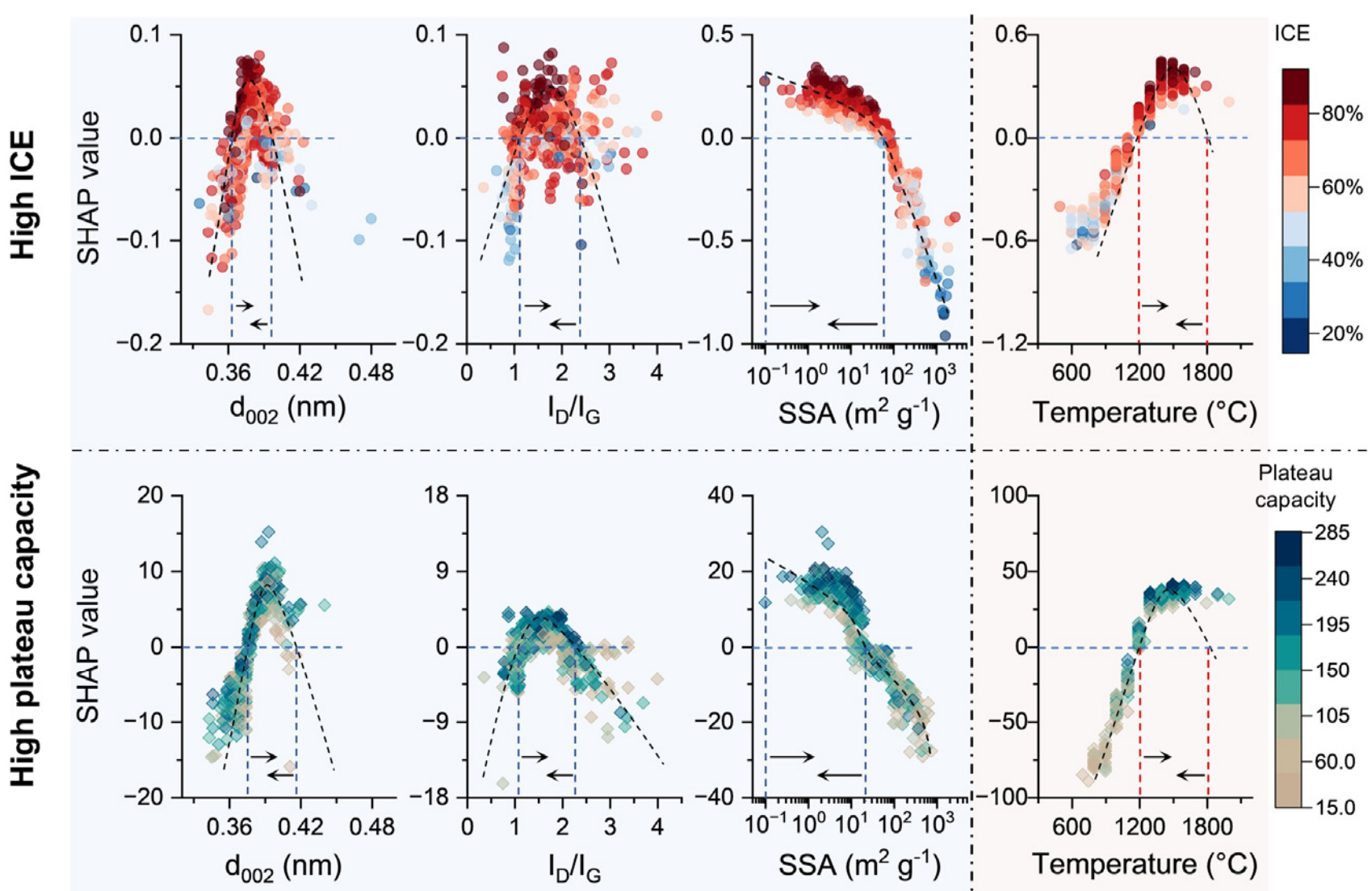

Figure R3. Relationship of ICE and reversible plateau capacity with each structure feature.

In this work, the recommended range for the microstructure of HC was determined to be low SSA (0.26-17.84 m^2 g^{-1}), moderate d_{002} (0.376-0.394 nm), and moderate I_D/I_G (1.10-2.30). The broad range of structures corresponding to the optimal performance of HC is related to multidimensional performance coupling and microstructure equivalence. The performance of HC is typically defined by a combination of various indicators, which often have interdependent relationships. Specifically, the microstructure features required for high ICE, high reversible capacity, high reversible plateau capacity, high rate capability, and high cycling stability in HC are not consistent. Therefore, the optimal structure under different weightings may fall within the same broad range of parameters, but the specific distribution of structures will differ. Furthermore, different microstructure combinations achieve similar performance through different physical mechanisms. There are compensatory relationships between certain structure parameters, such that a change in one parameter can be offset by a change in another, resulting in multiple structure combinations that achieve similar performance. Therefore, the optimal performance of HC corresponds to a variety of structure ranges, and although there are differences in structure, similar performance is ultimately achieved.

Our revision:

1. Added detailed description.

p 5, line 84-86 (Introduction)

Although numerous studies have reached a certain consensus regarding the influence of structure on performance, the range of microstructure characteristics for high-performance HC remains undetermined[26].

p 5, line 86-88 (Introduction)

Based on data from current literature regarding processes, structures, and performance, machine learning (ML) uncovers complex nonlinear relationships behind vast variables, revealing the key microstructure influence rules within trade-offs to provide quantifiable optimal ranges[27,28].

Comment #1. The authors mentioned that high I_D/I_G is beneficial for Na-ion diffusion. Is there a direct correlation between high I_D/I_G and fast Na-ion diffusion?

Response: We thank the reviewer for the insightful question regarding the correlation between high I_D/I_G and fast Na^+ diffusion. The I_D/I_G is the intensity ratio of the D peak to the G peak in Raman

testing, which is typically used to assess the degree of graphitization of HC. A high I_D/I_G indicates that the HC has a rich defect structure (***Adv. Mater. 2026**,* 38, 7, e13193). Defects are important components of HC. Creating defects is commonly approved in respect of enhancing fast Na^+ storage of HC (***Adv. Energy Mater. 2025,*** 15, 31, 2502082).

Defect structures usually have a relatively high surface energy and serve as adsorption sites for Na^+. These sites facilitate the enrichment of Na^+ on the material surface, thereby promoting the diffusion of Na^+ into the bulk phase (***Energy Environ. Sci. 2024,*** 17, 9, 2968-3003). Furthermore, some defect structures (such as oxygen-containing functional groups) modulate the distribution of the local electron cloud, reducing the activation energy (E_a) for Na^+ migration, thereby increasing the charge transfer rate (***Adv. Energy Mater. 2024**,* 14, 23, 2400125).

$$D = D_0 \exp\left(-\frac{E_a}{k_B T}\right)$$

However, the excessive defects lead to disordered carbon layer stacking, forming a complex network of pores. Na^+ must frequently change direction during transport, increasing the length and tortuosity of the effective diffusion path (***Adv. Energy Mater. 2023,*** 13, 18, 2300444). Therefore, simply pursuing a high I_D/I_G is not beneficial for rapid Na^+ diffusion. The I_D/I_G needs to be controlled within a reasonable range to form a conductive network with the surrounding carbon layer structure, thereby effectively improving the diffusion kinetics of Na^+.

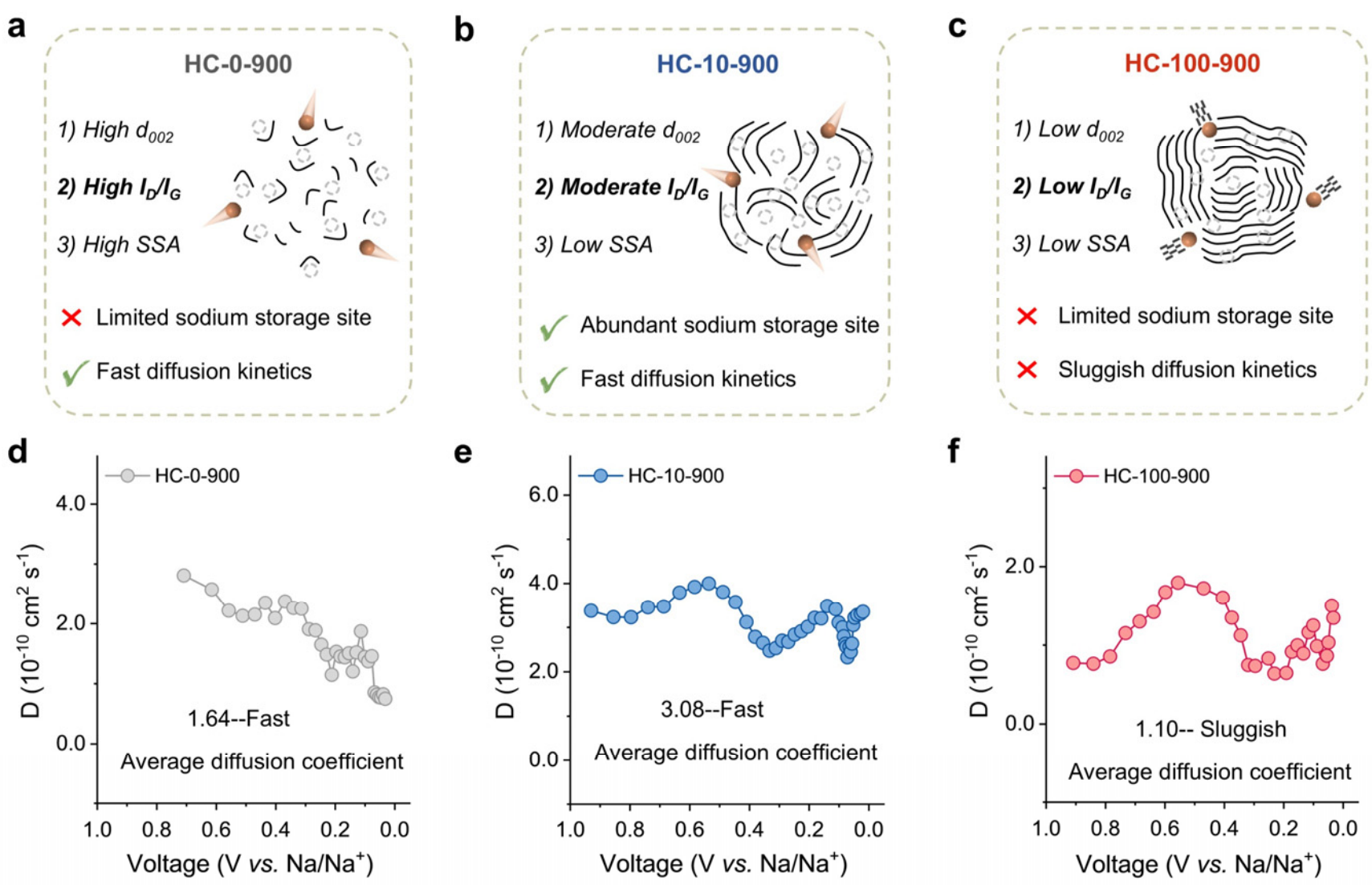

Figure R4. The (a-c) structure characteristics and (d-f) diffusion coefficient of HC-0-900, HC-10-900, and HC-100-900.

In the introduction, we noted that under high-temperature carbonization conditions, HC typically exhibits low I_D/I_G, low SSA, and low d_{002}, which increase the resistance to Na^+ diffusion due to the lack of an unobstructed diffusion pathway. Relatively speaking, increasing I_D/I_G, SSA, and d_{002} improves Na^+ diffusion kinetics by increasing open diffusion pathways, but this is not to pursue excessively high I_D/I_G. Moreover, due to compensatory interactions between structure features, I_D/I_G must work in concert with other structure features to more effectively facilitate Na^+ diffusion. This was further confirmed in the experiment (**Figure R4**): The HC-0-900 exhibits high I_D/I_G, high d_{002}, and high SSA. However, due to the complex diffusion network formed by randomly distributed carbon flakes, Na^+ frequently changes direction during diffusion, thereby increasing the number of effective diffusion paths and the tortuosity of the diffusion process. The HC-100-900 displays low I_D/I_G, low d_{002}, and low SSA. There is a lack of effective diffusion pathways due to the low defect density and long-range ordered carbon layers, which increases the resistance to Na^+ diffusion. In contrast, the HC-10-900 possesses moderate I_D/I_G, moderate d_{002}, and low SSA. Moderate defects and short-range carbon layers form an ordered diffusion network, which enhances the diffusion kinetics of Na^+. In terms of performance, the ICE, reversible capacity, and rate performance of the HC-10-900 (87.4%, 329.3 mAh g^{-1}, 205.1 mAh g^{-1} at 2 A g^{-1}) all outperform those of the HC-0-900 and HC-100-900. Therefore, appropriately increasing the I_D/I_G enhances the diffusion rate of Na^+, thereby improving the reversible storage and rate performance of HC.

Our revision:

1. Added detailed description.

p 4, line 58-60 (Introduction)

While appropriately increasing the I_D/I_G, SSA, and d_{002} of HC improves the diffusion kinetics of Na^+, this inevitably reduces the number of ordered carbon layers and nanopores, thereby limiting the sodium storage sites[11,12].

p 8, line 145-146 (Results)

Appropriately increasing d_{002} and I_D/I_G within a certain range is beneficial for improving the diffusion rate of Na^+, thereby enhancing the reversible sodium storage capacity of HC.

Supplementary Note 10 (Supplementary Information)

As pressure increases, the I_D/I_G of the HC decreases sequentially, indicating that surface-exposed defects are reduced due to the densification of the material. Among these, ordered carbon layers and

appropriate defects of HC-10-900 form a Na^+ diffusion network, which improves the diffusion kinetics of Na^+.

Comment #2. How does the C=O bond associate with graphene sheet curvature?

Response: We thank the reviewer for the question. The C=O bond is a key component of oxygen-containing functional groups in HC, which are widely utilized to modulate the microstructure of HC for optimizing electrochemical performance (***Adv. Energy Mater. 2021,*** 11, 1, 2002981). Introducing C=O bonds into HC not only increases active sites and adsorption energy but also accelerates electron transfer, promotes ion diffusion, and enhances structural stability (***Angew. Chem. Int. Ed. 2026,*** 65, 11, e25941).

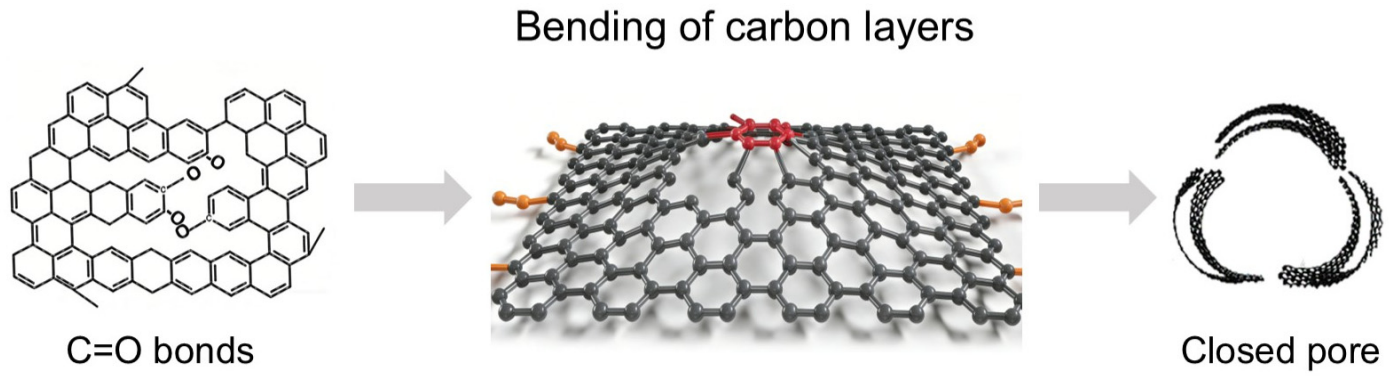

Figure R5. Schematic illustration of the formation of closed pores through the bending of the carbon layer induced by the C=O bond.

There is a significant coupling relationship of structure induction and electronic stabilization between the C=O bond and the curvature of the carbon layer (**Figure R5**). The curvature of the carbon layer is a key structure basis for the stable existence of C=O bonds on the surface and edges of HC, and the introduction of C=O bonds further induce and maintains local curvature of the carbon layer (***Adv. Mater. 2025,*** 37, 28, 2417886; ***Adv. Mater. 2025,*** 37, 11, 2418239; ***Energy Storage Mater. 2024,*** 67, 103282). **Structure Geometry:** In regions where the carbon layer has a high curvature, the hybridized orbitals of the carbon atoms fail to close, forming dangling bonds. Due to high surface energy, these dangling groups readily react with oxygen to form C=O bonds. **Electronic effects:** When a carbon atom forms a C=O bond with an oxygen atom, the local bond angle undergoes compression or stretching to accommodate the high electronegativity of the oxygen atom, resulting in bending of the carbon layer. **Thermal stability:** During low-temperature carbonization, C=O bonds coexist with high-curvature structures. During high-temperature carbonization, the curvature of the carbon layer decreases due to the decomposition of C=O bonds, leading to graphitization of the material. Therefore, introducing C=O bonds under low-temperature

conditions to increase the curvature of the carbon layer effectively constructs a closed-pore in HC.

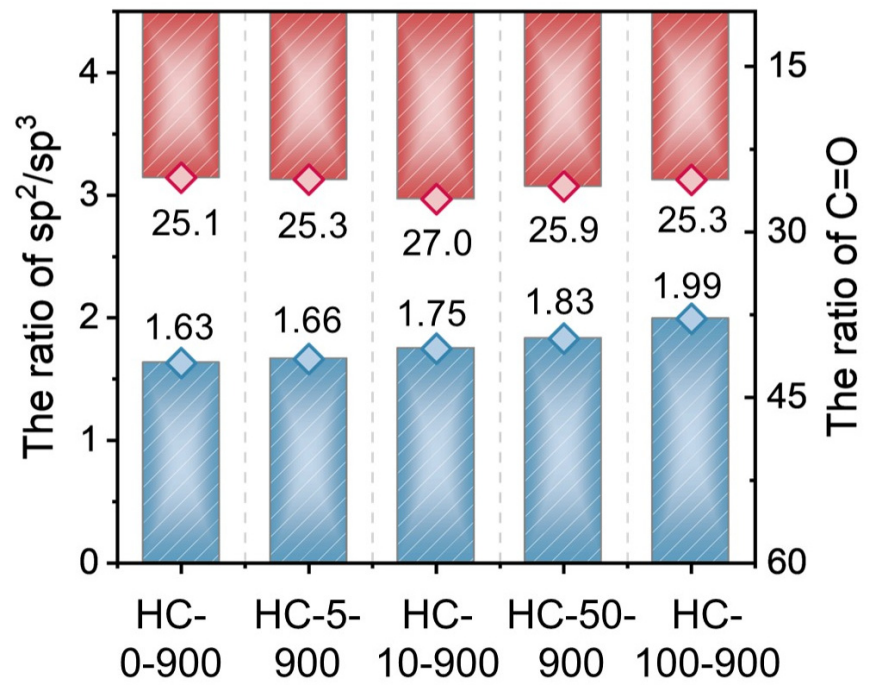

Figure R6. The sp^2/sp^3 ratio and C=O ratio of HC-0-900, HC-5-900, HC-10-900, HC-50-900, and HC-100-900.

In this work, thermal-force coupling directly modulates C=O bonds and carbon layers in HC, thereby influencing the closed-pore structure. Low-temperature thermal field carbonization induces a low degree of graphitization in HC. HC-0-900 possesses a C=O bond content of 25.1%, which induces the carbon sheets to bend, forming a rich closed-pore structure (**Figure R6**). However, this structure is disordered, with a high SSA and numerous defects. Under the influence of thermal-force coupling, the C=O bond content in HC first increases and then decreases as pressure increases, while the sp^2/sp^3 ratio continues to rise. Low-pressure assistance promotes C=O bonds cross-linking and the ordered stacking of carbon layers, inducing the formation of large-volume closed pores. However, under high pressure, the C=O bonds first cross-link and eventually break, as the carbon layer structure continues to develop toward a highly graphitized state, resulting in the elimination of the closed-pore structure. HC-10-900 has a high content of C=O bonds (27.0%) and a moderate carbon layer structure, which plays a crucial role in forming a closed-pore structure and promoting Na^+ diffusion.

Our revision:

1. Added detailed description.

p 12, line 237-240 (Results)

HC-10-900 exhibits more C=O bonds compared to other materials, which is beneficial for enhancing the curvature of the carbon layer to form a closed-pore structure[47]. Besides, the local curvature and large interlayer spacing induced by the C=O bonds provide open diffusion pathways for Na^+, preventing interlayer blockage.

Supplementary Note 14 (Supplementary Information)

This indicates that low-pressure assistance at the thermal field promotes C=O bonds cross-linking and the ordered stacking of carbon layers, inducing the formation of closed pores. However, under high-pressure assistance at the thermal field, the C=O bonds first cross-link and eventually break, as the carbon layer continues to develop toward a highly graphitized state, resulting in the elimination of closed-pores.

Comment #3. There is often a large drop in Na-ion diffusivity at the beginning of the plateau capacity from GITT measurement. However, the thermal-force coupling synthesized samples barely show any drop at the beginning of plateau capacity, showing that the Na-ion kinetics are significantly improved in these samples. Is there any possible reason for this difference between this work and other works?

Response: We thank the reviewer for this insightful question. The diffusion rate of Na^+ in HC is determined by a combination of microstructure characteristics, such as defects, carbon layers, and closed-pore structures. Under low-temperature thermal field carbonization, HC-0-900 exhibits a high SSA, a broad d_{002}, abundant defects, and randomly distributed carbon flakes. This highly disordered microstructure increases the tortuosity of Na^+ diffusion, resulting in a gradual decrease in the diffusion coefficient in GITT testing (**Figures R7a-d**). Compared to HC-0-900, the thermal-force coupling synthesized HC exhibits a trend of first decreasing and then increasing in the low-voltage plateau region (**Figures R7e, i**). This difference originates from thermal-force coupling promoting carbon layer stacking, which creates closed pores between the curved carbon layers, thereby achieving the insertion of carbon layer and the filling of closed pores of Na^+.

Compared to the high-voltage region, thermal-force coupling synthesized HC maintains a high diffusion coefficient in the low-voltage plateau region, which depends on the effective promotion between d_{002}, bulk defects, and closed-pore pore size. HC-10-900 possesses a short-range carbon layer, appropriate d_{002}, abundant bulk defects, and large closed-pore pore sizes (**Figures R7f-h**). The short-range carbon layer and the appropriate d_{002} provide an unobstructed diffusion path, facilitating the diffusion of Na^+ within the carbon layer. Abundant bulk defects connect the short-range carbon layers and closed pores, forming a diffusion network within the bulk phase of HC. Furthermore, the large pore sizes of the closed pores allow a large number of Na^+ to enter

simultaneously. Consequently, Na^+ achieves rapid diffusion into the carbon layers and closed pores. Compared to the high-voltage region, HC-100-900 still maintains a high diffusion coefficient in the low-voltage plateau region. Due to the long-range ordered carbon layers and the narrow d_{002}, which increase the diffusion resistance of Na^+ in the carbon layers, the diffusion coefficient of HC-100-900 is lower than that of HC-10-900 (**Figures R7j-l**). This was further confirmed by in-situ Raman spectroscopy, the G peak of HC-100-900 exhibits a relatively weak shift and a difficult return to its initial position during the charge-discharge process.

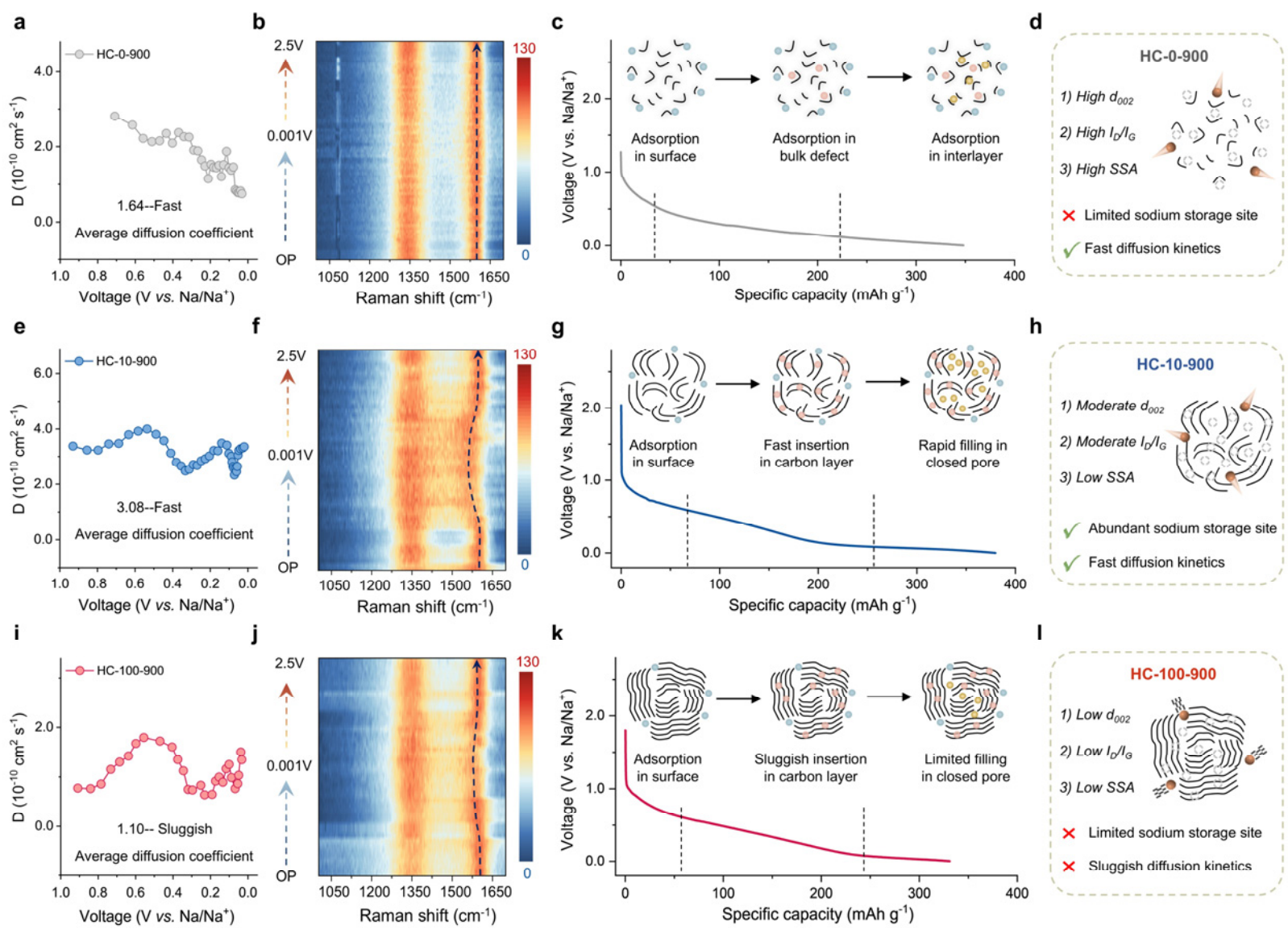

Figure R7. The diffusion coefficient, in-situ Raman, sodium storage mechanism, and structure characteristics of (a-d) HC-0-900, (e-h) HC-10-900, and (i-l) HC-100-900.

Our revision:

1. Added detailed description and references.

p 16, line 334-336 (Results)

This difference originates from thermal-force coupling promoting carbon layer stacking, which creates closed pores between the curved carbon layers, thereby achieving the insertion in carbon layer and the filling in closed pores of Na^+[52].

p 17, line 341-342 (Results)

The above results indicate that the excellent Na^+ diffusion and efficient electron transport of HC-10-900 are attributable to the construction of diffusion pathways and the formation of conductive networks.

Comment #4. Figure 56 in the supporting information was used to support the statement of the appearance of the quasi-metallic Na cluster. However, XPS is a very surface-limited detection. How does XPS present Na cluster formation, which takes place in the bulk of hard carbon?

Response: We greatly appreciate your professional comments. When Na^+ diffuses into the closed pores of HC, the Gibbs free energy change required for the formation of critical nuclei is significantly reduced due to the confined nanoscale space, facilitating the nucleation of sodium clusters within the closed pores. As the depth of discharge increases, more Na^+ diffuse into the closed pores, where they gradually aggregate to form nanoscale clusters (***Adv. Energy Mater. 2026,*** 16, 4, e05227; ***PNAS 2022,*** 119, 40, e2210203119). To detect signals from sodium metal clusters, in situ and ex-situ XRD, Raman, XPS, and NMR techniques are widely used to monitor the formation of sodium clusters. Furthermore, TEM testing and the phenolphthalein solution indicator method have also been employed to directly or indirectly observe quasi-metallic sodium clusters.

In-situ XRD analysis: The (100) and (101) crystal planes of the hexagonal quasi-sodium metal clusters were observed in the discharge range of 0.7–3.0 V via in-situ XRD tests, confirming the formation of the quasi-sodium metal clusters. During discharge at 0.01-0.3 V, the (002) and (011) crystal planes were observed, confirming that the quasi-sodium metal clusters transitioned from the hexagonal to the orthorhombic crystal system (***Adv. Mater. 2025,*** 37, 1, 2410673). **In-situ Raman analysis:** A distinct signal was observed at a low frequency of 0.1 V via in-situ Raman spectroscopy, and this signal became stronger during deep discharge, corresponding to the signal from Na_8 clusters (***Adv. Energy Mater. 2024,*** 14, 18, 2304300). **Ex-situ XPS analysis:** In the low-voltage region, the quasi-metalloid sodium peak of C−Na appears and shifts to the characteristic peak of Na metal with the intercalation of sodium into the interlayer, and as the discharge reaches 0.10 V, sodium initiates to fill the nanopores, resulting in the significant upsurge of the quasi-metallic sodium peak (***Adv. Mater. 2022,*** 34, 13, 2109282). **Ex-situ NMR analysis:** During the plateau region, the 23NassNMR spectra display a broad peak at 920–1040 ppm, corresponding to a Knight shift resulting from the formation of quasi-metallic sodium clusters (***Energy Environ. Sci. 2025,*** 18, 7, 3418-3418). **Ex-situ TEM analysis:** HC after full sodiation revealed clearly visible quasi-metallic Na nano clusters in TEM images because of Na^+ filling-pores (***Adv. Mater. 2025,*** 37, 28, 2417886). **Phenolphthalein solution indicator method:** The basic substance produced by the reaction between the sodium quasi-metal cluster and a protic solvent causes the phenolphthalein solution to turn pink, thereby

confirming the formation of the quasi-metallic sodium clusters (***Adv. Mater.* *2025,*** 37, 18, 2420251).

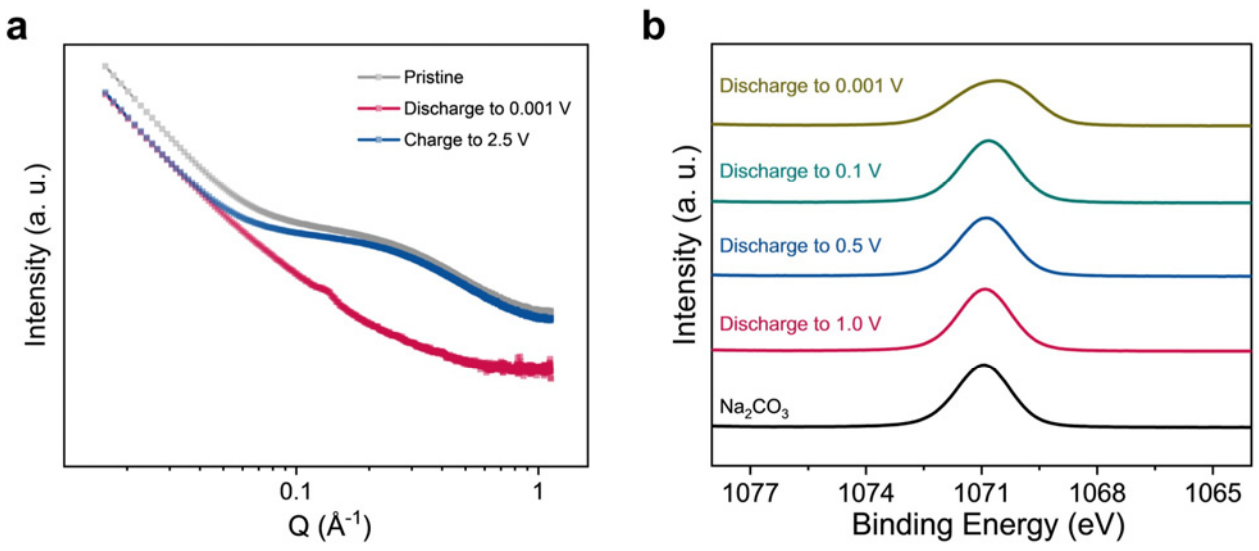

Figure R8. (a) Ex-situ SAXS and (b) Na 1s in ex-situ XPS of HC-10-900.

In our work, ex-situ SAXS was first used to investigate the filling behavior of Na^+ in the closed-pore structure of HC-10-900. The SAXS patterns of HC-10-900 in the initial stage exhibit a higher shoulder peak at ≈0.3 Å^{-1}, which originates from abundant closed pores (**Figure R8a**). When HC-10-900 is fully sodiation, the broad peak in the SAXS spectrum disappears, indicating that the closed-pore structure has been filled with Na^+. Ex-situ XPS analysis was conducted to further investigate the storage state of sodium within the closed pores (**Figure R8b**). In order to avoid the influence of air on the sample, the XPS samples were all carried in an inert atmosphere and transferred into the chamber using a vacuum transfer box. An ionic gun with an energy of 1 kV was selected, and the estimated sputter rate is about 5 nm s^{-1}, the etching depth is 10 nm. As the voltage was discharged to 0.5 V, the position of the Na peak in the XPS spectrum remained unchanged, indicating that sodium is stored in the ramp region in ionic form. When discharged to 0.1 V, the Na peak shifts toward the characteristic of metallic sodium, and upon further discharge to 0.001 V, this shift becomes even more pronounced, confirming the emergence of a metallic sodium phase in the plateau region.

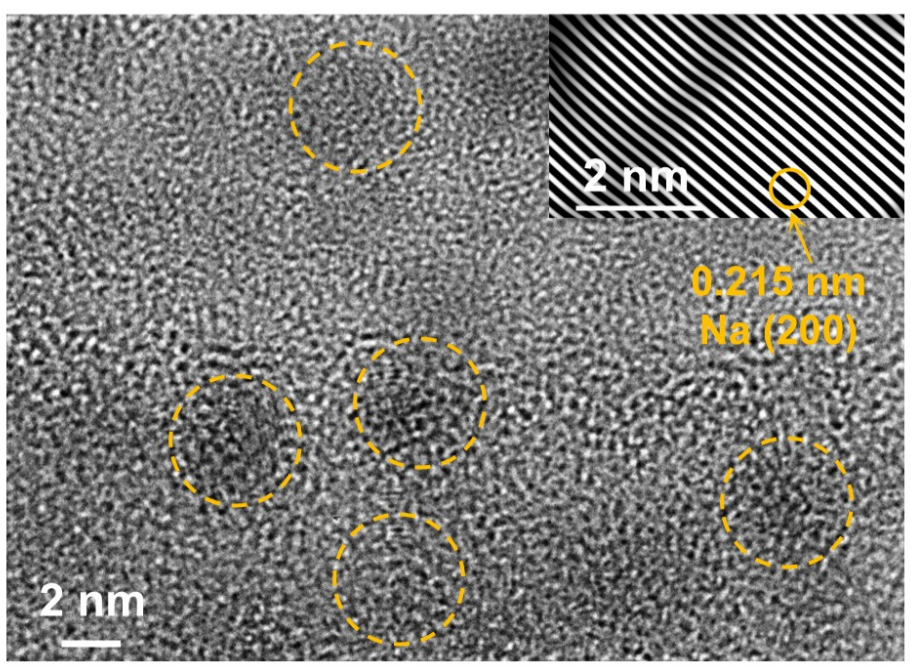

Figure R9. HRTEM image at discharge to 0.001 V of HC-10-900.

To observe the formation of quasi-metallic sodium clusters during the discharge process, HRTEM tests were supplemented for the fully discharged HC-10-900 electrode. A large number of sodium clusters were observed in **Figure R9**, while closed pores cannot be observed, suggesting that the plateau region is caused by the filling of the closed pores by the sodium clusters.

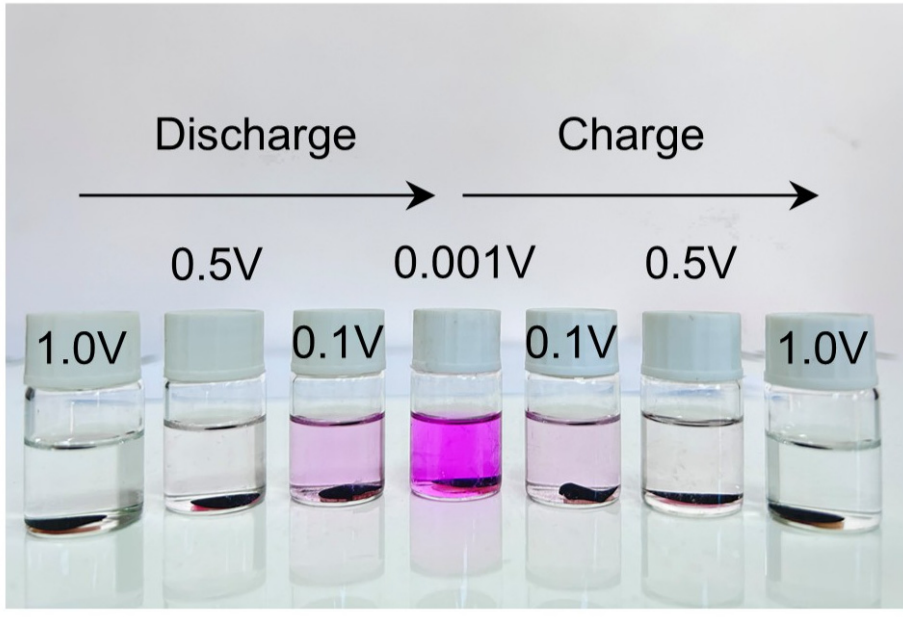

Figure R10. The color reaction of phenolphthalein of HC-10-900.

The presence of the quasi-metallic sodium cluster in HC-10-900 is further confirmed by the color reaction of phenolphthalein (**Figure R10**). The basic substance produced when the quasi-metallic sodium cluster reacts with a protic solvent causes a color change in the phenolphthalein solution. Along with the discharge process, the clear and transparent ethanol solution of phenolphthalein turns pink and gradually deepens, especially in the plateau region. Furthermore, when the voltage reaches 2.5 V, the broad peak of HC-10-900 in the SAXS spectrum returns to its initial position, demonstrating that the filling of sodium into the closed pores is reversible. We employed a variety of ex situ characterization techniques to demonstrate the filling behavior of Na^+ in closed pores and the storage state of quasi-metallic sodium clusters.

Our revision:

1. Added detailed description and references.

p 17, line 354-357 (Results)

Moreover, when discharged to 0.001 V, the sodium characteristic peak shifted toward the sodium-metal peak, and quasi-metallic sodium clusters were observed in the HRTEM test, suggesting that sodium is stored in the closed pore in the form of a quasi-metal[57] (Supplementary Fig. 63).

p 22, line 457-460 (Methodology)

To avoid the influence of air on the sample in the ex-situ XPS test, the electrodes were all carried in an inert atmosphere and transferred into the chamber using a vacuum transfer box. An ionic gun

with an energy of 1 kV was selected, and the estimated sputter rate is about 5 nm s^{-1}, the etching depth is 10 nm.

Supplementary Note 18 (Supplementary Information)

A large number of sodium clusters were observed in the HRTEM test, while closed pores cannot be observed, suggesting that the plateau region is caused by the filling of the closed pores by the sodium clusters[59]. The presence of the quasi-metallic sodium cluster in HC-10-900 is further confirmed by the color reaction of phenolphthalein. Along with the discharge process, the clear and transparent ethanol solution of phenolphthalein turns pink and gradually deepens, especially in the plateau region[60]. The filling behavior of Na^+ in closed pores and the storage state of quasi-metallic sodium clusters were demonstrated by a variety of ex situ characterization techniques.

Supplementary References (Supplementary Information)

[59] Xue, Y. et al. Substitution index-prediction rules for low-potential plateau of hard carbon anodes in sodium-ion batteries. *Adv. Mater.* **37**, 2417886 (2025).

[60] Mao, P. et al. Hard carbon with tailored microstructure via thermal regulation for high-efficiency sodium-ion batteries. *J. Colloid Interface Sci.* **710**, 140026 (2026).

2. Added figures.

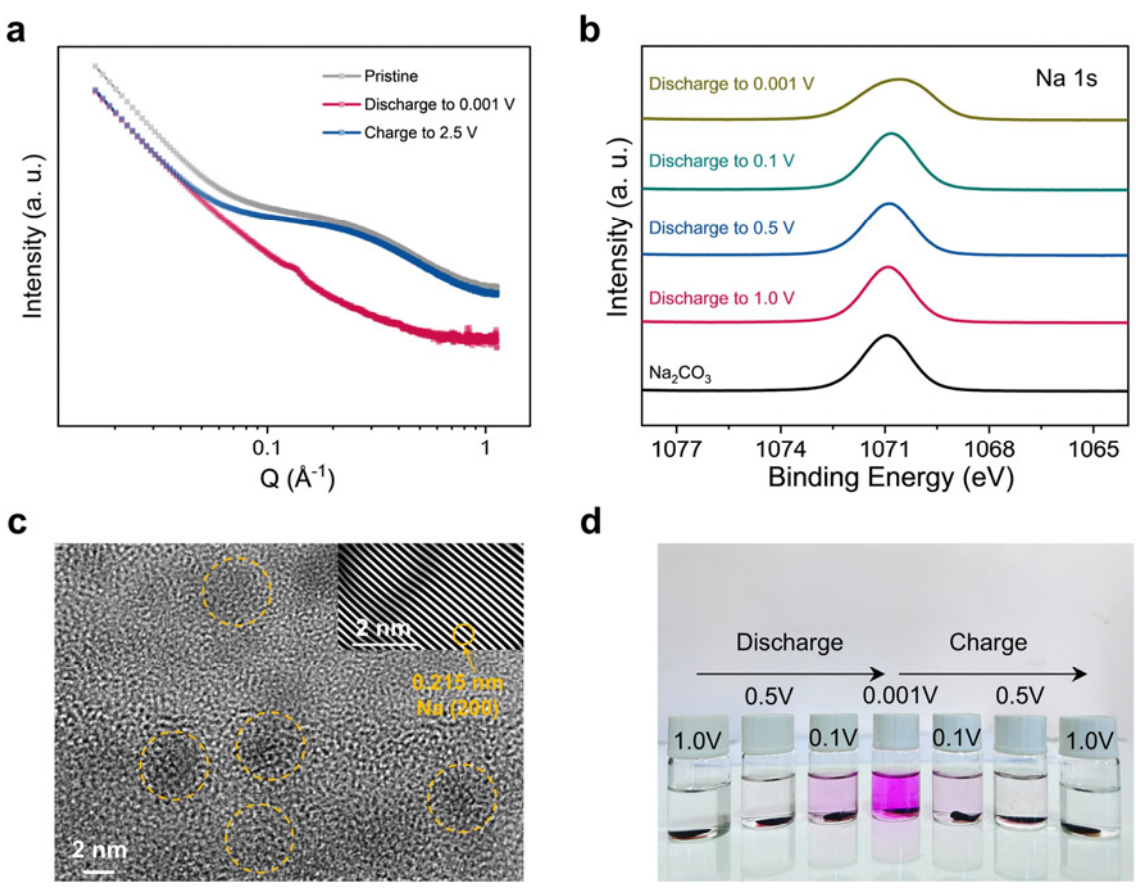

Supplementary Fig. 63. (a) Ex-situ SAXS, (b) ex-situ XPS, (c) HRTEM image at discharge to 0.001 V, and (d) the color reaction of phenolphthalein of HC-10-900.

Comment #5. It could be expected that there would be more functional groups or impurities left in the thermal-force coupling synthesized hard carbon because of the low synthesis temperature, the applied pressure, and the confinement of the mold. Is there a possibility that this could affect the performance of hard carbon?

Response: We thank the reviewer for the insightful question regarding the correlation between experimental conditions and the consistency of the HC structure. Before carbonization experiments, the heating equipment (tube furnaces, muffle furnaces, and hot-press carbonization furnaces) is evacuated using a vacuum pump to create a negative-pressure environment inside the chamber, thereby eliminating interference from air. Subsequently, the vacuum pump is shut off, and argon gas is introduced to fill the furnace chamber until exhaust gas is regularly expelled. Argon gas is continuously supplied throughout the entire carbonization process. Moreover, the hot-press mold is a highly dense graphite mold, which does not introduce graphite into the HC during the carbonization process. By following a rigorous experimental protocol, we minimize the impact of external environmental factors on the products.

Table R1. The yield of HC obtained from multiple batch experiments.

**Sample**	**Yield 1**	**Yield 2**	**Yield 3**	**Average yield**
HC-0-900	35.19%	35.84%	36.68%	35.90%
HC-0-1300	19.40%	19.70%	20.30%	19.80%
HC-10-900	43.08%	43.30%	43.83%	43.40%

Multiple batches of experiments under identical conditions: To verify the consistency in the preparation, structure, and performance of the HC, HC-0-900, HC-10-900, and HC-0-1300 were subjected to experiments, characterization, and testing of multiple batches. HC-0-900 was synthesized three times, yielding yields of 35.19%, 35.84%, and 36.68%, respectively, with an average yield of 35.90% (**Table R1**). HC-10-900 was synthesized three times, yielding yields of 43.08%, 43.30%, and 43.83%, respectively, with an average yield of 43.40%. HC-0-1300 was synthesized three times, yielding yields of 19.40%, 19.70%, and 20.30%, respectively, with an average yield of 19.80%. The yield remained consistent across experiments of multiple batches in HC-0-900, HC-10-900, and HC-0-1300, demonstrating excellent reproducibility in the synthesis of HC. In terms of structure, the (002) and (100) peaks of HC-0-900, HC-10-900, and HC-0-1300, synthesized in multiple batches, exhibit similar intensities and positions in the XRD patterns, respectively (**Figure R11**). Moreover, no characteristic peaks of graphite were observed in the XRD pattern, as the mold was made of graphite, demonstrating remarkable structure consistency.

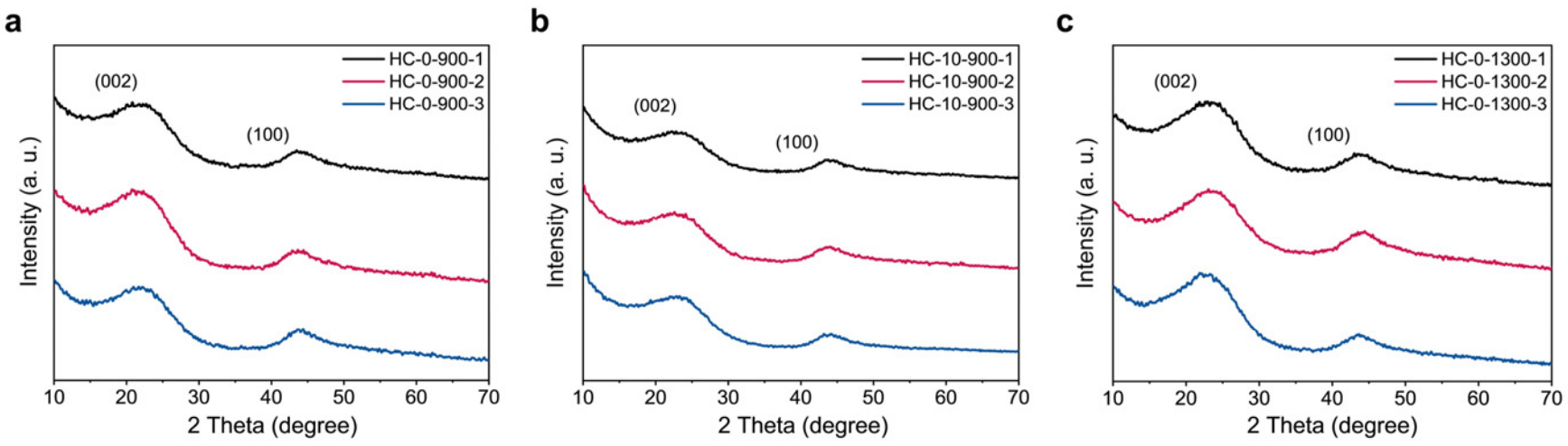

Figure R11. The XRD patterns of (a) HC-0-900, (b) HC-10-900, and (c) HC-0-1300 were obtained from multiple batch experiments.

In electrochemical performance testing, the HC-0-900 synthesized in multiple batches exhibited reversible capacities of 191.6 mAh g^{-1}, 194.0 mAh g^{-1}, and 188.2 mAh g^{-1}, respectively (**Figure R12**). The HC-10-900 synthesized in multiple batches exhibited reversible capacities of 333.7 mAh g^{-1}, 331.7 mAh g^{-1}, and 328.3 mAh g^{-1}, respectively. The HC-0-1300 synthesized in multiple batches exhibited reversible capacities of 276.7 mAh g^{-1}, 286.4 mAh g^{-1}, and 280.7 mAh g^{-1}, respectively. Multiple batches of HC-0-900, HC-10-900, and HC-0-1300 provided similar reversible capacities, respectively, demonstrating high consistency in performance. Therefore, based on multiple experiments, characterization, and performance testing indicate that neither thermal-force coupling carbonization nor single-thermal-field carbonization introduces additional functional groups or impurities into the HC, regardless of experimental conditions.

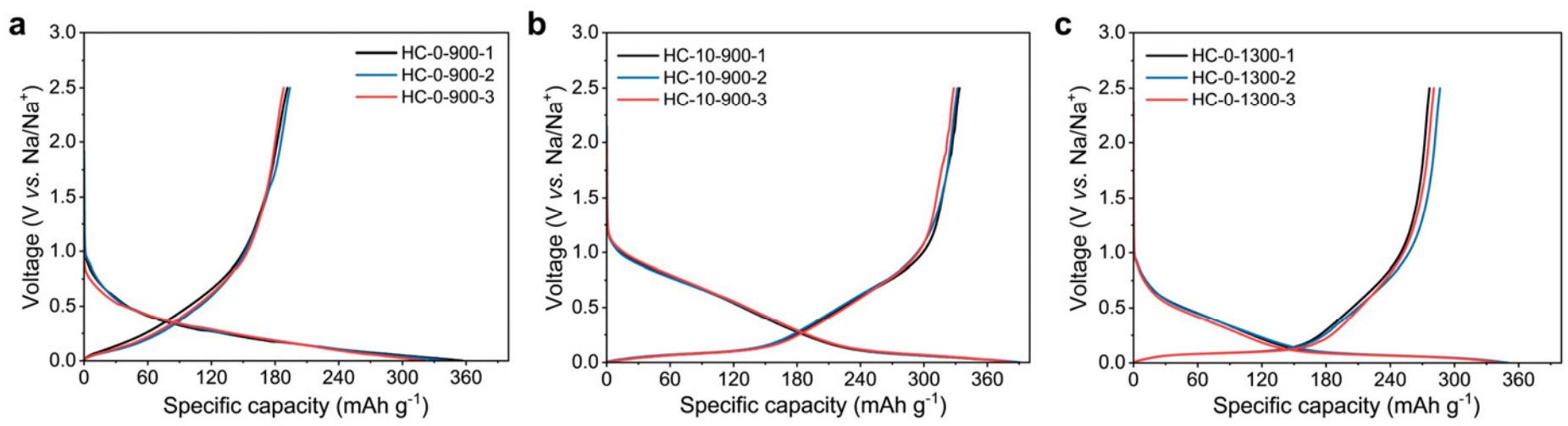

Figure R12. The initial charge-discharge curves at 25 mA g^{-1} of (a) HC-0-900, (b) HC-10-900, and (c) HC-0-1300 obtained from multiple batch experiments.

HC functional groups and impurities under different experimental conditions: In low-temperature thermal field carbonization and low-temperature thermal-force coupling carbonization, the HC samples exhibit broad (002) and (100) peaks in XRD patterns, which correspond to the structural characteristics of amorphous carbon. Furthermore, the absence of characteristic peaks of graphite in the XRD pattern confirms that impurities were not introduced into the thermal-force coupling-prepared HC. Compared to HC-0-900, the oxygen content in thermal-force coupling-

prepared HC gradually decreases as pressure increases. This is because pressure promotes the breaking of carbon-oxygen bonds and accelerates the ordered rearrangement of the carbon structure. Among these, HC-10-900 exhibits more C=O bonds compared to other materials, which is beneficial for enhancing the curvature of the carbon layer to form a closed-pore structure. Besides, the local curvature and large interlayer spacing induced by the C=O bond provide open diffusion pathways for Na^+, preventing interlayer blockage. Benefiting from this, the HC-10-900 exhibits excellent overall electrochemical performance, including high ICE, high reversible capacity, excellent rate capability, and outstanding cycling stability.

Our revision:

1. Added detailed description.

p 21, line 438-442 (Methodology)

Before carbonization experiments, the heating equipment (tube furnaces, muffle furnaces, and hot-press carbonization furnaces) is evacuated using a vacuum pump to create a negative-pressure environment inside the chamber, thereby eliminating interference from air. Subsequently, the vacuum pump is shut off, and argon gas is introduced to fill the furnace chamber until exhaust gas is regularly expelled. Argon gas is continuously supplied throughout the entire carbonization process.

Supplementary Note 14 (Supplementary Information)

Compared to LTC, the thermal-force coupling effect causes the oxygen content in the HC to decrease as pressure increases. And the C=O bond content first increases and then decreases as pressure increases, while the sp^2/sp^3 ratio continues to rise. This indicates that low-pressure assistance at the thermal field promotes C=O bonds cross-linking and the ordered stacking of carbon layers, inducing the formation of closed pores. However, under high-pressure assistance at the thermal field, the C=O bonds first cross-link and eventually break, as the carbon layer continues to develop toward a highly graphitized state, resulting in the elimination of closed-pores. HC-10-900 has a high content of C=O bonds (27.0%) and a moderate carbon layer structure, which promotes the formation of closed-pores. Moreover, the curvature of the carbon layer induced by the C=O bond facilitates the rapid Na^+ diffusion into the closed pores.

Comment #6. NVP is usually recognized as a stable cathode material, and the half-cell test for NVP in this work also shows nice stability. What is the reason for the degradation in the NVP/HC full

cell?

Response: We greatly appreciate your insightful question regarding HC-10-900//NVP full cell capacity degradation. We strongly agree that NVP cathode materials demonstrate excellent cycling stability in half-cell testing. Moreover, the HC-10-900 anode material also exhibits outstanding cycling stability in half-cell testing. However, the HC-10-900//NVP full cell shows significant capacity degradation during cycling tests. The reasons may include the following: 1) **An unreasonable ratio between the anode capacity and the cathode capacity (N/P ratio).** An excess of HC-10-900 anode material consumes sodium from the NVP cathode, forming an irreversible SEI layer, which leads to a decrease in capacity. Conversely, an excess of NVP cathode material causes sodium deposition and the formation of dendrites, which also results in a decline in cycling performance. 2) **Side reactions on the cathode surface.** Although the NVP structure is stable, the electrolyte undergoes oxidative decomposition on the NVP cathode surface at a high voltage of 3.8 V, forming a high-impedance CEI layer. 3) **Continuous consumption of electrolytes.** During the cycling process, the SEI and CEI layers break down and reform, resulting in the continuous consumption of the electrolyte.

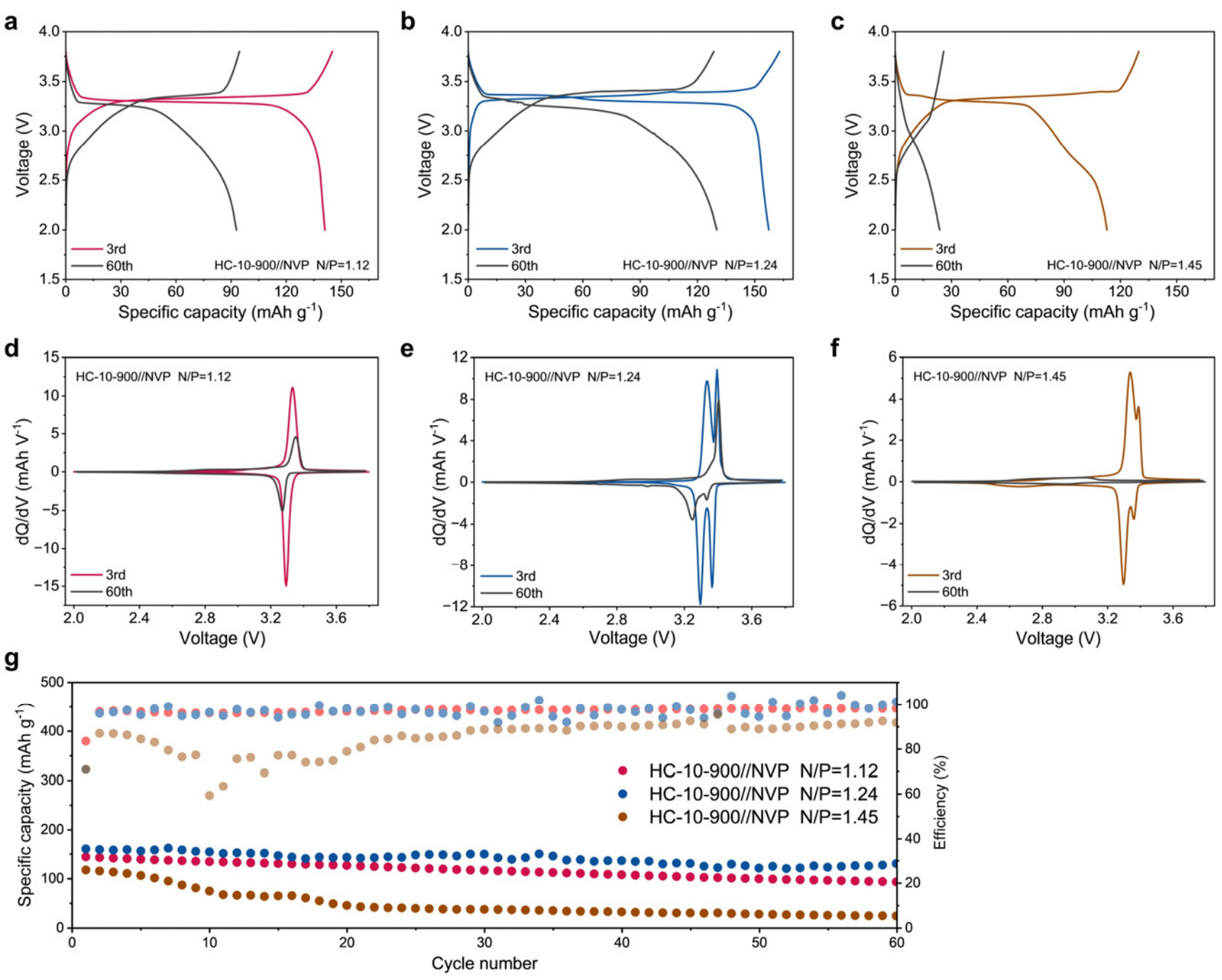

Figure R13. (a-c) Charge-discharge curves at 25 mA g^{-1} for HC-10-900//NVP full cells with N/P ratios of 1.12, 1.24, and 1.45, respectively. (d-f) The dQ/dV curves at 25 mA g^{-1} for HC-10-900//NVP full cells with N/P ratios of 1.12, 1.24, and 1.45, respectively. (g) Cycle performance at 25 mA g^{-1} HC-10-900//NVP full cells with N/P ratios of 1.12, 1.24, and 1.45, respectively.

To further analyze the causes of HC-10-900//NVP full cell capacity degradation, we assembled HC-10-900//NVP full cells with N/P ratios of 1.12, 1.24, and 1.45, respectively. The HC-10-900//NVP full-cell with N/P ratios of 1.12 maintains the working plateau at *ca.* 3.23-3.35 V (**Figure R13a**). The full cell provides a discharge capacity of 141.1 mAh g^{-1} in the 3rd cycle. The oxidation and reduction peaks of the full cell gradually weaken during the cycling process (**Figure R13d**). The capacity retention of the full cell is 64.7% after 60 cycles. This is primarily because the capacity at high voltages on the HC anode cannot be fully utilized, leading to an excess of NVP at the cathode, which facilitates dendrite formation, thereby reducing cycle stability. When the N/P ratio is increased to 1.24, the HC-10-900//NVP full-cell with N/P ratios of 1.24 maintains the working plateau at *ca.* 3.26-3.37 V (**Figure R13b**). The full cell provides a discharge capacity of 157.3 mAh g^{-1} in the 3rd cycle. The oxidation and reduction peaks of the full cell remain relatively stable throughout the cycling process (**Figure R13e**). The capacity retention of the full cell increased to 81.5%. after 60 cycles. When the N/P ratio continuously increases to 1.45, the working plateau and oxidation/reduction peaks of the HC-10-900//NVP full cell with an N/P ratio of 1.45 gradually disappear during cycling (**Figures R13c, f**). The full cell provides a discharge capacity of 112.8 mAh g^{-1} in the 3rd cycle. The capacity retention of the full cell is only for 20.3%. after 60 cycles (**Figure R13g**). This is attributed to the excess anode consuming sodium from the NVP cathode, forming an irreversible SEI layer, and the cathode being unable to supply sufficient sodium to the anode, resulting in a decline in cycling performance. Therefore, an unreasonable N/P ratio is the primary cause of capacity degradation throughout the HC-10-900//NVP full cell entire cycle life.

Our revision:

1. Added detailed description.

p 15, line 297-301 (Results)

Benefiting from the excellent low-voltage characteristics of HC-10-900, the HC-10-900//NVP full-cell still maintains the working plateau at *ca.* 3.26-3.37 V (Supplementary Fig. 45). The HC-10-900//NVP full cell provides a discharge capacity of 157.3 mAh g^{-1} in the 3rd cycle, and the capacity retention of the HC-10-900//NVP full cell is 81.5% after 60 cycles (Supplementary Fig. 46).

p 22, line 471 (Methodology)

The HC-10-900//NVP, HC-0-900//NFM, HC-0-1300//NFM, and HC-10-900//NFM full cells were assembled in coin cells (CR 2032) by NVP and NFM as cathode, respectively. The N/P of the full cell was controlled within the range of 1.1-1.3.

2. Added figures.

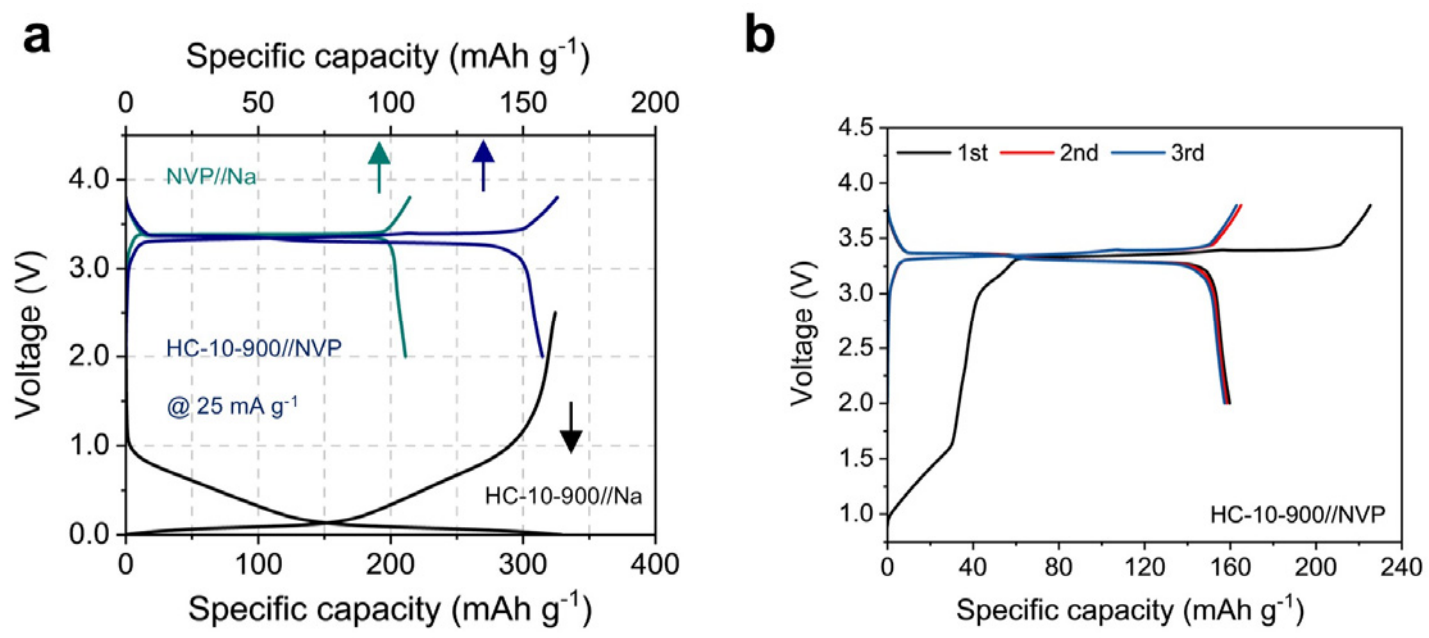

Supplementary Fig. 45. (a) Charge-discharge curves at 3rd of NVP//Na, HC-10-900//Na, and HC-10-900//NVP full cell. (b) Charge-discharge curves of HC-10-900//NVP full cell.

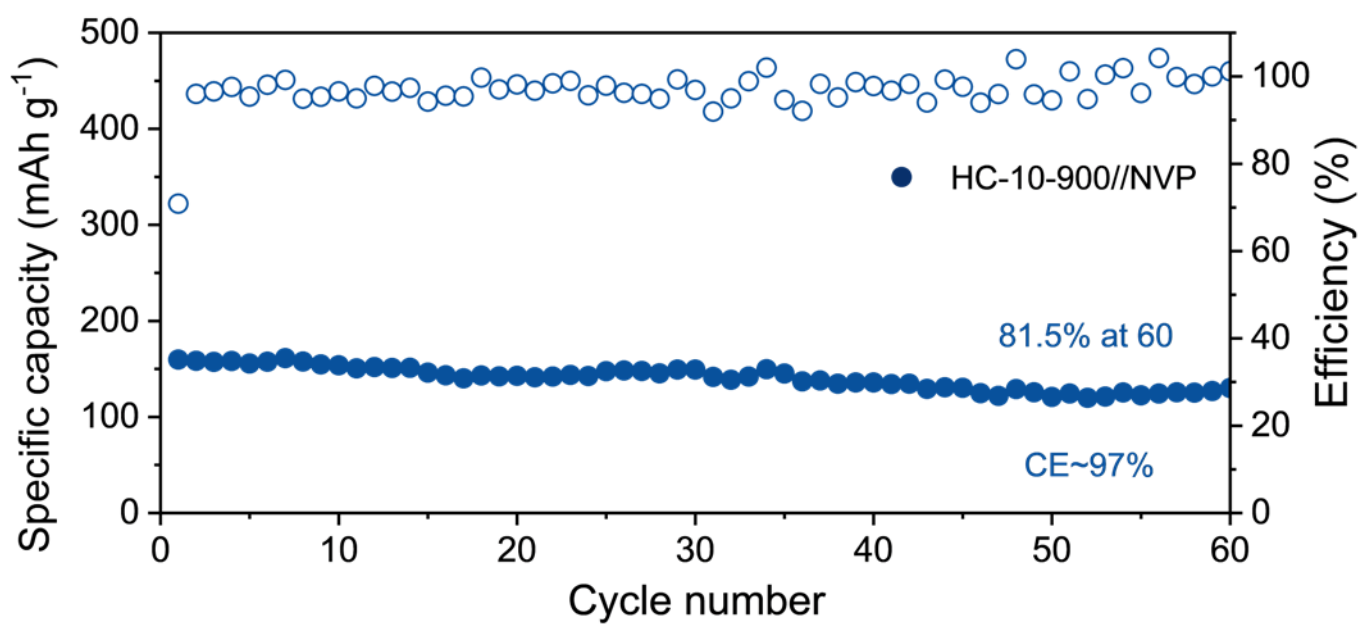

Supplementary Fig. 46. Cycle performance at 25 mA g^{-1} of HC-10-900//NVP full cell.

Comment #7. The length of 1 timestep was not mentioned in the MD simulation method.

Response: We deeply appreciate this detailed and insightful comment from the reviewer. We sincerely apologize for the oversight regarding the lack of the length of 1 timestep. The time step is a key parameter in molecular dynamics simulations, directly determining the numerical stability, physical accuracy, and reproducibility of the simulation. In the molecular dynamics simulations, we used a timestep of 0.15 to ensure that the high-frequency vibrational modes of the carbon atoms were accurately captured, while also ensuring the stability of the numerical integration.

Our revision:

1. Added detailed description.

p 21, line 15-16 (Methodology)

During the carbonization simulation, the temperature was set at 1173.15 K, and the pressures

were atmospheric, 10 MPa, and 100 MPa, respectively, followed by 2,000,000 timesteps in the NPT system. To balance computational efficiency and accuracy, one timestep is set to 0.15. The dynamics of atoms in the system follow the classical Newtonian motion.

We sincerely thank you for your constructive comments and careful evaluation of our work. We hope that the substantial revisions and new analyses provided in this response satisfactorily address all of your concerns.

Point-by-Point Response

(Black type: Reviewer's comments; Blue type: Our response; Red type: Our revision)

Reviewer #3 (Remarks to the Author):

This work merges computational and experimental approaches to assist in solving a long-standing problem with the performance and commercialization of sodium-ion batteries, which is to improve sodium storage sites and diffusion kinetics and thereby improve battery performance. The authors achieved this goal and could produce batteries with capacities as high as 1.47 Ah at a high energy density of 216.1 Wh/kg.

Response: We sincerely thank the reviewer for their comprehensive summary and highly positive evaluation of our work. We have carefully considered all your suggested changes and have incorporated them into the revised manuscript to further enhance its scientific rigor and clarity. Our detailed, point-by-point responses below.

Comment #1. For the paper to be suitable for a very high-impact-factor journal such as Nature Communications, the authors need to probe more into the findings and try to explain the observations. Explain how and why these results are obtained and how they match or differ from previous studies. For most parts of the work, results are reported without attempting to explain what accounted for the results. For example, why did HC-10-900 give a better reversible capacity and rate performance than HC-0-900? How does pressure influence the material obtained and, by extension, the performance? This is just an example, but try to dig more into all the claims made in the work and incorporate appropriate references to support the explanations.

Response: We are very grateful to the reviewers for their constructive suggestions on improving the quality and depth of the manuscript. To this end, we conducted an in-depth analysis of the relationship between the preparation process, structure characteristics, and performance metrics of hard carbon (HC) by combining structure characterization, molecular dynamics simulations, and electrochemical performance testing.

The HC-10-900 exhibits superior reversible capacity and rate performance compared to the HC-0-900, a characteristic determined by its microstructure. The HC-0-900 exhibits a highly disordered microstructure characterized by a high I_D/I_G, high SSA, high d_{002}, randomly distributed carbon

flakes, and abundant micropores (**Figure R1a**). Although high I_D/I_G, high d_{002}, and high SSA promote Na^+ diffusion to some extent, the randomly distributed carbon flakes create complex diffusion paths for Na^+, thereby reducing the overall efficiency of Na^+ diffusion. Furthermore, the high defects and randomly distributed carbon flakes provide extremely limited reversible sodium storage sites. In contrast, HC-10-900 exhibits microstructure characteristics that combine both ordered and disordered features, characterized by low SSA, moderate d_{002}, moderate I_D/I_G, short-range ordered carbon layers, and a rich closed-pore structure (**Figures R1b, c**). The low SSA, moderate d_{002}, moderate I_D/I_G, and short-range carbon layers form a diffusion network that facilitates rapid Na^+ transport, significantly enhancing Na^+ diffusion kinetics. At the same time, the short-range carbon layers and abundant closed-pore structures provide a wealth of reversible sodium storage sites at low-voltage. Thanks to the synergistic advantages of multi-component structure, the HC-10-900 outperforms the HC-0-900 in both reversible capacity and rate performance (**Figures R1d, e**).

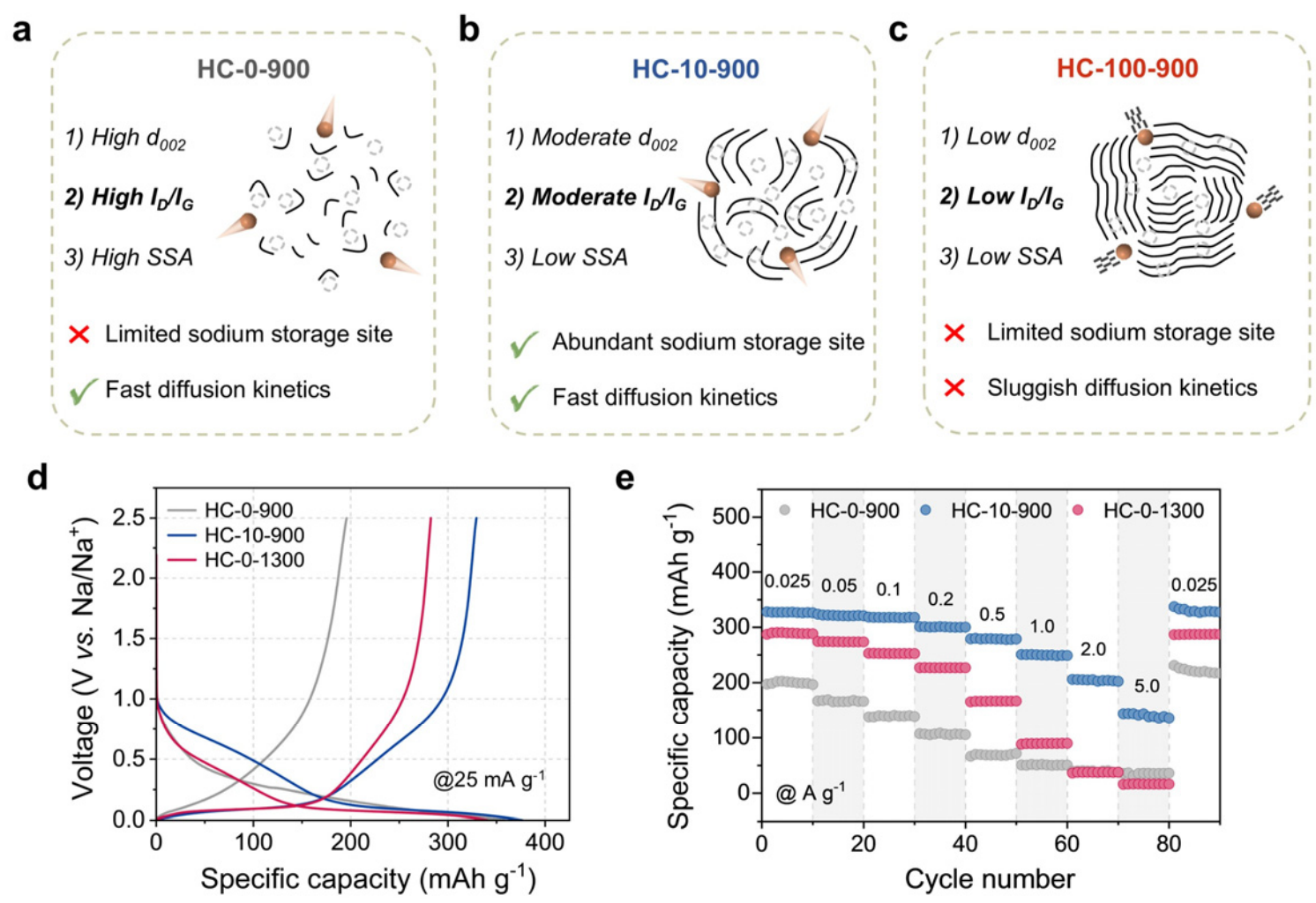

Figure R1. (a-c) The structure characteristics, (d) initial charging and discharging curves, and (e) rate performance. of HC-0-900, HC-10-900, and HC-100-900.

From the above, the microstructure advantages of HC-10-900 are significantly superior to those of HC-0-900, a result determined by the preparation process. The HC-0-900 and HC-10-900 share the same carbonization temperature in their preparation processes, the only difference lies in the presence or absence of pressure, which underscores the importance of pressure control in determining microstructure. Under low-temperature thermal field carbonization, the carbon matrix undergoes significant decomposition reactions, resulting in the breaking of carbon-oxygen bonds

and the release of decomposition gases, which hinder the orderly cross-linking of the carbon framework (**Figure R2a**). This is the primary cause of the highly disordered structure of HC-0-900. During low-temperature thermal-force coupling carbonization, the applied pressure creates a spatially confined carbonization environment, promoting the ordered cross-linking of carbon atoms and the formation of ordered carbon layers (**Figures R2b-d**). These carbon layers are further stacked under pressure to form a closed-pore structure. Moreover, surface defects gradually couple to form bulk defects as densification is induced by pressure. Therefore, the HC-10-900 provides wide layer spacing, a suitable carbon layer size, and abundant bulk defects under low-temperature thermal-force coupling carbonization, which is the microstructure basis for achieving excellent overall electrochemical performance.

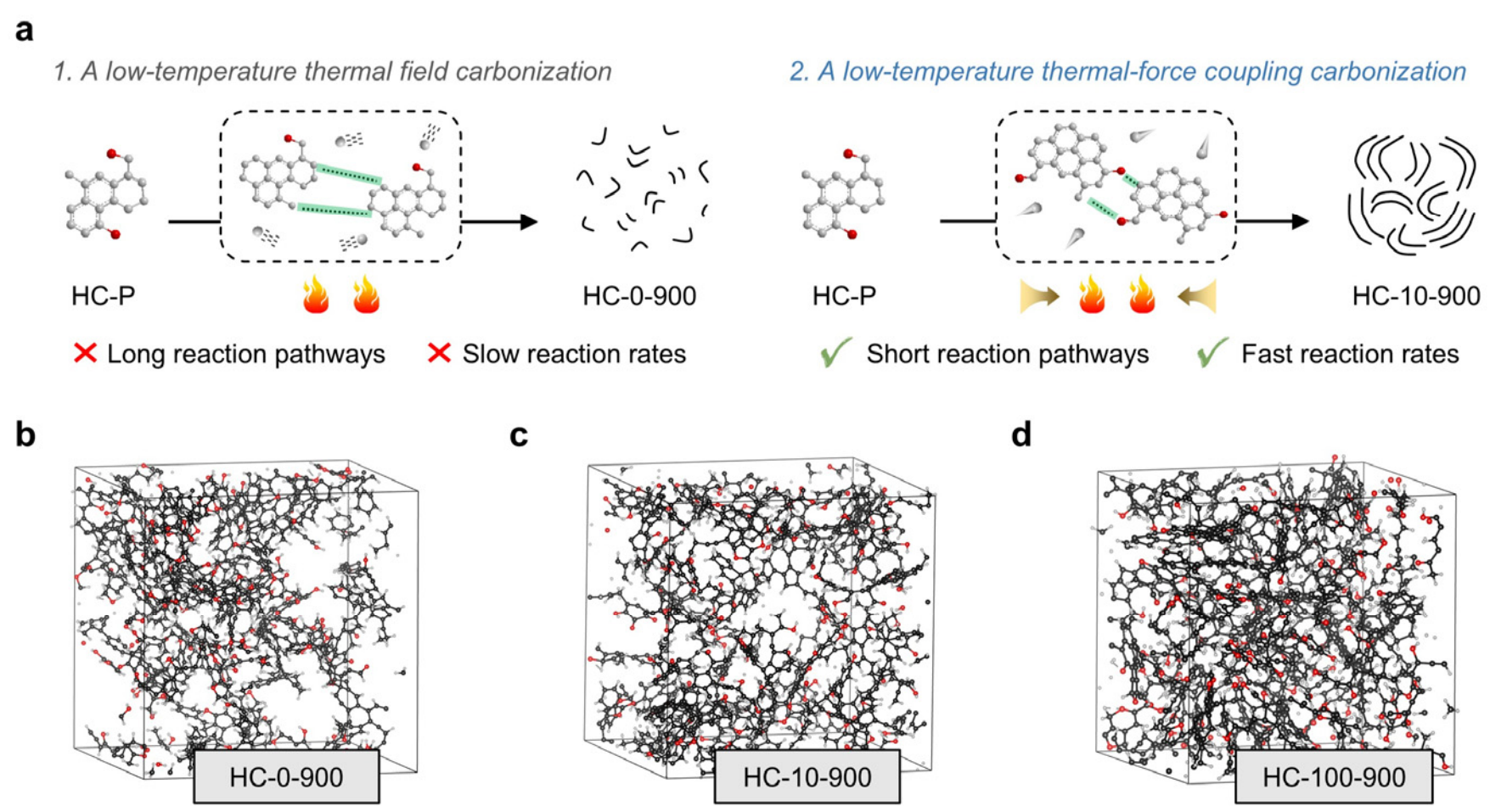

Figure R2. (a) Schematic diagram of a low-temperature thermal-force coupling strategy for preparing high-performance HC. (b-d) Molecular dynamics simulations of HC-0-900, HC-10-900, and HC-100-900.

Our revision:

1. Added detailed description.

p 9, line 183-185 (Results)

This morphological transition results from thermal-force coupling effects that promote the rearrangement of the carbon framework and interlayer slippage, which facilitates the reduction of SSA.

p 11, line 211-213 (Results)

Besides, the R factor, L_a, and L_c increase, and $|g^o|$ values decrease of HC with increasing pressure,

indicating that the compression and orderliness of the carbon layer become more pronounced[43,44] (Supplementary Fig. 21, 22).

p 11, line 215-217 (Results)

The I_D/I_G and SSA decrease progressively with increasing pressure, which is attributed to the densification of the material, where surface-exposed defects are gradually incorporated into the bulk defects.

p 11, line 230-233 (Results)

The changes in open-pore and closed-pore structures indicate that the disordered carbon layers at low pressure are rearranged to form new pores, which subsequently transform into a low-porosity, ordered graphitic arrangement at high pressure.

p 13, line 260-262 (Results)

The excellent reversible sodium storage performance of HC-10-900 is attributed to its carbon layers with wide layer spacing and abundant closed-pore structure.

p 13, line 265-267 (Results)

Thanks to the diffusion network formed by bulk defects and short-range carbon layers in HC-10-900, the diffusion kinetics of Na^+ within the carbon layers and closed pores are enhanced.

p 13, line 274-278 (Results)

Under a single thermal-field processing, HC-0-900 exhibits low RI values due to the highly disordered structure at low temperatures, which provides limited reversible sodium storage sites and complex diffusion pathways. Increasing the carbonization temperature promotes the formation of ordered carbon layers and a closed-pore structure, providing abundant reversible sodium storage sites, thereby resulting in the high RI values observed for HC-0-1300 (Supplementary Fig. 41).

p 14, line 285-286 (Results)

This is attributed to the synergistic effect of large layer spacing, ordered graphite layers, large-sized closed pore structures, and abundant bulk defects.

p 17, line 341-342 (Results)

The above results indicate that the excellent Na^+ diffusion and efficient electron transport of HC-10-900 are attributable to the construction of diffusion pathways and the formation of conductive networks.

Supplementary Note 6 (Supplementary Information)

The evolution mechanism of the HC microstructure under the LTFC was analyzed by adjusting the pressure. Under low-temperature thermal field carbonization (LTC), the carbon matrix undergoes significant decomposition reactions, resulting in the breaking of carbon-oxygen bonds and the release of decomposition gases, which hinder the orderly cross-linking of the carbon framework. This results in the HC-0-900 exhibiting high I_D/I_G, high SSA, high d_{002}, randomly distributed carbon flakes, and abundant micropores. During low-temperature and low-pressure coupling carbonization, the applied pressure creates a spatially confined carbonization environment, promoting the ordered cross-linking of carbon atoms and the formation of carbon layers. The stacking of small carbon layers induces the formation of high-diameter open pores. As the pressure increased to 10 MPa, the carbon layers were further compressed and grew in an ordered manner, inducing the formation of a closed-cell structure between the curved carbon layers. When the pressure reaches 50 MPa, the carbon layer size further increases and its curvature decreases, resulting in a reduction in the number and volume of closed pores. Under the high pressure of 100 MPa, the carbonization space is drastically compressed, accelerating the rate of ordered cross-linking among carbon atoms, thereby inducing the formation of long-range ordered graphite-like carbon layers. This also eliminates defects and closed pores, thereby blocking the diffusion pathways for Na^+. Among these, the HC-10-900 provides wide layer spacing, a suitable carbon layer size, and abundant bulk defects under LTFC, which is the microstructure basis for achieving excellent overall electrochemical performance.

Comment #2. Does the morphology of the materials influence their properties in any way? In other studies, it is found that morphology influences material properties as it relates to their ability to load and release drugs (https://pubs.acs.org/doi/10.1021/acsapm.4c01765) (https://pubs.rsc.org/en/content/articlelanding/2021/za/d5tb00289c/unauth). Can this translate to morphology being able to influence the battery performance of the HC's obtained here?

Response: We are highly grateful to the reviewers for raising insightful questions regarding the impact of material morphology on battery performance. We fully agree with the view of reviewers that the morphology of the material is a key factor in predicting drug delivery performance, which is crucial for explaining how the morphology of HC materials influences their electrochemical performance.

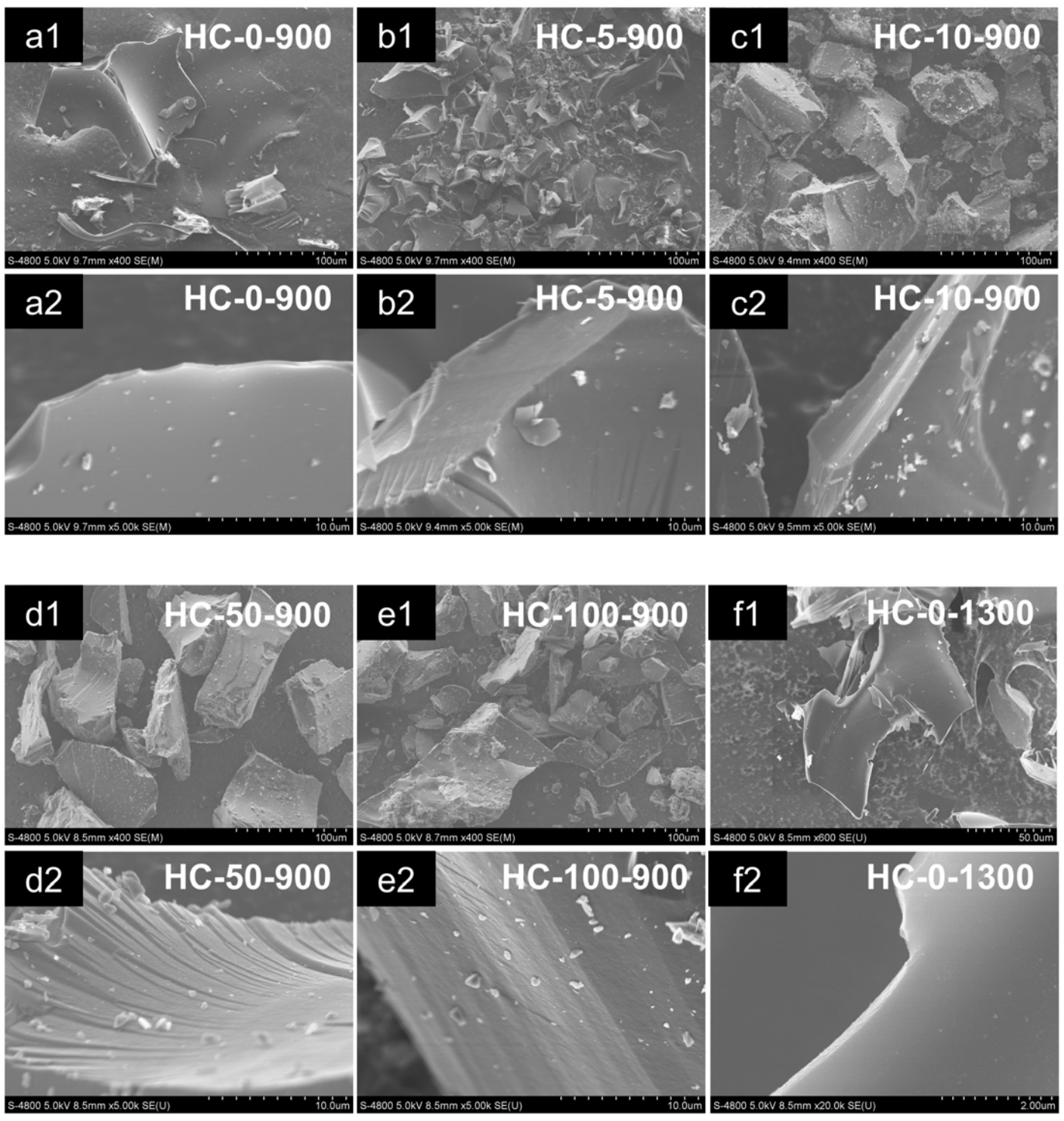

Figure R3. SEM images of (a) HC-0-900, (b) HC-5-900, (c) HC-10-900, (d) HC-50-900, (e) HC-100-900, and (f) HC-0-1300.

In this work, the morphology of HC-0-900 exhibits thin carbon flakes approximately 500 nm thick, whereas the thermal-force coupling-synthesized HC appears as micron-scale dense bulk material (**Figure R3**). These two distinctly different morphologies exhibit significant differences in both electrolyte wetting and side reactions, which consequently affect the electrochemical performance of HC. In nitrogen adsorption and desorption tests, thin carbon sheets exhibit high porosity and high SSA. This increases the contact area with the electrolyte, leading to the formation of a thicker SEI layer and an increase in side reactions, thereby reducing the ICE of HC (**Figure R4**). Furthermore, the SEI layer is prone to cracking and regenerating during the cycling process. The thin carbon sheets provide a large number of surface adsorption sites, enabling sodium storage at high voltages. In contrast, the densified bulk exhibits low SSA and low porosity, which minimizes the contact area with the electrolyte, forming a thin and robust SEI layer, thereby helping to improve the ICE of HC. Moreover, the densified bulk typically stores sodium in the carbon layers and closed pores, enhancing the reversible storage of Na^+ at low voltages. Therefore, the HC-0-900 with high SSA of 436.5 m^2 g^{-1} and high open volume of 0.148 cm^3 g^{-1} only provides low ICE of 54.7% and low reversible specific capacity of 195.8 mAh g^{-1}. The HC-10-900 with low SSA of 2.28 m^2 g^{-1} and

low open volume of 0.005 cm^3 g^{-1} provides high ICE of 87.4% and low reversible specific capacity of 329.3 mAh g^{-1}.

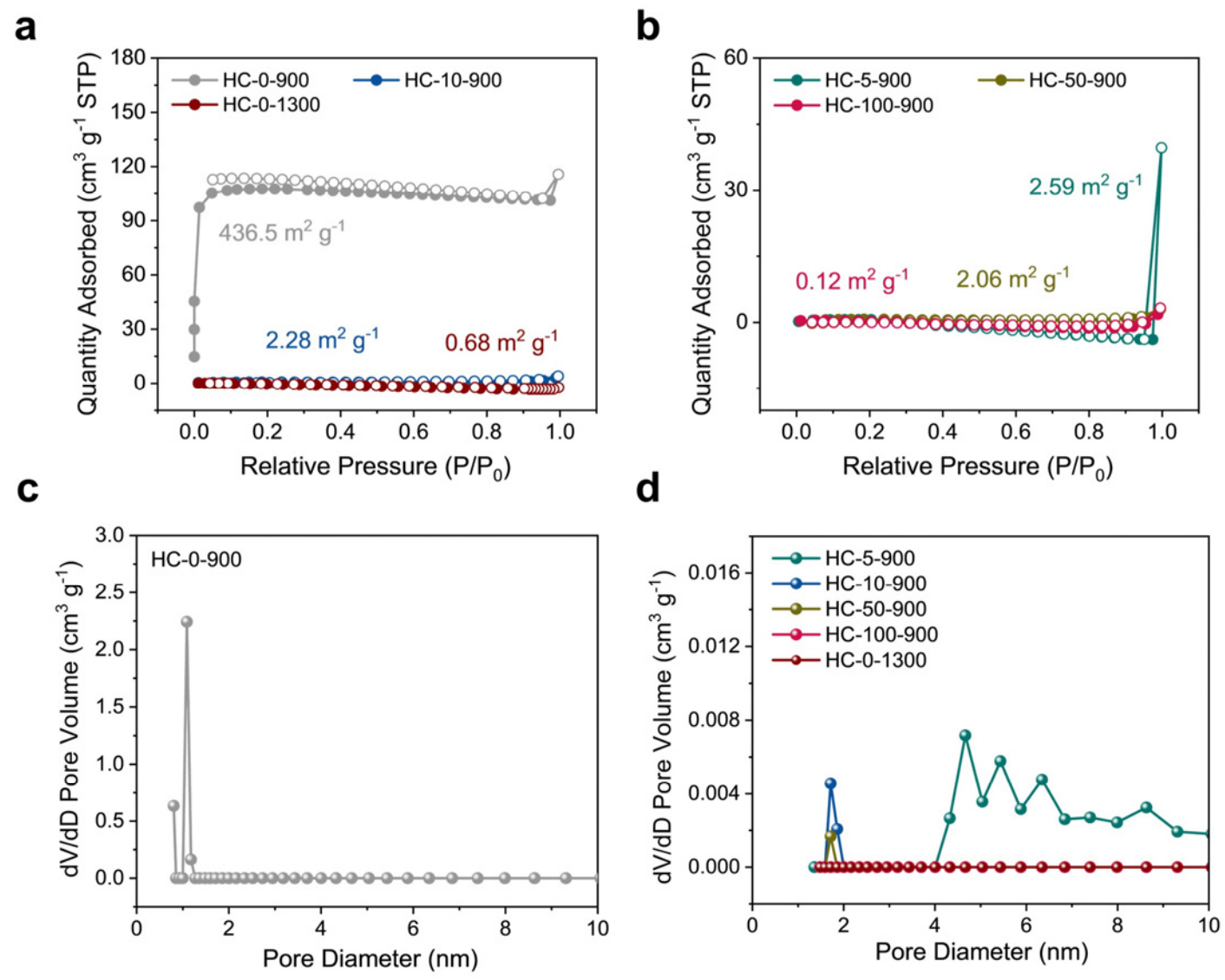

Figure R4. (a, b) Nitrogen adsorption/desorption isotherms and (c, d) Pore size distribution of HC-0-900, HC-5-900, HC-10-900, HC-50-900, HC-100-900.

Our revision:

1. Added detailed description and references.

p 9, line 183-185 (Results)

This morphological transition results from thermal-force coupling effects that promote the rearrangement of the carbon framework and interlayer slippage, which facilitates the reduction of SSA.

p 11, line 215-217 (Results)

The I_D/I_G and SSA decrease progressively with increasing pressure, which is attributed to the densification of the material, where surface-exposed defects are gradually incorporated into the bulk defects.

p 30, line 660-664 (References)

[27] Owusu, S. Y., Doulah, A. B. M. S. U., Edlabadkar, V. A., George, K. J. & Leventis, C. S. The effect of nanomorphology as quantified via the K-index on the drug delivery properties of isocyanate-derived aerogels. *ACS Appl. Polym. Mater.* **6**, 10655-10668 (2024).

[28] Owusu, S. Y., Boateng, M. A. & Soni, R. U. Machine learning predictions of drug release from isocyanate-derived aerogels. *J. Mater. Chem. B* **13**, 6233-6245 (2025).

Comment #3. The features used for machine learning (ML) analysis can be improved. The authors should include current density, crystallite size (L_a and L_c), annealing time, pore volume, and heating rate in the analysis, as these features can also significantly affect sodium-ion battery performance.

Response: We greatly appreciate the professional suggestions of the reviewers regarding the analysis of machine learning (ML) features. Building on the existing characteristic parameters (carbonization temperature, current density, d_{002}, I_D/I_G, and SSA), we have further incorporated carbonization time and heating rate into the process parameters, and added L_a, L_c, and pore volume (V_{total}) to the structure parameters, to conduct a more comprehensive and systematic analysis of the correlations among the process parameters, structure parameters, and performance parameters of HC.

During the revision process, we collected, mapped, and organized the added feature parameters. After re-evaluating them, we still decided not to incorporate these variables into the final ML model. Based on a comprehensive assessment, 1) Some variables lack sufficient incremental importance in the expanded model. 2) Parts of the variables exhibit a high proportion of missing values, and including them would significantly reduce the available sample size. 3) Some variables exhibit strong multicollinearity with existing core variables.

Table R1. The change in R^2 after progressively adding missing variables in the Random Forest (RF) model.

Step	Missingness of added features	Complete cases (n)	ICE 5-fold CV R^2	Plateau 5-fold CV R^2
Initial features	-	565	0.762	0.805
+ Carbonization time	7.3%	524	0.788	0.812
+ Heating rate	35.4%	348	0.785	0.825
+ V_{total}	46.5%	193	0.757	0.797
+ L_c	49.4%	80	0.592	0.682
+ L_a	54.7%	53	0.469	0.560

We conducted a sensitivity analysis with gradual incorporation of eigenvalues based on the key data from the original ICE and reversible plateau capacity (**Table R1**). In ascending order of missing rate, carbonization time, heating rate, V_{total}, L_c, and L_a were added sequentially. At each step, remove samples with missing values from the current feature set and recalculate the 5-fold cross-validation (CV) and hold-out test performance using the RF parameters locked for the original task. The results show that the 5-fold CV R^2 of the RF model based on initial features was 0.762 in ICE, after adding carbonization time, it increased to 0.788, and to 0.785 after adding heating rate (**Figure R5a**). However, when V_{total}, L_c, and L_a were further included, the 5-fold CV R^2 subsequently decreased to 0.757, 0.592, and 0.469, respectively. For reversible plateau capacity, the 5-fold CV R^2 of the RF model based on initial features was 0.805 (**Figure R5b**). After incorporating carbonization time and heating rate, these values increased to 0.812 and 0.825, respectively, but then decreased to 0.797, 0.682, and 0.560 after further including V_{total}, L_c, and L_a. As can be seen, directly incorporating carbonization time and heating rate into the RF model further increases the 5-fold CV R^2.

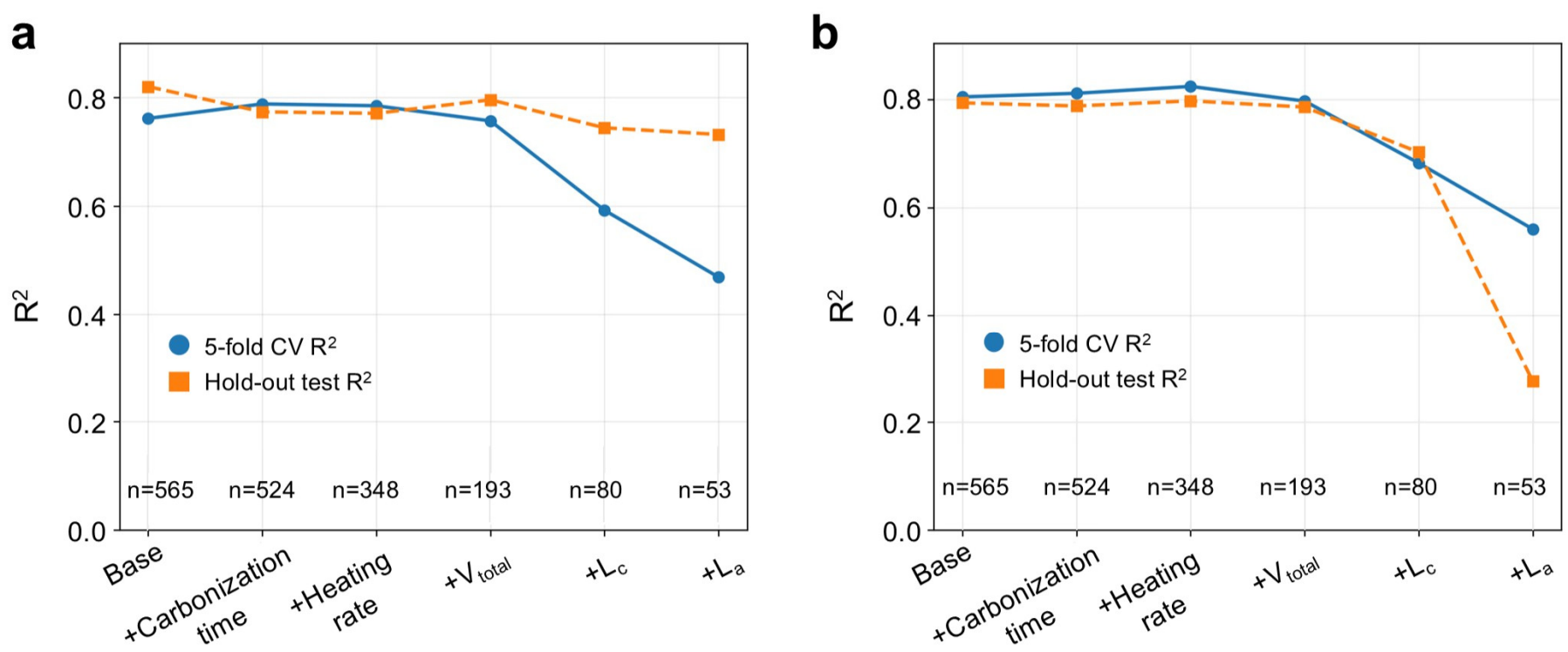

Figure R5. The R^2 change of the RF model after progressively adding missing variables in (a) ICE, and (b) reversible plateau capacity.

To analyze the extent to which carbonization time and heating rate contribute to the performance of HC, we present the Shapley Additive exPlanations (SHAP) Beeswarm after incorporating carbonization time and heating rate into the RF model (**Figure R6**). The results show that the dominant factors remain carbonization temperature, SSA, and current density in both ICE and reversible plateau capacity. In contrast, carbonization time and heating rate consistently rank at the bottom of the importance hierarchy, with their SHAP values mostly clustered around 0. Although these two variables yield a slight improvement in R^2, they do not demonstrate a sufficiently strong

or stable independent contribution. Given the current data coverage, they serve more as weak auxiliary corrections than as core drivers capable of redefining the feature set.

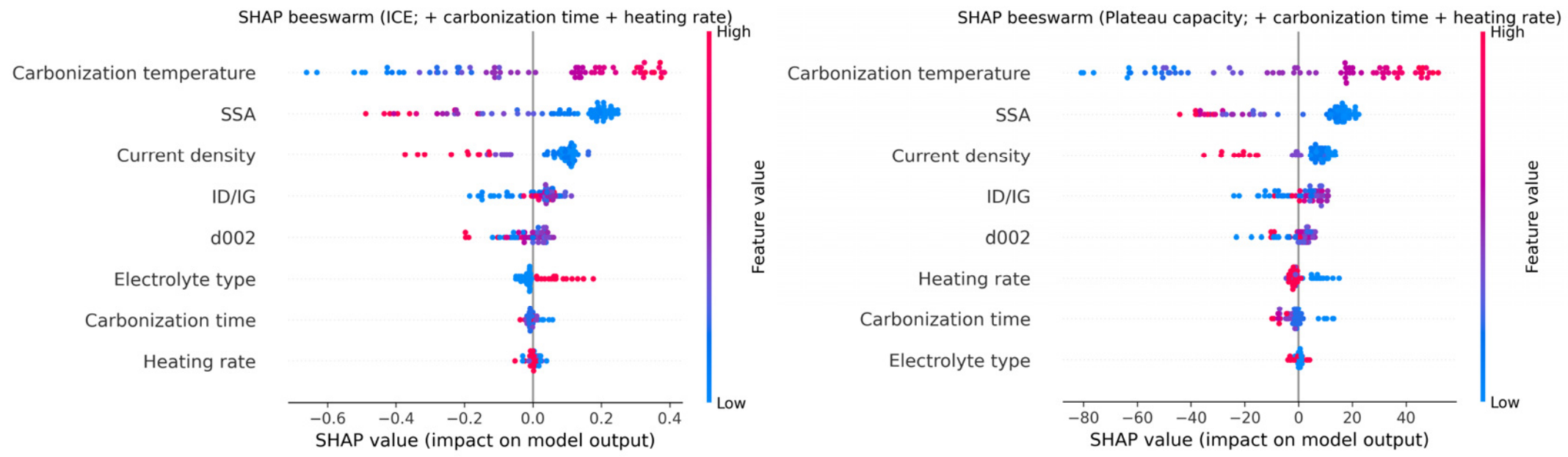

Figure R6. SHAP Beeswarm after incorporating carbonization time and heating rate into the RF model.

Although in this revision we did not simply incorporate these variables (carbonization time, heating rate, V_{total}, L_c, and L_a) into the main ML framework as a whole, we supplemented it with a more detailed mathematical analysis, including Pearson, Spearman, and Kendall correlation analyses between the missing variable and ICE, as well as reversible plateau capacity, correlation analyses between the missing variable and initial features, as well as among the variables themselves, univariate OLS with ICE and reversible plateau capacity as the dependent variable, and adjusted OLS after controlling for initial features. Analyze the statistical relationship between these variables with high missingness, potential multicollinearity, and marginal significance and the target variable using a combination of correlation, regression, and multicollinearity checks (**Figure R7**).

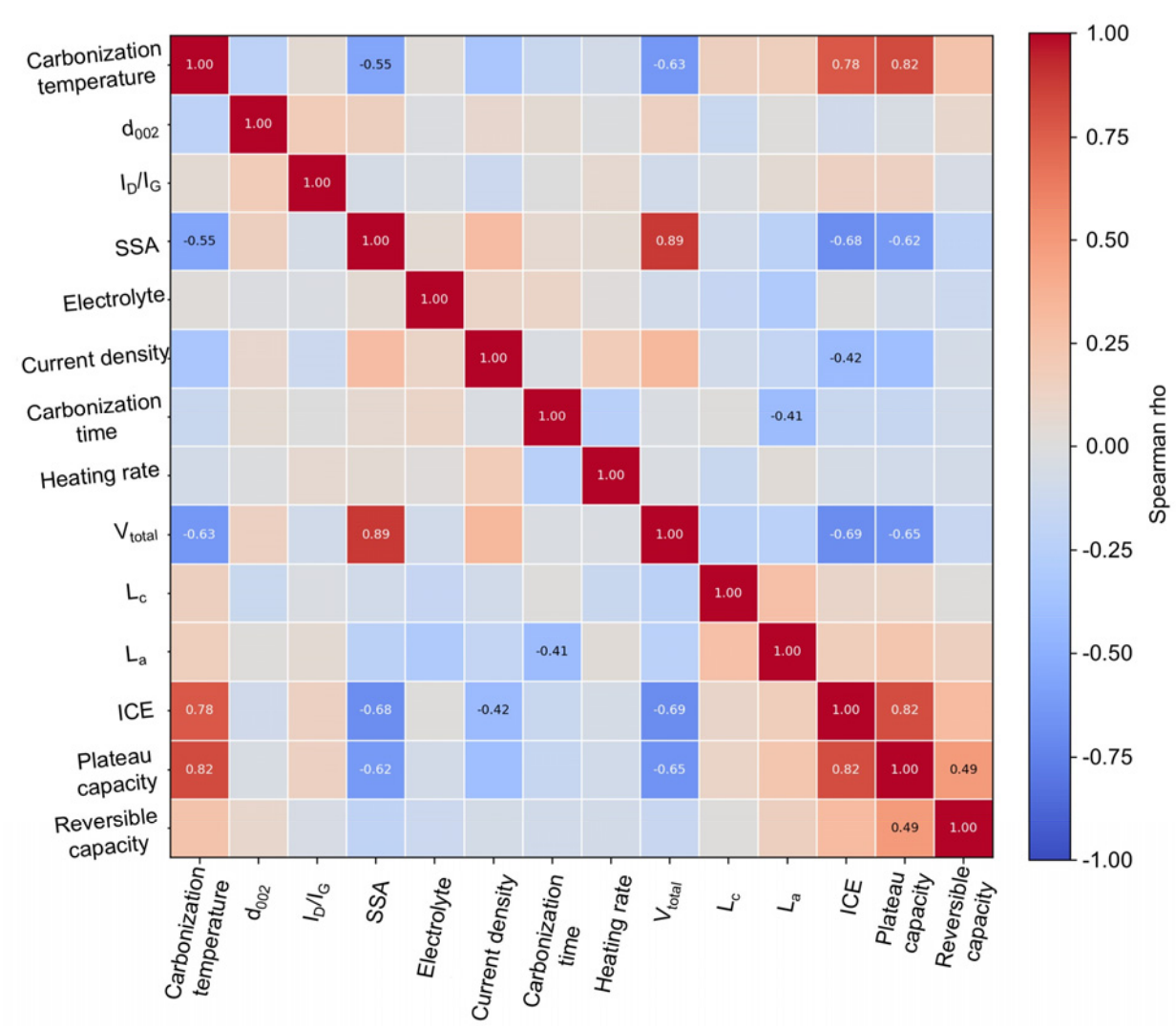

Figure R7. Spearman correlation heatmap between core features, missing variables, and the target variable.

The analysis results indicate that the directions of the three correlation coefficients are generally consistent, but there are significant differences in their intensity. Pore volume showed the strongest negative correlation with both target variables (**Figure R8**). For ICE, Pearson was -0.630, Spearman ρ was -0.693, and Kendall τ was -0.515. And for reversible plateau capacity, the values were -0.507, -0.654, and -0.483, respectively. The corresponding R^2 values for univariate OLS were 0.397 and 0.257. In contrast, carbonization time showed a weak negative correlation with the two target variables, while heating rate exhibited an even weaker correlation with them. The L_c and L_a showed primarily weak to moderate positive correlations with the two target variables, with L_a exhibiting a relatively stronger correlation with reversible plateau capacity (r = 0.227, ρ = 0.231, τ = 0.163). Analysis of the correlations among variables revealed that V_{total} was highly positively correlated with SSA (ρ = 0.886, τ = 0.707) and significantly negatively correlated with carbonization temperature (ρ = -0.627, τ = -0.476), indicating a clear overlap with existing core features. Adjusted OLS results further indicate that V_{total} remains significantly negatively correlated with ICE even after controlling for initial characteristics ($p = 6.4 \times 10^{-5}$), carbonization time remains significant only for reversible plateau capacity (p = 0.029), and heating rate is not significant for either of the two target variables.

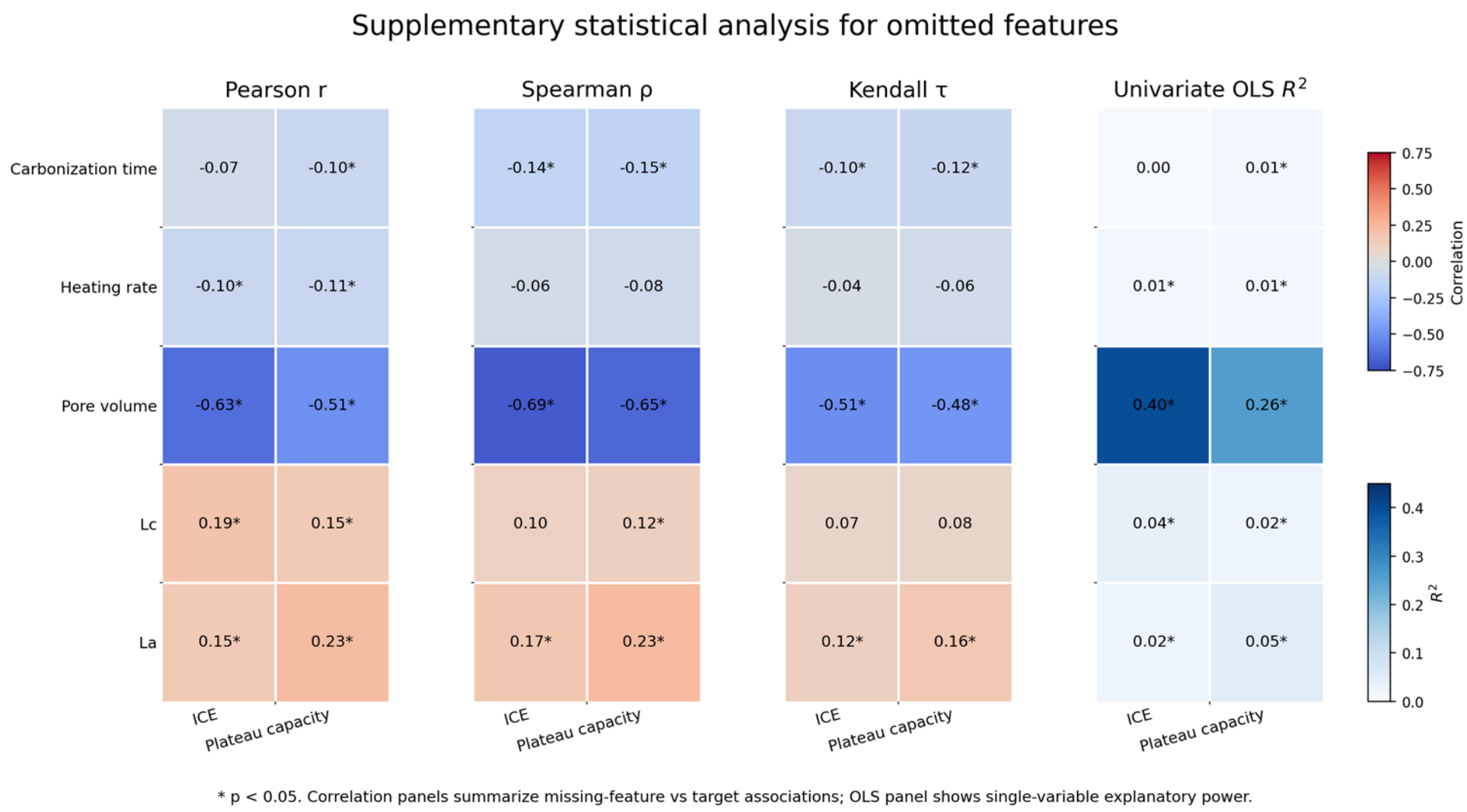

Figure R8. Pearson, Spearman, and Kendall correlations between missing variables and the target variable, and univariate explanatory power.

Therefore, given the current constraints of data coverage, independent information gain, and model stability, the additional variables are not yet sufficient to be elevated to the status of core

input variables in the ML model. In contrast, using it to supplement sensitivity analyses and statistical analyses provides a more accurate reflection of the current strength of the evidence. Among these, carbonization time and heating rate serve as priority candidate variables for tracking in subsequent data expansion, while V_{total}, L_c, and L_a are better suited as supplementary statistical evidence.

Our revision:

1. Added detailed description.

p 6, line 111-114 (Results)

Preparation parameters (carbonization temperature, carbonization time, and heating rate), microstructure parameters (d_{002}, I_D/I_G, SSA, total pore volume (V_{total}), medium basal coherence length (L_a), and crystal size in the stacking direction (L_c)), and performance parameters (ICE, reversible plateau capacity, and reversible capacity) are collected in the database by data mining from the massive literature.

p 6, line 117-120 (Results)

The carbonization temperature as a typical process parameter for regulating microstructure, the d_{002}, I_D/I_G, and SSA are crucial microstructure features for Na^+ diffusion, and ICE and reversible plateau capacity are key manifestations of reversible sodium storage sites (Supplementary Fig. 7, 8).

Supplementary Note 2 (Supplementary Information)

Compared to the carbonization temperature, the carbonization time and heating rate did not show a sufficiently strong and consistent independent contribution. Moreover, the proportion of missing values for V_{total}, L_c, and L_a is relatively high, and continuing to include them would rapidly reduce the number of complete samples and weaken the stability of the model. And there is significant multicollinearity among some of the variables, particularly, V_{total} overlaps significantly with SSA. Given current limitations in data coverage, independent information gain, and model stability, these variables (carbonization time, heating rate, V_{total}, L_c, and L_a) are not yet sufficient to serve as core input variables for machine learning (ML) models.

2. Added figures.

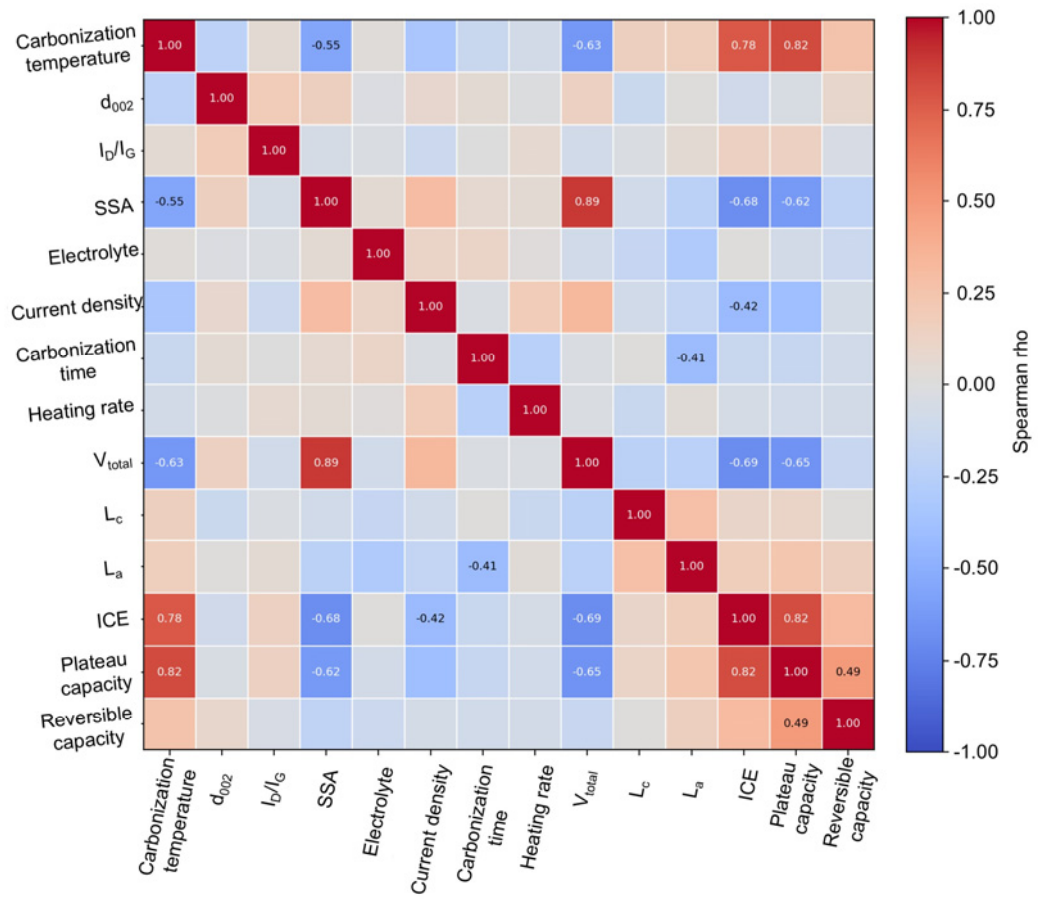

Supplementary Fig. 1. Spearman correlation heatmap between core features, missing variables, and the target variable.

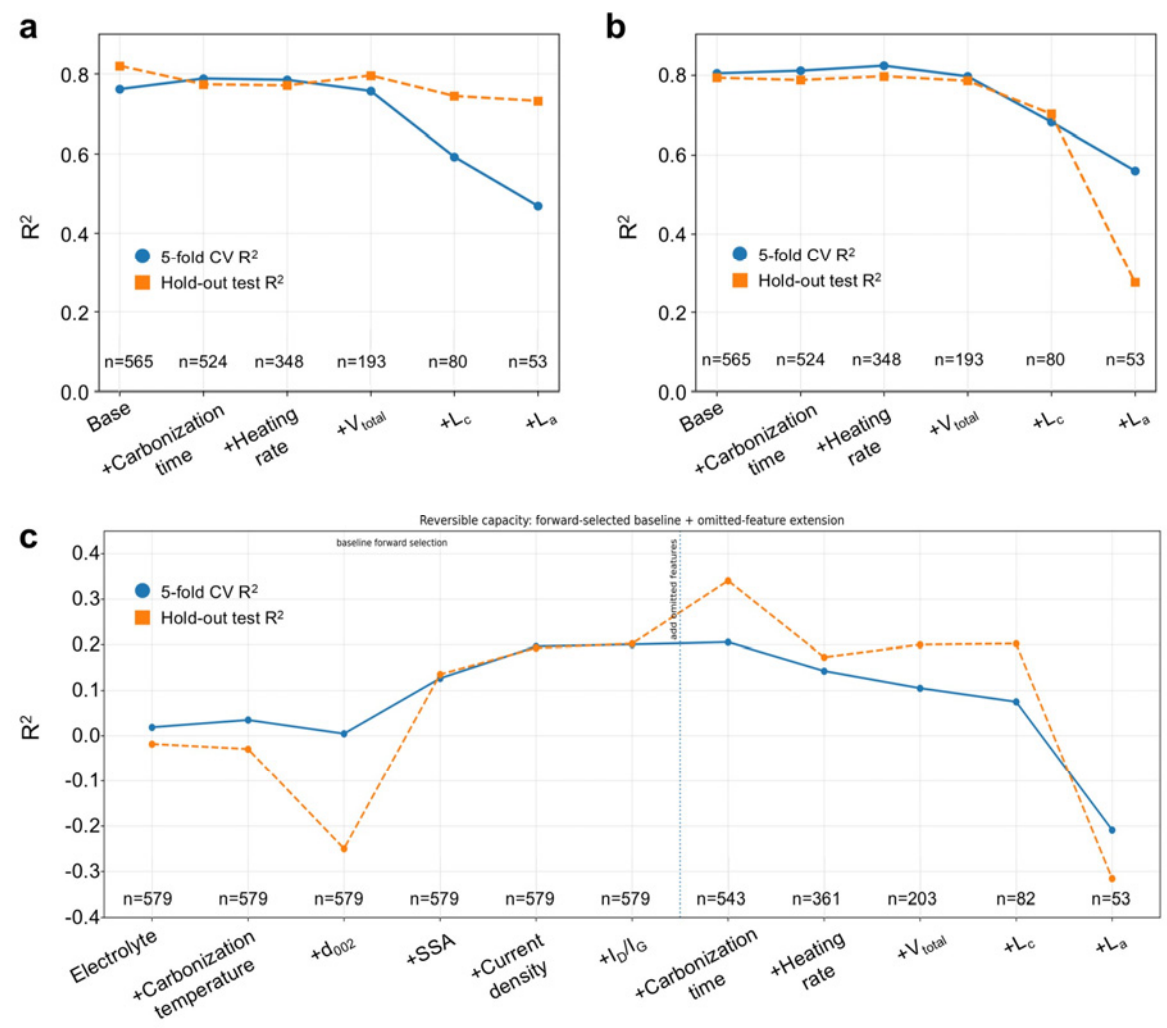

Supplementary Fig. 7. The R^2 change of the RF model after progressively adding missing variables in (a) ICE, (b) reversible plateau capacity, and (c) reversible capacity.

Comment #4. For work of high impact, such as this one, the K-fold cross-validation should be improved. Instead of 5-fold, can you switch it to 10-fold for us to see how it affects the analysis? The 8:2 training: testing is standard, so it is okay. But the validation can be improved to reduce bias.

Response: We deeply appreciate the insightful suggestions of reviewers for improving the robustness of the CV. Increasing the number of folds in K-fold CV from 5 to 10 is a crucial step in enhancing the rigor of ML research and strengthening the reliability of results. By increasing the proportion of the training set, the model can learn more detailed feature information. And by reducing the proportion of the test set, each sample is used more frequently in the test set, resulting in more stable evaluation results.

Table R2. Validation of consistency between 5-fold and 10-fold CV.

Task	5-fold CV R^2	5-fold test R^2	10-fold CV R^2	10-fold test R^2
ICE	0.763	0.826	0.767	0.826
Plateau capacity	0.805	0.794	0.805	0.797

To enhance the statistical reliability of the performance evaluation, especially given the limited dataset size typical of HC, we employed 10-fold CV (**Table R2**). The dataset was randomly partitioned into 10 equal subsets. In each iteration, 9 subsets were used for training and 1 for testing, repeated 10 times. The final performance metrics (R^2) were reported as the standard deviation over the 10 folds. To evaluate the robustness of the RF model, the results of 5-fold and 10-fold CV were first compared. After increasing the number of folds from 5 to 10, the CV R^2 of the RF model rose from 0.763 to 0.767 in ICE, while R^2 remained unchanged in reversible plateau capacity. The 5-fold and 10-fold CV results yielded similar R^2, demonstrating that the RF model exhibits excellent robustness, which is conducive to revealing the intrinsic physical relationships between the microstructure and electrochemical performance of HC.

Our revision:

1. Added detailed description.

p 6, line 124-126 (Results)

During the model evaluation phase, 5-fold cross-validation was used as the primary framework, supplemented by 10-fold cross-validation to confirm that the results were insensitive to the number of validation folds and demonstrated robustness[30].

2. Added figures and tables.

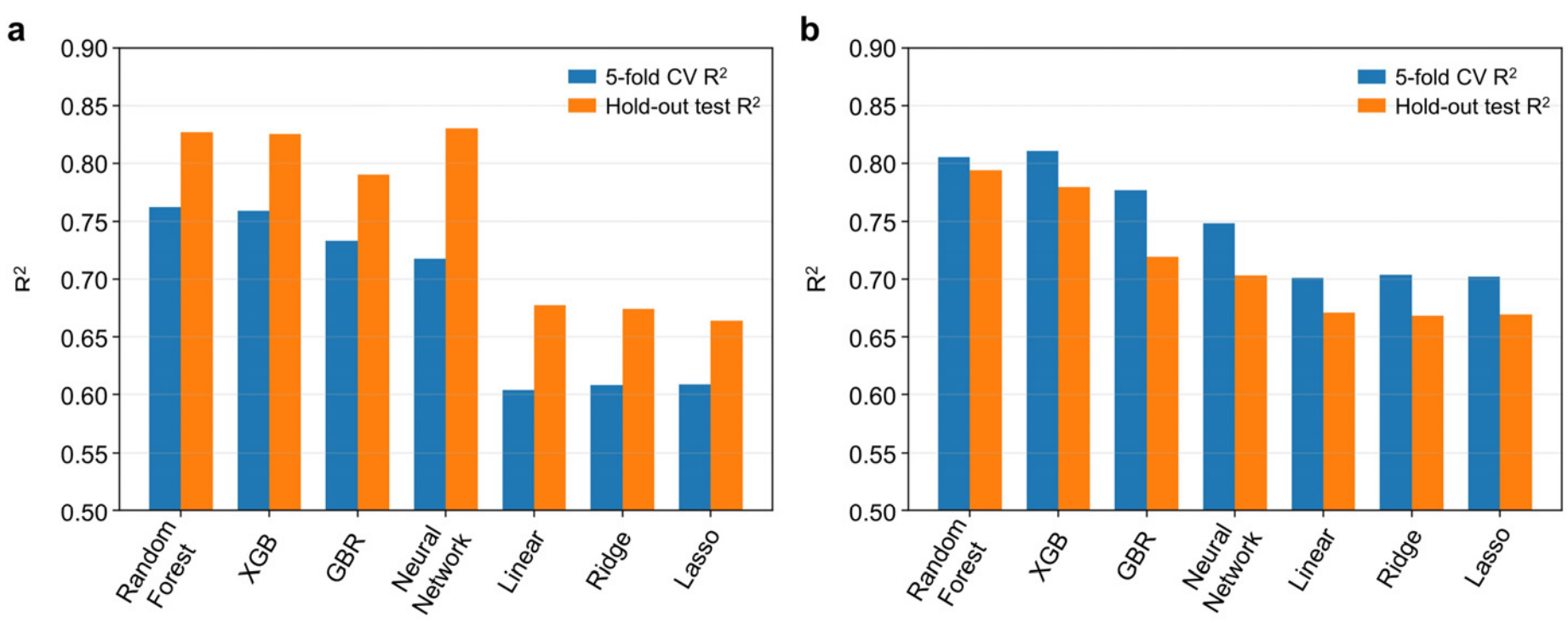

Supplementary Fig. 2. 5-fold cross-validation (CV) R^2 score of different regression models in (a) initial Coulombic efficiency (ICE) and (b) reversible plateau capacity.

Supplementary Table 2. The R^2 of the training and test sets of the ICE and reversible plateau capacity in the RF model.

Task	n	CV setting	CV R^2	Train-CV R^2	Test R^2	Test MAE	Test RMSE	CV setting	CV R^2	Test R^2
ICE	565	5-fold	0.763	0.764	0.826	0.257	0.311	10-fold	0.767	0.826
Plateau capacity	565	5-fold	0.805	0.806	0.794	26.93	33.40	10-fold	0.805	0.797

Comment #5. Can you explain how you adjusted the ML parameters to improve the accuracy of the findings?

Response: We sincerely appreciate the reviewers for pointing out the lack of transparency in ML methods. In ML research, tuning hyperparameters is a critical step in improving the predictive accuracy and robustness of a model. Since HC datasets are typically characterized by small sample sizes, high noise levels, and multi-parameter couplings, ML models must be selected with care, and parameters must be tuned until the model stabilizes to avoid overfitting and underfitting. Therefore, a strategy combining Bayesian optimization with grid search and random search is employed to tune parameters and compare various metrics under the optimal hyperparameters of different models, thereby obtaining the best model and optimal hyperparameters (**Table R3**).

Table R3. The hyperparameter search space for the main model.

Model	Hyperparameter search space
GBR (ICE)	n_estimators: 1–200; learning_rate: 0.01–1.0; max_depth: 1–10
GBR (plateau)	n_estimators: 1–100; learning_rate: 0.01–1.0; max_depth: 5–10
RF	n_estimators: 100–600; max_depth: 5–30; min_samples_split: 2–10; min_samples_leaf: 1–10; max_features: {sqrt, log2, None}; bootstrap: {True, False}
XGB	n_estimators: 1–150; max_depth: 5–10; learning_rate: 0.03–0.1 (log); subsample: 0.5–0.6; colsample_bytree: 0.6–1.0; reg_alpha / reg_lambda: 1e-5–1.0 (log)
Ridge	alpha: 0.01–10.0 (log)
Lasso	alpha: 0.01–10.0 (log)

In the ML algorithm calculations, seven regression models were evaluated for predictive analysis of HC, including Linear Regression, Gradient Boosted Regressor (GBR), Ridge Regression, Lasso Regression, Neural Network Regression, RF, and eXtreme Gradient Boosting (XGB). The data of the database were randomly divided into training and test sets in the ratio of 8:2. To improve model accuracy while preventing data leakage, hyperparameter optimization was performed exclusively on the training set using BayesSearchCV. The optimization objective was set to R^2, the random seed was fixed at 42, and the number of iterations was set to 20. During the model evaluation phase, a 5-fold CV was used as the primary framework, supplemented by a 10-fold CV to confirm that the results were insensitive to the number of validation folds and demonstrated robustness. In selecting the final model, this study did not rely solely on the highest R^2 values on the test set, but instead took into account a comprehensive balance of predictive performance, CV stability, the generalization gap, and model interpretability.

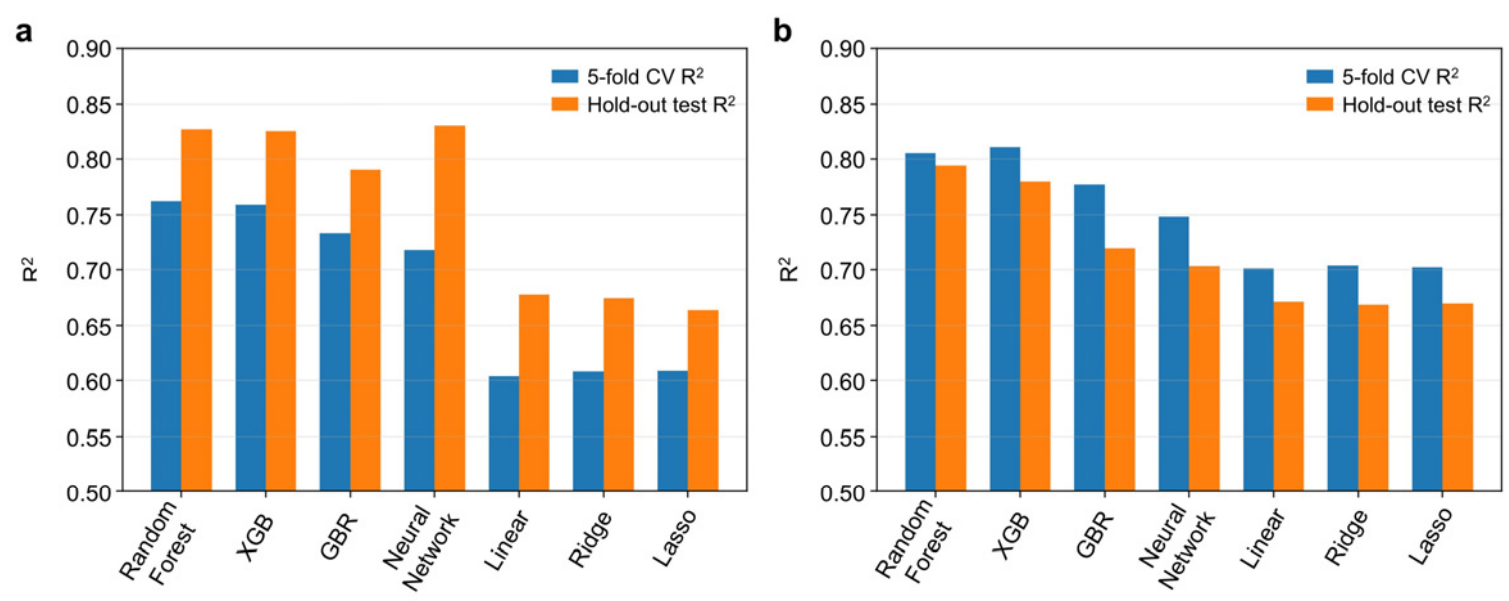

Figure R9. The performance comparison of multiple models in ICE and reversible plateau capacity.

In calculations of ICE, the 5-fold CV mean R^2 of RF, XGB, GBR, Neural Network, Linear, Ridge, and Lasso are 0.763, 0.759, 0.734, 0.718, 0.604, 0.608, and 0.608, respectively (**Figure R9a**). In contrast, the RF model exhibits the highest R^2 (0.826), indicating its strong regression performance. Besides, the RF model achieves an R^2 on the training set that is comparable to that of the XGB (0.825) and Neural Network (0.830) models and higher than that of other models. In calculations of reversible plateau capacity, the 5-fold CV mean R^2 of RF, XGB, GBR, Neural Network, Linear, Ridge, and Lasso are 0.805, 0.811, 0.777, 0.749, 0.701, 0.704 and 0.702, respectively (**Figure R9b**). Although the CV mean R^2 for RF (0.805) is slightly lower than that for XGB (0.811), the hold-out test R^2 for RF (0.794) is higher than that for XGB (0.780), resulting in a smaller generalization gap.

Compared to the other models, RF presented the most favorable performance. Besides, the small

difference in R^2 between the training and test sets indicates no overfitting for the RF model. More importantly, the RF model supports robust subsequent SHAP analysis, thereby addressing the need for cross-model interpretability. Based on this workflow, different RF models were developed for the two targets, and a preferred structure window that benefits both ICE and reversible plateau capacity was further identified through SHAP analysis.

Our revision:

1. Added detailed description.

p 6, line 120-124 (Results)

In the ML algorithm calculations, the data of the database were randomly divided into training and test sets in the ratio of 8:2, and hyperparameter optimization was performed exclusively on the training set using BayesSearchCV[29]. The optimization objective was set to R^2, the random seed was fixed at 42, and the number of iterations was set to 20.

p 7, line 132-136 (Results)

In calculations of ICE, the Random Forest (RF) model exhibits the highest 5-fold cross-validation mean R^2 (0.826), indicating strong regression performance (Supplementary Fig. 2). In calculations of reversible plateau capacity, the 5-fold cross-validation mean R^2 for RF (0.805) is slightly lower than that for eXtreme Gradient Boosting (XGB, 0.811), the hold-out test R^2 for RF (0.794) is higher than that for XGB (0.780), resulting in a smaller generalization gap (Table S1).

p 7, line 138-140 (Results)

Therefore, comprehensively evaluating the R^2 value, predictive performance, cross-validation stability, generalization gap, and interpretability, the RF model was selected as the final model.

2. Added figures and tables.

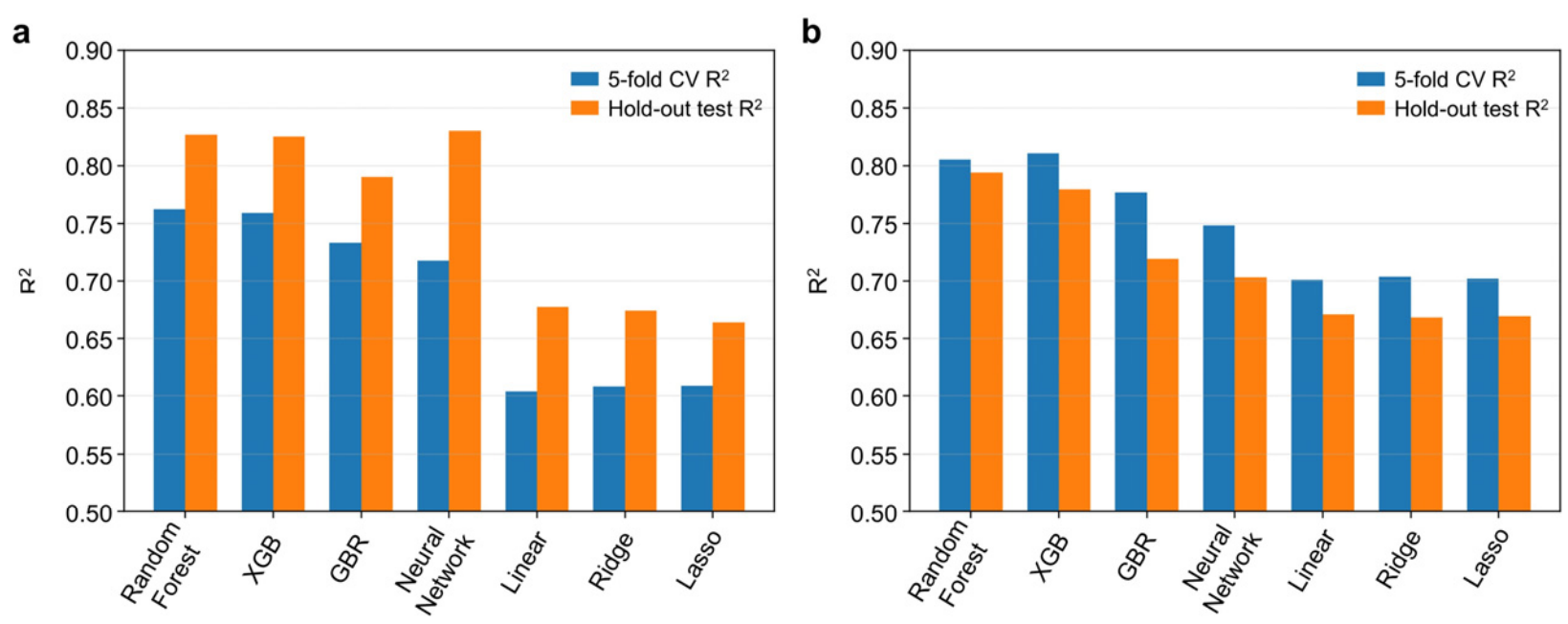

Supplementary Fig. 2. 5-fold cross-validation (CV) R^2 score of different regression models in (a) initial Coulombic efficiency (ICE) and (b) reversible plateau capacity.

Supplementary Table 1. The performance, generalization gaps, and interpretability comparison of multi-models.

Task	Model	5-fold CV mean R^2	5-fold CV std	Test R^2	Generalization gap	SHAP analysis
ICE	Random Forest	0.763	0.019	0.826	-0.062	Yes
	XGB	0.759	0.024	0.825	-0.062	Yes
	GBR	0.734	0.037	0.790	-0.052	Yes
	Neural Network	0.718	0.055	0.830	-0.106	No
	Linear	0.604	0.131	0.678	-0.062	Yes
	Ridge	0.608	0.136	0.674	-0.053	Yes
	Lasso	0.608	0.147	0.664	-0.041	Yes
Plateau capacity	Random Forest	0.805	0.041	0.794	0.012	Yes
	XGB	0.811	0.041	0.780	0.031	Yes
	GBR	0.777	0.041	0.719	0.060	Yes
	Neural Network	0.749	0.042	0.703	0.045	No
	Linear	0.701	0.047	0.671	0.030	Yes
	Ridge	0.704	0.047	0.668	0.036	Yes
	Lasso	0.702	0.047	0.669	0.033	Yes

Comment #6. Did the ranking obtained with SHAP match literature findings? Can you also do feature ablation ranking to confirm the SHAP results?

Response: We are very grateful for the suggestions of reviewers regarding the reliability of SHAP interpretations. In this work, carbonization temperature, SSA, and current density were found to be the primary contributors to ICE and reversible plateau capacity in the SHAP analysis of the RF model. Meanwhile, d_{002} and I_D/I_G maintained moderate contributions. These conclusions align with both experimental results and ML findings reported in the literature.

Experimental results: In numerous experimental studies, the carbonization temperature has always been a key and effective process parameter for modulating the microstructure of HC. As the carbonization temperature increases, the microstructure of HC tends toward graphitization, and the SSA decreases rapidly (***Adv. Mater.* 2025,** 37, 36, 2506066). This is the key structure basis for improving ICE and reversible plateau capacity. Since d_{002} and I_D/I_G simultaneously affect both

sodium ion diffusion and reversible storage, values that are too high or too low for either parameter make it difficult to achieve both a high ICE and a high reversible plateau capacity. The carbonization temperature and SSA show positive and negative correlations with the two performance indicators, respectively, and their relative contributions are higher than those of d_{002} and I_D/I_G, which is consistent with our ML findings.

Machine learning findings: ML is increasingly being used to uncover the relationship between the microstructure and electrochemical performance of HC. In particular, the SHAP analysis of RF, Gradient Boosting, Adaboost, Linear Regression, and XGBoost models determines the weights of each feature relative to the target value (***RSC Sustain. 2025,*** 3, 7, 3133-3143). In XGBoost models, the SSA shows a strong correlation with ICE and reversible capacity compared to other structure parameters (***J. Colloid Interface Sci. 2025,*** 692, 15, 137474). Besides, for capacity prediction, carbonization temperature is the most influential variable, accounting for 28.7% of the total importance, highlighting a dominant role in capacity regulation. Most positive SHAP values are linked to high-temperature samples, indicating that higher carbonization temperatures improve the predicted capacity. Regarding the I_D/I_G and d_{002}, both high and low values appear across the SHAP axis, while the strongest positive contributions are concentrated in the middle range, suggesting that moderate structural disorder offers an optimal balance between sodium storage sites and electronic transport pathways (***J. Energy Chem. 2026,*** 117, 70-80). This is consistent with the ranking of feature contributions to the target value obtained from the SHAP analysis of the RF model in ML.

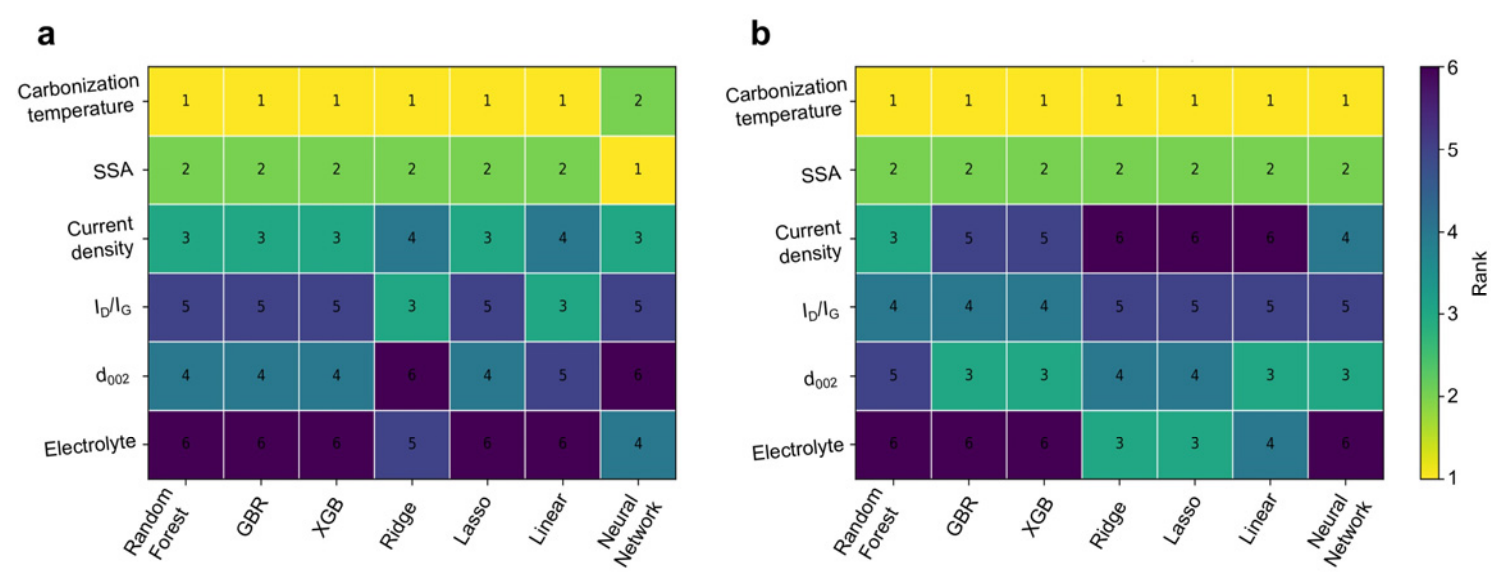

Figure R10. The heatmap of SHAP ranking consistency across different models in (a) ICE and (b) reversible plateau capacity.

We further conducted a cross-model SHAP ranking comparison of GBR, XGB, Ridge, and locked RF to analyze the contribution of each feature to the ICE and reversible plateau capacity, respectively. For ICE, the contributions of carbonization temperature and SSA consistently rank

among the top factors across different models, while current density also remains at a consistently high level of importance (**Figure R10a**). For reversible plateau capacity, carbonization temperature, and SSA play a consistently dominant role across different models (**Figure R10b**). Moreover, d_{002} and I_D/I_G both contribute moderately in different models for ICE and reversible plateau capacity. Notably, the ranking of features by their contribution to ICE and reversible plateau capacity remains consistent in the RF model.

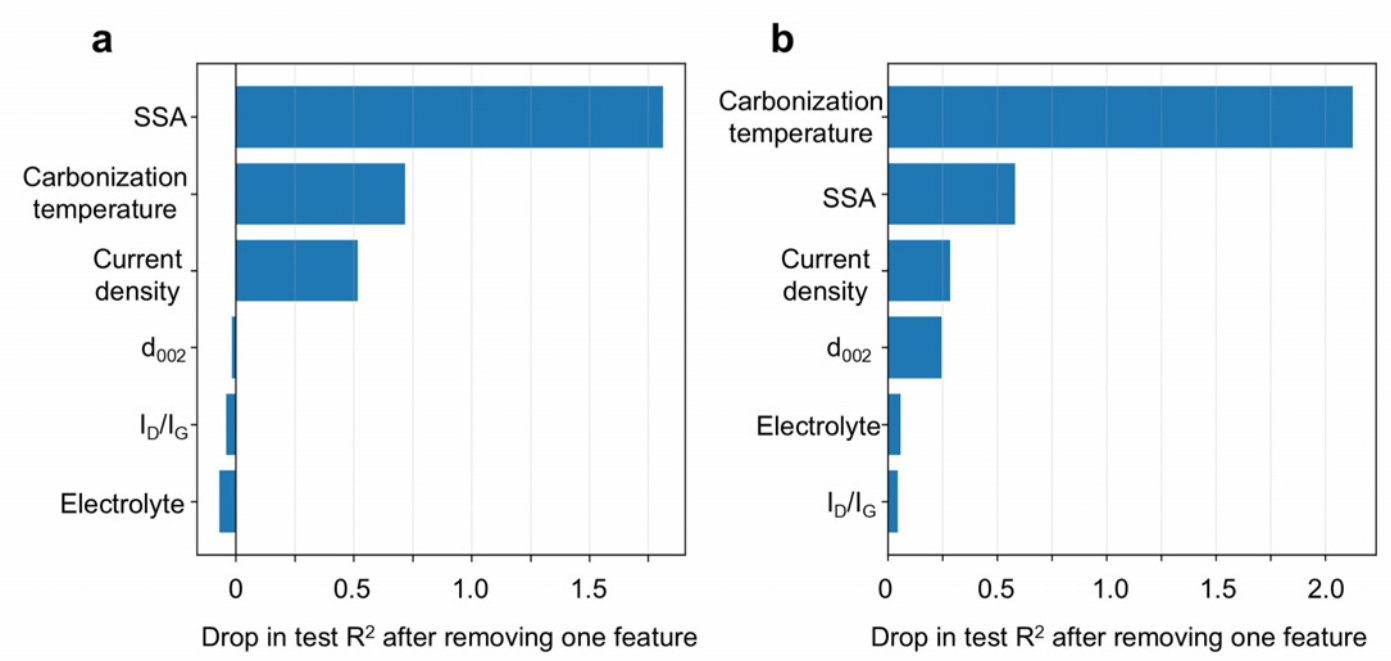

Figure R11. Robustness results for the leave-one-feature-out of the locked RF in (a) ICE and (b) reversible plateau capacity.

We have added permutation importance and leave-one-feature-out (feature ablation) analyses based on locked RF. For ICE, in the leave-one-feature-out analysis of locked RF, removing SSA causes the test R^2 to decrease by 0.181, removing carbonization temperature causes it to decrease by 0.072, and removing current density causes it to decrease by 0.052, which is highly consistent with the main ranking in SHAP (**Figure R11a**). For reversible plateau capacity, in the leave-one-feature-out analysis of locked RF, carbonization temperature is the most influential factor, removing this feature causes the test R^2 to decrease by 0.213 (**Figure R11b**). SSA is the next most influential factor, followed by current density and d_{002} as the second-tier variables. Therefore, through dual validation via SHAP interpretive analysis and feature ablation experiments, the reliability of the key influencing factors for various electrochemical performance metrics has been enhanced.

Our revision:

1. Added detailed description.

p 7, line 136-138 (Results)

Besides, the ranking of features by their contribution to ICE and reversible plateau capacity remains consistent in the RF model by a cross-model Shapley Additive exPlanations (SHAP) ranking comparison (Supplementary Fig. 3).

<u>*Supplementary Note 1 (Supplementary Information)*</u>

For ICE, in the leave-one-feature-out analysis of the locked RF model, removing SSA causes the test R^2 to decrease by 0.181, removing carbonization temperature causes it to decrease by 0.072, and removing current density causes it to decrease by 0.052, which is highly consistent with the main ranking in SHAP. For reversible plateau capacity, in the leave-one-feature-out analysis of the locked RF model, carbonization temperature is the most influential factor, removing this feature causes the test R^2 to decrease by 0.213. SSA is the next most influential factor, followed by current density and d_{002} as the second-tier variables. Therefore, through dual validation via SHAP interpretive analysis and feature ablation experiments, the reliability of the key influencing factors for various electrochemical performance metrics has been enhanced.

2. Added figures.

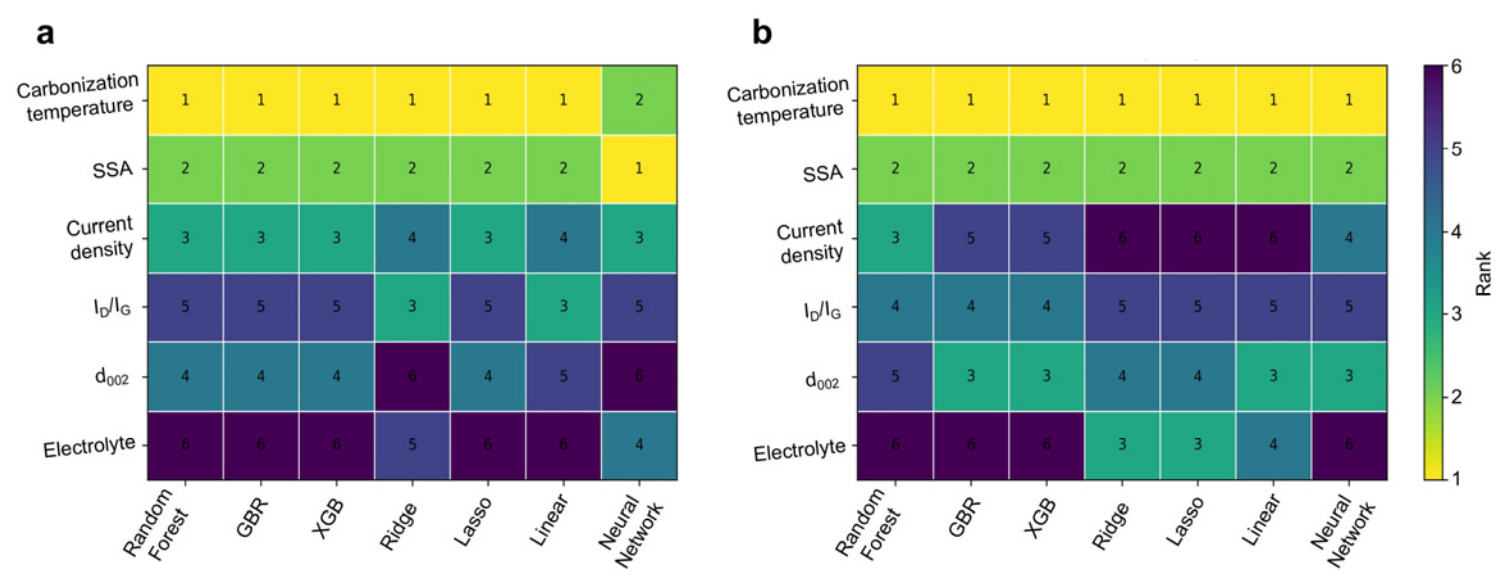

Supplementary Fig. 3. The heatmap of Shapley Additive exPlanations (SHAP) ranking consistency across different models in (a) ICE and (b) reversible plateau capacity.

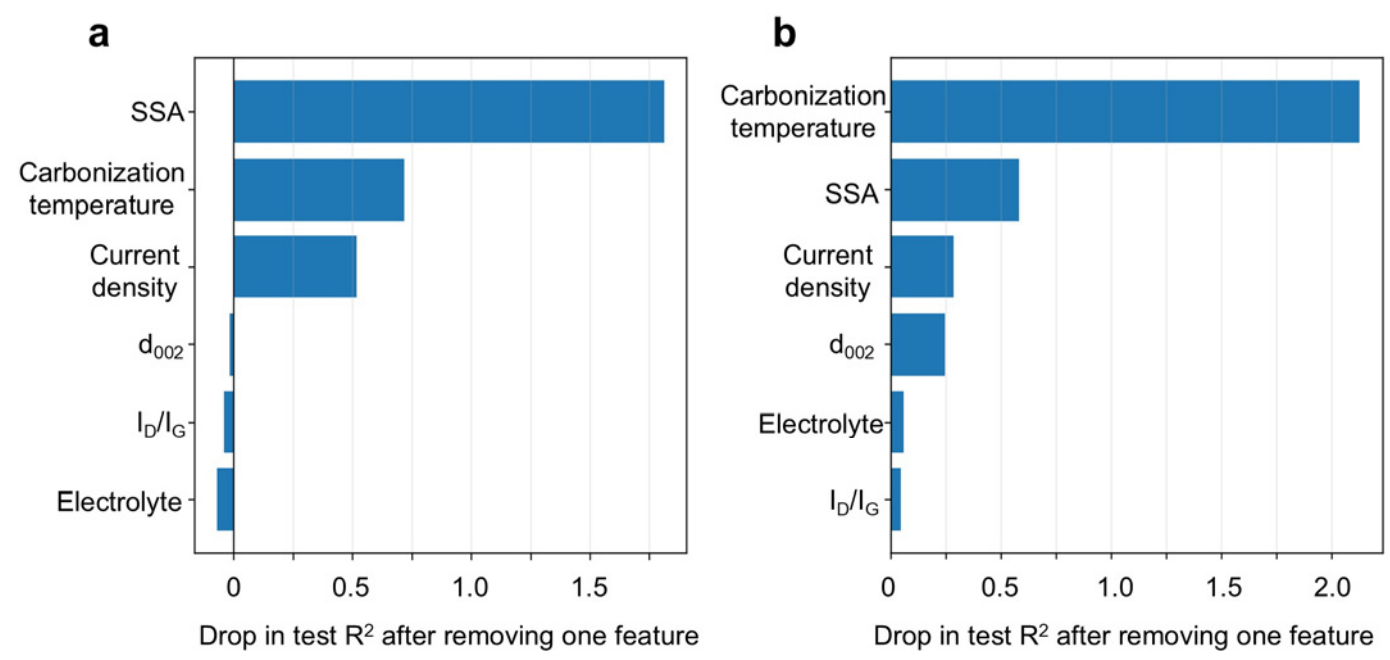

Supplementary Fig. 6. Robustness results for the leave-one-feature-out of the locked RF model in (a) ICE and (b) reversible plateau capacity.

Comment #7. ML was primarily used in this work to identify high-performing microstructures. Since a lot of data is available from the literature, and looking at the scope and high impact of this journal, it will be useful to use ML to predict the reversible capacities of the HC's as well. If the predictions match the experimental data obtained, that can further authenticate the ML analysis. For more information on how to go about that, you can refer to this latest work.

(https://pubs.rsc.org/en/content/articlehtml/2025/su/d5su00360a)

Response: We sincerely appreciate the valuable suggestions of the reviewer regarding the relationship between the microstructure and reversible capacity of HC. Reversible capacity, along with ICE and reversible plateau capacity, is a key performance parameter for evaluating the reversible storage performance of HC. We agree that independently modeling reversible capacity within a framework consistent with the main model would help further assess the information upper bound of the current literature-derived dataset and evaluate the applicability of the input features in this paper to another prediction objective.

Table R4. Modeling results for the added reversible capacity.

Metric	n	5-fold CV R^2	5-fold CV R^2 std	Train-CV R^2	Hold-out test R^2	Test MAE	Test RMSE
Value	579	0.201	0.053	0.197	0.206	46.22	58.99

A supplementary modeling task for reversible capacity has been added in this revision, strictly adhering to the original modeling strategy. First, the original feature parameters (Carburetion temperature, Current density, Electrolyte, d_{002}, I_D/I_G, and SSA) were retained, and the data were split into training and testing sets in an 8:2 ratio. RF parameters were optimized only within the training set using BayesSearchCV, and model performance was evaluated using both 5-fold CV and the independent testing set. The results indicate that the correlation between eigenvalues and reversible capacity is significantly more challenging compared to ICE and reversible plateau capacity. Using the RF algorithm yielded a 5-fold CV mean R^2 of 0.201 ± 0.053, a train-CV R^2 of 0.197, a hold-out test R^2 of 0.206, a test MAE of 46.22 mAh g^{-1}, and a test RMSE of 58.99 mAh g^{-1} (**Table R4**).

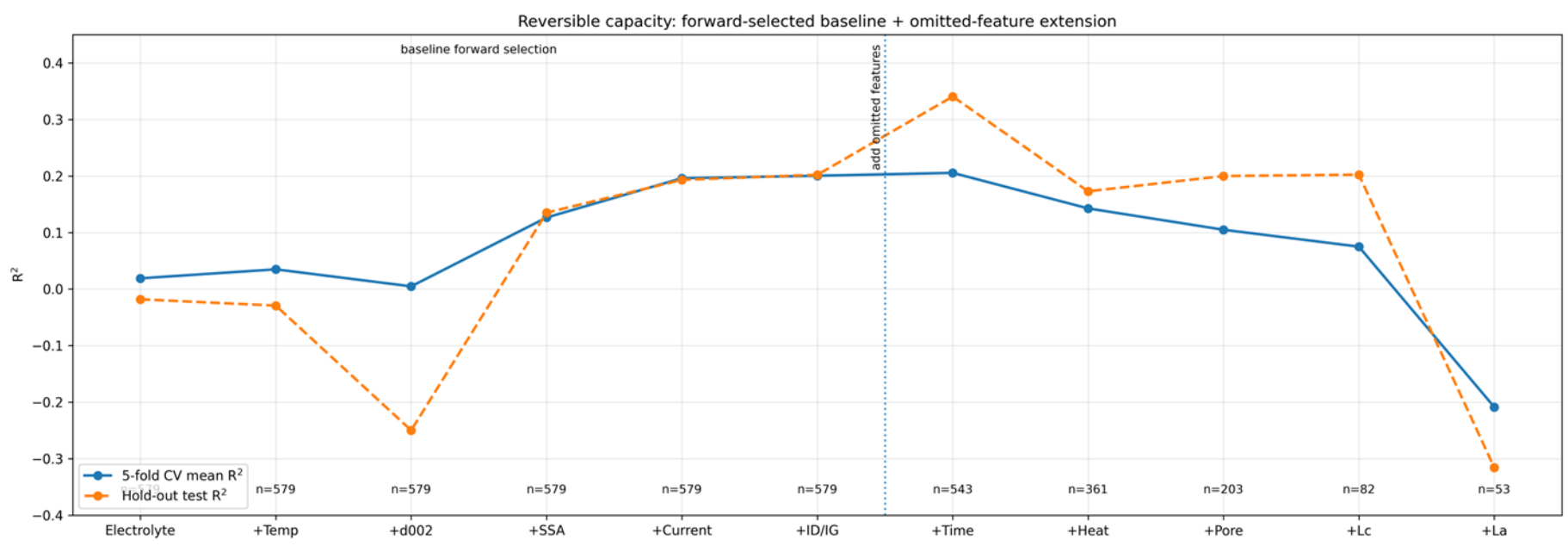

Figure R12. The change in R^2 after progressively adding missing variables in reversible capacity.

We conducted a sensitivity analysis with gradual incorporation of eigenvalues based on the key

data from the reversible capacity. In ascending order of missing rate, carbonization time, heating rate, V_{total}, L_c, and L_a were added sequentially (**Figure R12**). At each step, remove samples with missing values from the current feature set and recalculate the 5-fold CV and hold-out test performance using the RF parameters locked for the original task. The results show that added carbonization time, the 5-fold CV R^2 increased to 0.206, and decreased to 0.143 after adding heating rate. However, when V_{total}, L_c, and L_a were further included, the 5-fold CV R^2 subsequently decreased to 0.105, 0.075, and -0.209, respectively. This result suggests that reversible capacity should not be overemphasized in the current version as a criterion for validating the conclusions of the main model.

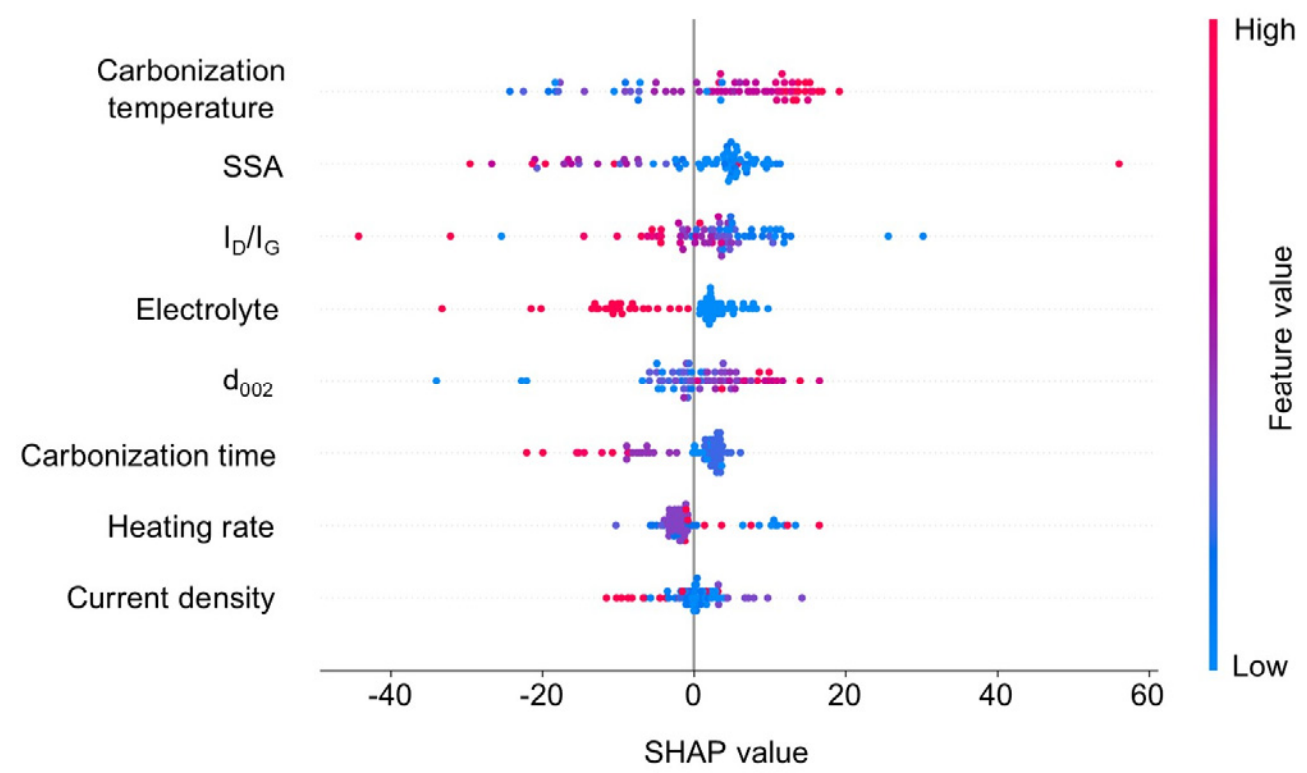

Figure R13. SHAP Beeswarm after incorporating carbonization time and heating rate into the RF model.

The main reason for the unclear correlation between the characteristic value and reversible capacity is that reversible capacity is contributed by two components: the slope region and the plateau region (**Figure R13**). In the slope region, Na^+ is adsorbed onto the defects, pore surfaces, and edge structures of HC. The high number of defects, SSA, and pore volume provide a large number of adsorption sites, thereby enabling a high sodium storage capacity. However, this structure also results in small L_a, L_c, and closed-pore volume due to the low carbonization temperature. In the plateau region, Na^+ is stored in the layers and closed pores of HC through insertion and filling behaviors, respectively. High L_a, high L_c, and high closed-pore volumes provide a large number of active sites, enabling high sodium storage capacity. However, the structure also exhibits low SSA, low d_{002}, low pore volume, and low defect due to the high carbonization temperature. Various structure parameters contribute to reversible capacity, meaning that a single structure parameter fails

to independently explain changes in reversible capacity. There is significant multicollinearity among the variables, making it difficult for the model to disentangle the independent contributions of these correlated variables, which results in low correlation.

Our revision:

1. Added detailed description and references.

Supplementary Note 2 (Supplementary Information)

The main reason for the unclear correlation between the characteristic value and reversible capacity is that reversible capacity is contributed by two components: the slope region and the plateau region. Various structure parameters contribute to reversible capacity, meaning that a single structure parameter fails to independently explain changes in reversible capacity. There is significant multicollinearity among the variables, making it difficult for the model to disentangle the independent contributions of these correlated variables, which results in low correlation.

p 31, line 669-670 (References)

[31] Owusu, S. Y. Machine learning prediction of the reversible capacities of a biomass-derived hard carbon anode for sodium-ion batteries. *RSC Sustain.* **3**, 3133-3143 (2025).

2. Added figures.

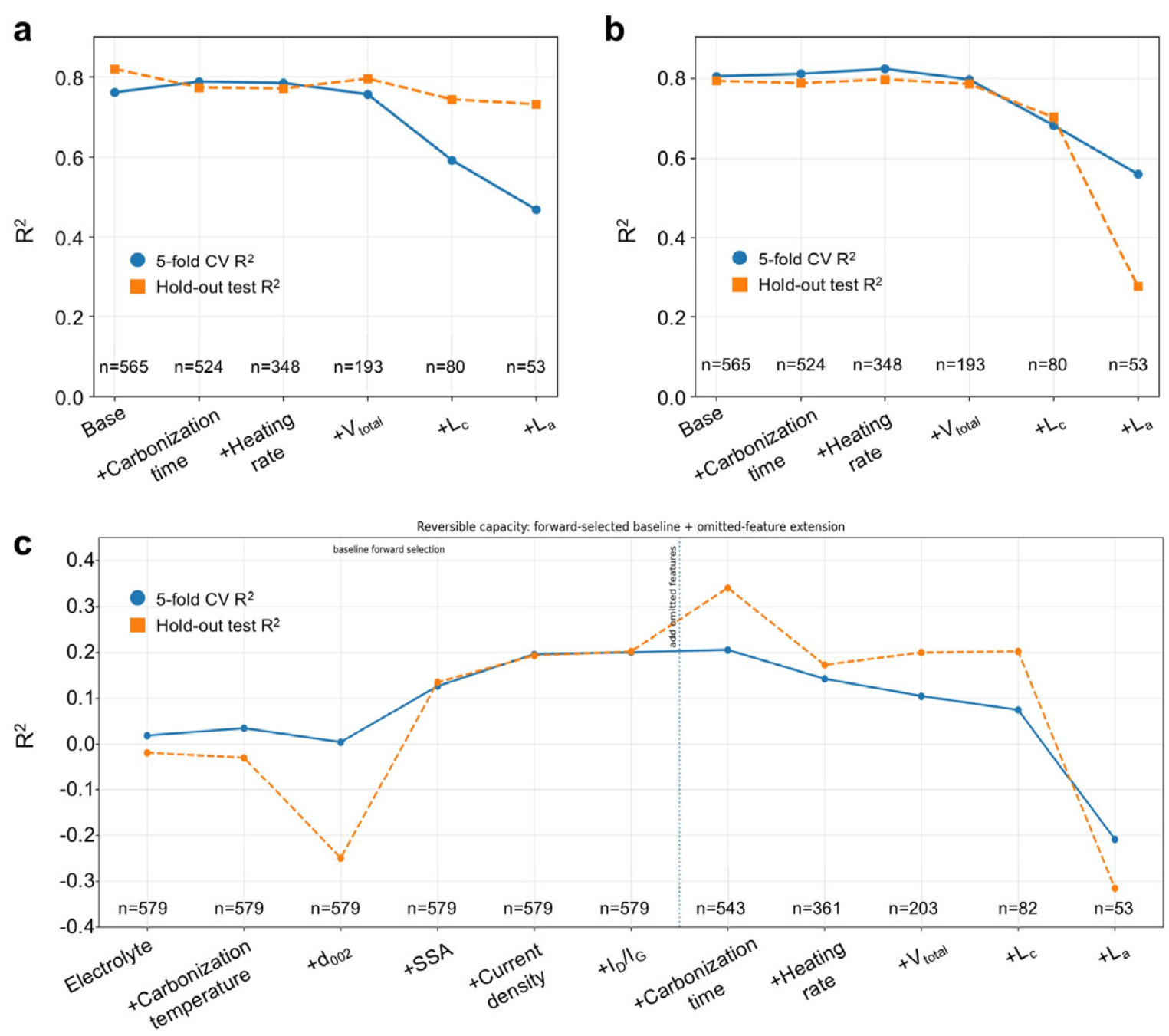

Supplementary Fig. 7. The R^2 change of the RF model after progressively adding missing variables in (a) ICE, (b) reversible plateau capacity, and (c) reversible capacity.

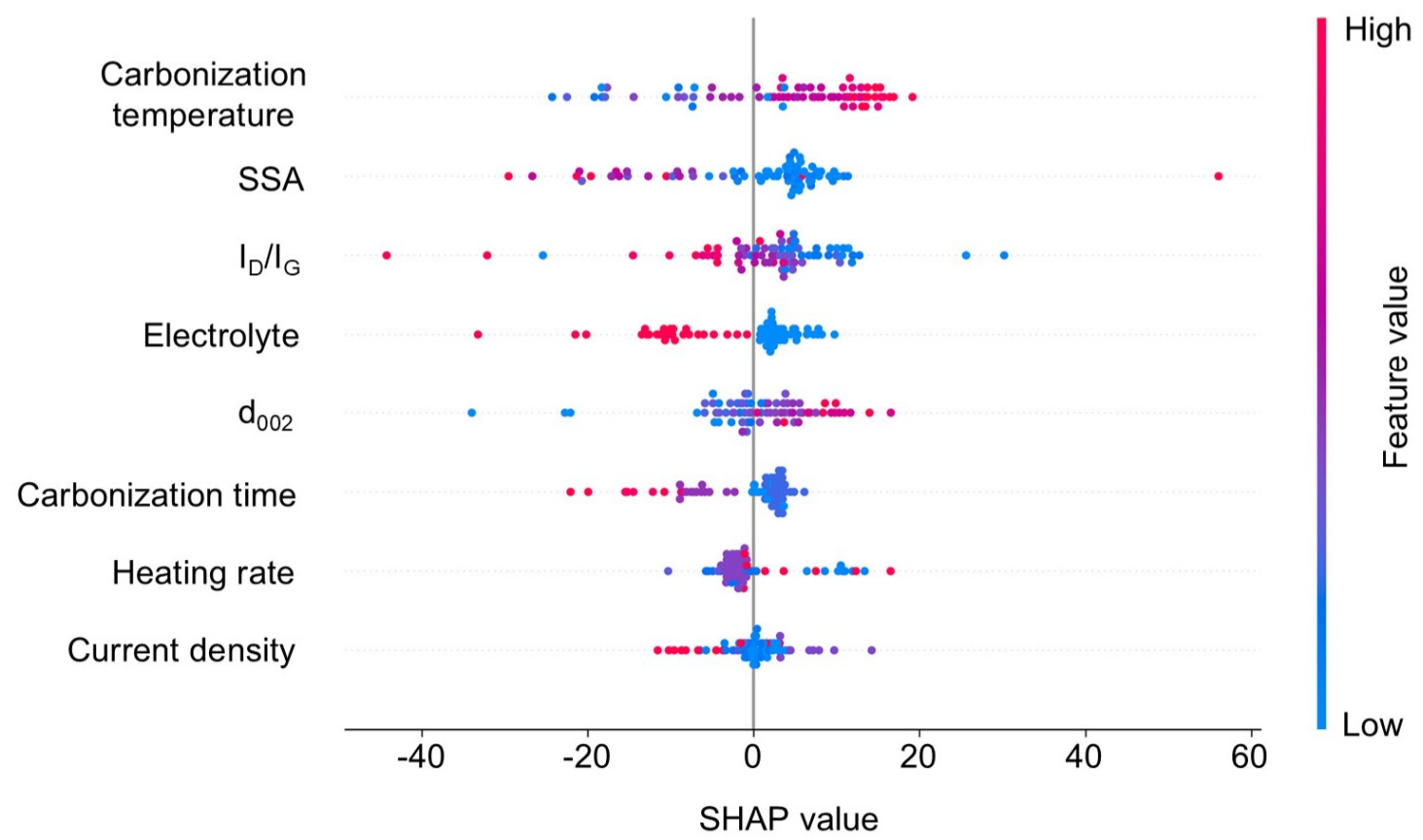

Supplementary Fig. 8. The SHAP analysis honeycomb diagram of reversible capacity.

Comment #8. Can you expand on the chemistry of the synthesis or suggest a mechanism for the transition of glucose into the HC? Is it only water that is lost during pyrolysis? Can we know the chemical structure of the HC? The XPS of HC shows the presence of some functional groups. Can you check the XPS of as-made samples and samples at 300 degrees Celsius also? So that there can be a direct comparison of the transition of the chemical composition along pyrolysis.

Response: We appreciate this insightful and forward-looking question. Glucose is the most common carbohydrate precursor for preparing HC. There is a strong correlation between the chemical composition of the intermediate products during the carbonization process of glucose and the microstructure of the resulting HC.

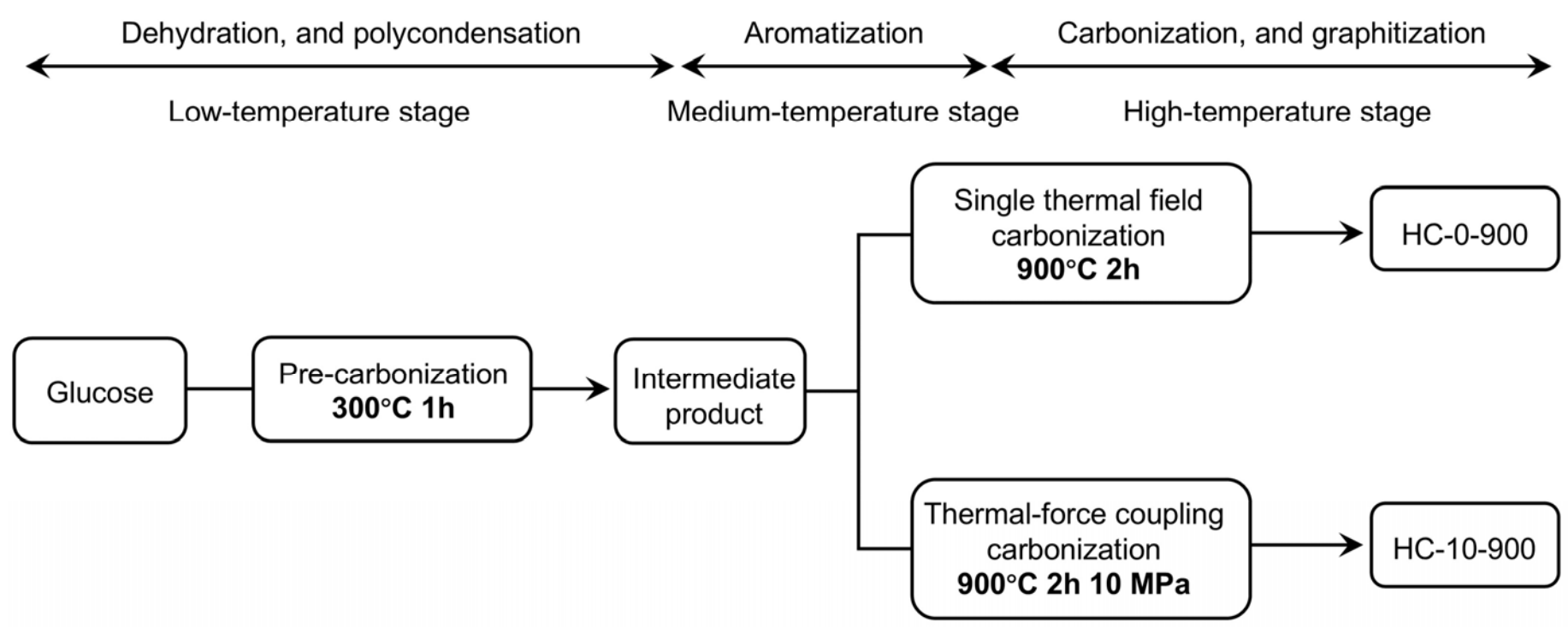

Figure R14. The carbonation process and chemical reactions of glucose.

The reaction mechanism of glucose synthesis in HC. In this study, glucose was first subjected to pre-carbonization at a low temperature of 300°C, followed by direct heating carbonization and thermal-force coupling carbonization, respectively, to produce the HC-0-900 and HC-10-900

(**Figure R14**). The process by which glucose is converted into HC involves dehydration and polycondensation, aromatization, carbonization, and graphitization reactions (***ACS Appl. Energy Mater. 2023,*** 6, 1, 181−190; ***Adv. Mater. 2021,*** 33, 40, 2102796). **Low-temperature stage:** Intermolecular dehydration occurs in the hydroxyl groups of glucose, producing caramel, which is an amorphous, oxygen-rich polymer. During this stage, the material begins to darken and gradually shrinks in volume. **Medium-temperature stage:** As the temperature rises, C-O bonds break, releasing gases such as CO_2 and H_2O. The fatty acid chains break down, gradually forming polycyclic aromatic hydrocarbon structures. **High-temperature stage:** The disordered carbon structure undergoes an ordered rearrangement, gradually increasing the size of the sp^2 carbon regions, and the structure tends toward stability. Compared to direct heating carbonization, thermal-force coupling carbonization increases the density of the carbon framework during the medium-temperature stage, inhibiting the cleavage of carbon-oxygen bonds, thereby reducing gas escape. During the high-temperature stage, pressure promotes the planar orientation and stacking of the carbon layers, further increasing their size, thereby enhancing the graphitization degree of the structure.

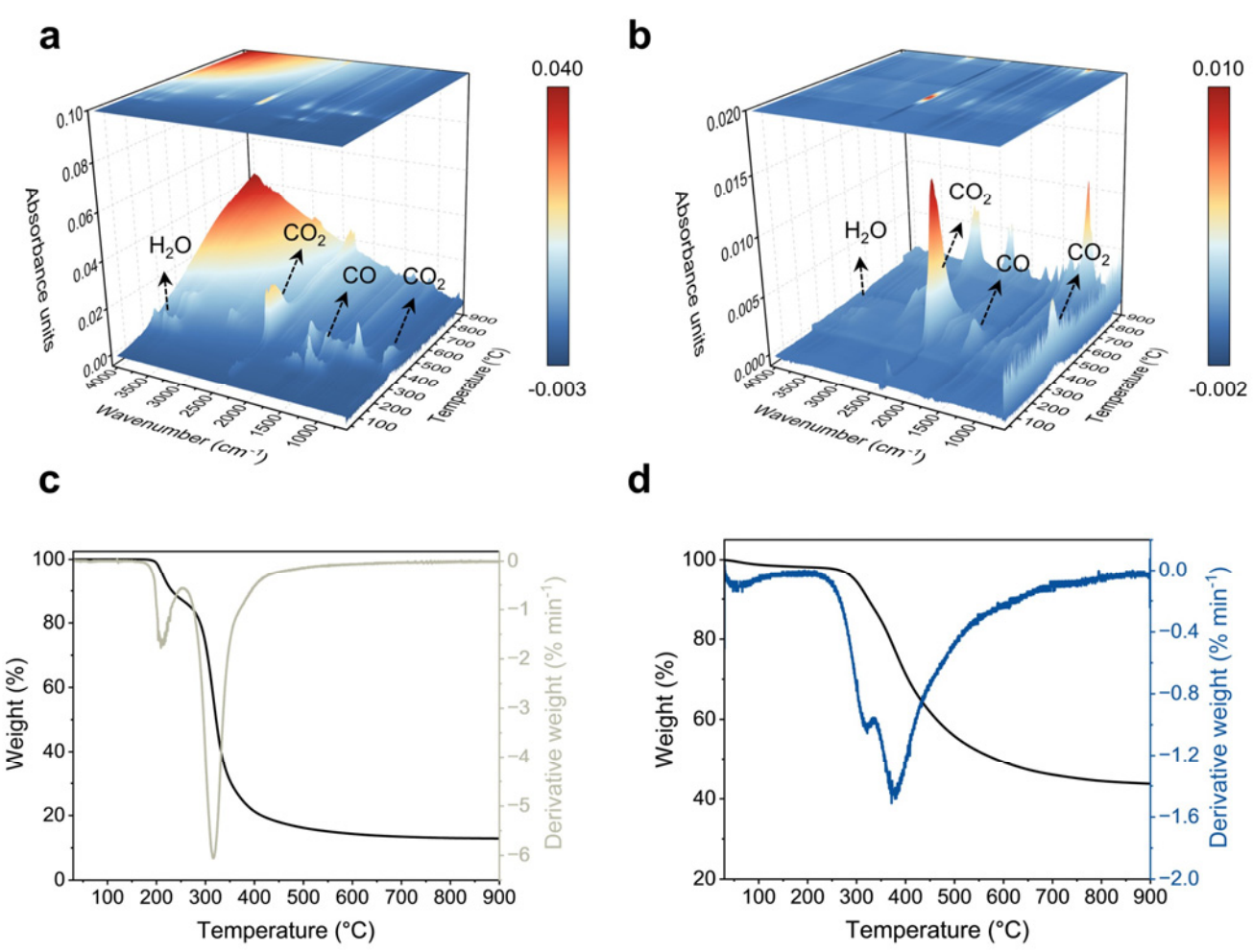

Figure R15. In situ thermogravimetry analysis-Fourier transform infrared (TGA-FTIR) of (a, c) the direct pyrolysis process of glucose and (b, d) the pyrolysis process of glucose after pre-carbonization.

Substance loss during the pyrolysis process: Throughout the pyrolysis process, the gases produced by the decomposition of glucose are highly complex. The H_2O is primarily formed during the low-temperature stage as a result of the dehydration reaction between hydroxyl and carboxyl groups. As the pyrolysis temperature rises, CO_2 and CO gases are gradually formed, which is

attributed to the breaking of carbon-oxygen bonds and the decomposition of oxygen-containing functional groups (**Figures R15a, c**). The pyrolysis of glucose undergoes intense deoxygenation and dehydrogenation reactions, accompanied by the release of large amounts of gas, resulting in a low carbonization yield of final HC (**Figures R15b, d**).

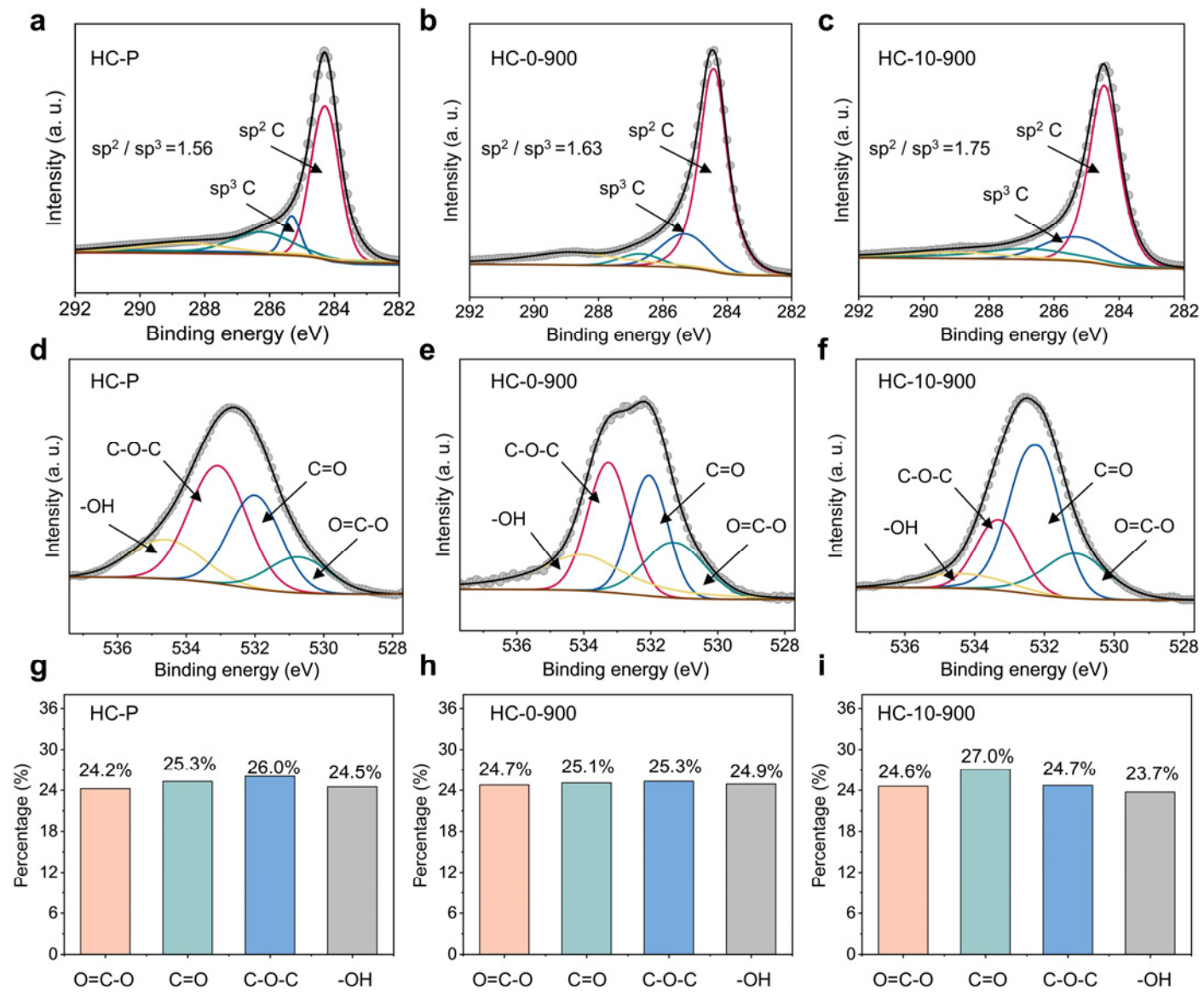

Figure R16. High-resolution (a-c) C 1s and (d-f) O 1s XPS spectra, and (g-i) the percentage of oxygen-containing functional groups of HC-P, HC-0-900, and HC-10-900.

Chemical composition changes during the pyrolysis of glucose and the chemical structure of HC. The chemical structure of HC is determined by the chemical composition of the intermediate products (HC-P) formed during pyrolysis. The HC-P obtained from the dehydration and condensation reaction of glucose following low-temperature pre-carbonization still possesses a high oxygen content of 16.9%. The surface of the HC-P is rich in carbon-oxygen bonds and oxygen-containing functional groups (**Figures R16a, d, g**). Furthermore, the sp^2/sp^3 ratio of the intermediate reaches as low as 1.56, indicating a low-crystallinity, unstable, and highly disordered carbon structure. After direct high-temperature carbonization of the HC-P, the oxygen content of HC-0-900 decreased to 10.3% due to the decomposition of carbon-oxygen bonds and oxygen-containing functional groups (**Figures R16b, e, h**). This is also accompanied by a significant gas release, causing the carbon framework to expand, which results in the HC-0-900 exhibiting high SSA, high defect, and high porosity. After thermal-force coupling carbonization of the HC-P, the oxygen content of HC-10-900 was further reduced to 7.9%, and the sp^2/sp^3 ratio increased to 1.75 (**Figures R16c, f, i**). During this process, the cross-linking of carbon-oxygen bonds and sp^2 carbon regions

and the stacking of carbon layers are enhanced as pressure promotes the densification of the material. HC-10-900 exhibits low SSA, low d_{002}, low porosity, and a locally ordered microcrystalline structure.

Our revision:

1. Added detailed description.

p 8, line 166-170 (Results)

First, glucose undergoes dehydration through pre-carbonization to yield a carbon matrix with an amorphous structure (HC-P, Supplementary Fig. 10). Under single thermal field conditions, the HC-P undergoes thermal decomposition and expansion, and the ordered cross-linking of carbon atoms requires overcoming long-range reaction pathways[38] (Supplementary Fig. 11).

Supplementary Note 3 (Supplementary Information)

Morphological and structure characterization reveal that HC-P exhibits irregular, thin carbon flakes, abundant defects, and randomly distributed, short carbon frameworks. The XRD pattern and Raman spectra further confirmed the amorphous structure of HC-P, due to significant decomposition reactions occurring during low-temperature treatment. HC-P exhibits a dense, amorphous polymer network due to dehydration-condensation during a low-temperature process, with no significant carbonization and aromatization having occurred, the material lacks a distinct pore structure. Furthermore, the sp^2/sp^3 ratio of HC-P reaches as low as 1.56 and rich oxygen-containing functional groups, indicating a low-crystallinity, unstable, and highly disordered carbon structure.

Supplementary Note 4 (Supplementary Information)

In situ TGA-FTIR analysis reveals characteristic gas-phase signatures of CO_2, CO, and H_2O during pyrolysis treatment, indicating significant decomposition reactions occur throughout the pyrolysis process. During the pre-carbonization process at 300°C of glucose, characteristic gas-phase peaks of CO_2 and H_2O were detected at 550-750 cm^{-1}, 2250 to 2500 cm^{-1}, and 2750-3750 cm^{-1}, indicating significant decomposition reactions resulting from dehydration between hydroxyl groups and the breaking of carbon-oxygen bonds. Furthermore, as the carbonization temperature increases to 900°C, the amount of CO_2 gas released continues to rise, and the decline in material quality becomes more pronounced. In contrast, when glucose undergoes pre-carbonization at 300°C followed by high-temperature carbonization at 900°C, CO_2 emissions are reduced, and carbon yield is increased.

2. Added figures.

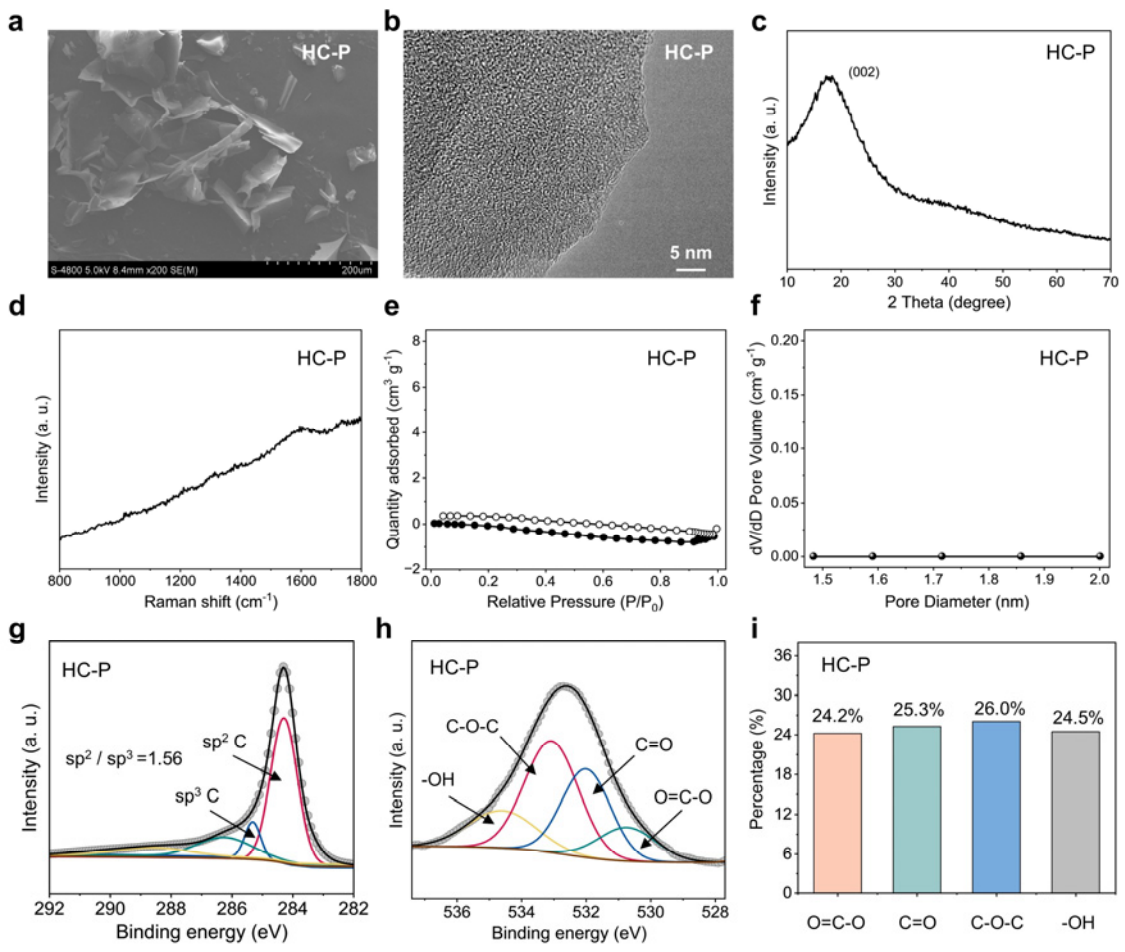

Supplementary Fig. 10. The microstructure of HC-P: (a) Scanning electron microscopy (SEM) image. (b) High-resolution transmission electron microscopy (HRTEM) image. (c) X-ray diffraction (XRD) pattern. (d) Raman spectra. (e) Nitrogen adsorption/desorption isotherms. (f) Pore size distribution. High-resolution (g) C 1s and (h) O 1s X-ray photoelectron spectroscopy (XPS) spectra. (i) The percentage of oxygen-containing functional groups.

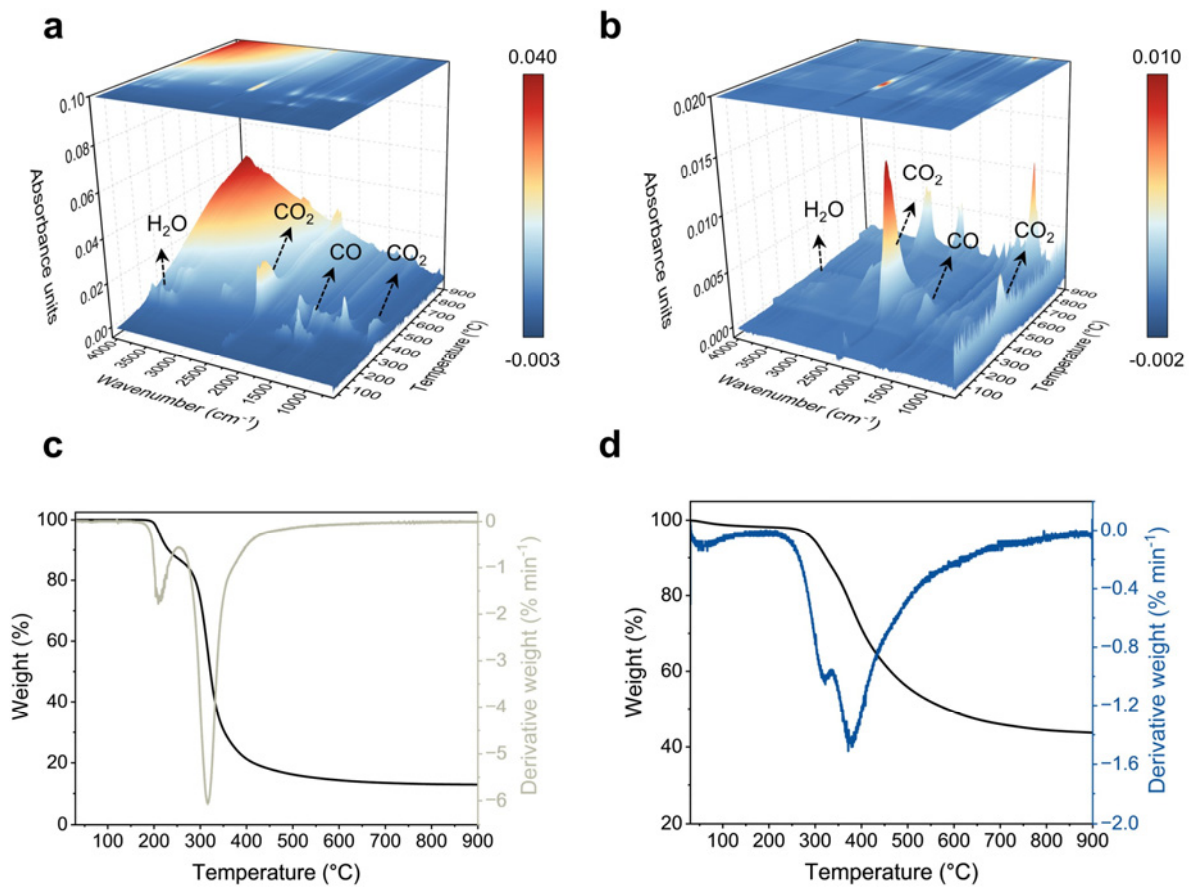

Supplementary Fig. 11. In situ thermogravimetry analysis-Fourier transform infrared (TGA-FTIR) of (a, c) the direct pyrolysis process of glucose and (b, d) the pyrolysis process of glucose after pre-carbonization.

Comment #9. Carbonization yield of ~50% seems low for commercialization purposes of these materials. Can you suggest ways in which the yields can be improved? Can you do direct carbonization without pre-aromatization at 300 degrees Celsius to see if the results of their battery performance are similar? So, we know if we can skip the intermediate step or not, or why that step is important. And also, if that can improve the yield without affecting the performance.

Response: We greatly appreciate your insightful questions regarding the carbonization yield of HC materials, which is crucial for assessing the commercial viability of HC materials.

Table R5. The yield of HC obtained from multiple batch experiments.

Sample	Yield 1	Yield 2	Yield 3	Average yield
HC-0-900	35.19%	35.84%	36.68%	35.90%
HC-0-1300	19.40%	19.70%	20.30%	19.80%
HC-10-900	43.08%	43.30%	43.83%	43.40%

Under low-temperature thermal field carbonization, high-temperature thermal field carbonization, and low-temperature thermal-force coupling carbonization, the carbonization yield of HC-0-900, HC-0-1300, and HC-10-900 are 35.90%, 19.80%, and 43.40%, respectively (**Table R5**). While achieving comparable electrochemical performance, the carbonization yield of HC-10-900 is significantly higher than that of HC-0-1300. Moreover, at the same carbonization temperature, low-temperature thermal-force coupling carbonization promotes material densification, which enhances the yield of HC. However, the carbonization yield of HC-10-900 still fails to meet the commercial demand for HC, a limitation dictated by the composition of the precursor. Glucose is composed of carbon, hydrogen, and oxygen, providing a high oxygen-to-carbon ratio. The oxygen and hydrogen vent out as gases during the carbonization process, resulting in a low carbonization yield. In this work, glucose-derived carbon was primarily employed as a representative model to elucidate the mechanisms underlying the microstructure evolution of HC during thermal-force coupling carbonization, owing to its high purity, simple composition, and well-defined structure. Furthermore, experimental and economic analyses have demonstrated that the thermal-force coupling carbonization strategy offers significant commercial advantages in terms of improving carbonization yield and reducing production costs of HC (**Figure R17**).

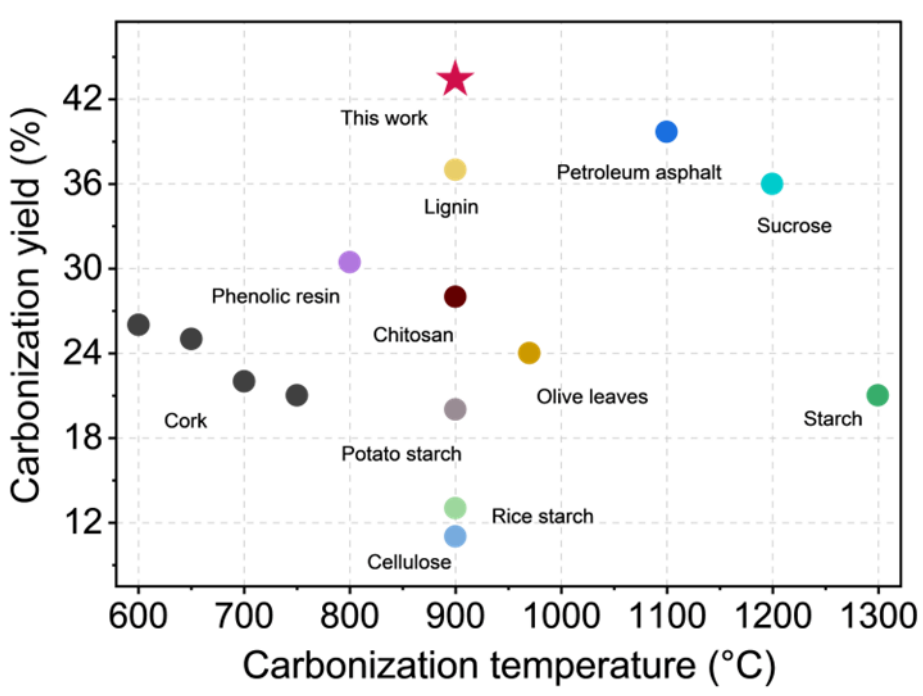

Figure R17. Comparison of carbonization yields for HC derived from different precursors.

In recent years, glucose-derived carbon has often been used as a model for HC to study its sodium storage mechanism (***Energy Environ. Sci. 2020,*** 13, 10, 3469-3479). Methods to improve its carbonization yield include: **1) Hydrothermal carbonization**. Following hydrothermal carbonization and high-temperature carbonization, the glucose-derived carbon exhibits a densified spherical morphology, which helps improve the yield. (***Adv. Energy Mater. 2022,*** 12, 18, 2200208). **2) Dehydration pre-carbonization.** Before high-temperature carbonization, the glucose undergoes a low-temperature dehydration process to remove some of the crystalline water and hydroxyl groups, thereby reducing weight loss during the subsequent high-temperature stage (***ACS Appl. Energy Mater. 2023,*** 6, 1, 181−190). **3) Chemical cross-linking.** By introducing high-carbon precursors into glucose and forming a more stable network structure through chemical cross-linking, the loss of volatile components during pyrolysis is reduced (***Chem. Eng. J. 2024,*** 490, 15, 151428). Furthermore, optimizing the carbonization temperature and controlling the heating rate to minimize the loss of carbon-containing materials also improves the final carbon yield.

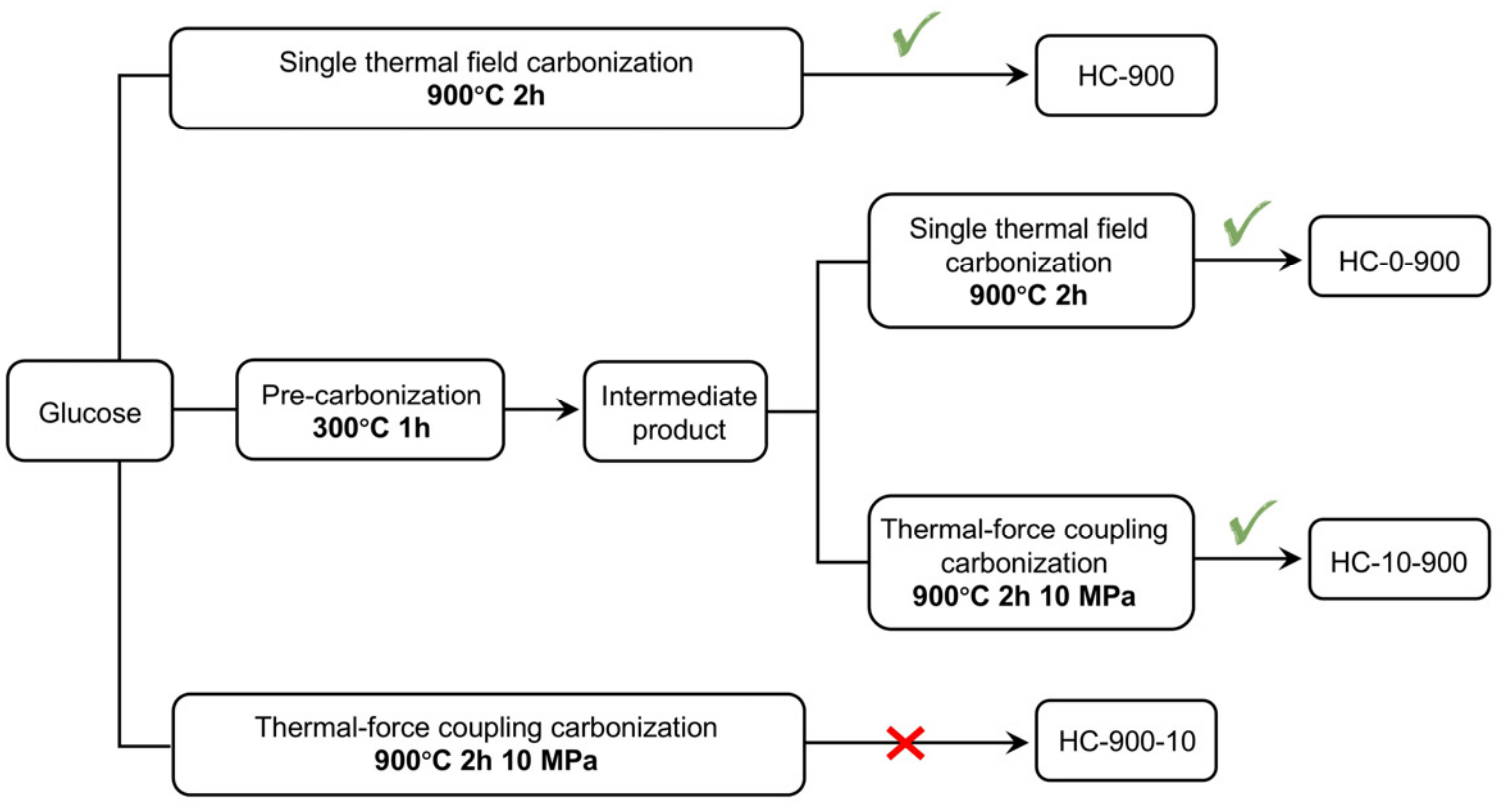

Figure R18. The carbonization process of glucose with and without pre-carbonization.

In terms of experimental design, the pre-carbonization process was adopted for the following reasons: On the one hand, the pre-carbonization process dehydrates the glucose, thereby reducing the loss of mass during subsequent high-temperature carbonization. On the other hand, the pre-carbonization process promotes the caramelization of glucose. The resulting caramelized products remain in solid particle form throughout the subsequent high-temperature carbonization process, allowing them to be compressed into dense blocks under thermal-force coupling conditions. However, when glucose is subjected to thermal-force coupling carbonization without prior carbonization treatment, it cannot be compressed into a dense block because the caramelization

reaction causes the glucose to soften (**Figure R18**).

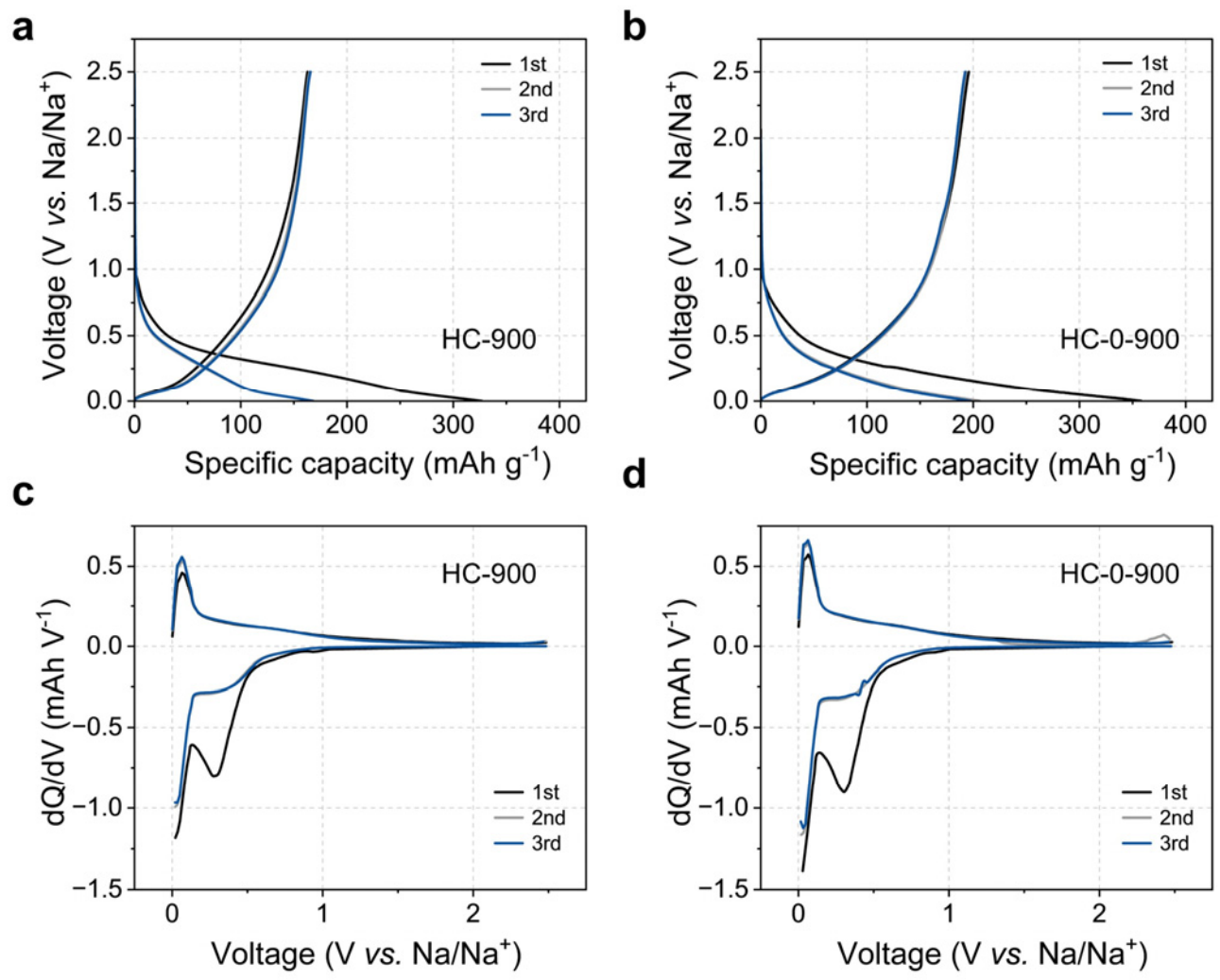

Figure R19. The Charge-discharge curves and dQ/dV curves of (a, c) HC-900 and (b, d) HC-0-900.

To further investigate the effect of the pre-carbonization process on the electrochemical performance of HC, we subjected glucose to direct carbonization at 900°C under atmospheric pressure. The resulting product was named HC-900. After undergoing pre-carbonization followed by high-temperature carbonization, the resulting material is designated HC-0-900. In electrochemical performance testing, the HC-900 provides an ICE of 49.8% and a charge capacity of 162.76 mAh g^{-1}. And the HC-0-900 provides the ICE of 54.7% and the charge capacity of 195.8 mAh g^{-1} (**Figure R19**). The ICE and charging capacity of the HC-900 are lower than those of the HC-0-900. This is attributed to the continuous occurrence of extensive decomposition reactions during the direct high-temperature carbonization of glucose, resulting in increased SSA and porosity. Both the HC-900 and HC-0-900 exhibit irreversible reduction peaks in the first discharge cycle, and their oxidation peaks are relatively weak, indicating that both have highly disordered structures. Therefore, pre-carbonization can mitigate decomposition reactions during the subsequent high-temperature carbonization process, enhancing the orderliness of the structure and thereby improving the reversible sodium storage performance of HC.

Our revision:

1. Added detailed description.

p 8, line 166-170 (Results)

First, glucose undergoes dehydration through pre-carbonization to yield a carbon matrix with an

amorphous structure (HC-P, Supplementary Fig. 10). Under single thermal field conditions, the HC-P undergoes thermal decomposition and expansion, and the ordered cross-linking of carbon atoms requires overcoming long-range reaction pathways[38] (Supplementary Fig. 11).

p 9, line 174-177 (Results)

In contrast, thermal-force coupling carbonization not only compacts the material into a dense block, significantly reducing the SSA, but also promotes the slow growth of the carbon structure, keeping the I_D/I_G and d_{002} within reasonable ranges (Supplementary Fig. 12, 13).

Supplementary Note 4 (Supplementary Information)

In situ TGA-FTIR analysis reveals characteristic gas-phase signatures of CO_2, CO, and H_2O during pyrolysis treatment, indicating significant decomposition reactions occur throughout the pyrolysis process. During the pre-carbonization process at 300°C of glucose, characteristic gas-phase peaks of CO_2 and H_2O were detected at 550-750 cm^{-1}, 2250 to 2500 cm^{-1}, and 2750-3750 cm^{-1}, indicating significant decomposition reactions resulting from dehydration between hydroxyl groups and the breaking of carbon-oxygen bonds. Furthermore, as the carbonization temperature increases to 900°C, the amount of CO_2 gas released continues to rise, and the decline in material quality becomes more pronounced. In contrast, when glucose undergoes pre-carbonization at 300°C followed by high-temperature carbonization at 900°C, CO_2 emissions are reduced, and carbon yield is increased.

2. Added figures.

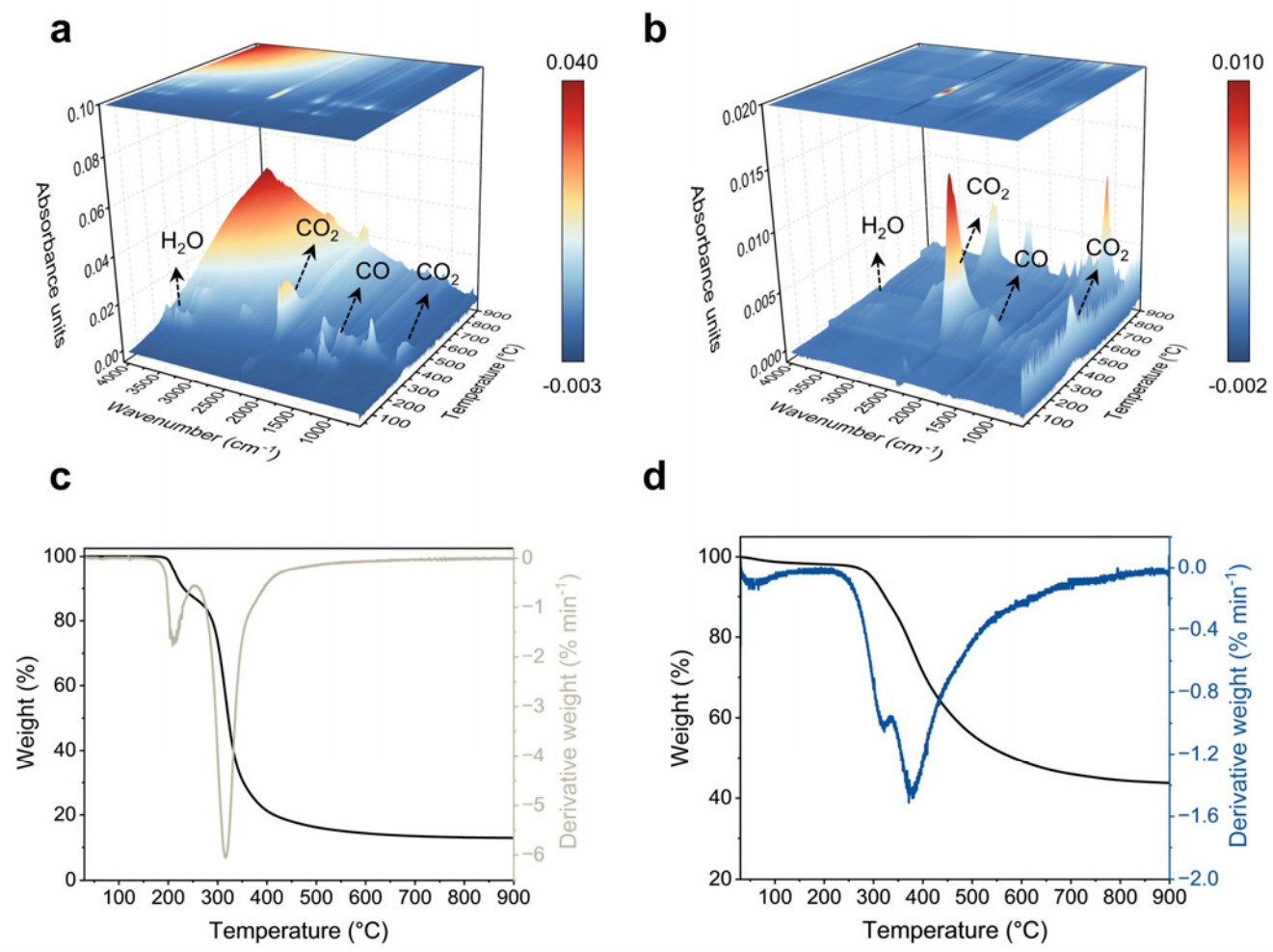

Supplementary Fig. 11. In situ thermogravimetry analysis-Fourier transform infrared (TGA-FTIR) of (a, c) the direct pyrolysis process of glucose and (b, d) the pyrolysis process of glucose after pre-carbonization.

Comment #10. The cost factor can also be improved by removing the conductive agent, as shown in some works. (https://pubs.acs.org/doi/abs/10.1021/acsaem.4c01769). Can it apply in this case? Can you try to do one analysis with the best sample without incorporating the conductive agent, and we can compare the battery performance?

Response: We thank the reviewer for this insightful question. Conductive agents typically influence the formation of the micro-conductive network within the electrode, thereby determining the overall electrochemical performance of sodium-ion batteries.

As a typical conductive agent, acetylene black is embedded between the HC particles, forming a three-dimensional conductive network that reduces the resistance to electron transport. Moreover, the electrons do not need to pass through the interior of HC, thereby shortening the path of electron transport (***Small Methods 2026,*** 10, 2500786). The addition of a conductive agent improves Na^+ diffusion kinetics, which reduces electrode polarization under high current conditions, and thereby achieves excellent rate performance and cycle stability. Without the addition of a conductive agent, it is difficult to form a conductive network within the electrode due to the irregular shape and uneven contact of the HC particles, which leads to increased resistance (***Carbon 2023,*** 213, 118191).

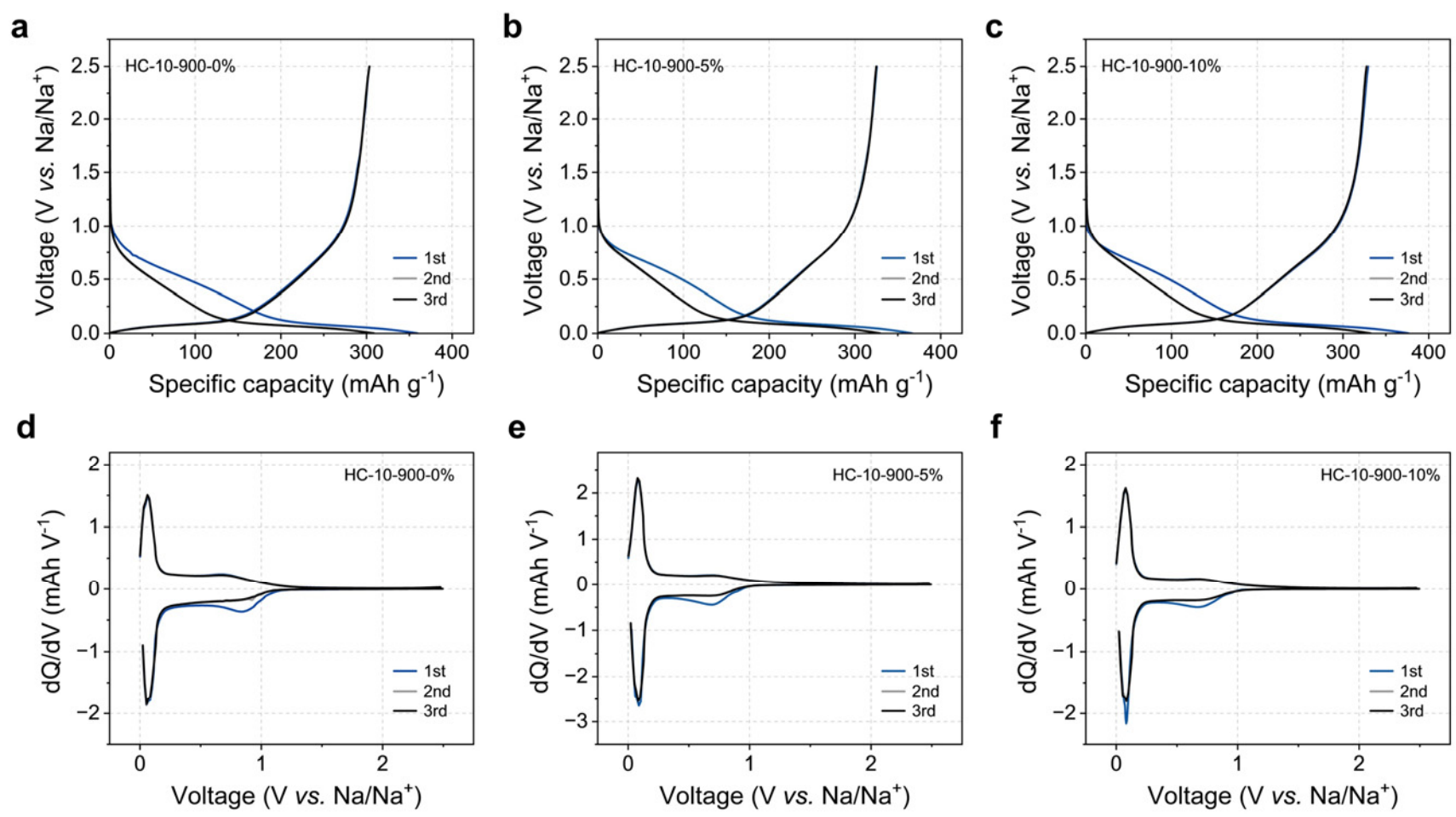

Figure R20. The Charge-discharge curves and dQ/dV curves of (a, d) HC-10-900-0% and (b, e) HC-10-900-5%, and (c, f) HC-10-900-10%.

To investigate the effect of conductive agents on the electrochemical performance of HC-10-900, we added the conductive agents at mass fractions of 10%, 5%, and 0% to the electrodes (**Figure R20**). In electrochemical performance testing, the initial discharge/charge capacities of HC-10-900-

10%, HC-10-900-5%, and HC-10-900-0% are 376.6/329.3, 366.9/325.6, and 359.7/303.2 mAh g^{-1}, respectively, with the corresponding ICE of 87.4%, 88.7%, and 84.3%. The reversible plateau capacities of HC-10-900-10%, HC-10-900-5%, and HC-10-900-0% are 159.4, 150.4, and 135.7 mAh g^{-1}, respectively. The HC-10-900-5% has a charge capacity similar to that of the HC-10-900-10%, but its ICE is slightly higher than that of the HC-10-900-10%. This indicates that both 5% and 10% mass fractions of conductive agents can ensure rapid electron transport within the electrode, thereby maximizing the high sodium storage capacity of HC. Notably, the increase in irreversible reactions in HC-10-900-10% is due to the adsorption of Na^+ by the conductive agent, which reduces the ICE. Both the reversible capacity, ICE, and reversible plateau capacity of the HC-10-900-0% decreased, which is attributed to high electronic transport resistance within the electrodes. Although HC-10-900 features short-range ordered carbon layers and abundant closed pores, which facilitate rapid Na^+ diffusion, its intrinsic conductivity is insufficient to support fast electron transport due to limited graphitization at low carbonization temperatures. Therefore, adding a small amount of conductive agent can compensate for the lack of intrinsic conductivity in HC while enabling rapid ion and electron transport.

Our revision:

1. Added detailed description and references.

p 17, line 341-342 (Results)

The above results indicate that the excellent Na^+ diffusion and efficient electron transport of HC-10-900 are attributable to the construction of diffusion pathways and the formation of conductive networks.

p 32, line 699-701 (References)

[44] Sundaramoorthy, S. et al. High-capacity anode for sodium-ion batteries using hard carbons derived from polyurea-cross-linked silica xerogel powders. *ACS Appl. Energy Mater.* **7**, 9289-9299 (2024).

Comment #11. Calculate the degree of graphitization using the obtained interlayer spacing values to see what influence it has on the reversible capacities and ICE. For reference on how the calculations can be done, you can refer to (https://www.sciencedirect.com/science/article/pii/S2667056925001221).

Response: We greatly appreciate your professional guidance, which is crucial for analyzing the degree of graphitization in HC structures. We calculated the degree of graphitization of HC material utilizing the d_{002} value based on the formula in the literature.

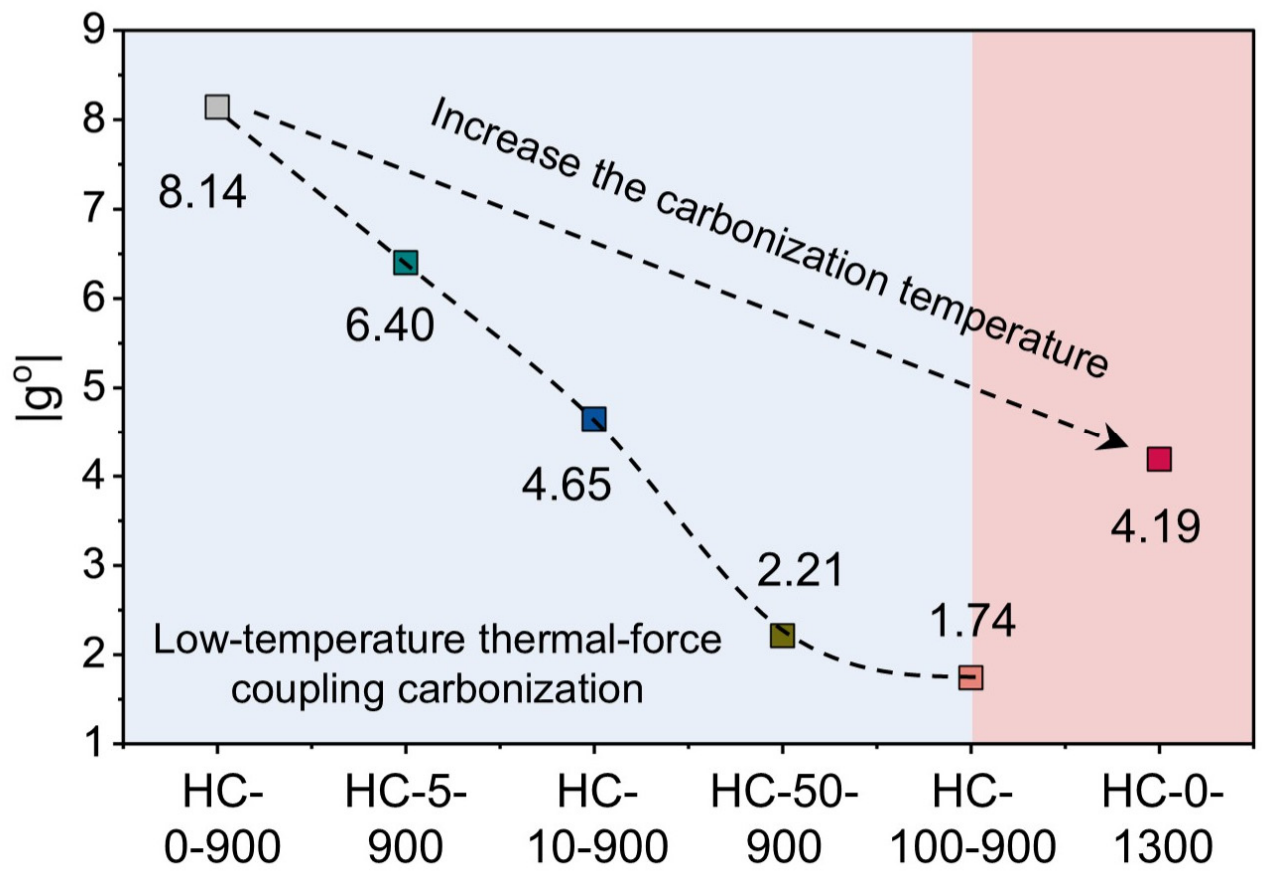

Figure R21. The |g°| of HC-0-900, HC-5-900, HC-10-900, HC-50-900, HC-100-900, and HC-0-1300.

In this work, the absolute value of g° is used to assess the degree of graphitization in HC materials. And the smaller the absolute value of g°, indicating a higher degree of graphitization. The |g°| of HC-0-900, HC-5-900, HC-10-900, HC-50-900, and HC-100-900 are 8.14, 6.40, 4.65, 2.21, and 1.74, respectively (**Figure R21**). This indicates that as pressure increases, the degree of graphitization of HC gradually increases. This is attributed to the fact that pressure-assisted thermal field carbonization promotes the ordered development of the carbon layer, and as pressure increases, the carbon layer evolves into a graphitized structure. Furthermore, the variation trend in the |g°| aligns with that of d_{002}, I_D/I_G, L_a, and L_c, collectively demonstrating that pressure can induce an ordered structure in HC at low carbonization temperatures. However, the degree of graphitization must be controlled within a reasonable range to simultaneously optimize both the Na^+ diffusion rate and reversible storage, thereby achieving high reversible capacity and ICE of HC. The HC-0-900 with a high |g°| of 8.14 exhibits a low degree of graphitization due to its highly disordered microstructure. This makes it difficult to provide sufficient reversible sodium storage sites and an efficient Na^+ diffusion rate, resulting in low reversible capacity and low ICE. In addition, the HC-100-900 with a high |g°| of 1.74 exhibits a high degree of graphitization, which is attributed to its long-range order and narrow carbon layer structure. This hinders the reversible storage of Na^+ due to the high

diffusion resistance of Na^+, leading to low reversible capacity and low ICE. In contrast, HC-10-900 with a high $|g^o|$ of 4.65 exhibits a moderate degree of graphitization, which is facilitated by its short-range ordered carbon layers and abundant closed-pore structure. This provides both a large number of reversible sodium storage sites and fast diffusion kinetics, thereby enabling high reversible capacity and high ICE.

Our revision:

1. Added detailed description and references.

p 11, line 211-213 (Results)

Besides, the R factor, L_a, and L_c increase, and $|g^o|$ values decrease of HC with increasing pressure, indicating that the compression and orderliness of the carbon layer become more pronounced[43,44] (Supplementary Fig. 21, 22).

p 32, line 696-698 (References)

[43] Owusu, S. Y., Soni, R. U. & Leventis, C. S. Catalytic synthesis of iron-doped graphitic aerogels from poly(phloroglucinol-terephthalaldehyde–urethane) precursors. *Carbon Trends* **21**, 100573 (2025).

Supplementary Note 9 (Supplementary Information)

The accurate position of the 002 peak of HC was measured by XRD tests. The d_{002} of HC was calculated by the Bragg equation. Thus, the graphitization degree could be calculated using formula[15].

$$g^o = (3.440 - d_{002})/(3.440 - 3.354)$$

Where g is the graphitization degree. The 3.440 is the interplanar spacing in carbon with a turbostratic structure. The d_{002} is the value of interlayer spacing of graphite obtained by XRD characterization, and the 3.354 is the interplanar spacing in an ideal defect-free crystal of graphite. (units are in Å)

In this work, the absolute value of g^o is used to assess the degree of graphitization in HC materials. And the smaller the absolute value of g^o, indicating a higher degree of graphitization. The $|g^o|$ of HC-0-900, HC-5-900, HC-10-900, HC-50-900, and HC-100-900 are 8.14, 6.40, 4.65, 2.21, and 1.74, respectively. This further indicates that as pressure increases, the degree of graphitization of HC gradually increases.

2. Added figures and tables.

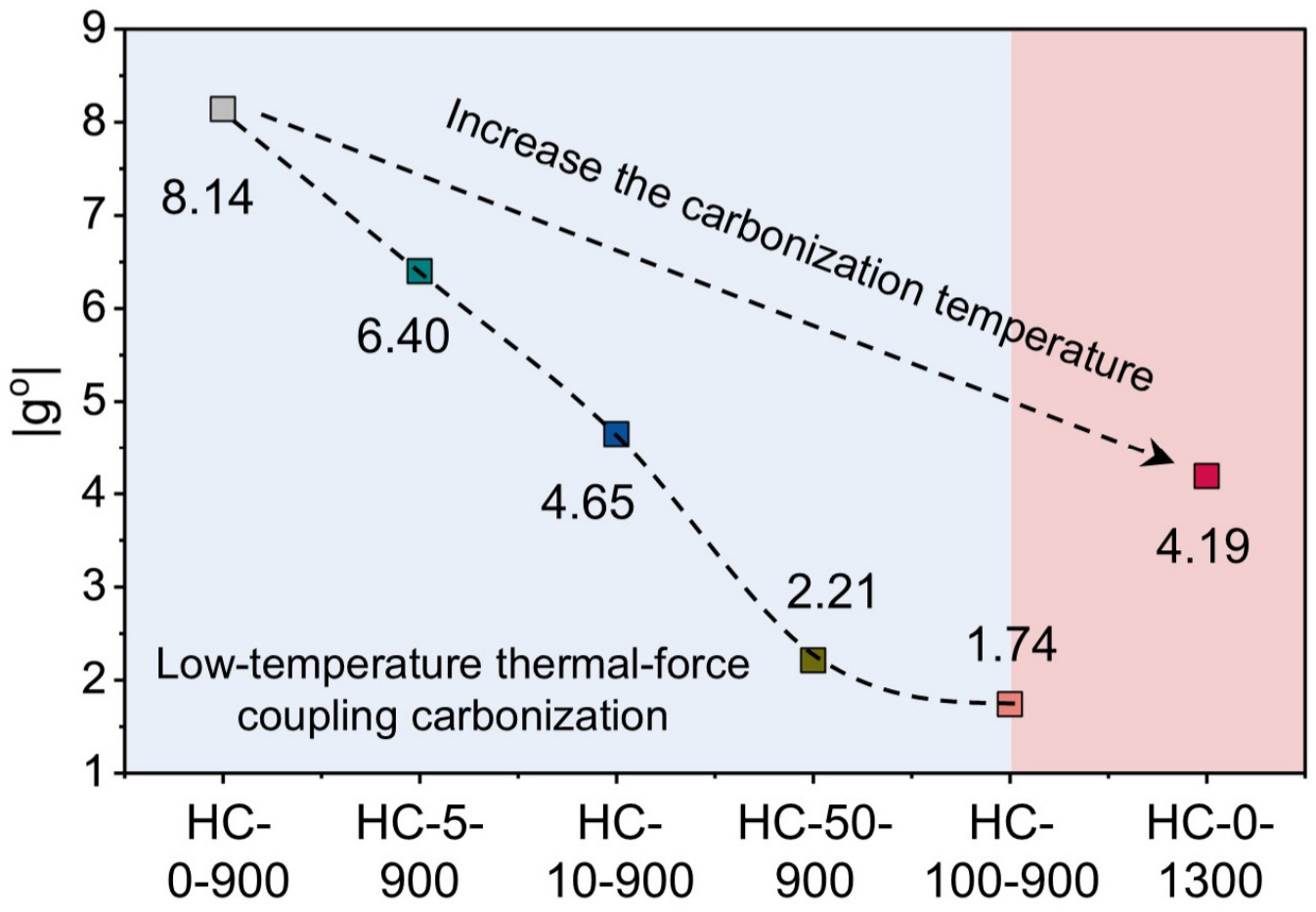

Supplementary Fig. 22. The |g°| of HC-0-900, HC-5-900, HC-10-900, HC-50-900, HC-100-900, and HC-0-1300.

Supplementary Table 3. Carbon layer structure of HC.

Sample	(002)	d_{002} (nm)	L_a (nm)	L_c (nm)	R-factor	|g°|	I_D/I_G
HC-0-900	21.44	0.414	8.01	1.787	1.787	8.14	1.72
HC-5-900	22.26	0.399	8.26	1.854	2.274	6.40	1.50
HC-10-900	23.14	0.384	8.53	1.990	2.372	4.65	1.33
HC-50-900	24.49	0.363	8.99	2.117	2.664	2.21	1.21
HC-100-900	24.77	0.359	10.29	2.312	3.436	1.74	1.01
HC-0-1300	23.40	0.380	9.79	1.895	3.205	4.19	1.05

Comment #12. Was the Raman data determined using the peak maxima values or the integrated peak intensities? The peak intensities give more accurate results since they are determined using the area under the curve rather than the peak height, and those values tend to be smaller. Can you make changes to reflect this if needed? The peak intensity fittings can be achieved in Origin.

Response: We are deeply grateful to the reviewers for this insightful inquiry into the Raman data analysis. In this work, the multi-peak fitting of the Raman spectrum is performed by the Gaussian fitting function of Origin software. Based on the Lorentz function, Raman spectra of carbon materials were further fitted with D_1 (1345 cm^{-1}), D_2 (1200 cm^{-1}), D_3 (1500 cm^{-1}), and G band (1590 cm^{-1}). The D_1 and G bands correspond to defect structure and graphitic lattice within graphene layers, respectively. The D_2 and D_3 bands correspond to changes in defects and oxygen-containing groups growing along the carbon edge or carbon layer, respectively.

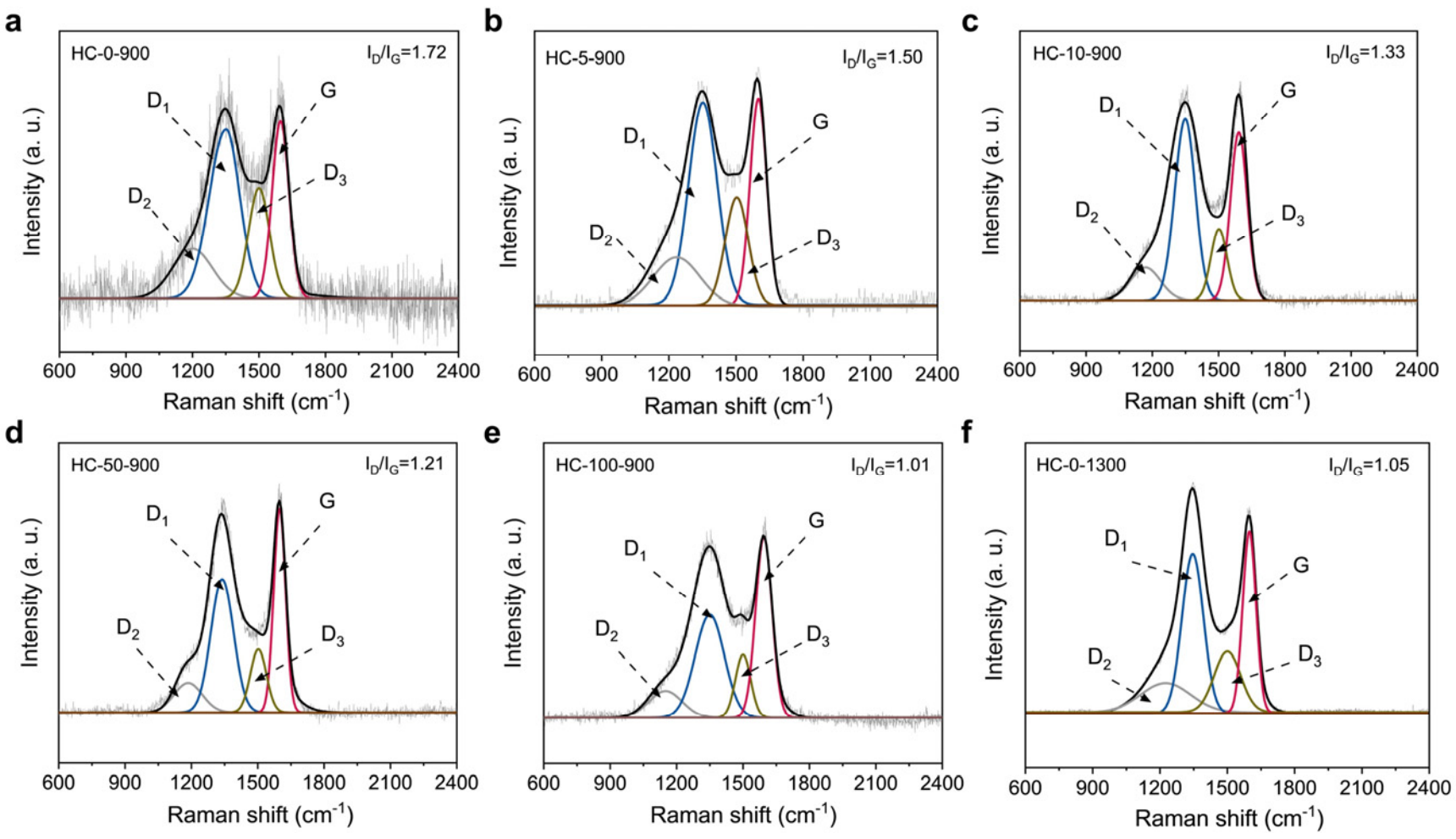

Figure R22. Raman spectra of (a) HC-0-900, (b) HC-5-900, (c) HC-10-900, (d) HC-50-900, (e) HC-100-900, and (f) HC-0-1300.

To improve the accuracy of the I_D/I_G in assessing the degree of graphitization of HC, we recalculated the I_D/I_G using the integrated areas of the D and G peaks. The I_D/I_G of HC-0-900, HC-5-900, HC-10-900, HC-50-900, HC-100-900, and HC-0-1300 were calculated as 1.72, 1.50, 1.33, 1.21, 1.01, and 1.05, respectively (**Figure R22**). The high I_D/I_G of HC-0-900 indicates a rich defect structure. This is attributed to the weak ordered cross-linking reaction and significant decomposition reaction during low-temperature carbonization. As pressure increases, the I_D/I_G of the HC decreases sequentially, indicating that surface-exposed defects are reduced due to the densification of the material. Among these, ordered carbon layers and appropriate defects of HC-10-900 form a Na^+ diffusion network, which improves the diffusion kinetics of Na^+. Compared to force fields, further increasing the carbonization temperature also promotes graphitization development of HC, yet this process is difficult to control. HC-0-1300 exhibits a low I_D/I_G, and its low-defect and highly ordered structure increases the resistance to Na^+ diffusion.

Our revision:

1. Added detailed description.

p 11, line 213-214 (Results)

The I_D/I_G of HC-0-900, HC-10-900, and HC-0-1300 were calculated as 1.72, 1.33, and 1.05, respectively (Supplementary Fig. 23).

Supplementary Note 10 (Supplementary Information)

The I_D/I_G of HC-0-900, HC-5-900, HC-10-900, HC-50-900, HC-100-900, and HC-0-1300 are

1.72, 1.50, 1.33, 1.21, 1.01, and 1.05 by using the integrated areas of the D and G peaks, respectively.

2. Added figures and tables.

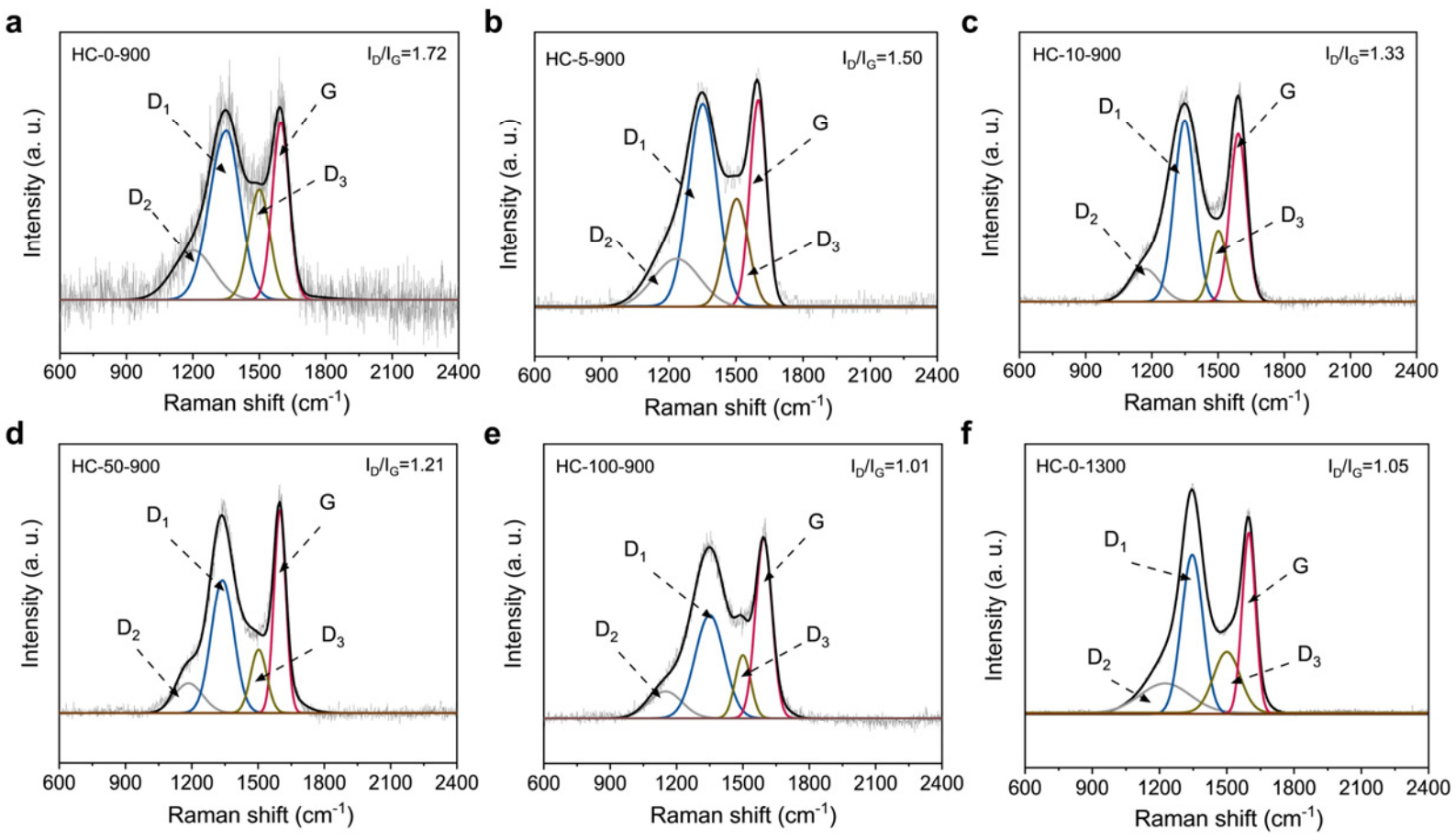

Supplementary Fig. 23. Raman spectra of (a) HC-0-900, (b) HC-5-900, (c) HC-10-900, (d) HC-50-900, (e) HC-100-900, and (f) HC-0-1300.

Supplementary Table 3. Carbon layer structure of HC.

Sample	(002)	d_{002} (nm)	L_a (nm)	L_c (nm)	R-factor	$\|g^o\|$	I_D/I_G
HC-0-900	21.44	0.414	8.01	1.787	1.787	8.14	1.72
HC-5-900	22.26	0.399	8.26	1.854	2.274	6.40	1.50
HC-10-900	23.14	0.384	8.53	1.990	2.372	4.65	1.33
HC-50-900	24.49	0.363	8.99	2.117	2.664	2.21	1.21
HC-100-900	24.77	0.359	10.29	2.312	3.436	1.74	1.01
HC-0-1300	23.40	0.380	9.79	1.895	3.205	4.19	1.05

We sincerely thank you for your constructive comments and careful evaluation of our work. We hope that the substantial revisions and new analyses provided in this response satisfactorily address all of your concerns.

Point-by-Point Response

(Black type: Reviewer's comments; Blue type: Our response; Red type: Our revision)

Reviewer #4 (Remarks to the Author):

This manuscript reports a pressure-assisted carbonization strategy to prepare hard carbon anodes at 900 °C, which is claimed to simultaneously enable abundant sodium storage sites and fast Na^+ diffusion. The authors combine a literature-derived machine-learning analysis with experimental synthesis and demonstrate promising electrochemical performance, including an Ah-level pouch cell with a reported energy density of 216.1 Wh kg^{-1}. However, the claims relating to machine learning-guided design, "thermal-force coupling" preparation, and sustainability appear to be overstated, and there are major issues to be addressed.

Response: We sincerely appreciate your recognition of our work, as well as the decision and constructive comments you have provided on our manuscript.

This study first employs machine learning to objectively identify the interrelationships among the preparation, structure, and performance of hard carbon (HC) from a large dataset, thereby identifying high-precision and promising ranges of structure parameters to serve as a design guideline and basis for high-performance HC. Since the current preparation of HC relies heavily on high-temperature carbonization, the thermal-force coupling carbonization is proposed to achieve both reversible sodium storage sites and diffusion kinetics in HC at low carbonization temperatures based on the structure findings of machine learning (ML). HC with a targeted structure exhibits excellent electrochemical performance in button cells and pouch cells, further validating the accuracy and predictive capability of the ML results. Based on an analysis of economic benefits and environmental impacts, low-temperature thermal-force carbonization demonstrates both sustainability and potential in practical applications.

To enhance the persuasiveness of our research content, we have supplemented the manuscript and Supplementary Information focused on ML, thermal-force coupling preparation, and economic analysis. **Machine learning:** We updated the database by adding process parameters (carbonization time, heating rate), structure parameters (pore volume (V_{total}), medium basal coherence length (L_a), and crystal size in the stacking direction (L_c)), and performance parameters (reversible capacity) to gain a more comprehensive understanding of their interrelationships. The selection of ML models

no longer relies solely on R^2 values, but instead takes into account a combination of predictive performance, cross-validation stability, generalization gap, and interpretability to improve the accuracy of conclusions. **Thermal-force coupling preparation:** We have focused on highlighting research advances in thermal-force coupling carbonization, as well as the mechanisms underlying microstructure evolution and the associated advantages with a variety of characterizations, tests, and molecular dynamics simulations. **Economic analysis:** The analysis highlights the economic benefits and environmental impact of small-scale laboratory-scale production.

We believe that these additions have enhanced the overall scientific rigor of the manuscript. Thank you once again for your valuable suggestions. Below, we discuss the specific suggestions you raised and provide point-by-point explanations.

Comment #1. The practical scientific value of this ML component is unclear, and it provides very limited predictive insight and appears largely disconnected from the experimental strategy.

Response: We sincerely appreciate the reviewers for their insightful comments regarding the limitations of the ML components in this study in terms of defining scientific value and predicting the depth of insights. We acknowledge that current ML models have certain limitations in providing independent, high-precision predictive insights due to the complexity of the microstructure of HC and the relative scarcity of experimental data. In this work, ML is primarily used to rapidly identify high-performance regions from a vast array of potential process and structure parameters. Bayesian optimization is employed to enhance the accuracy of interpretability analysis for models, thereby improving the efficiency of experimental design and validating known physical trends in HC.

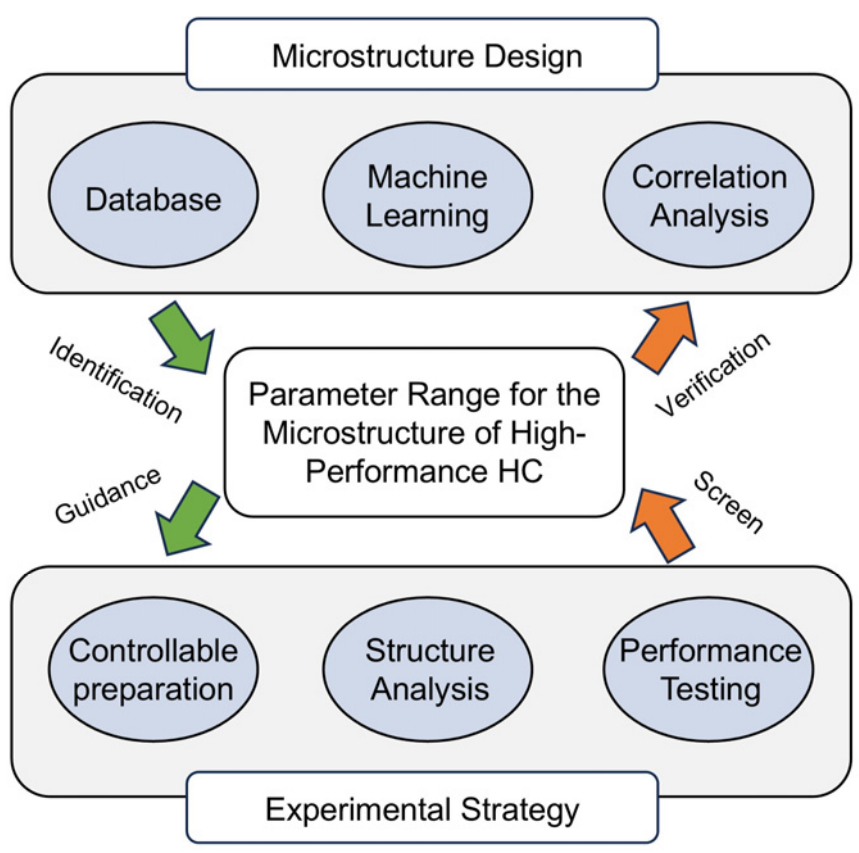

Figure R1. A closed-loop relationship between ML and experimental strategies.

Based on the range of structure parameters with high potential derived from ML, HC with the target structure was synthesized and identified through process optimization and structure characterization, combined with electrochemical performance testing to further validate the feasibility of the ML findings. Due to the highly complex contributions of diverse HC structures to electrochemical performance, repeated structure characterization and electrochemical performance testing are essential for validating the conclusions drawn from machine learning. Therefore, there is a closed-loop relationship between ML and experimental strategies in terms of guidance and validation (**Figure R1**).

Particularly in the selection of carbonization temperatures, the key microstructure parameters obtained through ML correspond to higher carbonization temperatures (1200-1800°C). High carbonization temperatures offer significant advantages in reducing SSA. Given the need to reduce both the carbonization temperature and the SSA, we employed a thermal-force coupling strategy to prepare HC with the targeted microstructure. On the one hand, the application of a force field can compress the material into a dense block, significantly reducing the SSA at low carbonization temperatures. On the other hand, force-field-assisted thermal field carbonization can promote the ordered development of the carbon structure at low carbonization temperatures. Consequently, we have not only reduced the SSA of the HC at low-carbonization temperatures (900°C) through pressure regulation, but also kept the I_D/I_G and d_{002} within reasonable ranges.

(1) First, the dataset is assembled from heterogeneous literature sources with substantial inconsistencies and hidden variables. These factors introduce strong uncertainty that limits the reliability of ML correlations. The reported R^2 value is only ~0.75, which is insufficient to support reliable conclusions.

Response: We greatly appreciate the reviewers for highlighting the critical issues regarding the heterogeneity of the dataset and the impact of latent variables on the reliability of the model. We fully agree that datasets assembled from heterogeneous sources introduce uncertainty. Data on process parameters, structure parameters, and performance parameters that are widely reported in the literature and come from consistent sources are collected into the database. For example, d_{002} was calculated using the Bragg equation, I_D/I_G was obtained from peak intensities in Raman

spectroscopy, and SSA was determined from nitrogen adsorption and desorption tests, ensuring data consistency to the greatest extent possible. Moreover, the database compiled from 565 samples collected from 245 literature sources is currently the most comprehensive database reported in the field of HC. A large database minimizes the impact of randomness and uncertainty in individual data points as much as possible.

To improve the accuracy and reliability of ML results, we have added a systematic comparison of various models, including Linear, Ridge, Lasso, GBR, XGB, Neural Network, and Random Forest (RF). When selecting the final model, we evaluate it based on R^2, predictive performance, cross-validation (CV) stability, the generalization gap, and interpretability. In calculations of ICE, the 5-fold CV mean R^2 of RF, XGB, GBR, Neural Network, Linear, Ridge, and Lasso are 0.763, 0.759, 0.734, 0.718, 0.604, 0.608, and 0.608, respectively (**Figure R2a**). In contrast, the RF model exhibits the highest R^2 (0.826), indicating its strong regression performance (**Figure R2b**). Besides, the RF model achieves an R^2 on the training set that is comparable to that of the XGB (0.825) and Neural Network (0.830) models and higher than that of other models. In calculations of reversible plateau capacity, the 5-fold CV mean R^2 of RF, XGB, GBR, Neural Network, Linear, Ridge, and Lasso are 0.805, 0.811, 0.777, 0.749, 0.701, 0.704, and 0.702, respectively. Although the CV mean R^2 for RF (0.805) is slightly lower than that for XGB (0.811), the hold-out test R^2 for RF (0.794) is higher than that for XGB (0.780), resulting in a smaller generalization gap. Therefore, after comprehensively evaluating the R^2 value, predictive performance, CV stability, generalization gap, and interpretability, the RF model was selected as the final model.

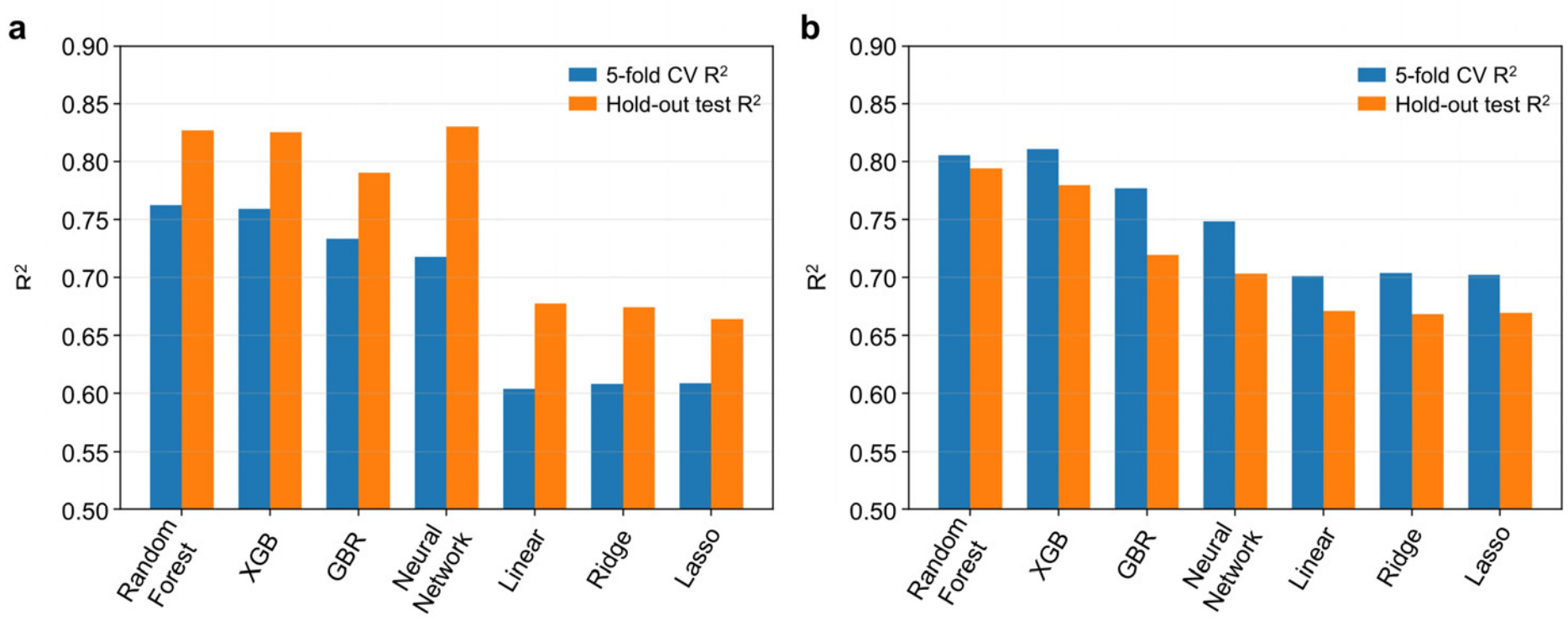

Figure R2. The performance comparison of multiple models in (a) ICE and (b) reversible plateau capacity.

We further conducted a cross-model Shapley Additive exPlanations (SHAP) ranking comparison of GBR, XGB, Ridge, and locked RF to analyze the contribution of each feature to the ICE and reversible plateau capacity, respectively. For ICE, the contributions of carbonization temperature and SSA consistently rank among the top factors across different models, while current density also remains at a consistently high level of importance (**Figure R3a**). For reversible plateau capacity, carbonization temperature, and SSA play a consistently dominant role across different models. Moreover, d_{002} and I_D/I_G both contribute moderately in different models for ICE and reversible plateau capacity (**Figure R3b**). Notably, the ranking of features by their contribution to ICE and reversible plateau capacity remains consistent in the RF model. Consequently, the conclusions drawn from ML demonstrate good robustness across multiple models, and the RF model stands out for its accuracy and reliability.

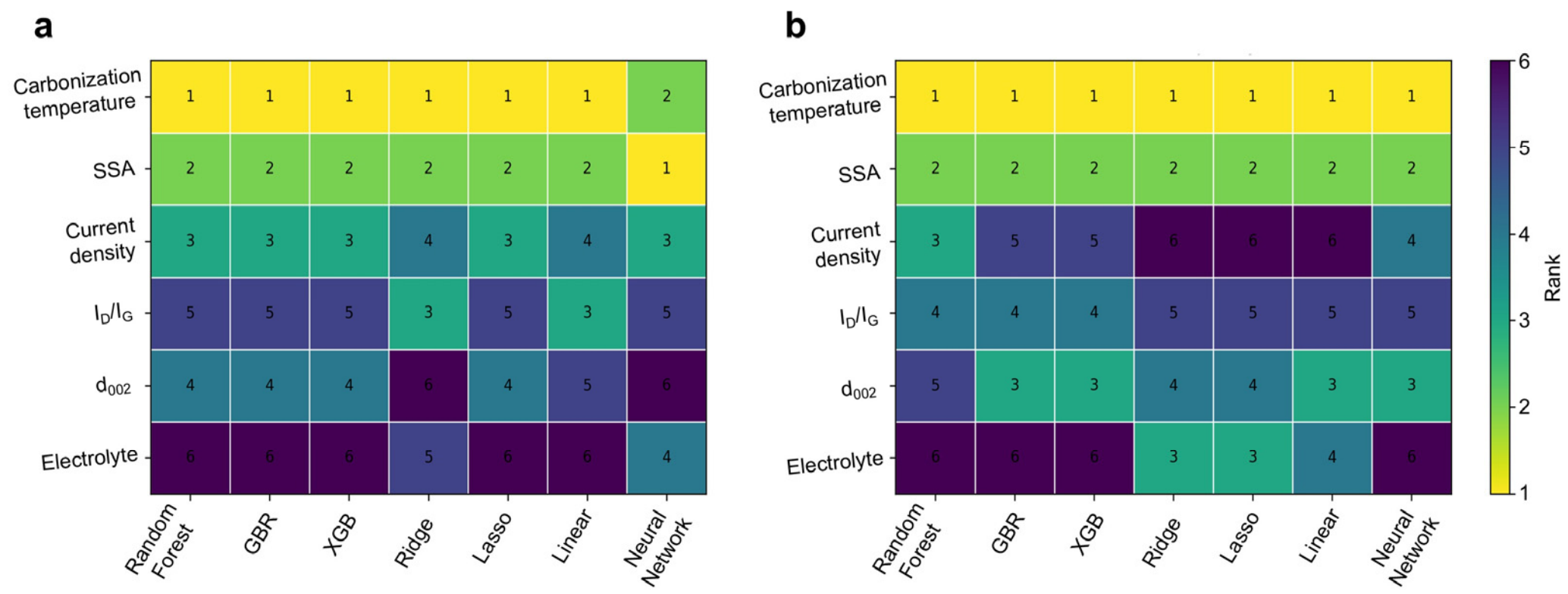

Figure R3. The heatmap of Shapley Additive exPlanations (SHAP) ranking consistency across different models in (a) ICE and (b) reversible plateau capacity.

Our revision:

1. Added detailed description.

p 6, line 124-126 (Results)

During the model evaluation phase, 5-fold cross-validation was used as the primary framework, supplemented by 10-fold cross-validation to confirm that the results were insensitive to the number of validation folds and demonstrated robustness[30].

p 7, line 132-136 (Results)

In calculations of ICE, the Random Forest (RF) model exhibits the highest 5-fold cross-validation mean R^2 (0.826), indicating strong regression performance (Supplementary Fig. 2). In calculations of reversible plateau capacity, the 5-fold cross-validation mean R^2 for RF (0.805) is slightly lower

than that for eXtreme Gradient Boosting (XGB, 0.811), the hold-out test R^2 for RF (0.794) is higher than that for XGB (0.780), resulting in a smaller generalization gap (Table S1).

p 7, line 136-138 (Results)

Besides, the ranking of features by their contribution to ICE and reversible plateau capacity remains consistent in the RF model by a cross-model Shapley Additive exPlanations (SHAP) ranking comparison (Supplementary Fig. 3).

p 7, line 138-140 (Results)

Therefore, comprehensively evaluating the R^2 value, predictive performance, cross-validation stability, generalization gap, and interpretability, the RF model was selected as the final model.

2. Added figures and tables.

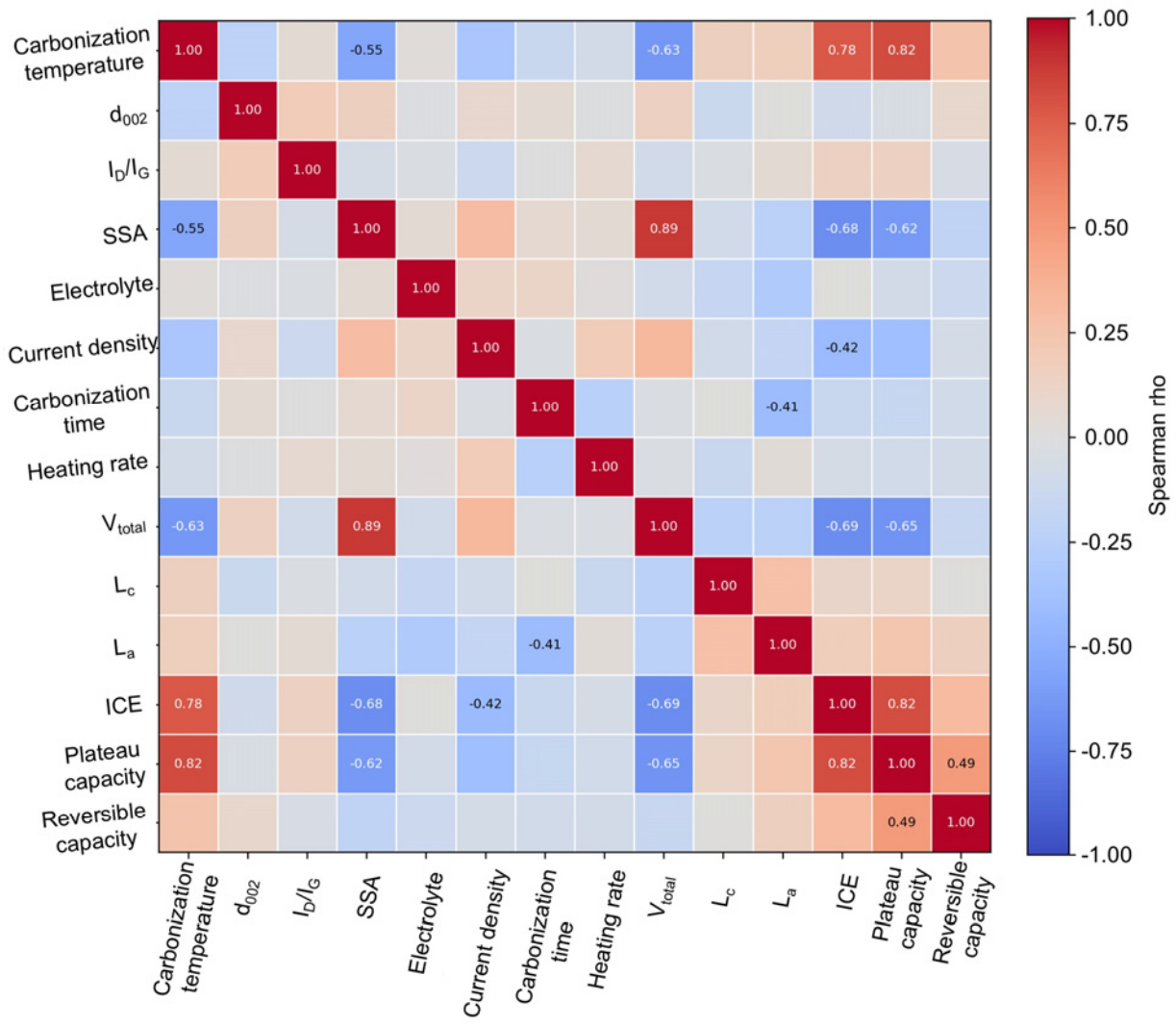

Supplementary Fig. 1. Spearman correlation heatmap between core features, missing variables, and the target variable.

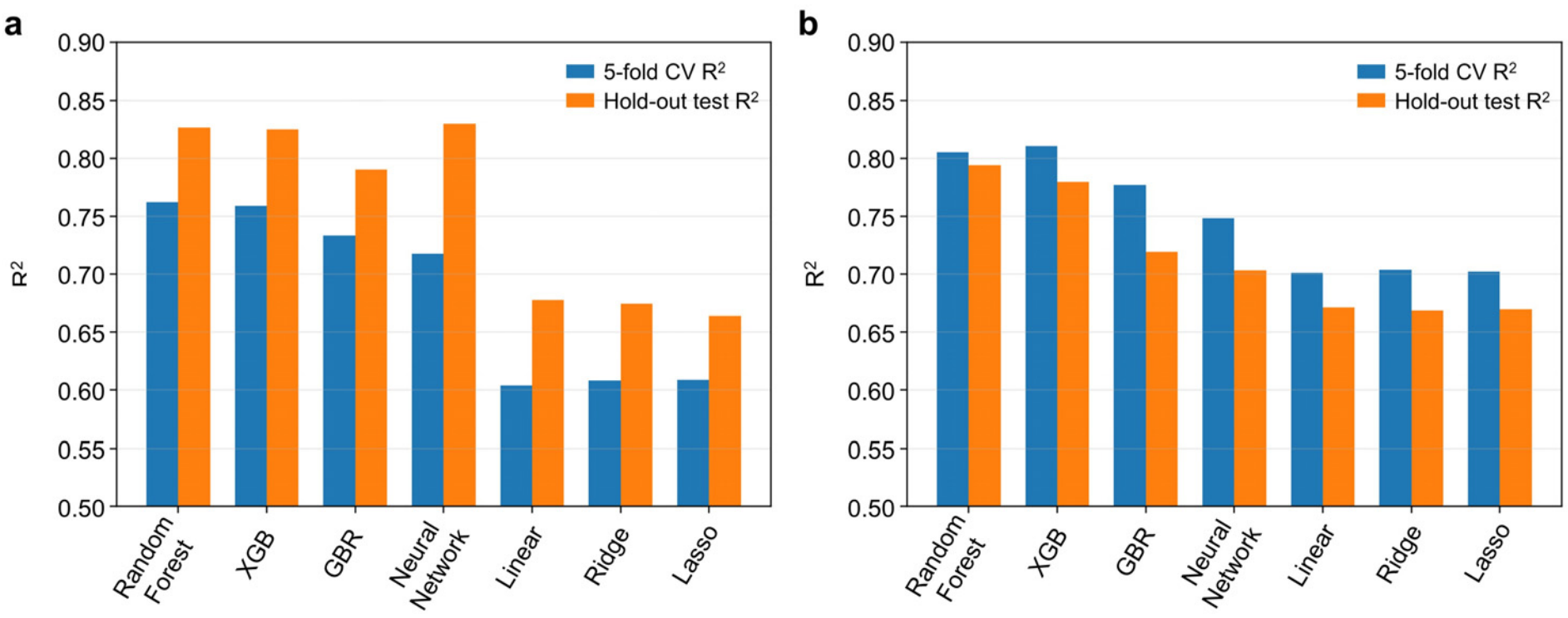

Supplementary Fig. 2. 5-fold cross-validation (CV) R^2 score of different regression models in (a) initial Coulombic efficiency (ICE) and (b) reversible plateau capacity.

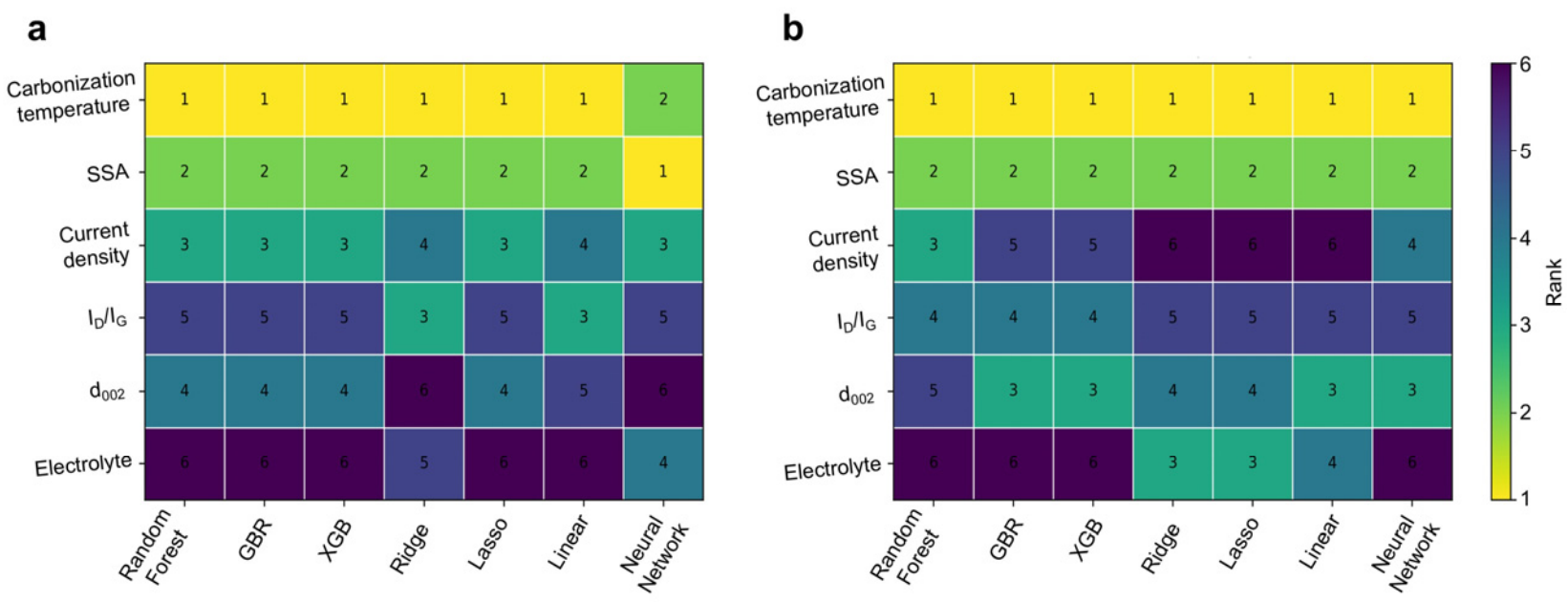

Supplementary Fig. 3. The heatmap of Shapley Additive exPlanations (SHAP) ranking consistency across different models in (a) ICE and (b) reversible plateau capacity.

Supplementary Table 1. The performance, generalization gaps, and interpretability comparison of multi-models.

Task	Model	5-fold CV mean R^2	5-fold CV std	Test R^2	Generalization gap	SHAP analysis
ICE	Random Forest	0.763	0.019	0.826	-0.062	Yes
	XGB	0.759	0.024	0.825	-0.062	Yes
	GBR	0.734	0.037	0.790	-0.052	Yes
	Neural Network	0.718	0.055	0.830	-0.106	No
	Linear	0.604	0.131	0.678	-0.062	Yes
	Ridge	0.608	0.136	0.674	-0.053	Yes
	Lasso	0.608	0.147	0.664	-0.041	Yes
Plateau capacity	Random Forest	0.805	0.041	0.794	0.012	Yes
	XGB	0.811	0.041	0.780	0.031	Yes
	GBR	0.777	0.041	0.719	0.060	Yes
	Neural Network	0.749	0.042	0.703	0.045	No
	Linear	0.701	0.047	0.671	0.030	Yes
	Ridge	0.704	0.047	0.668	0.036	Yes
	Lasso	0.702	0.047	0.669	0.033	Yes

(2) Second, the ML results do not appear to generate new insights beyond understanding already known in the hard-carbon field. It does not provide predictive capability, but a region of structure parameters. How do the ML-derived structural design principles translate into processing parameters? The current ML and experimental sections function as two largely independent narratives rather than a truly integrated design framework.

Response: We deeply appreciate the insightful questions of reviewers regarding the relationship between the ML and experimental sections. We fully agree that the conclusions drawn from ML do not go beyond the current understanding of the field of HC, as this is determined by the correlation between the structure and properties of HC. In this work, the correlation analysis of HC derived from ML is similar to the established views in the field of HC, which further supports the reliability of the ML findings. Unlike existing analyses in the HC field, ML identifies high-potential ranges of structure parameters from massive amounts of data.

Conventional wisdom generally holds that low SSA, moderate d_{002}, and moderate I_D/I_G are beneficial for improving the ICE and reversible plateau capacity of HC, yet there is no precise definition of the critical threshold. Training a nonlinear regression model using ML captures the complex nonlinear relationship between structure and performance. For example, when the SSA falls below a certain threshold, the increase in ICE tends to plateau, and further reductions in SSA may result in irreversible loss of micropore volume. In the synthesis of HC, structure parameters (SSA, d_{002}, and I_D/I_G) are often highly correlated. Traditional univariate analysis struggles to disentangle these intertwined effects, leading to misinterpretations of the role of single factors. The ML model utilizes multiple regression to statistically control for other variables, thereby independently assessing the contribution of structure and performance. The SHAP analysis further reveals the synergistic mechanisms among the parameters. When d_{002} is in a wider range, the negative impact of SSA on ICE is partially offset. Conversely, in a narrower d_{002}, even a slight increase in SSA leads to a rapid decrease in ICE.

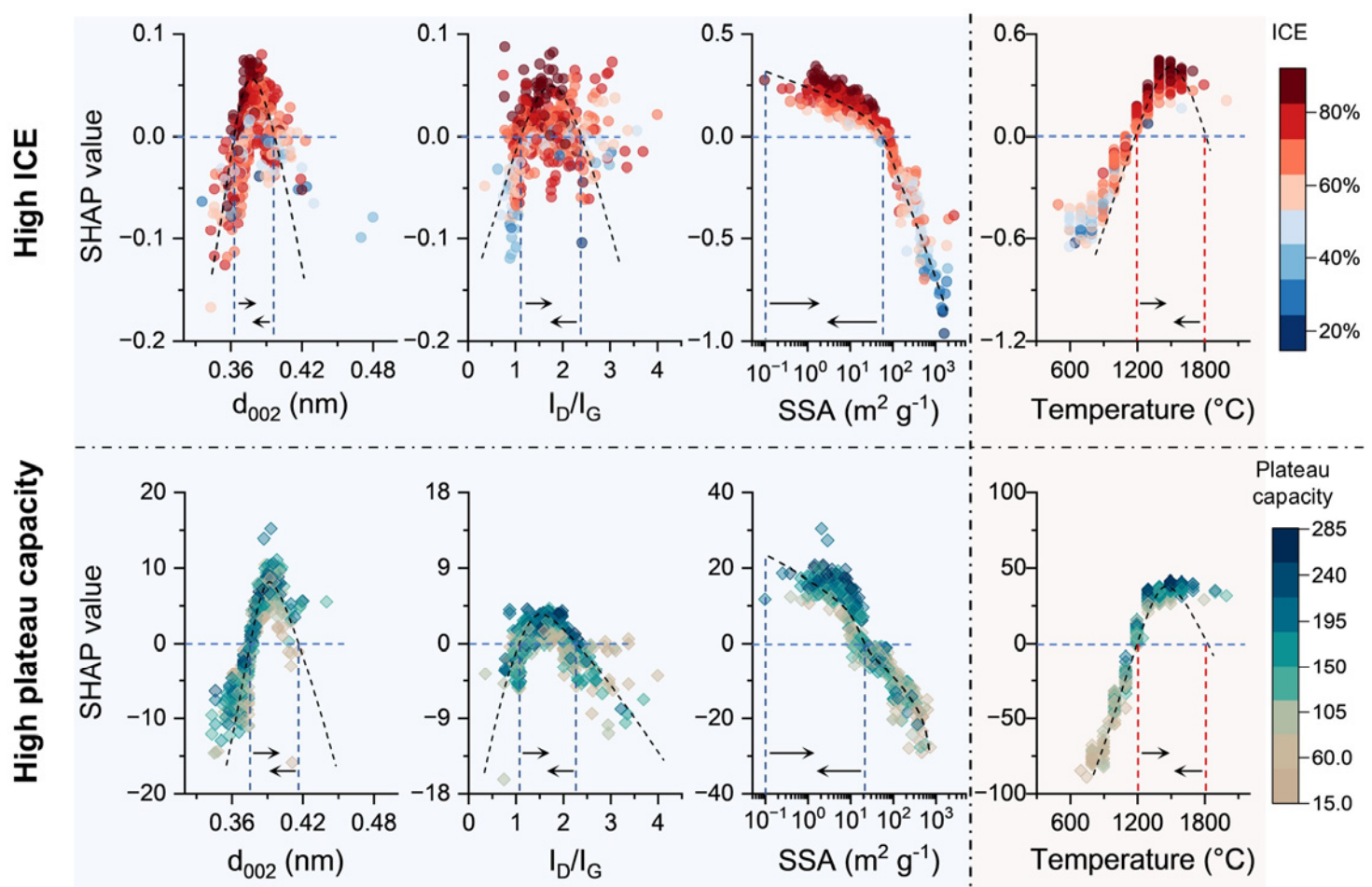

Figure R4. Relationship of ICE and reversible plateau capacity with each structure feature.

By leveraging the reliability algorithms of ML models, a suitable balance between performance metrics is identified to obtain a range of structure parameters with high-potential (**Figure R4**). In this work, HC with high ICE generally possesses a d_{002} of 0.360-0.394 nm, an I_D/I_G of 1.10-2.36, and a SSA of 0.1-77.0 m^2 g^{-1}. And HC with high reversible plateau capacity typically possesses a d_{002} of 0.376-0.420 nm, an I_D/I_G of 1.05-2.30, and a SSA of 0.26-17.84 m^2 g^{-1}. Integrating these two structure parameter ranges yields a well-defined microstructure "target zone" characterized by low SSA (0.26-17.84 m^2 g^{-1}), moderate d_{002} (0.376-0.394 nm), and moderate I_D/I_G (1.10-2.30).

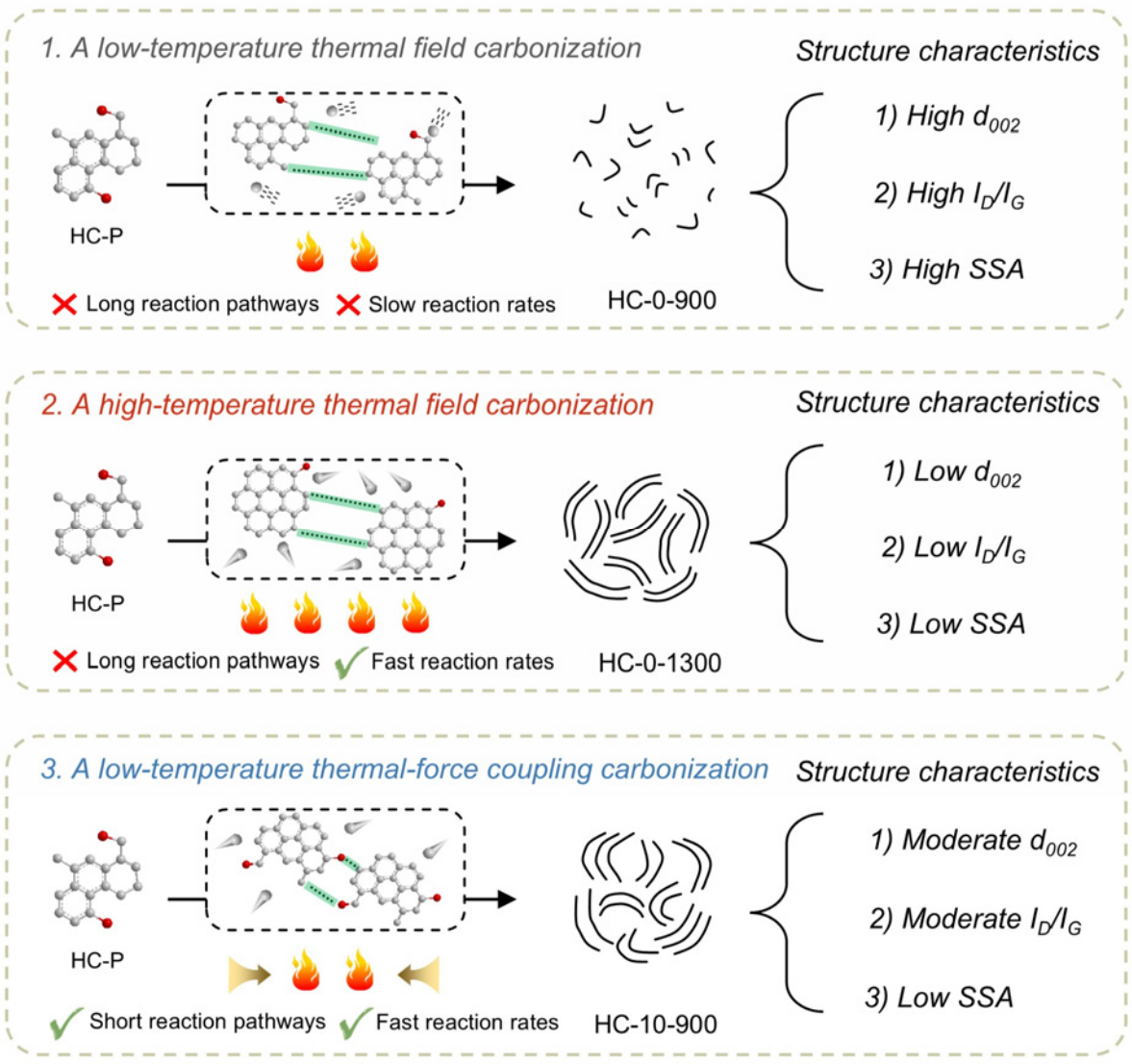

Figure R5. The relationship between the preparation process and structure characteristics of HC.

The accuracy and reliability of the structure parameter ranges obtained through ML need to be further validated through experimentation (**Figure R5**). The selection of process parameters relies on structure design principles derived from ML. The low SSA (0.26-17.84 m^2 g^{-1}) indicates that the HC exhibits a densified block-like morphology. Moderate d_{002} (0.376-0.394 nm) and Moderate I_D/I_G (1.10-2.30) correspond to moderate carbonization temperatures due to the negative correlation between carbonization temperature and d_{002} and I_D/I_G. The thermal-force coupling carbonization strategy simultaneously satisfies the dense block morphology and structure control of HC, thereby reducing the required carbonization temperature. Specifically, applying additional pressure in a low-temperature thermal field not only compacts the material into a dense block, significantly reducing the SSA, but also promotes the ordered growth of the carbon structure, keeping the I_D/I_G and d_{002} within reasonable ranges. Based on a combination of structure characterization, the structure

features of HC-10-900 with d_{002}=0.384 nm, I_D/I_G=1.33, and SSA=2.28 $m^2 g^{-1}$ conform to the range of structure parameters obtained through ML (**Figure R6**). Furthermore, the HC-10-900 exhibits excellent electrochemical performance in both button-cell and pouch-cell testing. Combined with structure characterization and performance testing, this further validates that the range of structure parameters obtained through ML is reliable and accurate in guiding the design of HC materials. Therefore, there is a closed-loop relationship between ML and experimental strategies in terms of guidance and validation.

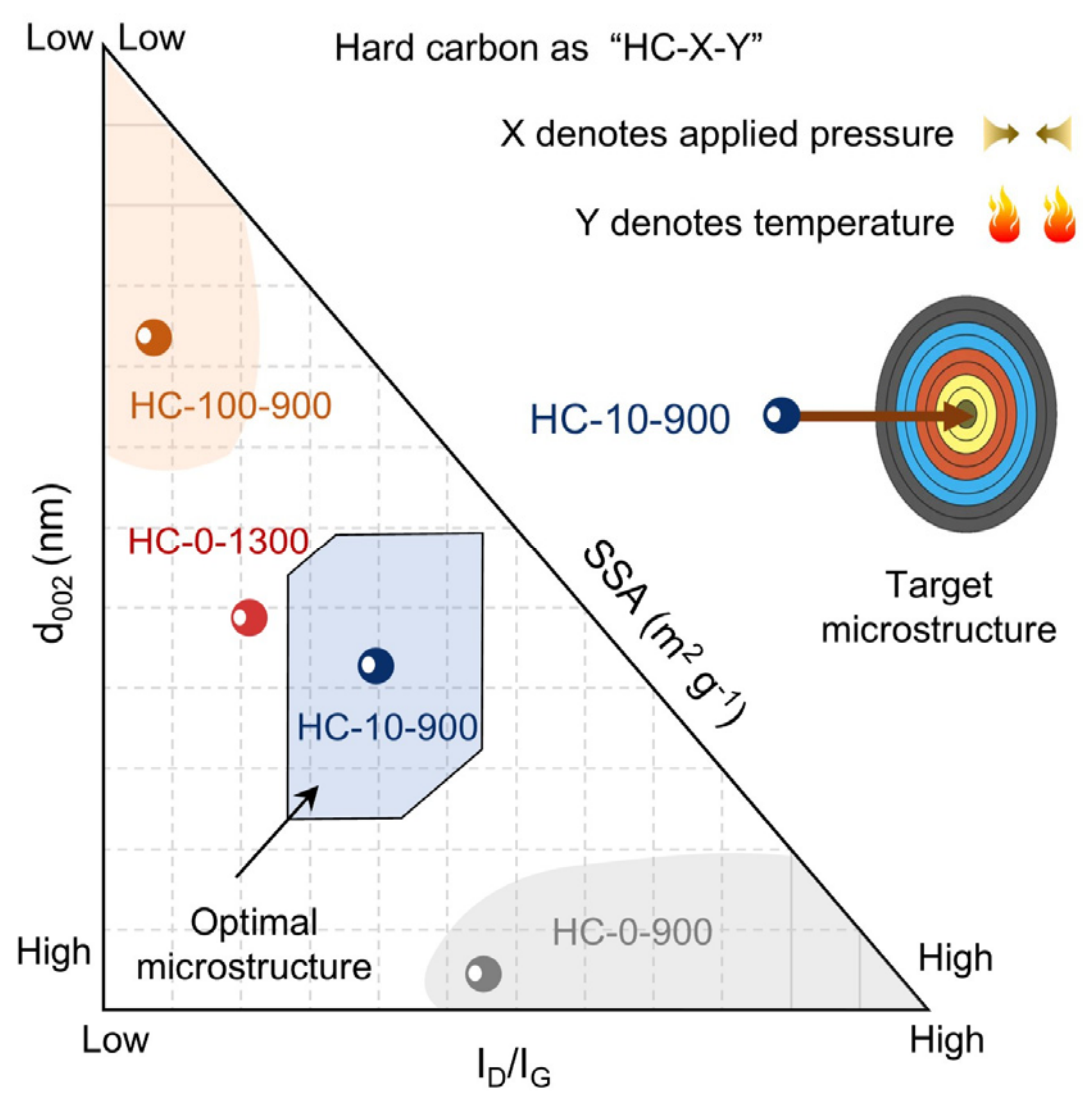

Figure R6. The screen of HC with target microstructures based on ML findings.

In this revision, we have further emphasized the distinctive features of ML and strengthened the connection between the machine learning and experimental sections.

Our revision:

1. Added detailed description.

p 9, line 174-177 (Results)

In contrast, thermal-force coupling carbonization not only compacts the material into a dense block, significantly reducing the SSA, but also promotes the slow growth of the carbon structure, keeping the I_D/I_G and d_{002} within reasonable ranges (Supplementary Fig. 12, 13).

p 11, line 217-219 (Results)

Comparing these quantitative parameters with the ML-defined microstructure target zone, it reveals that HC-10-900 satisfies the optimal combination of low SSA, moderate d_{002}, and moderate I_D/I_G (Fig. 2k, Table S3).

Supplementary Note 8 (Supplementary Information)

The increase in the R factor with rising pressure demonstrates that additional pressure promotes the development of structure graphitization due to the ordered and compact stacking of carbon layers. This has a similar promoting effect to raising the carbonization temperature, yet the pressure-induced effect is gradual and easier to control.

Comment #2. The manuscript presents "thermal-force coupling carbonization" as a new preparation paradigm. However, the method essentially corresponds to pressure-assisted or hot-press carbonization. Similar approaches have been widely used in powder metallurgy, carbon materials processing, and hot-press sintering. Moreover, it is not evident that this specific thermal–force coupling strategy is necessary. Indeed, the authors themselves note that many recent studies already report high-performance hard carbon materials within the proposed structural window, but using various synthesis approaches.

Response: We sincerely appreciate the reviewers for raising a series of critical questions regarding thermal-force coupling carbonization. We fully agree with the view of reviewers that the essence of thermal-force coupling carbonization, pressure-assisted carbonization, and hot-press carbonization lies in the dual action of thermal and force fields, which are widely utilized in powder metallurgy, carbon materials processing, and hot-press sintering. Notably, hot-press sintering is primarily used to enhance the density and eliminate internal porosity of materials in the field of powder metallurgy. Thermal-force coupling carbonization focuses not only on densifying the material to reduce the SSA but, more importantly, on the regulation of microstructure, including carbon layer stacking, pore restructuring, and defect coupling. Therefore, thermal-force coupling carbonization differs significantly from hot-press sintering in powder metallurgy in terms of regulating the microstructure characteristics of HC and elucidating the mechanisms underlying microstructure evolution. Next, we will explain the necessity of thermal-force coupling carbonization in terms of structure, preparation, mechanism, performance, and sustainability.

Structure: A range of structure parameters identified as promising based on ML, characterized by low SSA (0.26-17.84 $m^2 g^{-1}$), moderate d_{002} (0.376-0.394 nm), and moderate I_D/I_G (1.10-2.30). This typically corresponds to relatively high carbonization temperatures (1200-1800°C). HC exhibits high SSA, high d_{002}, and high I_D/I_G at low carbonization temperatures (900°C), these values

do not fall within the range of structure parameters obtained through ML. Therefore, it is necessary to introduce an additional force field into the low-temperature thermal field to achieve the effects of a high-temperature thermal field (**Figure R5**).

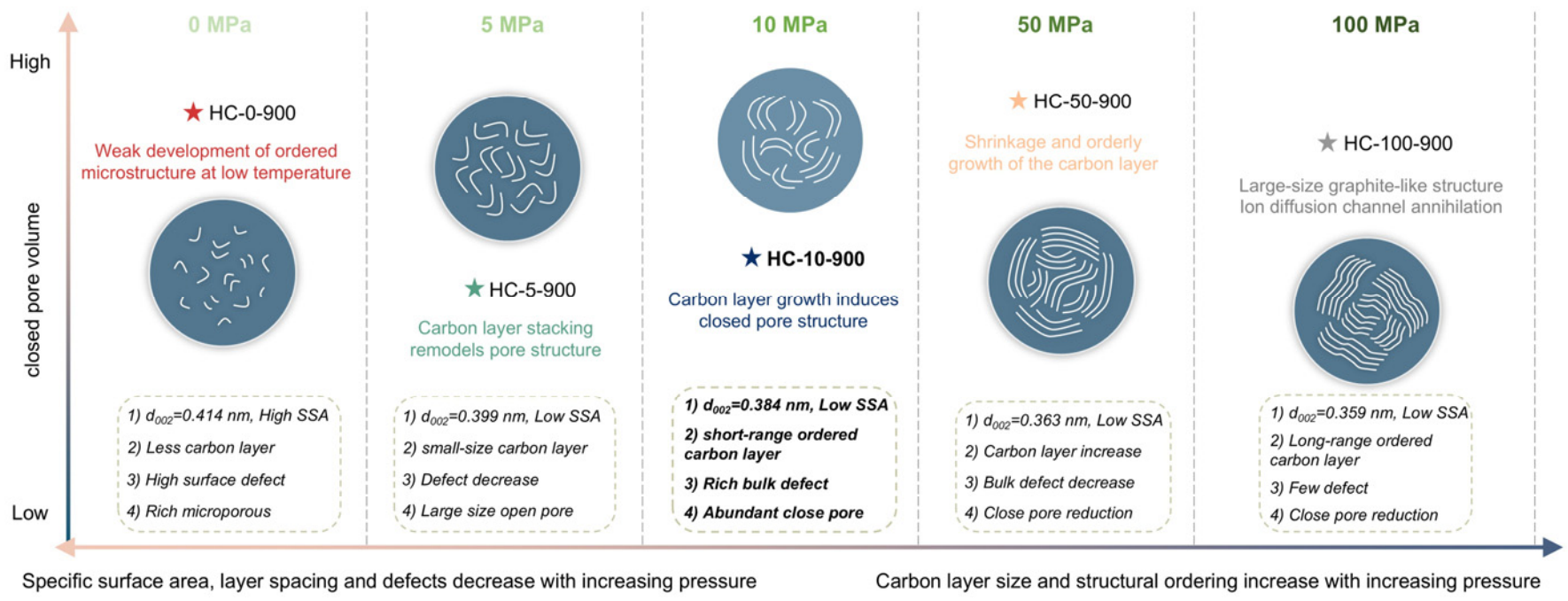

Figure R7. The evolution of HC microstructure under the low-temperature thermal-force coupling carbonization.

Preparation: The microstructure characteristics of HC are coordinated by maintaining a constant thermal field while simultaneously modulating the force field. Multidimensional structure characterization of HC confirms that as pressure increases, the number of stacked layers increases, the interlayer spacing gradually decreases, the structure evolves toward a graphitic state, and the volume of closed pores first increases and then decreases (**Figure R7**). These structure trends are consistent with those observed at increased carbonization temperatures. Among them, HC-10-900 microstructure with d_{002}=0.384 nm, I_D/I_G=1.33, and SSA=2.28 m^2 g^{-1} under a low temperature of 900°C with a pressure of 10 MPa, which falls within the range of structure parameters obtained through ML.

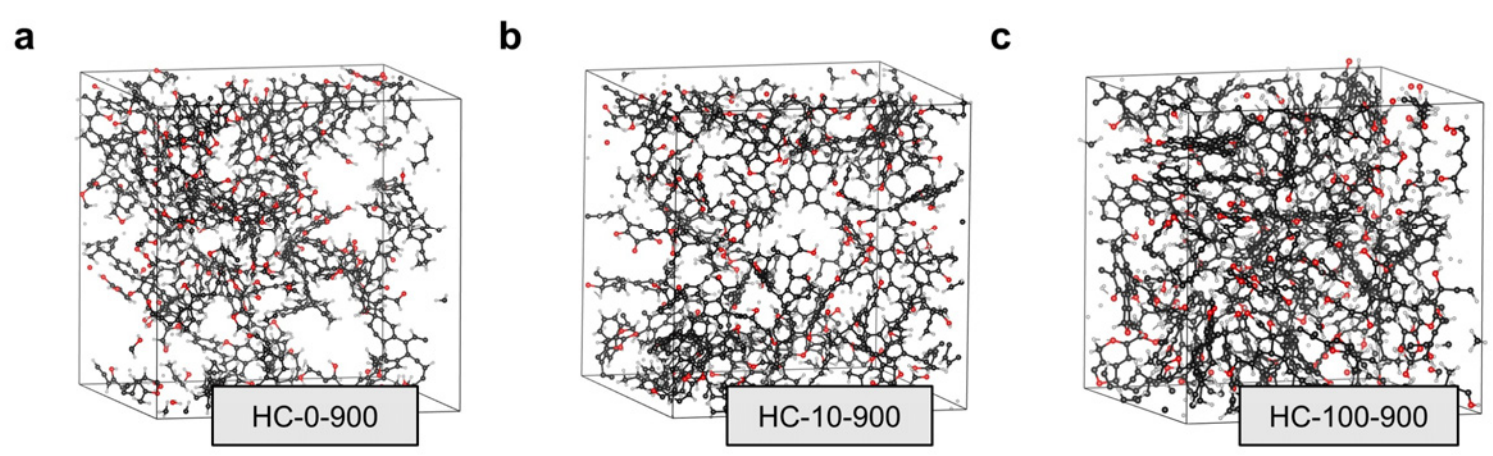

Figure R8. Molecular dynamics simulations of HC-0-900, HC-10-900, and HC-100-900.

Mechanism: There are currently few reports on the coupling mechanisms between thermal and force fields and the microstructure of HC. This study elucidated the mechanisms underlying the microstructure evolution of HC during thermal-force coupling carbonization processes through

multiscale structure characterization and molecular dynamics simulations (**Figure R8**). Under the thermal-force coupling effect, the short carbon fragments accelerate cross-linking to promote the epitaxial growth of the carbon layer and form a closed-pore structure between the curved pseudo-graphite microdomains.

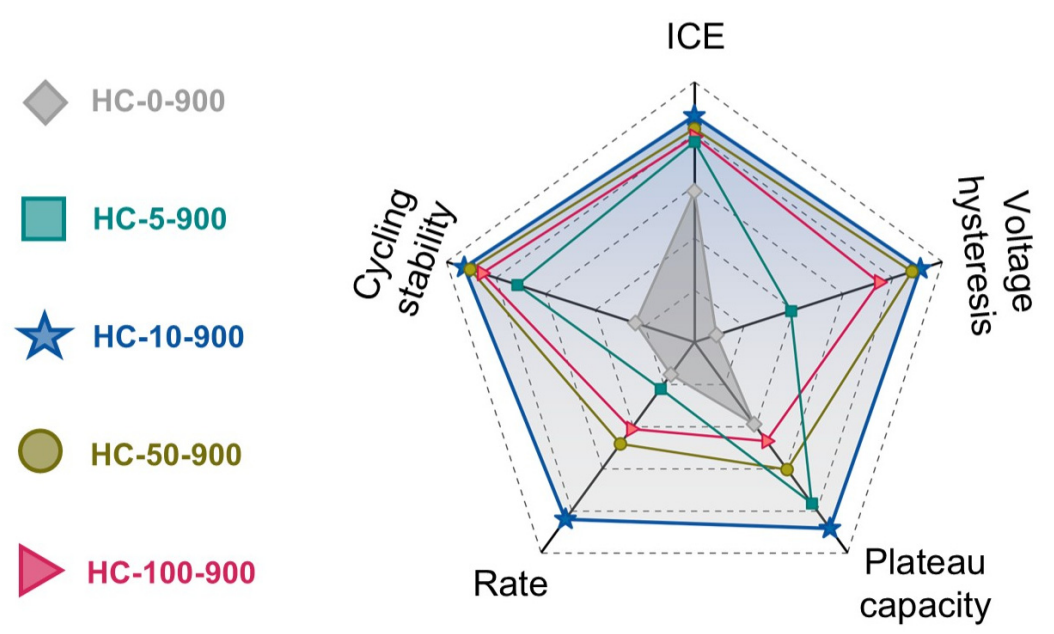

Figure R9. Comparison of electrochemical performance.

Performance: The HC-10-900 with targeted microstructure demonstrated high ICE, high reversible plateau capacity, high rate capability, and excellent cycling stability in half-cell testing (**Figure R9**). Moreover, the assembled Ah-level sodium-ion pouch cells with the HC-10-900 as anode also delivered high energy density and outstanding long-term cycling performance. This further validates the reliability of the ML findings and the feasibility of thermal-force coupling carbonization.

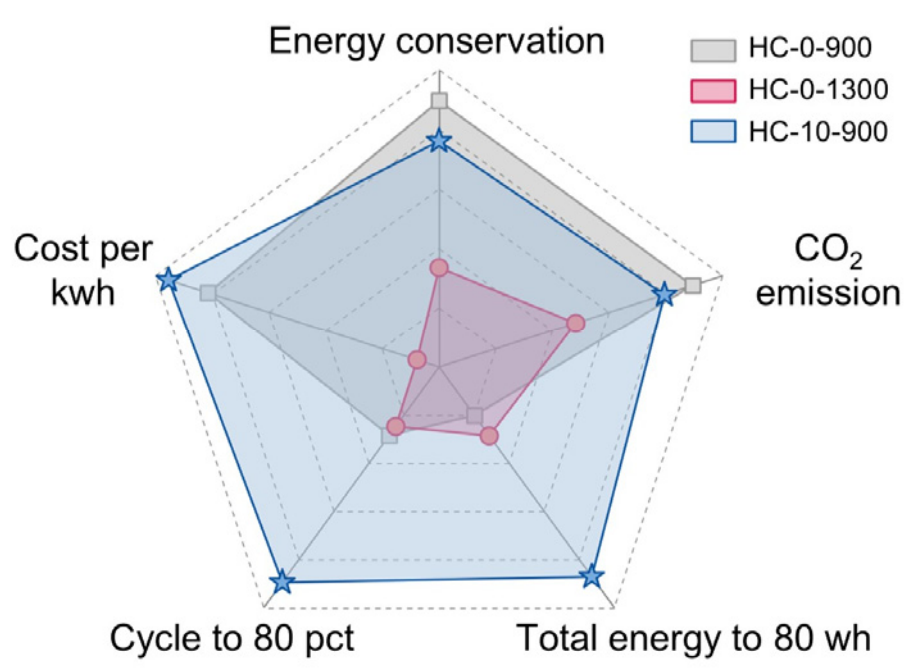

Figure R10. Comparison of the different preparation processes.

Sustainability: The study compared the advantages of low-temperature thermal field carbonization, low-temperature thermal-force coupling carbonization (LTFC), and high-temperature thermal field carbonization for the small-scale laboratory production of HC. In particular, thermal-force coupling carbonization has demonstrated significant advantages in

reducing energy consumption, increasing yield, and lowering carbon emissions (**Figure R10**). Through its high-value products and efficient process, LTFC achieves the dual improvements of environmental and economic benefits, which have significant potential for widespread applications.

Therefore, thermal-force coupling carbonization is of great significance for validating ML conclusions, decreasing carbonization temperatures, enabling the controlled production of HC, and enhancing economic efficiency.

We fully agree that recent research has reported numerous synthetic methods for preparing HC with the desired structure, such as pre-oxidation, chemical cross-linking, the template method, induced catalysis, and heteroatom doping (***Angew. Chem. Int. Ed. 2026,*** 65, 11, e25941; ***ACS Nano 2024***, 18, 18, 11941-11954; ***Energy Storage Mater. 2025***, 75, 104031). These methods focus on modifying the precursors prior to pyrolysis or optimizing the precursor structure during the pyrolysis process. This is further combined with thermal field carbonization to achieve the regulation of HC microstructures. These synthesis methods do not rely heavily on process parameters but instead depend on modification strategies, which often increase the cost of producing HC. Moreover, some modification strategies involve pretreatment through air oxidation and post-treatment of acidic and alkaline wastewater, which also have environmental impacts. Recently reported synthetic methods involving transient temperature treatment, flash carbonization, and Joule heating have demonstrated the precise design of HC microstructures on an extremely short timescale (***Adv. Funct. Mater. 2025,*** 35, 29, 2423559; ***Energy Storage Mater. 2026,*** 86, 104929; ***Chem. Eng. J. 2024***, 496, 15, 154103). The essence of these methods lies in rapidly heating the material to high temperatures within an extremely short time through the coupling of thermal and electric fields, thereby achieving a metastable state in which HC defects coexist with a locally graphitized structure. This exhibits high efficiency and low energy consumption in the preparation of HC with a targeted structure. In this study, HC with a targeted microstructure was prepared using low-temperature thermal-force coupling carbonization. On the one hand, HC is produced by adjusting process parameters to simplify the production process and reduce costs. On the other hand, the thermal-force coupling is utilized to reduce the carbonization temperature, reducing energy consumption and improving yield.

Our revision:

1. Added detailed description and references.

p 4, line 67-72 (Introduction)

Recently, structure modification strategies combined with high-temperature carbonization (pre-oxidation, molecular cross-linking, and heteroatom doping) are widely used to construct Na^+ diffusion pathways and sodium storage sites, achieving high initial Coulombic efficiency (ICE) and reversible plateau capacity of HC[16-18]. Due to the inability of these modification strategies to significantly suppress high-temperature-driven structure graphitization, the Na^+ diffusion kinetics often fail to match the abundance of storage sodium sites, resulting in a bottleneck characterized by "abundant sites but slow diffusion"[19].

p 9, line 174-177 (Results)

In contrast, thermal-force coupling carbonization not only compacts the material into a dense block, significantly reducing the SSA, but also promotes the slow growth of the carbon structure, keeping the I_D/I_G and d_{002} within reasonable ranges (Supplementary Fig. 12, 13).

p 9, line 183-185 (Results)

This morphological transition results from thermal-force coupling effects that promote the rearrangement of the carbon framework and interlayer slippage, which facilitates the reduction of SSA.

p 11, line 215-217 (Results)

The I_D/I_G and SSA decrease progressively with increasing pressure, which is attributed to the densification of the material, where surface-exposed defects are gradually incorporated into the bulk defects.

p 11, line 230-233 (Results)

The changes in open-pore and closed-pore structures indicate that the disordered carbon layers at low pressure are rearranged to form new pores, which subsequently transform into a low-porosity, ordered graphitic arrangement at high pressure.

p 29, line 636-645 (References)

[16] Liu, H. et al. Highways construction in amorphous carbon anode enables 10C fast-charging sodium-ion batteries. *Angew. Chem. Int. Ed.* **65**, e25941 (2026).

[17] Qiu, C. et al. One-step construction of closed pores enabling high plateau capacity hard carbon anodes for sodium-ion batteries: Closed-pore formation and energy storage mechanisms. *ACS Nano* **18**, 11941-11954 (2024).

[18] Wang, F. et al. Revitalizing sodium-ion batteries via controllable microstructures and advanced

electrolytes for hard carbon. *eScience* **4**, 100181 (2024).

[19] Xu, Y-J. et al. Cellulose-grafting boosted pyrolysis nucleation: Achieving low-temperature construction of hard carbon anodes with long low-voltage plateau and ultrafast Na storage kinetics. *Energy Storage Mater.* **75**, 104031 (2025).

Comment #3. This work repeated claim that high-temperature carbonization inevitably leads to strong trade-offs, while most comparisons are only made against HC-0-1300, which represents a relatively modest anode without optimization. However, recent advances in hard carbon synthesis—including transient temperature processing, flash carbonization, and Joule-heating-based synthesis—have demonstrated the ability to precisely engineer carbon microstructures within extremely short timescales while maintaining high energy efficiency. These approaches are not discussed or benchmarked in the manuscript.

Response: We greatly appreciate this highly constructive feedback from the reviewers. We strongly agree that comparing performance and manufacturing processes solely against the traditional process benchmark of HC-0-1300 is insufficient.

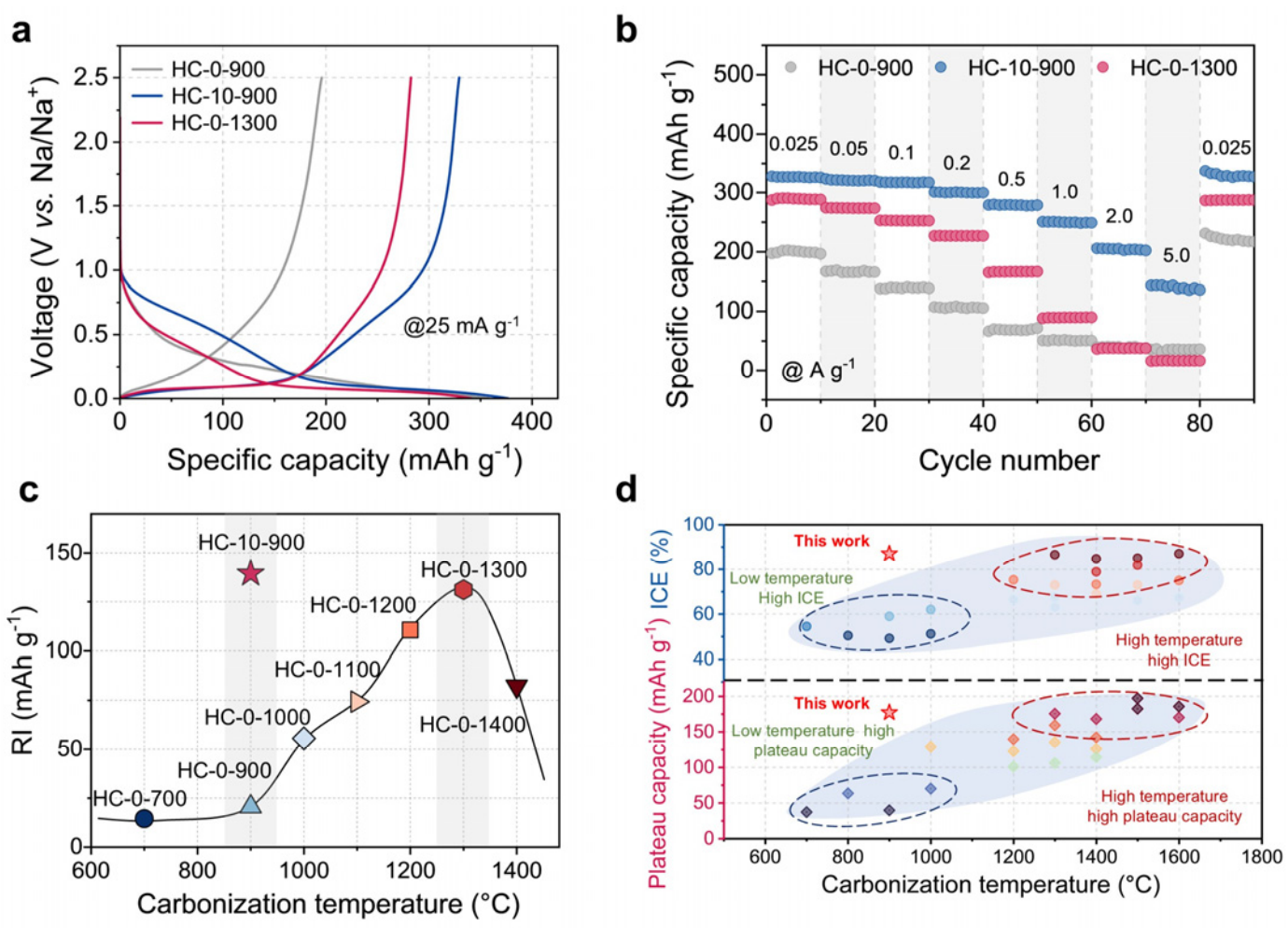

Figure R11. (a) Initial charging and discharging curves. (b) Rate performance. (c) RI of samples at different carbonization temperatures. (d) Comparison of ICE, plateau capacity, and carbonization temperature of HC-10-900 and other reported carbon materials.

In this study, the microstructure of the HC was primarily regulated through the optimization of process parameters. Single thermal field carbonization served as the control group, while low-temperature thermal coupling carbonization served as the experimental group. Maintaining

consistent precursors without modification, we compared the electrochemical performance of HC prepared at different carbonization temperatures with and without applied pressure to highlight the advantages of low-temperature thermal-force coupling carbonization. Moreover, the performance of HC prepared via low-temperature thermal-force coupling carbonization was compared with that of HC prepared at different carbonization temperatures based on different precursors and modification strategies (**Figure R11**). The results indicate that the ICE and reversible plateau capacity of HC are positively correlated with carbonization temperature under conventional thermal-field-driven carbonization processes. The high ICE and high reversible plateau capacity depend on high carbonization temperatures. Notably, HC prepared via low-temperature thermal-force coupling carbonization exhibits microstructure characteristics similar to those of HC prepared in a high-temperature thermal field, thereby enabling high ICE and high reversible plateau capacity at low carbonization temperatures. Besides, hard carbon prepared via low-temperature thermal-force coupling carbonization also provides excellent rate capability and cycle stability due to improved diffusion kinetics. Therefore, the thermal-force coupling synergy strategy significantly reduces the energy consumption of conventional processes under typical thermal field conditions, while simultaneously enabling the regulation of the microstructure of high-performance HC.

In recent years, the emerging ultra-rapid carbonization technology (transient temperature processing, flash carbonization, and Joule-heating-based synthesis) has been widely used in the production of HC due to its low energy consumption, high efficiency, and precise control capabilities (***Adv. Funct. Mater. 2025,*** 35, 29, 2423559; ***Chem. Eng. J. 2024***, 496, 15, 154103; ***Energy Storage Mater. 2026,*** 86, 104929). The rapid heating process prevents excessive graphitization of the structure, allowing the HC to form rich defects, abundant nanopores, and carbon layers with wide interlayer spacing at high carbonization temperatures (**Table R1**). This metastable structure combines high reversible sodium storage sites and fast diffusion kinetics, resulting in excellent overall electrochemical performance in HC. This metastable structure significantly increases the number of reversible sodium storage sites in HC at high carbonization temperatures and improves the kinetics of Na^+ diffusion. However, because these emerging technologies heat up too quickly, it is difficult to control the degree of graphitization in localized structures. The HC structure primarily exhibits long-range ordered carbon layers and a large-volume closed-pore structure. This structure achieves ultra-high ICE and high reversible plateau capacity,

but capacity also declines significantly at high rates, although rate performance has improved compared to conventional processes. Furthermore, the precision with which rapid carbonization technology regulates HC structures depends on the sophisticated level of the equipment.

Table R1. Comparison of low-temperature thermal-force coupling carbonization with current synthesis strategies and ultrafast carbonization technologies.

Anode	Modification strategies	Process Conditions	Structural Features	Electrochemical performance	Ref
Modification strategies based on traditional thermal field carbonization					
TC1500	Pore engineering	5°C min^{-1}<br>900°C 2h<br>1500°C 2h	d_{002}: 0.367 nm<br>I_D/I_G: 1.14<br>SSA: 1.8 m^2 g^{-1}	75.6% at 20 mA g^{-1}<br>331.51 mAh g^{-1}<br>131.4mAh g^{-1} at 3 A g^{-1}	61
HC-Pc + 1.0 K	Molten activation	5°C min^{-1}<br>300°C 1h<br>1600°C 2h	d_{002}: 0.382 nm<br>I_D/I_G: 1.29<br>SSA: 0.1 m^2 g^{-1}	84.4% at 30 mA g^{-1}<br>357.0 mAh g^{-1}<br>40.5mAh g^{-1} at 1 A g^{-1}	62
ZAHC-1500	Catalytic engineering	5°C min^{-1}<br>500°C 1h<br>2°C min^{-1}<br>1500°C 2h	d_{002}: 0.376 nm<br>I_D/I_G: 2.80<br>SSA: 3.7 m^2 g^{-1}	80.9% at 20 mA g^{-1}<br>342.9 mAh g^{-1}<br>111.9mAh g^{-1} at 1 A g^{-1}	63
CSM-0.8	Pre-oxidation	5°C min^{-1}<br>300°C 1h<br>1200°C 2h	d_{002}: 0.391 nm<br>I_D/I_G: 2.68<br>SSA: 3.0 m^2 g^{-1}	87.9% at 30 mA g^{-1}<br>339.3 mAh g^{-1}<br>99.1mAh g^{-1} at 0.6 A g^{-1}	64
HC-1.5	Heteroatom doping	3°C min^{-1}<br>600°C 2h<br>1200°C 2h	d_{002}: 0.365 nm<br>I_D/I_G: 1.12<br>SSA: 45 m^2 g^{-1}	68.1% at 30 mA g^{-1}<br>299.7 mAh g^{-1}<br>212mAh g^{-1} at 2 A g^{-1}	65
Emerging ultrafast synthesis technologies					
HC-S1200	-	120°C min^{-1}<br>1200°C 5min<br>25 MPa	d_{002}: 0.388 nm<br>I_D/I_G: 1.15<br>SSA: 8.7 m^2 g^{-1}	92% at 30 mA g^{-1}<br>340.9 mAh g^{-1}<br>145.8mAh g^{-1} at 3 A g^{-1}	66
HC600-J-1500	-	5°C min^{-1}<br>600°C 3h<br>1500°C	d_{002}: 0.393 nm<br>I_D/I_G: 2.40<br>SSA: 3.0 m^2 g^{-1}	92.4% at 25 mA g^{-1}<br>362.0 mAh g^{-1}<br>135.0mAh g^{-1} at 2 A g^{-1}	67
HC (30s)	-	5°C min^{-1}<br>800°C 3h<br>1550°C	d_{002}: 0.362 nm<br>I_D/I_G: 1.16<br>SSA: 8.3 m^2 g^{-1}	88.4% at 30 mA g^{-1}<br>289.8 mAh g^{-1}<br>187.5mAh g^{-1} at 1.5 A g^{-1}	2
CTSFS 1300	-	20 MPa<br>1300°C 10s	d_{002}: 0.398 nm<br>I_D/I_G: 1.13<br>SSA: 7.3 m^2 g^{-1}	58.8% at 50 mA g^{-1}<br>303.3 mAh g^{-1}<br>240.5mAh g^{-1} at 2 A g^{-1}	68
HC-10-900	-	5°C min^{-1}<br>300°C 1h<br>900°C 2h<br>10MPa	d_{002}: 0.384 nm<br>I_D/I_G: 1.33<br>SSA: 2.3 m^2 g^{-1}	87.4% at 25 mA g^{-1}<br>329.3 mAh g^{-1}<br>205.1mAh g^{-1} at 2 A g^{-1}<br>143.5mAh g^{-1} at 5 A g^{-1}	This work

Although emerging rapid carbonization technologies show great potential for overcoming the high-temperature trade-off, they are often limited by equipment complexity and process versatility. The low-temperature thermal-force coupling carbonization strategy proposed in this study achieves a balance between low energy consumption and high performance through the synergistic use of pressure and temperature under conventional equipment and mild process conditions.

Our revision:

1. Added detailed description and references.

p 4, line 72-74 (Introduction)

To optimize the carbonization process, emerging rapid carbonization technologies (thermal shock, Joule heating, and flash carbonization) are employed to construct a wide "highway" in HC for Na^+ diffusion[20-22].

p 4, line 74-76 (Introduction)

These technologies prevent the compact stacking of the carbon layer structure through short-duration high-temperature treatment and ultra-rapid heating rates, thereby optimizing Na^+ diffusion kinetics.

p 4, line 76-79 (Introduction)

Notably, the HC obtained through rapid carbonization typically exhibits long-range ordered carbon layers and a large-volume closed-pore structure, which further increases the sodium storage capacity[23,24]. And the improved Na^+ diffusion rate remains insufficient due to excessive packing and the long-range diffusion of Na^+.

p 13, line 281-284 (Results)

By breaking this temperature-performance constraint, the thermal-force strategy enables HC-10-900 to achieve unprecedented comprehensive electrochemical performance at substantially reduced temperatures (Supplementary Fig. 43).

p 14, line 284-286 (Results)

Moreover, compared with reported modification strategies and ultrafast synthesis techniques, thermal-force coupling carbonization also offers unique advantages (Table S6).

p 30, line 646-655 (References)

[20] Cui, J. et al. Data-driven intelligent carbonization unifies diverse biomass into high-

performance hard carbon negative electrodes. *Nat Commun.* (2026).

[21] Qiu, Y. et al. Rapid closed pore regulation of biomass-derived hard carbons based on flash joule heating for enhanced sodium ion storage. *Adv. Funct. Mater.* **35**, 2423559 (2025).

[22] Wang, H. et al. Thermal shock reducing amorphous carbon ratio in hard carbon for improved rate capability of sodium ion storage. *Carbon* **229**, 119528 (2024).

[23] Song, Z. et al. Joule heating for structure reconstruction of hard carbon with superior sodium ion storage performance. *Chem. Eng. J.* **496**, 154103 (2024).

[24] Luo, F. et al. Rapid flash heating of hard carbon: Enhanced sodium storage via hierarchical graphitization. *Energy Storage Mater*. **86**, 104929 (2026).

2. Added tables.

Supplementary Table 6. Comparison of low-temperature thermal-force coupling carbonization with current synthesis strategies and ultrafast carbonization technologies.

Anode	Modification strategies	Process Conditions	Structural Features	Electrochemical performance	Ref
Modification strategies based on traditional thermal field carbonization					
TC1500	Pore engineering	5°C min^{-1}<br>900°C 2h<br>1500°C 2h	d_{002}: 0.367 nm<br>I_D/I_G: 1.14<br>SSA: 1.8 m^2 g^{-1}	75.6% at 20 mA g^{-1}<br>331.51 mAh g^{-1}<br>131.4mAh g^{-1} at 3 A g^{-1}	61
HC-Pc + 1.0 K	Molten activation	5°C min^{-1}<br>300°C 1h<br>1600°C 2h	d_{002}: 0.382 nm<br>I_D/I_G: 1.29<br>SSA: 0.1 m^2 g^{-1}	84.4% at 30 mA g^{-1}<br>357.0 mAh g^{-1}<br>40.5mAh g^{-1} at 1 A g^{-1}	62
ZAHC-1500	Catalytic engineering	5°C min^{-1}<br>500°C 1h<br>2°C min^{-1}<br>1500°C 2h	d_{002}: 0.376 nm<br>I_D/I_G: 2.80<br>SSA: 3.7 m^2 g^{-1}	80.9% at 20 mA g^{-1}<br>342.9 mAh g^{-1}<br>111.9mAh g^{-1} at 1 A g^{-1}	63
CSM-0.8	Pre-oxidation	5°C min^{-1}<br>300°C 1h<br>1200°C 2h	d_{002}: 0.391 nm<br>I_D/I_G: 2.68<br>SSA: 3.0 m^2 g^{-1}	87.9% at 30 mA g^{-1}<br>339.3 mAh g^{-1}<br>99.1mAh g^{-1} at 0.6 A g^{-1}	64
HC-1.5	Heteroatom doping	3°C min^{-1}<br>600°C 2h<br>1200°C 2h	d_{002}: 0.365 nm<br>I_D/I_G: 1.12<br>SSA: 45 m^2 g^{-1}	68.1% at 30 mA g^{-1}<br>299.7 mAh g^{-1}<br>212mAh g^{-1} at 2 A g^{-1}	65
Emerging ultrafast synthesis technologies					
HC-S1200	-	120°C min^{-1}<br>1200°C 5min<br>25 MPa	d_{002}: 0.388 nm<br>I_D/I_G: 1.15<br>SSA: 8.7 m^2 g^{-1}	92% at 30 mA g^{-1}<br>340.9 mAh g^{-1}<br>145.8mAh g^{-1} at 3 A g^{-1}	66

HC600-J-1500	-	5°C min^{-1}<br>600°C 3h<br>1500°C	d_{002}: 0.393 nm<br>I_D/I_G: 2.40<br>SSA: 3.0 m^2 g^{-1}	92.4% at 25 mA g^{-1}<br>362.0 mAh g^{-1}<br>135.0mAh g^{-1} at 2 A g^{-1}	67
HC (30s)	-	5°C min^{-1}<br>800°C 3h<br>1550°C	d_{002}: 0.362 nm<br>I_D/I_G: 1.16<br>SSA: 8.3 m^2 g^{-1}	88.4% at 30 mA g^{-1}<br>289.8 mAh g^{-1}<br>187.5mAh g^{-1} at 1.5 A g^{-1}	2
CTSFS 1300	-	20 MPa<br>1300°C 10s	d_{002}: 0.398 nm<br>I_D/I_G: 1.13<br>SSA: 7.3 m^2 g^{-1}	58.8% at 50 mA g^{-1}<br>303.3 mAh g^{-1}<br>240.5mAh g^{-1} at 2 A g^{-1}	68
HC-10-900	-	5°C min^{-1}<br>300°C 1h<br>900°C 2h<br>10MPa	d_{002}: 0.384 nm<br>I_D/I_G: 1.33<br>SSA: 2.3 m^2 g^{-1}	87.4% at 25 mA g^{-1}<br>329.3 mAh g^{-1}<br>205.1mAh g^{-1} at 2 A g^{-1}<br>143.5mAh g^{-1} at 5 A g^{-1}	This work

Comment #4. When discussing sustainability or energy consumption, the authors claim to lower the carbonization temperature from 1300 °C to 900 °C. However, this relies on high-pressure hot pressing, which itself requires additional energy input and costly specialized equipment. More importantly, hot pressing presents substantial challenges for industrial-scale production, particularly in terms of throughput and continuous processing. The TEA and economic analysis are significantly flawed by only considering 10-50 g material. Such data is not reliable for evaluating scale-up feasibility and is far from industry practices.

Response: We sincerely appreciate the insightful questions of reviewers regarding industrial-scale production and economic feasibility. We strongly agree with lowering the carbonization temperature of HC from 1300°C to 900°C, which relies on the application of an additional force field, thereby increasing energy consumption. Traditional hot-press carbonization furnaces possess integrated high-temperature and high-pressure capabilities, resulting in a complex equipment design and high energy consumption. Since the preparation of HC-10-900 requires only a relatively low pressure of 10 MPa, a dedicated hot-press carbonization furnace is not necessary. The preparation of HC-10-900 relies on two independent and synergistic systems: first, a conventional muffle furnace to provide a thermal field of 900°C, and second, a high-temperature alloy apparatus to deliver a pressure of 10 MPa via gravitational force. The equipment requirements and energy consumption for this process are significantly lower than those of a hot-press carbonization furnace, while enabling a reduction in the carbonization temperature from 1300°C to 900°C. It is undeniable that

there are significant challenges in the throughput and continuous production of thermal-force coupling carbonization at the scale of mass production, due to limitations in the space of hot-pressing molds and laboratory conditions.

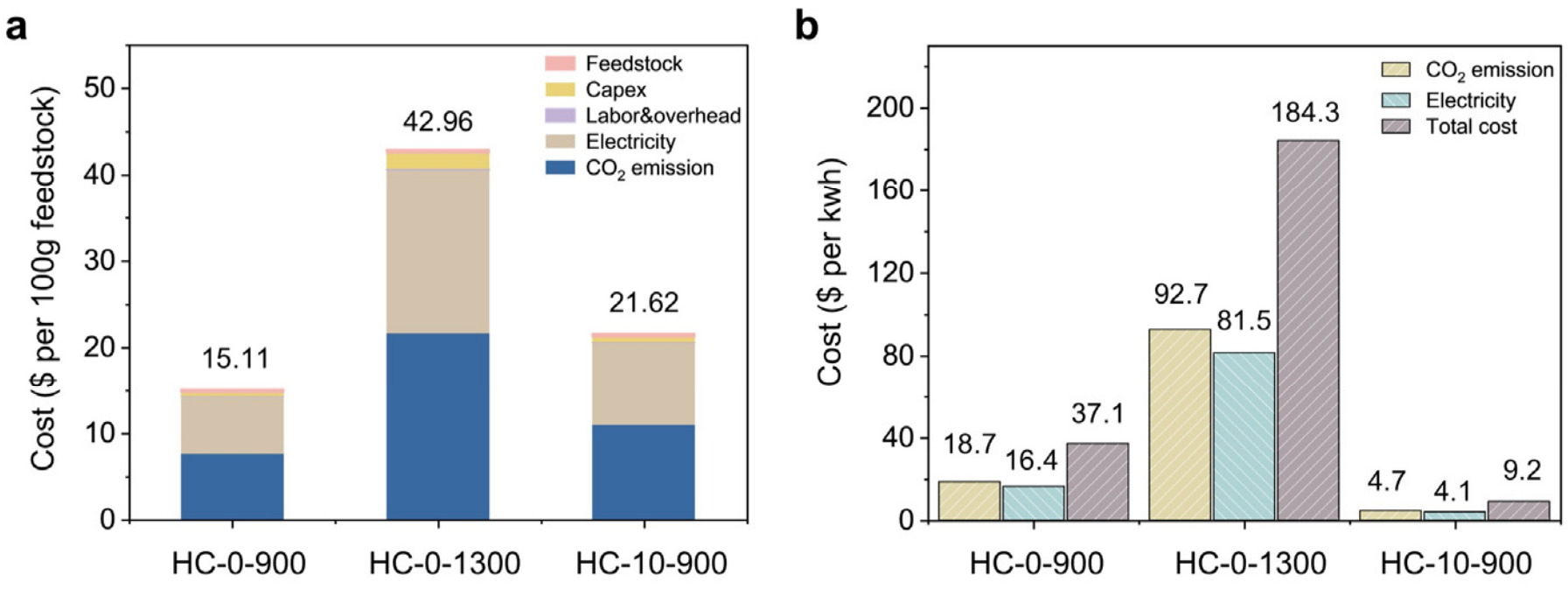

Figure R12. (a) Cost of preparing HC-0-900, HC-0-1300, and HC-10-900 using 100g feedstock. (b) The cost per kWh required in the full battery systems assembled in HC-0-900, HC-0-1300, and HC-10-900, respectively.

We strongly agree that evaluating large-scale production using only 10–50 g of material is insufficient. It is difficult to assess the scale-up potential of low-temperature thermal-force coupling carbonization through economic analysis due to limitations in laboratory conditions and mold space. In the economic analysis section, our process parameters, energy consumption, CO_2 emissions, and yield were all obtained from actual experiments to better assess the real differences between various preparation methods. Due to the experimental procedures and material preparation capabilities being limited to the laboratory small-scale trial stage, the economic benefits and environmental impacts obtained are more representative of assessments conducted for small-batch laboratory production. Although thermal-force coupling carbonization is constrained by equipment and production capacity, its significantly high carbon yield, low CO_2 emissions, and high-value products compensate for these limitations (**Figure R12**).

In this study, the focus of the techno-economic analysis is not on predicting the absolute costs and benefits of commercialization, but on identifying the relative strengths and weaknesses of different technological pathways under the same constraints. Current laboratory data cannot be directly equated with industrial-scale production. When scaling up from the laboratory to the factory, all technical approaches face challenges posed by the scaling effect. Within the current comparative framework, we assume that the technical approaches under comparison face similar levels of

engineering complexity during the scaling-up process. Since the cost of low-temperature thermal-force coupling carbonization is significantly lower than that of single-thermal-field carbonization at the laboratory stage, and the difficulty of scaling up both processes is comparable, if the costs of both increase by the same margin after industrialization, low-temperature thermal-force coupling carbonization will still maintain a cost advantage over single-thermal-field carbonization. Therefore, the relative rankings derived from the techno-economic analysis are robust in qualitative terms.

Our revision:

1. Added detailed description.

p 5, line 102-104 (Introduction)

Based on batch processing in the laboratory, low-temperature thermal-force (900°C-10 MPa) coupling carbonization significantly reduces energy consumption by 49.7% and carbon emission by 47.5% compared to traditional high-temperature (1300°C) carbonization.

p 19, line 390-392 (Results)

Within the current comparative framework, we assume that the three production technology pathways face similar levels of engineering complexity as they scale up.

p 20, line 405-406 (Results)

Through its high-value products and efficient process, LTFC achieves the dual improvements of environmental and economic benefits in the laboratory small-scale trial stage.

p 20, line 413-414 (Discussion)

Compared to traditional high-temperature (1300°C) derived HC, the HC-10-900 synthesized at 900°C under 10 MPa, simultaneously exhibits higher Na^+ storage sites and faster Na^+ diffusion kinetics, together with a large energy consumption reduction of 49.7% and a large carbon emission reduction of 47.5% at the laboratory small-scale trial stage.

Comment #5. While the pouch-cell performance is potentially promising, the origin and validity of the reported performance require further clarification. The detailed energy-density calculation should be defined, the cell-level parameters should be fully disclosed to enable fair comparison with previous reports, and to what extent the observed pouch-cell performance arises specifically from the proposed hard-carbon. Specifically, the ICE is only 87.4%. Was any prelithiation method used in a pouch cell to compensate? The areal density used (1.6 mg cm^{-2}) may be lower than typical

commercial Ah-level pouch cells (5-10 mg cm^{-2}).

Response: We are very grateful to the reviewers for their insightful questions regarding the details of the energy density calculations, areal density, and ICE compensation, which are crucial for assessing the practical value of this study. In response to your concerns, we have made the following comprehensive additions and clarifications in the revised draft.

Table R2. The specifications of HC-10-900//NFM pouch cell.

Cell component	Specification	Parameters
Anode (HC-10-900)	Active material mass loading (mg cm^{-2}, each side)	7.0
	Material mass (g)	7.25
	Size (mm × mm)	77*56
	Active material ratio	0.90
	Nickel tabs	0.21
Electrolyte	$NaPF_6$ (mol L^{-1})	1.0
	EC: DEC ratio	1:1
	Quality (g)	3.0
Separator (PP)	Size (mm × mm)	80*58
	Mass (g)	0.74
Cathode (NFM)	Active material mass loading (mg cm^{-2}, each side)	12.5
	Material mass	12.15
	Size (mm × mm)	75*56
	Active material ratio	0.96
	Aluminum tabs	0.06
Current collector	Aluminum foil (g)	2.58
Package (Al-plastic film)	Size (mm × mm)	90*74
	Mass (g)	1.86
Pouch cell	Total weight (g)	27.85
Pouch cell (1.5-3.95 V)	Discharge capacity (Ah)	1.47
	Specific energy density (Wh kg^{-1})	166.4
Pouch cell (1.5-4.3 V)	Discharge capacity (Ah)	1.90
	Specific energy density (Wh kg^{-1})	208.1

An assembled sodium-ion pouch cell primarily consists of the anode electrode, the cathode electrode, the aluminum current collector, the PP separator, the electrolyte, the aluminum tab, the nickel tab, and the aluminum-plastic film with pattern, with masses of 7.25 g, 12.15 g, 2.58 g, 0.74

g, 3.00 g, 0.06 g, 0.21 g, and 1.86 g, respectively (**Table R2**). The total weight of the sodium-ion pouch cell is 27.85 g. On the anode side, the active material mass loading is 7.0 mg cm^{-2}, the active material mass fraction is 90%, and the electrode dimensions are 77 mm × 56 mm. On the cathode side, the active material mass loading is 12.5 mg cm^{-2}, the active material mass fraction is 96%, and the electrode dimensions are 75 mm × 54 mm.

Based on the weight of the pouch cell, taking all components (electrode materials, binder, conductive agents, separator, electrolyte, tabs, current collectors, and Al plastic casing, etc) into account. The pouch cell provides a discharge capacity of 1.47 Ah at 1.5-3.95 V and a discharge energy density of 166.4 Wh kg^{-1} at 0.1C, and a discharge power density of 376.4 W kg^{-1} at 4.0C. The pouch cell provides a discharge capacity of 1.90 Ah at 1.5-4.3 V and a discharge energy density of 208.1 Wh kg^{-1} at 0.1C (**Figure R13**).

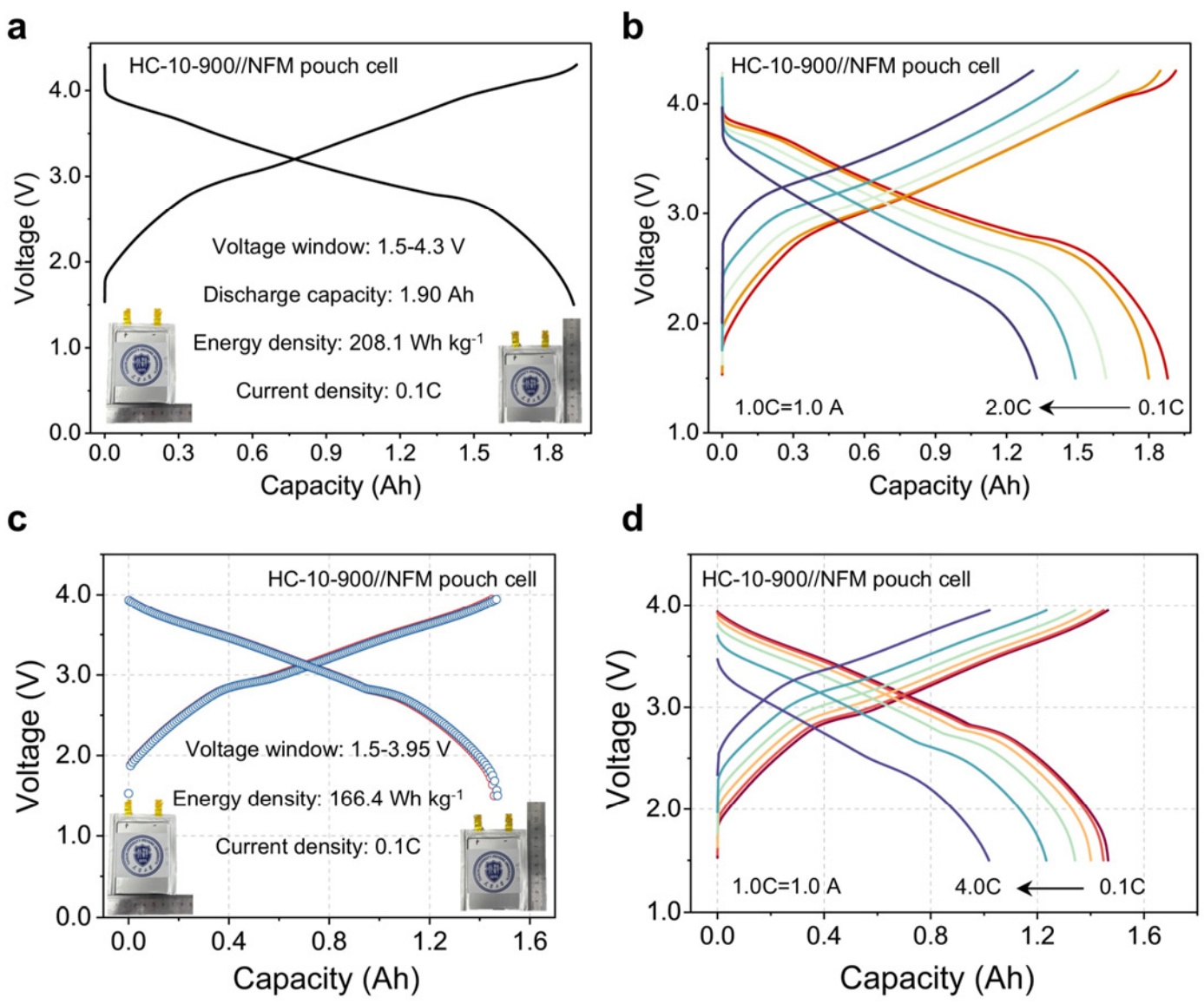

Figure R13. Charging and discharging curves at (a) 1.5-4.3 V and (c) 1.5-3.95 V of a sodium-ion pouch cell. Rate performance at (b) 1.5-4.3 V and (d) 1.5-3.95 V of the sodium-ion pouch cell.

The assembled sodium-ion pouch cells were not pre-sodiation beforehand. The assembled sodium-ion pouch cells were first aged (left to stand at 45°C for 24 h), then formed (charged at a constant current to 3.5 V at 0.1C), and finally aged (left to stand at room temperature for 24 h). Although the ICE of HC is only 87.4%, its efficiency can reach 98.9% during the second cycle. The HC with the areal loading of 1.6 mg cm^{-2} is primarily used in the assembly of coin-type sodium-ion batteries to evaluate their electrochemical performance. In the assembly of sodium-ion pouch cells, the areal loading of HC was increased to 7.0 mg cm^{-2} to enhance energy density, thereby evaluating

its potential for practical applications.

Table R3. Comparison of energy density in sodium-ion pouch cells.

Cathode	Anode	Electrolyte	Capacity (Ah)	Energy density ($Wh\ kg^{-1}$)	Ref.
$Na_{2/3}Ni_{1/3}Mn_{1/3}Ti_{1/3}O_2$	PFPI-13	1.0 M $NaPF_6$ in EC: DEC (1:1)	2.2	108.5	6
$NaNi_{1/3}Fe_{1/3}Mn_{1/3}O_2$	OP-HC	1.0 M $NaSO_3CF_3$ in DEGDME	1.239	150.0	4
$Na_3V_2(PO_4)_3$	PPHC900	1.0 M $NaClO_4$ in EC: DMC (1:1)	0.1	135.2	5
$Na_4Fe_3(PO_4)_2P_2O_7$	Metallic Sodium	1.0 M $NaPF_6$ in DEGDME	2.28	203.0	1
$Na_3V_2(PO_4)_3$	Metallic Sodium	NZSP–TLM	0.1145	220.0	69
$Na_3V_2(PO_4)_3$	HC (30 s)	1.0 M $NaPF_6$ in diglyme	--	189.3 (Based on active material mass)	2
$Na_3V_2(PO_4)_3$@C	CFG	1.0 M $NaClO_4$ in diglyme	--	144.0 (Based on active material mass)	3
$NaNi_{1/3}Fe_{1/3}Mn_{1/3}O_2$	TAC-1400	1.0 M $NaClO_4$ in EC: DEC (1:1)	0.4	254.3 (Based on active material mass)	70
$Na_3V_2(PO_4)_3$	G1500	1.0 M $NaPF_6$ in EC: DMC (1:1)	--	209.0 (Based on active material mass)	26
$Na_3V_2(PO_4)_3$	PAHC-1500	1.0 M $NaPF_6$ in diglyme	--	242.1 (Based on active material mass)	7
$NaNi_{1/3}Fe_{1/3}Mn_{1/3}O_2$	HC-10-900	1.0 M $NaPF_6$ in EC: DEC (1:1) (1.5-4.3 V)	1.90 at 0.1 A<br>1.33 at 2.0 A	208.1<br>129.7	This work
$NaNi_{1/3}Fe_{1/3}Mn_{1/3}O_2$	HC-10-900	1.0 M $NaPF_6$ in EC: DEC (1:1) (1.5-3.95 V)	1.47 at 0.1 A<br>1.23 at 2.0 A	166.4<br>124.3	This work

The sodium-ion pouch cells assembled using layered oxides ($NaNi_{1/3}Fe_{1/3}Mn_{1/3}O_2$) as the cathode material and other HC materials as the anode material were compared to highlight the advantages

of the HC anode in this study. Currently, the energy density of sodium-ion pouch batteries generally remains below 200 Wh kg^{-1}, with a maximum cycle life of 600 cycles and a capacity retention rate of less than 80%. This study benefits from the high sodium storage capacity and fast diffusion kinetics of the HC anode, which ensures that sodium-ion pouch cells deliver a high energy density of 208.1 Wh kg^{-1} at 1.5-4.3 V, a high energy density of 166.4 W kg^{-1} at 1.5-3.95 V, and high capacity retention of 96.1% after 1000 cycles (**Table R3**).

Our revision:

1. Added detailed description.

p 3, line 43-45 (Abstract)

Benefiting from this unprecedented low-temperature construction, the SIB pouch cell exhibits a milestone high energy density of 208.1 Wh kg^{-1} at 1.5-4.3 V, outperforming previously reported Ah-level SIBs.

p 15, line 308-311 (Results)

The HC-10-900//NFM pouch cell provided an impressive energy density of 208.1 Wh kg^{-1} at 1.5-4.3 V based on the entire device mass (Supplementary Fig. 52). Besides, the HC-10-900//NFM pouch cell provided a discharge capacity of 1.47 Ah at 1.5-3.95 V and an energy density of 166.4 Wh kg^{-1} (Fig. 3d).

p 15, line 313-314 (Results)

The capacity and energy density of HC-10-900//NFM pouch cell, surpassing previously reported Ah-level SIBs (Fig. 3f, Table S8)

p 20, line 415-416 (Discussion)

Notably, the Ah level HC-10-900//NFM pouch cell delivers a high energy density of 208.1 Wh kg^{-1} at 1.5-4.3 V based on the entire device mass, outperforming previously reported Ah-level SIBs.

Supplementary Note 15 (Supplementary Information)

An assembled sodium-ion pouch cell primarily consists of the anode electrode, the cathode electrode, the aluminum current collector, the PP separator, the electrolyte, the aluminum tab, the nickel tab, and the aluminum-plastic film with pattern, with masses of 7.25 g, 12.15 g, 2.58 g, 0.74 g, 3.00 g, 0.06 g, 0.21 g, and 1.86 g, respectively. The total weight of the sodium-ion pouch cell is 27.85 g. On the anode side, the active material mass loading is 6.3 mg cm^{-2}, the active material mass fraction is 90%, and the electrode dimensions are 77 mm × 56 mm. On the cathode side, the active

material mass loading is 12.5 mg cm^{-2}, the active material mass fraction is 96%, and the electrode dimensions are 75 mm × 54 mm.

Based on the weight of the pouch cell, taking all components (electrode materials, binder, conductive agents, separator, electrolyte, tabs, current collectors, and Al plastic casing, etc) into account. The pouch cell provides a discharge capacity of 1.47 Ah at 1.5-3.95 V and a discharge energy density of 166.4 Wh kg^{-1}. The pouch cell provides a discharge capacity of 1.90 Ah at 1.5-4.3 V and a discharge energy density of 208.1 Wh kg^{-1}.

2. Added figures and tables.

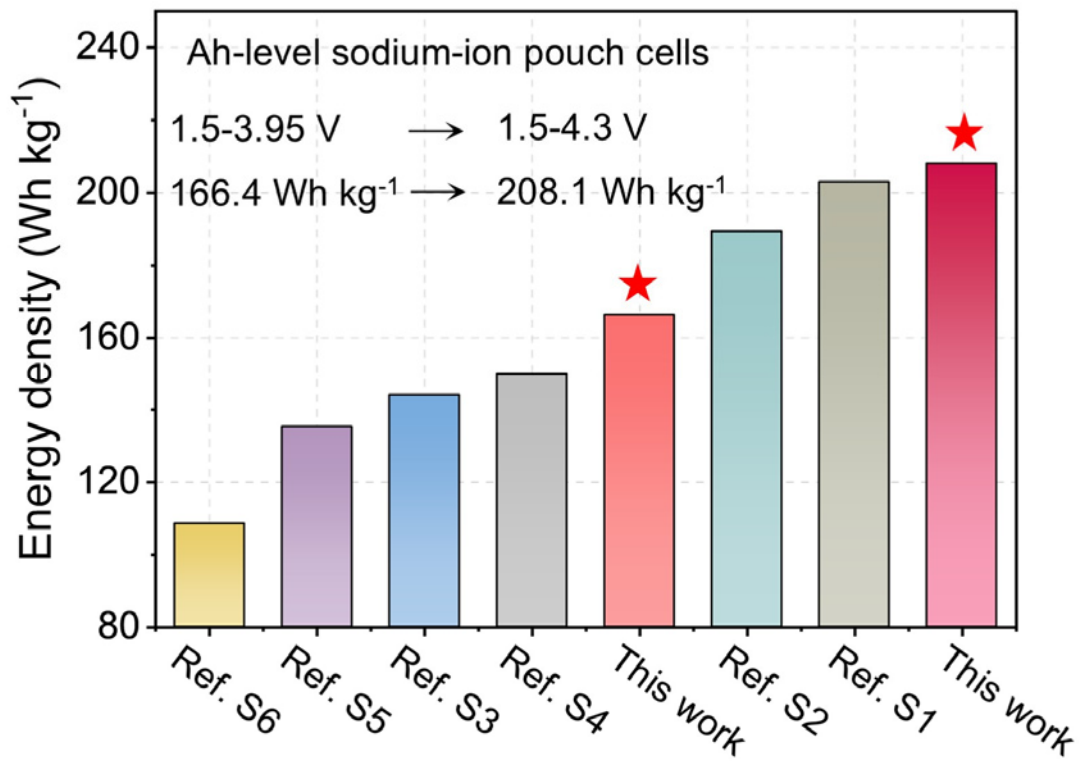

Figure 3. (f) Comparison of energy density in sodium-ion pouch cells.

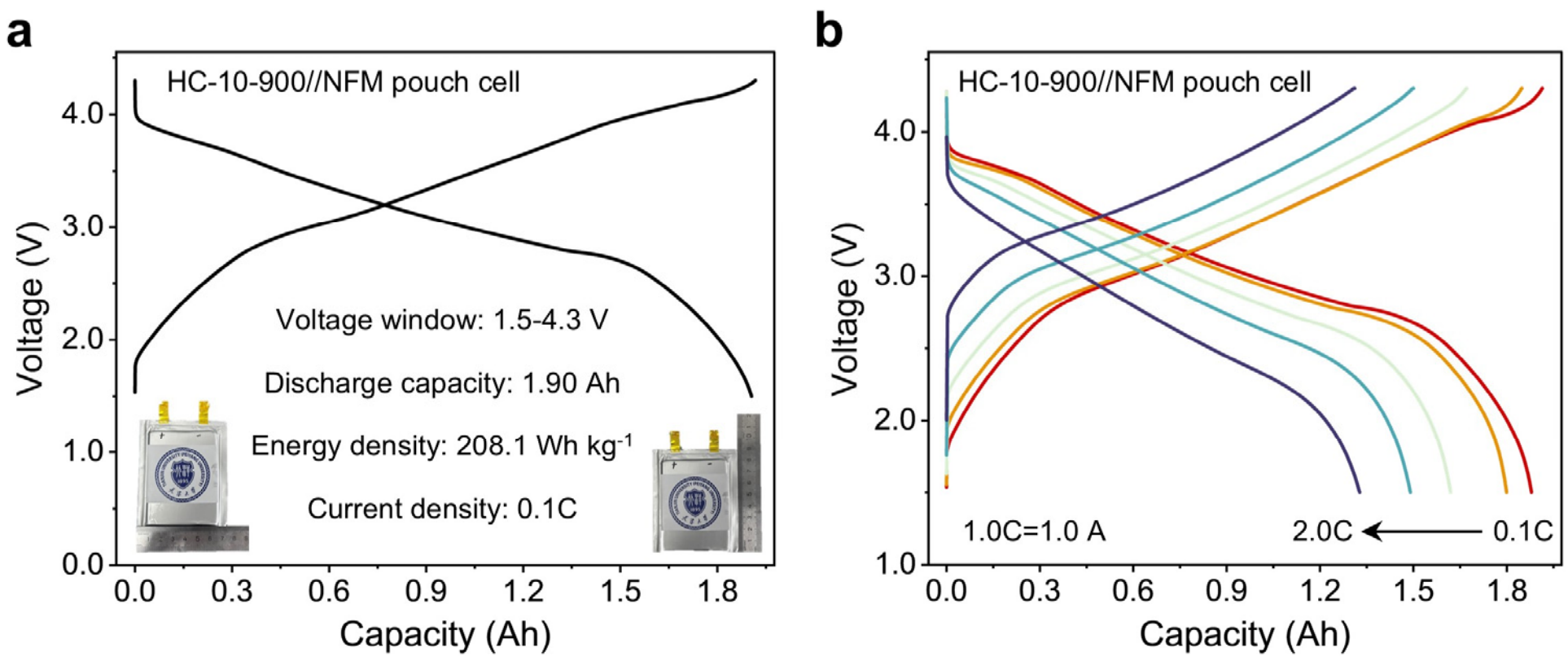

Supplementary Fig. 52. (a) Charging and discharging curves at 1.5-4.3 V of the sodium-ion pouch cell. (b) Rate performance at 1.5-4.3 V of the sodium-ion pouch cell.

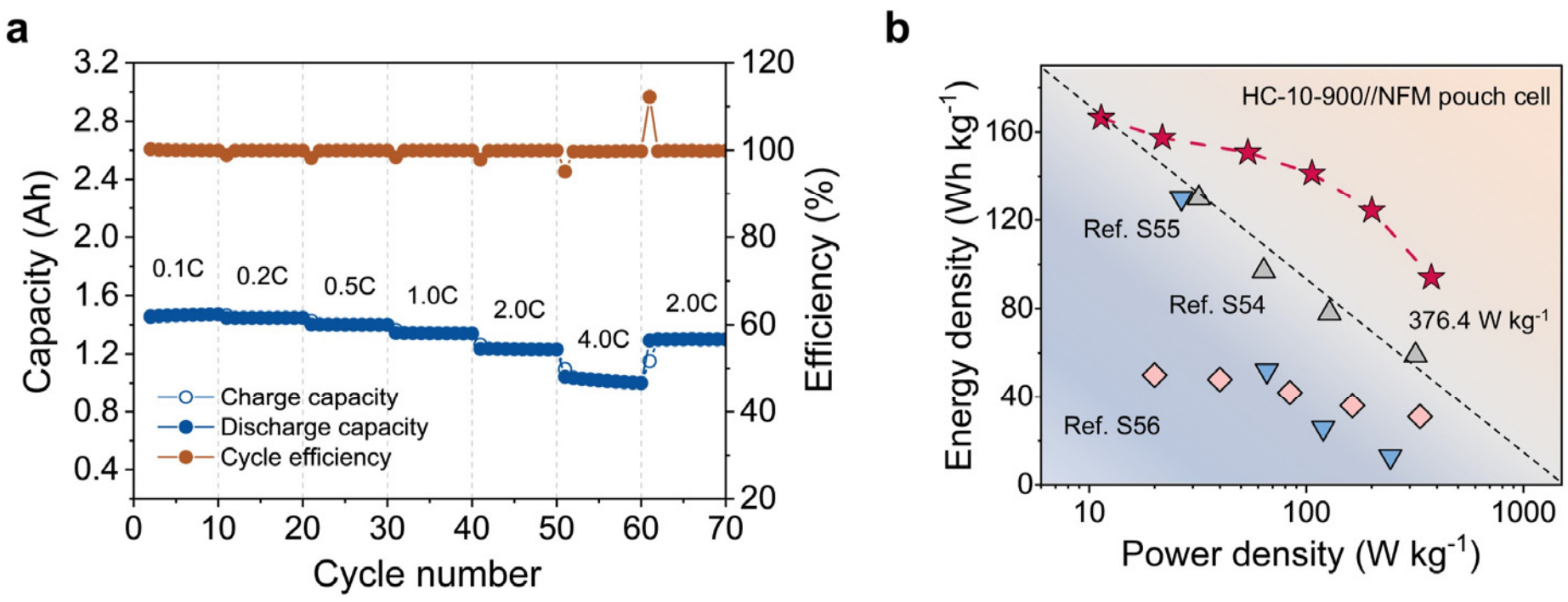

Supplementary Fig. 53. (a) Rate performance at 1.5-3.95 V of the sodium-ion pouch cell. (b) Comparison of power density in sodium-ion pouch cells[54-56].

Supplementary Table 8. The specifications of HC-10-900//NFM pouch cell.

Cell component	Specification	Parameters
Anode (HC-10-900)	Active material mass loading (mg cm^{-2}, each side)	7.0
	Material mass (g)	7.25
	Size (mm × mm)	77*56
	Active material ratio	0.90
	Nickel tabs	0.21
Electrolyte	$NaPF_6$ (mol L^{-1})	1.0
	EC: DEC ratio	1:1
	Quality (g)	3.0
Separator (PP)	Size (mm × mm)	80*58
	Mass (g)	0.74
Cathode (NFM)	Active material mass loading (mg cm^{-2}, each side)	12.5
	Material mass	12.15
	Size (mm × mm)	75*56
	Active material ratio	0.96
	Aluminum tabs	0.06
Current collector	Aluminum foil (g)	2.58
Package (Al-plastic film)	Size (mm × mm)	90*74
	Mass (g)	1.86
Pouch cell	Total weight (g)	27.85
Pouch cell (1.5-3.95 V)	Discharge capacity (Ah)	1.47
	Specific energy density (Wh kg^{-1})	166.4
Pouch cell (1.5-4.3 V)	Discharge capacity (Ah)	1.90
	Specific energy density (Wh kg^{-1})	208.1

Supplementary Table 9. Comparison of energy density in sodium-ion pouch cells.

**Cathode**	**Anode**	**Electrolyte**	**Capacity (Ah)**	**Energy density ($Wh\ kg^{-1}$)**	**Ref.**
$Na_{2/3}Ni_{1/3}Mn_{1/3}Ti_{1/3}O_2$	PFPI-13	1.0 M $NaPF_6$ in EC: DEC (1:1)	2.2	108.5	6
$NaNi_{1/3}Fe_{1/3}Mn_{1/3}O_2$	OP-HC	1.0 M $NaSO_3CF_3$ in DEGDME	1.239	150.0	4
$Na_3V_2(PO_4)_3$	PPHC900	1.0 M $NaClO_4$ in EC: DMC (1:1)	0.1	135.2	5
$Na_4Fe_3(PO_4)_2P_2O_7$	Metallic Sodium	1.0 M $NaPF_6$ in DEGDME	2.28	203.0	1
$Na_3V_2(PO_4)_3$	Metallic Sodium	NZSP–TLM	0.1145	220.0	69
$Na_3V_2(PO_4)_3$	HC (30 s)	1.0 M $NaPF_6$ in diglyme	--	189.3 (Based on active material mass)	2
$Na_3V_2(PO_4)_3$@C	CFG	1.0 M $NaClO_4$ in diglyme	--	144.0 (Based on active material mass)	3
$NaNi_{1/3}Fe_{1/3}Mn_{1/3}O_2$	TAC-1400	1.0 M $NaClO_4$ in EC: DEC (1:1)	0.4	254.3 (Based on active material mass)	70
$Na_3V_2(PO_4)_3$	G1500	1.0 M $NaPF_6$ in EC: DMC (1:1)	--	209.0 (Based on active material mass)	26
$Na_3V_2(PO_4)_3$	PAHC-1500	1.0 M $NaPF_6$ in diglyme	--	242.1 (Based on active material mass)	7
$NaNi_{1/3}Fe_{1/3}Mn_{1/3}O_2$	HC-10-900	1.0 M $NaPF_6$ in EC: DEC (1:1) (1.5-4.3 V)	1.90 at 0.1 A<br>1.33 at 2.0 A	208.1<br>129.7	This work
$NaNi_{1/3}Fe_{1/3}Mn_{1/3}O_2$	HC-10-900	1.0 M $NaPF_6$ in EC: DEC (1:1) (1.5-3.95 V)	1.47 at 0.1 A<br>1.23 at 2.0 A	166.4<br>124.3	This work

Comment #6. Maybe avoid using "an ideal microstructure". How do you know it is ideal?

Response: We sincerely appreciate the questions of reviewers regarding the ideal microstructure. We apologize for the exaggerated claims. Based on the range of structure parameters obtained

through ML, we mistakenly interpret the microstructure of HC that falls within these ranges as the ideal microstructure. We have carefully revised and reviewed to enhance the rigor and persuasiveness of the manuscript. Particularly, we have removed descriptions of ideal microstructures from the text to avoid misunderstanding.

Our revision:

1. Added detailed description.

p 3, line 42-43 (Abstract)

Herein, we overturn this long-standing constraint through a machine learning design paradigm and low-temperature thermal-force coupling carbonization that enables the microstructure in HC of both abundant sodium storage sites and rapid Na^+ diffusion.

p 3, line 48 (Abstract)

This work not only provides a double-field regulation strategy to prepare high-performance HC under low temperature, but also contributes a viable pathway toward sustainable SIBs.

p 4, line 22 (Introduction)

By a thermal-force coupling preparation strategy, we obtain the HC (HC-10-900) with a targeted microstructure, features by d_{002}=0.384 nm, I_D/I_G=1.33, and SSA=2.28 m^2 g^{-1} under a low temperature of 900°C with a pressure of 10 MPa.

p 5, line 98-99 (Results)

Notably, the thermal-force coupling strategy enables HC-10-900 carbonization at only 900°C to achieve a targeted microstructure, which enables reducing the carbonization temperature by 400°C compared to thermal-field-driven processing (1300°C, Fig. 2l).

p 20, line 410 (Discussion)

Low-temperature thermal-force coupling carbonization guided by ML-driven microstructure design was established to tailor promising HC microstructures, in which d_{002}=0.384 nm, I_D/I_G=1.33, and SSA=2.28 m^2 g^{-1} that perfectly belong to the targeted microstructure with a zone of moderate d_{002} (0.376-0.394 nm), moderate I_D/I_G (1.10-2.30), and low SSA (0.26-17.84 m^2 g^{-1}).

Comment #7. The introduction states only the initial stage of hard carbon research, without introducing recent progress and understanding. This raises many misinterpretations of their methods.

Response: We are very grateful to the reviewers for their thoughtful suggestions, which are essential

to improving the quality of the manuscript. We fully agree that the field of hard carbon research has made significant progress in recent years, particularly in terms of microstructural optimization, ultra-rapid carbonization techniques, and a deeper understanding of data-driven design. The introduction in the initial draft did not adequately reflect these advances, which could indeed lead readers to underestimate the innovativeness and relevance of our work. To address this issue, we have thoroughly rewritten and expanded the introduction in the revised draft.

Our revision:

1. Added detailed description and references.

p 4, line 67-94 (Introduction)

Recently, structure modification strategies combined with high-temperature carbonization (pre-oxidation, molecular cross-linking, and heteroatom doping) are widely used to construct Na^+ diffusion pathways and sodium storage sites, achieving high initial Coulombic efficiency (ICE) and reversible plateau capacity of HC[16-18]. Due to the inability of these modification strategies to significantly suppress high-temperature-driven structure graphitization, the Na^+ diffusion kinetics often fail to match the abundance of storage sodium sites, resulting in a bottleneck characterized by "abundant sites but slow diffusion"[19]. To optimize the carbonization process, emerging rapid carbonization technologies (thermal shock, Joule heating, and flash carbonization) are employed to construct a wide "highway" in HC for Na^+ diffusion[20-22]. These technologies prevent the compact stacking of the carbon layer structure through short-duration high-temperature treatment and ultra-rapid heating rates, thereby optimizing Na^+ diffusion kinetics. Notably, the HC obtained through rapid carbonization typically exhibits long-range ordered carbon layers and a large-volume closed-pore structure, which further increases the sodium storage capacity[23,24]. And the improved Na^+ diffusion rate remains insufficient due to excessive Na^+ packing and the long-range diffusion.

The complex evolution of various microstructure features (d_{002}, I_D/I_G, SSA, nanopores, and carbon layer) is inherent to the high-temperature carbonization, posing significant challenges to the optimization of the HC microstructure[25]. Furthermore, traditional research methods are unable to systematically elucidate the relationship between structure and performance due to the limited number of experiments and low efficiency. Although numerous studies have reached a certain consensus regarding the influence of structure on performance, the range of microstructure characteristics for high-performance HC remains undetermined[26]. Based on data from current

literature regarding processes, structures, and performance, machine learning (ML) uncovers complex nonlinear relationships behind vast variables, revealing the key microstructure influence rules within trade-offs to provide quantifiable optimal ranges[27,28]. Moreover, introducing an additional driving force during the low-temperature carbonization mitigates the impact of temperature on the structure, gradually promotes the ordered development of the structure, achieving a balance between sodium storage sites and diffusion kinetics. Importantly, transitioning from high-temperature carbonization to low-temperature carbonization represents a crucial technology challenge to achieve the optimal microstructure of HC, in response to the call for energy conservation and emission reduction.

p 29, line 636-664 (References)

[16] Liu, H. et al. Highways construction in amorphous carbon anode enables 10C fast-charging sodium-ion batteries. *Angew. Chem. Int. Ed.* **65**, e25941 (2026).

[17] Qiu, C. et al. One-step construction of closed pores enabling high plateau capacity hard carbon anodes for sodium-ion batteries: Closed-pore formation and energy storage mechanisms. *ACS Nano* **18**, 11941-11954 (2024).

[18] Wang, F. et al. Revitalizing sodium-ion batteries via controllable microstructures and advanced electrolytes for hard carbon. *eScience* **4**, 100181 (2024).

[19] Xu, Y-J. et al. Cellulose-grafting boosted pyrolysis nucleation: Achieving low-temperature construction of hard carbon anodes with long low-voltage plateau and ultrafast Na storage kinetics. *Energy Storage Mater.* **75**, 104031 (2025).

[20] Cui, J. et al. Data-driven intelligent carbonization unifies diverse biomass into high-performance hard carbon negative electrodes. *Nat Commun.* (2026).

[21] Qiu, Y. et al. Rapid closed pore regulation of biomass-derived hard carbons based on flash joule heating for enhanced sodium ion storage. *Adv. Funct. Mater.* **35**, 2423559 (2025).

[22] Wang, H. et al. Thermal shock reducing amorphous carbon ratio in hard carbon for improved rate capability of sodium ion storage. *Carbon* **229**, 119528 (2024).

[23] Song, Z. et al. Joule heating for structure reconstruction of hard carbon with superior sodium ion storage performance. *Chem. Eng. J.* **496**, 154103 (2024).

[24] Luo, F. et al. Rapid flash heating of hard carbon: Enhanced sodium storage via hierarchical graphitization. *Energy Storage Mater*. **86**, 104929 (2026).

[25] Wang, A. et al. Lignin derived hard carbon for sodium ion batteries: Recent advances and future perspectives. *Prog. Mater. Sci.* **152**, 101452 (2025).

[26] Wen, Q. et al. Carbon engineering for sodium batteries: Multi-role architectures bridging material design and hybrid system innovation. *Chem. Soc. Rev.* **54**, 9317-9369 (2025).

[27] Owusu, S. Y., Doulah, A. B. M. S. U., Edlabadkar, V. A., George, K. J. & Leventis, C. S. The effect of nanomorphology as quantified via the K-index on the drug delivery properties of isocyanate-derived aerogels. *ACS Appl. Polym. Mater.* **6**, 10655-10668 (2024).

[28] Owusu, S. Y., Boateng, M. A. & Soni, R. U. Machine learning predictions of drug release from isocyanate-derived aerogels. *J. Mater. Chem. B* **13**, 6233-6245 (2025).

Comment #8. The authors state that microstructure features are coupled in the traditional thermal field, but independent in theirs. Such independent control is not supported by any evidence. Note: a different combination of feature parameters does not indicate independent control.

Response: We sincerely appreciate the insightful analysis of reviewers of both the coupling and independence of microstructure features. We strongly agree that merely having different combinations of microstructure parameters does not equate to independent control of those parameters. We will correct this error in the revised manuscript to improve rigor.

Both conventional thermal field carbonization and thermal-force coupling carbonization result in coupled changes in microstructure characteristics, differing only in the degree of these changes. Conventional thermal-field carbonization primarily relies on high carbonization temperatures to control the microstructure of HC. However, high carbonization temperatures deliver a large amount of energy to the material very rapidly, making it difficult to control the coupling rates between various structure features. This results in a high degree of graphitization, with rapid decreases in defect, interlayer spacing, and SSA. Due to the rapid changes in microstructure features, it is difficult to achieve both a high sodium storage sites and fast diffusion kinetics. In contrast, low-temperature thermal-force coupling carbonization offers a more moderate reaction rate due to the low carbonization temperature. Furthermore, the force field induces an increase in the efficiency of the low-temperature thermal field through its gradual action. Specifically, under conditions of low-temperature carbonization combined with additional pressure, the stacking and growth of the carbon layer are gradually promoted, surface defects are transformed into bulk defects, the SSA is rapidly

reduced, and a closed-pore structure gradually forms. This achieves structure characteristics that cannot be balanced through carbonization in a single thermal field alone, thereby simultaneously fulfilling the requirements for high sodium storage sites and rapid diffusion kinetics.

Therefore, the strategy of low-temperature thermal-force coupling breaks the strong coupling between temperature and structure evolution in conventional thermal fields. External pressure provides an additional control dimension that can compensate for the adverse effects at low temperatures.

Our revision:

1. Added detailed description.

p 5, line 80-82 (Introduction)

The complex evolution of various microstructure features (d_{002}, I_D/I_G, SSA, nanopores, and carbon layer) is inherent to the high-temperature carbonization, posing significant challenges to the optimization of the HC microstructure[25].

p 5, line 88-91 (Introduction)

Moreover, introducing an additional driving force during the low-temperature carbonization mitigates the impact of temperature on the structure, gradually promotes the ordered development of the structure, achieving a balance between sodium storage sites and diffusion kinetics.

p 9, line 174-177 (Results)

In contrast, thermal-force coupling carbonization not only compacts the material into a dense block, significantly reducing the SSA, but also promotes the slow growth of the carbon structure, keeping the I_D/I_G and d_{002} within reasonable ranges (Supplementary Fig. 12, 13).

Supplementary Note 8 (Supplementary Information)

The increase in the R factor with rising pressure demonstrates that additional pressure promotes the development of structure graphitization due to the ordered and compact stacking of carbon layers. This has a similar promoting effect to raising the carbonization temperature, yet the pressure-induced effect is gradual and easier to control.

Supplementary Note 10 (Supplementary Information)

Among these, ordered carbon layers and appropriate defects of HC-10-900 form a Na^+ diffusion network, which improves the diffusion kinetics of Na^+. Compared to force fields, further increasing the carbonization temperature also promotes graphitization development of HC, yet this process is

difficult to control. HC-0-1300 exhibits a low I_D/I_G, and its low-defect and highly ordered structure increases the resistance to Na^+ diffusion.

Comment #9. How do the authors perform MD with thermal and pressure fields to guide experiments?

Response: We are very grateful to the reviewers for raising critical questions regarding the molecular dynamics simulations of thermal-force coupling carbonization. We use molecular dynamics simulations to reproduce the evolution of carbon structures under thermal and force fields at the atomic scale to reveal the thermal-force coupling mechanism of carbonization, rather than to provide direct guidance for experiments (**Figure R14**).

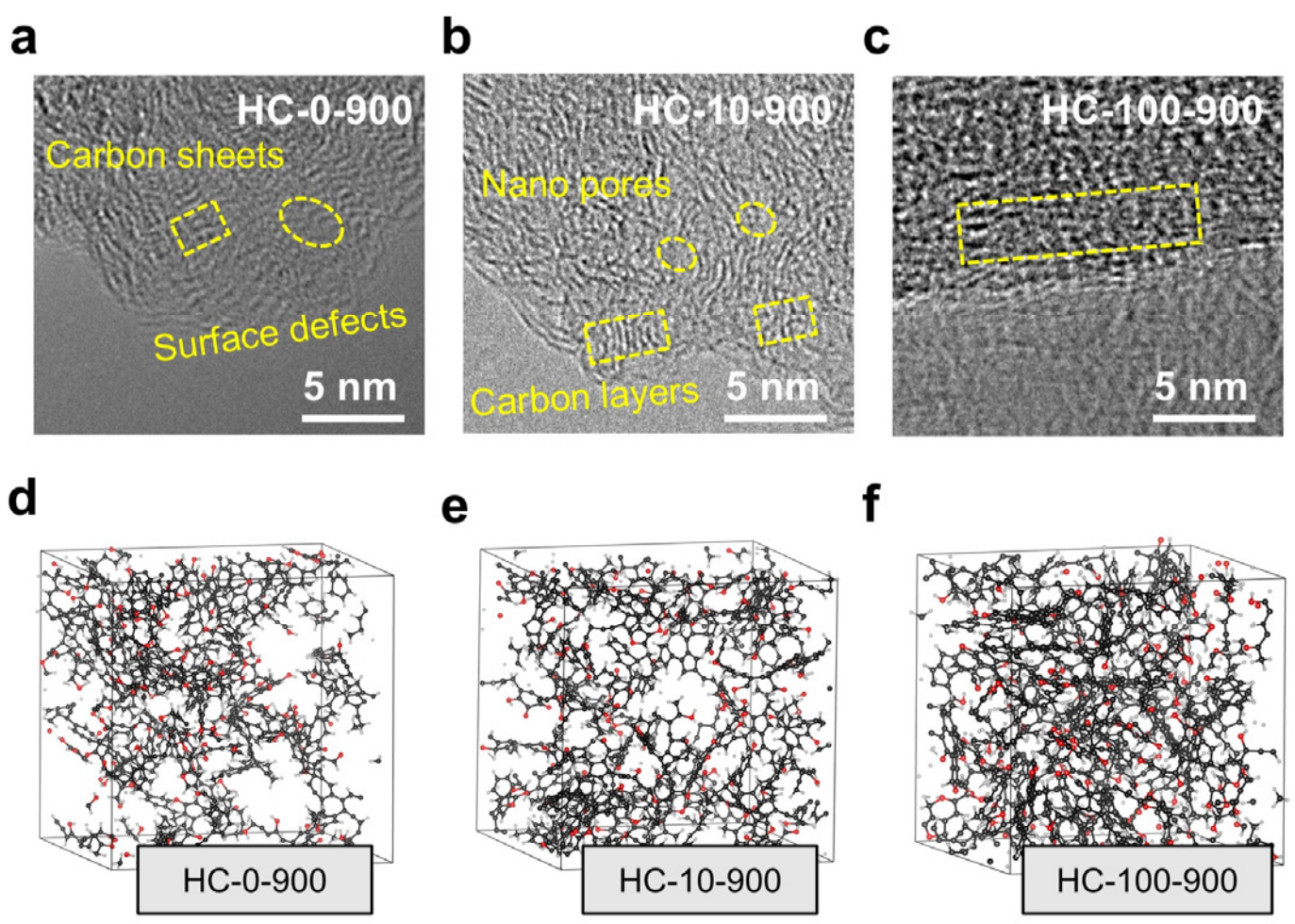

Figure R14. (a-c) HRTEM images, and (d-f) Molecular dynamics simulations of HC-0-900, HC-10-900, and HC-100-900.

The overall experiment was conducted by formulating the experimental design and related parameters based on the structure parameters determined through ML. In molecular dynamics simulations, parameters are determined based on experimental conditions, and the results are intended to provide further theoretical support for our experimental findings. We have simply simulated the thermal decomposition process of glucose to verify the rationality of the relevant findings. The disordered structure of HC is highly dependent on synthesis conditions, particularly the heat treatment temperature, which makes it difficult to establish a universal structure model. Although a simulation temperature is set when using molecular dynamics modeling, it is difficult to correlate it with experimental conditions. All-atom molecular dynamics simulations are typically

conducted on the nanosecond timescale, which is several orders of magnitude smaller than experimental annealing times. The vast difference in time scales complicates comparisons between simulations and experiments (***Carbon 2017***, 119, 1-9). Therefore, the molecular dynamics simulation results presented in this paper are primarily intended to qualitatively validate the thermal-force coupling carbonization mechanism, rather than to directly guide the precise setting of experimental parameters.

Our revision:

1. Added detailed description and references.

p 9, line 190-193 (Results)

Molecular dynamics (MD) simulations confirm that under the thermal-force coupling effect, the short carbon fragments accelerate cross-linking to promote the epitaxial growth of the carbon layer and form a closed-pore structure between the curved pseudo-graphite microdomains (Figs. 2g-i and Supplementary Figs. 17, 18).

p 10, line 193-194 (Results)

Meanwhile, variations in the ordered structure of the unit cell exhibit consistent trends with characterization results of HRTEM (Fig. 2j).

p 24, line 521-527 (Methodology)

The disordered microstructure of HC exhibits strong dependence on synthesis parameters, particularly annealing temperature, which significantly hinders the development of a structure model. Although MD simulations allow for the specification of specific thermodynamic temperatures, it is difficult to establish a direct mapping between the simulated system and the actual experimental environment. The time scale of all-atom MD simulations is typically limited to the nanosecond range, whereas experimental annealing processes often last for several hours. This vast gap of several orders of magnitude between the two time scales makes comparisons between simulations and experiments highly complex[64].

p 34, line 744-745 (References)

[64] Li, J. Peng, C. Wang, J. Li, J. & Zhang, H. Towards an atomistic understanding of hard carbon electrode materials and sodium behaviors. *Diam. Relat. Mater.* **129,** 109355 (2022).

Comment #10. Figure errors and grammar issues, such as "HC-0-90" should be HC-0-900, etc.

Response: We sincerely thank the reviewer for this careful observation. We will correct this error in the revised manuscript and perform a thorough grammar and spelling check throughout the text to ensure overall linguistic accuracy.

Our revision:

1. Grammar or spelling correction.

Supplementary Information

Supplementary Fig. 20. (a) X-ray diffraction (XRD) pattern and (b) L_a, L_c of HC-0-900, HC-5-900, HC-10-900, HC-50-900, HC-100-900, and HC-0-1300.

Supplementary Fig. 27. (a) The volume distribution of closed pores in HC-0-9000, HC-10-900, and HC-100-900. (b) The open pore volume and closed pore volume of HC-0-900, HC-5-900, HC-10-900, HC-50-900, and HC-100-900. (c) The BET SSA and SAXS SSA of HC-0-9000, HC-10-900, and HC-100-900.

2. Modified figures

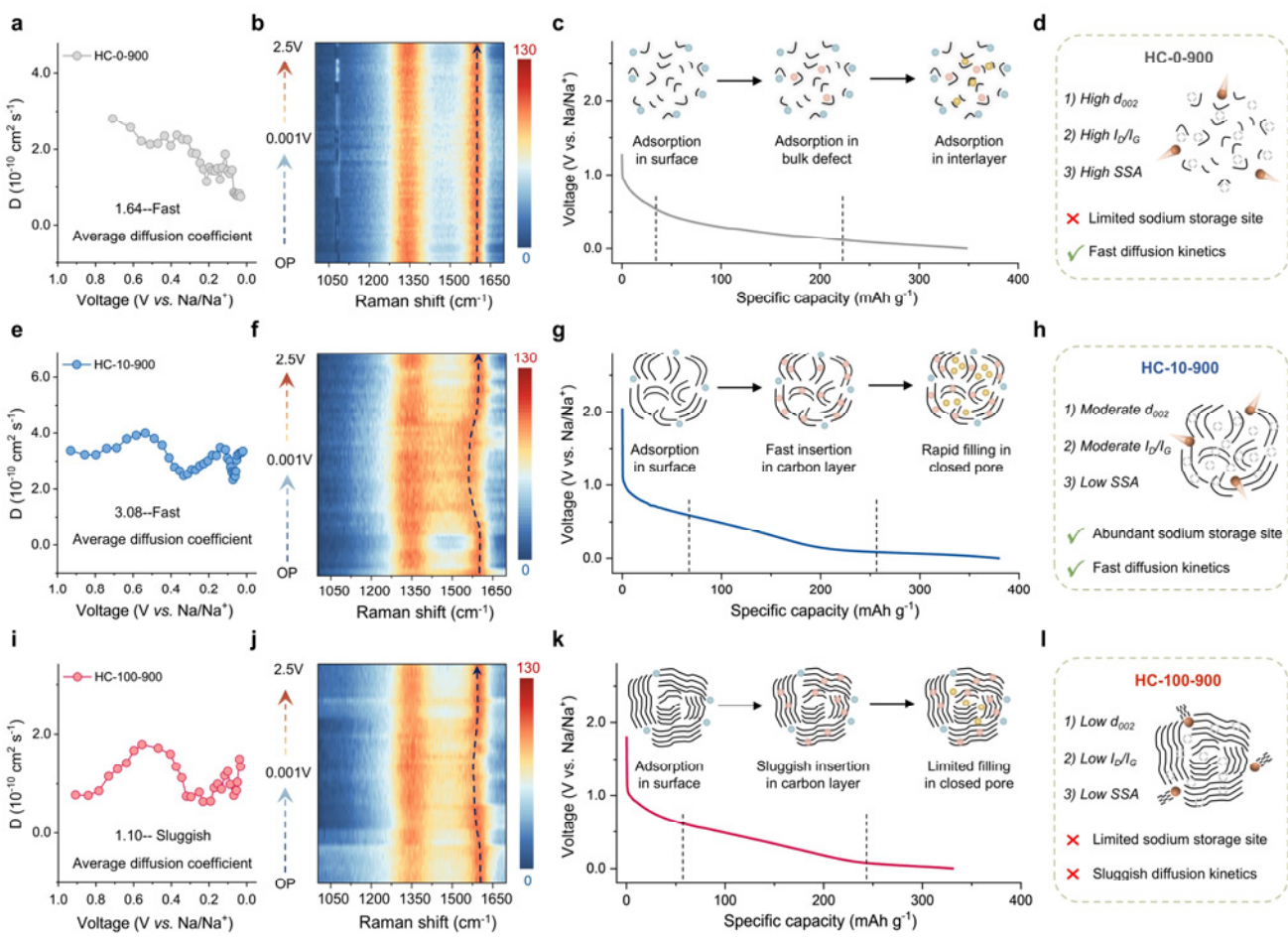

Figure 4. The diffusion coefficient, in-situ Raman, sodium storage mechanism, and structure characteristics of (a-d) HC-0-900, (e-h) HC-10-900, and (i-l) HC-100-900.

We sincerely thank you for your constructive comments and careful evaluation of our work. We hope that the substantial revisions and new analyses provided in this response satisfactorily address all of your concerns.

Point-by-Point Response

(Black type: Reviewer's comments; Blue type: Our response; Red type: Our revision)

Reviewer #1 (Remarks to the Author):

The revised ML part has been substantially improved, and I appreciate the authors' efforts in this regard. The additional model comparison, validation have made this part much more transparent and complete. However, I remain concerned about the framing of the economic and environmental assessment. The authors have clarified that no standard LCIA method and no discounted cash-flow model were used; the analysis is basically a simplified laboratory-scale bottom-up cost and CO_2 accounting exercise. Therefore, I do not think it should be referred to as a formal LCA or TEA. Since this section is not the core contribution of the manuscript, I do not suggest to redo a complete LCA/TEA, but I strongly recommend that the authors should revise the terminology and tone down the related claims. Terms like CAPEX and OPEX should also be used with caution, because the current CAPEX is just an allocated equipment depreciation cost, while the OPEX-like term appears to be a simplified labor/overhead proxy rather than a complete operating expenditure. I would recommend authors to use a much more conservative framing style, such as "laboratory-scale cost and CO_2 emission assessment", this would be more appropriate.

Response: We are sincerely grateful to the reviewers for their recognition of the improvements made to the ML section, and we also appreciate your insightful and forward-thinking comments on the economic and environmental assessment section. The economic and environmental assessments presented in this study are preliminary and were conducted only on a laboratory scale. They are intended to demonstrate the potential advantages of low-temperature thermal-force coupling strategies in terms of energy consumption and cost reduction. Comprehensive life cycle assessments (LCA) and techno-economic analyses (TEA) require detailed process simulations, supply chain data, and industrial-scale engineering parameters. Future work will focus on scaling up the process to validate these preliminary findings.

Following your advice, we revised the terminology and toned down the wording in the relevant sections of the text to eliminate potential misinterpretation and ensure academic rigor. The CAPEX and OPEX have been revised as equipment depreciation costs and labor costs. The economic and environmental analysis has been changed to a laboratory-scale cost and CO_2 emission assessment.

We firmly believe that these revisions will render the discussion in the economic and environmental assessment section of the paper more rigorous and objective, in line with academic standards, and accurately reflect the actual contribution of this study. We would like to thank you once again for your valuable suggestions, which have greatly enhanced the quality of the paper.

Our revision:

1. Revised detailed description.

p 18, line 371 (Results)

Laboratory-scale cost and CO_2 emission assessment

p 18, line 372-373 (Results)

To compare the LTC, high-temperature thermal field carbonization (HTC), and LTFC, we conducted a laboratory-scale cost and CO_2 emission assessment.

p 19, line 396-397 (Results)

We evaluated the laboratory-scale cost and environmental performance of HC materials preparation by comparing it with LTC, HTC, and LTFC methods[58,59].

p 19, line 401-403 (Results)

The specific calculation details of the laboratory-scale cost are presented in Tables S9 and S10. In the comprehensive evaluation of three different pathways, the primary private costs of HC production include feedstock, equipment depreciation, labor and overhead, and electricity expenses.

p 25, line 544-545 (Methods)

The production cost of HC is decomposed into four major components: feedstock, equipment depreciation, labor and overhead, and electricity consumption.

p 26, line 551-554 (Methods)

Equipment depreciation cost (C_{eqpt}($/100g)) arises from the pre-carbonization and pyrolysis stages, which is a function of the process-specific processing time (h) and the hourly equipment depreciation rate c_{eqpt}($/hr):

$$C_{eqpt} = c_{eqpt} * \left(h_{pre} + h_{pyro} * B\right),$$

p 26, line 562-564 (Methods)

Labor and overhead costs (C_{labor}($/100g)) are assumed to scale proportionally with total equipment depreciation cost, using a fixed ratio (λ):

$$C_{labor} = \lambda * C_{eqpt}.$$

2. Revised tables.

Supplementary Table 9. Parameter assumptions for the laboratory-scale cost and CO_2 emission assessment.

Process	Variables	Unit	Data source	HC-10-900	HC-0-1300	HC-0-900
Full battery	Anode mass	mg	Experiment	3.23	4.88	3.57
Full battery	Cathode mass	mg	Experiment	2.99	4.4	9.28
Full battery	C1_full battery	Wh	Experiment	0.002174	0.002585	0.001586
Full battery	C80_capacity	Wh	Experiment	0.001955	0.001749	0.001127
Full battery	R80	--	Experiment	0.8906	0.6517	0.7122
Full battery	N/P	--	Experiment	1.08	1.11	1.06
Half battery	C1_half_battery	Wh	Experiment	0.000159	0.000182	0.000107
Half battery	C40_half_battery	Wh	Experiment	0.000158	0.000155	0.000088
Half battery	R40	--	Experiment	0.9937	0.8517	0.8224
Half battery	Anode mass	mg	Experiment	1.56	2.06	0.91
Pre-carbonization	Pre-carbonization Energy	kWh/1kg feedstock	Experiment	1.72	1.72	1.72
Pre-carbonization	Pre-carbonization output ratio	--	Experiment	0.528	0.528	0.528
Pre-carbonization	Mass fraction of C in pre-treated feed	%	Experiment	67.5	67.5	67.5
Pre-carbonization	Pre-carbonization CO_2 emissions	g/100g feed	Experiment	16.14	16.14	16.14
Pre-carbonization	Hours run	--	Experiment	2	2	2
Pyrolysis	HC mass per 8g pre-treated feed	g	Experiment	3.472	1.584	2.872
Pyrolysis	Mass fraction of C in HC	%	Experiment	93.5	82.50	82.75
Pyrolysis	Pyrolysis CO_2 emission	g/8g feed	Experiment	7.87	14.98	11.06
Pyrolysis	Pyrolysis Energy	kWh/8g feedstock	Experiment	15.4	30.6	10.6
Pyrolysis	Hard Carbon output ratio	--	Experiment	0.0787	0.1498	0.1106
Pyrolysis	Hours run	--	Experiment	5	8	5
	Electricity price	$/kWh	Assumption	0.088	0.088	0.088
	Carbon intensity	$kgCO_2/kWh$	Assumption	0.5	0.5	0.5
	SCC_China	$/kgCO_2$	Assumption	0.015	0.015	0.015

SCC_Global	$/kg$CO_2$	Assumption	0.2	0.2	0.2
Hourly equipment depreciation cost	$/hr	Assumption	0.016	0.033	0.011
Labor & overhead (% of equipment depreciation cost)	--	Assumption	0.05	0.05	0.05
Feedstock price	$/kg	Assumption	4	4	4

Supplementary Table 11. Cost of preparing HC using 100g feedstock.

-	Composition	HC-0-900	HC-10-900	HC-0-1300
Precursor	$C_6H_{12}O_6$ (g)	100	100	100
Private costs	Feedstock price ($)	0.4	0.4	0.4
	Equipment depreciation ($)	0.40	0.59	1.85
	Labor and overhead ($)	0.02	0.03	0.09
	Electricity ($)	6.68	9.64	19.00
Social costs	CO_2 emissions of electricity ($)	7.59	10.95	21.59
	CO_2 emissions of materials ($)	0.018	0.014	0.023
Total cost	Private and social costs ($)	15.11	21.62	42.95
HC	Yield (g)	18.96	22.92	10.45

Supplementary Table 12. The cost per kWh required for the full battery systems assembled in HC.

-	Composition	HC-0-900	HC-10-900	HC-0-1300
Private costs	Feedstock price ($/kg HC)	21.10	17.46	38.26
	Equipment depreciation ($/kg HC)	21.22	25.86	176.79
	Labor and overhead ($/kg HC)	1.06	1.29	8.84
	Electricity ($/kg HC)	352.46	420.58	1817.51
Social costs	CO_2 emissions ($/kg HC)	401.46	478.53	2067.55
Total cost	Private and social costs ($/kg HC)	797.31	943.72	4108.95
Full battery	Total energy (kWh/kg HC)	21.47	102.50	22.30
Cost per kWh	Equipment depreciation ($/kWh)	0.99	0.25	7.93
	Electricity ($/kWh)	16.42	4.10	81.50
	CO_2 emissions ($/kWh)	18.70	4.67	92.72
	Total cost ($/kWh)	37.14	9.21	184.26

We sincerely thank you for your constructive comments and careful evaluation of our work. We hope that the substantial revisions and new analyses provided in this response satisfactorily address all of your concerns.

Point-by-Point Response

(Black type: Reviewer's comments; Blue type: Our response; Red type: Our revision)

<u>Reviewer #4 (Remarks to the Author):</u>

The authors have struggled to address the previous comments. This manuscript attempts to present a new paradigm for hard carbon anodes by combining Machine Learning (ML) with a "Thermal-Force Coupling" carbonization. While the electrochemical performance reported is high, the scientific novelty and contribution can be low. It becomes evident that the ML integration is redundant and the sustainability claims are largely unsubstantiated. The core contribution of this work is the synthesis (thermal + force field) of a hard carbon material with improved electrochemical performance. This advancement may not meet the high standards for publication in Nature Communications.

Response: We sincerely thank the reviewer for their comprehensive summary of our work, as well as the constructive comments you have provided on our manuscript. We have carefully considered all your suggested changes regarding machine learning (ML), thermal-force coupling carbonization, and feasibility assessment, and have incorporated them into the revised manuscript.

In terms of ML, we highlighted the significant differences between conclusions derived from ML and those obtained through traditional empirical rules, clarified the purpose of ML in this research, which is not to predict high-performance hard carbon (HC) materials, and enhanced the correlation between ML and thermal-force coupling preparation. **In terms of thermal-force coupling carbonization**, from the perspectives of kinetics and thermodynamics, molecular dynamics simulations and structure characterization have comprehensively revealed that applying pressure reduces the activation energy for carbon structure rearrangement at low carbonization temperatures, thereby enhancing the thermal-force coupling mechanism of HC microstructure evolution. **In terms of feasibility assessment**, we reduced the numerical precision of energy consumption and carbon emissions, returning to scientific rationality.

We believe that these additions have enhanced the overall scientific rigor and novelty of the manuscript. Thank you once again for your valuable suggestions. Below, we discuss the specific suggestions you raised and provide point-by-point explanations.

Comment #1. The authors' response to previous concerns regarding the ML component remains unsatisfactory. While they have provided improved R2 values and model comparisons, they have failed to address the teleological flaw in their research design: this ML provides very limited predictive insight and appears largely disconnected from the experimental strategy. The "Target Zone" identified is already a well-established consensus in the field. Using ML to confirm what is already known adds technical complexity without providing new scientific insight. In addition, in its current form, the ML acts as a "black box" decorator rather than a functional discovery tool.

Response: We sincerely appreciate the reviewers for their insightful comments regarding the limitations of the ML components in this study in terms of defining scientific value and predicting the depth of insights. We fully agree that if ML is used solely to confirm established consensus, it loses its scientific value. In the revised manuscript, the ML component is not intended to replicate established consensus and predict high-performance HC, but rather to analyze the nonlinear relationship between complex microstructural parameters and electrochemical performance. We utilized interpretability tools (SHAP value, feature importance analysis) to identify key structural parameters in the black box of ML. This further guides the low-temperature thermal-force coupling preparation of high-performance HC.

Traditional empirical methods are typically based on a limited number of experiments and univariate control logic, evaluating the effects of d_{002}, I_D/I_G, and SSA in isolation, which often overlooks the interactions between these parameters (***Energy Environ. Sci., 2025,*** 18, 4312-4323). High I_D/I_G may compensate for the kinetic lag caused by moderate d_{002} by providing additional active sites, and such synergistic and antagonistic effects cannot be captured by traditional empirical methods. Mai et al. used first-principles calculations to determine the energy required for Na^+ to insert into carbon layers with different d_{002} (0.34 nm, 0.37 nm, and 0.40 nm) (***Small 2022***, 18, 2105303). The results indicate that as the interlayer spacing increases, Na^+ can more easily insert into the carbon layers. Furthermore, Bai et al. also calculated the energy barrier of Na^+ insertion into HC with d_{002} of 0.34, 0.36, and 0.38 nm using DFT. When the d_{002} is 0.34 nm (similar to that of graphite), the energy barrier is 0.49 eV, making Na^+ insertion impossible (***Energy Environ. Sci., 2024***, 17, 1387-1396). The energy barrier is below 0.20 eV after increasing the d_{002} to over 0.38 nm, indicating that Na^+ can easily intercalate into the interlayers (**Figure R1**). Theoretical calculations of a single variable indicate that the wider the d_{002}, the shorter the distance required for the diffusion

of Na^+ to overcome the maximum energy barrier. However, actually, a larger d_{002} does not necessarily promote reversible sodium storage, a larger d_{002} leads to irreversible adsorption on the surface of the carbon layer.

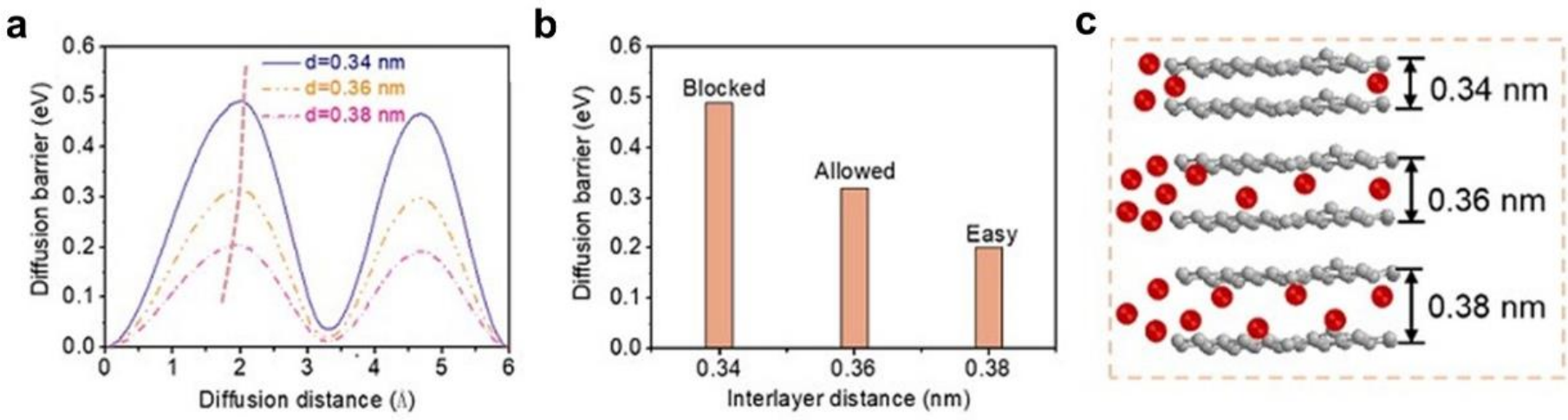

Figure R1. (a) and (b) The diffusion rate of sodium ions between different diffusion regions. (c) The calculation models for the diffusion behavior of Na^+ into HC.

Furthermore, traditional consensus mechanisms often lack quantitative boundaries and only provide vague guidance, making it impossible to determine critical thresholds (***Energy Environ. Sci., 2024,*** 17, 2856-2863). This results in high trial-and-error costs and performance fluctuations in experimental design. **Moreover, traditional empirical methods struggle to address multiple performance trade-offs simultaneously.** Feng et al. investigated the contributions of the carbon layer number, d_{002}, and L_a and L_c structural features to capacity, concluding that HC with 4 layers, a L_a value of 3.3 nm, and a L_c value of 4.4 nm offers higher capacity (***Energy Environ. Sci., 2024***, 17, 1387-1396). Wang et al. evaluated the relationships between the electrochemical performance and microstructural features (i.e., d_{002}, A_D/A_G, L_a, L_c, open pore volumes, closed pore volumes, and pseudo-graphite ratios) of the obtained samples (***Adv. Energy Mater., 2025***, 15, 2405294). For the initial capacity loss (irreversible capacities) and ICEs, the irreversible capacity increases with the increase in open pore volume and the A_D/A_G value, while the ICE exhibits an opposite trend (**Figure R2**). For the high voltage sloping region, the slope capacity displays a similar variation trend as the number of open nanopores and defects. For the low voltage plateau region, the plateau capacity is positively correlated with the number of closed nanopores and the ratio of pseudo-graphite. Therefore, the author proposes that the samples with higher reversible capacity and plateau capacity present multiple microstructural merits in terms of a high ratio of pseudo-graphite and closed pore volume, large d_{002}, as well as moderate microcrystalline size (L_a and L_c) and defect amount (A_D/A_G). However, this consensus is relatively vague and lacks substantial data to support it.

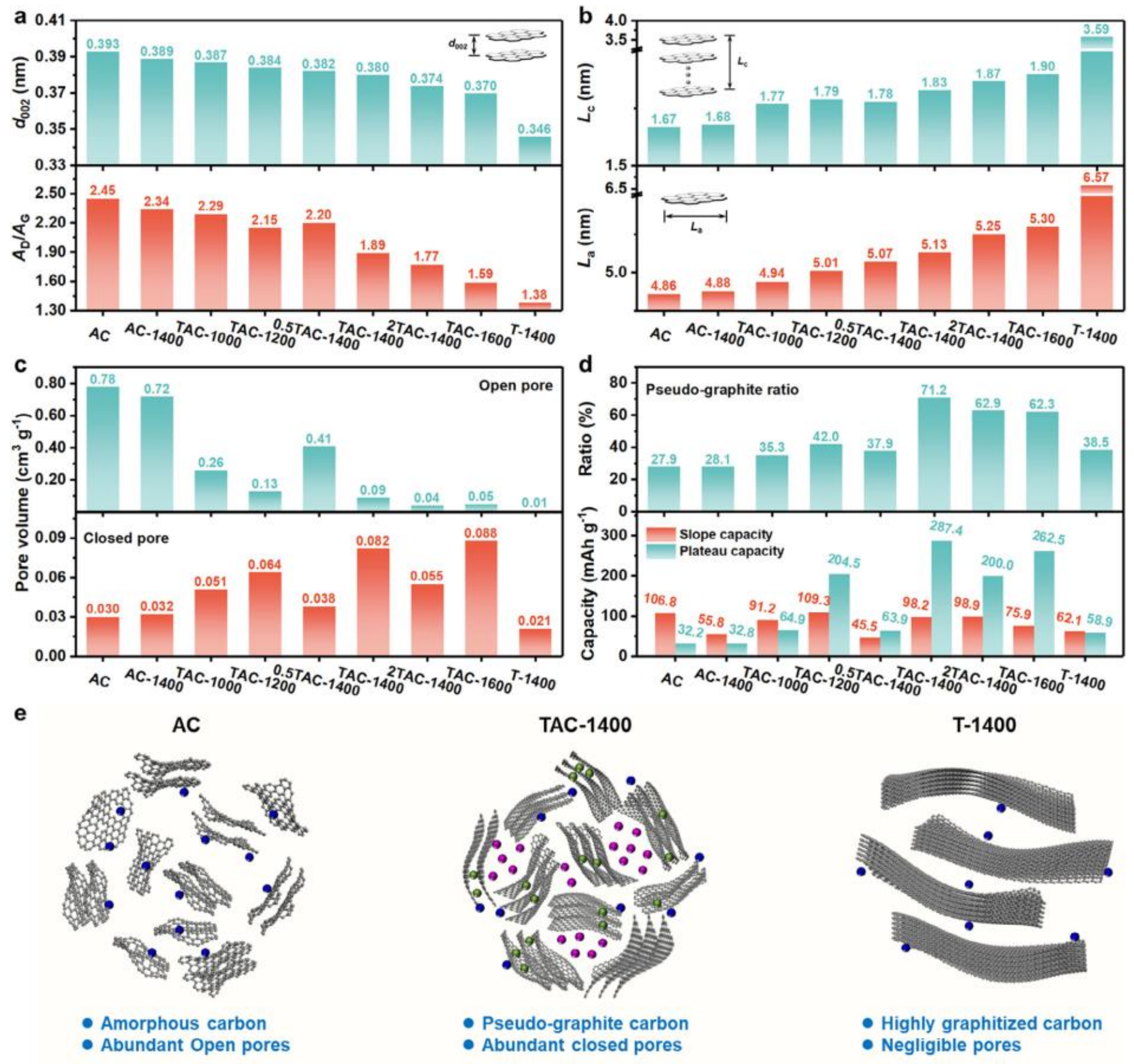

Figure R2. Structure-performance relationships. (a) Comparison of the values of d_{002} and A_D/A_G of different samples. (b) Comparison of the values of L_c and L_a of different samples. (c) Comparison of open/closed pore volumes of different samples. (d) Comparison of pseudo-graphite ratios and capacities of different samples. (e) Schematic illustration of the sodium storage behaviors in AC, TAC-1400, and T-1400.

The rapid accumulation of experimental data, computational simulations, and literature reports provides a valuable foundation for applying ML techniques to uncover hidden correlations within complex datasets. By identifying potential structure–performance relationships, ML models can generate data-driven hypotheses and guide experimental design toward more targeted directions (***Nat. Commun., 2024,*** 15, 344). Such an approach shifts part of the research paradigm from empirical trial-and-error toward hypothesis-driven validation. The ML models can efficiently screen large synthesis parameter spaces, establish quantitative relationships between structural descriptors and electrochemical performance, and assist in identifying key structural features that govern sodium storage behavior. These predictions can subsequently be verified through focused experiments, thereby accelerating materials discovery and improving research efficiency (**Figure R3**).

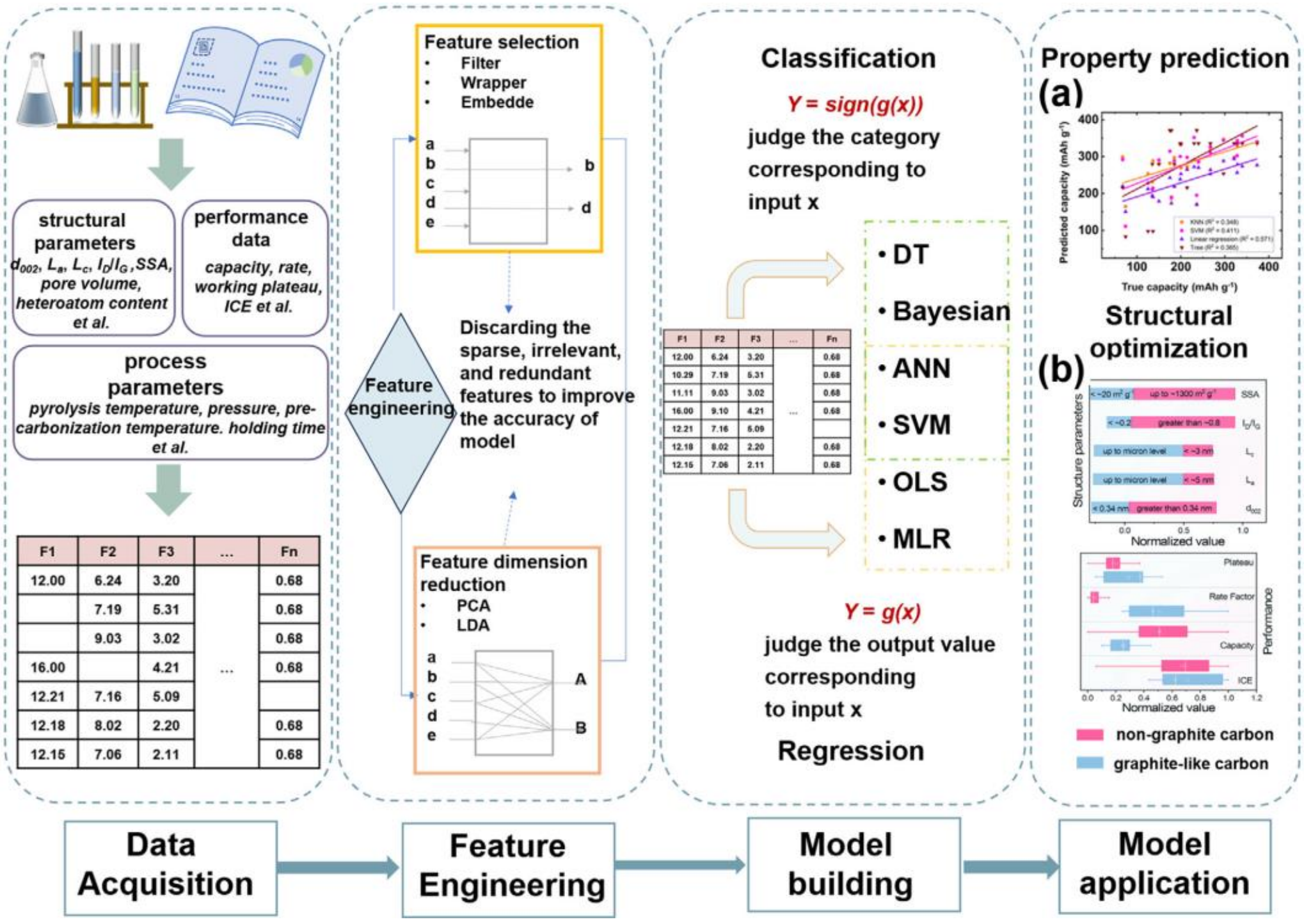

Figure R3. Workflow of ML (a) Prediction of the reversible capacities of some samples whose capacities were assumed to be unknown and modeled for authentication purposes. (b) Summary and comparison of the structure and properties of different types of carbon materials.

In optimization studies regarding microstructure, defects, and pores, and heteroatom doping, the value of ML lies in quantifying the complex interactions among multiple structural parameters. Liu et al. generated approximately 20000 sets of virtual structural data, corresponding to HC within defined parameter ranges (e.g., L_a of 2.5 – 5 nm, L_c of 1.5 – 2.5 nm, ***J. Mater. Chem. A 2022***, 10, 8031–8046). This approach enabled accurate performance prediction and guided inverse design based on structure (**Figure R4a-c**). Zhang et al. trained models based on structural features, including heteroatom content, graphite microcrystal size, pore structure parameters, and I_D/I_G, using cross-validation and data augmentation techniques to enhance model robustness (***J. Mater. Chem. A 2025***, 13, 17748–17771). They not only confirmed the influence trends of single parameters but also revealed the synergistic and antagonistic effects among parameters through SHAP analysis (**Figure R4d**). These studies indicate that the design of high-performance HC does not pursue extreme values of a single structural parameter, but rather seeks an optimal "balance zone" where multiple parameters synergistically interact. The ML models serve as a powerful tool for identifying and defining this balance zone.

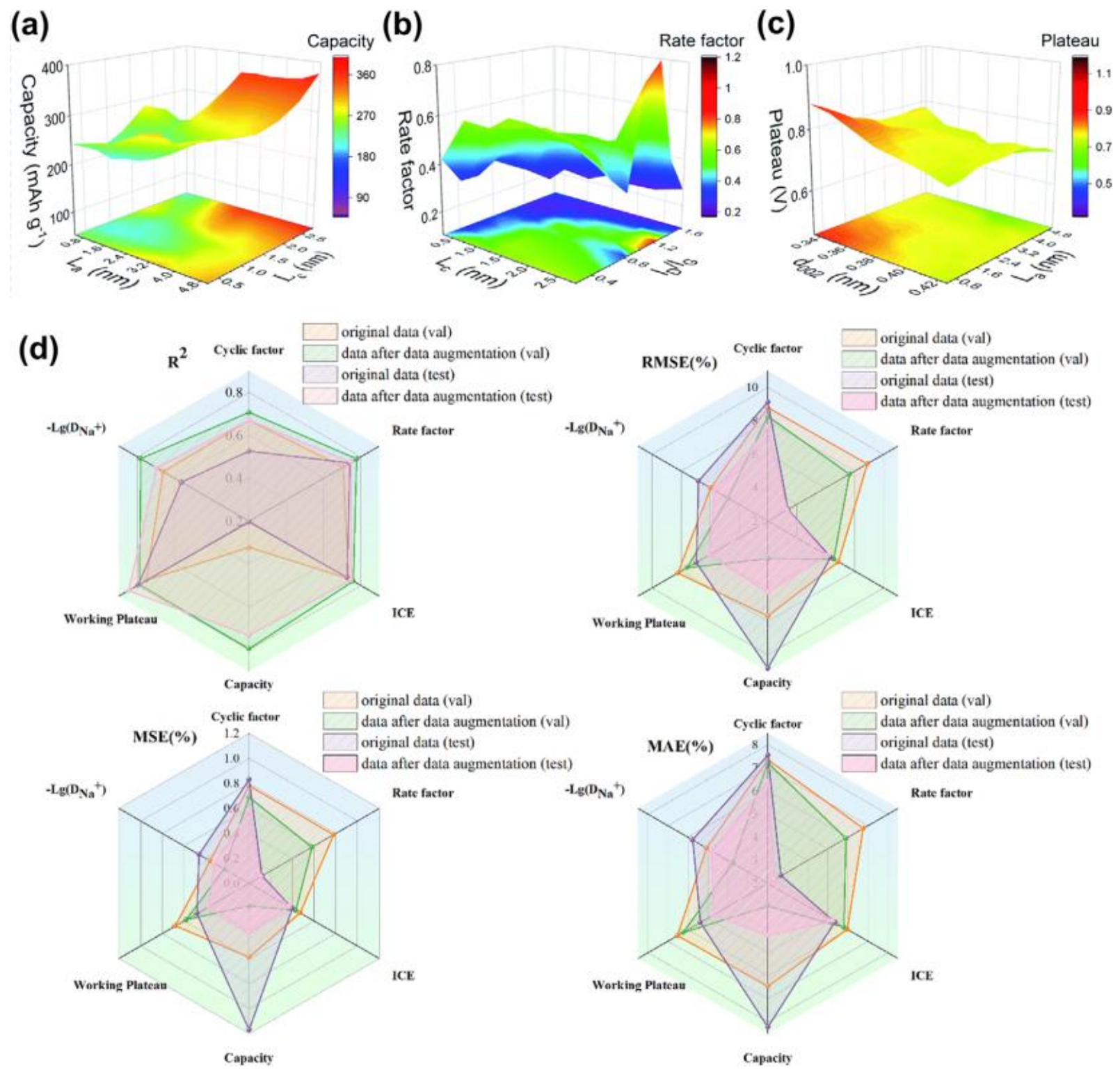

Figure R4. (a–c) Final prediction performance for 20000 sets of artificially constructed structure data. (d) For different output targets, comparisons were made using their respective optimal models. The specific models are as follows: cyclic factor, CatBoost; rate factor, AdaBoost; ICE, Bagging; capacity, Stacking; working plateau, GBDT; $-lgD_{Na+}$, XGB.

By incorporating ML algorithms, this study decouples the complex and highly nonlinear relationship between the microstructure parameters of HC and its electrochemical performance, thereby overcoming the limitations of traditional empirical rules. Specifically, we collected 11 feature values and 3 target values for ML (**Figure R5a**). By analyzing the correlations among the variables, we identified highly correlated feature values (carbonization temperature, d_{002}, I_D/I_G, and SSA) and target values (ICE and reversible plateau capacity) to improve the accuracy of the ML model. Based on the Random Forest model algorithm, the analysis using SHAP values not only quantifies the independent contributions of various feature values (carbonization temperature, d_{002}, I_D/I_G, and SSA) to performance but also reveals the interactions among these parameters (**Figure R5b, c**). The results indicate a significant synergistic effect between d_{002} and I_D/I_G. Even at high I_D/I_G, a smaller d_{002} can still maintain excellent ion diffusion kinetics, thereby compensating for the kinetic disadvantages associated with insufficient d_{002}. Furthermore, by using SHAP values to fit

the nonlinear response curves between key parameters and performance, we identified threshold effects that were overlooked by traditional empirical rules, forming a distinct inflection point in the performance trade-off. This is not an absolute prediction of a single structure for high-performance HC, but rather a microstructural synergy window that maximizes electrochemical performance under complex, multivariate coupling, characterized by high carbonization temperature (1200-1800°C), low SSA (0.26-17.84 m^2 g^{-1}), moderate d_{002} (0.376-0.394 nm), and moderate I_D/I_G (1.10-2.30, **Figure R6**). This transition from qualitative consensus to quantitative mechanisms not only elaborates the interactions among microstructural features but also provides precise data-driven guidance under multiple constraints, demonstrating the unique value of ML in the development of high-performance HC materials.

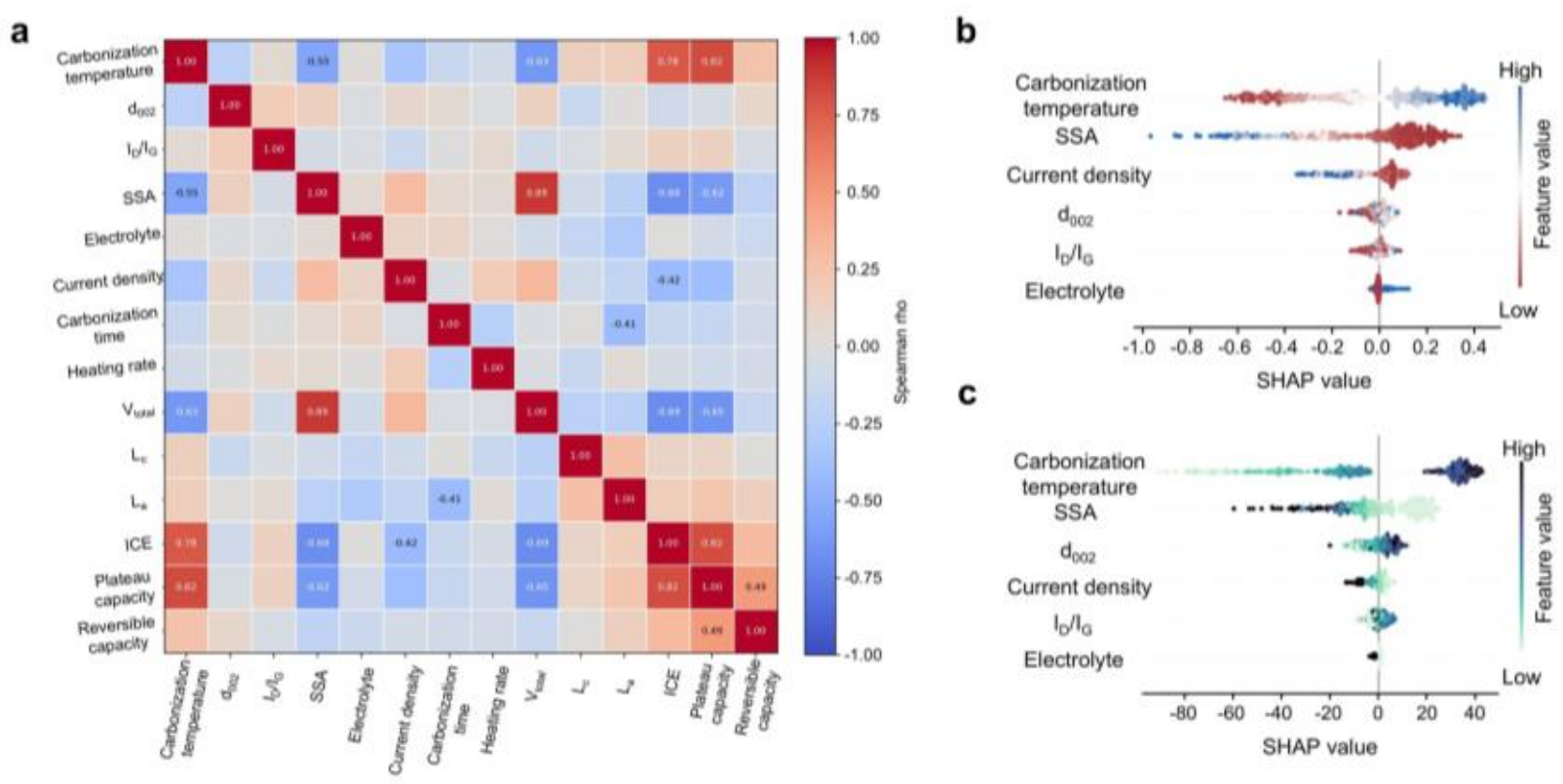

Figure R5. (a) Spearman correlation heatmap between core features, missing variables, and the target variable. The SHAP analysis honeycomb diagram of (b) ICE and (c) reversible plateau capacity.

There is a strong intrinsic correlation and logical complementarity between the nonlinear microstructure-property relationships revealed by ML and low-temperature thermal-force coupling preparation strategies. This correlation is evident in the following three aspects. **(1) ML algorithms provide target parameter windows for experimental strategies**. Based on the results of ML analysis, conventional low-temperature pyrolysis often results in high d_{002}, high I_D/I_G, and high SSA, deviating from the microstructural synergy window. Applying pressure at low temperatures not only rapidly reduces SSA but also promotes carbon layer rearrangement and structural graphitization. Through thermal-force coupling effects, d_{002}, I_D/I_G, and SSA are forcibly maintained within the microstructural synergy window. **(2) Thermal-force coupling mechanisms explain the nonlinear microstructural interaction effects revealed by ML.** ML models have revealed a complex,

nonlinear relationship between d_{002} and I_D/I_G, characterized by both synergistic and antagonistic effects. Neither an increase in carbonization temperature nor an increase in pressure alone is sufficient to achieve optimal performance. Our experiments confirm that low temperature provides the thermodynamic driving force for the formation of the fundamental carbon framework, while pressure provides the mechanical work required to overcome the activation energy barrier and facilitate the rearrangement of carbon layers. This thermal-force coupling synergy corresponds to the multi-parameter coupling mechanism identified through ML. **(3) Establishing a closed-loop process of data-driven design and experimental validation.** This study uses ML to decouple and transform vague empirical rules into quantitative structure-performance mappings. Subsequently, through low-temperature thermal-force coupling synthesis, we validated the microstructure of the high-performance HC obtained via ML.

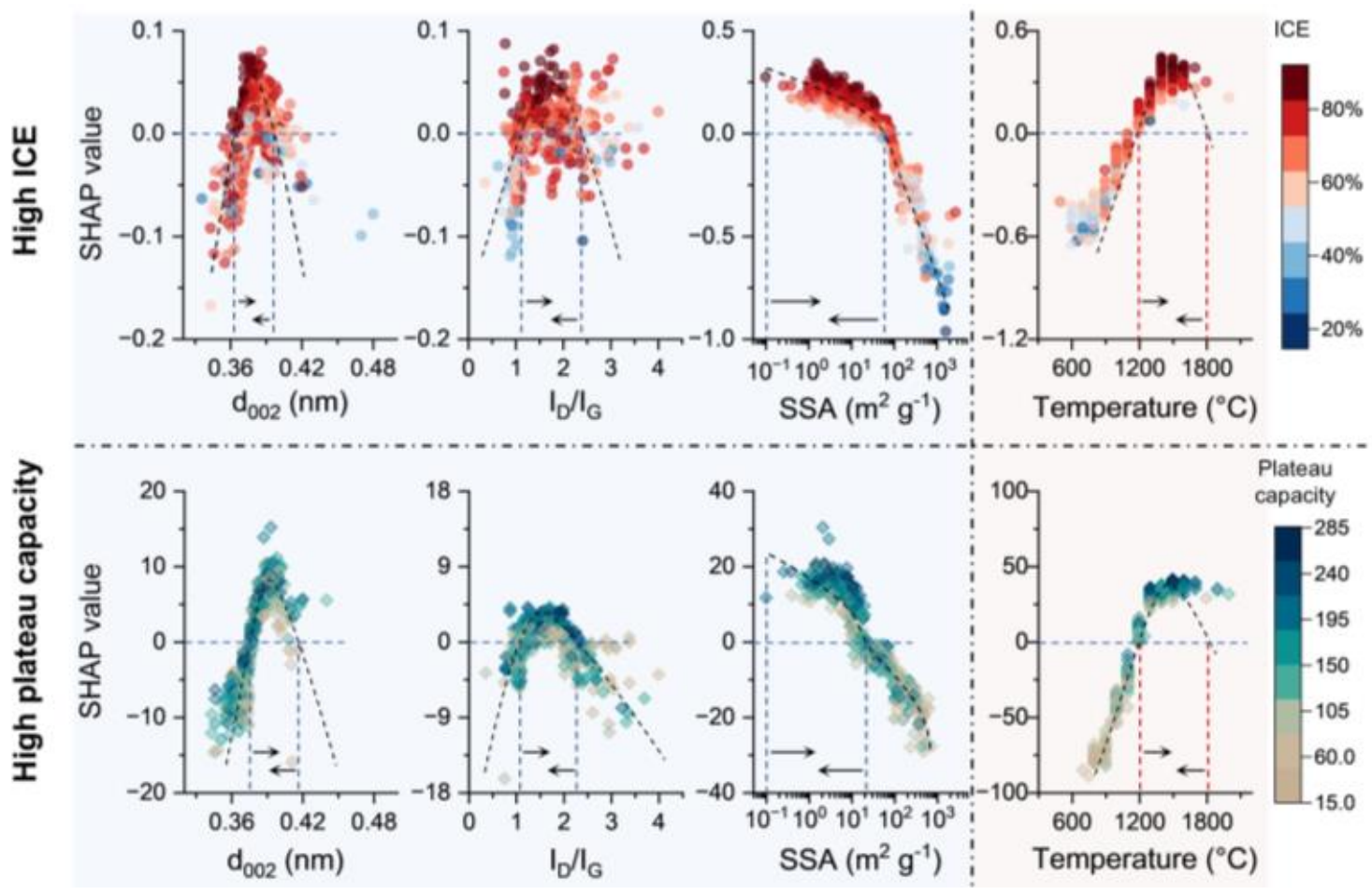

Figure R6. Relationship of ICE and reversible plateau capacity with each structure feature.

The electrochemical properties of HC are determined by its microstructure, rather than directly by the carbonization temperature. Based on ML algorithms, traditional views often regard carbonization temperature as a key process parameter, but temperature is merely one of the process parameters used to induce microstructural evolution. This study demonstrates that applying pressure at lower temperatures can also reproduce microstructures similar to those obtained through high-temperature processes. This suggests that temperature is not the only means of achieving specific microstructures. This reverse design thinking, which focuses on structure as the goal and process as the means, profoundly reveals the strong correlation between ML results and experimental validation.

Our revision:

1. Revised detailed description.

p 5, line 84-85 (Introduction)

Although numerous studies have reached a certain consensus regarding the influence of structure on performance, it is limited to qualitative descriptions and thus fails to provide precise guidance[26].

p 5, line 86-88 (Introduction)

Based on data from current literature regarding processes, structures, and performance, machine learning (ML) uncovers complex nonlinear relationships behind vast variables, revealing the key microstructure influence rules within trade-offs to provide quantifiable structure parameter thresholds[27,28].

p 5, line 94-96 (Introduction)

We analyze the nonlinear relationship between complex structure parameters and electrochemical performance based on the "preparation-microstructure-performance" database through the ML design paradigm.

p 8, line 156-159 (Results)

These findings establish that low SSA suppresses irreversible interfacial reactions, while moderate d_{002} and I_D/I_G provide an optimal balance between reversible sodium storage sites and Na^+ diffusion kinetics. The I_D/I_G may compensate for the kinetic lag caused by d_{002} by providing additional active sites.

p 9, line 166-167 (Results)

Traditional views often regard high carbonization temperature as a key process parameter, but temperature is only one of the process methods used to induce microstructural evolution.

p 9, line 167-169 (Results)

Thermal-force coupling strategies are proposed to prepare HC conforming to the microstructure synergy range at low carbonization temperatures, validating the accuracy of ML-driven design (**Fig. 2a**).

Comment #2. The "Force-Field" (pressure) is a new primary experimental variable, yet the authors have failed to move beyond a phenomenological description. There is a lack of understanding of how 10-100 MPa of pressure compensates for a 400°C thermal gap. Without evidence of how

pressure lowers the activation energy for carbon layer rearrangement or alters degassing kinetics, the manuscript remains a report of an experimental observation, not a study of a scientific mechanism.

Response: We are very grateful to the reviewers for their insightful suggestions regarding the activation energy reduction for carbon layer rearrangement due to thermal-force coupling. We fully agree that the manuscript lacks an explanation of the microscopic mechanisms underlying the pressure-compensated thermal effect and that experimental phenomena alone are insufficient to reveal the scientific essence. In response to your concerns, we have included the following significant additions and in-depth analyses in the revised manuscript regarding kinetics, thermodynamics, molecular dynamics simulations, and structural characterization.

Thermodynamic Analysis: For any material, the molar Gibbs free energy satisfies the equation ($dG = VdP - SdT$). During the graphitization process of HC, the reaction Gibbs free energy is $\Delta G(P, T) = G_{graphite}(P, T) - G_{hard\ carbon}(P, T)$. At a constant temperature, as the pressure increases from P_0 to P, the equation can be approximated as $\Delta G(P, T) \approx \Delta G(P_0, T) + \Delta V(P-P_0)$. Among these, $\Delta V = V_{graphite} - V_{hard\ carbon}$. For low-density, highly porous, and loosely structured carbon precursors, the graphitization process not only involves the ordering of bonds but also the densification of the structure. In this study, the true density of HC produced by thermal-force coupling carbonization was generally lower than that of graphite (2.26 g cm^{-3}), resulting in $\Delta V < 0$ (**Table R1**). Compared to other materials, HC-10-900 has the lowest true density and the lowest ΔV, and its ΔG value decreases more significantly. Therefore, carbon structures exhibit a significant thermodynamic driving force for ordered rearrangement at lower temperatures through the application of pressure.

Table R1. The true density of samples.

Sample	True density (g cm^{-3})
HC-0-900	1.8551
HC-5-900	1.7685
HC-10-900	1.6109
HC-50-900	1.9614
HC-100-900	2.1426
Graphite	2.2600

Kinetics Analysis: According to the Arrhenius equation, a set of rate constants k corresponding to different temperatures T is measured at each fixed pressure P. The slope is then determined based on the linear relationship between lnk and 1/T, thereby yielding the apparent activation energy at that pressure.

Arrhenius equation: $k(P,T) = A(P)exp\left[-\frac{E_aP}{RT}\right]$

Take the natural logarithm: $\ln k(P,T) = \ln A(p) - \frac{E_aP}{R}\frac{1}{T}$

To compare the apparent activation energies, we kept the pressure constant (Atmospheric pressure and 10 MPa) and varied the carbonization temperature (900, 1100, 1300°C), converting the degree of graphitization of HC into the rate constant (k). The graphitization degree (R-factor) is obtained by measuring the ratio between the (002) peak intensity (B) and the background intensity (A) in the XRD pattern (**Figure R7**). The low R factor indicates that under the formation conditions, the graphene layers lacked sufficient thermal energy to rotate into a parallel alignment.

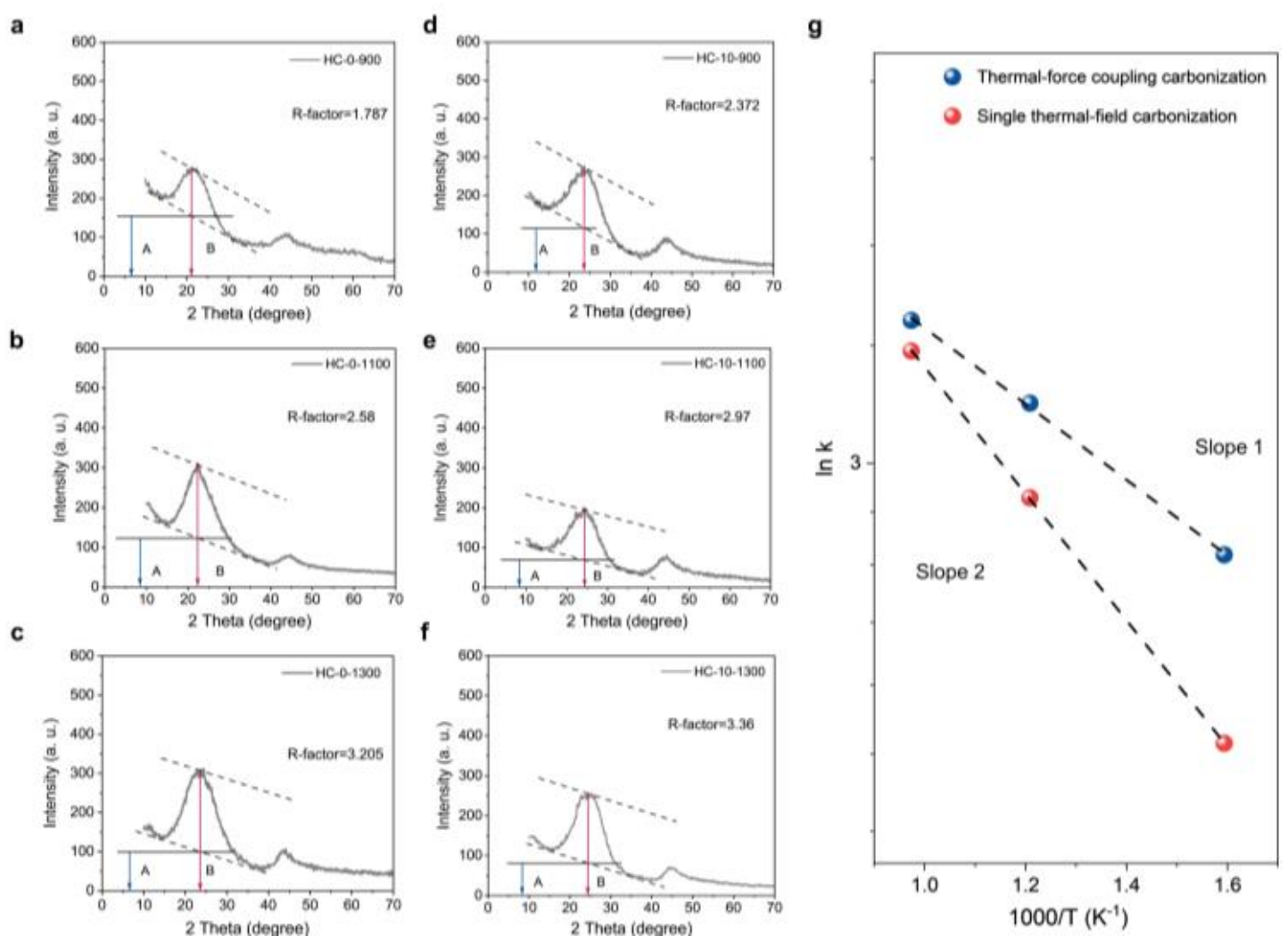

Figure R7. The R-factor of (a) HC-0-900, (b) HC-0-1100, (c) HC-0-1300, (d) HC-10-900, (e) HC-10-1100, and (f) HC-10-1300. (g) Comparison of activation energies of thermal-force coupling carbonization and single thermal-field carbonization.

Calculations show that the slope of the thermal-force coupling process is significantly smaller than that of the conventional pyrolysis process, indicating that the additional pressure significantly reduces the activation energy required for the ordered rearrangement of the carbon layer. The pressure compresses the gaps between the carbon layers, increases the van der Waals interactions

between carbon atoms, and reduces the diffusion activation energy required for relative sliding and stacking between the carbon layers, thereby compensating for the lack of thermal kinetic energy at low temperatures.

Molecular dynamics simulation: Molecular dynamics simulations with a reactive force field were further applied to reveal the thermal-force coupling mechanisms underlying the microstructural evolution of HC (**Figure R8**). 337 glucose was randomly packed in the simulation cell with side lengths of 40 Å, which is close to the true density of glucose. During the pre-carbonization stage, the carbon material exhibits a highly disordered amorphous structure due to the thermal decomposition of glucose, generating a large amount of gas. During the pyrolysis simulation, the temperature was set at 1173.15 K, and the pressures were atmospheric, 10 MPa, and 100 MPa, respectively, followed by a simulation of 300 ps in the NPT (constant pressure and temperature) system. The HC consists of irregular and short carbon fragments during atmospheric and low-temperature carbonization, exposing more defects and pore structures, which are consistent with the characterized structure. Under the pressure of 10 MPa, the short carbon fragments accelerate cross-linking to promote the epitaxial growth of the carbon layer and form a closed-pore structure between the curved pseudo-graphite microdomains, which provide extra sodium storage sites. As the pressure is increased to 100 MPa, the carbon layers are more closely packed, and the orderliness of the surface structure is further increased.

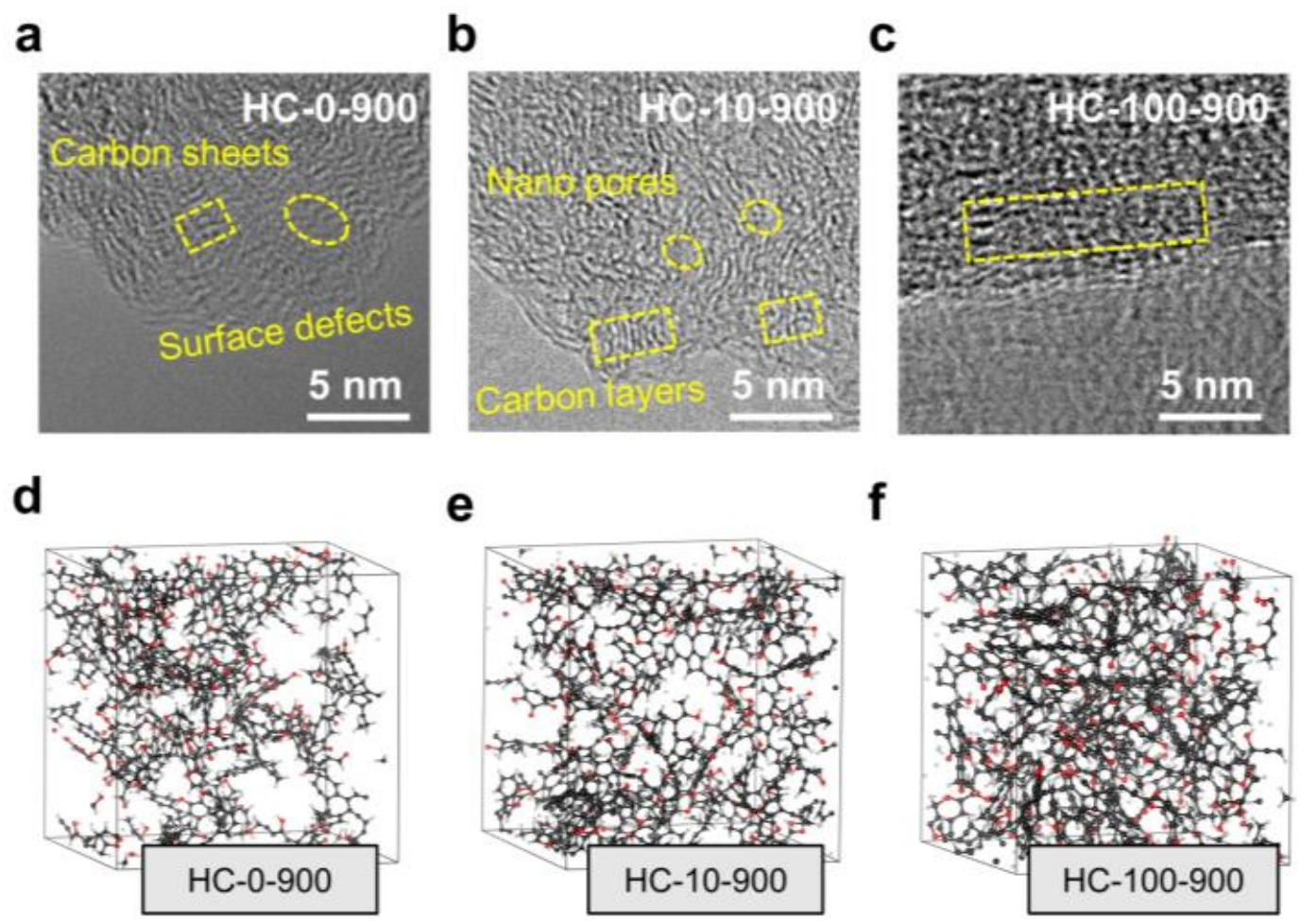

Figure R8. (a-c) HRTEM images, and (d-f) Molecular dynamics simulations of HC-0-900, HC-10-900, and HC-100-900.

In summary, structural characterization, thermodynamic and kinetic analyses, and molecular

dynamics simulations have demonstrated that the thermal-force coupling mechanism not only facilitates effective cross-linking and stacking of the carbon structure but also prevents excessive graphitization at high temperatures, thereby precisely controlling the microstructure of high-performance HC with low SSA, moderate d_{002}, and moderate I_D/I_G.

Our revision:

1. Revised detailed description.

p 10, line 193-195 (Results)

Since the graphitization process of the material is accompanied by volume contraction, the application of pressure significantly reduces the Gibbs free energy, providing the thermodynamic driving force for structural ordering at low temperatures.

p 10, line 195-198 (Results)

Furthermore, the applied pressure enhances interlayer van der Waals interactions by compressing the interlayer spacing, reducing the activation energy required for relative sliding, rotation, and stacking of the carbon layers, thereby effectively compensating for the lack of thermal energy at low temperatures (Supplementary Fig. 16).

Supplementary Note 6 (Supplementary Information)

According to the Arrhenius equation, a set of rate constants k corresponding to different temperatures T (900, 1100, 1300℃) is measured at fixed pressure P (Atmospheric pressure and 10 MPa). The slope is then determined based on the linear relationship between lnk and 1/T, thereby yielding the apparent activation energy at that pressure.

Arrhenius equation: $k(P,T) = A(P)exp\left[-\frac{E_a P}{RT}\right]$

Take the natural logarithm: $\ln k(P,T) = \ln A(p) - \frac{E_a P}{R}\frac{1}{T}$

Here, the degree of graphitization of HC is converted into a rate constant (k). The graphitization degree (R-factor) is obtained by measuring the ratio between the (002) peak intensity (B) and the background intensity (A) in the XRD pattern[14]. The low R factor indicates that under the formation conditions, the graphene layers lacked sufficient thermal energy to rotate into a parallel alignment. Calculations show that the slope of the thermal-force coupling process is significantly smaller than that of the conventional pyrolysis process, indicating that the additional pressure significantly reduces the activation energy required for the ordered rearrangement of the carbon layer.

2. Revised figures.

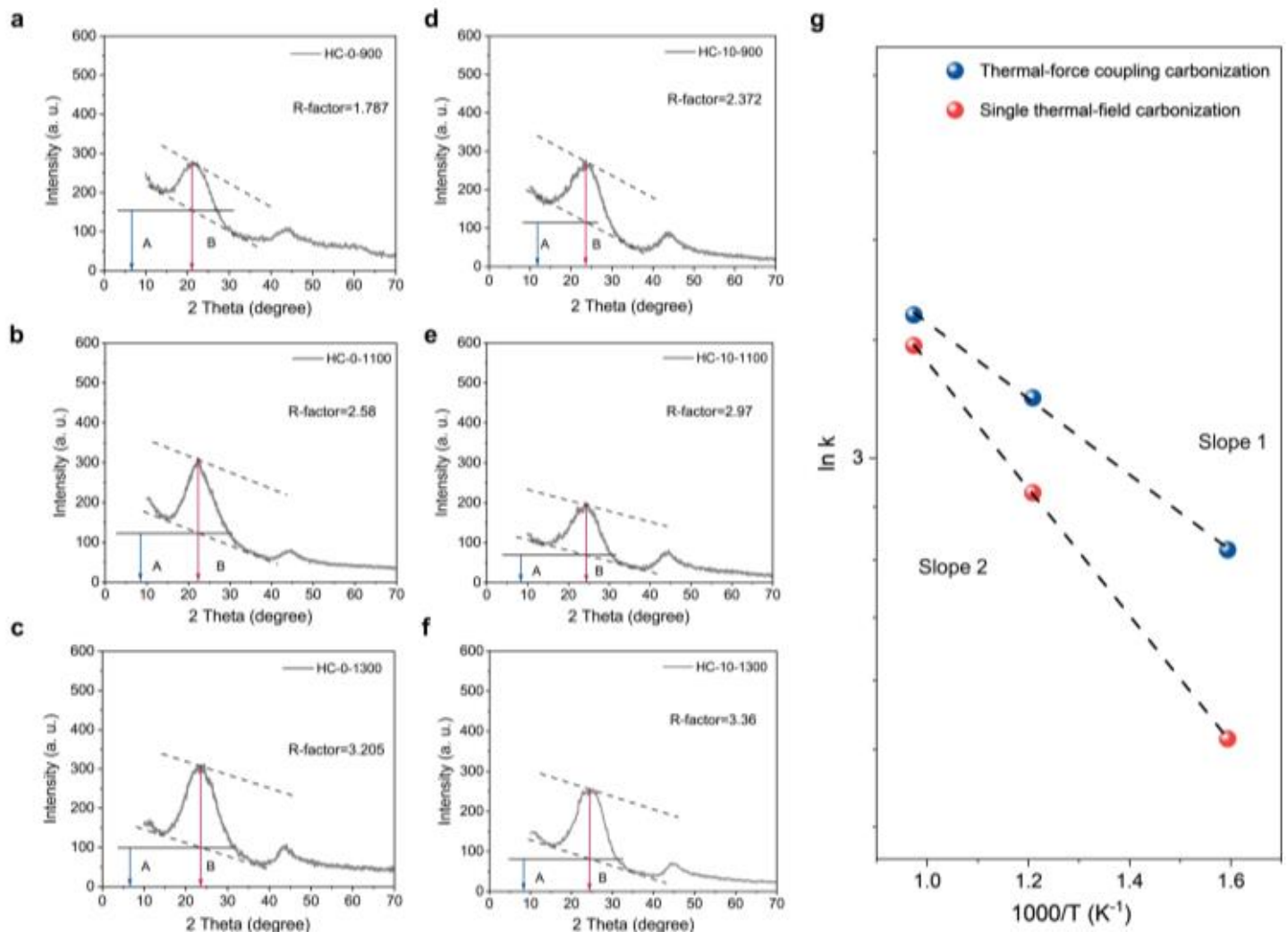

Supplementary Fig. 16. The R-factor of (a) HC-0-900, (b) HC-0-1100, (c) HC-0-1300, (d) HC-10-900, (e) HC-10-1100, and (f) HC-10-1300. (g) Comparison of activation energies of thermal-force coupling carbonization and single thermal-field carbonization.

Comment #3. The authors continue to emphasize highly specific energy (49.7%) and carbon reduction (47.5%), which are highly speculative. Quantifying "sustainability" to a 0.1% precision based on a laboratory pellet press is scientifically disingenuous and inappropriate. There are just too many factors that can easily jeopardize these idealized numbers.

Response: We are sincerely grateful to the reviewers for their insightful comments regarding precision, uncertainty, and scientific rigor in the economic analysis. The figures for energy consumption and carbon emissions are not estimates, but are calculated based on electricity usage and CO_2 emissions. Due to the energy-intensive laboratory-scale batch processing, which primarily includes the pre-carbonization and carbonization processes. The energy consumption is primarily reflected in electricity usage, and the carbon emissions are primarily reflected in CO_2 emissions. The electricity consumption is calculated by an electricity meter. We assume that the carbon-containing gases produced during the pre-carbonization and high-temperature carbonization stages consist solely of CO_2. Determine the mass percentage of the C element in pre-carbonized and high-temperature carbonized products by complete combustion using a carbon-sulfur analyzer. Calculating CO_2 emissions for the pre-carbonization and high-temperature carbonization stages based on the constant carbon content. The electricity consumption and CO_2 emissions for low-temperature thermal-force coupling carbonization were 107.8 kWh and 51.94 g, respectively, while

those for high-temperature thermal field carbonization were 214.2 kWh and 98.87 g. Calculations show that low-temperature thermal-force coupling carbonization significantly reduces energy consumption by 49.7% and carbon emissions by 47.5%. To address your concerns, we have toned down the numerical comparisons in the revised manuscript and clarified the energy consumption and carbon emissions associated with different pathways to enhance the scientific rigor of the study.

Our revision:

1. Revised detailed description.

p 3, line 45-47 (Abstract)

Remarkably, the required carbonization temperature reduces from 1300°C to 900°C, leading to a significant reduction in energy consumption and carbon emissions.

p 5, line 102-104 (Introduction)

Based on batch processing in the laboratory, low-temperature thermal-force (900°C-10 MPa) coupling carbonization significantly reduces energy consumption (from 214.2 to 107.8 kWh) and carbon emission (from 98.87 to 51.94 g) compared to traditional high-temperature (1300°C) carbonization.

p 20, line 418-421 (Discussion)

Compared to traditional high-temperature (1300°C) derived HC, the HC-10-900 synthesized at 900°C under 10 MPa simultaneously exhibits higher Na^+ storage sites and faster Na^+ diffusion kinetics, together with a large energy consumption reduction (from 214.2 to 107.8 kWh) and a large carbon emission reduction of (from 98.87 to 51.94 g).

We sincerely thank you for your constructive comments and careful evaluation of our work. We hope that the substantial revisions and new analyses provided in this response satisfactorily address all of your concerns.
