# Supplementary material for: 900°C thermal-force coupling carbonization tailoring hard carbon microstructures enables high performance sodium-ion pouch batteries
Source: Nat Commun. 2026 Sep 8;17:10620. doi: 10.1038/s41467-026-77440-0 (PMC13650117; doi:10.1038/s41467-026-77440-0)
Supplement: Supplementary file 4 [file 41467_2026_77440_MOESM3_ESM.zip › Supplementary Data files (Include molecular dynamics configuration and the parameter database of hard carbon for machine learning)/Molecular dynamics configuration/Inventory of Supplementary items-Molecular dynamics information.docx]

| **Sample** | **Figure panel** | **System composition** | | | | | | **Simulation stage** |
| --- | --- | --- | --- | --- | --- | --- | --- | --- |
|  |  | **Atom-type mapping** | | | **Cell dimensions (Å)** | **Temperature (K)** | **Pressure (atm)** |  |
|  |  | **Type** | **Element** | **number of atoms** |  |  |  |  |
| Glucose | Figure S17a | 1 | H | 4044 | 40.062 40.133  40.132 | 300 | 1 | Initial configuration |
|  |  | 6 | C | 2022 |  |  |  |  |
|  |  | 8 | O | 2022 |  |  |  |  |
| HC-P | Figure S17b | 1 | H | 802 | 29.05  29.10  29.10 | 300 | 1 | Intermediate configuration |
|  |  | 6 | C | 1079 |  |  |  |  |
|  |  | 8 | O | 154 |  |  |  |  |
| HC-0-900 | Figure 2g | 1 | H | 768 | 36.81  36.87  36.87 | 1173.15 | 1 | Final configuration |
|  |  | 6 | C | 1071 |  |  |  |  |
|  |  | 8 | O | 140 |  |  |  |  |
| HC-10-900 | Figure 2h | 1 | H | 752 | 34.24  34.30  34.30 | 1173.15 | 98.69 | Final configuration |
|  |  | 6 | C | 1068 |  |  |  |  |
|  |  | 8 | O | 135 |  |  |  |  |
| HC-100-900 | Figure 2i | 1 | H | 754 | 29.06  29.11  29.11 | 1173.15 | 986.9 | Final configuration |
|  |  | 6 | C | 1070 |  |  |  |  |
|  |  | 8 | O | 139 |  |  |  |  |
