# Supplementary material for: 900°C thermal-force coupling carbonization tailoring hard carbon microstructures enables high performance sodium-ion pouch batteries
Source: Nat Commun. 2026 Sep 8;17:10620. doi: 10.1038/s41467-026-77440-0 (PMC13650117; doi:10.1038/s41467-026-77440-0)
Supplement: Supplementary file 4 [file 41467_2026_77440_MOESM3_ESM.zip › Supplementary Data files (Include molecular dynamics configuration and the parameter database of hard carbon for machine learning)/Supplementary Data 1 (Legends-The parameter database of hard carbon for machine learning).doc]

**Supplementary Data**

**900°C Thermal-Force Coupling Carbonization Tailoring Hard Carbon Microstructures Enables High Performance Sodium-Ion Pouch Batteries**

**Supplementary Data 1**. The parameter database of hard carbon for machine learning, include process parameters, structural parameters and performance parameters.

| Ref. | Sample | Carbonization temperature  (°C) | Carbonization time (h) | Heating rate  (°C/min) | *d_002_* (nm) | *I_D_*/*I_G_* | SSA  (m^2^ g^-1^) | *L_a_* (nm) | *L_c_* (nm) | V_total_  (cm^3^ g^-1^) | Electrolyte | Current density  (mA g^-1^) | ICE (%) | Reversible capacity  (mAh g^-1^) | Reversible plateau capacity  (mAh g^-1^) |
| --- | --- | --- | --- | --- | --- | --- | --- | --- | --- | --- | --- | --- | --- | --- | --- |
| 1 | HC-900 | 900 | 2 | 3 | 0.387 | 1.110 | 209.30 | - | - | - | 1 M NaPF_6_ in diglyme | 20 | 62.10 | 138.40 | 0.00 |
|  | HC-1000 | 1000 | 2 | 3 | 0.385 | 1.230 | 571.70 | - | - | - | 1 M NaPF_6_ in diglyme | 20 | 64.10 | 204.10 | 0.00 |
|  | HC-1100 | 1100 | 2 | 3 | 0.382 | 1.170 | 33.03 | - | - | - | 1 M NaPF_6_ in diglyme | 20 | 72.40 | 342.40 | 106.00 |
|  | HC-1200 | 1200 | 2 | 3 | 0.380 | 1.160 | 167.30 | - | - | - | 1 M NaPF_6_ in diglyme | 20 | 66.40 | 263.50 | 125.00 |
|  | HC-1100A | 1100 | 2 | 3 | 0.382 | 1.160 | 46.30 | - | - | - | 1 M NaPF_6_ in diglyme | 20 | 72.10 | 350.30 | 140.00 |
| 2 | EU1100 | 1100 | 0.5 | 5 | 0.380 | 2.630 | 231.10 | - | - | - | 1 M NaCF_3_SO_3_ in diglyme | 20 | 63.60 | 221.00 | 62.00 |
|  | EU1300 | 1300 | 0.5 | 5 | 0.375 | 2.020 | 240.60 | - | - | - | 1 M NaCF_3_SO_3_ in diglyme | 20 | 68.20 | 225.00 | 81.00 |
|  | EU1100M | 1100 | 0.5 | 5 | 0.388 | 2.970 | 29.20 | - | - | - | 1 M NaCF_3_SO_3_ in diglyme | 20 | 69.50 | 252.00 | 72.00 |
|  | EU1300M | 1300 | 0.5 | 5 | 0.378 | 2.390 | 34.50 | - | - | - | 1 M NaCF_3_SO_3_ in diglyme | 20 | 74.60 | 268.00 | 118.00 |
| 3 | HFHCN | 1400 | 8 | 1 | 0.370 | 0.870 | 280.00 | - | - | 0.0876 | 1 M NaClO_4_ in EC: DMC (1:1) | 20 | 61.10 | 390.80 | 191.60 |
|  | CHCM | 1400 | 8 | 1 | 0.380 | 1.040 | 0.70 | - | - | - | 1 M NaClO_4_ in EC: DMC (1:1) | 20 | 75.70 | 333.20 | 181.50 |
| 4 | Cel-1500 | 1500 | 2 | - | 0.372 | 1.856 | 10.80 | 3.968 | 1.311 | 0.0027 | 1 M NaPF_6_ in EC: DEC (1:1) | 20 | 76.60 | 342.50 | 200.00 |
|  | Cel-O250–1500 | 1500 | 2 | - | 0.376 | 1.772 | 7.70 | 4.140 | 1.228 | 0.0023 | 1 M NaPF_6_ in EC: DEC (1:1) | 20 | 77.90 | 344.40 | 237.00 |
|  | Cel-O300–1500 | 1500 | 2 | - | 0.383 | 1.764 | 8.70 | 4.392 | 1.147 | 0.0022 | 1 M NaPF_6_ in EC: DEC (1:1) | 20 | 74.80 | 426.80 | 266.80 |
|  | Cel-Ar300–1500 | 1500 | 2 | - | 0.389 | 1.755 | 11.10 | 4.694 | 1.149 | 0.0024 | 1 M NaPF_6_ in EC: DEC (1:1) | 20 | 75.60 | 361.00 | 217.30 |
| 5 | GLU-Zn5 | 1400 | 2 | 5 | 0.383 | 1.116 | 3.57 | 3.660 | 0.840 | - | 1 M NaCF_3_SO_3_ in diglyme | 50 | 82.30 | 327.27 | 203.64 |
|  | GLU-Zn10 | 1400 | 2 | 5 | 0.393 | 1.057 | 4.69 | 3.710 | 0.990 | - | 1 M NaCF_3_SO_3_ in diglyme | 50 | 84.00 | 333.48 | 210.00 |
|  | GLU-Zn20 | 1400 | 2 | 5 | 0.378 | 1.012 | 4.40 | 3.130 | 1.420 | - | 1 M NaCF_3_SO_3_ in diglyme | 50 | 78.80 | 256.73 | 154.18 |
| 6 | BA | 1200 | 2 | 5 | 0.372 | 0.950 | 5.51 | 4.310 | - | 0.0026 | 1 M NaPF_6_ in EC: EDC (1:1) | 50 | 66.80 | 347.60 | 164.20 |
|  | MMZ | 1200 | 2 | 5 | 0.364 | 1.430 | 0.40 | 2.850 | 1.040 | 0.0094 | 1 M NaPF_6_ in EC: EDC (1:1) | 50 | 68.02 | 206.50 | 109.90 |
|  | IQL | 1200 | 2 | 5 | 0.365 | 1.170 | 0.74 | 3.000 | 0.780 | 0.0010 | 1 M NaPF_6_ in EC: EDC (1:1) | 50 | 67.42 | 220.00 | 99.20 |
| 7 | CNFs | 800 | 1 | 5 | 0.355 | 1.620 | 294.50 | - | - | 0.1550 | 1 M NaPF_6_ in DME | 200 | 48.50 | 170.00 | 0.00 |
| 8 | MCC-650 | 650 | 1 | 3 | 0.365 | 2.180 | 1529.10 | - | - | 1.4490 | 1 M NaPF_6_ in DME | 100 | 14.70 | 168.42 | 0.00 |
| 9 | HC | 750 | 2 | 3 | 0.378 | 1.290 | 1752.00 | - | - | 1.5456 | 1 M NaClO_4_ in EC: DEC (1:1) | 100 | 33.10 | 256.00 | 0.00 |
|  | P-HC-2 | 750 | 2 | 3 | 0.391 | 2.040 | 706.00 | - | - | 0.6814 | 1 M NaClO_4_ in EC: DEC (1:1) | 100 | 42.10 | 394.00 | 0.00 |
| 10 | NHCM-1200 | 1200 | 3 | 3 | 0.410 | 2.690 | 14.50 | 8.900 | 2.500 | - | 1 M NaPF_6_ in diglyme | 30 | 71.00 | 336.00 | 174.72 |
|  | NHCM-1400 | 1400 | 3 | 3 | 0.370 | 1.990 | 8.60 | 11.500 | 3.000 | - | 1 M NaPF_6_ in diglyme | 30 | 87.00 | 399.00 | 203.49 |
| 11 | FC-800 | 1400 | 2 | 3 | 0.363 | 1.490 | 14.30 | - | - | 0.0310 | 1 M NaPF_6_ in EC: DEC (1:1) | 20 | 84.20 | 279.90 | 174.90 |
|  | FC-0.5–600 | 1400 | 2 | 3 | 0.371 | 1.620 | 26.30 | - | - | 0.0320 | 1 M NaPF_6_ in EC: DEC (1:1) | 20 | 84.00 | 287.30 | 186.90 |
|  | FC-800–0.5–600 | 1400 | 2 | 3 | 0.370 | 1.540 | 6.80 | - | - | 0.0220 | 1 M NaPF_6_ in EC: DEC (1:1) | 20 | 84.70 | 326.80 | 218.60 |
| 12 | HC-Cell | 1500 | 1 | 5 | 0.378 | 1.610 | 12.10 | 4.200 | 1.100 | 0.0084 | 1 M NaPF_6_ in EC: DMC (1:1) with 2% FEC | 37.2 | 87.00 | 307.60 | 214.83 |
|  | HC-L | 1500 | 1 | 5 | 0.375 | 1.640 | 6.00 | 4.500 | 1.100 | 0.0052 | 1 M NaPF_6_ in EC: DMC (1:1) with 2% FEC | 37.2 | 80.30 | 273.20 | 194.29 |
|  | HC-Ch | 1500 | 1 | 5 | 0.373 | 1.590 | 2.40 | 4.500 | 1.200 | 0.0031 | 1 M NaPF_6_ in EC: DMC (1:1) with 2% FEC | 37.2 | 86.00 | 256.30 | 169.96 |
|  | HC-P-St | 1500 | 1 | 5 | 0.377 | 1.630 | 2.40 | 4.200 | 1.100 | 0.0025 | 1 M NaPF_6_ in EC: DMC (1:1) with 2% FEC | 37.2 | 88.60 | 291.60 | 199.62 |
|  | HC-R-St | 1500 | 1 | 5 | 0.379 | 1.620 | 14.70 | 4.000 | 1.000 | 0.0097 | 1 M NaPF_6_ in EC: DMC (1:1) with 2% FEC | 37.2 | 85.80 | 308.60 | 205.32 |
| 13 | HC | 1100 | 2 | - | 0.360 | 1.780 | 9.80 | - | - | - | 1 M NaClO_4_ in EC: DEC (1:1) | 25 | 68.34 | 257.20 | 115.88 |
| 14 | CHC-1100 | 1100 | 1 | 5 | 0.404 | 2.580 | 8.44 | 4.240 | 1.190 | - | 1 M NaClO_4_ in EC: DEC (1:1) | 30 | 67.90 | 327.90 | 148.48 |
|  | CHC-1300 | 1300 | 1 | 5 | 0.397 | 2.390 | 7.12 | 4.330 | 1.240 | - | 1 M NaClO_4_ in EC: DEC (1:1) | 30 | 71.40 | 373.20 | 199.63 |
| 15 | A-HC1100 | 1100 | 3 | 2 | 0.372 | 1.240 | 3.88 | - | - | - | 1 M NaClO_4_ in EC: DEC (1:1) with 5% FEC | 15 | 67.29 | 305.00 | 125.00 |
| 16 | HC700 | 700 | 3 | 5 | 0.381 | 3.380 | 1.08 | - | - | 0.0060 | 1 M NaPF_6_ in diglyme | 30 | 53.34 | 170.40 | 37.60 |
|  | HC1100 | 1100 | 3 | 5 | 0.374 | 2.660 | 1.87 | - | - | 0.0060 | 1 M NaPF_6_ in diglyme | 30 | 79.13 | 217.00 | 97.00 |
|  | HC1500 | 1500 | 3 | 5 | 0.358 | 1.630 | 4.49 | - | - | 0.0100 | 1 M NaPF_6_ in diglyme | 30 | 79.07 | 206.20 | 118.30 |
| 17 | N/O-HC-1200 | 1200 | - | 5 | 0.385 | 2.270 | 4.71 | - | - | 0.0570 | 1 M NaClO_4_ in EC: DEC (1:1) with 5% FEC | 30 | 81.60 | 319.70 | 186.20 |
| 18 | HC | 1200 | 2 | 5 | 0.399 | 1.150 | 17.84 | - | - | 0.0144 | 1 M NaPF_6_ in EC: DEC (1:1) | 50 | 64.70 | 306.40 | 157.88 |
|  | HC-Al_2_O_3_-5 | 1200 | 2 | 5 | 0.393 | 1.050 | 6.91 | - | - | 0.0099 | 1 M NaPF_6_ in EC: DEC (1:1) | 50 | 81.10 | 334.90 | 189.73 |
| 19 | PCS | 800 | 2 | - | 0.390 | 2.110 | 468.00 | - | 1.210 | 0.1620 | 1 M NaPF_6_ in DEGDME | 100 | 47.10 | 155.30 | 0.00 |
| 20 | S-NC | 600 | 2 | 5 | 0.420 | 1.550 | 21.15 | - | - | 0.1310 | 1 M NaClO_4_ in EC: DEC (1:1) with 5% FEC | 100 | 50.70 | 464.70 | 0.00 |
| 21 | EPNHC-1400 | 1400 | 1 | 2 | 0.389 | 1.408 | 3.37 | 1.875 | 1.764 | 0.0073 | 1 M NaPF_6_ in DME | 50 | 82.60 | 403.90 | 243.07 |
| 22 | HC 900 | 900 | 8 | - | 0.375 | 1.710 | 479.00 | 1.650 | 1.070 | - | 1 M NaPF_6_ in EC: DEC (1:1) | 15 | 48.20 | 164.00 | 0.00 |
|  | HC 1100 | 1100 | 8 | - | 0.374 | 1.770 | 277.00 | 1.700 | 1.110 | - | 1 M NaPF_6_ in EC: DEC (1:1) | 15 | 70.90 | 265.00 | 108.97 |
|  | HC 1300 | 1300 | 8 | - | 0.378 | 1.440 | 1.60 | 1.780 | 1.140 | - | 1 M NaPF_6_ in EC: DEC (1:1) | 15 | 81.50 | 273.00 | 141.35 |
|  | HC 1400 | 1400 | 8 | - | 0.372 | 1.599 | 29.40 | 2.020 | 1.230 | - | 1 M NaPF_6_ in EC: DEC (1:1) | 15 | 74.30 | 286.00 | 175.77 |
|  | HC 1500 | 1500 | 8 | - | 0.372 | 1.990 | 2.85 | 2.340 | 1.240 | - | 1 M NaPF_6_ in EC: DEC (1:1) | 15 | 88.00 | 220.00 | 137.32 |
|  | HC 1600 | 1600 | 8 | - | 0.368 | 2.050 | 16.15 | 2.310 | 1.320 | - | 1 M NaPF_6_ in EC: DEC (1:1) | 15 | 81.60 | 200.00 | 128.27 |
| 23 | MPC | 800 | 2 | 5 | 0.381 | 1.090 | 547.60 | - | - | 2.5640 | 1 M NaPF_6_ in diglyme | 100 | 43.00 | 149.00 | 0.00 |
| 24 | FC-3h-1300 | 1300 | 2 | 1 | 0.383 | 2.422 | 74.00 | 4.14 | - | 0.2190 | 1 M NaPF_6_ in DEGDEM | 20 | 80.70 | 435.50 | 274.48 |
| 25 | PFNC-1100 | 1100 | 2 | 5 | 0.381 | 1.680 | 67.00 | 1.320 | - | - | 1 M NaPF_6_ in DEC: EC (1:1) | 20 | 64.78 | 188.20 | 67.89 |
|  | PFNC-1300 | 1300 | 2 | 5 | 0.380 | 1.530 | 35.53 | 1.450 | - | - | 1 M NaPF_6_ in DEC: EC (1:1) | 20 | 72.88 | 315.50 | 157.28 |
| 26 | PCNF-1 | 1400 | 2 | 5 | 0.364 | 1.235 | 69.00 | - | - | - | 1 M NaPF_6_ in diglyme | 50 | 81.60 | 285.50 | 166.48 |
|  | PCNF-2 | 1400 | 2 | 5 | 0.362 | 1.224 | 68.20 | - | - | - | 1 M NaPF_6_ in diglyme | 50 | 82.60 | 292.30 | 164.80 |
| 27 | N/S HC | 1100 | 4 | 5 | 0.387 | 2.100 | 29.32 | - | - | 0.0434 | 1 M NaPF_6_ in EC: DEC (1:1) with 5% FEC | 30 | 66.00 | 290.00 | 87.36 |
| 28 | PF-S-55-1200 | 1200 | 2 | - | 0.380 | 2.100 | 2.30 | - | - | 0.0070 | 1 M NaClO_4_ in EC: DEC (1:1) | 30 | 86.40 | 323.00 | 209.30 |
| 29 | PTA-Lys-700 | 700 | 2 | 2 | 0.370 | 1.020 | 161.20 | - | - | 0.0921 | 1 M NaClO_4_ in EC: DMC (1:1) | 100 | 55.20 | 278.10 | 0.00 |
|  | PTA-Lys-800 | 800 | 2 | 2 | 0.390 | 1.030 | 189.70 | - | - | 0.2954 | 1 M NaClO_4_ in EC: DMC (1:1) | 100 | 51.30 | 367.20 | 0.00 |
|  | PTA-Lys-900 | 900 | 2 | 2 | 0.410 | 1.050 | 192.80 | - | - | 0.1224 | 1 M NaClO_4_ in EC: DMC (1:1) | 100 | 53.80 | 318.30 | 0.00 |
| 30 | SW700 | 700 | 2 | 5 | 0.386 | 1.530 | 290.00 | 2.130 | 2.020 | - | 1 M NaClO_4_ in EC: DEC (1:1) | 25 | 50.00 | 211.00 | 0.00 |
|  | SW900 | 900 | 2 | 5 | 0.381 | 1.420 | 255.00 | 3.250 | 2.190 | - | 1 M NaClO_4_ in EC: DEC (1:1) | 25 | 57.00 | 284.00 | 106.23 |
|  | SW1100 | 1100 | 2 | 5 | 0.371 | 1.380 | 90.00 | 6.890 | 2.450 | - | 1 M NaClO_4_ in EC: DEC (1:1) | 25 | 62.00 | 256.00 | 96.83 |
| 31 | P-HC | 1200 | 3 | 3 | 0.380 | 1.050 | 63.90 | - | - | 0.0360 | 1 M NaPF_6_ in EC: DEC (1:1) | 50 | 69.00 | 345.23 | 165.83 |
| 32 | G1000 | 1000 | 2 | - | 0.410 | 0.880 | 382.00 | 1.390 | 1.210 | - | 1 M NaPF_6_ in EC: DMC (1:1) | 30 | 62.88 | 213.95 | 52.71 |
|  | G1300 | 1300 | 2 | - | 0.393 | 0.990 | 10.00 | 1.480 | 1.250 | - | 1 M NaPF_6_ in EC: DMC (1:1) | 30 | 84.12 | 279.07 | 151.94 |
|  | G1500 | 1500 | 2 | - | 0.383 | 1.100 | 11.00 | 1.550 | 1.320 | - | 1 M NaPF_6_ in EC: DMC (1:1) | 30 | 86.90 | 346.53 | 217.99 |
|  | G1700 | 1700 | 2 | - | 0.363 | 1.130 | 7.00 | 2.170 | 1.410 | - | 1 M NaPF_6_ in EC: DMC (1:1) | 30 | 79.24 | 267.7 | 200.52 |
|  | G1900 | 1900 | 2 | - | 0.355 | 1.180 | 7.00 | 2.420 | 1.750 | - | 1 M NaPF_6_ in EC: DMC (1:1) | 30 | 70.06 | 190.2 | 134.31 |
| 33 | CAC-M4 | 800 | - | 10 | 0.359 | 2.320 | 108.00 | 3.090 | 1.320 | 0.0300 | 1 M NaClO_4_ in EC: DEC (1:1) | 50 | 53.15 | 341.52 | 0.00 |
| 34 | PFC | 1200 | 3 | 5 | 0.348 | 1.630 | 100.70 | - | - | 0.0770 | 1 M NaClO_4_ in EC: DEC (1:1) | 30 | 73.3 | 163.30 | 67.30 |
|  | PPFC-1-10-1200 | 1200 | 3 | 5 | 0.381 | 1.890 | 49.10 | - | - | 0.0380 | 1 M NaClO_4_ in EC: DEC (1:1) | 30 | 76.20 | 287.30 | 161.10 |
|  | PPFC-1-6-1200 | 1200 | 3 | 5 | 0.394 | 1.910 | 4.80 | - | - | 0.0400 | 1 M NaClO_4_ in EC: DEC (1:1) | 30 | 77.90 | 308.70 | 184.90 |
|  | PPFC-1-2-1200 | 1200 | 3 | 5 | 0.374 | 1.850 | 29.60 | - | - | 0.1090 | 1 M NaClO_4_ in EC: DEC (1:1) | 30 | 74.40 | 264.40 | 145.80 |
|  | PPFC-1-6-1100 | 1100 | 3 | 5 | 0.408 | 2.140 | 13.00 | - | - | 0.0450 | 1 M NaClO_4_ in EC: DEC (1:1) | 30 | 75.50 | 276.80 | 133.02 |
|  | PPFC-1-6-1300 | 1300 | 3 | 5 | 0.379 | 1.870 | 4.20 | - | - | - | 1 M NaClO_4_ in EC: DEC (1:1) | 30 | 80.40 | 276.00 | 149.72 |
| 35 | NSDC | 700 | 5 | - | 0.384 | 1.040 | 1041.20 | - | - | 0.6000 | 1 M NaPF_6_ in EC: PC (1:1) with 2% FEC | 100 | 33.27 | 366.10 | 0.00 |
| 36 | CC-1000 | 1000 | 2 | 5 | 0.403 | 2.110 | 393.84 | - | - | - | 1 M NaClO_4_ in EC: DMC (1:1) | 30 | 52.56 | 128.25 | 52.80 |
| 37 | SNS-0 | 800 | 2 | - | 0.370 | 1.090 | 477.84 | - | - | 0.6250 | 1 M NaClO_4_ in EC: PC (1:1) | 25 | 35.40 | 194.00 | 38.40 |
|  | SNS-0.5 | 800 | 2 | - | 0.380 | 1.060 | 449.07 | - | - | 0.7690 | 1 M NaClO_4_ in EC: PC (1:1) | 25 | 36.97 | 210.00 | 32.68 |
|  | SNS-1 | 800 | 2 | - | 0.380 | 1.170 | 282.87 | - | - | 0.3810 | 1 M NaClO_4_ in EC: PC (1:1) | 25 | 50.44 | 289.00 | 63.30 |
|  | SNS-1.5 | 800 | 2 | - | 0.380 | 1.090 | 505.32 | - | - | 0.6300 | 1 M NaClO_4_ in EC: PC (1:1) | 25 | 37.65 | 215.00 | 43.97 |
| 38 | NOS-HC900 | 900 | 2 | 5 | 0.392 | 1.020 | 55.80 | - | - | 0.2900 | 1 M NaPF_6_ in EC: DEC: EMC (1:1:1) with 0.5% FEC | 100 | 43.30 | 425.30 | 0.00 |
| 39 | NUCS | 800 | 2 | 5 | 0.391 | 0.960 | 75.70 | - | - | - | 1 M NaPF_6_ in diglyme | 100 | 63.00 | 176.30 | 46.33 |
| 40 | N-CNS-900 | 900 | 2 | 2 | 0.364 | 1.100 | 491.00 | 0.380 | 0.210 | 0.170 | 1 M NaPF_6_ in DME | 50 | 51.16 | 170.50 | 25.75 |
|  | N-CNS-1200 | 1200 | 2 | 2 | 0.377 | 0.980 | 341.00 | 3.150 | 1.550 | 0.110 | 1 M NaPF_6_ in DME | 50 | 65.40 | 183.60 | 51.50 |
| 41 | LC | 1200 | 2 | 5 | 0.346 | 0.980 | 169.52 | - | - | 0.2810 | 1 M NaClO_4_ in EC: DEC (1:1) with 5% FEC | 30 | 59.90 | 290.20 | 97.00 |
|  | PCLC-1 | 1200 | 2 | 5 | 0.386 | 1.750 | 5.95 | - | - | 0.0240 | 1 M NaClO_4_ in EC: DEC (1:1) with 5% FEC | 30 | 85.30 | 312.20 | 185.70 |
|  | PCLC-10 | 1200 | 2 | 5 | 0.374 | 1.490 | 4.60 | - | - | 0.0310 | 1 M NaClO_4_ in EC: DEC (1:1) with 5% FEC | 30 | 79.70 | 275.30 | 151.90 |
| 42 | HC-C1500 | 1500 | 1 | 5 | 0.367 | 1.420 | 11.20 | 3.900 | 1.150 | 0.0141 | 1 M NaPF_6_ in EC: DMC (1: 1) | 7.44 | 83.00 | 286.00 | 186.75 |
|  | HC-MC1500 | 1500 | 1 | 5 | 0.373 | 1.550 | 8.50 | 3.830 | 1.040 | 0.0064 | 1 M NaPF_6_ in EC: DMC (1: 1) | 7.44 | 85.00 | 329.00 | 227.76 |
|  | HC-MG1500 | 1500 | 1 | 5 | 0.375 | 1.540 | 6.00 | 3.760 | 1.080 | 0.0069 | 1 M NaPF_6_ in EC: DMC (1: 1) | 7.44 | 85.00 | 317.00 | 218.93 |
|  | HC-MG1600 | 1600 | 1 | 5 | 0.370 | 1.440 | 8.20 | 3.940 | 1.130 | 0.0070 | 1 M NaPF_6_ in EC: DMC (1: 1) | 7.44 | 88.00 | 313.00 | 229.34 |
| 43 | PHCS1 | 800 | 2 | 5 | 0.394 | 0.957 | 305.05 | - | - | 0.1921 | 1 M NaPF_6_ in diglyme | 100 | 52.10 | 115.20 | 0.00 |
|  | PHCS3 | 800 | 2 | 5 | 0.416 | 0.976 | 46.27 | - | - | 0.0893 | 1 M NaPF_6_ in diglyme | 100 | 58.10 | 206.30 | 29.01 |
| 44 | BPHC | 1000 | 4 | - | 0.373 | 2.700 | 49.60 | 1.810 | 1.430 | - | 1 M NaClO_4_ in EC: PC (1:1) with 5% FEC | 100 | 64.00 | 189.28 | 42.71 |
| 45 | CCS-15 | 1400 | 3 | 5 | 0.418 | 1.480 | 3.63 | - | 3.200 | 0.0063 | 1 M NaPF_6_ in DME | 30 | 72.70 | 238.10 | 120.00 |
|  | CCS-30 | 1400 | 3 | 5 | 0.420 | 1.740 | 1.47 | - | 2.980 | 0.0051 | 1 M NaPF_6_ in DME | 30 | 83.40 | 332.50 | 230.10 |
|  | CCS-45 | 1400 | 3 | 5 | 0.419 | 1.430 | 2.93 | - | 3.120 | 0.0055 | 1 M NaPF_6_ in DME | 30 | 78.90 | 260.80 | 168.00 |
|  | CCS-60 | 1400 | 3 | 5 | 0.417 | 1.400 | 4.34 | - | 3.170 | 0.0078 | 1 M NaPF_6_ in DME | 30 | 77.10 | 251.90 | 130.00 |
| 46 | WFC1400 | 1400 | 3 | 3 | 0.374 | 1.175 | 52.10 | - | - | 0.0611 | 1 M NaPF_6_ in EC: DMC (1:1) | 20 | 81.10 | 273.70 | 188.87 |
|  | WFOC250-1400 | 1400 | 3 | 3 | 0.382 | 1.180 | 15.30 | - | - | 0.0331 | 1 M NaPF_6_ in EC: DMC (1:1) | 20 | 90.10 | 308.00 | 216.19 |
|  | WFOC280-1400 | 1400 | 3 | 3 | 0.398 | 1.198 | 10.80 | - | - | 0.0238 | 1 M NaPF_6_ in EC: DMC (1:1) | 20 | 89.40 | 290.10 | 200.40 |
| 47 | PNDCs | 900 | 2 | 5 | 0.375 | 2.210 | 195.70 | - | - | 0.1393 | 1 M NaClO_4_ in PC with 5% FEC | 100 | 54.70 | 147.40 | 19.88 |
|  | PNDCs-500CDs | 900 | 2 | 5 | 0.392 | 2.890 | 181.30 | - | - | 0.1409 | 1 M NaClO_4_ in PC with 5% FEC | 100 | 62.30 | 207.15 | 60.09 |
|  | PNDCs-500CDs1200 °C | 1200 | 2 | 5 | 0.382 | 2.280 | 17.40 | - | - | 0.0828 | 1 M NaClO_4_ in PC with 5% FEC | 100 | 68.40 | 219.22 | 76.08 |
| 48 | WFC-1800 | 1800 | 2 | - | 0.365 | 0.750 | 13.00 | - | - | - | 1 M NaPF_6_ in DME | 20 | 80.50 | 214.70 | 89.50 |
| 49 | HCF-1200 | 1200 | 2 | - | 0.393 | 1.090 | 5.00 | - | - | - | 1 M NaPF_6_ in diglyme | 20 | 76.00 | 213.19 | 107.81 |
|  | HCF-1400 | 1400 | 2 | - | 0.371 | 0.940 | 2.00 | - | - | - | 1 M NaPF_6_ in diglyme | 20 | 89.00 | 261.08 | 174.73 |
| 50 | SC-pre-800 | 800 | 2 | 5 | 0.390 | 3.110 | 252.30 | - | - | - | 1 M NaClO_4_ in EC: DMC: EMC (1:1:1) with 2% FEC | 30 | 61.50 | 201.80 | 50.00 |
|  | SC-1000 | 1000 | 2 | 5 | 0.385 | 2.940 | 84.60 | - | - | - | 1 M NaClO_4_ in EC: DMC: EMC (1:1:1) with 2% FEC | 30 | 75.70 | 246.40 | 112.00 |
|  | SC-1200 | 1200 | 2 | 5 | 0.383 | 2.350 | 6.70 | - | - | - | 1 M NaClO_4_ in EC: DMC: EMC (1:1:1) with 2% FEC | 30 | 75.50 | 265.40 | 158.00 |
|  | SC-1400 | 1400 | 2 | 5 | 0.379 | 1.980 | 3.30 | - | - | - | 1 M NaClO_4_ in EC: DMC: EMC (1:1:1) with 2% FEC | 30 | 83.40 | 309.20 | 219.00 |
|  | SC-1600 | 1600 | 2 | 5 | 0.371 | 1.800 | 1.90 | - | - | - | 1 M NaClO_4_ in EC: DMC: EMC (1:1:1) with 2% FEC | 30 | 83.80 | 287.7 | 236.00 |
| 51 | e-TSC8 | 800 | - | 5 | 0.368 | 1.084 | 239.89 | 4.114 | 1.257 | 0.0680 | 1 M NaPF_6_ in DEGDME | 10 | 48.10 | 161.36 | 64.08 |
|  | e-TSC20 | 2000 | - | 5 | 0.343 | 1.306 | 17.70 | 12.818 | 7.159 | 0.0060 | 1 M NaPF_6_ in DEGDME | 10 | 62.90 | 178.25 | 120.58 |
|  | e-ASC8 | 800 | - | 5 | 0.376 | 1.102 | 138.47 | 3.899 | 0.936 | 0.0400 | 1 M NaPF_6_ in DEGDME | 10 | 52.70 | 192.82 | 48.93 |
| 52 | PB-1000 | 1000 | 2 | - | 0.354 | 0.940 | 131.24 | 2.140 | 0.680 | - | 1 M NaPF_6_ in DME | 100 | 57.60 | 255.93 | 78.42 |
| 53 | (N, S)-C1190 | 1190 | 2 | 5 | 0.376 | 0.960 | 296.17 | - | - | - | 1 M NaPF_6_ in diglyme | 50 | 65.96 | 327.59 | 0.00 |
| 54 | LP5-1000 | 1000 | 2 | - | 0.402 | 1.180 | 325.00 | - | - | - | 1 M NaPF_6_ in DEGDME | 30 | 66.00 | 181.00 | 53.65 |
|  | LP5-1200 | 1200 | 2 | - | 0.397 | 1.150 | 103.00 | - | - | - | 1 M NaPF_6_ in DEGDME | 30 | 74.00 | 232.00 | 109.66 |
|  | LP3-1400 | 1400 | 2 | - | 0.390 | 1.160 | 46.00 | - | - | - | 1 M NaPF_6_ in DEGDME | 30 | 80.00 | 303.00 | 172.61 |
|  | LP7-1400 | 1400 | 2 | - | 0.388 | 1.050 | 29.00 | - | - | - | 1 M NaPF_6_ in DEGDME | 30 | 76.00 | 260.00 | 120.08 |
| 55 | HC-1100 | 1100 | 5 | 5 | 0.411 | 1.664 | 15.61 | - | - | - | 1 M NaClO_4_ in EC: DEC (1: 1) | 100 | 57.10 | 121.75 | 25.00 |
| 56 | CM-140 | 1200 | 2 | 5 | 0.367 | 1.850 | 115.90 | - | - | 0.1420 | 1 M NaClO_4_ in EC: DEC (1: 1) | 30 | 68.20 | 265.80 | 162.10 |
|  | CM-160 | 1200 | 2 | 5 | 0.379 | 1.890 | 70.20 | - | - | 0.0540 | 1 M NaClO_4_ in EC: DEC (1: 1) | 30 | 74.10 | 294.30 | 200.40 |
|  | CM-180 | 1200 | 2 | 5 | 0.384 | 1.960 | 30.00 | - | - | 0.0120 | 1 M NaClO_4_ in EC: DEC (1: 1) | 30 | 78.40 | 327.40 | 230.50 |
|  | CM-200 | 1200 | 2 | 5 | 0.401 | 2.200 | 47.30 | - | - | 0.0300 | 1 M NaClO_4_ in EC: DEC (1: 1) | 30 | 74.30 | 287.80 | 188.50 |
| 57 | HC | 1400 | 2 | - | 0.372 | 1.630 | 22.55 | 3.820 | 1.830 | 0.0160 | 1 M NaClO_4_ in EC: DMC (1: 1) | 20 | 76.62 | 276.00 | 92.40 |
|  | HCH-600 | 1400 | 2 | - | 0.377 | 1.510 | 27.99 | 3.760 | 1.710 | 0.0220 | 1 M NaClO_4_ in EC: DMC (1: 1) | 20 | 80.67 | 325.00 | 186.50 |
|  | HCH-800 | 1400 | 2 | - | 0.379 | 1.590 | 37.20 | 3.330 | 1.670 | 0.0280 | 1 M NaClO_4_ in EC: DMC (1: 1) | 20 | 77.75 | 300.80 | 150.00 |
|  | HCH-1000 | 1400 | 2 | - | 0.381 | 1.710 | 51.99 | 3.160 | 1.370 | 0.0310 | 1 M NaClO_4_ in EC: DMC (1: 1) | 20 | 74.47 | 284.60 | 140.30 |
|  | HCH-1200 | 1400 | 2 | - | 0.378 | 1.810 | 78.46 | 3.230 | 1.460 | 0.0520 | 1 M NaClO_4_ in EC: DMC (1: 1) | 20 | 76.39 | 264.10 | 95.82 |
| 58 | CHC-1100 | 1100 | 3 | 2 | 0.376 | 1.100 | 9.07 | 2.330 | 1.090 | - | 1 M NaPF_6_ in DME | 50 | 59.91 | 278.40 | 159.10 |
|  | CHC-1300 | 1300 | 3 | 2 | 0.365 | 1.030 | 4.73 | 2.840 | 1.140 | - | 1 M NaPF_6_ in DME | 50 | 70.59 | 317.40 | 189.10 |
| 59 | WPET-800 | 800 | 2 | 4 | 0.396 | 2.890 | 583.00 | - | - | 0.2920 | 1 M NaClO_4_ in EC: PC (1:1) | 30 | 45.00 | 254.00 | 0.00 |
|  | WPET-1000 | 1000 | 2 | 4 | 0.379 | 2.640 | 205.00 | - | - | 0.1860 | 1 M NaClO_4_ in EC: PC (1:1) | 30 | 66.00 | 337.00 | 153.60 |
| 60 | HC | 1300 | 2 | - | 0.380 | 1.040 | 90.75 | 1.600 | 1.420 | 0.0360 | 1 M NaPF_6_ in diglyme | 50 | 73.00 | 363.00 | 151.70 |
| 61 | HCS-1000 | 1000 | 2 | - | 0.393 | 2.090 | 329.00 | - | - | 0.1540 | 1 M NaPF_6_ in diglyme | 50 | 65.40 | 245.40 | 107.89 |
|  | G@H-1000 | 1000 | 2 | - | 0.389 | 2.030 | 478.40 | - | - | 0.2180 | 1 M NaPF_6_ in diglyme | 50 | 55.80 | 158.40 | 27.89 |
|  | GH5-1000 | 1000 | 2 | - | 0.364 | 2.010 | 13.30 | - | - | 0.0050 | 1 M NaPF_6_ in diglyme | 50 | 70.30 | 302.50 | 108.00 |
| 62 | MPC | 800 | 2 | 5 | 0.380 | 0.630 | 1079.88 | - | 1.220 | 2.3172 | 1 M NaClO_4_ in EC: DEC (1:1) | 100 | 40.05 | 338.00 | 0.00 |
| 63 | HC | 900 | 5 | - | 0.399 | 0.890 | 578.20 | 4.700 | 2.000 | - | 1 M NaClO_4_ in EC: PC (1:1) with 5% FEC | 50 | 36.29 | 187.23 | 0.00 |
|  | O-HC | 900 | 5 | - | 0.399 | 0.950 | 352.70 | 4.500 | 1.600 | - | 1 M NaClO_4_ in EC: PC (1:1) with 5% FEC | 50 | 54.05 | 323.70 | 0.00 |
| 64 | NCC1000 | 1000 | 1 | 5 | 0.382 | 1.370 | 38.00 | - | - | - | 1 M NaClO_4_ in EC: DMC (1:1) | 30 | 73.00 | 256.00 | 141.00 |
|  | HTCNCC1500 | 1500 | 1 | 5 | 0.373 | 1.520 | 13.00 | - | - | - | 1 M NaClO_4_ in EC: DMC (1:1) | 30 | 90.00 | 314.00 | 239.00 |
| 65 | L-HC | 1400 | - | 5 | 0.382 | 2.300 | 161.20 | 2.105 | 1.329 | 0.0121 | 1 M NaPF_6_ in diglyme | 100 | 64.00 | 231.00 | 128.90 |
|  | CL-HC | 1400 | - | 5 | 0.384 | 2.200 | 53.00 | 2.055 | 1.281 | 0.0177 | 1 M NaPF_6_ in diglyme | 100 | 69.00 | 259.00 | 160.10 |
|  | HL-HC | 1400 | - | 5 | 0.373 | 1.930 | 64.20 | 2.053 | 1.409 | 0.1107 | 1 M NaPF_6_ in diglyme | 100 | 65.00 | 236.00 | 108.60 |
|  | CHL-HC | 1400 | - | 5 | 0.383 | 2.440 | 30.40 | 2.147 | 1.296 | 0.0493 | 1 M NaPF_6_ in diglyme | 100 | 70.00 | 240.00 | 150.72 |
| 66 | Glu | 1500 | 1 | 10 | 0.372 | 1.150 | 24.00 | 4.260 | 1.150 | 0.0180 | 1 M NaClO_4_ in EC: DEC: PC (1:1:1) | 20 | 77.90 | 277.50 | 169.30 |
|  | Mg-Glu | 1500 | 1 | 10 | 0.371 | 1.240 | 82.00 | 4.280 | 1.040 | 0.1870 | 1 M NaClO_4_ in EC: DEC: PC (1:1:1) | 20 | 79.70 | 367.10 | 242.60 |
| 67 | FHC-1100 | 1100 | 2 | 5 | 0.393 | 2.300 | 187.50 | - | - | - | 1 M NaPF_6_ in diglyme | 100 | 72.70 | 203.20 | 107.40 |
|  | FHC-1500 | 1500 | 2 | 5 | 0.363 | 1.720 | 46.60 | - | - | - | 1 M NaPF_6_ in diglyme | 100 | 71.80 | 144.00 | 114.00 |
| 68 | ENMC | 600 | 2 | 5 | 0.377 | 0.880 | 690.00 | - | - | 0.0950 | 1 M NaClO_4_ in EC: DEC (1:1) with 5% FEC | 100 | 49.60 | 276.40 | 0.00 |
|  | BNPC | 600 | 2 | 5 | 0.363 | 0.840 | 468.00 | - | - | 0.0920 | 1 M NaClO_4_ in EC: DEC (1:1) with 5% FEC | 100 | 44.20 | 251.70 | 0.00 |
| 69 | G-160-1300 | 1300 | 1 | - | 0.364 | 1.900 | 105.00 | - | - | - | 1 M NaPF_6_ in EC: DEC (1:1) | 25 | 72.00 | 258.00 | 159.17 |
|  | G-180-1300 | 1300 | 1 | - | 0.360 | 1.900 | 2.50 | - | - | - | 1 M NaPF_6_ in EC: DEC (1:1) | 25 | 85.00 | 293.00 | 197.23 |
|  | G-200-1300 | 1300 | 1 | - | 0.362 | 1.800 | 0.10 | - | - | - | 1 M NaPF_6_ in EC: DEC (1:1) | 25 | 89.00 | 300.00 | 204.15 |
|  | G-220-1300 | 1300 | 1 | - | 0.364 | 1.800 | 6.00 | - | - | - | 1 M NaPF_6_ in EC: DEC (1:1) | 25 | 84.00 | 308.00 | 209.69 |
|  | G-240-1300 | 1300 | 1 | - | 0.362 | 1.900 | 8.00 | - | - | - | 1 M NaPF_6_ in EC: DEC (1:1) | 25 | 75.00 | 282.00 | 186.85 |
| 70 | HC-900 | 900 | 2 | - | 0.387 | 1.240 | 3.00 | - | - | - | 1 M NaPF_6_ in EC: DEC (1:1) | 20 | 68.00 | 278.00 | 129.00 |
|  | HC-1100 | 1100 | 2 | - | 0.381 | 1.180 | 3.80 | - | - | - | 1 M NaPF_6_ in EC: DEC (1:1) | 20 | 70.00 | 250.00 | 137.00 |
|  | HC-1300 | 1300 | 2 | - | 0.375 | 1.110 | 1.42 | - | - | - | 1 M NaPF_6_ in EC: DEC (1:1) | 20 | 75.00 | 237.00 | 143.00 |
| 71 | JFC900S1.5 | 900 | 3 | 2 | 0.367 | 1.005 | 556.96 | - | - | - | 1 M NaClO_4_ in EC: DEC (1:1) | 100 | 37.68 | 233.33 | 0.00 |
| 72 | SCNs-850 | 850 | 2 | 2 | 0.391 | 1.380 | 137.83 | - | - | 0.3200 | 1 M NaClO_4_ in EC: DEC (1:1) | 50 | 55.90 | 517.30 | 0.00 |
|  | SCNs-950 | 950 | 2 | 2 | 0.370 | 1.280 | 222.89 | - | - | 0.3700 | 1 M NaClO_4_ in EC: DEC (1:1) | 50 | 51.80 | 372.00 | 0.00 |
| 73 | NPWSC-700 | 700 | 2 | 5 | 0.424 | 0.950 | 1271.00 | - | - | 1.1000 | 1 M NaClO_4_ in EC: DMC: EMC (1:1:1) with 2% FEC | 74.4 | 24.70 | 374.00 | 0.00 |
| 74 | HCM-800 | 800 | 4 | - | 0.374 | 2.090 | 494.62 | 1.531 | 0.705 | 0.2339 | 1 M NaPF_6_ in diglyme | 50 | 39.87 | 223.20 | 0.00 |
|  | HCM-900 | 900 | 4 | - | 0.371 | 2.000 | 445.91 | 1.960 | 0.737 | 0.2086 | 1 M NaPF_6_ in diglyme | 50 | 51.44 | 234.10 | 54.86 |
|  | HCM-1000 | 1000 | 4 | - | 0.377 | 1.950 | 435.32 | 2.163 | 0.745 | 0.1336 | 1 M NaPF_6_ in diglyme | 50 | 61.34 | 323.70 | 129.81 |
|  | HCM-1300 | 1300 | 4 | - | 0.378 | 1.760 | 22.98 | 2.271 | 0.842 | 0.0083 | 1 M NaPF_6_ in diglyme | 50 | 71.70 | 376.50 | 171.26 |
|  | HCM-1300-  ZBE | 1300 | 4 | - | 0.391 | 1.720 | 48.91 | 2.306 | 1.028 | 0.0178 | 1 M NaPF_6_ in diglyme | 50 | 77.63 | 500.80 | 116.40 |
|  | HCM-1500-  ZBE | 1500 | 4 | - | 0.379 | 1.700 | 58.41 | 2.457 | 1.149 | 0.0094 | 1 M NaPF_6_ in diglyme | 50 | 72.20 | 391.80 | 232.48 |
| 75 | CNS-600 | 600 | 2 | 5 | 0.386 | 1.360 | 394.00 | - | 1.300 | - | 1 M NaPF_6_ in DME | 100 | 49.00 | 201.00 | 0.00 |
|  | CNS-1000 | 1000 | 2 | 5 | 0.374 | 1.320 | 356.00 | - | 1.370 | - | 1 M NaPF_6_ in DME | 100 | 67.00 | 351.00 | 42.60 |
|  | CNS-1300 | 1300 | 2 | 5 | 0.368 | 1.280 | 41.00 | - | 1.390 | - | 1 M NaPF_6_ in DME | 100 | 69.00 | 435.00 | 159.26 |
|  | PF-1300 | 1300 | 2 | 5 | 0.363 | 1.250 | 28.00 | - | 1.440 | - | 1 M NaPF_6_ in DME | 100 | 69.00 | 346.00 | 145.05 |
| 76 | HC-700 | 700 | 2 | 5 | 0.403 | 2.100 | 352.06 | - | - | - | 1 M NaClO_4_ in EC: DMC (1:1) | 30 | 56.50 | 116.24 | 0.00 |
|  | HC-900 | 900 | 2 | 5 | 0.399 | 2.010 | 131.80 | - | - | - | 1 M NaClO_4_ in EC: DMC (1:1) | 30 | 67.70 | 131.65 | 35.06 |
|  | HC-1100 | 1100 | 2 | 5 | 0.394 | 1.770 | 18.74 | - | - | - | 1 M NaClO_4_ in EC: DMC (1:1) | 30 | 81.60 | 226.96 | 128.45 |
|  | HC-1400 | 1400 | 2 | 5 | 0.386 | 1.540 | 6.64 | - | - | - | 1 M NaClO_4_ in EC: DMC (1:1) | 30 | 89.10 | 299.61 | 188.79 |
| 77 | NOCS | 800 | 2 | - | 0.375 | 1.760 | 211.00 | - | - | 0.0900 | 1 M NaSO_3_CF_3_ in diglyme | 50 | 61.40 | 314.00 | 0.00 |
| 78 | NP-PCN | 800 | 2 | 3 | 0.470 | 1.080 | 435.34 | - | - | - | 1 M NaClO_4_ in DMC: EC (1:1) with 5% FEC | 100 | 38.50 | 262.00 | 0.00 |
| 79 | GHC-80 | 1400 | 1 | - | 0.377 | 1.956 | 1.82 | 4.930 | 1.50 | - | 1 M NaPF_6_ in EC: DMC (1:1) | 30 | 90.40 | 343.00 | 240.14 |
|  | GHC-200 | 1400 | 1 | - | 0.388 | 2.132 | 5.65 | 4.740 | 1.340 | - | 1 M NaPF_6_ in EC: DMC (1:1) | 30 | 80.10 | 292.00 | 208.65 |
|  | GHC-150 | 1400 | 1 | - | 0.380 | 2.058 | 4.78 | 4.810 | 1.420 | - | 1 M NaPF_6_ in EC: DMC (1:1) | 30 | 84.00 | 313.00 | 221.80 |
|  | GHC-50 | 1400 | 1 | - | 0.370 | 1.925 | 4.07 | 5.020 | 1.690 | - | 1 M NaPF_6_ in EC: DMC (1:1) | 30 | 86.20 | 335.00 | 240.00 |
| 80 | SCNs | 1000 | 2 | 0.5 | 0.377 | 1.570 | 273.20 | - | - | 0.3500 | 1 M NaClO_4_ in EC: DEC (1:1) with 5% FEC | 20 | 55.10 | 314.90 | 105.01 |
| 81 | WYM-1100 | 1100 | 3 | 5 | 0.374 | 2.260 | 48.19 | - | - | - | 1 M NaClO_4_ in EC: DEC (1:1) with 5% FEC | 100 | 69.80 | 210.60 | 53.28 |
| 82 | JPC-D | 800 | 2 | - | 0.379 | 3.380 | 210.90 | - | - | 0.1280 | 1 M NaPF_6_ in EC: DEC (1:1) | 30 | 66.00 | 328.00 | 81.04 |
|  | JPC-M | 800 | 2 | - | 0.367 | 3.570 | 516.00 | - | - | 0.2480 | 1 M NaPF_6_ in EC: DEC (1:1) | 30 | 52.00 | 283.00 | 0.00 |
| 83 | LCS-37 | 1200 | 2 | 5 | 0.388 | 1.905 | 2.49 | - | - | 0.0084 | 1 M NaClO_4_ in EC: DEC (1:1) with 5% FEC | 30 | 80.00 | 272.00 | 123.22 |
|  | LCS-55 | 1200 | 2 | 5 | 0.382 | 1.869 | 1.09 | - | - | 0.0048 | 1 M NaClO_4_ in EC: DEC (1:1) with 5% FEC | 30 | 81.80 | 328.00 | 174.17 |
|  | LCS-73 | 1200 | 2 | 5 | 0.377 | 1.848 | 1.48 | - | - | 0.0053 | 1 M NaClO_4_ in EC: DEC (1:1) with 5% FEC | 30 | 82.90 | 356.00 | 196.16 |
|  | LCS-82 | 1200 | 2 | 5 | 0.361 | 1.631 | 4.93 | - | - | 0.0126 | 1 M NaClO_4_ in EC: DEC (1:1) with 5% FEC | 30 | 76.40 | 317.00 | 167.69 |
|  | LC | 1200 | 2 | 5 | 0.345 | 1.592 | 169.52 | - | - | 0.2367 | 1 M NaClO_4_ in EC: DEC (1:1) with 5% FEC | 30 | 59.90 | 290.00 | 96.86 |
| 84 | MHCS-600 | 600 | 2 | 5 | 0.382 | 2.740 | 301.00 | - | 1.262 | 0.2960 | 1 M NaPF_6_ in DME | 100 | 60.42 | 132.00 | 0.00 |
|  | MHCS-1300 | 1300 | 2 | 5 | 0.370 | 1.620 | 101.00 | - | 1.311 | 0.1400 | 1 M NaPF_6_ in DME | 100 | 66.55 | 230.00 | 91.15 |
| 85 | H1000 | 1000 | 3 | - | 0.379 | 2.040 | 73.38 | - | - | - | 1 M NaCF_3_SO_3_ in DIGLYME | 50 | 62.42 | 225.37 | 99.79 |
|  | H1100 | 1100 | 3 | - | 0.376 | 1.790 | 64.15 | - | - | - | 1 M NaCF_3_SO_3_ in DIGLYME | 50 | 69.08 | 252.88 | 123.35 |
| 86 | NP-CNFs-1.0 | 800 | 2 | - | 0.385 | 1.110 | 12.98 | - | - | 0.0220 | 1 M NaClO_4_ in EC: DMC: EMC (1:1:1) with 5% FEC | 50 | 66.00 | 295.40 | 51.30 |
|  | N-CNFs | 800 | 2 | - | 0.361 | 1.010 | 12.41 | - | - | 0.0220 | 1 M NaClO_4_ in EC: DMC: EMC (1:1:1) with 5% FEC | 50 | 65.30 | 194.30 | 63.40 |
| 87 | GLC-700 | 700 | - | - | 0.480 | 0.950 | 498.23 | - | - | - | 1 M NaClO_4_ in EC: PC (1:1) | 20 | 35.80 | 222.56 | 0.00 |
| 88 | HC-900 | 900 | 3 | 2 | 0.399 | 1.790 | 645.00 | - | - | 0.6100 | 1 M NaClO_4_ in EC: DMC (1:1) with 5% NaNO_3_ | 100 | 42.80 | 132.90 | 45.50 |
|  | HC-1100 | 1100 | 3 | 2 | 0.381 | 1.640 | 547.00 | - | - | 0.1400 | 1 M NaClO_4_ in EC: DMC (1:1) with 5% NaNO_3_ | 100 | 47.50 | 195.2 | 90.10 |
| 89 | CSDHC | 1000 | 2 | - | 0.372 | 1.040 | 40.00 | - | - | 0.0680 | 1 M NaClO_4_ in EC: DEC (1:1) | 100 | 64.00 | 265.6 | 94.37 |
|  | CSDHC 280/15 | 1000 | 2 | - | 0.364 | 1.080 | 60.00 | - | - | 0.0775 | 1 M NaClO_4_ in EC: DEC (1:1) | 100 | 54.00 | 225.72 | 45.89 |
|  | CSDHC 300/30 | 1000 | 2 | - | 0.360 | 1.060 | 655.00 | - | - | 0.4240 | 1 M NaClO_4_ in EC: DEC (1:1) | 100 | 37.00 | 184.63 | 0.00 |
| 90 | P-HCB | 700 | 2 | 5 | 0.423 | 0.970 | 356.40 | - | - | - | 1 M NaPF_6_ in DME | 200 | 71.85 | - | 0.00 |
| 91 | HC-1100 | 1100 | 2 | 10 | 0.387 | 2.220 | 35.63 | 1.255 | 0.798 | 0.0330 | 1 M NaPF_6_ in DEG: DME (1:1) | 30 | 72.70 | 266.10 | 140.53 |
|  | HC-1300 | 1300 | 2 | 10 | 0.385 | 2.200 | 13.04 | 1.188 | 0.852 | 0.0170 | 1 M NaPF_6_ in DEG: DME (1:1) | 30 | 84.10 | 348.50 | 227.27 |
|  | HC-1500 | 1500 | 2 | 10 | 0.371 | 2.040 | 3.76 | 1.806 | 0.983 | 0.0170 | 1 M NaPF_6_ in DEG: DME (1:1) | 30 | 85.60 | 310.00 | 239.39 |
|  | HC-1700 | 1700 | 2 | 10 | 0.370 | 1.970 | 3.69 | 1.701 | 1.027 | 0.0140 | 1 M NaPF_6_ in DEG: DME (1:1) | 30 | 87.60 | 314.50 | 240.00 |
| 92 | PDD0-15 | 1500 | 2 | - | 0.354 | 1.080 | 75.11 | 5.070 | 1.500 | - | 1 M NaPF_6_ in EC: DMC (1:1) | 20 | 79.31 | 201.00 | 100.89 |
|  | PDD2-15 | 1500 | 2 | - | 0.360 | 1.100 | 71.12 | 4.710 | 1.370 | - | 1 M NaPF_6_ in EC: DMC (1:1) | 20 | 78.95 | 246.00 | 132.37 |
|  | PDD4-15 | 1500 | 2 | - | 0.365 | 1.130 | 60.83 | 4.040 | 1.280 | - | 1 M NaPF_6_ in EC: DMC (1:1) | 20 | 80.92 | 253.00 | 135.03 |
|  | PDD6-15 | 1500 | 2 | - | 0.372 | 1.180 | 61.15 | 3.790 | 1.250 | - | 1 M NaPF_6_ in EC: DMC (1:1) | 20 | 77.79 | 276.00 | 152.34 |
|  | PDD8-15 | 1500 | 2 | - | 0.373 | 1.220 | 59.40 | 3.470 | 1.230 | - | 1 M NaPF_6_ in EC: DMC (1:1) | 20 | 80.19 | 281.00 | 163.03 |
|  | PDD10-15 | 1500 | 2 | - | 0.375 | 1.270 | 77.02 | 3.250 | 1.150 | - | 1 M NaPF_6_ in EC: DMC (1:1) | 20 | 80.61 | 375.00 | 202.67 |
| 93 | MLC-1000 | 1000 | 2 | 3 | 0.410 | 4.000 | 11.20 | 3.250 | 0.960 | - | 1 M NaPF_6_ in EC: DEC: DMC (1:1:1) | 20 | 72.30 | 216.40 | 81.96 |
|  | MLC-1200 | 1200 | 2 | 3 | 0.396 | 3.700 | 19.70 | 3.670 | 1.010 | - | 1 M NaPF_6_ in EC: DEC: DMC (1:1:1) | 20 | 72.80 | 281.60 | 143.53 |
|  | MLC-1400 | 1400 | 2 | 3 | 0.392 | 2.560 | 10.50 | 4.040 | 1.160 | - | 1 M NaPF_6_ in EC: DEC: DMC (1:1:1) | 20 | 80.10 | 292.60 | 178.82 |
|  | MLC-1600 | 1600 | 2 | 3 | 0.346 | 2.380 | 10.30 | 4.370 | 1.620 | - | 1 M NaPF_6_ in EC: DEC: DMC (1:1:1) | 20 | 79.50 | 290.70 | 184.31 |
|  | MCC-1200 | 1200 | 2 | 3 | 0.392 | 2.220 | 103.10 | 4.030 | 1.050 | - | 1 M NaPF_6_ in EC: DEC: DMC (1:1:1) | 20 | 68.10 | 239.5 | 120.63 |
|  | MCC-1400 | 1400 | 2 | 3 | 0.388 | 1.960 | 5.80 | 4.450 | 1.090 | - | 1 M NaPF_6_ in EC: DEC: DMC (1:1:1) | 20 | 87.30 | 343.30 | 216.50 |
|  | MCC-1600 | 1600 | 2 | 3 | 0.351 | 1.850 | 4.00 | 4.780 | 1.460 | - | 1 M NaPF_6_ in EC: DEC: DMC (1:1:1) | 20 | 80.60 | 256.60 | 163.06 |
| 94 | PO-SC-19 | 800 | - | - | 0.405 | 0.970 | 39.40 | - | - | - | 1 M NaPF_6_ in EC: DMC (1:1) | 30 | 60.00 | 267.00 | 0.00 |
|  | PO-SC-S-1000 | 1000 | - | - | 0.358 | 0.980 | 9.50 | - | - | - | 1 M NaPF_6_ in EC: DMC (1:1) | 30 | 75.00 | 173.00 | 0.00 |
| 95 | C1300 | 1300 | 6 | - | 0.379 | 1.280 | 29.30 | - | - | 0.0400 | 1 M NaPF_6_ in DEGDME | 10 | 84.30 | 274.20 | 176.64 |
| 96 | NC | 900 | 3 | 3 | 0.361 | 0.830 | 10.42 | - | - | 0.0140 | 1 M NaPF_6_ in DEGDME | 20 | 64.09 | 192.43 | 36.88 |
|  | 2BNC | 900 | 3 | 3 | 0.362 | 0.850 | 12.30 | - | - | 0.0320 | 1 M NaPF_6_ in DEGDME | 20 | 68.16 | 231.83 | 65.56 |
|  | 5BNC | 900 | 3 | 3 | 0.363 | 0.870 | 12.29 | - | - | 0.0240 | 1 M NaPF_6_ in DEGDME | 20 | 66.90 | 256.90 | 75.00 |
|  | 10BNC | 900 | 3 | 3 | 0.364 | 0.880 | 8.20 | - | - | 0.0140 | 1 M NaPF_6_ in DEGDME | 20 | 69.51 | 242.55 | 76.60 |
| 97 | PHC800 | 800 | 2 | - | 0.387 | 2.040 | 136.00 | 3.390 | 1.150 | 0.2260 | 1 M NaPF_6_ in EC: DEC (1:1) | 50 | 59.88 | 211.40 | 53.72 |
|  | PHC1000 | 1000 | 2 | - | 0.385 | 2.010 | 120.70 | 3.250 | 1.180 | 0.2100 | 1 M NaPF_6_ in EC: DEC (1:1) | 50 | 64.08 | 237.60 | 85.20 |
|  | PHC1200 | 1200 | 2 | - | 0.386 | 1.960 | 4.90 | 3.730 | 1.200 | 0.0160 | 1 M NaPF_6_ in EC: DEC (1:1) | 50 | 66.77 | 251.19 | 121.43 |
|  | PHC1400 | 1400 | 2 | - | 0.366 | 1.910 | 4.10 | 4.240 | 1.250 | 0.0140 | 1 M NaPF_6_ in EC: DEC (1:1) | 50 | 76.45 | 258.14 | 137.44 |
|  | PHC1600 | 1600 | 2 | - | 0.343 | 1.890 | 4.10 | 6.220 | 5.210 | 0.0100 | 1 M NaPF_6_ in EC: DEC (1:1) | 50 | 72.77 | 248.00 | 150.00 |
|  | PSC800 | 800 | 2 | - | 0.347 | 1.660 | 8.40 | 4.490 | 1.580 | 0.0150 | 1 M NaPF_6_ in EC: DEC (1:1) | 50 | 66.03 | 193.95 | 0.00 |
|  | PSC1000 | 1000 | 2 | - | 0.348 | 1.390 | 3.50 | 5.360 | 1.760 | 0.0050 | 1 M NaPF_6_ in EC: DEC (1:1) | 50 | 61.57 | 141.44 | 0.00 |
| 98 | O-CNFs | 650 | 2 | 3 | 0.370 | 1.040 | 5.74 | - | - | 0.0102 | 1 M NaClO_4_ in DMC: EMC (1:1) | 100 | 46.57 | 181.50 | 0.00 |
|  | O/S-CNFs | 650 | 2 | 3 | 0.380 | 1.060 | 10.86 | - | - | 0.0141 | 1 M NaClO_4_ in DMC: EMC (1:1) | 100 | 52.80 | 355.40 | 0.00 |
| 99 | HCK-15 | 1200 | 2 | - | 0.410 | 1.040 | 221.59 | - | - | 0.0760 | 1 M NaClO_4_ in PC with 5% FEC | 50 | 65.00 | 305.00 | 179.00 |
| 100 | DG1000 | 1000 | 2 | 5 | 0.388 | 1.050 | 85.10 | 1.290 | - | 0.0480 | 1 M NaClO_4_ in EC: DMC (1:1) | 30 | 60.54 | 197.43 | 67.89 |
|  | HG1300 | 1300 | 2 | 5 | 0.385 | 1.080 | 34.40 | 1.310 | - | 0.0240 | 1 M NaClO_4_ in EC: DMC (1:1) | 30 | 85.00 | 347.40 | 180.00 |
|  | DG1300 | 1300 | 2 | 5 | 0.378 | 1.120 | 28.00 | 1.340 | - | 0.0160 | 1 M NaClO_4_ in EC: DMC (1:1) | 30 | 85.00 | 261.30 | 146.00 |
|  | HG1500 | 1500 | 2 | 5 | 0.377 | 1.120 | 26.10 | 1.340 | - | 0.0190 | 1 M NaClO_4_ in EC: DMC (1:1) | 30 | 82.60 | 320.75 | 212.25 |
|  | DG1500 | 1500 | 2 | 5 | 0.357 | 1.390 | 22.60 | 1.490 | - | 0.0140 | 1 M NaClO_4_ in EC: DMC (1:1) | 30 | 72.17 | 222.92 | 118.58 |
| 101 | H200–1100 | 1100 | 3 | 2 | 0.398 | 2.590 | 22.90 | - | - | 0.0140 | 1 M NaClO_4_ in EC: DEC (1:1) | 20 | 80.50 | 295.70 | 155.86 |
|  | H300–1100 | 1100 | 3 | 2 | 0.387 | 2.410 | 2.96 | - | - | 0.0040 | 1 M NaClO_4_ in EC: DEC (1:1) | 20 | 82.50 | 369.80 | 200.00 |
|  | H400–1100 | 1100 | 3 | 2 | 0.390 | 2.690 | 54.13 | - | - | 0.0340 | 1 M NaClO_4_ in EC: DEC (1:1) | 20 | 77.70 | 330.70 | 168.28 |
|  | A300–1100 | 1100 | 3 | 2 | 0.394 | 2.440 | 5.26 | - | - | 0.0070 | 1 M NaClO_4_ in EC: DEC (1:1) | 20 | 80.10 | 346.70 | 180.69 |
| 102 | HC-MC | 1500 | 1 | 5 | 0.361 | 1.430 | 1.50 | 4.100 | 1.100 | 0.0039 | 1 M NaPF_6_ in EC: DMC (1:1) with 2% FEC | 37.2 | 85.40 | 294.70 | 203.38 |
|  | HC-MG | 1500 | 1 | 5 | 0.367 | 1.490 | 1.30 | 4.100 | 1.000 | 0.0036 | 1 M NaPF_6_ in EC: DMC (1:1) with 2% FEC | 37.2 | 85.50 | 299.80 | 204.00 |
|  | HC-CH | 1500 | 1 | 5 | 0.357 | 1.360 | 2.20 | 4.100 | 1.200 | 0.0062 | 1 M NaPF_6_ in EC: DMC (1:1) with 2% FEC | 37.2 | 85.60 | 277.70 | 195.14 |
|  | HC-M | 1500 | 1 | 5 | 0.364 | 1.430 | 2.20 | 4.100 | 1.100 | 0.0056 | 1 M NaPF_6_ in EC: DMC (1:1) with 2% FEC | 37.2 | 84.80 | 304.30 | 219.81 |
| 103 | ordinary carbon | 1200 | 3 | - | 0.369 | 1.850 | 223.18 | - | - | 0.1200 | 1 M NaPF_6_ in diglyme | 100 | 64.10 | 256.00 | 140.58 |
| 104 | M9 | 900 | 1 | - | 0.373 | 0.960 | 62.30 | - | 1.200 | 0.0560 | 1 M NaPF_6_ in EC: DMC (1:1) | 20 | 65.32 | 231.13 | 89.74 |
|  | M11 | 1100 | 1 | - | 0.371 | 0.980 | 12.70 | - | 1.200 | 0.0200 | 1 M NaPF_6_ in EC: DMC (1:1) | 20 | 70.84 | 244.52 | 109.78 |
| 105 | SAC-750 | 750 | 4 | 3 | 0.374 | 0.970 | 1641.00 | - | - | 1.3100 | 1 M NaPF_6_ in DEGDME | 100 | 21.39 | 287.00 | 0.00 |
| 106 | PAN4-PEG4 | 1200 | 2 | 3 | 0.373 | 0.950 | 42.60 | - | - | 0.0330 | 1 M NaPF_6_ in DEGDME | 50 | 69.50 | 264.40 | 91.90 |
|  | PAN4-PEG8 | 1200 | 2 | 3 | 0.383 | 0.960 | 83.20 | - | - | 0.0500 | 1 M NaPF_6_ in DEGDME | 50 | 68.30 | 337.60 | 126.10 |
|  | PAN4-PEG12 | 1200 | 2 | 3 | 0.384 | 0.990 | 60.70 | - | - | 0.0450 | 1 M NaPF_6_ in DEGDME | 50 | 67.50 | 282.70 | 100.90 |
| 107 | NCM | 1100 | 2 | - | 0.377 | 1.340 | 10.90 | 3.540 | 1.560 | 0.0243 | 1 M NaClO_4_ in EC: DEC (1:1) | 25 | 75.30 | 217.30 | 89.96 |
|  | ONCM-55 | 1100 | 2 | - | 0.399 | 1.360 | 42.00 | 3.280 | 1.460 | 0.0384 | 1 M NaClO_4_ in EC: DEC (1:1) | 25 | 77.00 | 264.30 | 137.96 |
|  | ONCM-73 | 1100 | 2 | - | 0.402 | 1.370 | 57.00 | 3.350 | 1.450 | 0.0391 | 1 M NaClO_4_ in EC: DEC (1:1) | 25 | 63.70 | 237.70 | 125.51 |
| 108 | LS-800 | 800 | 2 | 5 | 0.360 | 0.700 | 40.00 | - | - | - | 1 M NaClO_4_ in EC: DEC (1:1) | 100 | 50.34 | 281.90 | 0.00 |
| 109 | PC-1200 | 1200 | 2 | 2 | 0.377 | 1.560 | 1.85 | 3.336 | 1.269 | - | 1 M NaPF_6_ in DMC: EC (1:1) | 20 | 80.60 | 282.00 | 171.62 |
|  | PC-1400 | 1400 | 2 | 2 | 0.375 | 1.460 | 1.77 | 3.928 | 1.347 | - | 1 M NaPF_6_ in DMC: EC (1:1) | 20 | 84.60 | 327.00 | 214.19 |
|  | PC-1600 | 1600 | 2 | 2 | 0.372 | 1.350 | 1.89 | 4.556 | 1.369 | - | 1 M NaPF_6_ in DMC: EC (1:1) | 20 | 84.30 | 288.00 | 200.00 |
|  | PET-1200 | 1200 | 2 | 2 | 0.381 | 1.420 | 1.00 | 3.758 | 1.210 | - | 1 M NaPF_6_ in DMC: EC (1:1) | 20 | 80.80 | 308.00 | 184.17 |
|  | PET-1400 | 1400 | 2 | 2 | 0.380 | 1.350 | 1.00 | 4.035 | 1.378 | - | 1 M NaPF_6_ in DMC: EC (1:1) | 20 | 86.10 | 342.00 | 230.37 |
|  | PET-1600 | 1600 | 2 | 2 | 0.363 | 1.010 | 2.66 | 4.490 | 1.421 | - | 1 M NaPF_6_ in DMC: EC (1:1) | 20 | 83.20 | 315.00 | 218.74 |
| 110 | PL-600 | 600 | 2 | 5 | 0.368 | 1.100 | 7.50 | - | - | - | 1 M NaPF_6_ in DEC: EC (1:1) | 50 | 53.31 | 434.50 | 0.00 |
| 111 | o-PDC-350-1400 | 1400 | 3 | 2 | 0.363 | 1.200 | 13.38 | 3.440 | 1.313 | - | 1 M NaClO_4_ in DEC: EC (1:1) | 100 | 73.38 | 276.80 | 162.88 |
| 112 | C-1:2 | 1300 | 2 | 5 | 0.352 | 1.600 | 9.90 | - | 1.768 | 0.0094 | 1 M NaPF_6_ in diglyme | 100 | 69.44 | 281.33 | 122.76 |
|  | C-1:4 | 1300 | 2 | 5 | 0.377 | 1.960 | 3.60 | - | 1.382 | 0.0020 | 1 M NaPF_6_ in diglyme | 100 | 71.50 | 315.43 | 150.86 |
|  | C-1:6 | 1300 | 2 | 5 | 0.376 | 2.180 | 9.00 | - | 1.392 | 0.0210 | 1 M NaPF_6_ in diglyme | 100 | 62.46 | 309.74 | 145.64 |
|  | C-1:10 | 1300 | 2 | 5 | 0.382 | 2.190 | 6.40 | - | 1.305 | 0.0098 | 1 M NaPF_6_ in diglyme | 100 | 73.44 | 308.83 | 145.87 |
| 113 | CMGL | 1200 | 2 | 5 | 0.390 | 1.090 | 95.70 | - | 1.240 | - | 1 M NaClO_4_ in PC: DEC (1:1) with 5% FEC | 30 | 69.40 | 315.00 | 98.25 |
| 114 | SHCs-1300 | 1300 | - | - | 0.373 | 1.243 | 3.67 | 5.176 | 1.387 | - | 0.6 M NaPF_6_ in EC: EMC (1:1) | 30 | 86.50 | 317.00 | 175.41 |
|  | SHCs-1500 | 1500 | - | - | 0.368 | 1.232 | 1.60 | 5.392 | 1.408 | - | 0.6 M NaPF_6_ in EC: EMC (1:1) | 30 | 90.50 | 305.00 | 150.81 |
| 115 | NPPC-2 | 900 | 2 | 5 | 0.405 | 1.866 | 821.88 | - | - | 0.6180 | 1 M NaClO_4_ in EC: DEC (1:1) with 2% FEC | 50 | 42.80 | 423.20 | 0.00 |
| 116 | PureL-1300 | 1300 | 2 | - | 0.375 | 1.290 | 4.90 | 3.820 | 1.560 | 0.0068 | 1 M NaClO_4_ in EC: DEC (1:1) | 25 | 70.10 | 340.50 | 237.02 |
|  | 0.05NPL-1300 | 1300 | 2 | - | 0.370 | 1.210 | 7.70 | 4.410 | 1.680 | 0.0080 | 1 M NaClO_4_ in EC: DEC (1:1) | 25 | 78.70 | 335.60 | 236.90 |
|  | 0.10NPL-1300 | 1300 | 2 | - | 0.364 | 1.180 | 10.60 | 5.360 | 1.790 | 0.0164 | 1 M NaClO_4_ in EC: DEC (1:1) | 25 | 82.40 | 306.90 | 207.18 |
|  | 0.20NPL-1300 | 1300 | 2 | - | 0.358 | 1.130 | 14.40 | 6.270 | 1.840 | 0.0271 | 1 M NaClO_4_ in EC: DEC (1:1) | 25 | 79.10 | 307.60 | 211.05 |
| 117 | CLP | 1200 | 2 | 5 | 0.390 | 1.080 | 33.86 | 4.750 | 1.350 | - | 1 M NaClO_4_ in EC: DEC (1:1) | 20 | 70.20 | 336.40 | 122.84 |
| 118 | HCMS 37 | 1400 | 2 | - | 0.387 | 1.980 | 5.68 | - | - | - | 1 M NaPF_6_ in EC: DMC (1:1) | 30 | 82.00 | 351.30 | 244.49 |
|  | HCMS 55 | 1400 | 2 | - | 0.397 | 2.130 | 6.87 | - | - | - | 1 M NaPF_6_ in EC: DMC (1:1) | 30 | 81.00 | 356.80 | 237.00 |
|  | HCMS 73 | 1400 | 2 | - | 0.399 | 2.250 | 2.32 | - | - | - | 1 M NaPF_6_ in EC: DMC (1:1) | 30 | 88.00 | 373.40 | 260.63 |
|  | HCMS-82-15 | 1400 | 2 | - | 0.401 | 2.300 | 4.62 | - | - | - | 1 M NaPF_6_ in EC: DMC (1:1) | 30 | 85.00 | 361.60 | 260.00 |
| 119 | BFHC-NC0 | 1400 | 2 | 5 | 0.355 | 1.350 | 4.08 | 3.530 | 1.010 | 0.0053 | 1 M NaClO_4_ in EC: DEC (3:7) | 20 | 76.40 | 272.00 | 165.38 |
|  | BFHC-NC5 | 1400 | 2 | 5 | 0.360 | 0.770 | 18.87 | 5.210 | 1.410 | 0.0095 | 1 M NaClO_4_ in EC: DEC (3:7) | 20 | 86.60 | 358.00 | 222.67 |
|  | BFHC-NC10 | 1400 | 2 | 5 | 0.358 | 1.030 | 8.86 | 4.840 | 1.120 | 0.0075 | 1 M NaClO_4_ in EC: DEC (3:7) | 20 | 83.40 | 338.00 | 210.21 |
| 120 | SRNDC-700 | 700 | 4 | 3 | 0.358 | 1.060 | 347.88 | - | - | 0.2200 | 1 M NaClO_4_ in PC with 5% FEC | 100 | 60.50 | 432.40 | 0.00 |
|  | SRNDC-800 | 800 | 4 | 3 | 0.352 | 1.010 | 435.13 | - | - | 0.5110 | 1 M NaClO_4_ in PC with 5% FEC | 100 | 52.20 | 232.86 | 0.00 |
| 121 | HC-1200 | 1200 | 2 | 3 | 0.385 | 1.010 | 49.00 | - | - | - | 1 M NaClO_4_ in PC with 5% FEC | 50 | 68.00 | 319.30 | 200.00 |
|  | HC-1200M | 1200 | 2 | 3 | 0.379 | 0.990 | 70.00 | - | - | - | 1 M NaClO_4_ in PC with 5% FEC | 50 | 63.00 | 323.60 | 187.70 |
| 122 | MFCP-900 | 900 | 3 | 3 | 0.397 | 2.990 | 763.82 | - | - | 0.3520 | 1 M NaPF_6_ in diglyme | 20 | 42.70 | 78.30 | 0.00 |
| 123 | RC-1200 | 1200 | 2 | 5 | 0.376 | 2.950 | 8.40 | 3.303 | 1.510 | - | 1 M NaClO_4_ in EC: PC (1:1) | 30 | 79.94 | 222.00 | 108.74 |
|  | RC-GO-1200 | 1200 | 2 | 5 | 0.380 | 3.360 | 21.06 | 3.906 | 1.521 | - | 1 M NaClO_4_ in EC: PC (1:1) | 30 | 74.80 | 274.20 | 167.96 |
|  | RC-LO-1200 | 1200 | 2 | 5 | 0.385 | 3.320 | 21.19 | 3.809 | 1.514 | - | 1 M NaClO_4_ in EC: PC (1:1) | 30 | 80.90 | 246.80 | 123.30 |
| 124 | FC-HC | 1300 | 6 | 4 | 0.354 | 1.155 | 67.86 | - | - | - | 1 M NaPF_6_ in EC: DEC (1:1) | 20 | 80.00 | 275.00 | 163.02 |
|  | MCC-HC | 1300 | 6 | 4 | 0.356 | 1.081 | 97.34 | - | - | - | 1 M NaPF_6_ in EC: DEC (1:1) | 20 | 79.00 | 300.00 | 180.12 |
| 125 | ZAD-3–800 | 800 | 1 | - | 0.374 | 1.560 | 678.00 | - | - | 0.2250 | 1 M NaClO_4_ in EC: DEC (1:1) | 50 | 37.00 | 293.00 | 0.00 |
| 126 | C-1 | 900 | 8 | - | 0.368 | 1.330 | 103.30 | - | - | - | 1 M NaCF_3_SO_3_ in diglyme | 20 | 66.00 | 322.47 | 0.00 |
| 127 | 800-SPC | 800 | 2 | - | 0.371 | 2.860 | 50.94 | - | 1.440 | 0.0345 | 1 M NaClO_4_ in EC: DMC (1:1) | 100 | 61.20 | 333.00 | 0.00 |
| 128 | SAPC1000 | 1000 | 3 | - | 0.372 | 3.380 | 20.60 | - | - | - | 1 M NaClO_4_ in EC: PC (1:1) | 20 | 62.91 | 212.00 | 69.37 |
|  | SAPC1200 | 1200 | 3 | - | 0.367 | 2.610 | 415.80 | - | - | - | 1 M NaClO_4_ in EC: PC (1:1) | 20 | 67.26 | 265.00 | 80.00 |
| 129 | PFHC-1 | 1100 | 3 | - | 0.389 | 2.064 | 338.00 | - | - | - | 1 M NaClO_4_ in EC: DEC (1:1) | 20 | 62.50 | 243.40 | 125.79 |
|  | PFHC-8 | 1100 | 3 | - | 0.385 | 2.194 | 328.00 | - | - | - | 1 M NaClO_4_ in EC: DEC (1:1) | 20 | 69.10 | 277.00 | 139.37 |
| 130 | HC-1000 | 1000 | 2 | 3 | 0.377 | 1.852 | 93.25 | 3.280 | 3.125 | 0.0200 | 1 M NaPF_6_ in EC: DMC (1:1) | 20 | 74.38 | 238.50 | 115.43 |
|  | HC-1200 | 1200 | 2 | 3 | 0.376 | 1.695 | 54.04 | 3.430 | 3.541 | 0.0180 | 1 M NaPF_6_ in EC: DMC (1:1) | 20 | 79.62 | 245.70 | 147.29 |
|  | HC-1400 | 1400 | 2 | 3 | 0.371 | 1.449 | 17.34 | 3.650 | 3.571 | 0.0180 | 1 M NaPF_6_ in EC: DMC (1:1) | 20 | 86.43 | 292.70 | 203.21 |
|  | HC-1600 | 1600 | 2 | 3 | 0.370 | 1.205 | 11.99 | 3.760 | 7.337 | 0.0050 | 1 M NaPF_6_ in EC: DMC (1:1) | 20 | 77.01 | 262.20 | 201.40 |
| 131 | NPHC | 1300 | 6 | 0.5 | 0.380 | 1.170 | 285.80 | - | - | 0.0560 | 1 M NaClO_4_ in EC: DEC (1:1) | 25 | 72.57 | 336.00 | 176.47 |
|  | NHC | 1300 | 6 | 0.5 | 0.372 | 1.050 | 6.90 | - | - | 0.0013 | 1 M NaClO_4_ in EC: DEC (1:1) | 25 | 73.00 | 292.00 | 150.33 |
|  | HC-commercial | 1300 | 6 | 0.5 | 0.378 | 1.030 | 2.90 | - | - | 0.0004 | 1 M NaClO_4_ in EC: DEC (1:1) | 25 | 78.82 | 268.00 | 138.56 |
| 132 | HCS | 1100 | 5 | 5 | 0.390 | 1.070 | 122.50 | - | - | - | 1 M NaClO_4_ in EC: DEC (1:1) with 2% FEC | 100 | 56.83 | 308.00 | 0.00 |
| 133 | NPC | 1200 | - | 5 | 0.392 | 1.220 | 158.00 | - | - | - | 1 M NaPF_6_ in EC: DEC (1:1) | 33 | 59.00 | 342.00 | 163.38 |
| 134 | P-700 | 700 | 2 | 5 | 0.351 | 1.620 | 5.75 | - | - | - | 1 M NaClO_4_ in PC | 30 | 57.00 | 215.00 | 0.00 |
| 135 | ECC-1100 | 1100 | 2 | - | 0.380 | 1.510 | 316.00 | - | - | 0.4810 | 1 M NaClO_4_ in EC: DMC (1:1) | 100 | 54.30 | 306.00 | 112.57 |
|  | CC-1100 | 1100 | 2 | - | 0.370 | 1.340 | 296.00 | - | - | 0.2700 | 1 M NaClO_4_ in EC: DMC (1:1) | 100 | 55.80 | 215.00 | 72.79 |
| 136 | CBC1000 | 1000 | 2 | 3 | 0.386 | 2.220 | 74.70 | - | 1.570 | 0.0440 | 1 M NaCF_3_SO_3_ in DEC: EC (1:1) | 100 | 68.00 | 217.50 | 67.64 |
| 137 | NP-HCS | 800 | 3 | 3 | 0.380 | 1.030 | 188.00 | - | - | 0.3600 | 1 M NaClO_4_ in EC: DEC (1:1) with 5% FEC | 100 | 51.25 | 230.62 | 0.00 |
|  | NP-HPCS | 800 | 3 | 3 | 0.410 | 1.100 | 478.00 | - | - | 0.5500 | 1 M NaClO_4_ in EC: DEC (1:1) with 5% FEC | 100 | 46.90 | 274.00 | 0.00 |
| 138 | CBC13 | 1050 | - | - | 0.366 | 1.110 | 154.00 | 2.130 | 1.730 | 0.2030 | 1 M NaCF_3_SO_3_ in DEGDME | 50 | 57.90 | 505.00 | 0.00 |
|  | CBCH13 | 1050 | - | - | 0.365 | 1.140 | 162.00 | 2.110 | 1.730 | 0.2250 | 1 M NaCF_3_SO_3_ in DEGDME | 50 | 57.40 | 175.00 | 0.00 |
| 139 | HCS-900 | 900 | - | - | 0.373 | 2.640 | 3.30 | 1.070 | 1.960 | 0.0030 | 1 M NaClO_4_ in EC: DMC (1:1) | 30 | 55.80 | 217.80 | 76.50 |
|  | HCS-1100 | 1100 | - | - | 0.374 | 2.190 | 9.52 | 1.120 | 2.080 | 0.0150 | 1 M NaClO_4_ in EC: DMC (1:1) | 30 | 68.60 | 314.50 | 180.90 |
|  | HCS-1300 | 1300 | - | - | 0.370 | 1.980 | 24.02 | 1.210 | 2.130 | 0.0190 | 1 M NaClO_4_ in EC: DMC (1:1) | 30 | 72.00 | 303.60 | 174.87 |
|  | HCS-1500 | 1500 | - | - | 0.366 | 1.580 | 11.58 | 1.310 | 2.410 | 0.0160 | 1 M NaClO_4_ in EC: DMC (1:1) | 30 | 73.60 | 304.00 | 200.50 |
|  | HCS-1700 | 1700 | - | - | 0.363 | 1.450 | 9.61 | 1.590 | 2.810 | 0.0130 | 1 M NaClO_4_ in EC: DMC (1:1) | 30 | 71.60 | 301.50 | 214.07 |
|  | HCS-1900 | 1900 | - | - | 0.358 | 1.310 | 11.25 | 1.610 | 3.070 | 0.0130 | 1 M NaClO_4_ in EC: DMC (1:1) | 30 | 68.80 | 294.60 | 212.56 |
| 140 | LPCHP | 800 | 3 | 5 | 0.359 | 1.740 | 5.00 | - | - | - | 1 M NaClO_4_ in EC: DMC (1:1) with 5% FEC | 20 | 53.00 | 143.00 | 0.00 |
| 141 | 1200HCMNT | 1200 | 2 | - | 0.390 | 1.630 | 10.06 | - | 1.900 | - | 1 M NaClO_4_ in EC: DMC (1:1) | 30 | 79.00 | 228.60 | 98.77 |
|  | 1400HCMNT | 1400 | 2 | - | 0.380 | 1.710 | 9.16 | - | 1.890 | - | 1 M NaClO_4_ in EC: DMC (1:1) | 30 | 80.00 | 292.50 | 120.90 |
| 142 | RFHC-1300 | 1300 | 3 | - | 0.398 | 2.325 | 563.52 | 3.103 | 1.425 | - | 1 M NaClO_4_ in EC: DEC (1:1) | 25 | 64.00 | 240.00 | 135.22 |
|  | RFHC-1500 | 1500 | 3 | - | 0.389 | 2.007 | 450.23 | 3.427 | 1.472 | - | 1 M NaClO_4_ in EC: DEC (1:1) | 25 | 57.00 | 214.00 | 124.91 |
| 143 | HC-7 | 700 | 2 | 10 | 0.379 | 2.130 | 307.20 | - | - | - | 1 M NaClO_4_ in EC: DEC (1:1) | 20 | 40.00 | - | 0.00 |
|  | NHC-7 | 700 | 2 | 10 | 0.394 | 2.700 | 602.10 | - | - | - | 1 M NaClO_4_ in EC: DEC (1:1) | 20 | 34.00 | 324.70 | 0.00 |
| 144 | PCF | 1100 | 2 | - | 0.373 | 2.285 | 35.74 | 5.981 | 1.671 | 0.0790 | 1 M NaClO_4_ in EC: DMC (1:1) | 25 | 69.90 | 226.30 | 93.01 |
|  | MCCF-1 | 1100 | 2 | - | 0.374 | 2.296 | 47.02 | 5.407 | 1.567 | 0.0850 | 1 M NaClO_4_ in EC: DMC (1:1) | 25 | 74.40 | 254.60 | 113.15 |
|  | MCCF-5 | 1100 | 2 | - | 0.379 | 2.827 | 88.86 | 5.334 | 1.552 | 0.1310 | 1 M NaClO_4_ in EC: DMC (1:1) | 25 | 73.70 | 366.50 | 146.34 |
|  | MCCF-10 | 1100 | 2 | - | 0.380 | 2.553 | 355.02 | 4.577 | 1.548 | 0.2820 | 1 M NaClO_4_ in EC: DMC (1:1) | 25 | 68.40 | 320.70 | 129.58 |
| 145 | HC-1200 | 1200 | - | 5 | 0.403 | 1.280 | 11.33 | - | - | - | 1 M NaClO_4_ in PC with 5% FEC | 50 | 70.39 | 326.70 | 120.69 |
|  | HC-1200H | 1200 | - | 5 | 0.381 | 1.330 | 12.73 | - | - | - | 1 M NaClO_4_ in PC with 5% FEC | 50 | 68.96 | 284.73 | 90.64 |
| 146 | PL-1300 | 1300 | 3 | - | 0.375 | 1.077 | 6.78 | 2.820 | 1.670 | - | 1 M NaClO_4_ in EC: DEC (1:1) | 25 | 76.70 | 338.50 | 241.11 |
|  | LA31-1300 | 1300 | 3 | - | 0.366 | 1.024 | 5.34 | 3.140 | 1.780 | - | 1 M NaClO_4_ in EC: DEC (1:1) | 25 | 82.00 | 296.50 | 193.75 |
|  | LA13-1300 | 1300 | 3 | - | 0.356 | 1.002 | 4.74 | 3.540 | 1.890 | - | 1 M NaClO_4_ in EC: DEC (1:1) | 25 | 74.80 | 280.00 | 172.22 |
| 147 | a-LC-1000 | 1000 | 5 | 5 | 0.380 | 3.050 | 56.00 | - | - | - | 1 M NaPF_6_ in EC: DEC (1:1) | 20 | 77.00 | 214.00 | 73.46 |
|  | a-LC-1200 | 1200 | 5 | 5 | 0.379 | 2.960 | 31.00 | - | - | - | 1 M NaPF_6_ in EC: DEC (1:1) | 20 | 82.00 | 256.00 | 108.46 |
|  | a-LC-1400 | 1400 | 5 | 5 | 0.374 | 2.430 | 11.00 | - | - | - | 1 M NaPF_6_ in EC: DEC (1:1) | 20 | 83.00 | 242.00 | 125.77 |
|  | a-LC-1600 | 1600 | 5 | 5 | 0.369 | 2.160 | 5.00 | - | - | - | 1 M NaPF_6_ in EC: DEC (1:1) | 20 | 83.00 | 234.00 | 138.08 |
| 148 | BPC-1300 | 1300 | 2 | 3 | 0.374 | 1.590 | 20.74 | - | 1.680 | 0.0270 | 1 M NaClO_4_ in EC: DEC (1:1) with 2% FEC | 50 | 72.00 | 319.00 | 116.91 |
| 149 | CLRS-2-1000 | 1000 | 1 | 5 | 0.374 | 1.540 | 11.80 | 4.790 | 1.090 | - | 1 M NaClO_4_ in EC: DEC (1:1) with 5% FEC | 25 | 61.00 | 267.00 | 80.00 |
|  | CLRS-2-1300 | 1300 | 1 | 5 | 0.368 | 1.410 | 12.60 | 5.220 | 1.140 | - | 1 M NaClO_4_ in EC: DEC (1:1) with 5% FEC | 25 | 70.00 | 329.00 | 182.90 |
|  | CLRS-2-1600 | 1600 | 1 | 5 | 0.361 | 0.780 | 11.20 | 5.950 | 1.270 | - | 1 M NaClO_4_ in EC: DEC (1:1) with 5% FEC | 25 | 74.00 | 324.00 | 202.80 |
|  | CLRS-12-1000 | 1000 | 1 | 5 | 0.375 | 1.790 | 13.20 | 5.510 | 1.130 | - | 1 M NaClO_4_ in EC: DEC (1:1) with 5% FEC | 25 | 62.00 | 265.00 | 93.00 |
|  | CLRS-12-1300 | 1300 | 1 | 5 | 0.370 | 1.140 | 16.30 | 6.130 | 1.270 | - | 1 M NaClO_4_ in EC: DEC (1:1) with 5% FEC | 25 | 74.00 | 347.00 | 190.80 |
|  | CLRS-12-1600 | 1600 | 1 | 5 | 0.361 | 0.780 | 14.60 | 6.540 | 1.360 | - | 1 M NaClO_4_ in EC: DEC (1:1) with 5% FEC | 25 | 72.00 | 317.00 | 210.60 |
| 150 | CWS | 1100 | 6 | 10 | 0.385 | 1.051 | 32.70 | - | - | 0.0140 | 1 M NaClO_4_ in EC: DMC (1:1) | 100 | 56.55 | 141.20 | 91.15 |
|  | CWS-C | 1100 | 6 | 10 | 0.389 | 1.047 | 8.80 | - | - | - | 1 M NaClO_4_ in EC: DMC (1:1) | 100 | 59.45 | 184.50 | 98.65 |
|  | CWS-K | 1100 | 6 | 10 | 0.378 | 1.084 | 16.30 | - | - | - | 1 M NaClO_4_ in EC: DMC (1:1) | 100 | 53.40 | 167.90 | 105.44 |
|  | CWS-CK | 1100 | 6 | 10 | 0.387 | 1.050 | 9.40 | - | - | 0.0005 | 1 M NaClO_4_ in EC: DMC (1:1) | 100 | 64.00 | 244.60 | 149.32 |
| 151 | GCS-0-1200 | 1200 | 6 | - | 0.394 | 1.160 | 38.30 | 3.540 | 2.460 | 0.0099 | 1 M NaClO_4_ in EC: DMC (1:1) | 100 | 71.20 | 296.00 | 175.00 |
| 152 | GC1000 | 1000 | 2 | 5 | 0.375 | 1.150 | 11.00 | 0.900 | - | 0.0210 | 1 M NaPF_6_ in EC: DMC (1:1) | 30 | 72.00 | 265.00 | 88.38 |
|  | GC1300 | 1300 | 2 | 5 | 0.368 | 1.320 | 4.00 | 1.000 | - | 0.0200 | 1 M NaPF_6_ in EC: DMC (1:1) | 30 | 83.00 | 301.00 | 134.97 |
| 153 | HHC | 1300 | 2 | 2 | 0.408 | 1.016 | 25.58 | - | - | - | 1 M NaClO_4_ in EC: PC (1:1) | 100 | 65.90 | 294.03 | 144.40 |
| 154 | 12-200-1300 | 1300 | 2 | - | 0.383 | 1.900 | 103.00 | 7.610 | 2.700 | 0.0540 | 1 M NaPF_6_ in EC: DEC (1:1) | 25 | 70.00 | 330.00 | 179.75 |
| 155 | STH | 600 | 2 | 5 | 0.362 | 0.420 | 8.67 | - | - | 0.00001 | 1 M NaClO_4_ in EC: DEC (1:1) | 100 | 51.00 | 72.00 | 0.00 |
| 156 | CCNs-600 | 600 | 2 | 5 | 0.398 | 0.750 | 262.00 | - | - | 0.2300 | 1 M NaClO_4_ in EC: DEC (1:1) with 5% FEC | 50 | 47.00 | 360.00 | 0.00 |
|  | CCNs-800 | 800 | 2 | 5 | 0.369 | 0.890 | 586.00 | - | - | 0.3400 | 1 M NaClO_4_ in EC: DEC (1:1) with 5% FEC | 50 | 39.33 | 265.00 | 0.00 |
| 157 | N330 | 1050 | - | - | 0.366 | 1.070 | 78.00 | 2.260 | 1.700 | 0.1560 | Ether-based electrolyte | 50 | 61.32 | 232.41 | 0.00 |
|  | CBN10 | 1050 | - | - | 0.365 | 1.100 | 233.00 | 2.480 | 1.670 | 0.2620 | Ether-based electrolyte | 50 | 69.31 | 192.58 | 0.00 |
|  | CBN54 | 1050 | - | - | 0.360 | 1.240 | 783.00 | 2.240 | 1.830 | 0.8390 | Ether-based electrolyte | 50 | 60.61 | 101.38 | 0.00 |
| 158 | SB-750 | 750 | 6 | 5 | 0.381 | 1.330 | 8.00 | - | - | - | 1 M NaClO_4_ in EC: DEC (1:1) | 30 | 60.00 | 250.00 | 53.23 |
|  | SB-1050 | 1050 | 6 | 5 | 0.374 | 0.750 | 2.00 | - | - | - | 1 M NaClO_4_ in EC: DEC (1:1) | 30 | 72.00 | 275.00 | 109.01 |
| 159 | C-HC | 1300 | 3 | - | 0.410 | 0.787 | 4.00 | - | - | - | 0.8 M NaPF_6_ in EC: DMC (1:1) | 20 | 85.00 | 310.00 | 186.44 |
| 160 | CNFs | 800 | 1 | 10 | 0.386 | 0.990 | 465.00 | - | - | - | 1 M NaPF_6_ in DME | 50 | 54.85 | 200.00 | 0.00 |
| 161 | GL-800 | 800 | 2 | 5 | 0.397 | 2.500 | 205.54 | 4.390 | 1.280 | - | 1 M NaClO_4_ in EC: DEC (1:1) with 5% FEC | 30 | 50.00 | 313.97 | 49.32 |
|  | GL-1000 | 1000 | 2 | 5 | 0.393 | 2.220 | 220.92 | 5.470 | 1.510 | - | 1 M NaClO_4_ in EC: DEC (1:1) with 5% FEC | 30 | 51.21 | 383.01 | 93.70 |
| 162 | RH1600 | 1600 | - | 3 | 0.386 | 1.180 | 218.00 | 7.140 | - | 0.4100 | 0.6 M NaPF_6_ in EC: DMC (1:1) | 30 | 53.10 | 276.00 | 190.00 |
| 163 | HCCS800 | 800 | 2 | 5 | 0.391 | 1.870 | 76.67 | 1.560 | 0.710 | 0.0670 | 1 M NaClO_4_ in EC: DEC (1:1) with 5% FEC | 50 | 59.80 | 292.00 | 81.00 |
|  | HCCS900 | 900 | 2 | 5 | 0.387 | 1.820 | 147.16 | 1.630 | 0.750 | 0.0720 | 1 M NaClO_4_ in EC: DEC (1:1) with 5% FEC | 50 | 63.10 | 310.10 | 81.44 |
|  | HCCS1000 | 1000 | 2 | 5 | 0.383 | 1.690 | 253.46 | 1.760 | 0.800 | 0.1100 | 1 M NaClO_4_ in EC: DEC (1:1) with 5% FEC | 50 | 64.90 | 337.90 | 129.64 |
|  | HCCS1100 | 1100 | 2 | 5 | 0.380 | 1.650 | 312.63 | 1.980 | 0.970 | 0.1140 | 1 M NaClO_4_ in EC: DEC (1:1) with 5% FEC | 50 | 62.20 | 281.20 | 99.72 |
|  | HCCS1000A | 1000 | 2 | 5 | 0.369 | 1.050 | 1988.90 | 1.850 | 0.690 | 1.6300 | 1 M NaClO_4_ in EC: DEC (1:1) with 5% FEC | 50 | 37.30 | 458.60 | 0.00 |
| 164 | SC-800 | 800 | 4 | 5 | 0.400 | 0.930 | 331.40 | - | - | - | 1 M NaClO_4_ in EC: PC (1:1) | 50 | 45.45 | 504.85 | 0.00 |
| 165 | 0AC | 1000 | 2 | 3 | 0.353 | 1.560 | 2.74 | - | 1.660 | 0.0100 | 1 M NaPF_6_ in EC: DMC (1:1) | 30 | 69.00 | 130.10 | 0.00 |
|  | 10AC | 1000 | 2 | 3 | 0.374 | 1.980 | 6.15 | - | 0.852 | 0.0270 | 1 M NaPF_6_ in EC: DMC (1:1) | 30 | 71.00 | 177.50 | 60.28 |
|  | 30AC | 1000 | 2 | 3 | 0.370 | 2.030 | 10.95 | - | 0.925 | 0.0910 | 1 M NaPF_6_ in EC: DMC (1:1) | 30 | 63.00 | 203.50 | 61.00 |
| 166 | CCI-1000 | 1000 | 2 | - | 0.386 | 1.200 | 73.00 | - | 1.570 | - | 1 M NaClO_4_ in EC: PC (1:1) | 20 | 66.00 | 262.00 | 66.51 |
| 167 | PSDHCs-2 | 800 | 2 | 10 | 0.377 | 2.490 | 338.00 | - | - | 0.2150 | 1 M NaClO_4_ in EC: PC (1:1) | 30 | 60.00 | 247.00 | 61.73 |
|  | PSDHCs-6 | 800 | 2 | 10 | 0.381 | 2.510 | 334.00 | - | - | 0.2640 | 1 M NaClO_4_ in EC: PC (1:1) | 30 | 43.00 | 152.00 | 27.08 |
| 168 | HC-1700 | 1700 | 0.33 | 5 | 0.367 | 0.781 | 4.70 | - | - | - | 0.6 M NaPF_6_ in diglyme | 30 | 92.00 | 280.00 | 214.52 |
| 169 | LS1000 | 1000 | 2 | - | 0.391 | 2.450 | 751.60 | - | - | 0.2330 | 1 M NaClO_4_ in PC with 2% FEC | 50 | 45.80 | 256.00 | 63.48 |
| 170 | LJ-1500 | 1500 | 3 | 5 | 0.370 | 1.640 | 7.39 | 3.500 | 1.700 | - | 1 M NaClO_4_ in EC: DEC (1:1) | 25 | 86.21 | 334.50 | 259.70 |
| 171 | BAM-CF | 1000 | 1 | - | 0.397 | 1.226 | 29.10 | - | - | 0.0440 | 1 M NaClO_4_ in PC: DMC (1:1) with 5% FEC | 50 | 72.70 | 320.00 | 134.52 |
|  | PAU-CF | 1000 | 1 | - | 0.390 | 1.032 | 16.55 | - | - | 0.0540 | 1 M NaClO_4_ in PC: DMC (1:1) with 5% FEC | 50 | 72.90 | 302.00 | 134.00 |
| 172 | HC | 790 | 2 | 5 | 0.360 | 0.980 | 743.00 | - | - | - | 1 M NaClO_4_ in EC: DMC (1:1) | 50 | 41.67 | 190.30 | 0.00 |
| 173 | 3DHPCM | 800 | 1.5 | 5 | 0.366 | 1.130 | 206.38 | - | - | 0.1630 | 1 M NaPF_6_ in EC: DEC (1:1) | 50 | 62.20 | 286.00 | 0.00 |
| 174 | 3DHSC-2 | 1500 | 2 | - | 0.365 | 1.070 | 100.00 | 4.990 | 1.330 | - | 1 M NaClO_4_ in EC: DEC (1:1) | 50 | 64.40 | 208.00 | 94.51 |
|  | 3DHSC-4 | 1500 | 2 | - | 0.372 | 1.090 | 119.00 | 4.800 | 1.220 | - | 1 M NaClO_4_ in EC: DEC (1:1) | 50 | 60.00 | 215.50 | 69.36 |
| 175 | GLC-1000 | 1000 | 2 | 5 | 0.381 | 2.970 | 235.00 | 1.969 | 1.311 | 0.3700 | 1 M NaCF_3_SO_3_ in diglyme | 20 | 72.65 | 251.00 | 74.28 |
| 176 | CC-Pre-800 | 800 | 2 | - | 0.403 | 2.870 | 11.67 | 6.700 | 1.280 | 0.0130 | 1 M NaPF_6_ in EC: DMC (1:1) | 30 | 62.00 | 256.00 | 82.00 |
|  | CC-1200 | 1200 | 2 | - | 0.397 | 2.470 | 9.23 | 7.800 | 1.250 | 0.0140 | 1 M NaPF_6_ in EC: DMC (1:1) | 30 | 71.00 | 311.00 | 160.00 |
|  | CC-1400 | 1400 | 2 | - | 0.392 | 2.220 | 8.57 | 8.660 | 1.360 | 0.0110 | 1 M NaPF_6_ in EC: DMC (1:1) | 30 | 76.00 | 326.00 | 189.00 |
|  | CC-1600 | 1600 | 2 | - | 0.376 | 1.890 | 5.54 | 10.170 | 1.450 | 0.0150 | 1 M NaPF_6_ in EC: DMC (1:1) | 30 | 81.00 | 358.00 | 231.00 |
| 177 | SGHC-800 | 800 | 2.5 | 5 | 0.397 | 3.080 | 31.00 | 2.619 | 1.761 | - | 1 M NaClO_4_ in EC: DEC (1:1) | 50 | 58.60 | 294.90 | 91.09 |
|  | SGHC-1000 | 1000 | 2.5 | 5 | 0.388 | 2.700 | 20.80 | 3.381 | 1.813 | - | 1 M NaClO_4_ in EC: DEC (1:1) | 50 | 68.10 | 354.80 | 165.95 |
| 178 | C90-150 | 800 | 3 | 2 | 0.400 | 1.800 | 528.00 | - | 1.340 | 0.8900 | 1 M NaPF_6_ in EC: MEC (1:1) | 27 | 42.48 | 192.00 | 0.00 |
| 179 | ME-CC | 1400 | 2 | 4 | 0.375 | 1.110 | 134.00 | - | - | - | 1 M NaClO_4_ in EC: DEC (1:1) | 30 | 73.71 | 188.50 | 66.10 |
|  | ME-CNT-CC | 1400 | 2 | 4 | 0.378 | 1.080 | 146.00 | - | - | - | 1 M NaClO_4_ in EC: DEC (1:1) | 30 | 78.39 | 203.60 | 81.99 |
| 180 | HC-21-1550 | 1550 | 2 | - | 0.391 | 1.240 | 1.44 | - | - | - | 1 M NaPF_6_ in EC: DMC (1:1) | 30 | 86.00 | 384.00 | 278.79 |
|  | HC-81-1550 | 1550 | 2 | - | 0.389 | 1.245 | 0.39 | - | - | - | 1 M NaPF_6_ in EC: DMC (1:1) | 30 | 82.00 | 351.00 | 238.09 |
|  | HC-1550 | 1550 | 2 | - | 0.386 | 1.243 | 0.26 | - | - | - | 1 M NaPF_6_ in EC: DMC (1:1) | 30 | 80.00 | 304.00 | 212.12 |
| 181 | CHTC1000 | 1000 | 1 | 5 | 0.394 | 1.260 | 232.00 | - | - | - | 1 M NaClO_4_ in EC: DMC (1:1) | 30 | 50.00 | 178.00 | 40.36 |
|  | CHTC1300 | 1300 | 1 | 5 | 0.392 | 1.340 | 34.00 | - | - | - | 1 M NaClO_4_ in EC: DMC (1:1) | 30 | 75.00 | 297.00 | 164.63 |
|  | CCD1500 | 1500 | 1 | 5 | 0.371 | 1.670 | 12.00 | - | - | - | 1 M NaClO_4_ in EC: DMC (1:1) | 30 | 89.00 | 279.00 | 173.75 |
|  | CHTC1500 | 1500 | 1 | 5 | 0.381 | 1.620 | 40.00 | - | - | - | 1 M NaClO_4_ in EC: DMC (1:1) | 30 | 76.00 | 298.00 | 181.63 |
| 182 | AC | 1100 | 2 | - | 0.389 | 1.050 | 10.80 | 9.610 | 1.040 | 0.0240 | 1 M NaPF_6_ in EC: DMC (1:1) | 30 | 79.00 | 263.80 | 93.68 |
|  | 3DAC | 1100 | 2 | - | 0.408 | 1.110 | 32.80 | 7.980 | 0.896 | 0.0740 | 1 M NaPF_6_ in EC: DMC (1:1) | 30 | 75.00 | 280.10 | 81.92 |
| 183 | HAS-900 | 900 | 2 | - | 0.402 | 2.080 | 2.70 | 4.660 | 1.180 | - | 1 M NaClO_4_ in EC: DEC (1:1) | 25 | 66.00 | 286.00 | 90.73 |
|  | HAS-1100 | 1100 | 2 | - | 0.393 | 1.820 | 46.10 | 5.020 | 1.220 | - | 1 M NaClO_4_ in EC: DEC (1:1) | 25 | 65.00 | 306.00 | 153.17 |
|  | HASH-900 | 900 | 2 | - | 0.392 | 1.890 | 27.90 | 5.030 | 1.170 | - | 1 M NaClO_4_ in EC: DEC (1:1) | 25 | 73.00 | 321.00 | 99.19 |
|  | HASH-1100 | 1100 | 2 | - | 0.388 | 1.670 | 56.70 | 5.870 | 1.200 | - | 1 M NaClO_4_ in EC: DEC (1:1) | 25 | 77.00 | 359.00 | 177.82 |
|  | HASH-1300 | 1300 | 2 | - | 0.382 | 1.450 | 13.40 | 6.190 | 1.220 | - | 1 M NaClO_4_ in EC: DEC (1:1) | 25 | 79.00 | 400.00 | 234.68 |
|  | HASH-1500 | 1500 | 2 | - | 0.372 | 1.350 | 12.40 | 6.510 | 1.260 | - | 1 M NaClO_4_ in EC: DEC (1:1) | 25 | 80.00 | 358.00 | 264.31 |
| 184 | N, P-CS | 1200 | 2 | - | 0.391 | 1.692 | 638.90 | - | 1.238 | 0.2921 | 1 M NaClO_4_ in EC: DEC (1:1) | 50 | 53.10 | 309.00 | 67.84 |
| 185 | CNSA-6 | 900 | 2 | 10 | 0.378 | 1.640 | 1886.00 | - | - | 1.0200 | 1 M NaClO_4_ in EC: DEC (1:1) | 100 | 60.27 | 653.18 | 0.00 |
| 186 | PSHC-900 | 900 | 2 | 10 | 0.373 | 2.580 | 224.02 | 6.501 | 1.068 | 0.1220 | 1 M NaClO_4_ in EC: PC (1:1) | 30 | 60.09 | 271.00 | 91.75 |
|  | PSHC-1000 | 1000 | 2 | 10 | 0.363 | 2.480 | 137.07 | 6.765 | 1.072 | 0.0640 | 1 M NaClO_4_ in EC: PC (1:1) | 30 | 62.16 | 317.00 | 108.25 |
|  | PSHC-1100 | 1100 | 2 | 10 | 0.360 | 2.460 | 194.49 | 6.806 | 1.134 | 0.1000 | 1 M NaClO_4_ in EC: PC (1:1) | 30 | 64.55 | 284.00 | 118.56 |
| 187 | HCM55-1400 | 1400 | 1 | 2 | 0.392 | 1.830 | 6.50 | - | - | - | 0.8 M NaPF_6_ in DMC: EC (1:1) | 30 | 82.00 | 318.60 | 219.38 |
| 188 | CP | 1000 | 1 | 10 | 0.440 | 1.020 | 1.86 | 3.860 | 1.130 | - | 1 M NaClO_4_ in PC with 5% FEC | 20 | 67.30 | 310.20 | 135.00 |
| 189 | HC-1100 | 1100 | 2 | - | 0.401 | 1.150 | 78.97 | - | - | 0.0300 | 1 M NaClO_4_ in PC with 5% FEC | 50 | 69.20 | 313.82 | 161.56 |
|  | HC-1100H | 1100 | 2 | - | 0.394 | 1.180 | 103.83 | - | - | 0.0400 | 1 M NaClO_4_ in PC with 5% FEC | 50 | 65.20 | 246.52 | 109.40 |
| 190 | HC | 1300 | - | 1 | 0.375 | 1.350 | 1.80 | 2.820 | 1.130 | - | 1 M NaClO_4_ in EC: DEC (1:1) | 20 | 78.30 | 306.40 | 181.80 |
| 191 | RS-900 | 900 | 3 | - | 0.403 | 1.852 | 325.27 | 3.610 | 1.100 | 0.1800 | 1 M NaClO_4_ in EC: DEC (1:1) | 25 | 49.20 | 177.70 | 39.53 |
|  | RS-1100 | 1100 | 3 | - | 0.398 | 1.640 | 82.01 | 3.830 | 1.460 | 0.050 | 1 M NaClO_4_ in EC: DEC (1:1) | 25 | 73.10 | 338.80 | 125.78 |
|  | RS-1300 | 1300 | 3 | - | 0.391 | 1.520 | 36.20 | 4.520 | 1.600 | 0.040 | 1 M NaClO_4_ in EC: DEC (1:1) | 25 | 77.00 | 372.00 | 206.18 |
|  | RS-1500 | 1500 | 3 | - | 0.375 | 1.390 | 23.87 | 6.180 | 1.880 | 0.020 | 1 M NaClO_4_ in EC: DEC (1:1) | 25 | 79.40 | 343.10 | 194.82 |
| 192 | HC-1000 | 1000 | 1 | 5 | 0.386 | 2.150 | 15.60 | 7.480 | 9.310 | - | 1 M NaPF_6_ in EC: DEC (1:1) | 25 | 78.30 | 242.00 | 133.00 |
|  | HC-1200 | 1200 | 1 | 5 | 0.384 | 2.020 | 35.10 | 8.290 | 10.020 | - | 1 M NaPF_6_ in EC: DEC (1:1) | 25 | 84.50 | 260.00 | 171.00 |
|  | HC-1300 | 1300 | 1 | 5 | 0.376 | 1.730 | 26.70 | 8.420 | 10.290 | - | 1 M NaPF_6_ in EC: DEC (1:1) | 25 | 87.60 | 277.00 | 196.00 |
|  | HC-1400 | 1400 | 1 | 5 | 0.371 | 1.690 | 34.70 | 8.540 | 10.890 | - | 1 M NaPF_6_ in EC: DEC (1:1) | 25 | 88.40 | 274.00 | 205.00 |
|  | HC-1000 | 1000 | 1 | 5 | 0.385 | 1.890 | 103.00 | 8.250 | 8.670 | - | 1 M NaPF_6_ in EC: DEC (1:1) | 25 | 70.70 | 223.00 | 134.00 |
|  | HC-1200 | 1200 | 1 | 5 | 0.383 | 1.790 | 66.50 | 8.380 | 9.690 | - | 1 M NaPF_6_ in EC: DEC (1:1) | 25 | 80.70 | 264.00 | 188.00 |
|  | HC-1300 | 1300 | 1 | 5 | 0.379 | 1.710 | 38.60 | 8.730 | 10.350 | - | 1 M NaPF_6_ in EC: DEC (1:1) | 25 | 86.50 | 295.00 | 217.00 |
|  | HC-1400 | 1400 | 1 | 5 | 0.376 | 1.580 | 33.30 | 9.240 | 10.720 | - | 1 M NaPF_6_ in EC: DEC (1:1) | 25 | 88.30 | 300.00 | 226.00 |
| 193 | HPCMs | 700 | 1 | 5 | 0.376 | 0.920 | 1006.00 | - | - | 0.3900 | 1 M NaClO_4_ in EC: DMC (1:1) | 100 | 31.40 | 261.00 | 0.00 |
| 194 | CPP | 900 | 1 | 5 | 0.410 | 1.070 | 171.54 | - | 1.510 | - | 1 M NaClO_4_ in EC: PC (1:1) | 100 | 59.80 | 221.50 | 53.73 |
| 195 | HC-5 | 1300 | 2 | 5 | 0.413 | 3.226 | 34.00 | 2.150 | 1.300 | 0.0180 | 1 M NaClO_4_ in EC: DEC (1:1) | 20 | 86.10 | 361.00 | 196.01 |
|  | HC-2 | 1300 | 2 | 2 | 0.401 | 2.500 | 20.00 | 2.260 | 1.350 | 0.0140 | 1 M NaClO_4_ in EC: DEC (1:1) | 20 | 85.20 | 350.00 | 215.96 |
|  | HC-1 | 1300 | 2 | 1 | 0.398 | 2.380 | 13.00 | 2.400 | 1.420 | 0.0060 | 1 M NaClO_4_ in EC: DEC (1:1) | 20 | 81.30 | 344.00 | 210.20 |
|  | HC-0.5 | 1300 | 2 | 0.5 | 0.391 | 1.923 | 1.74 | 2.530 | 1.460 | - | 1 M NaClO_4_ in EC: DEC (1:1) | 20 | 78.90 | 338.00 | 225.72 |
| 196 | CFs-P15 | 1000 | 5 | - | 0.383 | 1.120 | 50.63 | - | - | 0.0200 | 1 M NaClO_4_ in PC with 2% FEC | 50 | 55.70 | 288.00 | 93.74 |
| 197 | PCNS1000 | 1000 | 2 | 5 | 0.382 | 1.090 | 546.00 | - | - | - | 1 M NaClO_4_ in EC: DMC (1:1) | 100 | 66.00 | 296.00 | 0.00 |
| 198 | P750 | 750 | 4 | 2 | 0.345 | 0.910 | 21.80 | 1.460 | 1.340 | - | 1 M NaPF_6_ in EC: DEC (1:1) with 5% FEC | 20 | 54.70 | 152.00 | 0.00 |
|  | PS600 | 600 | 4 | 2 | 0.361 | 0.930 | 44.10 | 1.300 | 1.240 | - | 1 M NaPF_6_ in EC: DEC (1:1) with 5% FEC | 20 | 49.70 | 291.00 | 0.00 |
|  | PS750 | 750 | 4 | 2 | 0.366 | 1.050 | 75.20 | 1.410 | 1.380 | - | 1 M NaPF_6_ in EC: DEC (1:1) with 5% FEC | 20 | 49.10 | 378.00 | 0.00 |
| 199 | carbon sheets | 600 | 3 | 10 | 0.400 | 1.040 | 430.00 | - | - | 0.1980 | 0.5 M NaPF_6_ in aliphatic carbonates | 20 | 50.00 | 153.00 | 0.00 |
| 200 | FGS-800 | 800 | 3 | 5 | 0.430 | 0.930 | 586.65 | - | - | - | 1 M NaClO_4_ in EC: PC (1:1) with 2% FEC | 50 | 44.00 | - | 0.00 |
| 201 | PCNSs-700 | 700 | 2 | - | 0.382 | 1.650 | 313.00 | - | - | - | 1 M NaSO_3_CF_3_ in diglyme | 50 | 59.66 | 318.00 | 0.00 |
|  | HPCNSs-700-2 | 700 | 2 | - | 0.370 | 1.400 | 320.00 | - | - | - | 1 M NaSO_3_CF_3_ in diglyme | 50 | 56.92 | 222.00 | 0.00 |
|  | HPCNSs-700-6 | 700 | 2 | - | 0.370 | 1.370 | 379.00 | - | - | - | 1 M NaSO_3_CF_3_ in diglyme | 50 | 51.20 | 192.00 | 0.00 |
| 202 | SSHC1100 | 1100 | 2 | 2 | 0.395 | 0.356 | 234.50 | - | 0.800 | 0.1180 | 1 M NaClO_4_ in EC: DEC (1:1) with 2% FEC | 20 | 57.50 | 241.50 | 93.03 |
| 203 | PGY-800 | 800 | 5 | 5 | 0.385 | 2.940 | 9.97 | 8.400 | 1.090 | - | 1 M NaClO_4_ in EC: DEC (1:1) | 50 | 53.10 | 146.75 | 24.53 |
| 204 | NMC600 | 600 | 2 | 10 | 0.371 | 0.850 | 539.00 | - | - | 0.5600 | 1 M NaClO_4_ in EC: DEC (1:1) with 2% FEC | 30 | 41.72 | 392.53 | 0.00 |
|  | NMC700 | 700 | 2 | 10 | 0.366 | 0.940 | 531.00 | - | - | 0.6100 | 1 M NaClO_4_ in EC: DEC (1:1) with 2% FEC | 30 | 40.44 | 434.60 | 0.00 |
|  | NMC800 | 800 | 2 | 10 | 0.363 | 0.930 | 508.00 | - | - | 0.7100 | 1 M NaClO_4_ in EC: DEC (1:1) with 2% FEC | 30 | 40.94 | 372.19 | 0.00 |
|  | NMC900 | 900 | 2 | 10 | 0.359 | 0.920 | 467.00 | - | - | 0.8000 | 1 M NaClO_4_ in EC: DEC (1:1) with 2% FEC | 30 | 36.87 | 339.52 | 0.00 |
| 205 | p-MBC900 | 900 | 2 | - | 0.400 | 1.290 | 198.46 | - | - | 0.1300 | 1 M NaClO_4_ in EC: PC (1:1) | 50 | 52.00 | 161.20 | 0.00 |
| 206 | HPNDC | 700 | 1 | 5 | 0.380 | 0.890 | 1883.00 | - | - | - | 1 M NaClO_4_ in EC: DMC (1:1) | 100 | 31.90 | 400.00 | 0.00 |
| 207 | LHA-900 | 900 | - | - | 0.399 | 1.670 | 6.36 | 4.630 | 1.490 | - | 1 M NaClO_4_ in EC: DEC (1:1) | 25 | 59.00 | 286.00 | 84.35 |
|  | LHA-1500 | 1500 | - | - | 0.368 | 1.124 | 3.21 | 4.920 | 1.760 | - | 1 M NaClO_4_ in EC: DEC (1:1) | 25 | 73.00 | 345.00 | 182.43 |
|  | LHA-1600 | 1600 | - | - | 0.362 | 1.087 | 1.06 | 5.120 | 1.800 | - | 1 M NaClO_4_ in EC: DEC (1:1) | 25 | 75.00 | 314.00 | 170.24 |
| 208 | N-CNTs | 700 | 3 | 5 | 0.375 | 0.976 | 736.90 | - | - | - | 1 M NaClO_4_ in PC: EC (1:1) | 50 | 39.00 | 268.60 | 0.00 |
| 209 | KHC-900 | 900 | 2 | 5 | 0.390 | 1.490 | 1.00 | - | 1.130 | - | 1 M NaClO_4_ in EC: DEC (1:1) | 25 | 61.20 | 243.00 | 86.30 |
| 210 | HC1100C2h | 1100 | 2 | 3 | 0.374 | 1.760 | 126.60 | 2.500 | 1.520 | - | 1 M NaClO_4_ in EC: PC (1:1) | 20 | 64.00 | 220.00 | 107.14 |
|  | HC1300C2h | 1300 | 2 | 3 | 0.371 | 1.670 | 81.50 | 2.630 | 1.580 | - | 1 M NaClO_4_ in EC: PC (1:1) | 20 | 74.00 | 280.00 | 168.37 |
|  | HC1500C2h | 1500 | 2 | 3 | 0.367 | 1.500 | 8.90 | 2.930 | 1.640 | - | 1 M NaClO_4_ in EC: PC (1:1) | 20 | 83.00 | 330.00 | 221.43 |
|  | HC1600C2h | 1600 | 2 | 3 | 0.366 | 1.500 | 2.40 | 2.940 | 1.650 | - | 1 M NaClO_4_ in EC: PC (1:1) | 20 | 86.00 | 270.00 | 173.98 |
| 211 | HC-1300 | 1300 | 2 | 2 | 0.377 | 1.490 | 117.00 | 3.920 | 1.830 | 0.0700 | 1 M NaClO_4_ in EC: DEC (1:1) with 5% FEC | 20 | 63.40 | 335.29 | 196.08 |
| 212 | HC | 1100 | 5 | - | 0.377 | 1.300 | 8.50 | - | - | - | 1 M NaPF_6_ in EC: DEC (1:1) | 20 | 80.17 | 283.00 | 149.00 |
|  | P-HC | 1100 | 5 | - | 0.395 | 1.390 | 7.30 | - | - | - | 1 M NaPF_6_ in EC: DEC (1:1) | 20 | 74.02 | 359.00 | 202.00 |
|  | S-HC | 1100 | 5 | - | 0.383 | 1.180 | 5.20 | - | - | - | 1 M NaPF_6_ in EC: DEC (1:1) | 20 | 80.39 | 328.00 | 177.00 |
| 213 | NGHC | 1000 | 6 | 5 | 0.380 | 1.200 | 40.00 | - | - | - | 1 M NaClO_4_ in EC: DMC (1:1) | 100 | 56.61 | 214.00 | 81.51 |
| 214 | NDPC | 700 | 1 | - | 0.374 | 0.870 | 1300.00 | - | - | 0.5200 | 1 M NaClO_4_ in EC: DEC (1:1) | 100 | 33.70 | 419.00 | 0.00 |
| 215 | PHC800 | 800 | 2 | 5 | 0.417 | 3.030 | 557.00 | - | 1.2500 | 0.2900 | 1 M NaClO_4_ in EC: DEC (1:1) | 30 | 33.30 | 110.00 | 0.00 |
|  | PHC1400 | 1400 | 2 | 5 | 0.381 | 1.890 | 40.00 | - | 1.850 | 0.0300 | 1 M NaClO_4_ in EC: DEC (1:1) | 30 | 85.40 | 370.00 | 231.69 |
|  | PHC1600 | 1600 | 2 | 5 | 0.375 | 1.670 | 4.00 | - | 1.910 | 0.0100 | 1 M NaClO_4_ in EC: DEC (1:1) | 30 | 83.50 | 346.00 | 240.00 |
| 216 | P1300 | 1300 | 2 | 5 | 0.376 | 1.050 | 33.10 | 1.806 | - | 0.0300 | 1 M NaClO_4_ in PC | 10 | 74.30 | 222.90 | 119.89 |
|  | P1500 | 1500 | 2 | 5 | 0.368 | 1.110 | 16.90 | 2.080 | - | 0.0240 | 1 M NaClO_4_ in PC | 10 | 74.90 | 271.14 | 177.40 |
| 217 | HPC-2-2 | 800 | 1 | 5 | 0.390 | 1.890 | 1430.00 | - | 0.780 | 0.8400 | 1 M NaClO_4_ in EC: DEC (1:1) | 100 | 30.53 | 174.00 | 0.00 |
|  | HPC-2-4 | 800 | 1 | 5 | 0.390 | 1.940 | 1580.00 | - | 0.690 | 1.0900 | 1 M NaClO_4_ in EC: DEC (1:1) | 100 | 29.19 | 174.00 | 0.00 |
| 218 | mPFR-HCA | 800 | 4 | 1 | 0.336 | 0.943 | 664.00 | - | - | - | 1 M NaClO_4_ in EC: PC (1:1) | 100 | 32.89 | 245.00 | 0.00 |
| 219 | HMNC-700 | 700 | 2 | 5 | 0.395 | 0.980 | 677.70 | - | - | 0.7920 | 1 M NaClO_4_ in EC: DEC (1:1) | 100 | 43.70 | 427.00 | 0.00 |
| 220 | NCG700 | 700 | 2 | 10 | 0.360 | 0.980 | 94.10 | - | - | 0.1200 | 1 M NaClO_4_ in EC: DEC (1:1) | 30 | 50.00 | 336.00 | 0.00 |
| 221 | C@G | 700 | 2 | - | 0.375 | 0.934 | 488.00 | - | - | - | 1 M NaClO_4_ in EC: DEC (1:1) | 50 | 44.00 | 355.00 | 0.00 |
| 222 | F-CP | 1100 | 5 | 5 | 0.400 | 1.010 | 46.40 | - | - | - | 1 M NaClO_4_ in EC: DEC (1:1) | 200 | 52.30 | 230.00 | 100.00 |
| 223 | Suc-1100°C | 1100 | 6 | - | 0.375 | 2.090 | 24.80 | 2.540 | 1.150 | 0.0152 | 1 M NaPF_6_ in EC: DEC (1:1) | 40 | 72.50 | 339.60 | 110.66 |
|  | Suc-1400°C | 1400 | 6 | - | 0.372 | 1.840 | 8.00 | 3.180 | 1.170 | 0.0080 | 1 M NaPF_6_ in EC: DEC (1:1) | 40 | 85.20 | 314.10 | 178.13 |
|  | Suc-1600°C | 1600 | 6 | - | 0.371 | 1.440 | 5.00 | 3.500 | 1.190 | 0.0059 | 1 M NaPF_6_ in EC: DEC (1:1) | 40 | 84.50 | 300.00 | 176.95 |
| 224 | PAN-CNFs-1250 | 1250 | 0.5 | - | 0.367 | 0.970 | 14.60 | 5.050 | 2.020 | - | 1 M NaClO_4_ in EC: DMC (1:1) | 20 | 72.00 | 271.00 | 125.00 |
| 225 | CDC-F-900 | 900 | 2 | 5 | 0.401 | 0.890 | 294.45 | 1.611 | 0.241 | 0.4300 | 1 M NaClO_4_ in EC: DEC: EMC (1:1:1) with 2% FEC | 100 | 55.40 | 290.79 | 0.00 |
| 226 | NAC-1000 | 1000 | - | 5 | 0.371 | 1.190 | 5.46 | - | - | 0.0340 | 1 M NaPF_6_ in DME | 50 | 64.70 | 223.10 | 72.73 |
|  | NAC-1200 | 1200 | - | 5 | 0.394 | 1.090 | 7.07 | - | - | 0.0400 | 1 M NaPF_6_ in DME | 50 | 74.20 | 371.30 | 142.98 |
|  | NAC-1400 | 1400 | - | 5 | 0.373 | 0.960 | 6.84 | - | - | 0.0360 | 1 M NaPF_6_ in DME | 50 | 77.50 | 288.90 | 142.00 |
| 227 | NPHC41-1100 | 1100 | 2 | 5 | 0.393 | 1.020 | 2.13 | 4.778 | 0.888 | - | 1 M NaPF_6_ in EC: DEC (1:1) | 15 | 68.27 | 313.00 | 205.90 |
| 228 | HHC1 | 1500 | 2 | 2 | 0.370 | 1.834 | 8.34 | 8.170 | 1.670 | - | 1 M NaPF_6_ in EC: DEC (1:1) | 20 | 74.00 | 334.90 | 256.80 |
|  | HHC12 | 1500 | 2 | 2 | 0.380 | 1.908 | 2.83 | 7.490 | 1.580 | - | 1 M NaPF_6_ in EC: DEC (1:1) | 20 | 75.20 | 369.20 | 285.80 |
| 229 | CAC | 1300 | 2 | 5 | 0.384 | 2.410 | 1656.70 | - | - | 0.7740 | 1 M NaClO_4_ in EC: DEC (1:1) | 30 | 19.40 | 101.80 | 0.00 |
| 230 | PFCC@PP | 1300 | 2 | - | 0.382 | 1.460 | 5.80 | - | - | 0.0480 | 1 M NaClO_4_ in EC: DEC (1:1) | 30 | 87.80 | 309.30 | 208.00 |
| 231 | L-700-1300 | 1300 | 2 | - | 0.381 | 1.620 | 9.40 | 4.110 | 1.030 | 0.0270 | 1 M NaPF_6_ in diglyme | 30 | 83.50 | 244.70 | 133.73 |
|  | LK-650-1300 | 1300 | 2 | - | 0.392 | 1.670 | 56.60 | 3.890 | 0.990 | 0.0510 | 1 M NaPF_6_ in diglyme | 30 | 86.00 | 301.40 | 201.81 |
| 232 | HC | 950 | 6 | 5 | 0.369 | 2.150 | 407.00 | - | - | 0.3000 | 1 M NaClO_4_ in EC: PC (1:1) | 50 | 47.33 | 156.18 | 15.00 |
|  | EHC | 950 | 6 | 5 | 0.365 | 2.530 | 19.00 | - | - | 0.0300 | 1 M NaClO_4_ in EC: PC (1:1) | 50 | 60.17 | 270.75 | 84.12 |
| 233 | HHC | 800 | 3 | 2 | 0.380 | 2.670 | 7.00 | - | - | 0.0100 | 1 M NaPF_6_ in diglyme | 20 | 70.20 | 279.30 | 0.00 |
|  | TZ-PS-800 | 800 | 3 | 2 | 0.362 | 2.530 | 2.00 | - | - | - | 1 M NaPF_6_ in diglyme | 20 | 58.30 | 165.00 | 0.00 |
|  | CO-PS-800 | 800 | 3 | 2 | 0.383 | 2.790 | 445.00 | - | - | 0.2500 | 1 M NaPF_6_ in diglyme | 20 | 46.00 | 132.10 | 0.00 |
| 234 | HC-HT160 | 1600 | 2 | - | 0.346 | 1.600 | 13.28 | 0.810 | 0.940 | - | 1 M NaOT_f_ in diglyme | 100 | 80.00 | 262.10 | 200.00 |
|  | HC-HT190 | 1600 | 2 | - | 0.346 | 1.710 | 15.95 | 0.740 | 0.900 | - | 1 M NaOT_f_ in diglyme | 100 | 79.60 | 287.50 | 223.70 |
| 235 | Coal | 1200 | 2 | 5 | 0.375 | 1.032 | 120.60 | - | - | 0.0840 | 1 M NaPF_6_ in DEGDME | 30 | 79.00 | 251.06 | 109.04 |
|  | CG0.1 | 1200 | 2 | 5 | 0.387 | 0.967 | 18.20 | - | - | 0.0210 | 1 M NaPF_6_ in DEGDME | 30 | 84.00 | 294.50 | 152.00 |
|  | Glucose | 1200 | 2 | 5 | 0.399 | 1.013 | 22.10 | - | - | 0.0300 | 1 M NaPF_6_ in DEGDME | 30 | 75.00 | 279.47 | 109.33 |
| 236 | UHC | 1300 | 3 | 5 | 0.380 | 1.200 | 2.78 | - | 2.110 | 0.0035 | 1 M NaPF_6_ in DEGDME | 20 | 83.49 | 269.41 | 184.91 |
|  | RHC | 1300 | 3 | 5 | 0.370 | 1.260 | 19.97 | - | 2.250 | 0.0012 | 1 M NaPF_6_ in DEGDME | 20 | 86.54 | 308.36 | 206.58 |
|  | PHC | 1300 | 3 | 5 | 0.370 | 1.270 | 4.01 | - | 2.610 | 0.0061 | 1 M NaPF_6_ in DEGDME | 20 | 85.96 | 284.32 | 194.97 |
|  | SHC | 1300 | 3 | 5 | 0.370 | 1.240 | 3.43 | - | 2.150 | 0.0060 | 1 M NaPF_6_ in DEGDME | 20 | 82.80 | 264.30 | 178.72 |
| 237 | TS-1400 | 1400 | 2 | - | 0.379 | 1.230 | 2.80 | 3.470 | 1.030 | - | 1 M NaPF_6_ in diglyme | 30 | 84.69 | 261.60 | 140.77 |
|  | THFS-1400 | 1400 | 2 | - | 0.382 | 1.320 | 4.10 | 3.430 | 1.040 | - | 1 M NaPF_6_ in diglyme | 30 | 86.80 | 296.80 | 195.58 |
| 238 | TC-1000 | 1000 | 4 | 3 | 0.375 | 2.210 | 2095.55 | 1.758 | 1.199 | 0.1400 | 1 M NaPF_6_ in diglyme | 100 | 68.00 | 354.00 | 0.00 |
|  | Ti-900 | 900 | 4 | 3 | 0.393 | 2.260 | 2788.23 | 0.986 | 0.871 | 1.3000 | 1 M NaPF_6_ in diglyme | 100 | 80.70 | 234.00 | 0.00 |
| 239 | P-HC | 1300 | 2 | 5 | 0.379 | 1.790 | 10.79 | 2.024 | 0.944 | - | 1 M NaPF_6_ in diglyme | 20 | 80.30 | 303.30 | 199.42 |
|  | R-HC | 1300 | 2 | 5 | 0.371 | 1.570 | 7.34 | 2.381 | 1.024 | - | 1 M NaPF_6_ in diglyme | 20 | 82.10 | 334.30 | 225.15 |
|  | T-HC | 1300 | 2 | 5 | 0.366 | 1.330 | 3.51 | 2.373 | 1.103 | - | 1 M NaPF_6_ in diglyme | 20 | 76.50 | 240.30 | 99.42 |
| 240 | L1300 | 1300 | 3 | 2 | 0.394 | 1.970 | 380.14 | 2.760 | 1.020 | 0.0990 | 1 M NaClO_4_ in EC: DEC (1:1) | 20 | 72.30 | 324.90 | 198.26 |
|  | LS1300 | 1300 | 3 | 2 | 0.390 | 2.160 | 132.09 | 2.810 | 1.010 | 0.0300 | 1 M NaClO_4_ in EC: DEC (1:1) | 20 | 75.40 | 356.90 | 215.70 |
|  | S1300 | 1300 | 3 | 2 | 0.382 | 2.310 | 44.61 | 2.970 | 0.920 | 0.0070 | 1 M NaClO_4_ in EC: DEC (1:1) | 20 | 82.20 | 306.80 | 155.23 |
| 241 | PHC-1300 | 1300 | 2 | 5 | 0.382 | 1.230 | 1.07 | 4.897 | 0.953 | - | 1 M NaPF_6_ in EC: DEC (1:1) | 15 | 86.35 | 359.14 | 225.89 |
|  | HC-1300 | 1300 | 2 | 5 | 0.366 | 1.200 | 1.01 | 5.929 | 0.906 | - | 1 M NaPF_6_ in EC: DEC (1:1) | 15 | 72.68 | 239.43 | 128.27 |
| 242 | CFR | 1300 | 2 | 2 | 0.380 | 1.340 | 7.32 | 3.812 | 0.922 | 0.0124 | 1 M NaPF_6_ in diglyme | 30 | 85.66 | 303.86 | 191.85 |
|  | CFR-PVB3 | 1300 | 2 | 2 | 0.386 | 1.570 | 10.54 | 3.681 | 0.914 | 0.0308 | 1 M NaPF_6_ in diglyme | 30 | 88.50 | 367.00 | 260.00 |
|  | CFR-PVB6 | 1300 | 2 | 2 | 0.392 | 1.600 | 430.46 | 3.486 | 0.928 | 0.0181 | 1 M NaPF_6_ in diglyme | 30 | 58.49 | 119.74 | 46.99 |
| 243 | PAO-300-10 | 1200 | 2 | 5 | 0.369 | 1.670 | 12.08 | - | 1.760 | - | 1 M NaClO_4_ in EC: DEC (1:1) | 30 | 69.70 | 275.00 | 122.69 |
| 244 | LPP-2-1400 | 1400 | 2 | - | 0.376 | 1.060 | 5.60 | - | - | - | 1 M NaPF_6_ in EC: DMC (1:1) | 30 | 89.00 | 307.00 | 174.03 |
|  | LPP-2-1600 | 1600 | 2 | - | 0.360 | 1.020 | 11.60 | - | - | - | 1 M NaPF_6_ in EC: DMC (1:1) | 30 | 83.00 | 234.00 | 153.31 |
|  | LPP-1-1400 | 1400 | 2 | - | 0.385 | 1.080 | 16.50 | - | - | - | 1 M NaPF_6_ in EC: DMC (1:1) | 30 | 82.00 | 280.00 | 165.75 |
|  | LPP-3-1400 | 1400 | 2 | - | 0.361 | 1.010 | 18.50 | - | - | - | 1 M NaPF_6_ in EC: DMC (1:1) | 30 | 84.00 | 260.00 | 150.00 |
| 245 | VHC-800 | 800 | 3 | 10 | 0.387 | 1.966 | 114.27 | 1.328 | 0.929 | - | 1 M NaPF_6_ in diglyme | 25 | 66.43 | 249.00 | 70.84 |
|  | VHC-900 | 900 | 3 | 10 | 0.386 | 1.749 | 89.59 | 1.675 | 0.912 | - | 1 M NaPF_6_ in diglyme | 25 | 67.21 | 224.52 | 70.84 |
|  | VHC-1200 | 1200 | 3 | 10 | 0.380 | 1.338 | 227.57 | 1.724 | 0.947 | - | 1 M NaPF_6_ in diglyme | 25 | 72.43 | 293.73 | 139.51 |
|  | VHC-1300 | 1300 | 3 | 10 | 0.367 | 1.269 | 287.21 | 2.121 | 0.980 | - | 1 M NaPF_6_ in diglyme | 25 | 72.35 | 196.19 | 98.09 |

**Reference**

1. Li, Y. et al. Dual-functionalized Ca enables high sodiation kinetics for hard carbon in sodium-ion batteries. *ACS Appl. Mater. Interfaces* **16**, 2397-2407 (2024).
2. Sun, S. & Wang, L. Eucommia ulmoides barks-derived anodes for sodium ion battery and method to improve electrochemical performances by modifying defects. *Electron. Mater. Lett.* **20**, 474-483 (2024).
3. Wu, Z. et al. Hierarchical flower-like porous carbon nanosheets with richly exposed (002) planes for high-performance sodium-ion batteries. *ACS Appl. Nano Mater.* **7**, 433-443 (2024).
4. Zhao, B. et al. Regulating oxygen functionalities of cellulose-derived hard carbon toward superior sodium storage. *J. Mater. Chem. A* **12**, 5834-5845 (2024).
5. Zhang, Z. et al. Tuning nanopore structure of hard carbon anodes by zinc gluconate for high capacity sodium ion batteries. *Batteries Supercaps* **7**, e202300552 (2024).
6. Feng, X. et al. Unlocking the local structure of hard carbon to grasp sodium-ion diffusion behavior for advanced sodium-ion batteries. *Energy Environ. Sci.* **17**, 1387-1396 (2024).
7. Zou, J. et al. A superior sodium-ion anode material from PAN/Lignin-derived carbon nanofibers with inter-connected structure prepared through phosphoric acid induced cross-linking. *J. Power Sources* **593**, 233994 (2024).
8. Li, J. et al. Advanced carbon based materials for fabrications of sodium ion hybrid capacitors with high electrochemical performance. *Electrochem.* **91**, 057003 (2023).
9. Wang, T. et al. Agar-derived slope-dominated carbon anode with puparium like nano-morphology for cost-effective SIBs. *Small* **20**, 2309809 (2023).
10. Chen, R. et al. Amine-aldehyde condensation-derived N-doped hard carbon microspheres for high-capacity and robust sodium storage. *Small* **19**, 2303790 (2023).
11. Zhang, Z. et al. An effective strategy to prepare non-graphitic carbon with increased pseudo-graphitic content for sodium-ion battery anode with enhanced plateau capacity. *Chem. Eng. J.* **477**, 147188 (2023).
12. Escamilla-Perez, A. M. et al. Biopolymer-based hard carbons: Correlations between properties and performance as a Na-ion battery anode. *ACS Appl. Energy Mater.* **6**, 7419-7432 (2023).
13. Zhu, Y. et al. Carbon nanosheet with controlled porous and disorganized structures as high-rate anode for high-energy Na-ion hybrid capacitors. *Electrochim. Acta* **473**, 143497 (2024).
14. Qin, L. et al. Cellulose as a novel precursor to construct high-performance hard carbon anode toward enhanced sodium-ion batteries. *Diam. Relat. Mater.* **136**, 110065 (2023).
15. Xu, Y. et al. Constructing abundant oxygen-containing functional groups in hard carbon derived from anthracite for high-performance sodium-ion batteries. *Nanomaterials* **13**, 3002 (2023).
16. Chen, X. et al. Cow leather-derived N/O co doped hard carbon as a high-performance anode material for sodium-ion batteries. *Mater. Today Commun.* **37**, 107361 (2023).
17. Sun, N. et al. Design advanced nitrogen/oxygen co-doped hard carbon microspheres from phenolic resin with boosted Na-storage performance. *J. Power Sources* **564**, 232879 (2023).
18. Yu, C. et al. Engineering homotype heterojunctions in hard carbon to induce stable solid electrolyte interfaces for sodium-ion batteries. *Carbon Energy* **5**, e220 (2023).
19. Cheng, D. et al. Engineering ultrathin carbon layer on porous hard carbon boosts sodium storage with high initial coulombic efficiency. *ACS Nano* **17**, 19063-19075 (2023).
20. Cui, Y. et al. Enhancing high-capacity and high-rate sodium-ion storage through synergistic N,S dual doping of hard carbon. *Chem. Asian J*. **18**, e202300449 (2023).
21. Fan, C. et al. Epoxy phenol novolac resin: A novel precursor to construct high performance hard carbon anode toward enhanced sodium-ion batteries. *Carbon* **205**, 353-364 (2023).
22. Jang, H. et al. Exploring the storage mechanism of alkali ions in non-graphitic hard carbon anodes. *J. Electrochem. Soc.* **170**, 090538 (2023).
23. Tong, Y., Wu, Y., Liu, Z., Yin, Y., Sun, Y. & Li H. Fabricating multi-porous carbon anode with remarkable initial coulombic efficiency and enhanced rate capability for sodium-ion batteries. *Chin. Chem. Lett.* **34**, 107443 (2023).
24. Chen, X. et al. Filling carbon: a microstructure-engineered hard carbon for efficient alkali metal ion storage. *Energy Environ. Sci.* **16**, 4041-4053 (2023).
25. Zhang, K.-Y. et al. Flaky N-doped hard carbon anode material for sodium-ion batteries. *Phys. Scripta* **98**, 125977 (2023).
26. Chen, J. et al. Flexible carbon nanofibers with multichannel and porous structure as freestanding anode for excellent sodium storage. *J. Energy Storage* **79**, 110072 (2024).
27. Zhang, H. et al. Flexible precursor modulation toward selective heteroatom doping in a hard-carbon anode for sodium-ion batteries. *Energy Fuels* **37**, 15127-15137 (2023).
28. Xu, R. et al. Hard carbon anodes derived from phenolic resin/sucrose cross-linking network for high-performance sodium-ion batteries. *Battery Energy* **2**, 20220054 (2023).
29. Huang, G., Kong, Q., Yao, W. & Wang, Q. High proportion of active nitrogen-doped hard carbon based on mannich reaction as anode material for high-performance sodium-ion batteries. *ChemSusChem* **16**, e202202070 (2023).
30. Verma, B., Raj, H., Rajput, H. & Sil, A. Insights into the electrochemical properties of bagasse-derived hard carbon anode materials for sodium-ion battery. *Ionics* **29**, 5205-5216 (2023).
31. Liu, M. et al. Interfacial-catalysis-enabled layered and inorganic-rich SEI on hard carbon anodes in ester electrolytes for sodium-ion batteries. *Adv. Mater.* **35**, 2300002 (2023).
32. Guo, Z. et al. Investigating the superior performance of hard carbon anodes in sodium-ion compared with lithium- and potassium-ion batteries. *Adv. Mater*. **35**, 2304091 (2023).
33. Wang, H. et al. Mechanochemistry transforming high-surface-area coal-based activated carbon into densified carbon with optimized multi-scale structures for enhanced sodium/potassium ion storage. *Electrochim. Acta* **475**, 143579 (2024).
34. Zhou, H. et al. Microstructure regulation of resin-based hard carbons via esterification cross-linking for high-performance sodium-ion batteries. *Inorg. Chem. Front*. **10**, 2404-2413 (2023).
35. Sun, C. et al. Modulating electronic structure with heteroatom dopants in porous hard carbon towards ultralong lifespan sodium-ion batteries. *Chem. Eng. J.* **470**, 144419 (2023).
36. Song, N.-J. et al. Modulating the graphitic domains and pore structure of corncob-derived hard carbons by pyrolysis to improve sodium storage. *Molecules* **28**, 3595 (2023).
37. Rao, Y.B., Saisrinu, Y., Khatua, S., Bharathi, K.K. & Patro, L.N. Nitrogen doped soap-nut seeds derived hard carbon as an efficient anode material for Na-ion batteries. *J. Alloys Compd.* **968**, 171917 (2023).
38. Zhao, Q. et al. Nitrogen/oxygen/sulfur tri-doped hard carbon nanospheres derived from waste tires with high sodium and potassium anodic performances. *Inorg. Chem. Front.* **10**, 2574-2585 (2023).
39. Wu, S. et al. Nitrogen/phosphorus co-doped ultramicropores hard carbon spheres for rapid sodium storage. *Carbon* **218**, 118756 (2024).
40. Zhao, Y. et al. Novel structural design and adsorption/insertion coordinating quasi-metallic Na storage mechanism toward high-performance hard carbon anode derived from carboxymethyl cellulose. *Small* **19**, 2303296 (2023).
41. Chen, H., Sun, N., Wang, Y., Soomro, R.A. & Xu, B. One stone two birds: Pitch assisted microcrystalline regulation and defect engineering in coal-based carbon anodes for sodium-ion batteries. *Energy Storage Mater.* **56**, 532-541 (2023).
42. Beda, A., Rabuel, F., Rahmouni, O., Morcrette, M. & Ghimbeu, C.M. Optimization of tannin-derived hard carbon spheres for high-performance sodium-ion batteries. *J. Mater. Chem. A* **11**, 4365-4383 (2023).
43. Wu, S. et al. P-doped hard carbon microspheres for sodium-ion battery anodes with superior rate and cyclic performance. *Inorg. Chem. Front.* **10**, 5908-5916 (2023).
44. Deng, L. et al. Phosphate-induced reaction to prepare coal-based P-doped hard carbon with a hierarchical porous structure for improved sodium-ion storage. *Molecules* **28**, 4921 (2023).
45. Ding, H. et al. Pre-crosslinking enables a promising cyclodextrin-derived carbon anode with large plateau capacity for sodium ion batteries. *New J. Chem.* **48**, 1182-1191 (2024).
46. Chen, Y. et al. Pre-oxidation strategy transforming waste foam to hard carbon anodes for boosting sodium storage performance. *Small* **20**, 2307132 (2023).
47. Huang, Y. et al. Rationally designing closed pore structure by carbon dots to evoke sodium storage sites of hard carbon in low-potential region. *Adv. Funct. Mater*. **34**, 2308392 (2023).
48. Wang, H. et al. Recycling of waste coal-based filter materials into carbon anode materials for potassium and sodium-ion batteries. *Mater. Today Sustain*. **24**, 100578 (2023).
49. Gao, Y. et al. Recycling spent masks to fabricate flexible hard carbon anode toward advanced sodium energy storage. *J. Electroanal. Chem*. **941**, 117525 (2023).
50. Wang, J., Li, F., Duan, Y., Tao, H. & Yang, X. Sawdust-derived hard carbon as a high-performance anode for sodium-ion batteries. *Ionics* **29**, 2311-2318 (2023).
51. Lee, G. et al. Selective synthesis of soft and hard carbons from a single precursor through tailor-made stabilization for anode in sodium-ion batteries. *Chem. Eng. J.* **479**, 147766 (2024).
52. Yu, L. et al. Study of stable sodium ion storage in porous carbon derived from puffball biomass. *Ind. Crops Prod.* **208**, 117805 (2024).
53. Lu, B. et al. Synthesis of low-cost and high-performance dual-atom doped carbon-based materials with a simple green route as anodes for sodium-ion batteries. *Molecules* **28**, 7314 (2023).
54. Shi, L. et al. Tailoring the microstructure and solid electrolyte interface of hard carbon to realize high-initial-coulombic-efficiency and high-rate sodium storage. *Electrochim. Acta* **459**, 142557 (2023).
55. Cheng, Y. et al. The effect of thermal treatment temperature on the crystal structure and electrochemical performance of the coconut shell-based hard carbon. *Solid State Ionics* **402**, 116374 (2023).
56. Zhang, S., Sun, N., Jiang, M., Soomro, R.A. & Xu, B. Trash to treasure: Sulfonation-assisted transformation of waste masks into high-performance carbon anode for sodium-ion batteries. *Carbon* **209**, 118034 (2023).
57. Lou, X. et al. Understanding the improved sodium ion storage in wood-derived hard carbon anodes by hydrogen treatment. *J. Electrochem. Soc.* **170**, 120510 (2023).
58. Sun, H. et al. Unraveling the effect of carbon morphology evolution in hard carbons on sodium storage performance. *Inorg. Chem. Front.* **10**, 6547-6556 (2023).
59. Nagmani & Puravankara, S. Utilization of PET derived hard carbon as a battery-type, higher plateau capacity anode for sodium-ion and potassium-ion batteries. *J. Electroanal. Chem.* **946**, 117731 (2023).
60. Lu, Z. et al. Zinc single-atom-regulated hard carbons for high-rate and low-temperature sodium-ion batteries. *Adv. Mater.* **35**, 2211461 (2023).
61. Yuan, M. et al. A general multi-interface strategy toward densified carbon materials with enhanced comprehensive electrochemical performance for Li/Na-ion batteries. *Small* **18**, 2105738 (2022).
62. Muruganantham, R., Wang, F.-M. & Liu, W.-R. A green route N, S-doped hard carbon derived from fruit-peel biomass waste as an anode material for rechargeable sodium-ion storage applications. *Electrochim. Acta* **424**, 140573 (2022).
63. Zhao, Y., Ye, J., Zhang, P., Li, Z. & Zhao, H. Abnormal preferential oxygen functionalization on the surface of soft/hard carbon for sodium storage. *Appl. Surf. Sci.* **602**, 154336 (2022).
64. Xie, F. et al. Achieving high initial Coulombic efficiency for competent Na storage by microstructure tailoring from chiral nematic nanocrystalline cellulose. *Carbon Energy* **4**, 914-923 (2022).
65. Jin, Q. et al. Agricultural wastes for full-cell sodium-ion batteries: Engineering biomass components to maximize the performance and economic prospects. *ACS Sustain. Chem. Eng.* **11**, 536-546 (2022).
66. Chen, X. et al. An overall understanding of sodium storage behaviors in hard carbons by an "adsorption-intercalation/filling" hybrid mechanism. *Adv. Energy Mater*. **12**, 2200886 (2022).
67. Hou, Z. et al. Biomass-derived hard carbon with interlayer spacing optimization toward ultrastable Na-ion storage. *ACS Appl. Mater. Interfaces* **15**, 1367-1375 (2023).
68. Huang, S. et al. Boosting surface-dominated sodium storage of carbon anode enabled by coupling graphene nanodomains, nitrogen-doping, and nanoarchitecture engineering. *Adv. Funct. Mater.* **32**, 2203279 (2022).
69. Bobyleva, Z.V. et al. Caramelization as a key stage for the preparation of monolithic hard carbon with advanced performance in sodium-ion batteries. *ACS Appl. Energy Mater.* **6**, 181-190 (2022).
70. Surta, T.W. et al. Combining experimental and theoretical techniques to gain an atomic level understanding of the defect binding mechanism in hard carbon anodes for sodium ion batteries. *Adv. Energy Mater.* **12**, 2200647 (2022).
71. Li, X., Yang, C., Wang, S., Mao, X. & Yu, K. Comprehensive study on improving the sodium storage performance of low-defect biomass-derived carbon through S or N doping. *Diam. Relat. Mater.* **129**, 109382 (2022).
72. Sun, B. et al. Edge-enriched and S-doped carbon nanorods to accelerate electrochemical kinetics of sodium/potassium storage. *Carbon* **201**, 776-784 (2023).
73. Wang, P., Wang, H., Liang, C. & Yu, K. Effects of nitrogen and phosphorus co-doped layering on lithium/sodium-ion storage properties of biomass-derived carbonaceous electrode materials. *J. Alloys Compd*. **922**, 166233 (2022).
74. Yin, X. et al. Enabling fast Na^+^ transfer kinetics in the whole-voltage-region of hard-carbon anodes for ultrahigh-rate sodium storage. *Adv. Mater.* **34**, 2109282 (2022).
75. Cheng, D. et al. Engineering carbon nanosheets with hexagonal ordered conical macropores as high-performance sodium-ion battery anodes. *J. Colloid Interface Sci.* **625**, 978-989 (2022).
76. Liu, J. et al. Facile synthesis of high quality hard carbon anode from Eucalyptus wood for sodium-ion batteries. *Chem. Pap.* **76**, 7465-7473 (2022).
77. Zhou, C., Xing, G., Yang, H., Liu, S. & Meng, C. Facile synthesis of N/O dual-doped carbon spheres with excellent electrochemical performance. *Solid State Sci.* **132**, 106980 (2022).
78. Sun, J. et al. Gradient supramolecular preorganization endows the derived N/P dual-doped carbon nanosheets with tunable storage performance toward sodium-ion batteries. *Ind. Eng. Chem. Res.* **61**, 6997-7008 (2022).
79. Zhang, H., Zhang, W. & Huang, F. Graphene inducing graphitization: Towards a hard carbon anode with ultrahigh initial coulombic efficiency for sodium storage. *Chem. Eng. J.* **434**, 134503 (2022).
80. Sun, M. et al. Hierarchical porous and sandwich-like sulfur-doped carbon nanosheets as high-performance anodes for sodium-ion batteries. *Ind. Eng. Chem. Res.* **61**, 2126-2135 (2022).
81. Li, Q., Gao, Y. & Wen, H. Investigation of pyrolysed anthracite as an anode material for sodium ion batteries. *New J. Chem*. **46**, 13575-13581 (2022).
82. Nagmani, Verma, P. & Puravankara, S. Jute-fiber precursor-derived low-cost sustainable hard carbon with varying micro/mesoporosity and distinct storage mechanisms for sodium-ion and potassium-ion batteries. *Langmuir* **38**, 15703-15713 (2022).
83. Chen, H., Sun, N., Zhu, Q., Soomro, R.A. & Xu, B. Microcrystalline hybridization enhanced coal-based carbon anode for advanced sodium-ion batteries. *Adv. Sci.* **9**, 2200023 (2022).
84. Tang, S. et al. Microporous carbon nanospheres with fast sodium storage capability enabled by dominant capacitive behavior. *Langmuir* **38**, 7331-7340 (2022).
85. Shi, G., Han, Z., Hu, L., Wang, B. & Huang, F. N/O co-doped hard carbon derived from cocklebur fruit for sodium-ion storage. *ChemElectroChem* **9**, e202200138 (2022).
86. Gao, S. et al. Na storage activity or inertness of P-configurations in N, P dual doped carbon nanofibers: Bulk vs surface. *ACS Appl. Mater. Interfaces* **14**, 56836-56846 (2022).
87. Li, H.-J. et al. Nano casting construction of few-graphene-layers carbon with tunable layer spacing as ultra-stable anode for sodium-ion batteries. *Electrochim. Acta* **419**, 140423 (2022).
88. Gao, Y., Piao, S., Jiang, C. & Zou, Z. Navel orange peel-derived hard carbons as high performance anode materials of Na and Li-ion batteries. *Diam. Relat. Mater.* **129**, 109329 (2022).
89. Surendran, V., Hema, R.K., Hassan, M.S.O., Vijayan, V. & Shaijumon, M.M. Open or Closed? Elucidating the correlation between micropore nature and sodium storage mechanisms in hard carbon. *Batteries Supercaps* **5**, e202200316 (2022).
90. Wang, Z. et al. Phosphorus/sulfur co-doped hard carbon with a well-designed porous bowl-like structure and enhanced initial coulombic efficiency for high-performance sodium storage. *J. Alloys Compd.* **911**, 164979 (2022).
91. Xu, T., Qiu, X., Zhang, X. & Xia, Y. Regulation of surface oxygen functional groups and pore structure of bamboo-derived hard carbon for enhanced sodium storage performance. *Chem. Eng. J.* **452**, 139514 (2023).
92. Shao, W. et al. Replacing "Alkyl" with "Aryl" for inducing accessible channels to closed pores as plateau-dominated sodium-ion battery anode. *SusMat* **2**, 319-334 (2022).
93. Wu, X.-S., Dong, X.-L., Wang, B.-Y., Xia, J.-L. & Li, W.-C. Revealing the sodium storage behavior of biomass-derived hard carbon by using pure lignin and cellulose as model precursors. *Renew. Energy* **189**, 630-638 (2022).
94. Xie, F. et al. Screening heteroatom configurations for reversible sloping capacity promises high-power Na-ion batteries. *Angew. Chem. Int. Ed.* **61**, e202116394 (2022).
95. Wang, P. et al. Self-supported hard carbon anode from fungus-treated basswood towards sodium-ion batteries. *Nano Res.* **16**, 3832-3838 (2023).
96. Jeon, I. et al. Sodium storage behavior and long cycle stability of boron-doped carbon nanofibers for sodium-ion battery anodes. *Electrochim. Acta* **439**, 141730 (2023).
97. Yuan, M. et al. Sodium storage mechanism of nongraphitic carbons: A general model and the function of accessible closed pores. *Chem. Mater.* **34**, 3489-3500 (2022).
98. Long, B. et al. Stabilization residual oxygen reduces sulfur activity in hard carbon anode for sodium-ion batteries. *J. Mater. Sci.* **57**, 17711-17721 (2022).
99. Dong, R. et al. Tailoring defects in hard carbon anode towards enhanced Na storage performance. *Energy Mater. Adv.* **2022**, 9896218 (2022).
100. Xu, Z. et al. The role of hydrothermal carbonization in sustainable sodium-ion battery anodes. *Adv. Energy Mater.* **12**, 2200208 (2022).
101. Song, M. et al. Towards enhanced sodium storage of hard carbon anodes: Regulating the oxygen content in precursor by low-temperature hydrogen reduction. *Energy Storage Mater.* **51**, 620-629 (2022).
102. Beda, A., Escamilla-Perez, A.M., Simonin, L. & Ghimbeu, C.M. Vegetal-extracted polyphenols as a natural hard carbon anode source for Na-ion batteries. *ACS Appl. Energy Mater.* **5**, 4774-4787 (2022).
103. Cui, X.-Y. et al. A carbon-based material with a hierarchical structure and intrinsic heteroatom sites for sodium-ion storage with ultrahigh rate and capacity. *Nanoscale* **13**, 15731-15742 (2021).
104. Kumar, U., Wu, J., Sharma, N. & Sahajwalla, V. Biomass derived high areal and specific capacity hard carbon anodes for sodium-ion batteries. *Energy Fuels* **35**, 1820-1830 (2021).
105. Senthil, C., Park, J.W., Shaji, N., Sim, G.S. & Lee, C.W. Biomass seaweed-derived nitrogen self-doped porous carbon anodes for sodium-ion batteries: Insights into the structure and electrochemical activity. *J. Energy Chem.* **64**, 286-295 (2022).
106. Xu, R. et al. Boosting sodium storage performance of hard carbons by regulating oxygen functionalities of the cross-linked asphalt precursor. *Carbon* **206**, 94-104 (2023).
107. Fan, L. et al. Boosting the high capacitance-controlled capacity of hard carbon by using surface oxygen functional groups for fast and stable sodium storage. *ACS Appl. Energy Mater.* **4**, 11436-11446 (2021).
108. Wang, Y., Wang, K., Yong, B., Deng, L. & Zhang, P. Constructing advanced high-performance sodium-ion batteries anode materials via the morphology tuning strategy of lignin-derived carbon. *J. Mater. Res.* **36**, 3460-3471 (2021).
109. Chen, D. et al. Direct conversion of ester bond-rich waste plastics into hard carbon for high-performance sodium storage. *Carbon* **173**, 253-261 (2021).
110. Feng, X. et al. Effect of different nitrogen configurations on sodium storage properties of carbon anodes for sodium ion batteries. *ACS Appl. Mater. Interfaces* **13**, 56285-56295 (2021).
111. Guo, H.-Y. et al. Effect of the air oxidation stabilization of pitch on the microstructure and sodium of hard carbons. *New Carbon Mater.* **36**, 1073-1080 (2021).
112. Cheng, D. et al. Electrochemical storage mechanism of sodium in carbon materials: A study from soft carbon to hard carbon. *Carbon* **182**, 758-769 (2021).
113. Zhu, Z. et al. Elucidating electrochemical intercalation mechanisms of biomass-derived hard carbon in sodium-/potassium-ion batteries. *Carbon Energy* **3**, 541-553 (2021).
114. Yang, B. et al. Engineering hard carbon with high initial coulomb efficiency for practical sodium-ion batteries. *J. Power Sources* **492**, 229656 (2021).
115. Chen, C. et al. Experimental design and theoretical evaluation of nitrogen and phosphorus dual-doped hierarchical porous carbon for high-performance sodium-ion storage. *J. Mater. Sci. Technol.* **76**, 11-19 (2021).
116. Yang, J. et al. From micropores to ultra-micropores inside hard carbon: Toward enhanced capacity in room-/low-temperature sodium-ion storage. *Nano-Micro Lett.* **13**, 98-98 (2021).
117. Zhu, Z. et al. Green energy application technology of litchi pericarp-derived carbon material with high performance. *J. Clean. Prod.* **286**, 124960 (2021).
118. Zhang, H., Zhang, W. & Huang, F. Hard carbon microsphere with expanded graphitic interlayers derived from a highly branched polymer network as ultrahigh performance anode for practical sodium-ion batteries. *ACS Appl. Mater. Interfaces* **13**, 61180-61188 (2021).
119. Kumaresan, T.K., Masilamani, S.A., Raman, K., Karazhanov, S.Z. & Subashchandrabose, R. High performance sodium-ion battery anode using biomass derived hard carbon with engineered defective sites. *Electrochim. Acta* **368**, 137574 (2021).
120. Yin, B. et al. Increasing accessible subsurface to improving rate capability and cycling stability of sodium-ion batteries. *Adv. Mater.* **33**, 2100808 (2021).
121. Yu, K., Wang, X., Yang, H., Bai, Y. & Wu, C. Insight to defects regulation on sugarcane waste-derived hard carbon anode for sodium-ion batteries. *J. Energy Chem.* **55**, 499-508 (2021).
122. Ren, Q. et al. Manipulating free-standing, flexible and scalable microfiber carbon papers unlocking ultra-high initial Coulombic efficiency and storage sodium behavior. *Chem. Eng. J.* **425**, 131656 (2021).
123. Lou, Z. et al. Microcrystalline regulation of bituminous coal derived hard carbon by pre-oxidation strategy for improved sodium-ion storage. *Fuel* **310**, 122072 (2022).
124. Kim, J.-B. et al. Microstructural investigation into Na-ion storage behaviors of cellulose-based hard carbons for Na-ion batteries. *J. Phys. Chem. C* **125**, 14559-14566 (2021).
125. Li, X. et al. One-pot fabrication of pitch-derived soft carbon with hierarchical porous structure and rich sp^2^ carbon for sodium-ion battery. *J. Mater. Sci. Mater. Electron.* **32**, 21944-21956 (2021).
126. Sun, W. et al. Sodium hypophosphite-assist pyrolysis of coal pitch to synthesis P-doped carbon nanosheet anode for ultrafast and long-term cycling sodium-ion batteries. *J. Alloys Compd.* **889**, 161678 (2021).
127. Yin, R. et al. Structural evaluation of coal-tar-pitch-based carbon materials and their Na^+^ storage properties. *Coatings* **11**, 948 (2021).
128. Cheng, H., Tang, Z., Luo, X. & Zheng, Z. Spartina alterniflora-derived porous carbon using as anode material for sodium-ion battery. *Sci. Total Environ.* **777**, 146120 (2021).
129. Zhang, G. et al. Tailoring a phenolic resin precursor by facile pre-oxidation tactics to realize a high-initial-Coulombic-efficiency hard carbon anode for sodium-ion batteries. *ACS Appl. Mater. Interfaces* **13**, 31650-31659 (2021).
130. Li, R.-R. et al. Temperature-regulated biomass-derived hard carbon as a superior anode for sodium-ion batteries. *Mater. Chem. Front.* **5**, 7595-7605 (2021).
131. Huang, S. et al. Three-dimensional hierarchical porous hard carbon for excellent sodium/potassium storage and mechanism investigation. *Mater. Today Energy* **20**, 100673 (2021).
132. Yasin, G. et al. A novel strategy for the synthesis of hard carbon spheres encapsulated with graphene networks as a low-cost and large-scalable anode material for fast sodium storage with an ultralong cycle life. *Inorg. Chem. Front.* **7**, 402-410 (2020).
133. Chen, Z. et al. Activation-free N-doped porous carbon to enhance surface-driven K storage vs intercalation dominated Na storage. *Appl. Surf. Sci.* **506**, 144909 (2020).
134. Ghosh, S. et al. Binder less-integrated freestanding carbon film derived from pitch as light weight and high-power anode for sodium-ion battery. *Electrochim. Acta* **353**, 136566 (2020).
135. Xiao, N. et al. Boosting the sodium storage performance of coal-based carbon materials through structure modification by solvent extraction. *Carbon* **162**, 431-437 (2020).
136. Lu, M., Huang, Y. & Chen, C. Cedarwood bark-derived hard carbon as an anode for high-performance sodium-ion batteries. *Energy Fuels* **34**, 11489-11497 (2020).
137. Wang, H. et al. Chemical grafting-derived N, P co-doped hollow microporous carbon spheres for high-performance sodium-ion battery anodes. *Appl. Surf. Sci.* **518**, 146221 (2020).
138. Xiao, W. et al. Engineering surface oxygenated functionalities on commercial carbon toward ultrafast sodium storage in ether-based electrolytes. *ACS Appl. Mater. Interfaces* **12**, 37116-37127 (2020).
139. Zhang, X. et al. Extended low-voltage plateau capacity of hard carbon spheres anode for sodium ion batteries. *J. Power Sources* **476**, 228550 (2020).
140. Xu, F. et al. Facile regulation of carbon frameworks from microporous to low-porous via molecular crosslinker design and enhanced Na storage. *Carbon* **167**, 896-905 (2020).
141. Yu, Z.-E. et al. Hard carbon micro-nano tubes derived from kapok fiber as anode materials for sodium-ion batteries and the sodium-ion storage mechanism. *Chem. Commun.* **56**, 778-781 (2020).
142. Zhang, Q., Deng, X., Ji, M., Li, Y. & Shi, Z. Hard carbon microspheres derived from resorcinol formaldehyde resin as high-performance anode materials for sodium-ion battery. *Ionics* **26**, 4523-4532 (2020).
143. Wang, X.-K. et al. Hierarchical porous hard carbon enables integral solid electrolyte interphase as robust anode for sodium-ion batteries. *Rare Met.* **39**, 1053-1062 (2020).
144. Ren, Q. et al. High-performance sodium-ion storage: multi-channel carbon nanofiber freestanding anode contrived via ingenious solvent-induced phase separation. *J. Mater. Chem. A* **8**, 19898-19907 (2020).
145. Yu, K. et al. Hyperaccumulation route to Ca-rich hard carbon materials with cation self-incorporation and interlayer spacing optimization for high-performance sodium-ion batteries. *ACS Appl. Mater. Interfaces* **12**, 10544-10553 (2020).
146. Zhang, J., Yu, B., Zhang, Y. & Wang, C. Lignin-derived hard carbon microspheres synthesized via emulsion-solvent evaporation as anode for sodium storage. *Energy Technol.* **8**, 1901423 (2020).
147. Zou, Y. et al. Low-cost lignite-derived hard carbon for high-performance sodium-ion storage. *J. Mater. Sci*. **55**, 5994-6004 (2020).
148. Chen, C. et al. Nonignorable influence of oxygen in hard carbon for sodium ion storage. *ACS Sustain. Chem. Eng.* **8**, 1497-1506 (2020).
149. Zhang, Y. et al. Optimizing the crystallite structure of lignin-based nanospheres by resinification for high-performance sodium-ion battery anodes. *Energy Technol.* **8**, 1900694 (2020).
150. Yang, L., Hu, M., Zhang, H., Yang, W. & Lv, R. Pore structure regulation of hard carbon: Towards fast and high-capacity sodium-ion storage. *J. Colloid Interface Sci.* **566**, 257-264 (2020).
151. Yang, L. et al. Salt and sugar derived high power carbon microspheres anode with excellent low-potential capacity. *Carbon* **163**, 288-296 (2020).
152. Alptekin, H. et al. Sodium storage mechanism investigations through structural changes in hard carbons. *ACS Appl. Energy Mater.* **3**, 9918-9927 (2020).
153. Xie, H. et al. Solid electrolyte interface stabilization via surface oxygen species functionalization in hard carbon for superior performance sodium-ion batteries. *J. Mater. Chem. A* **8**, 3606-3612 (2020).
154. Bobyleva, Z.V. et al. Unveiling pseudocapacitive behavior of hard carbon anode materials for sodium-ion batteries. *Electrochim. Acta* **354**, 136647 (2020).
155. Arie, A.A., Tekin, B., Demir, E. & Demir-Cakan, R. Utilization of the indonesian's spent tea leaves as promising porous hard carbon precursors for anode materials in sodium ion batteries. *Waste Biomass Valor.* **11**, 3121-3131 (2020).
156. Gao, L. et al. 2D ultrathin carbon nanosheets with rich N/O content constructed by stripping bulk chitin for high-performance sodium ion batteries. *Nanoscale* **11**, 12626-12636 (2019).
157. Xiao, W. et al. Boosting the sodium storage behaviors of carbon materials in ether-based electrolyte through the artificial manipulation of microstructure. *Nano Energy* **66**, 104177 (2019).
158. Rath, P.C. et al. Carbonaceous anodes derived from sugarcane bagasse for sodium-ion batteries. *ChemSusChem* **12**, 2302-2309 (2019).
159. Sun, D. et al. Engineering the trap effect of residual oxygen atoms and defects in hard carbon anode towards high initial Coulombic efficiency. *Nano Energy* **64**, 103937 (2019).
160. Jin, Q. et al. Experimental design and theoretical calculation for sulfur-doped carbon nanofibers as a high performance sodium-ion battery anode. *J. Mater. Chem. A* **7**, 10239-10245 (2019).
161. Sun, N. et al. Extended "Adsorption-Insertion" model: A new insight into the sodium storage mechanism of hard carbons. *Adv. Energy Mater.* **9**, 1901351 (2019).
162. Rybarczyk, M.K. et al. Hard carbon derived from rice husk as low cost negative electrodes in Na-ion batteries. *J. Energy Chem.* **29**, 17-22 (2019).
163. Li, C. et al. Heteroatom-doped hierarchically porous carbons derived from cucumber stem as high-performance anodes for sodium-ion batteries. *J. Mater. Sci.* **54**, 5641-5657 (2019).
164. Li, Y. et al. High-performance Na-ion storage of S-doped porous carbon derived from conjugated microporous polymers. *Nano-Micro Lett.* **11**, 60 (2019).
165. Sun, Y., Lu, P., Liang, X., Chen, C. & Xiang, H. High-yield microstructure-controlled amorphous carbon anode materials through a pre-oxidation strategy for sodium ion batteries. *J. Alloys Compd.* **786**, 468-474 (2019).
166. Li, D. et al. Ion- and air-tailored micro-honeycomb structures for superior Na-ion storage in coir-derived hard carbon. *New J. Chem.* **43**, 10449-10457 (2019).
167. Ren, X. et al. Lath-shaped biomass derived hard carbon as anode materials with super rate capability for sodium-ion batteries. *J. Electroanal. Chem.* **841**, 63-72 (2019).
168. Peuvot, K., Hosseinaei, O., Tomani, P., Zenkert, D. & Lindbergh, G. Lignin based electrospun carbon fiber anode for sodium ion batteries. *J. Electrochem. Soc.* **166**, A1984-A1990 (2019).
169. Wu, F. et al. Lotus seedpod-derived hard carbon with hierarchical porous structure as stable anode for sodium-ion batteries. *ACS Appl. Mater. Interfaces* **11**, 12554-12561 (2019).
170. Wang, P., Fan, L., Yan, L. & Shi, Z. Low-cost water caltrop shell-derived hard carbons with high initial coulombic efficiency for sodium-ion battery anodes. *J. Alloys Compd.* **775**, 1028-1035 (2019).
171. Zhang, X. et al. Microtubular carbon fibers derived from bamboo and wood as sustainable anodes for lithium and sodium ion batteries. *J. Porous Mater.* **26**, 1821-1830 (2019).
172. Hu, X. et al. Nitrogen-rich hierarchically porous carbon as a high-rate anode material with ultra-stable cyclability and high capacity for capacitive sodium-ion batteries. *Nano Energy* **56**, 828-839 (2019).
173. Gong, J. et al. Novel method of fabricating free-standing and nitrogen-doped 3D hierarchically porous carbon monoliths as anodes for high-performance sodium-ion batteries by supercritical CO_2_ foaming. *ACS Appl. Mater. Interfaces* **11**, 9125-9135 (2019).
174. Qiu, D. et al. Precise carbon structure control by salt template for high performance sodium-ion storage. *J. Energy Chem.* **31**, 101-106 (2019).
175. Lu, P.-R., Xia, J.-L. & Dong, X.-L. Rapid sodium-ion storage in hard carbon anode material derived from ganoderma lucidum residue with inherent open channels. *ACS Sustain. Chem. Eng.* **7**, 14841-14847 (2019).
176. Li, Y. et al. Regulating pore structure of hierarchical porous waste cork-derived hard carbon anode for enhanced Na storage performance. *Adv. Energy Mater.* **9**, 1902852 (2019).
177. Wang, K. et al. Sodium storage in hard carbon with curved graphene platelets as the basic structural units. *J. Mater. Chem. A* **7**, 3327-3335 (2019).
178. Velez, V. et al. Synthesis of novel hard mesoporous carbons and their applications as anodes for Li and Na ion batteries. *Carbon* **147**, 214-226 (2019).
179. Liu, C., Chu, J., Liu, Y., Lyu, Y. & Guo, B. The synergistic effect of carbon coating and CNTs compositing on the hard carbon anode for sodium ion batteries. *RSC Adv.* **9**, 21667-21670 (2019).
180. Meng, Q. et al. Tuning the closed pore structure of hard carbons with the highest Na storage capacity. *ACS Energy Lett.* **4**, 2608-2612 (2019).
181. Xie, F. et al. Unveiling the role of hydrothermal carbon dots as anodes in sodium-ion batteries with ultrahigh initial coulombic efficiency. *J. Mater. Chem. A* **7**, 27567-27575 (2019).
182. Lu, P., Sun, Y., Xiang, H., Liang, X. & Yu, Y. 3D amorphous carbon with controlled porous and disordered structures as a high-rate anode material for sodium-ion batteries. *Adv. Energy Mater.* **8**, 1702434 (2018).
183. Zhu, Y., Chen, M., Li, Q., Yuan, C. & Wang, C. A porous biomass-derived anode for high-performance sodium-ion batteries. *Carbon* **129**, 695-701 (2018).
184. Qin, D. et al. A sustainable route from corn stalks to N, P-dual doping carbon sheets toward high performance sodium-ion batteries anode. *Carbon* **130**, 664-671 (2018).
185. Li, J. et al. Constructing graphene-like nanosheets on porous carbon framework for promoted rate performance of Li-ion and Na-ion storage. *Electrochim. Acta* **271**, 92-102 (2018).
186. Xu, S.-D. et al. Curly hard carbon derived from pistachio shells as high-performance anode materials for sodium-ion batteries. *J. Mater. Sci.* **53**, 12334-12351 (2018).
187. Zhang, H. et al. Design advanced carbon materials from lignin-based interpenetrating polymer networks for high performance sodium-ion batteries. *Chem. Eng. J.* **341**, 280-288 (2018).
188. Zhu, Z. et al. Expanded biomass-derived hard carbon with ultrastable performance in sodium-ion batteries. *J. Mater. Chem. A* **6**, 1513-1522 (2018).
189. Wu, F. et al. Expanding interlayer spacing of hard carbon by natural K^+^ doping to boost Na-ion storage. *ACS Appl. Mater. Interfaces* **10**, 27030-27038 (2018).
190. Lu, H. et al. Exploring sodium-ion storage mechanism in hard carbons with different microstructure prepared by ball-milling method. *Small* **14**, 1802694 (2018).
191. Wang, J. et al. Facile hydrothermal treatment route of reed straw-derived hard carbon for high performance sodium ion battery. *Electrochim. Acta* **291**, 188-196 (2018).
192. Izanzar, I. et al. Hard carbons issued from date palm as efficient anode materials for sodium-ion batteries. *Carbon* **137**, 165-173 (2018).
193. Ou, J., Yang, L. & Zhang, Z. Hierarchical porous nitrogen-doped carbon material for high performance sodium ion batteries. *J. Mater. Sci. Mater. Electron.* **29**, 16478-16485 (2018).
194. Zhang, Y. et al. Honeycomb-like hard carbon derived from pine pollen as high-performance anode material for sodium-ion batteries. *ACS Appl. Mater. Interfaces* **10**, 42796-42803 (2018).
195. Xiao, L. et al. Low-defect and low-porosity hard carbon with high Coulombic efficiency and high capacity for practical sodium ion battery anode. *Adv. Energy Mater.* **8**, 1703238 (2018).
196. Wu, F. et al. Phosphorus-doped hard carbon nanofibers prepared by electrospinning as an anode in sodium-ion batteries. *ACS Appl. Mater. Interfaces* **10**, 21335-21342 (2018).
197. Hao, M. et al. Pitch-derived N-doped porous carbon nanosheets with expanded interlayer distance as high-performance sodium-ion battery anodes. *Fuel Process. Technol.* **177**, 328-335 (2018).
198. Li, W., Wang, K., Cheng, S. & Jiang, K. Self-polymerized disordered carbon enabling high sodium storage performance through expanded interlayer spacing by bound sulfur atoms. *ChemElectroChem* **5**, 3206-3212 (2018).
199. Tang, J., Barker, J. & Pol, V.G. Sodium-ion battery anodes comprising carbon sheets: Stable cycling in half- and full-pouch cell configuration. *Energy Technol.* **6**, 213-220 (2018).
200. Luo, D. et al. Surface-dominated sodium storage towards high capacity and ultrastable anode material for sodium-ion batteries. *Adv. Funct. Mater*. **28**, 1805371 (2018).
201. Zhou, C. et al. The non-ignorable impact of surface oxygen groups on the electrochemical performance of N/O dual-doped carbon anodes for sodium ion batteries. *J. Electrochem. Soc.* **165**, A1447-A1454 (2018).
202. Zhu, X., Jiang, X., Liu, X., Xiao, L. & Cao, Y. A green route to synthesize low-cost and high-performance hard carbon as promising sodium-ion battery anodes from sorghum stalk waste. *Green Energy Environ*. **2**, 310-315 (2017).
203. Wang, C. et al. Controlling pseudographtic domain dimension of dandelion derived biomass carbon for excellent sodium-ion storage. *J. Power Sources* **358**, 85-92 (2017).
204. Liu, H. et al. Creative utilization of natural nanocomposites: nitrogen-rich mesoporous carbon for a high-performance sodium ion battery. *J. Mater. Chem. A* **5**, 9572-9579 (2017).
205. Zhang, T. et al. Enhanced capacities of carbon nanosheets derived from functionalized bacterial cellulose as anodes for sodium ion batteries. *RSC Adv.* **7**, 50336-50342 (2017).
206. Ou, J., Yang, L. & Xi, X. Hierarchical porous nitrogen doped carbon derived from horn comb as anode for sodium-ion storage with high performance. *Electron. Mater. Lett.* **13**, 66-71 (2017).
207. Zhu, Y., Chen, M., Li, Q., Yuan, C. & Wang, C. High-yield humic acid-based hard carbons as promising anode materials for sodium-ion batteries. *Carbon* **123**, 727-734 (2017).
208. Ding, K. et al. Intertwined nitrogen-doped carbon nanotubes for high-rate and long-life sodium-ion battery anodes. *ChemElectroChem* **4**, 2542-2546 (2017).
209. Wang, P. et al. Kelp-derived hard carbons as advanced anode materials for sodium-ion batteries. *J. Mater. Chem. A* **5**, 5761-5769 (2017).
210. Wang, K. et al. Low-cost and high-performance hard carbon anode materials for sodium-ion batteries. *ACS Omega* **2**, 1687-1695 (2017).
211. Qiu, S. et al. Manipulating adsorption-insertion mechanisms in nanostructured carbon materials for high-efficiency sodium ion storage. *Adv. Energy Mater.* **7**, 1700403 (2017).
212. Li, Z. et al. Mechanism of Na-ion storage in hard carbon anodes revealed by heteroatom doping. *Adv. Energy Mater.* **7**, 1602894 (2017).
213. Sharma, N., Gawli, Y., Ahmad, A., Muhammed, M. & Ogale, S. Nanotubular hard carbon derived from renewable natural seed gel for high performance sodium-ion battery anode. *ChemistrySelect* **2**, 6909-6915 (2017).
214. Ou, J., Yang, L., Zhang, Z. & Xi, X. Nitrogen-doped porous carbon derived from horn as an advanced anode material for sodium ion batteries. *Micropor. Mesopor. Mater.* **237**, 23-30 (2017).
215. Zhang, T. et al. Pinecone biomass-derived hard carbon anodes for high-performance sodium-ion batteries. *RSC Adv.* **7**, 41504-41511 (2017).
216. Kim, K. et al. Tailored carbon anodes derived from biomass for sodium-ion storage. *ACS Sustain. Chem. Eng.* **5**, 8720-8728 (2017).
217. Wang, H. et al. Biomass derived hierarchical porous carbons as high-performance anodes for sodium-ion batteries. *Electrochim. Acta* **188**, 103-110 (2016).
218. Yu, Z.-L. et al. Ion-catalyzed synthesis of microporous hard carbon embedded with expanded nanographite for enhanced lithium/sodium storage. *J. Am. Chem. Soc.* **138**, 14915-14922 (2016).
219. Zhang, Y. et al. Lithium and sodium storage in highly ordered mesoporous nitrogen-doped carbons derived from honey. *J. Power Sources* **335**, 20-30 (2016).
220. Liu, H. et al. Nitrogen-doped carbon/graphene hybrid anode material for sodium-ion batteries with excellent rate capability. *J. Power Sources* **319**, 195-201 (2016).
221. Yin, L. et al. Self-assembly of disordered hard carbon/graphene hybrid for sodium-ion batteries. *J. Power Sources* **305**, 156-160 (2016).
222. Wang, P. et al. Fluorine-doped carbon particles derived from lotus petioles as high-performance anode materials for sodium-ion batteries. *J. Phys. Chem. C* **119**, 21336-21344 (2015).
223. Bommier, C., Surta, T.W., Dolgos, M. & Ji, X. New mechanistic insights on Na-ion storage in nongraphitizable carbon. *Nano Lett.* **15**, 5888-5892 (2015).
224. Jin, J., Shi, Z.-q. & Wang, C.-y. Electrochemical performance of electrospun carbon nanofibers as free-standing and binder-free anodes for sodium-ion and lithium-ion batteries. *Electrochim. Acta* **141**, 302-310 (2014).
225. He, B. et al. A generic F-doped strategy for biomass hard carbon to achieve fast and stable kinetics in sodium/potassium-ion batteries. *Chem. Eng. J.* **490**, 151636 (2024).
226. Zhao, Y. et al. Advanced structural engineering design for tailored microporous structure via adjustable graphite sheet angle to enhance sodium-ion storage in anthracite-based carbon anode. *Adv. Funct. Mater.* **34**, 2405174 (2024).
227. Wang, H. et al. Ammonium polyphosphate assisted thermal polymerization of pre-oxidized asphalt to prepare N/P double-doped hard carbon for improved sodium ion storage. *J. Energy Storage* **85**, 111045 (2024).
228. Guo, L. et al. Boosting molecular cross-linking in a phenolic resin for spherical hard carbon with enriched closed pores toward enhanced sodium storage ability. *ACS Appl. Mater. Interfaces* **16**, 27419-27428 (2024).
229. Zhang, S., Sun, N., Li, X., Soomro, R.A. & Xu, B. Closed pore engineering of activated carbon enabled by waste mask for superior sodium storage. *Energy Storage Mater*. **66**, 103183 (2024).
230. Li, X., Sun, N., Zhang, S., Soomro, R.A. & Xu, B. Closed pore structure engineering from ultra-micropores with the assistance of polypropylene for boosted sodium ion storage. *J. Mater. Chem. A* **12**, 12015-12025 (2024).
231. Ji, W.-J. et al. Free radicals trigger the closure of open pores in lignin-derived hard carbons toward improved sodium-storage capacity. *J. Energy Chem.* **94**, 551-559 (2024).
232. Mohit & Hashmi, S.A. Hard carbon anode derived from pre-treated bio-waste sugarcane bagasse for high capacity sodium-ion battery fabricated with bio-deFgradable porous polymer electrolyte. *J. Energy Storage* **83**, 110694 (2024).
233. Qiu, Y. et al. Hybrid hard carbon framework derived from polystyrene bearing distinct molecular crosslinking for enhanced sodium storage. *Carbon Energy* **6**, e479 (2024).
234. Li, Y. et al. Hydrothermally assisted conversion of switchgrass into hard carbon as anode materials for sodium-ion batteries. *ACS Appl. Mater. Interfaces* **16**, 28461-28472 (2024).
235. Zhou, Z., Wang, Z. & Fan, L. In-situ capture defects through molecule grafting assisted in coal-based hard carbon anode for sodium-ion batteries. *Chem. Eng. J.* **490**, 151428 (2024).
236. Wu, C. et al. Industrial-scale Hard carbon designed to regulate electrochemical polarization for fast sodium storage. *Angew. Chem. Int. Ed.* **63**, e202406889 (2024).
237. Zhang, X. et al. Insight into the effect of structural differences among pitch fractions on sodium storage performance of pitch-derived hard carbons. *Carbon* **226**, 119165 (2024).
238. Yuan, L. et al. Modulating intrinsic defect structure of fibrous hard carbon for super-fast and high-areal sodium energy storage. *Adv. Energy Mater.* **14**, 2400125 (2024).
239. Zhang, X. et al. Molecular engineering to regulate the pseudo-graphitic structure of hard carbon for superior sodium energy storage. *Small* **20**, 2311778 (2024).
240. Song, M. et al. New insights into the effect of hard carbons microstructure on the diffusion of sodium ions into closed pores. *Chin. Chem. Lett.* **35**, 109266 (2024).
241. Wang, H. et al. P-doped hard carbon material for anode of sodium ion battery was prepared by using polyphosphoric acid modified petroleum asphalt as precursor. *Electrochim. Acta* **477**, 143812 (2024).
242. Cheng, M.-G. et al. Preparation of enriched closed-pores hard carbon for high-performance sodium-ion batteries by the Poly(vinyl butyral) template method. *ACS Appl. Energy Mater.* **7**, 3452-3461 (2024).
243. Wang, B. et al. Resolution of the reciprocity between radical species from precursor and closed pore formation in hard carbon for sodium storage. *J. Colloid Interface Sci.* **664**, 681-690 (2024).
244. Shi, L. et al. Tailoring the dual precursors coupled hard carbon by embedding the pitch-derived graphitic domains to achieve high-performance sodium storage. *J. Power Sources* **596**, 234093 (2024).
245. Pan, G. et al. Vascular tissue-derived hard carbon with ultra-high rate capability for sodium-ion storage. *Carbon* **224**, 118955 (2024).
